# Dual Ni/photoredox-catalyzed asymmetric cross-coupling to access chiral benzylic boronic esters

**DOI:** 10.1038/s41467-021-21947-1

**Published:** 2021-03-12

**Authors:** Purui Zheng, Pan Zhou, Dong Wang, Wenhao Xu, Hepan Wang, Tao XU

**Affiliations:** 1 Shanghai Key Laboratory of Chemical Assessment and Sustainability, School of Chemical Science and Engineering, Tongji University, Shanghai, People’s Republic of China; 2 Department of Polymeric Materials, School of Materials Science and Engineering, Tongji University, Shanghai, People’s Republic of China

**Keywords:** Asymmetric catalysis, Synthetic chemistry methodology, Photocatalysis

## Abstract

The flourishing Ni/photoredox-catalyzed asymmetric couplings typically rely on redox-neutral reactions. In this work, we report a reductive cross-coupling of aryl iodides and α-chloroboranes under a dual catalytic regime to further enrich the metallaphotoredox chemistry. This approach proceeds under mild conditions (visible light, ambient temperature, no strong base) to access the versatile benzylic boronic esters with good functional group tolerance and excellent enantioselectivities.

## Abstract (web summary)

Metallaphototoredox catalysis has been rarely applied to reductive cross-couplings, in contrast to typical redox-neutral methods. Here, the authors report a mild Ni/photoredox-catalyzed reductive cross-coupling of aryl iodides and α-chloroboranes, further enriching the metallaphotoredox chemistry.

## Introduction

**Fig. 1:**
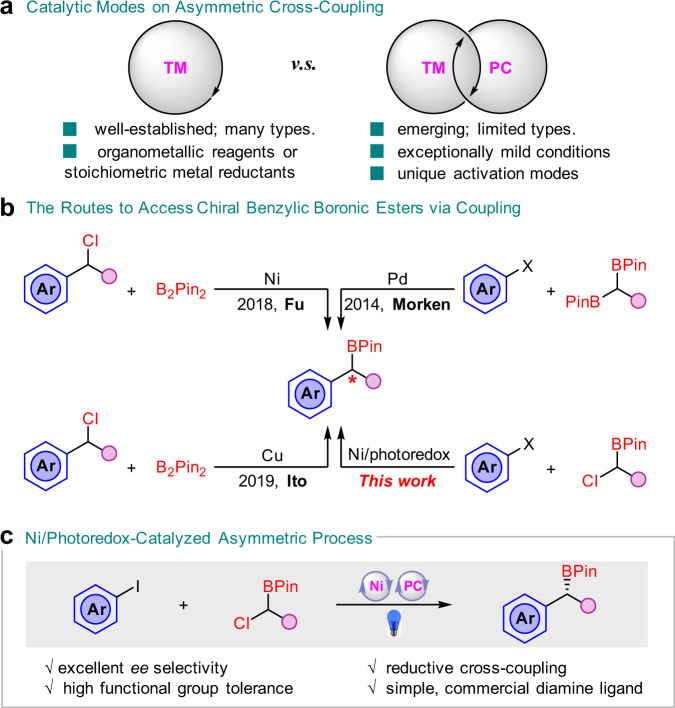
TM-catalyzed asymmetric cross-couplings. **a** Catalytic modes on asymmetric cross-coupling. **b** The routes to access chiral benzylic boronic esters via coupling. **c** Ni/photoredox-catalyzed asymmetric process. (TM: transition metal; PC: photoredox catalyst).

The merger of photoredox catalysis with transition metal catalysts, named as “metallaphotoredox”, has become a popular strategy to construct C–C bonds in the past decade^1^. This dual catalytic platform has led to many new discoveries, which were unfeasible or difficult to achieve with a single catalytic system^2–6^. Such cooperative regime, especially with nickel, provides a novel catalytic mode in asymmetric cross-couplings under exceptionally mild conditions and avoids the use of air-sensitive organometallic reagents or stoichiometric metal reductants (Fig. 1a)^7,8^. Currently, dual Ni/photoredox-catalyzed asymmetric couplings typically involve redox-neutral methods, such as Suzuki–Miyaura reactions, but few reductive cross-couplings have been reported^9–15^. We speculated that the application of dual catalytic system in asymmetric reductive cross-couplings could enable the use of various electrophiles and increase the range of accessible reaction types^16–18^. In addition, more ligand selections are likely to achieve high enantioselectivity in the absence of stoichiometric metal reductants because of circumventing the possible coordination between the metal reductant and the ligand.

However, the challenges also remain. For instance, rigorous coordinative control over several reduction cycles is needed to avoid homo-couplings or other side products. Furthermore, because of the high tendency to protodehalogenation of both halide components in the presence of reducing reagents (amines, Hantzsch ester (HEH)), the efficiency is limited. Based on these challenges, the application of this strategy upon the enantioselective reductive cross-couplings is still very less to date.

As a privileged structural class, chiral benzylic boronic esters are important precursors in organic synthesis due to their versatilities on the stereospecific conversion to many kinds of bonds^19^. In addition to the ease of handling and high air- and moisture-stability, many methods have therefore been devised to access enantioenriched benzylic boronic esters, such as hydroboration of alkenes^20–23^ or alkynes^24^, and others^25–28^. Meanwhile, asymmetric cross-couplings are also very popular (Fig. 1b). A palladium-catalyzed asymmetric coupling of bis(pinacolato)diboron (B_2_Pin_2_) with aryl halides, mainly bearing electron-donating groups, was reported by the Morken group^29^. A nickel- and a copper-catalyzed enantioselective borylation of secondary benzylic chlorides with B_2_Pin_2_, were published by the Fu^30^ and Ito^31^ group, respectively. However, in these cases, the enantiomeric excess (*ee*) values of the products were generally not ideal (<87%). Although several racemic couplings under reductive conditions have been developed^32–36^, to the best of our knowledge, asymmetric reductive cross-coupling with the dual catalytic system constitutes a potentially powerful yet unreported route to construct chiral benzylic boronic esters. Herein, we report a Ni/photoredox-catalyzed asymmetric coupling with broad substrate scopes and excellent enantioselectivities (basically above 90% *ee*) by using a simple, commercially available ligand (Fig. 1c).

## Results

### Reaction conditions development

We commenced our investigation of this asymmetric coupling with the reaction between 4-iodobenzotrifluroride (**1a**) and α-chloroborane (**2a**) with dual Ni/photoredox catalysts^37^. Systematic evaluation of all the reaction parameters indicated that the transformation proceeded smoothly under the conditions listed in entry 1, Table 1, to give the product **3a** in 94% yield with 93% *ee* (Table 1, entry 1). Other Ni catalysts gave lower conversions (entries 2–3). The use of other ligands (**L2**, **L3**), especially the bis(oxazoline) ligand **L4**, which is particularly common in cross-couplings^30^, resulted in diminished outcomes (entry 4–6). Examination of other photocatalysts or solvents identified a rather significant decrease in yields and/or enantioselectivities. (entries 7–10, see supporting information). Although the exact reasons remain elusive, the mixed solvent system possibly increased the dielectric constant of the reaction medium^30,38^, thus improving the catalytic efficiency and suppressing the protodehalogenation product. A trace amount of product was detected in the absence of Et_3_N and a low yield was obtained with Li_2_CO_3_ (entries 11–12). Considering Et_3_N can act as a reductant in photoredox-catalyzed reactions, it is reasonable that the reaction still worked without HEH, albeit with lower efficiency (entry 13). The reaction can proceed for one or fewer catalytic cycles without the photocatalyst, which may suggest a mechanistic scenario more complex than previous proposition (entry 14). Control reactions performed in the absence of Ni catalyst or light resulted in no detectable product formation, confirming the essential role of each of these components in the dual catalytic process (entry 15).

**Table 1:** Optimization of reaction conditions.


**Entry**^**a**^	**Changes**	**Yield (%)**	***Ee*** **(%)**
1	No change	94 (86^b^)	93
2	Using Ni(cod)_2_	48	51
3	Using NiI_2_	43	90
4	L2 instead of L1	93	87
5	L3 instead of L1	19	91
6	L4 instead of L1	3	n.d.
7	[Ir(dFCF_3_ppy)dtbbpy]PF_6_ as PC	43	83
8	Ru(bpy)_3_Cl_2_6H_2_O as PC	12	30
9	DME as solvent	45	94
10	DMA as solvent	42	82
11	No Et_3_N	<5	n.d.
12	Li_2_CO_3_ instead of TEA	36	85
13	No HEH	45	78
14	No 4CzIPN	7	90
15	No Ni or no light	0	---

^a^Standard conditions: **1a** (1.6 equiv.), **2a** (0.2 mmol), NiBr_2_·DME (10 mol%), **L1** (12 mol%), 4CzIPN (1 mol%), HEH (2 equiv.), TEA (5 equiv.) DME/DMA (4 mL, *v*/*v* = 5/1), Blue LEDs (30 W), 18–25 ^o^C, 8–10 h. Yields were determined by GC with ^*n*^dodecane as an internal standard. The *ee* values were determined by HPLC.

^b^Isolated yield was given in the parenthesis.

**Fig. 2:**
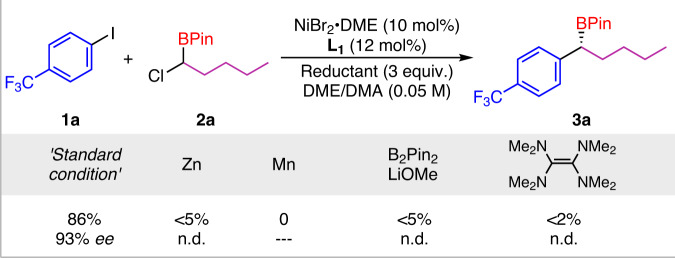
The reaction with other reductants. Comparison reaction with other reductive conditions.

In addition, perhaps most notable, the comparison reactions with other common reductive systems were tested to identify the difference and powerful efficiency of this Ni/photoredox-catalyzed coupling (Fig. 2). The results showed that whether the metal reductants Zn/Mn or the organic reagents B_2_Pin_2_ and tetrakis(dimethylamino)ethylene (TDAE) gave only traces of the product or no product, thus further strengthening the high superiority of the dual catalytic method on this C–Cl bond transformation.

### Substrate scope

Having developed the enantioselective catalytic reaction of this cross-coupling, we sought to investigate the generality and utility of this dual Ni/photoredox-catalyzed method (Some products were found to be prone to decompose in column, thus isolated yields after oxidation were given. See Table 2). We firstly tested various aryl iodides with either electron-withdrawing or electron-donating groups (the reaction with *p*-CF_3_-C_6_H_4_Br gave the product **3a** in 21% yield with 91% *ee* under the standard conditions). The excellent enantioselectivities and satisfactory yields were generally given whether the substituents on the *para-*, *meta-*, or *ortho*- positions, in an effort to showcase the mild nature of this transformation. The electron-poor substrates obviously facilitated the couplings, as demonstrated with compounds **3a**–**3f**. The reactions with strong electron-rich aryl iodides (**3i**) led to a decreased but still acceptable results, thus serving to highlight the complementary and discrepant nature of this method compared to the established method with palladium where the two-electron transmetalation regime was involved^29^. The chemoselectivity with different electrophiles was studied under the current conditions. The exclusive selection on aryl iodide was observed in the presence of aryl chloride or triflate group (**3x**, **3y**, **3z**) while a mixture was obtained with 1-bromo-4-iodobenzene as a substrate, possibly due to the existing reactivity of aryl bromides. It is worth mentioning that, this method can afford high enantioselectivities not only for the bulky or *ortho*-substituted aryl substrates as previous reports^29–31^, but also for the less sterically hindered components (such as **3g**, **3h**), thus expanding its application in organic synthesis.

**Table 2:** Scope of Ni/photoredox-catalyzed coupling of aryl iodides with α-chloroboranes^a^.

^a^Reaction conditions: **1** (1.6 equiv.), **2** (0.2 mmol), NiBr_2_·DME (10 mol%), **L1** (12 mol%), 4CzIPN (1 mol%), HEH (2 equiv.), TEA (5 equiv.), DME/DMA (4 mL, *v*/*v* = 5/1), Blue LEDs (30 W), 18–25 ^o^C, 8–10 h. Isolated yields were given. The *ee* values were determined of the corresponding alcohol after oxidation by HPLC.

^b^Isolated yield and *ee* of the corresponding alcohol after oxidation were given.

^c^**2a** (2.0 equiv.).

The scope of α-chloroborane was then explored in the dual catalytic system. Overall, a wide range of the chloroboronic esters could be smoothly transferred to the corresponding products with very high *ee* values. For the complex molecules (**3nn**-**3pp**), this Ni/photoredox-catalyzed coupling was also suitable, thus demonstrating the potential of this methodology in the late-stage functionalization. The reaction also exhibited comparable efficiency on a larger scale, as the products (**3a**, **3y**) were isolated in good yields and undiminished *ee* on a 1 mmol scale. In addition, Table 2 also highlighted the robustness on functional group compatibility of this approach. The trifluoromethyl (**3a**, **3v**), trifluoromethoxy (**3b**, **3p**), ester (**3c**, **3hh**, **3ii**), amide (**3f**), nitrile (**3e**, **3n**, **3dd**), aryl halides (**3d**, **3w**, **3x**), triflate (**3z**), phenol (**3m**), ether (**3i**, **3ee**), alkyl chloride (**3ff**, **3gg**), alkenyl (**3mm**) groups were all compatible under the optimized conditions.

### Competing and comparison reactions

**Fig. 3:**
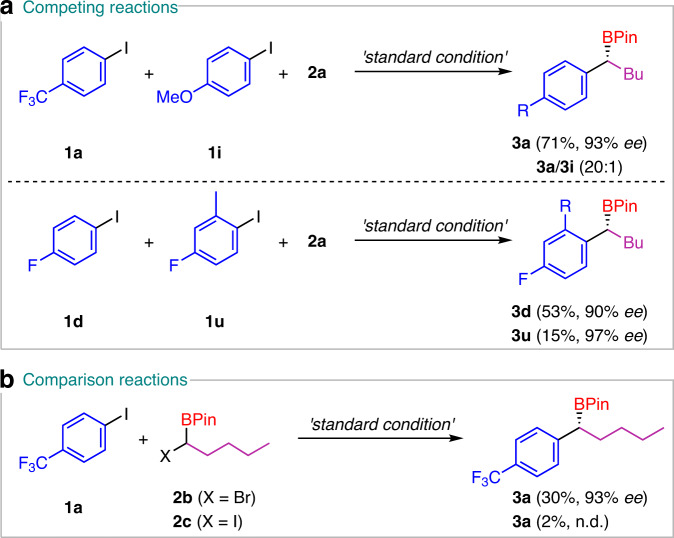
Competing and comparison reactions. **a** The reaction with aryl halides with different properties. **b** Test on the α-bromo- or α-iodoborane.

To clarify the features on this dual catalytic system, competing reactions were conducted. Although both electron-rich and electron-poor aryl iodides can participate in this transformation, the substrate with an electron-withdrawing group favored when both are present, as evidenced by isolation of product **3a** in Fig. 3a. Steric effect was also investigated. When **1d** and **1u** were used simultaneously, the less sterically hindered component gave the main product **3d**.

α-Bromo- and α-iodoboranes, which are often used in Ni-catalyzed cross-couplings^34,39^, were also tested (Fig. 3b). Surprisingly, when α-bromoborane was employed, the target product was isolated in only 30% yield with a similar enantioselectivity. A trace amount of product was observed in GC when the more active α-iodoborane was used. The protodehalogenation product was the main product in these two reactions. These results show the importance of concerted rates of reductive processes in this dual catalytic system and the difference from the single catalytic regime.

### Further applications

**Fig. 4:**
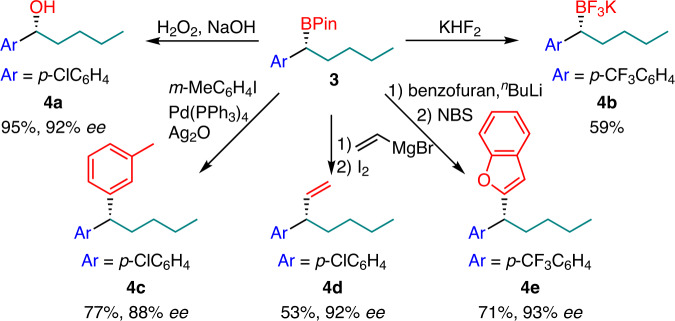
Further application of the products. Derivatization of the chiral benzylic boronic esters.

One advantage of this method is that the resulting molecules contain a versatile boron group, which is convenient for further functionalization to construct many other chiral bonds. We demonstrated this in a series of examples in Fig. 4. For example, the boranes can be oxidized to chiral alcohol (**4a**) or transformed to the potassium trifluoroborate salt (**4b**) in good yields. The chiral 1,1-diarylalkanes, which are widely useful but not easily accessible, can be conveniently obtained from these products with a Pd-catalyzed coupling reaction (**4c**). Meanwhile, some other C_sp3_–C_sp2_ bonds were readily formed to introduce alkenyl or heterocycles according to the reported methods (**4d**, **4e**)^40,41^.

### Mechanistic studies

**Fig. 5:**
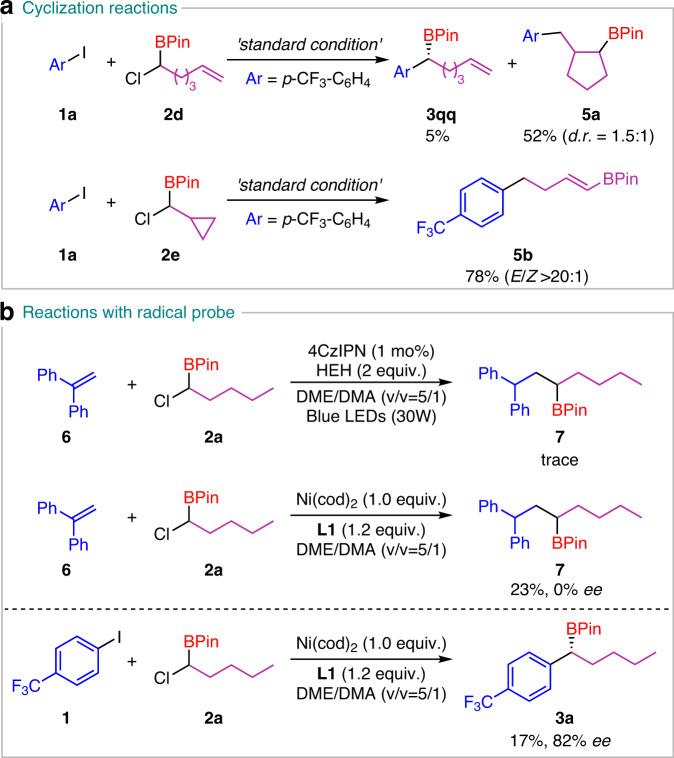
Mechanistic studies. **a** Cyclization reactions. **b** Reactions with radical probe.

**Fig. 6:**
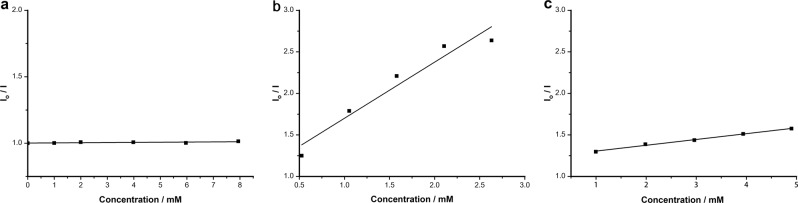
Stern–Volmer quenching experiments. **a** With substrate **2a**. **b** With HEH. **c** With Et_3_N.

To give some insights into the mechanism of the coupling, some experiments were performed (Fig. 5). The substrate **2d** with an alkene chain was investigated. The cyclization product **5a** was isolated in 52% yield and direct cross-coupling **3qq** was only detected in 5% GC yield, indicating the production of the radical intermediate. The same conclusion is also deduced from the reaction with radical clock substrate **2e** (Fig. 5a). Although it was reported that an alkyl halide can be reduced to a radical species via a single-electron reduction by photocatalysts^42^, herein, the luminescence quenching experiments indicated that HEH and Et_3_N are more likely to quench the excited state of 4CzIPN (Fig. 6). The reactions with a radical probe also proved this point (Fig. 5b). In the absence of Ni/**L1**, the α-chloroborane can not react with 1,1-diphenylethylene **6** to give any radical-trapped product **7**. However, in the stoichiometric studies with Ni(cod)_2_/**L1**, product **7** can be obtained in 30% yield with no enantioselectivity. Hence, the free alkyl radical should be generated from α-chloroborane by the reduction of Ni species rather than 4CzIPN^43–46^.

**Fig. 7:**
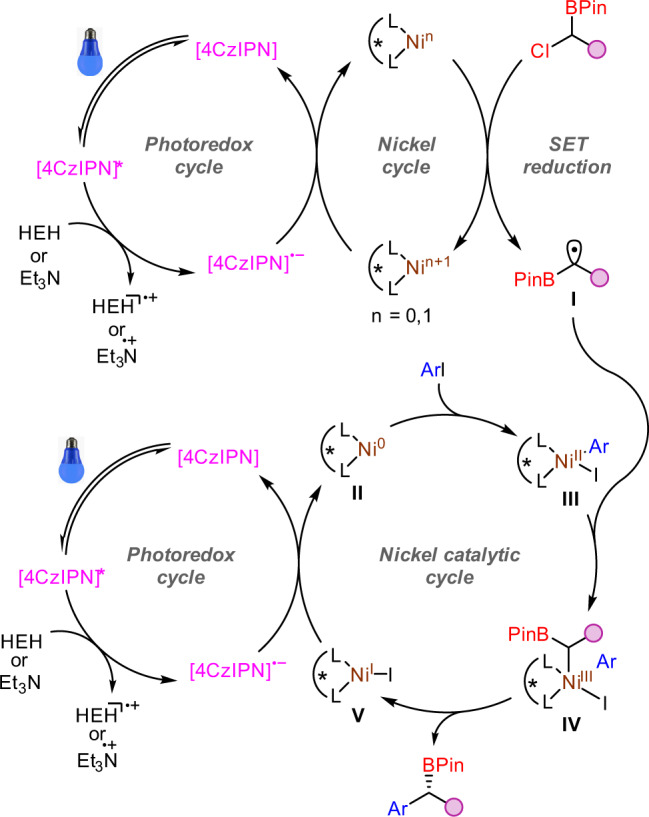
Proposed mechanism. A proposed catalytic cycle for the dual catalytic coupling.

Based on these experiments, a proposed mechanism is shown in Fig. 7. Light excitation of photocatalyst 4CzIPN generates the photoexcited [4CzIPN]*, which is reduced by HEH or Et_3_N to the reduced ground-state photocatalyst [4CzIPN]^·-^. Then, combination with the nickel cycle, it is responsible to produce a boron-stabilized radical **I**. Meanwhile, the oxidative addition of aryl iodide to the ligated Ni(0) **II** forms a Ni(II) species **III**. Then oxidation by radical **I** accesses the high-valent Ni(III) intermediate **IV**, and subsequent reductive elimination affords the product and releases Ni(I) species **V**. Reduction of **V** would regenerate the Ni(0) and 4CzIPN catalyst, closing the dual catalytic cycle. However, at this moment, we can not exculde another catalytic cycle in which the alkyl radical is trapped by Ni(0), then the alkyl Ni(I) species is oxidated by aryl hailde^47,48^.

## Discussion

In summary, herein a dual Ni/photoredox-catalyzed asymmetric reductive cross-coupling of aryl iodides with α-chloroboranes was developed. The mild conditions enable the process to give excellent enantioselectivities and accommodate a broad palette of architectures with many functional groups. Mechanistic studies suggest the alkyl radical is probably generated via reduction by nickel. The resulting chiral benzylic boronic esters, which have important versatilities in organic synthesis demonstrated the high potential of this transformation.

## Methods

### General procedure for the dual catalytic coupling

In a N_2_-filled glovebox, an oven-dried 10-mL Schlenk tube containing a Teflon stir bar was charged with 4CzIPN (0.002 mmol), NiBr_2_·DME (0.02 mmol), **L1** (0.024 mmol), HEH (0.4 mmol), TEA (1.0 mmol). Then the tube was sealed with a septum and taken out. DME/DMA (*v*/*v* = 5/1, 4 mL) were added via syringe under N_2_ atmosphere. After stirring for 30 min at room temperature, aryl halide (0.32 mmol) and α-chloroborane (0.2 mmol) were added. The reaction mixture was stirred and irradiated under blue light (*λ* = 450–455 nm) for 8–10 h, while the temperature was controlled at 18–25 °C. Upon completed, the mixture was diluted with EtOAc and quenched with water. The aqueous solution was extracted with EtOAc three times. The combined organic layers were dried, concentrated and purified by flash column chromatography using PE/EtOAc as the eluent to afford the coupling product. The *ee* value was determined by high-performance liquid chromatography analysis using the corresponding alcohol after oxidation of the product.

## Supplementary information

Supplementary Informaiton

## Data Availability

The authors declare that the data supporting the finding of this study are available within the paper and its Supplementary Information file. The experimental procedures and characterization of all new compounds are provided in Supplementary Information.
