# Supplementary material for: Dual Ni/photoredox-catalyzed asymmetric cross-coupling to access chiral benzylic boronic esters
Source: Nat Commun. 2021 Mar 12;12:1646. doi: 10.1038/s41467-021-21947-1 (PMC7954797; doi:10.1038/s41467-021-21947-1)
Supplement: Supplementary file 1 — Supplementary Informaiton [file 41467_2021_21947_MOESM1_ESM.pdf]

Dual Ni/Photoredox-Catalyzed Asymmetric Cross-Coupling to Access Chiral Benzylic Boronic Esters

*Purui Zheng, Pan Zhou, Dong Wang, Wenhao Xu, Hepan Wang, and Tao XU**

Supplementary Information

Supplementary Note 1 .... S2

Supplementary Table, Methods and Figures S3

Optimizaiton tables.. S3

General methords S6

Control and mechanistic eperiments S14

The characterization of the compounds S22

Spectra data of the compounds S91

Supplementary References.. S205

1. Supplementary Note 1

^{1}H and ^{13}C spectra were recorded on a Bruker Avance 400, 600 spectrometers, and $CDCl_3$ was purchased from J&K. Chemical shifts are given in ppm with the internal standards as TMS (0 ppm for ^{1}H) and $CDCl_3$ (77.0 ppm for ^{13}C). Flash column chromatography was performed on silica gel 60 (particle size 200-400 mesh ASTM, purchased from Yantai, China) and eluted with petroleum ether/ethyl acetate. GC spectra were recorded on Agilent Technologies 7890A spectrometer; GC-MS spectra were conducted on Shimadzu GC-MS-QP2010 SE W spectrometer; HPLC analysis was performed on Thermo Fisher Ultimate 3000 spectrometer; Optical rotation was measured using a Rudolph AUTOPOL VI polarimeter; High resolution mass spectra HRMS-ESI were obtained from a Bruker micrOTOF-II instrument;

The blue LEDs were purchased from www.taobao.com. The reaction tubes were positioned 4-6 cm from the LEDs (**Supplementary Figure S1**), and the temperature was controlled between 19 ^{o}C and 25 ^{o}C using fan cooling.

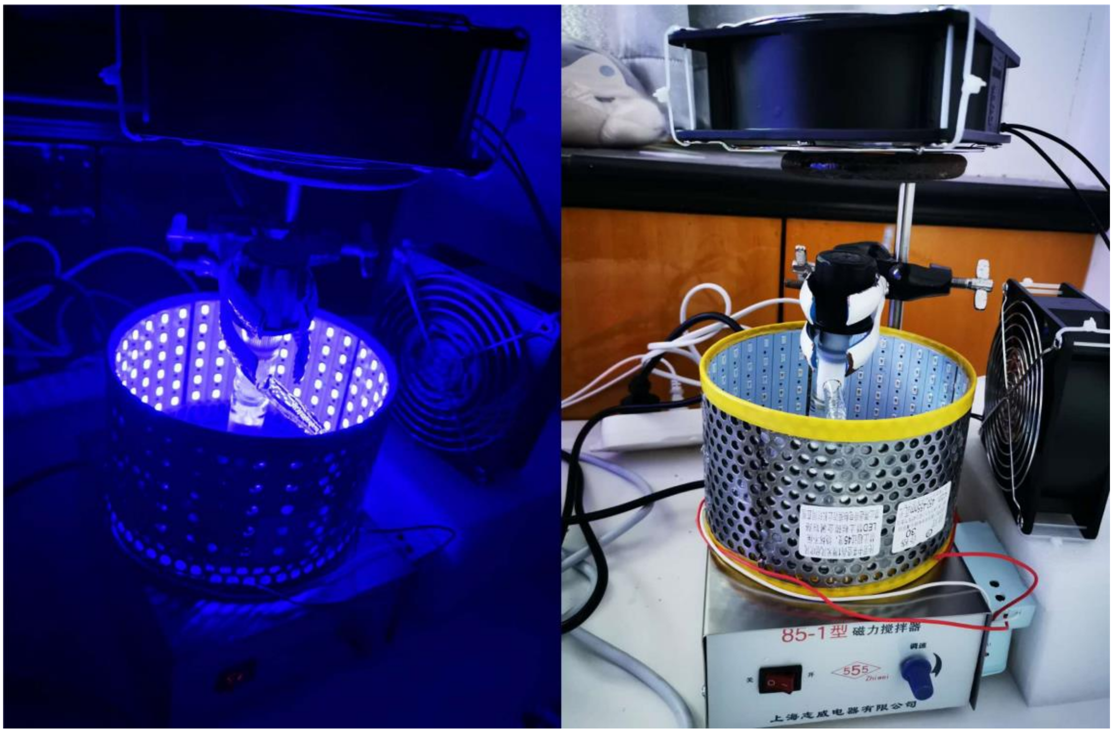

Supplementary Figure S1. Photochemical Setup

Unless otherwise noted, all other reagents and starting materials were purchased from commercial sources and used without further purification. Solvents were dried and purified according to the procedure from ‘Purification of Laboratory Chemicals book’.

2 Supplementary Tables, Methods and Figures

2.1 Optimization table

General procedure A for Reaction Optimization: An oven-dried 10-mL Schlenk tube containing a Teflon stir bar was charged with 4CzIPN (0.002 mmol, 1 mol%), Ni-catalyst (0.02 mmol, 10 mol%), ligand (0.024 mmol, 12 mol%), HEH (0.4 mmol, 2 equiv.), TEA (1.0 mmol, 5 equiv.). Then the tube was sealed with a septum and taken out of the N_2-filled glovebox. The solvent (4 mL) was added via syringe under N_2 atmosphere and the reaction mixture was stirred for 30 min at room temperature. Then aryl halide **1a** (0.32 mmol, 1.6 equiv.) and **2a** (0.2 mmol) were added via micro-syringes. Once added, the tube was closed again, and the reaction mixture was stirred and irradiated under blue light (λ = 450-455 nm) for 8-10 hours, while the temperature was controlled at approximately 20 ^{o}C by cooling with fans and air-conditioner. Upon completed, the mixture was diluted with EtOAc and n-dodecane as a GC internal standard was added. The yield was determined by GC analysis, and the *ee* value was obtained by HPLC analysis using the relevant alcohol after purification and oxidation.

Supplementary Table 1. Screening results on ligand

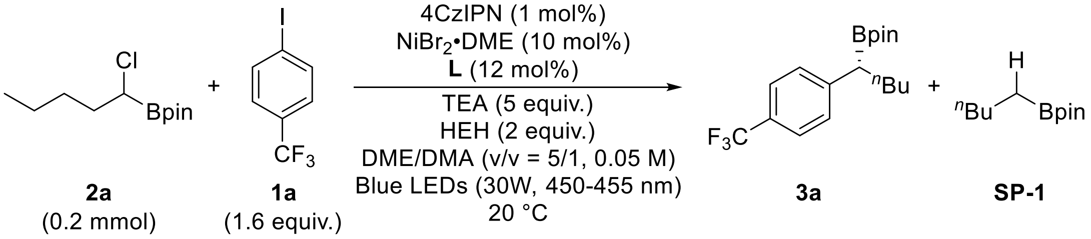

Entry	Ligand	Yield	SP-1	Ee
**1**	**L1**	94% (86% isolated yield）	9%	93%
**2**	**L2**	93%	11%	87%
**3**	**L3**	19%	59%	91%
**4**	**L4**	39%	45%	44%
**5**	**L5**	3%	38%	n.d.

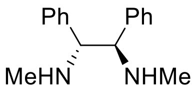

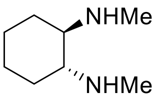

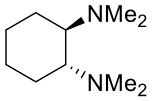

L1 **L2** **L3**

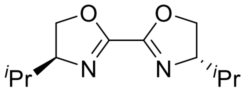

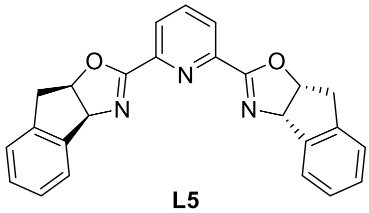

L4 **L5**

Supplementary Table 2. Screening results on Ni-source

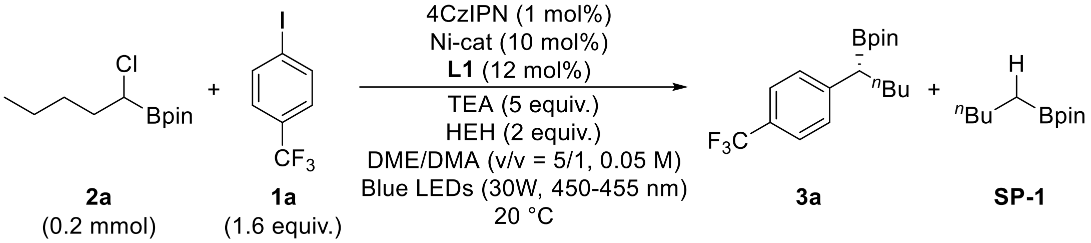

Entry	Ni-cat	Yield	SP-1	Ee
**1**	$NiBr_2\cdot DME$	94%	9%	93%
**2**	NiI_2	43%	37%	90%
**3**	$Ni(cod)_2$	48%	20%	51%
**4**	$NiCl_2\cdot glyme$	45%	13%	95%

Supplementary Table 3. Screening results on solvents

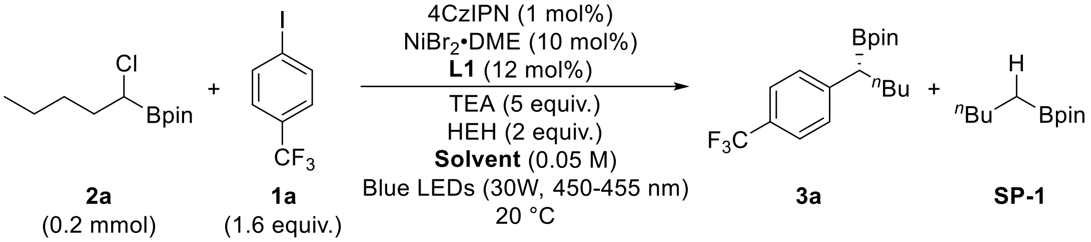

Entry	Solvent	Yield	SP-1	Ee
**1**	DMA	42%	52%	82%
**2**	DME	45%	8%	94%
**3**	DME/DMA (v/v = 5/1)	94%	9%	93%
**4**	THF/DMA (v/v = 5/1)	74%	10%	95%

Supplementary Table 4. Control reactions

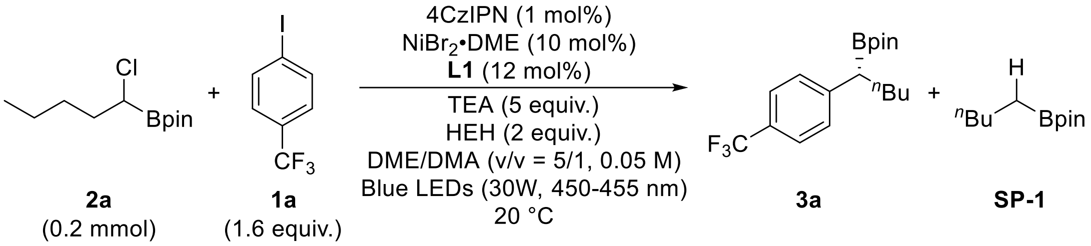

Entry	Changes	Yield	SP-1	Ee
**1**	No changes	94%	9%	93%
**2**	$[Ir(dF(CF_3)ppy)_2(dtbbpy)]PF_6$	43%	30%	83%
**3**	$Ru(bpy)_3Cl_2{\cdot}6H_2O$	12%	27%	30%
**4**	5% Ni, 6% **L1**, 0.5% 4CzIPN	90%	10%	93%
**5**	Li_2CO_3	36%	50%	85%
**6**	K_2HPO_4	36%	49%	87%
**7**	No HEH	45%	4%	78%
**8**	No TEA	<5%	71%	n.d.
**9**	No light	0	2%	-
**10**	No 4CzIPN	7%	7%	90%
**11**	No Ni	0	27%	-

2.2 General methords

General procedure B for arylation of α-chloro alkyl boronic esters:

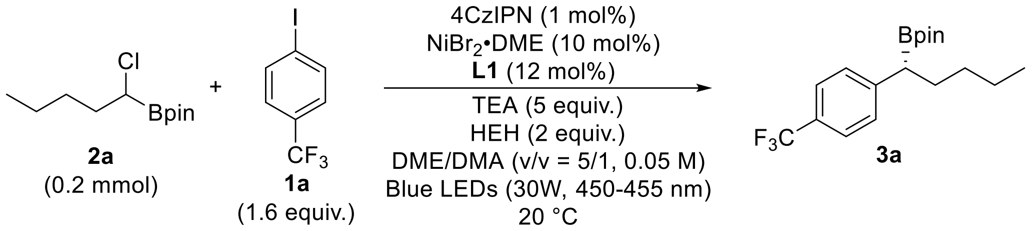

An oven-dried 10-mL Schlenk tube containing a Teflon stir bar was charged with 4CzIPN (0.002 mmol, 1 mol%), $NiBr_2$·DME (0.02 mmol, 10 mol%), **L1** (0.024 mmol, 12 mol%), HEH (0.4 mmol, 2 equiv.), TEA (1.0 mmol, 5 equiv.). Then the tube was sealed with a septum and taken out of the N_2-filled glovebox. DME/DMA (v/v = 5/1, 4 mL) were added via syringe under N_2 atmosphere and the reaction mixture was stirred for 30 min at room temperature. Then aryl halide **1a** (0.32 mmol, 1.6 equiv.) and **2a** (0.2 mmol) were added via micro-syringes. Once added, the tube was closed again, and the reaction mixture was stirred and irradiated under blue light (λ = 450-455 nm) for 8-10 hours, while the temperature was controlled at approximately 20 ^{o}C by cooling with fans and air-conditioner. Upon completed, the mixture was diluted with EtOAc and quenched with water. The aqueous solution was extracted with EtOAc three times. The combined organic layers were dried over anhydrous Na_2SO_4, filtered through Celite, and concentrated *in vacuo*. The residues were purified by silica gel column chromatography with a gradient eluent of petroleum ether/ethyl acetate affording the product **3a** (58.8 mg, 86% yield, 93% *ee*). The *ee* value was determined by HPLC analysis using the relative alcohol after oxidation of the product.

General procedure C for scale-up reactions:

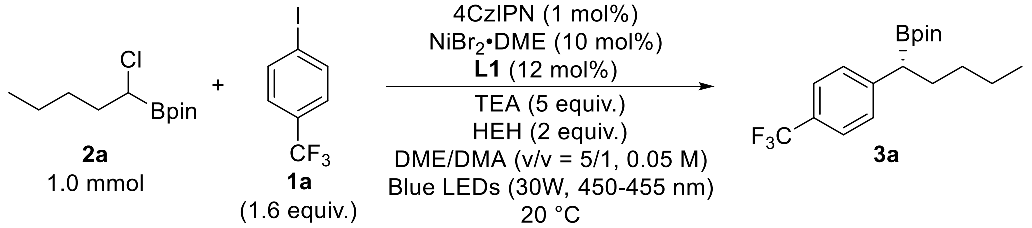

An oven-dried 50-mL Schlenk tube containing a Teflon stir bar was charged with 4CzIPN (0.01 mmol, 7.9 mg, 1 mol%), $NiBr_2$·DME (0.1 mmol, 30.8 mg, 10 mol%), **L1** (0.12 mmol, 28.8 mg, 12 mol%), HEH (2.0 mmol, 504 mg, 2 equiv.), TEA (5.0 mmol, 505 mg, 5 equiv.). Then the tube was sealed with a septum and taken out of the N_2-filled glovebox. DME/DMA (v/v = 5/1, 20 mL) were added via syringe under N_2 atmosphere and the reaction mixture was stirred for 30 min at room temperature. Then aryl halide **1a** (1.6 mmol, 435.2 mg, 1.6 equiv.) and **2a** (1.0 mmol, 232 mg) were

added via micro-syringes. Once added, the tube was closed again, and the reaction mixture was stirred and irradiated under blue light (λ = 450-455 nm) for 16 hours, while the temperature was controlled at approximately 20 °C by cooling with fans and air-conditioner. Upon completed, the mixture was diluted with EtOAc and quenched with water. The aqueous solution was extracted with EtOAc three times. The combined organic layers were dried over anhydrous Na_2SO_4, filtered through Celite, and concentrated *in vacuo*. The residues were purified by silica gel column chromatography with a gradient eluent of petroleum ether/ethyl acetate affording the product **3a** (208.6 mg, 61%, 93% *ee*). The *ee* value was determined by HPLC analysis using the relative alcohol after oxidation of the product.

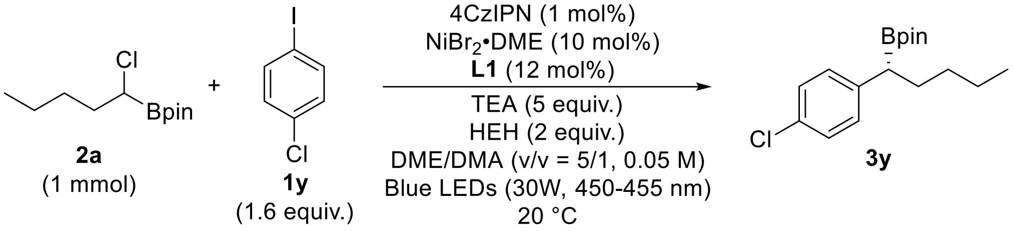

An oven-dried 50-mL Schlenk tube containing a Teflon stir bar was charged with 4CzIPN (0.01 mmol, 7.9 mg, 1 mol%), $NiBr_2$·DME (0.1 mmol, 30.8 mg, 10 mol%), **L1** (0.12 mmol, 28.8 mg, 12 mol%), HEH (2.0 mmol, 504 mg, 2 equiv.), TEA (5.0 mmol, 505 mg, 5 equiv.). Then the tube was sealed with a septum and taken out of the N_2-filled glovebox. DME/DMA (v/v = 5/1, 20 mL) were added via syringe under N_2 atmosphere and the reaction mixture was stirred for 30 min at room temperature. Then aryl halide **1y** (1.6 mmol, 380.8 mg, 1.6 equiv.) and **2a** (1.0 mmol, 232 mg) were added via micro-syringes. Once added, the tube was closed again, and the reaction mixture was stirred and irradiated under blue light (λ = 450-455 nm) for 16 hours, while the temperature was controlled at approximately 20 °C by cooling with fans and air-conditioner. Upon completed, the mixture was diluted with EtOAc and quenched with water. The aqueous solution was extracted with EtOAc three times. The combined organic layers were dried over anhydrous Na_2SO_4, filtered through Celite, and concentrated *in vacuo*. The residues were purified by silica gel column chromatography with a gradient eluent of petroleum ether/ethyl acetate affording the product **3y** (206.4 mg, 67%, 92% ee). The *ee* value was determined by HPLC analysis using the relative alcohol after oxidation of the product.

General procedure D for synthesis of the starting materials:

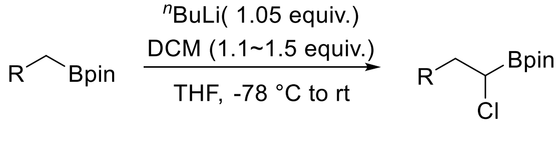

According to the reference[S1], an oven-dried 100 mL screw-cap round-bottom Schlenk tube equipped with a Teflon stir bar was added with dichloromethane (16 mL, 1.5 equiv.) and anhydrous THF (20 mL) under N_2 atmosphere, then the tube was

cooled to -100 °C in a liquid N_2/EtOH bath followed by dropwise addition of ^{n}BuLi (6.6 mL, 10.5 mmol, 1.6 M in Hexane) via a syringe. After stirring for 30 min, a solution of alkyl pinacol boronate ester (10 mmol) in THF (5 mL) was added to the reaction at the same temperature. Then the resultant mixture was allowed to slowly worm to room temperature and stirred overnight. Once finished, a large amount of dichloromethane was added to precipitate LiCl and the solution was filtered and concentrated. The crude material was purified by silica gel column chromatography with a gradient eluent of petroleum ether/ethyl acetate affording the relative product.

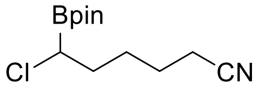

2dd

^{1}H NMR (400 MHz, Chloroform-*d*) δ 3.42 (dd, *J* = 8.4, 6.4 Hz, 1H), 2.38 (t, *J* = 6.8 Hz, 2H), 1.92-1.79 (m, 2H), 1.76-1.59 (m, 3H), 1.63-1.52 (m, 1H), 1.29 (s, 12H); ^{13}C NMR (101 MHz, Chloroform-*d*) δ 119.3, 84.4, 32.9, 26.2, 24.8, 24.4, 24.4, 16.9; HRMS: m/z (ESI) calculated $[M+Na]^+$:280.1246, found: 280.1250.

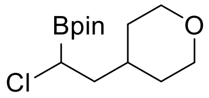

2ee

^{1}H NMR (400 MHz, Chloroform-*d*) δ 4.05-3.80 (m, 2H), 3.51 (dd, *J* = 11.6, 5.2 Hz, 1H), 3.39 (td, *J* = 11.6, 2.2 Hz, 2H), 1.90-1.77 (m, 2H), 1.74-1.61 (m, 2H), 1.57 (d, *J* = 13.0 Hz, 1H), 1.38-1.20 (m, 2H), 1.29 (s, 12H); ^{13}C NMR (101 MHz, Chloroform-*d*) δ 84.4, 67.9 (d, *J* = 2.5 Hz), 40.3, 33.2, 32.3, 31.6, 24.5, 24.5; HRMS: m/z (ESI) calculated $[M+Na]^+$:239.1399, found: 239.1395.

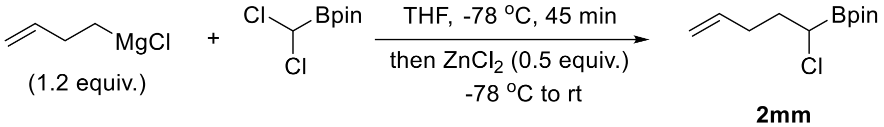

2mm

According to the reference[S2], an oven-dried 50 mL screw-cap round-bottom Schlenk tube equipped with a Teflon stir bar was added with 2-(dichloromethyl)-4,4,5,5-tetramethyl-1,3,2-dioxaborolane (2.1 g, 10 mmol) and anhydrous THF (40 mL) under N_2 atmosphere, then the tube was cooled to -78 °C followed by dropwise addition of the Grignard reagent (12 mL, 12 mmol, 1.2 equiv., 1 M in hexane) via a syringe. After stirring for 45 min, a solution of $ZnCl_2$ (5 mmol, 0.5 equiv.) in 5 mL THF was added in one portion. The resultant mixture was allowed to slowly worm to room temperature and stirred overnight. Upon completed, a 40 mL saturated NH_4Cl aqueous solution was added to quench the reaction. The aqueous solution was extracted with EtOAc three times. The combined organic layers were dried over anhydrous Na_2SO_4, filtered and concentrated. The pure **2mm** was obtained through distillation of the crude material. ^{1}H NMR (400 MHz, Chloroform-*d*) δ 5.79

(ddt, J = 17.0, 10.0, 6.6 Hz, 1H), 5.07 (dd, J = 17.0, 1.8 Hz, 1H), 5.00 (dd, J = 10.0, 1.8 Hz, 1H), 3.44 (dd, J = 8.4, 6.6 Hz, 1H), 2.33-2.13 (m, 2H), 2.02-1.80 (m, 2H), 1.29 (s, 12H); ^{13}C NMR (101 MHz, Chloroform-*d*) δ 137.2, 115.6, 84.4, 33.1, 31.3, 24.6, 24.5; HRMS: m/z (EI) calculated $[M]^+$: 230.1239, found: 230.1245.

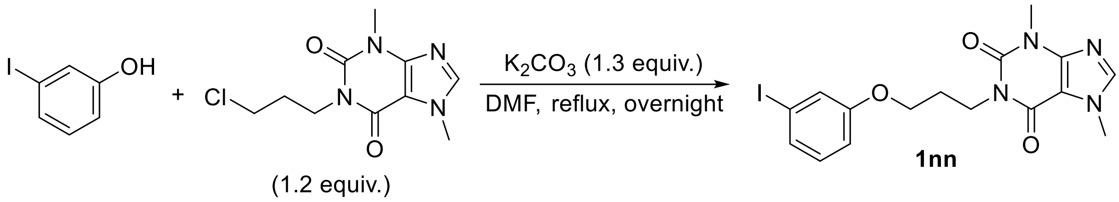

According to the reference[S3], a 100 mL round-bottom flask equipped with a stirring Teflon stir bar was charged with 3-iodophenol (10mmol), 1-(3-Chloropropyl)-3,7-dimethyl-1H-purine-2,6-(3H,7H)-dione (12 mmol), K_2CO_3 (13 mmol) and DMF (40 mL). The reaction solution was refluxed overnight. Upon finished, 50 mL water was added to quench the reaction and the aqueous solution was extracted with EtOAc three times. The combined organic layers were dried over anhydrous Na_2SO4 and concentrated *in vacuo*. The crude material was purified by silica gel column chromatography with a gradient eluent of petroleum ether/ethyl acetate affording the relative product **1nn**. ^{1}H NMR (400 MHz, Chloroform-*d*) δ 7.68 (d, J = 8.4 Hz, 2H), 7.56 (d, J = 7.4 Hz, 1H), 7.48 (d, J = 8.4 Hz, 2H), 7.44 (s, 1H), 7.12-7.01 (m, 3H), 6.89 (d, J = 9.0 Hz, 1H), 6.70 (dd, J = 9.0, 2.4 Hz, 1H), 3.89 (s, 2H), 3.84 (s, 3H), 2.45 (s, 3H); ^{13}C NMR (101 MHz, Chloroform-*d*) δ 168.9, 168.3, 156.1, 150.9, 139.4, 136.3, 135.1, 133.7, 131.2, 130.8, 130.7, 130.6, 130.4, 129.2, 121.0, 115.0, 111.8, 111.6, 101.1, 93.5, 55.7, 30.4, 13.4; HRMS: m/z (ESI) calculated $[M+H]^+$: 441.0418, found: 441.0430.

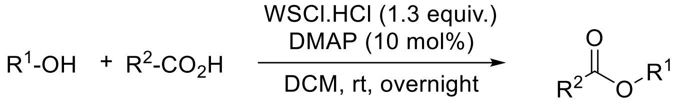

According to the reference[S4], a 100 mL round-bottom flask equipped with a stirring Teflon stir bar was charged with carboxylic acid (1.2 mmol), DMAP (1.0 mmol, 10 mol%) and DCM (40 mL). WSCl·HCl (13 mmol, 1.3 equiv.) was carefully added to the stirring solution. Subsequently alcohol (10 mmol) was added in one portion and the reaction mixture was stirred overnight. Upon finished, the solution was filtered and concentrated. The relative ester was obtained by recrystallization of the crude material using PE/EA.

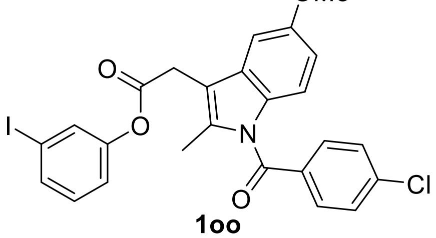

1oo

^{1}H NMR (400 MHz, Chloroform-*d*) δ 7.51 (s, 1H), 7.26-7.20 (m, 1H), 7.14-7.09 (m,

1H), 7.00-6.89 (m, 1H), 6.78 (dd, *J* = 7.8, 2.4 Hz, 1H), 4.23 (t, *J* = 6.8 Hz, 2H), 4.04 (t, *J* = 5.8 Hz, 2H), 3.94 (s, 3H), 3.57 (s, 3H), 2.22-2.09 (m, 2H); ^{13}C NMR(101 MHz, Chloroform-*d*) δ 159.4, 155.3, 151.5, 148.8, 141.5, 130.6, 129.7, 123.7, 113.9, 107.6, 94.2, 66.4, 38.9, 33.5, 29.7, 27.7; HRMS: m/z (ESI) calculated $[M+H]^+$: 560.0121, found: 560.0120.

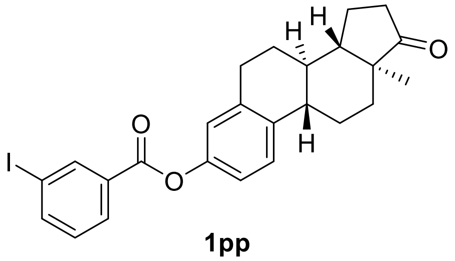

1pp

^{1}H NMR (400 MHz, Chloroform-*d*) δ 8.52 (s, 1H), 8.15 (d, *J* = 7.8 Hz, 1H), 7.95 (d, *J* = 7.8 Hz, 1H), 7.34 (d, *J* = 8.4 Hz, 1H), 7.25 (dd, *J* = 7.8, 7.8 Hz, 1H), 6.97 (dd, *J* = 8.4, 2.2 Hz, 1H), 6.93 (s, 1H), 2.97-2.88 (m, 2H), 2.52 (dd, *J* = 18.8, 8.6 Hz, 1H), 2.47 -2.39 (m, 1H), 2.32 (td, *J* = 10.8, 3.5 Hz, 1H), 2.21-2.08 (m, 2H), 2.07-1.92 (m, 2H), 1.69-1.40 (m, 6H), 0.93 (s, 3H); ^{13}C NMR (101 MHz, Chloroform-*d*) δ 220.6, 163.9, 148.6, 142.3, 138.9, 138.1, 137.6, 131.5, 130.2, 129.3, 126.5, 121.5, 118.7, 93.9 , 50.4, 47.9, 44.2, 38.0, 35.8, 31.5, 29.4, 26.3, 25.7, 21.6, 13.8; HRMS: m/z (ESI) calculated $[M+Na]^+$: 523.0741, found: 523.0723.

General procedure E for applications of the products:

Oxidation of 3y

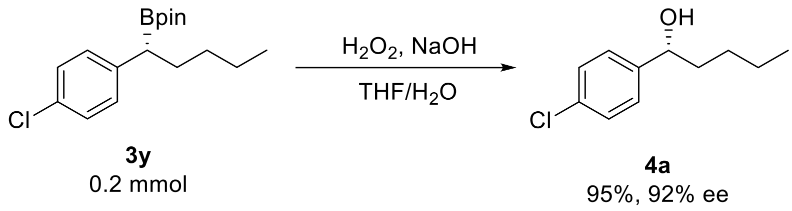

A 25 mL round-bottom flask was charged with **3y** (0.2 mmol, 61.6 mg) and THF (2 mL) placed in an ice bath. At approximately 0 ^{o}C, a 2N NaOH aqueous solution (1.0 mmol, 0.5 mL, 5 equiv.) was added, then H_2O_2 (2.0 equiv., 0.4 mL, 30% aqueous solution) was added. The reaction mixture was stirred for 4~6 hours and the temperature were wormed slowly to room temperature. Upon **3y** was consumed completely, the solution was quenched with 2mL saturated sodium thiosulfate aqueous. The aqueous solution was extracted with EtOAc three times. The combined organic layers were dried over anhydrous Na_2SO_4, filtered and concentrated. The crude material was purified by silica gel column chromatography with a gradient eluent of petroleum ether/ethyl acetate affording **4a** (37.6 mg, 95%, 92% *ee*).

Synthesis of 4b

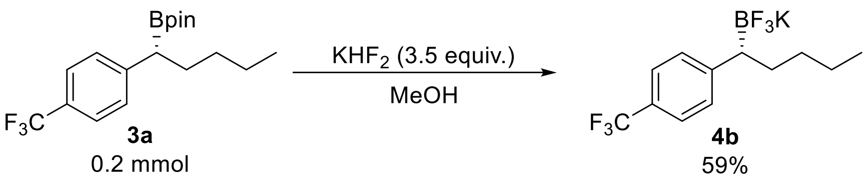

In a 25 mL round-bottom flask, an aqueous solution of KHF_2 (0.7 mmol, 54.6 mg, 3.5 M) was added to a stirring solution of **3a** (68.4 mg) in MeOH (2 mL) at 0 ^{o}C. Once added, the ice bath was removed and the reaction mixture was stirred overnight. Upon finished, the resultant solution was concentrated *in vacuo*, and the residues were washed with hexane/dichloromethane to afford pure **4b** (38.0 mg, 59%).

Arylation of 3y

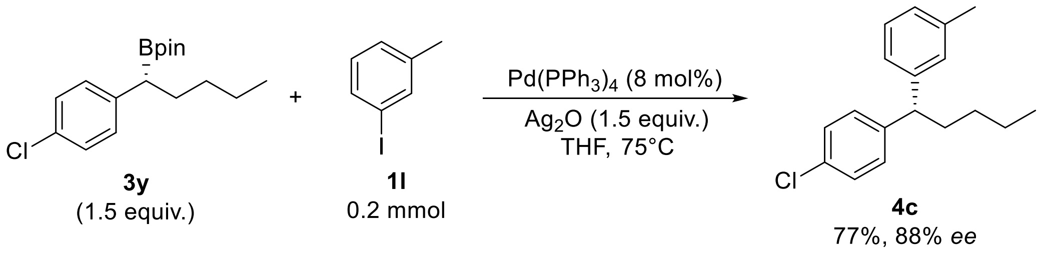

A 10 mL oven-dried Schleck tube was charged with $Pd(PPh_3)_4$ (0.016 mmol,18.5

mg, 8 mol%), Ag_2O (3.0 mmol, 695.2 mg, 1.5 equiv.) in the N_2-filled glovebox. Then the tube was sealed with a septum and taken out of the N_2-filled glovebox. **3y** (0.3 mmol, 92.4 mg, 1.5 equiv.), **1l** (0.2 mmol, 43.6 mg) and THF (4 mL) were added via syringe under N_2 atmosphere and the reaction mixture was stirred at 75 ^oC overnight. The reaction solution was cooled to room temperature and diluted with EtOAc, filtered through Celite, and concentrated *in vacuo*. The residues were purified by silica gel column chromatography with a gradient eluent of petroleum ether/ethyl acetate affording **4c** (41.9 mg, 77%, 88% *ee*). The *ee* value was determined by HPLC analysis.

Vinylation of 3y

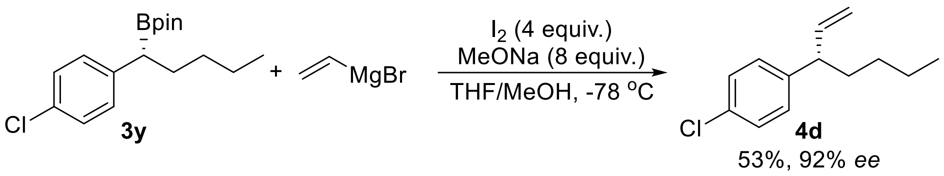

An oven-dried 25 mL screw-cap Schlenk tube equipped with a Teflon stir bar was added with a solution of **3y** (61.6 mg, 0.2 mmol) in anhydrous THF (2 mL) under N_2 atmosphere via syringe. Then a solution of vinylmagnesium bromide (0.8 mmol, 1.6 mL, 0.5 M in THF) was added dropwise, and the mixture was stirred for 30 min at room temperature. Then the tube was cooled to -78 ^oC and a solution of I_2 (0.8 mmol in 2 mL MeOH) was added dropwise. The solution was stirred for another 30 min at this temperature, followed by addition of a solution of NaOMe (1.6 mmol in 2 mL MeOH). The reaction mixture was allowed to worm to room temperature and stirred for another 2 hours. Once finished, the reaction was quenched with 2mL saturated sodium thiosulfate aqueous. The aqueous solution was extracted with EtOAc three times. The combined organic layers were dried over anhydrous Na_2SO_4, filtered and concentrated. The crude material was purified by silica gel column chromatography with a gradient eluent of petroleum ether/ethyl acetate affording **4d** (22.0 mg, 53%, 92% *ee*). The *ee* value was determined by HPLC analysis using the relative alcohol obtained after hydroboration of vinyl group and oxidation of the boryl group[S5].

Synthesis of 4e

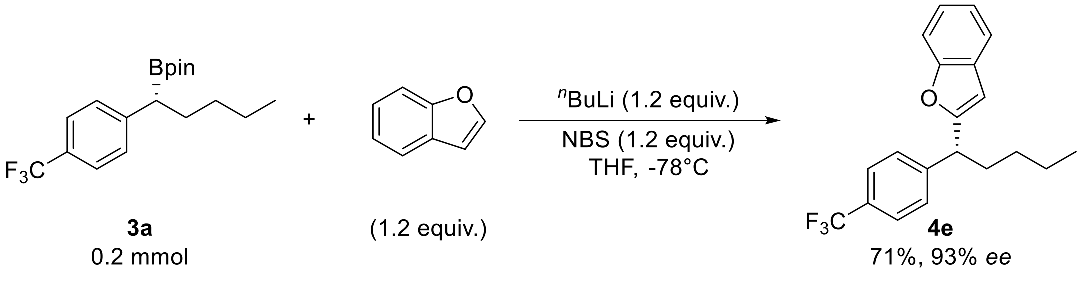

An oven-dried 25 mL screw-cap Schlenk tube equipped with a Teflon stir bar was added with a solution of 1-benzofuran (28.3 mg, 0.24 mmol) and anhydrous THF (2

mL) under N_2 atmosphere, then the tube was cooled to -78 ^oC followed by dropwise addition of ^{n}BuLi (0.15 mL, 0.24 mmol, 1.6 M in Hexane) via micro syringe. The reaction mixture was allowed to worm to room temperature and a solution of **3a** (68.4 mg, 0.2 mmol, 0.2 M in THF) was subsequently added dropwise. After stirring for 1 hour, the resultant mixture was cooled to -78 ^oC and a solution of NBS (45 mg, 0.25 mmol, 0.5 M in THF) was added dropwise. The reaction was stirred for another 1 hour at the same temperature. Upon completed, the reaction was quenched with 2mL saturated sodium thiosulfate aqueous. The aqueous solution was extracted with EtOAc three times. The combined organic layers were dried over anhydrous Na_2SO_4, filtered and concentrated. The crude material was purified by silica gel column chromatography with a gradient eluent of petroleum ether/ethyl acetate affording **4e** (47.1 mg, 71%, 93% *ee*). The *ee* value was determined by HPLC analysis.

2.3 Control and mechanistic experiments

2.3.1 Competing Reactions

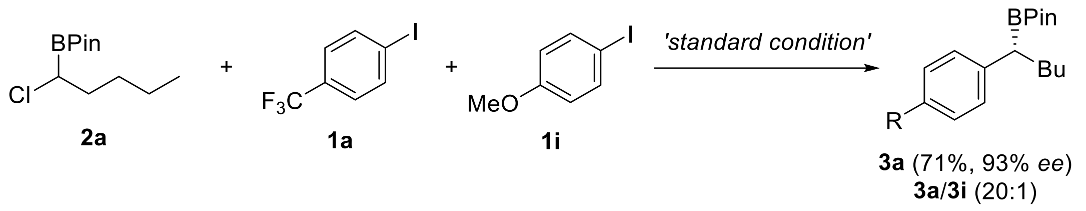

An oven-dried 10-mL Schlenk tube containing a Teflon stir bar was charged with 4CzIPN (0.002 mmol, 1 mol%), $NiBr_2$·DME (0.02 mmol, 10 mol%), **L1** (0.024 mmol, 12 mol%), HEH (0.4 mmol, 2 equiv.), TEA (1.0 mmol, 5 equiv.). Then the tube was sealed with a septum and taken out of the N_2-filled glovebox. DME/DMA (v/v = 5/1, 4 mL) were added via syringe under N_2 atmosphere and the reaction mixture was stirred for 30 min at room temperature. Then **1a** (0.32 mmol, 1.6 equiv.), **1i** (0.32 mmol, 1.6 equiv.) and **2a** (0.2 mmol) were added via micro-syringes. Once added, the tube was closed again, and the reaction mixture was stirred and irradiated under blue light (λ = 450-455 nm) for 8-10 hours, while the temperature was controlled at approximately 20 ^{o}C by cooling with fans and air-conditioner. Upon completed, the mixture was diluted with EtOAc and quenched with water. The aqueous solution was extracted with EtOAc three times. The combined organic layers were dried over anhydrous Na_2SO_4, filtered through Celite, and concentrated *in vacuo*. The ratio of **3a/3i** was determined by HNMR analysis. The residues were purified by silica gel column chromatography with a gradient eluent of petroleum ether/ethyl acetate affording the product **3a** (48.6 mg, 71% yield, 93% *ee*). The *ee* value was determined by HPLC analysis using the relative alcohol after oxidation of the product.

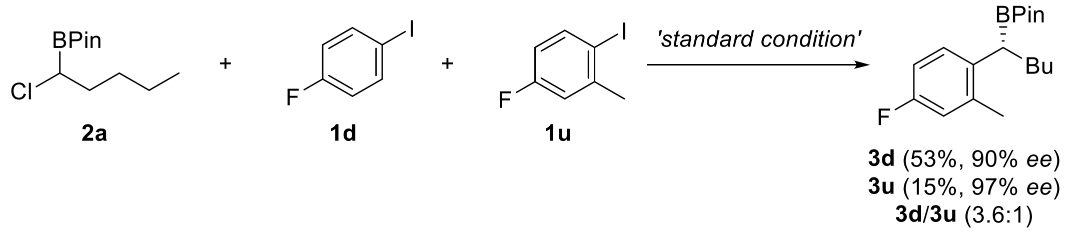

An oven-dried 10-mL Schlenk tube containing a Teflon stir bar was charged with 4CzIPN (0.002 mmol, 1 mol%), $NiBr_2$·DME (0.02 mmol, 10 mol%), **L1** (0.024 mmol, 12 mol%), HEH (0.4 mmol, 2 equiv.), TEA (1.0 mmol, 5 equiv.). Then the tube was sealed with a septum and taken out of the N_2-filled glovebox. DME/DMA (v/v = 5/1, 4 mL) were added via syringe under N_2 atmosphere and the reaction mixture was stirred for 30 min at room temperature. Then **1d** (0.32 mmol, 1.6 equiv.), **1u** (0.32 mmol, 1.6 equiv.) and **2a** (0.2 mmol) were added via micro-syringes. Once added, the tube was closed again, and the reaction mixture was stirred and irradiated under blue light (λ = 450-455 nm) for 8-10 hours, while the temperature was controlled at

approximately 20 °C by cooling with fans and air-conditioner. Upon completed, the mixture was diluted with EtOAc and quenched with water. The aqueous solution was extracted with EtOAc three times. The combined organic layers were dried over anhydrous Na_2SO_4, filtered through Celite, and concentrated *in vacuo*. The ratio of **3d/3u** was determined by FNMR analysis of the crude material. The residues were purified by silica gel column chromatography with a gradient eluent of petroleum ether/ethyl acetate affording a mixture of two products (40.1 mg. **3d**, 53% yield, 90% *ee*; **3u**, 15% yield, 97% *ee*). The *ee* values were determined by HPLC analysis using the relative alcohols after oxidation of the products.

2.3.2 Comparison Reactions

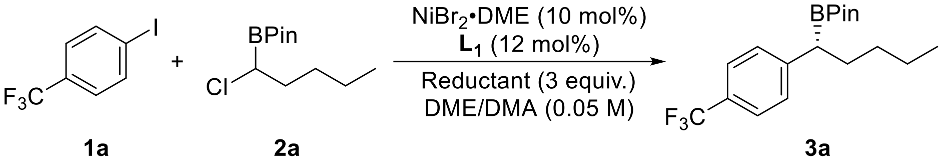

'Standard condition'	Zn	Mn	B_2Pin_2 NaO^{t}Bu	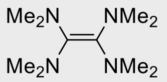
86% 93% *ee*	<5% n.d.	0 ---	< 5% n.d.	< 2% n.d.

An oven-dried 10-mL Schlenk tube containing a Teflon stir bar was charged with $NiBr_2$·DME (0.02 mmol, 10 mol%), **L1** (0.024 mmol, 12 mol%), reductant (0.6 mmol, 3 equiv.). Then the tube was sealed with a septum and taken out of the N_2-filled glovebox. DME/DMA (v/v = 5/1, 4 mL) were added via syringe under N_2 atmosphere and the reaction mixture was stirred for 30 min at room temperature. Then **1a** (0.32 mmol, 1.6 equiv.) and **2a** (0.2 mmol) were added via micro-syringes. Once added, the tube was closed again, and the reaction mixture was stirred for 12 hours at room temperature. Upon completed, the mixture was diluted with EtOAc and n-Dodecane as a GC internal standard was added. The yield was determined by GC analysis.

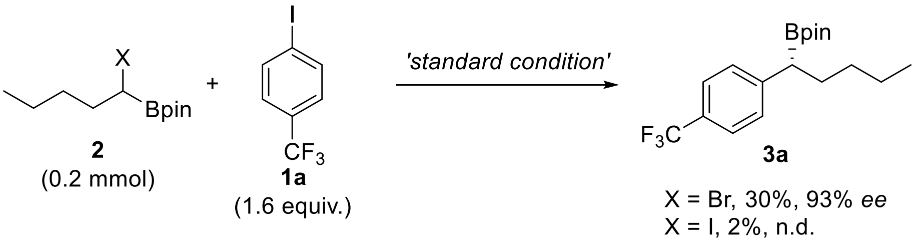

An oven-dried 10-mL Schlenk tube containing a Teflon stir bar was charged with 4CzIPN (0.002 mmol, 1 mol%), $NiBr_2$·DME (0.02 mmol, 10 mol%), **L1** (0.024 mmol, 12 mol%), HEH (0.4 mmol, 2 equiv.), TEA (1.0 mmol, 5 equiv.). Then the tube was sealed with a septum and taken out of the N_2-filled glovebox. DME/DMA (v/v = 5/1, 4 mL) were added via syringe under N_2 atmosphere and the reaction mixture was stirred for 30 min at room temperature. Then **1a** (0.32 mmol, 1.6 equiv.) and **2** (0.2

mmol) were added via micro-syringes. Once added, the tube was closed again, and the reaction mixture was stirred and irradiated under blue light (λ = 450-455 nm) for 8-10 hours, while the temperature was controlled at approximately 20 °C by cooling with fans and air-conditioner. Upon completed, the mixture was diluted with EtOAc and quenched with water. The aqueous solution was extracted with EtOAc three times. The combined organic layers were dried over anhydrous Na_2SO_4, filtered through Celite, and concentrated *in vacuo*. The residues were purified by silica gel column chromatography with a gradient eluent of petroleum ether/ethyl acetate affording the product **3a**. The *ee* value was determined by HPLC analysis using the relative alcohol after oxidation of the product.

2.3.3 Mechanistic Studies Experiments

Cyclization Reactions

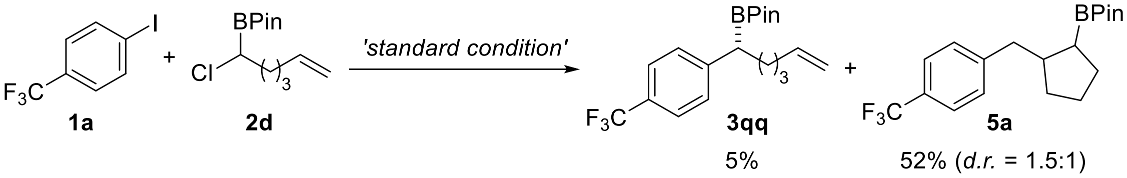

An oven-dried 10-mL Schlenk tube containing a Teflon stir bar was charged with 4CzIPN (0.002 mmol, 1 mol%), $NiBr_2$·DME (0.02 mmol, 10 mol%), **L1** (0.024 mmol, 12 mol%), HEH (0.4 mmol, 2 equiv.), TEA (1.0 mmol, 5 equiv.). Then the tube was sealed with a septum and taken out of the N_2-filled glovebox. DME/DMA (v/v = 5/1, 4 mL) were added via syringe under N_2 atmosphere and the reaction mixture was stirred for 30 min at room temperature. Then **2d** (0.32 mmol, 1.6 equiv.) and **1a** (0.2 mmol) were added via micro-syringes. Once added, the tube was closed again, and the reaction mixture was stirred and irradiated under blue light (λ = 450-455 nm) for 8-10 hours, while the temperature was controlled at approximately 20 °C by cooling with fans and air-conditioner. Upon completed, the mixture was diluted with EtOAc and quenched with water. The aqueous solution was extracted with EtOAc three times. The combined organic layers were dried over anhydrous Na_2SO_4, filtered through Celite, and concentrated *in vacuo*. The yield of **3qq** was determined by HNMR analysis using CH_2Br_2 as internal standard. The residues were purified by silica gel column chromatography with a gradient eluent of petroleum ether/ethyl acetate affording the product **5a** (52%, 36.8 mg, dr = 1.5:1), the dr value was determined by HNMR analysis of the purified product. ^{1}H NMR (400 MHz, Chloroform-*d*) δ 7.57 – 7.45 (m, 5H, **5a-1**+**5a-2**), 7.34-7.27 (m, 5H, **5a-1**+**5a-2**), 2.90 (dd, *J* = 13.2, 5.0 Hz, 1.5H, **5a-2**), 2.77 (dd, *J* = 13.2, 6.6 Hz, 1H, **5a-1**), 2.63 (dd, *J* = 13.2, 7.8 Hz, 1H, **5a-1**), 2.53-2.44 (m, 1.5H, **5a-2**), 2.42-2.30 (m, 1.5H, **5a-2**), 2.23-2.12 (m, 1H, **5a-1**), 1.90-1.67 (m, 5H, **5a-1**+**5a-2**), 1.59-1.45 (m, 5H, **5a-1**+**5a-2**), 1.36-1.28 (m, 5H, **5a-1**+**5a-2**), 1.27 (s, 9H, **5a-2**), 1.26 (s, 9H, **5a-2**), 1.14 (s, 12H, **5a-1**), 1.04-0.78 (m, 2.5H, **5a-1**+**5a-2**); ^{13}C NMR (101 MHz, Chloroform-*d*) δ 146.8, 146.4, 129.3, 129.1,

128.0, 127.9 (q, J = 32.3 Hz, 127.9 (d, J = 32.2 Hz), 125.1 (q, J = 4.7 Hz), 125.0 (q, J = 3.7 Hz), 124.4 (d, J = 271.6 Hz), 83.0, 82.8, 44.7, 43.9, 42.4, 39.3, 33.7, 31.7, 28.6, 27.2, 25.9, 25.0, 24.9, 24.6, 24.6, 24.6. HRMS: m/z (ESI) calculated $[M+H]^+$: 355.2054, found: 355.2056.

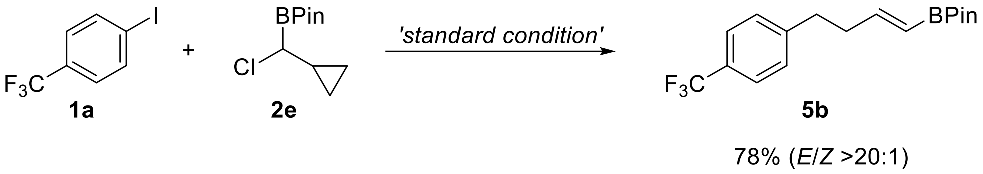

An oven-dried 10-mL Schlenk tube containing a Teflon stir bar was charged with 4CzIPN (0.002 mmol, 1 mol%), $NiBr_2$·DME (0.02 mmol, 10 mol%), **L1** (0.024 mmol, 12 mol%), HEH (0.4 mmol, 2 equiv.), TEA (1.0 mmol, 5 equiv.). Then the tube was sealed with a septum and taken out of the N_2-filled glovebox. DME/DMA (v/v = 5/1, 4 mL) were added via syringe under N_2 atmosphere and the reaction mixture was stirred for 30 min at room temperature. Then **1a** (0.32 mmol, 1.6 equiv.) and **2e** (0.2 mmol) were added via micro-syringes. Once added, the tube was closed again, and the reaction mixture was stirred and irradiated under blue light (λ = 450-455 nm) for 8-10 hours, while the temperature was controlled at approximately 20 °C by cooling with fans and air-conditioner. Upon completed, the mixture was diluted with EtOAc and quenched with water. The aqueous solution was extracted with EtOAc three times. The combined organic layers were dried over anhydrous Na_2SO_4, filtered through Celite, and concentrated *in vacuo*. The residues were purified by silica gel column chromatography with a gradient eluent of petroleum ether/ethyl acetate affording the product **5b** (78%, 50.9 mg, E/Z > 20:1), and the E/Z value was obtained by HNMR analysis of the purified product. ^{1}H NMR (400 MHz, Chloroform-*d*) δ 7.52 (d, J = 8.0 Hz, 2H), 7.29 (d, J = 8.0 Hz, 2H), 6.66 (dt, J = 18.0, 6.2 Hz, 1H), 5.49 (d, J = 18.0 Hz, 1H), 2.80 (t, J = 8.2 Hz, 2H), 2.48 (dt, J = 8.2, 6.2 Hz, 2H), 1.27 (s, 12H); ^{13}C NMR (101 MHz, Chloroform-*d*) δ 152.5 , 145.8, 128.6, 128.2 (q, J = 32.2 Hz), 125.2 (q, J = 3.8 Hz), 124.3 (q, J = 271.7 Hz), 83.1, 36.9, 34.3, 24.7. HRMS: m/z (ESI) calculated $[M+Na]^+$: 349.1557, found: 349.1569.

Reactions with radical probes

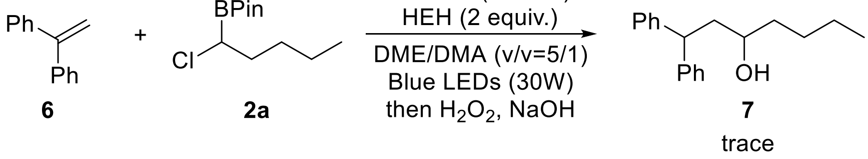

An oven-dried 10-mL Schlenk tube containing a Teflon stir bar was charged with 4CzIPN (0.002 mmol, 1 mol%) and HEH (0.4 mmol, 2 equiv.). Then the tube was sealed with a septum and taken out of the N_2-filled glovebox. DME/DMA (v/v = 5/1, 4 mL) were added via syringe under N_2 atmosphere, then **6** (0.6 mmol, 3.0 equiv.) and **2a** (0.2 mmol) were added via micro-syringes. Once added, the tube was closed again,

and the reaction mixture was stirred and irradiated under blue light (λ = 450-455 nm) for 6-8 hours, while the temperature was controlled at approximately 20 °C by cooling with fans and air-conditioner. Upon completed, he mixture was diluted with EtOAc and quenched with water. The aqueous solution was extracted with EtOAc three times. The combined organic layers were dried over anhydrous Na_2SO_4, filtered through Celite, and concentrated *in vacuo*. The residues were dissolved in 1 mL THF. At approximately 0 °C, 2*N* NaOH aqueous solution (1.0 mmol, 0.5 mL, 5 equiv.) was added to the stirring solution, then H_2O_2 (2.0 equiv., 0.4 mL, 30% aqueous solution) was added. The reaction mixture was stirred for 4~6 hours and the temperature were wormed slowly to room temperature. Once finished, the solution was quenched with 2 mL saturated sodium thiosulfate aqueous. The aqueous solution was extracted with EtOAc three times. The combined organic layers were dried over anhydrous Na_2SO_4, filtered and concentrated. Then the residues were diluted with EtOAc and n-Dodecane as a GC internal standard was added. The yield was determined by GC analysis and trace amount of **7** was detected.

Ni(COD)$_2$ Stoichiometry Studies

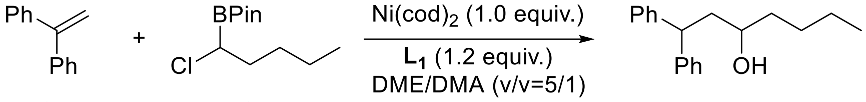

6 **2a** **7**

23%, racemic

An oven-dried 10-mL Schlenk tube containing a Teflon stir bar was charged with Ni(cod)$_2$ (0.05 mmol, 1.0 equiv.) and **L1** (0.06 mmol, 1.2 equiv.). Then the tube was sealed with a septum and taken out of the N_2-filled glovebox. DME/DMA (v/v = 5/1, 1 mL) were added via syringe under N_2 atmosphere, then **6** (0.15 mmol, 3.0 equiv.) and **2a** (0.05 mmol) were added via micro-syringes. Once added, the tube was closed again, and the reaction mixture was stirred at room temperature for 6-8 hours. Upon completed, the mixture was diluted with EtOAc and quenched with water. The aqueous solution was extracted with EtOAc three times. The combined organic layers were dried over anhydrous Na_2SO_4, filtered through Celite, and concentrated *in vacuo*. The residues were dissolved in 1 mL THF. At approximately 0 °C, 2*N* NaOH aqueous solution (1.0 mmol, 0.5 mL, 5 equiv.) was added to the stirring solution, then H_2O_2 (2.0 equiv., 0.4 mL, 30% aqueous solution) was added. The reaction mixture was stirred for 4~6 hours and the temperature were wormed slowly to room temperature. Once finished, the solution was quenched with 2mL saturated sodium thiosulfate aqueous. The aqueous solution was extracted with EtOAc three times. The combined organic layers were dried over anhydrous Na_2SO_4, filtered and concentrated. The crude material was purified by silica gel column chromatography with a gradient eluent of petroleum ether/ethyl acetate affording the racemic product **7** (23% yield, 3.1 mg), and *ee* value was determined by HPLC analysis using the derivative alcohol. ^{1}H NMR (400 MHz, Chloroform-*d*) δ 7.32-7.22 (m, 8H), 7.21-7.13 (m, 2H), 4.24 (dd, *J* = 10.2, 5.6 Hz, 1H), 3.55-3.39 (m, 1H), 2.30-2.19 (m, 1H), 2.14-2.04 (m, 1H), 1.52 -1.43 (m, 2H), 1.40-1.22 (m, 6H), 0.87 (t, *J* = 6.8 Hz, 3H); ^{13}C NMR (101 MHz,

Chloroform-*d*) δ 145.3, 144.2, 128.5, 128.5, 128.1, 127.7, 126.3, 126.1, 69.8, 47.6, 43.2, 37.7, 27.7, 22.7, 14.0; HRMS: m/z (ESI) calculated $[M+Na]^+$: 291.1719, found: 291.1721.

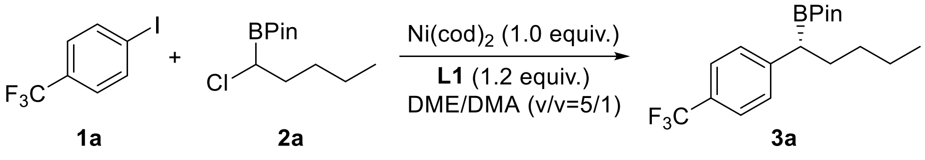

An oven-dried 10-mL Schlenk tube containing a Teflon stir bar was charged with $Ni(cod)_2$ (0.05 mmol, 1.0 equiv.) and **L1** (0.06 mmol, 1.2 equiv.). Then the tube was sealed with a septum and taken out of the N_2-filled glovebox. DME/DMA (v/v = 5/1, 1 mL) were added via syringe under N_2 atmosphere, then **1a** (0.15 mmol, 3.0 equiv.) and **2a** (0.05 mmol) were added via micro-syringes. Once added, the tube was closed again, and the reaction mixture was stirred at room temperature overnight. Upon completed, the mixture was diluted with EtOAc and quenched with water. The aqueous solution was extracted with EtOAc three times. The combined organic layers were dried over anhydrous Na_2SO_4, filtered through Celite, and concentrated *in vacuo*. The residues were purified by silica gel column chromatography with a gradient eluent of petroleum ether/ethyl acetate affording the racemic product **3a** (17% yield, 82% *ee*, 2.9 mg), and *ee* value was determined by HPLC analysis using the derivative alcohol.

2.3.4 Stern-Volmer Quenching Studies

The stern-volmer Fluorescence experiments were conducted using Hitachi Instrument F-7000 spectrofluorometer with a screw-top quartz cuvette (45 mm x 10 mm x 10 mm).

DME/DMA (v/v = 5:1) solution was sparged with N_2 for at least 1 hour and then transferred into N_2-filled glovebox. All the solutions were prepared in N_2-filled glovebox and the general procedures were as follows:

Photocatalyst solution

To an oven dried 100 mL flask 4CzIPN (4 mg, 0.005 mmol) was dissolved in 60 mL DME/DMA (v/v = 5:1) to freshly prepare an 8.5 x 10^{-5} M 4CzIPN solution.

α-Chloro pentanal pinacol boronate ester (1a) solution

To an oven dried 5 mL vail **1a** (232.0 mg, 1.0 mmol) was dissolved in 1 mL DME/DMA (v/v = 5:1) to freshly prepare a 1.0 M solution of **1a**.

TEA solution

To an oven dried 5 mL vail TEA (50.5 mg, 0.5 mmol) was dissolved in 2 mL DME/DMA (v/v = 5:1) to freshly prepare a 0.25 M TEA solution.

HEH solution

To an oven dried 5 mL vail TEA (25.2 mg, 0.1 mmol) was dissolved in 2 mL DME/DMA (v/v = 5:1) to freshly prepare a 0.05 M HEH solution.

The solutions were irradiated at 418 nm, and the emission were measured from 450 nm to 700 nm. The emission intensities were recorded at the maximum wavelength (λ_{em} = 528 nm). The Stern-Volmer Quenching plots of 4CzIPN solution were obtained by **1a**, TEA and HEH quencher respectively.

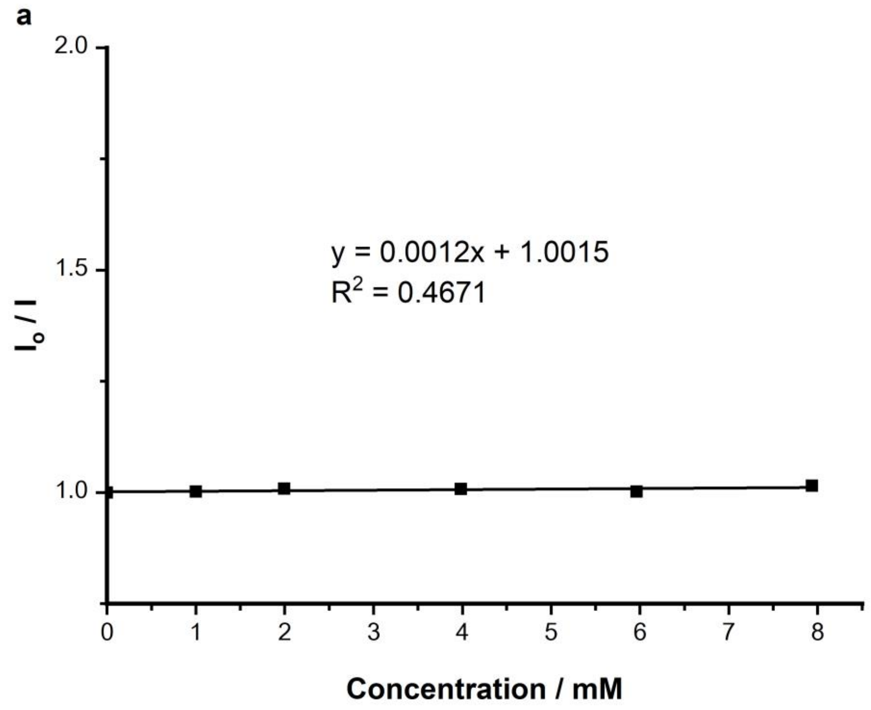

Supplementary Figure S2. Stern-Volmer Quenching Plot of **1a**

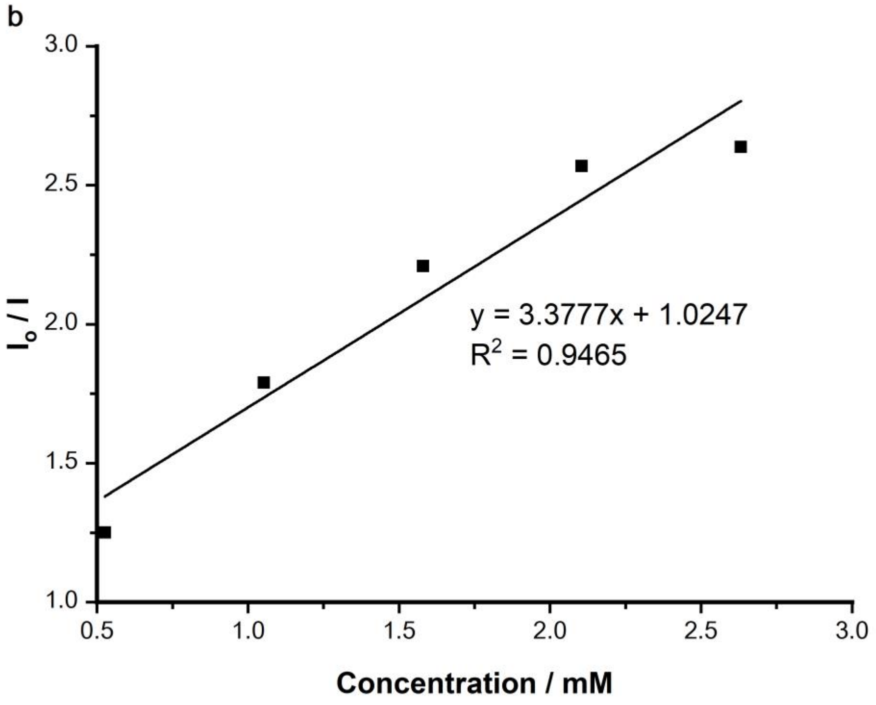

Supplementary Figure S3. Stern-Volmer Quenching Plot of HEH

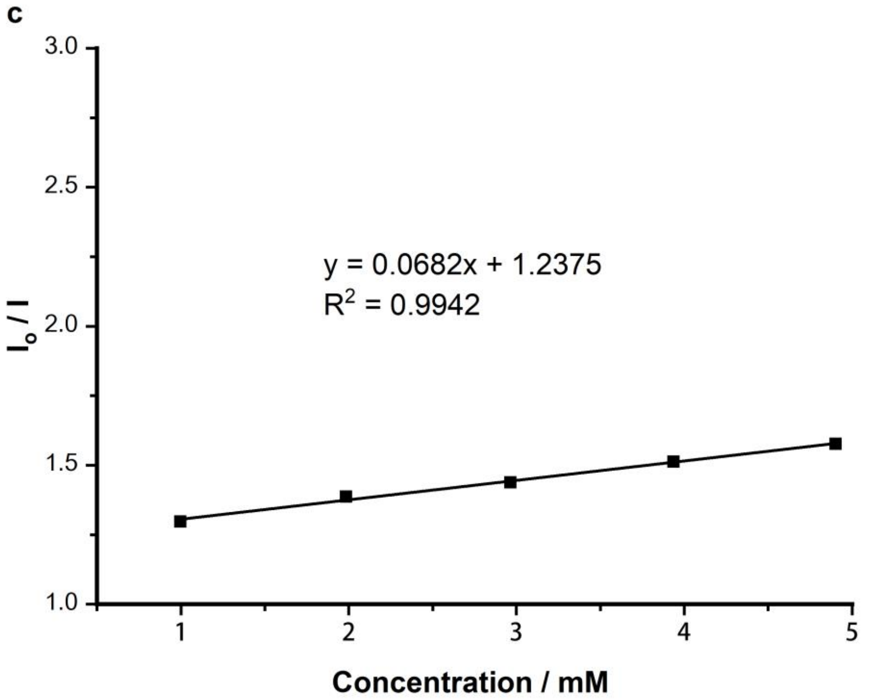

Supplementary Figure S4. Stern-Volmer Quenching Plot of TEA

2.4 The characterization of the compounds

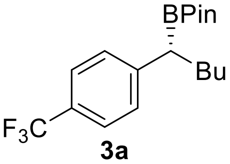

3a

The title compound **3a** was synthesized according to General Procedure B, and it was purified by column chromatography on silica gel (86% yield, 93% *ee*, 58.8 mg, colorless oil).

^{1}H NMR (400 MHz, Chloroform-d) δ 7.50 (d, *J* = 8.0 Hz, 2H), 7.31 (d, *J* = 8.0 Hz, 2H), 2.36 (dd, *J* = 7.9, 8.0 Hz, 1H), 1.92-1.80 (m, 1H), 1.71-1.60 (m, 1H), 1.35-1.20 (m, 4H), 1.21 (s, 6H), 1.19 (s, 6H), 0.86 (t, *J* = 7.1 Hz, 3H); ^{13}C NMR (101 MHz, Chloroform-*d*) δ 147.9, 128.5, 127.3 (q, *J* = 32.0 Hz), 124.5 (q, *J* = 271.7 Hz), 125.1 (q, *J* = 3.8 Hz), 83.5, 32.0, 31.4, 24.6, 24.6, 22.6, 14.0; HRMS: m/z (ESI) calculated $[M+Na]^+$:365.1870, found: 365.1881. $[\alpha]_D^{25}$ = -7.99 (*c* = 0.25, $CHCl_3$).
The enantiomeric excess of **3a** was determined by chiral HPLC analysis compared to the corresponding racemate alcohol.
Conditions: ChiralPak IC column; hexane/^{i}PrOH = 98:2; flow rate = 1.0 mL/min; λ= 220 nm; t_{R1}(major)= 5.7 min; t_{R2}(minor)= 6.4 min.

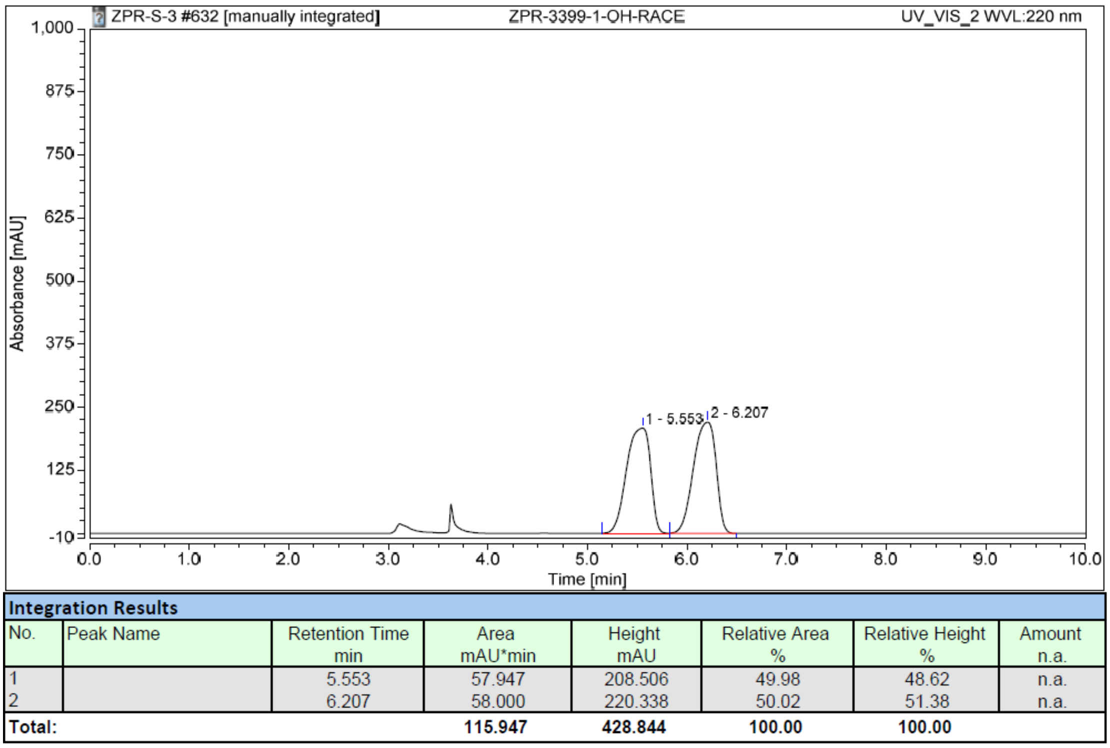

Integration Results							
No.	Peak Name	Retention Time min	Area mAU*min	Height mAU	Relative Area %	Relative Height %	Amount n.a.
1		5.553	57.947	208.506	49.98	48.62	n.a.
2		6.207	58.000	220.338	50.02	51.38	n.a.
**Total:**			**115.947**	**428.844**	**100.00**	**100.00**	

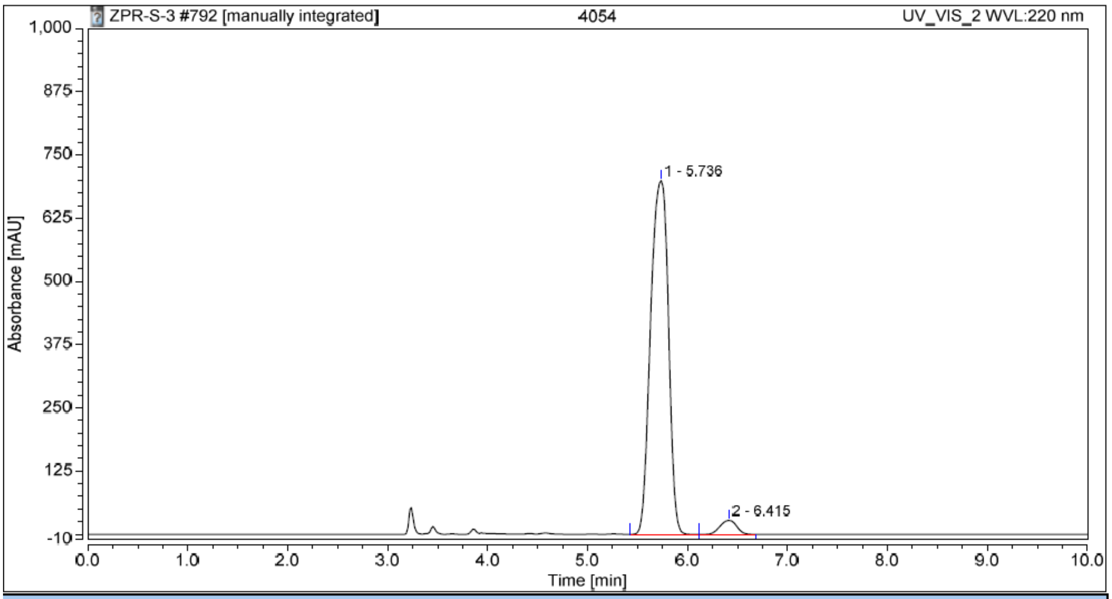

Integration Results

No.	Peak Name	Retention Time min	Area mAU*min	Height mAU	Relative Area %	Relative Height %	Amount n.a.
1		5.736	143.707	699.029	96.45	96.22	n.a.
2		6.415	5.288	27.479	3.55	3.78	n.a.
**Total:**			**148.995**	**726.508**	**100.00**	**100.00**	

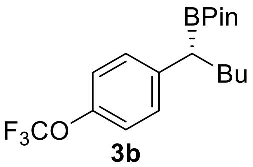

3b

The title compound **3b** was synthesized according to General Procedure B, and it was purified by column chromatography on silica gel (65% yield, 90% *ee*, 46.5 mg, colorless oil).

^{1}H NMR (400 MHz, Chloroform-*d*) δ 7.21 (d, *J* = 8.2 Hz, 2H), 7.09 (d, *J* = 8.2 Hz, 2H), 2.30 (dd, *J* = 7.9, 8.0 Hz, 1H), 1.901.78 (m, 1H), 1.661.56 (m, 1H), 1.401.22 (m, 4H), 1.21 (s, 6H), 1.19 (s, 6H), 0.86 (t, *J* = 7.1 Hz, 3H); ^{13}C NMR (101 MHz, Chloroform-*d*) δ 146.9, 142.3, 129.4, 120.0 (q, *J* = 257.2 Hz), 120.7, 83.4, 32.3, 31.4, 24.6, 24.5, 22.6. HRMS: m/z (ESI) calculated [M+H]$^+$: 359.2000, found: 359.1980. $[\alpha]_D^{25}$ = -15.853 (*c* = 0.25, $CHCl_3$).

The enantiomeric excess of **3b** was determined by chiral HPLC analysis compared to the corresponding racemate alcohol.

Conditions: ChiralPak IC column; hexane/^{i}PrOH = 98:2; flow rate = 1.0 mL/min; λ= 220 nm; t_{R1}(major)= 5.6 min; t_{R2}(minor)= 6.0 min.

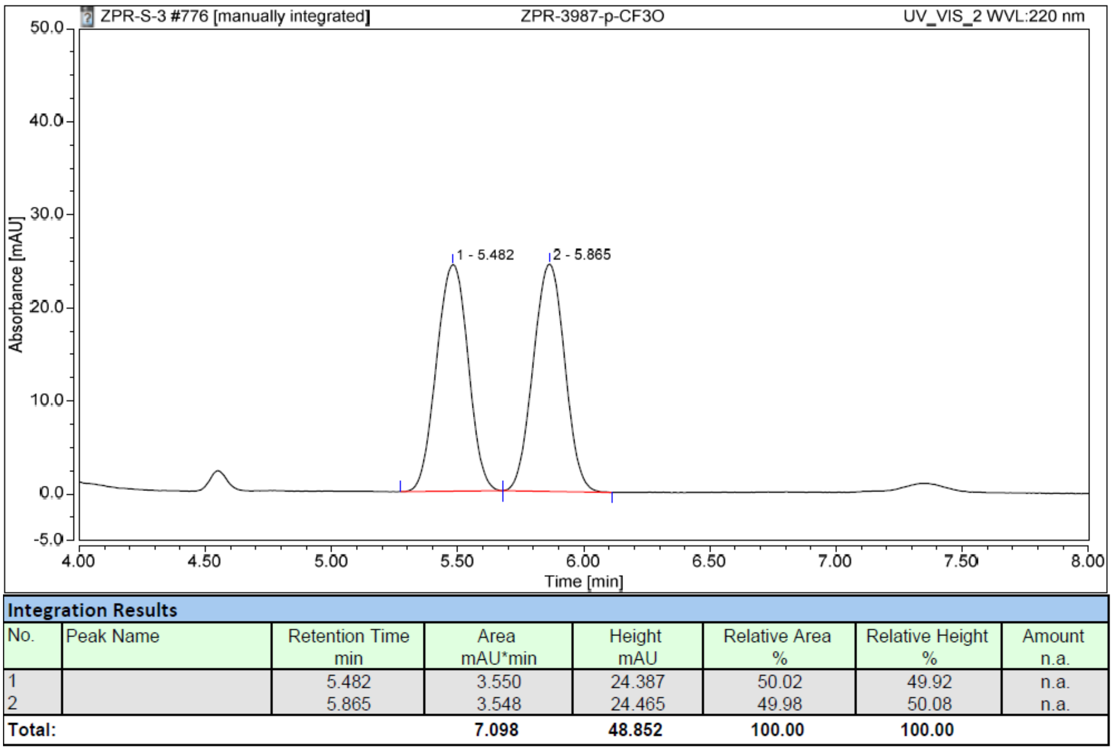

Integration Results							
No.	Peak Name	Retention Time min	Area mAU*min	Height mAU	Relative Area %	Relative Height %	Amount n.a.
1		5.482	3.550	24.387	50.02	49.92	n.a.
2		5.865	3.548	24.465	49.98	50.08	n.a.
**Total:**			**7.098**	**48.852**	**100.00**	**100.00**	

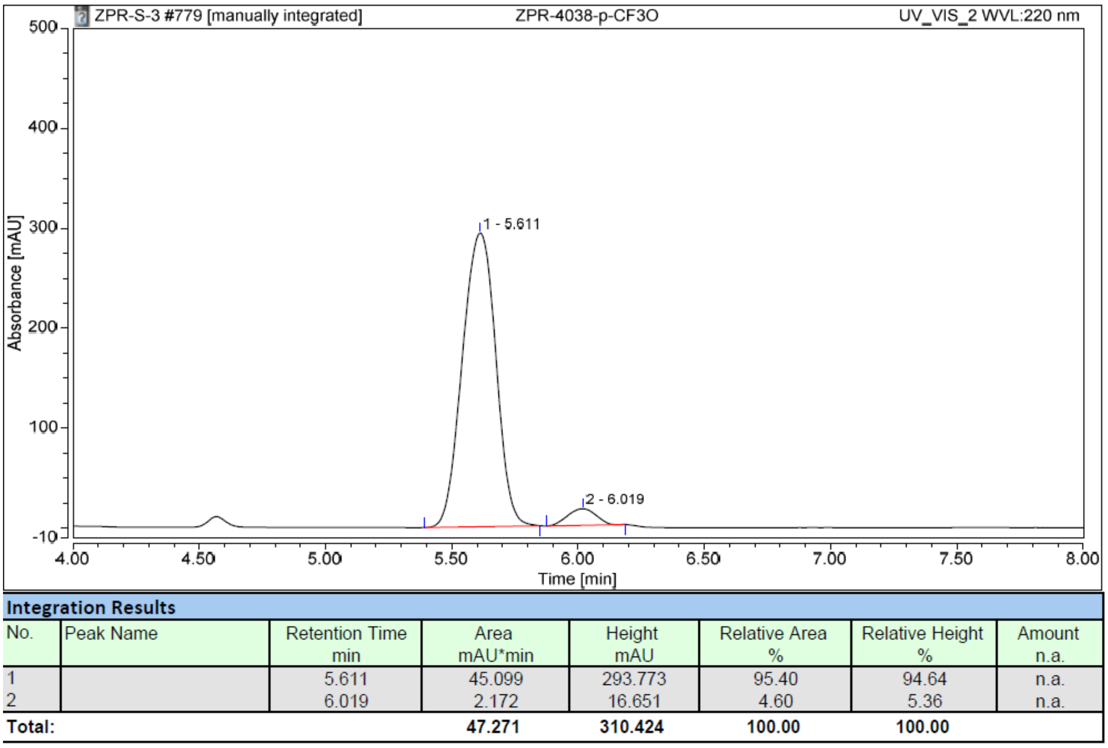

Integration Results							
No.	Peak Name	Retention Time min	Area mAU*min	Height mAU	Relative Area %	Relative Height %	Amount n.a.
1		5.611	45.099	293.773	95.40	94.64	n.a.
2		6.019	2.172	16.651	4.60	5.36	n.a.
**Total:**			**47.271**	**310.424**	**100.00**	**100.00**	

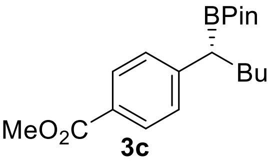

3c

The title compound **3c** was synthesized according to General Procedure B, and it was purified by column chromatography on silica gel (56% yield, 96% *ee*, 37.2 mg, colorless oil).

^{1}H NMR (400 MHz, Chloroform-*d*) δ 7.93 (d, *J* = 8.4 Hz, 2H), 7.27 (d, *J* = 8.4 Hz, 2H), 3.89 (s, 3H), 2.37 (dd, *J* = 7.9, 8.0 Hz, 1H), 1.94-1.79 (m, 1H), 1.73-1.61 (m, 1H), 1.38-1.22 (m, 4H), 1.19 (s, 6H), 1.18 (s, 6H), 0.85 (t, *J* = 7.1 Hz, 3H); ^{13}C NMR (101 MHz, Chloroform-*d*) δ 167.3, 149.4, 129.6, 128.3, 127.0, 83.4, 51.9, 31.7, 31.5, 24.6, 24.5, 22.6, 14.0; HRMS: m/z (ESI) calculated $[M+Na]^{+}$: 405.2183, found: 405.2201. $[\alpha]_D^{25}$ = -16.371 (*c* = 0.25, $CHCl_3$).
The enantiomeric excess of **3c** was determined by chiral HPLC analysis compared to the corresponding racemate alcohol.
Conditions: ChiralPak IC column; hexane/^{i}PrOH = 90:10; flow rate = 1.0 mL/min; λ= 254 nm; t_{R1}(major)= 10.4 min; t_{R2}(minor)= 10.9 min.

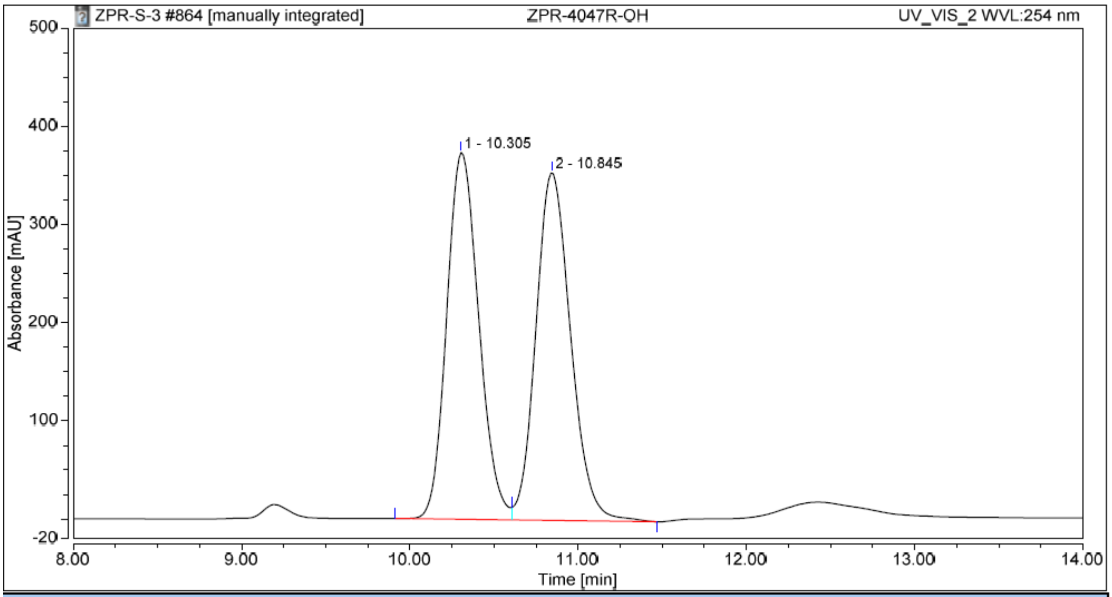

Integration Results

No.	Peak Name	Retention Time min	Area mAU*min	Height mAU	Relative Area %	Relative Height %	Amount n.a.
1		10.305	82.547	373.613	49.35	51.32	n.a.
2		10.845	84.728	354.338	50.65	48.68	n.a.
**Total:**			**167.274**	**727.951**	**100.00**	**100.00**	

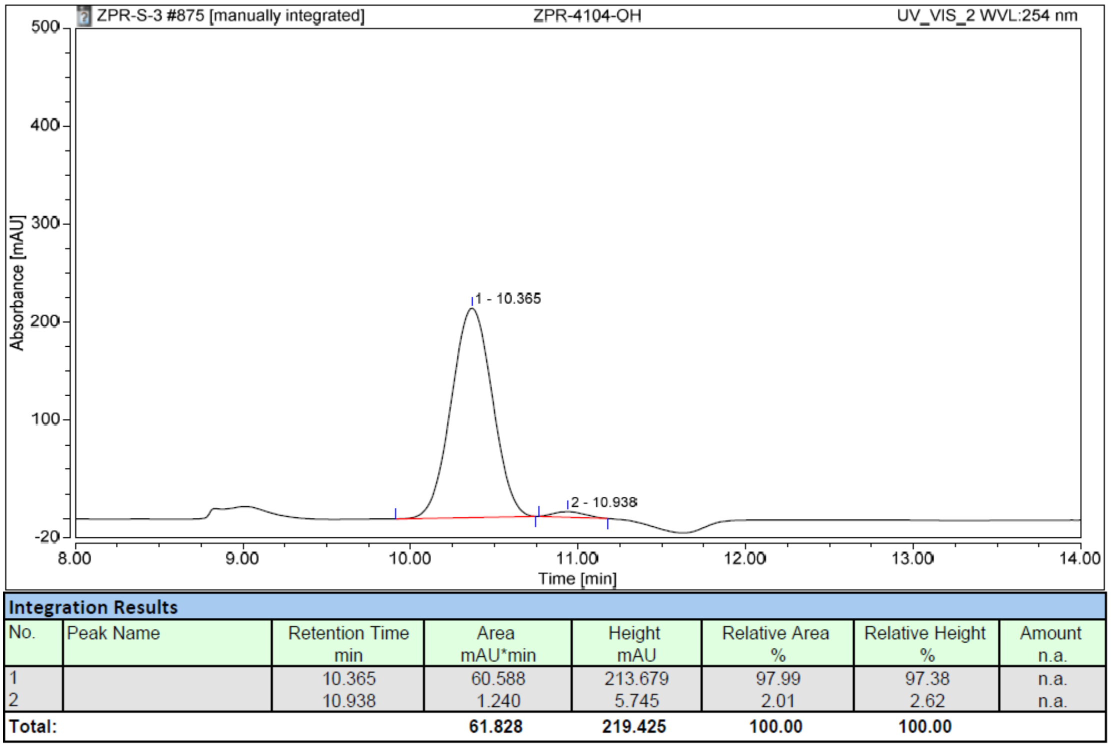

Integration Results

No.	Peak Name	Retention Time min	Area mAU*min	Height mAU	Relative Area %	Relative Height %	Amount n.a.
1		10.365	60.588	213.679	97.99	97.38	n.a.
2		10.938	1.240	5.745	2.01	2.62	n.a.
**Total:**			**61.828**	**219.425**	**100.00**	**100.00**	

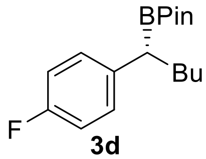

3d

The title compound **3d** was synthesized according to General Procedure B, and it was purified by column chromatography on silica gel (62% yield, 90% *ee*, 36.2 mg, colorless oil)

^{1}H NMR (400 MHz, Chloroform-*d*) δ 7.23-7.08 (m, 2H), 7.08-6.80 (m, 2H), 2.27 (dd, *J* = 7.8, 7.8 Hz, 1H), 1.88-1.74 (m, 1H), 1.65-1.55 (m, 1H), 1.34-1.25 (m, 4H), 1.20 (s, 6H), 1.19 (s, 6H), 0.85 (t, *J* = 7.0 Hz, 3H); ^{13}C NMR (101 MHz, Chloroform-*d*) δ 160.8 (d, *J* = 242.3 Hz), 139.0 (d, *J* = 3.0 Hz), 129.5 (d, *J* = 7.6 Hz), 114.9 (d, *J* = 20.8 Hz), 83.3, 32.4, 31.4, 24.6, 24.5, 22.6, 14.0; HRMS: m/z (ESI) calculated $[M+NH_4]^+$:315.1902, found: 315.1904. $[\alpha]_D^{25}$ = -7.463 (*c* = 0.25, $CHCl_3$).
The enantiomeric excess of **3d** was determined by chiral HPLC analysis compared to the corresponding racemate alcohol.
Conditions: ChiralPak IG column; hexane/^{i}PrOH = 98:2; flow rate = 1.0 mL/min; λ= 220 nm; t_{R1}(major)= 13.6 min; t_{R2}(minor)= 12.1 min.

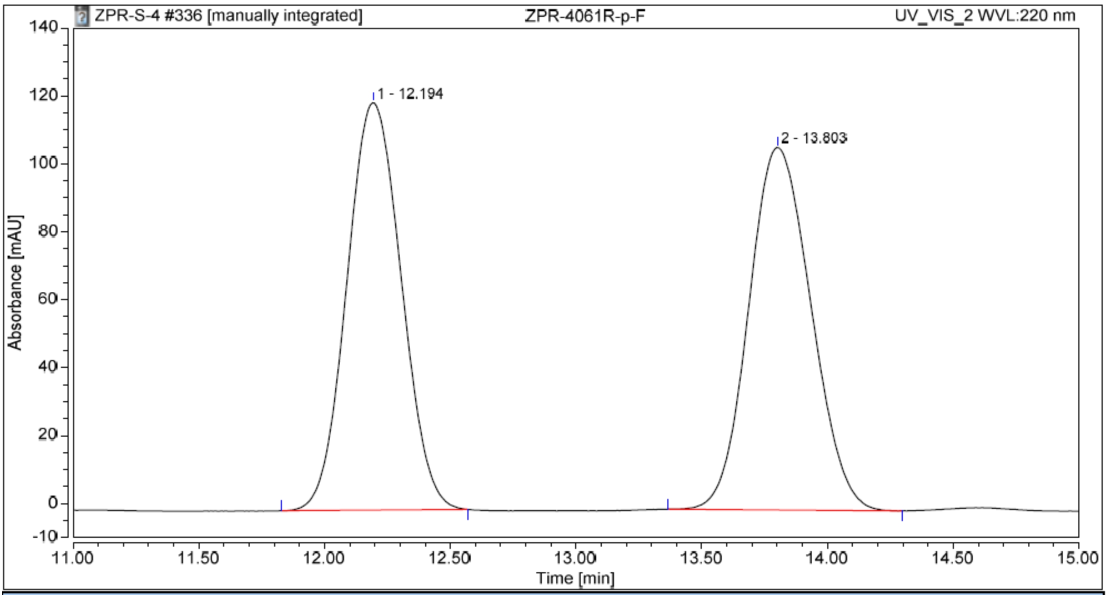

Integration Results							
No.	Peak Name	Retention Time min	Area mAU*min	Height mAU	Relative Area %	Relative Height %	Amount n.a.
1		12.194	30.093	119.989	49.56	52.92	n.a.
2		13.803	30.631	106.735	50.44	47.08	n.a.
**Total:**			**60.724**	**226.724**	**100.00**	**100.00**	

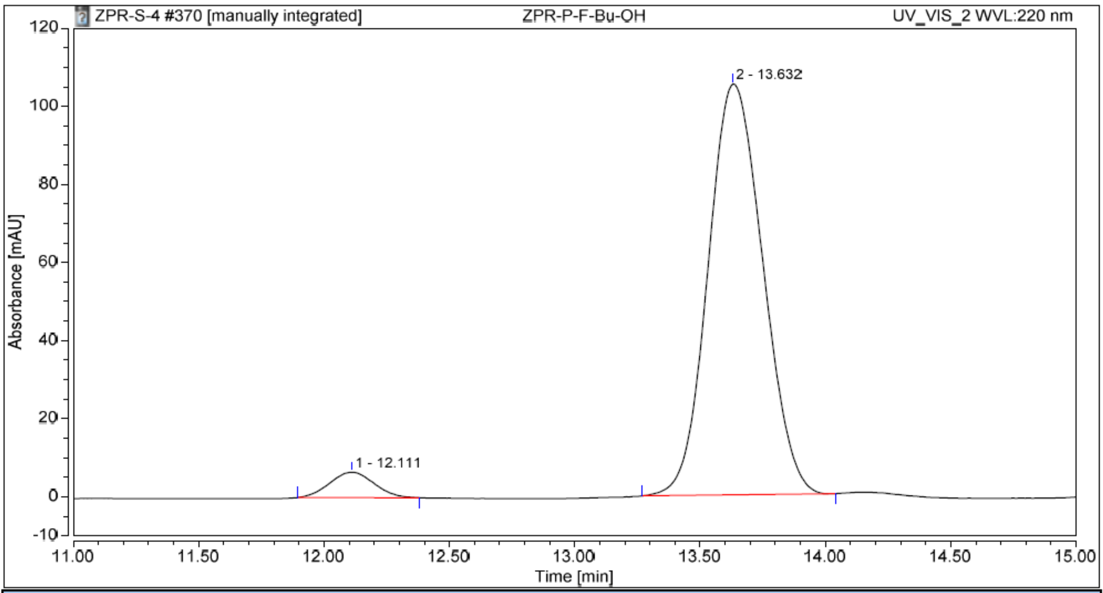

Integration Results							
No.	Peak Name	Retention Time min	Area mAU*min	Height mAU	Relative Area %	Relative Height %	Amount n.a.
1		12.111	1.327	6.491	4.75	5.81	n.a.
2		13.632	26.609	105.298	95.25	94.19	n.a.
**Total:**			**27.936**	**111.789**	**100.00**	**100.00**	

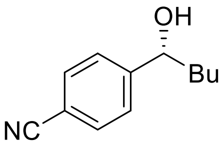

3e

The title compound **3e** was synthesized according to General Procedure B, and it was purified by column chromatography on silica gel (47% yield, 94% *ee*, 17.8 mg, colorless oil)

^{1}H NMR (400 MHz, Chloroform-*d*) δ 7.64 (d, *J* = 8.0 Hz, 2H), 7.46 (d, *J* = 8.0 Hz, 2H), 4.78-4.73 (m, 1H), 1.97 (br, 1H), 1.81-1.67 (m, 2H), 1.42-1.28 (m, 4H), 0.89 (t, *J* = 6.0 Hz, 3H); ^{13}C NMR (101 MHz, Chloroform-*d*) δ 150.2, 132.2, 126.5, 118.8, 111.1, 73.8, 38.9, 27.6, 22.5, 13.9; HRMS: m/z (ESI) calculated $[M+H]^+$: 190.1226, found: 190.1230. $[\alpha]_D^{25}$ = +13.183 (*c* = 0.25, $CHCl_3$).

The enantiomeric excess of **3e** was determined by chiral HPLC analysis compared to the corresponding racemate alcohol.

Conditions: ChiralPak IA column; hexane/^{i}PrOH = 95:5; flow rate = 0.8 mL/min; λ= 220 nm; t_{R1}(major)= 18.8 min; t_{R2}(minor)= 19.7 min.

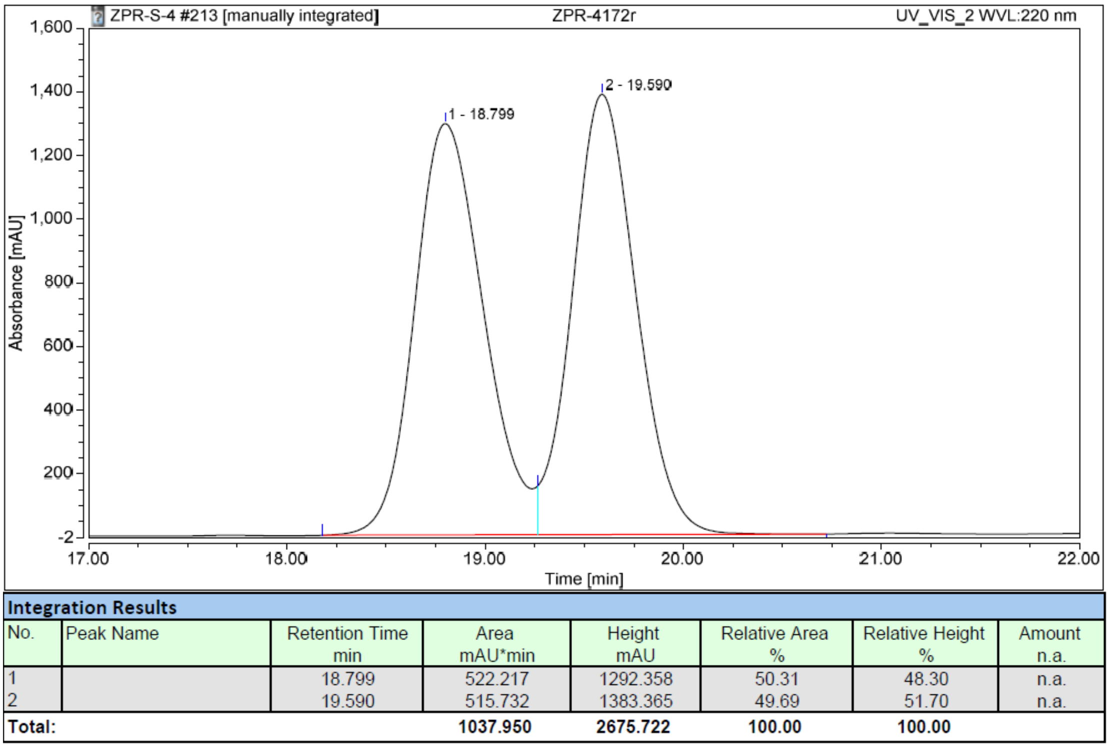

Integration Results

No.	Peak Name	Retention Time min	Area mAU*min	Height mAU	Relative Area %	Relative Height %	Amount n.a.
1		18.799	522.217	1292.358	50.31	48.30	n.a.
2		19.590	515.732	1383.365	49.69	51.70	n.a.
**Total:**			**1037.950**	**2675.722**	**100.00**	**100.00**	

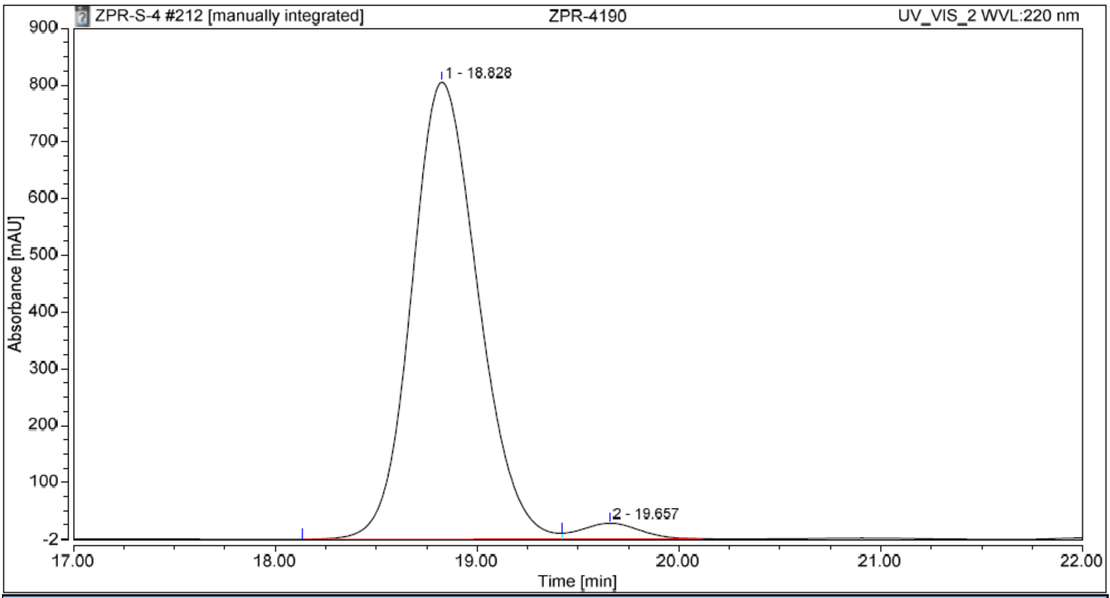

Integration Results

No.	Peak Name	Retention Time min	Area mAU*min	Height mAU	Relative Area %	Relative Height %	Amount n.a.
1		18.828	306.417	805.107	97.05	96.67	n.a.
2		19.657	9.304	27.702	2.95	3.33	n.a.
**Total:**			**315.720**	**832.809**	**100.00**	**100.00**	

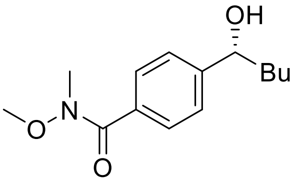

3f

The title compound **3f** was synthesized according to General Procedure B, and it was purified by column chromatography on silica gel (78% yield, 90% *ee*, 39.2 mg, colorless oil)

^{1}H NMR (400 MHz, Chloroform-*d*) δ 7.63 (s, 1H), 7.56 (d, *J* = 7.4 Hz, 1H), 7.45 (d, *J* = 7.8 Hz, 1H), 7.38 (dd, *J* = 7.4, 7.8 Hz, 1H), 4.73-4.68 (m, 1H), 3.56 (s, 3H), 3.36 (s, 3H), 2.09 (br, 1H), 1.85-1.72 (m, 2H), 1.47-1.26 (m, 4H), 0.88 (t, *J* = 7.0 Hz, 3H); ^{13}C NMR (151 MHz, Chloroform-*d*) δ 169.9, 144.9, 134.1, 128.1, 128.1, 127.1, 125.7, 74.3, 61.0, 38.8, 27.8, 22.5, 14.0; HRMS: m/z (ESI) calculated $[M+Na]^+$:274.1414, found: 274.1408. $[\alpha]_D^{25}$ = +19.152 (*c* = 0.25, $CHCl_3$).
The enantiomeric excess of **3f** was determined by chiral HPLC analysis compared to the corresponding racemate alcohol.
Conditions: ChiralPak IB column; hexane/^{i}PrOH = 95:5; flow rate = 1.0 mL/min; λ= 254 nm; t_{R1}(major)= 43.2min; t_{R2}(minor)= 41.4 min.

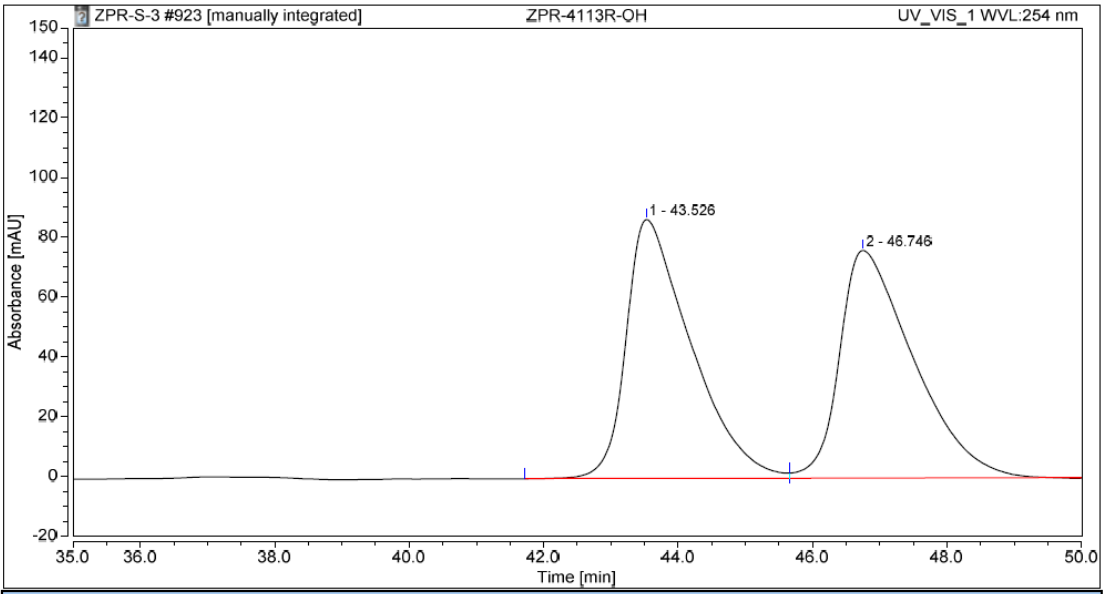

Integration Results

No.	Peak Name	Retention Time min	Area mAU*min	Height mAU	Relative Area %	Relative Height %	Amount n.a.
1		43.526	96.686	86.575	50.03	53.22	n.a.
2		46.746	96.588	76.096	49.97	46.78	n.a.
**Total:**			**193.275**	**162.671**	**100.00**	**100.00**	

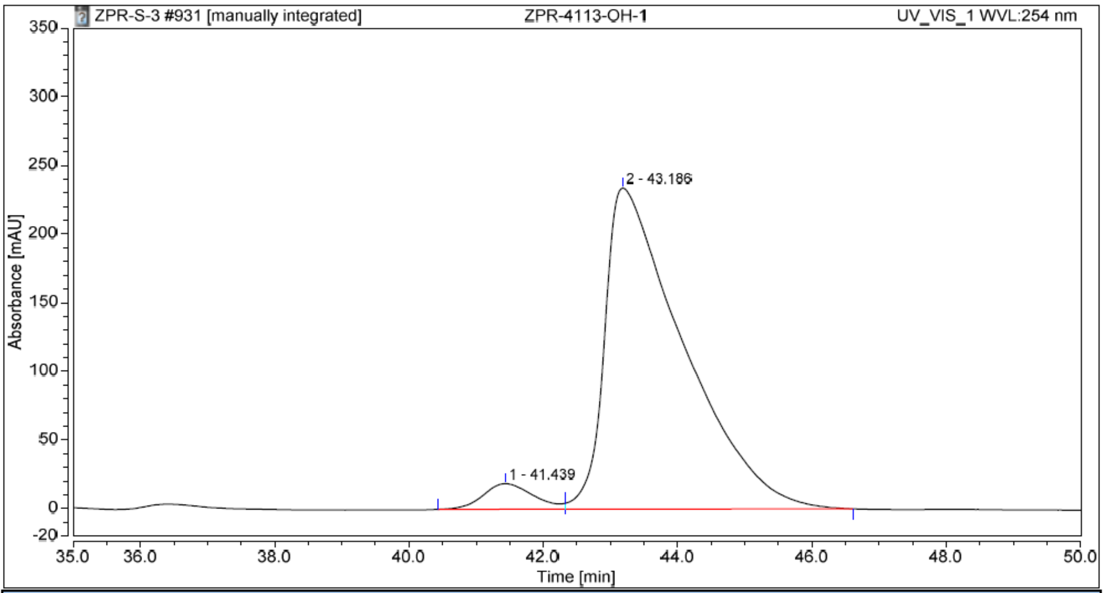

Integration Results

No.	Peak Name	Retention Time min	Area mAU*min	Height mAU	Relative Area %	Relative Height %	Amount n.a.
1		41.439	16.200	18.658	4.85	7.39	n.a.
2		43.186	317.652	233.963	95.15	92.61	n.a.
**Total:**			**333.852**	**252.621**	**100.00**	**100.00**	

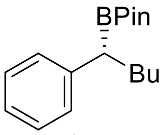

3g

The title compound **3g** was synthesized according to General Procedure B, and it was purified by column chromatography on silica gel (80% yield, 92% *ee*, 43.8 mg,

colorless oil).^{1}H NMR (400 MHz, Chloroform-*d*) δ 7.28-7.18 (m, 4H), 7.15-7.07 (m, 1H), 2.29 (dd, J = 8.0, 8.0 Hz, 1H), 1.93-1.78 (m, 1H), 1.71-1.57 (m, 1H), 1.37-1.24 (m, 4H), 1.20 (s, 6H), 1.18 (s, 6H), 0.86 (t, J = 7.0 Hz, 3H); ^{13}C NMR (101 MHz, Chloroform-*d*) δ 143.5, 128.3, 128.2, 125.0, 83.2, 32.3, 31.5, 24.6, 24.5, 22.7, 14.0; HRMS: m/z (ESI) calculated $[M+H]^+$: 275.2177, found: 275.2179. $[\alpha]_D^{25}$ = -22.987 (c = 0.25, $CHCl_3$), [ref [S6] (R-**3g**, 96% *ee*) $[\alpha]_D^{20}$ = -16 S17 (c = 0.11, $CHCl_3$),]. The absolute configuration of **3g** is assumed to be *R*, analogous to other products.

The enantiomeric excess of **3g** was determined by chiral HPLC analysis compared to the corresponding racemate alcohol.

Conditions: ChiralPak IG column; hexane/^{i}PrOH = 98:2; flow rate = 1.0 mL/min; λ= 220 nm; t_{R1}(major)= 14.1 min; t_{R2}(minor)= 13.2 min.

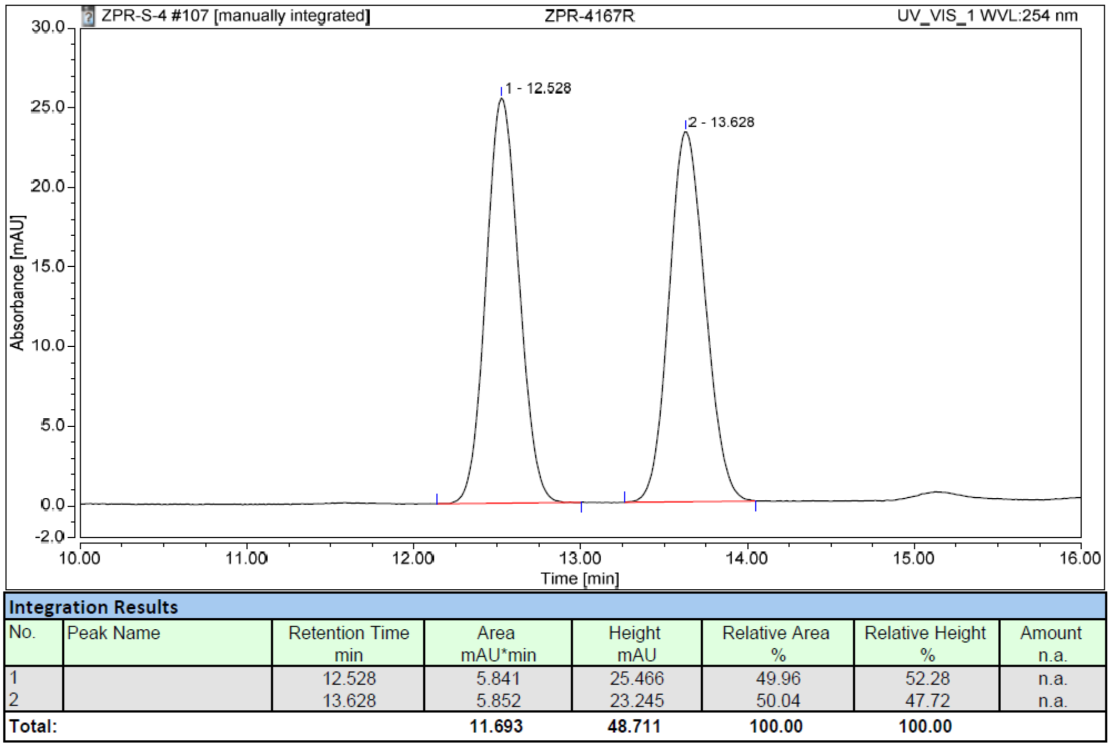

Integration Results							
No.	Peak Name	Retention Time min	Area mAU*min	Height mAU	Relative Area %	Relative Height %	Amount n.a.
1		12.528	5.841	25.466	49.96	52.28	n.a.
2		13.628	5.852	23.245	50.04	47.72	n.a.
**Total:**			**11.693**	**48.711**	**100.00**	**100.00**	

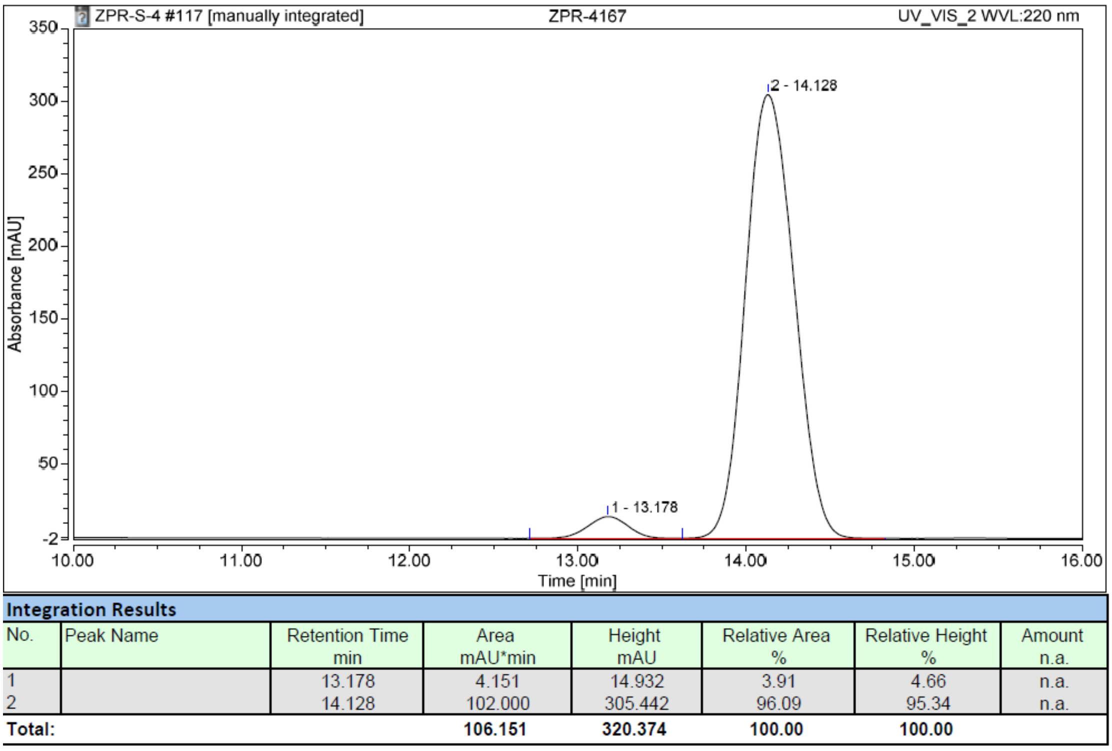

Integration Results

No.	Peak Name	Retention Time min	Area mAU*min	Height mAU	Relative Area %	Relative Height %	Amount n.a.
1		13.178	4.151	14.932	3.91	4.66	n.a.
2		14.128	102.000	305.442	96.09	95.34	n.a.
**Total:**			**106.151**	**320.374**	**100.00**	**100.00**	

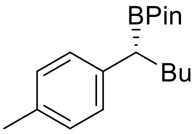

3h

The title compound **3h** was synthesized according to General Procedure B, and it was purified by column chromatography on silica gel (71% yield, 91% *ee*, 40.9 mg, colorless oil).

^{1}H NMR (400 MHz, Chloroform-*d*) δ 7.09 (d, *J* = 8.1 Hz, 2H), 7.05 (d, *J* = 8.1 Hz, 2H), 2.29 (s, 3H), 2.24 (dd, *J* = 7.9, 8.0 Hz, 1H), 1.87-1.75 (m, 1H), 1.67-1.56 (m, 1H), 1.34-1.22 (m, 4H), 1.20 (s, 6H), 1.19 (s, 6H), 0.85 (t, *J* = 7.1 Hz, 3H); ^{13}C NMR (101 MHz, Chloroform-d) δ 140.3, 134.3, 128.9, 128.2, 83.1, 32.5, 31.5, 24.6, 24.6, 22.7, 21.0, 14.0; HRMS: m/z (ESI) calculated [M+Na]$^{+}$: 311.2153, found: 311.2161. $[\alpha]_D^{25}$ = -10.576 (*c* = 0.25, $CHCl_3$).

The enantiomeric excess of **3h** was determined by chiral HPLC analysis compared to the corresponding racemate alcohol.

Conditions: ChiralPak IC column; hexane/^{i}PrOH = 98:2; flow rate = 1.0 mL/min; λ= 254; t_{R1}(major)= 12.8 min; t_{R2}(minor)= 13.9in.

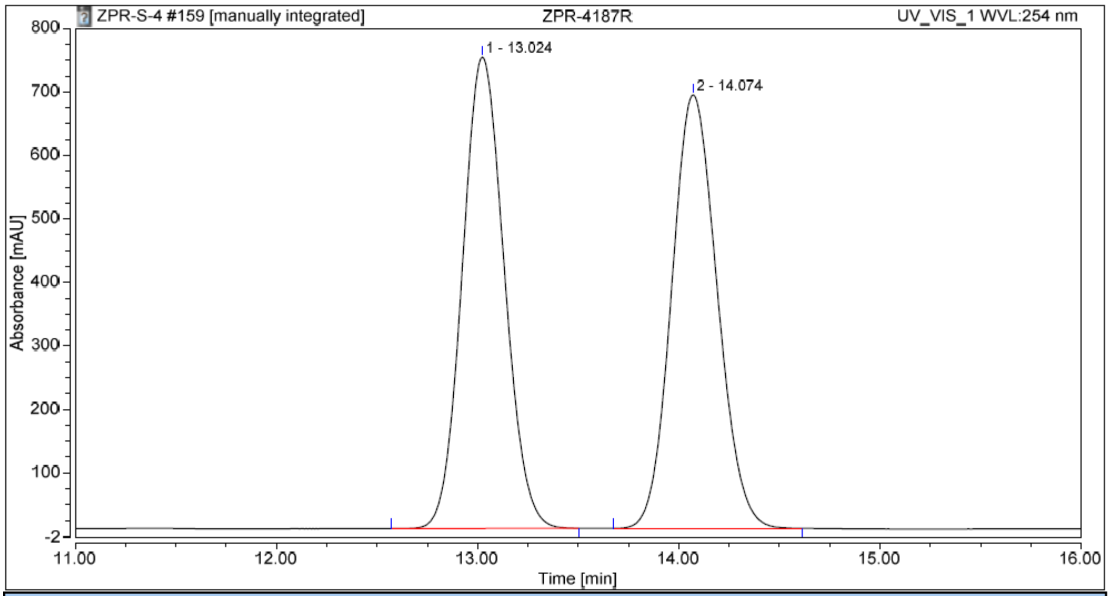

Integration Results

No.	Peak Name	Retention Time min	Area mAU*min	Height mAU	Relative Area %	Relative Height %	Amount n.a.
1		13.024	178.891	742.182	49.98	52.10	n.a.
2		14.074	179.045	682.449	50.02	47.90	n.a.
**Total:**			**357.935**	**1424.630**	**100.00**	**100.00**	

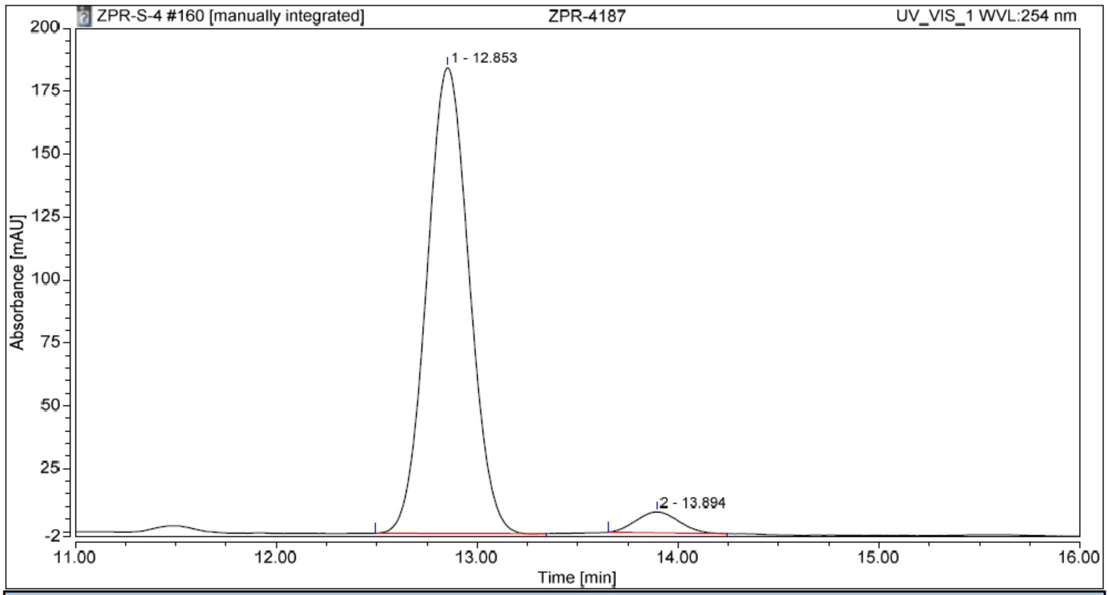

Integration Results

No.	Peak Name	Retention Time min	Area mAU*min	Height mAU	Relative Area %	Relative Height %	Amount n.a.
1		12.853	45.148	185.134	95.71	95.74	n.a.
2		13.894	2.023	8.236	4.29	4.26	n.a.
**Total:**			**47.170**	**193.370**	**100.00**	**100.00**	

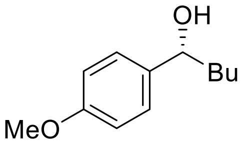

3i

The title compound **3i** was synthesized according to General Procedure B, and it was purified by column chromatography on silica gel (48% yield, 82% *ee*, 18.6 mg, colorless oil).

^{1}H NMR (400 MHz, Chloroform-*d*) δ 7.27 (d, J = 7.6 Hz, 2H), 6.88 (d, J = 7.6 Hz, 1H), 4.61 (dd, J = 6.8 Hz, 1H), 3.81 (s, 3H), 1.87-1.75 (m, 2H), 1.72-1.64 (m, 1H), 1.38-1.30 (m, 3H), 1.25-1.15 (m, 1H), 0.88 (t, J = 6.6 Hz, 3H); ^{13}C NMR (101 MHz, Chloroform-*d*) δ 159.0, 137.1, 127.1, 113.8, 74.3, 55.3, 38.7, 28.0, 22.6, 14.0; HRMS: m/z (EI) calculated $[M]^+$:194.1301, found: 194.1301. $[\alpha]_D^{25}$ = +15.747 (c = 0.25, $CHCl_3$).
The enantiomeric excess of **3i** was determined by chiral HPLC analysis compared to the corresponding racemate alcohol.
Conditions: ChiralPak IC column; hexane/^{i}PrOH = 90:10; flow rate = 1.0 mL/min; λ= 220 nm; t_{R1}(major)= 7.3 min; t_{R2}(minor)= 7.8 min.

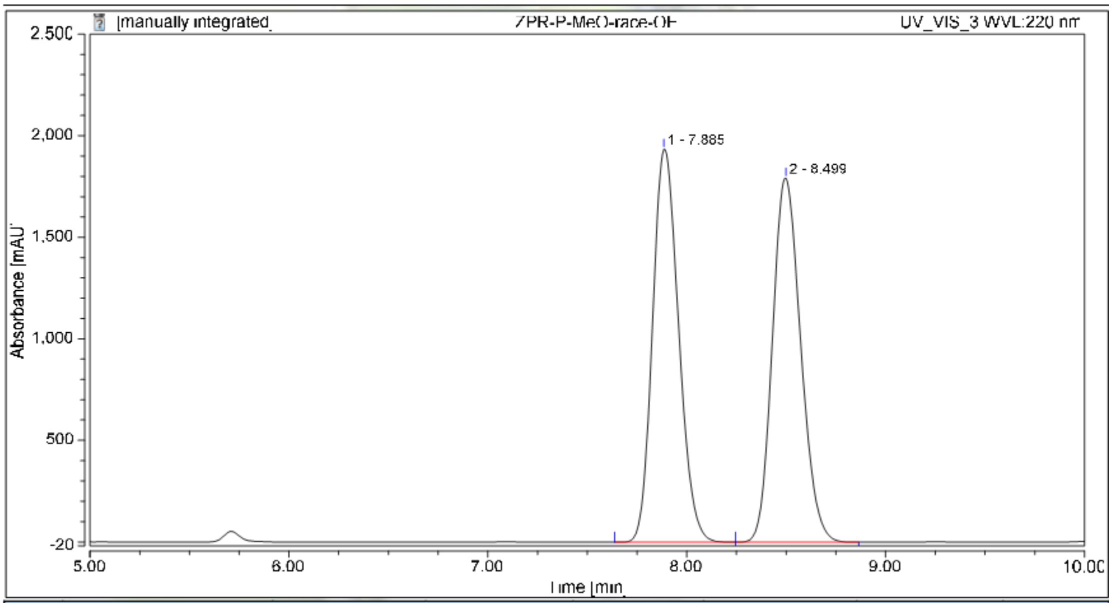

Integration Results							
No.	Peak Name	Retention Time min	Area mAU*min	Height mAU	Relative Area %	Relative Height %	Amount n.a.
1		7.885	293.106	1934.802	49.82	51.91	n.a.
2		8.499	295.279	1792.204	50.18	48.09	n.a.
Total:			588.385	3727.006	100.00	100.00	

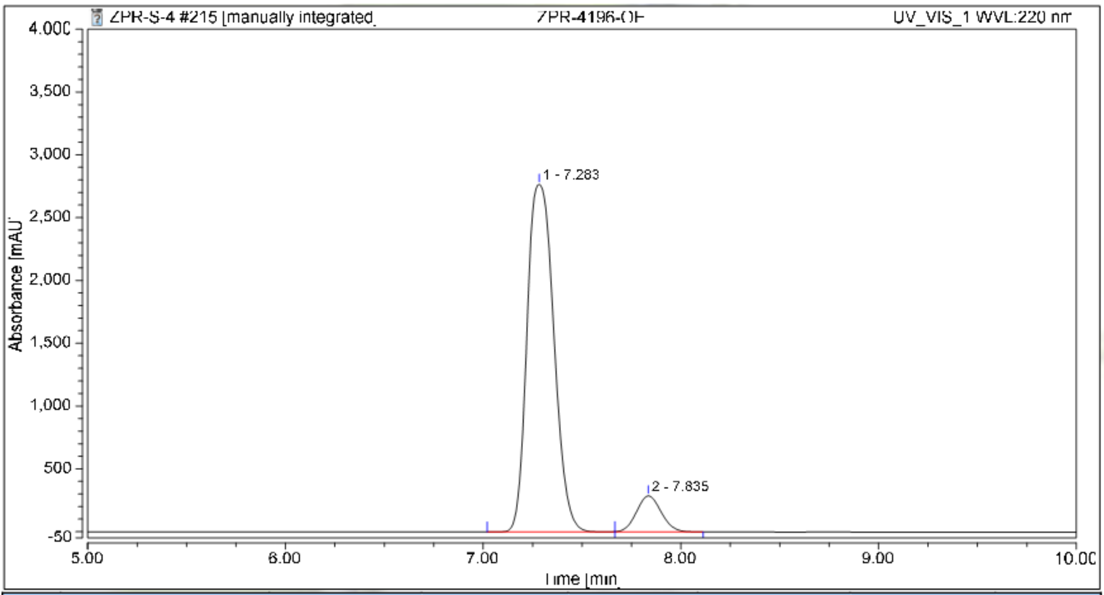

Integration Results							
No.	Peak Name	Retention Time min	Area mAU*min	Height mAU	Relative Area %	Relative Height %	Amount n.a.
1		7.283	434.502	2768.362	91.28	90.62	n.a.
2		7.835	41.493	286.706	8.72	9.38	n.a.
**Total:**			**475.995**	**3055.069**	**100.00**	**100.00**	

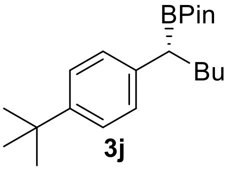

3j

The title compound **3j** was synthesized according to General Procedure B, and it was purified by column chromatography on silica gel (49% yield, 89% *ee*, 32.3 mg, colorless oil).

^{1}H NMR (400 MHz, Chloroform-d) δ 7.25 (d, *J* = 8.3 Hz, 2H), 7.12 (d, *J* = 8.3 Hz, 2H), 2.26 (dd, *J* = 7.9, 7.9 Hz, 1H), 1.87-1.76 (m, 1H), 1.67-1.57 (m, 1H), 1.29 (s, 9H), 1.28-1.24 (m, 4H), 1.21 (s, 6H), 1.20 (s, 6H), 0.86 (t, *J* = 7.0 Hz, 3H); ^{13}C NMR (101 MHz, Chloroform-d) δ 147.6, 140.2, 127.9, 125.1, 83.1, 34.2, 32.6, 31.6, 31.4, 24.6, 24.6, 22.7, 14.0; HRMS: m/z (ESI) calculated $[M+H]^+$: 331.2808, found: 331.2801. $[\alpha]_D^{25}$ = -13.303 (*c* = 0.25, $CHCl_3$).
The enantiomeric excess of **3j** was determined by chiral HPLC analysis compared to the corresponding racemate alcohol.
Conditions: ChiralPak IG column; hexane/^{i}PrOH = 98:2; flow rate = 1.0 mL/min; λ= 220 nm; t_{R1}(major)= 12.6 min; t_{R2}(minor)= 11.5 min.

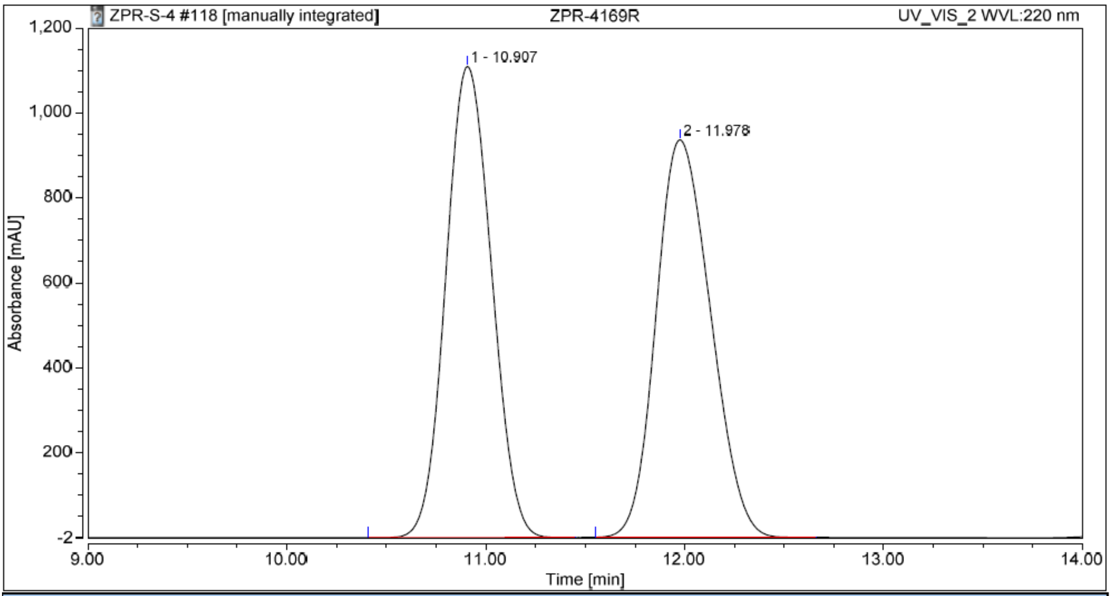

Integration Results

No.	Peak Name	Retention Time min	Area mAU*min	Height mAU	Relative Area %	Relative Height %	Amount n.a.
1		10.907	295.257	1109.608	49.84	54.24	n.a.
2		11.978	297.150	936.142	50.16	45.76	n.a.
**Total:**			**592.407**	**2045.750**	**100.00**	**100.00**	

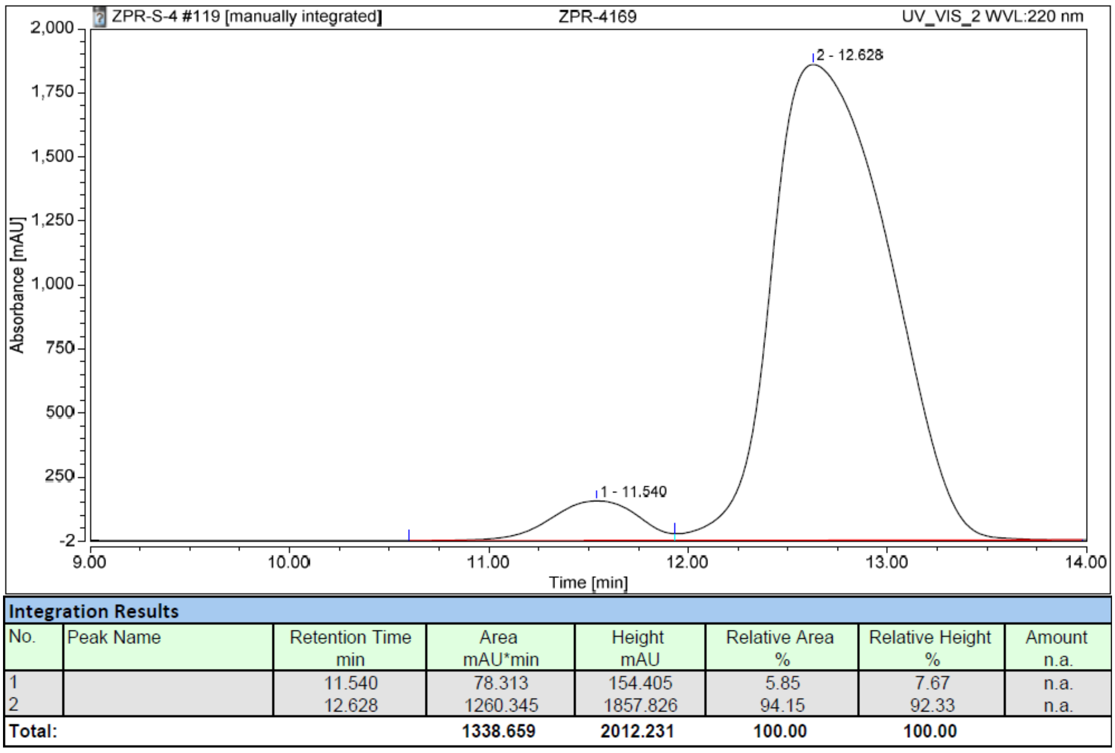

Integration Results

No.	Peak Name	Retention Time min	Area mAU*min	Height mAU	Relative Area %	Relative Height %	Amount n.a.
1		11.540	78.313	154.405	5.85	7.67	n.a.
2		12.628	1260.345	1857.826	94.15	92.33	n.a.
**Total:**			**1338.659**	**2012.231**	**100.00**	**100.00**	

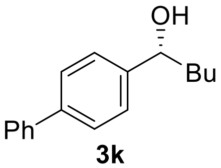

3k

The title compound 3k was synthesized according to General Procedure B, and it was

purified by column chromatography on silica gel (51% yield, 94% *ee*, 24.5 mg, white solid)

^{1}H NMR (400 MHz, Chloroform-*d*) δ 7.63-7.54 (m, 4H), 7.47-7.38 (m, 4H), 7.34 (dd, *J* = 7.6, 7.2 Hz, 1H), 4.70 (dd, *J* = 6.6, 6.8 Hz, 1H), 1.93 (br, 1H), 1.87-1.80 (m, 1H), 1.79-1.68 (m, 1H), 1.44-1.27 (m, 4H), 0.90 (t, *J* = 7.0 Hz, 3H); ^{13}C NMR (101 MHz, Chloroform-*d*) δ 143.9, 140.8, 140.4, 128.7, 127.2, 127.1, 127.0, 126.3, 74.4, 38.8, 28.0, 22.6, 14.0; HRMS: m/z (ESI) calculated [M]$^{+}$:240.1514, found: 240.1510. $[\alpha]_D^{25}$ = +33.961 (c = 0.25, $CHCl_3$)
The enantiomeric excess of **3k** was determined by chiral HPLC analysis compared to the corresponding racemate alcohol.
Conditions: ChiralPak IG column; hexane/^{i}PrOH = 98:2; flow rate = 1.0 mL/min; λ= 220 nm; t_{R1}(major)= 26.5 min; t_{R2}(minor)= 27.7 min.

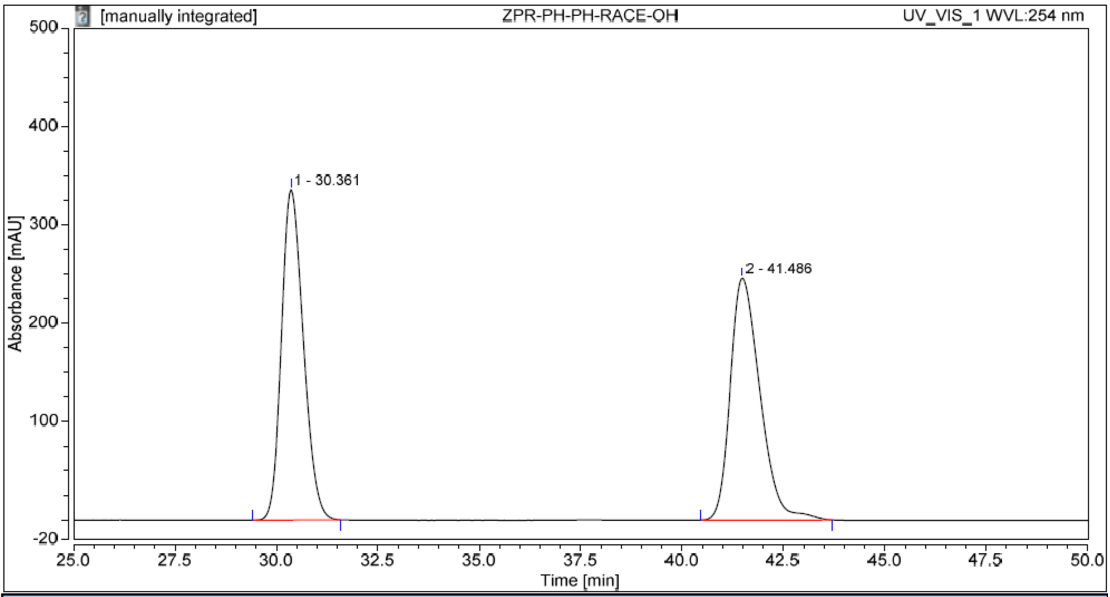

Integration Results

No.	Peak Name	Retention Time min	Area mAU*min	Height mAU	Relative Area %	Relative Height %	Amount n.a.
1		30.361	210.091	336.037	50.08	57.71	n.a.
2		41.486	209.420	246.205	49.92	42.29	n.a.
**Total:**			**419.511**	**582.241**	**100.00**	**100.00**	

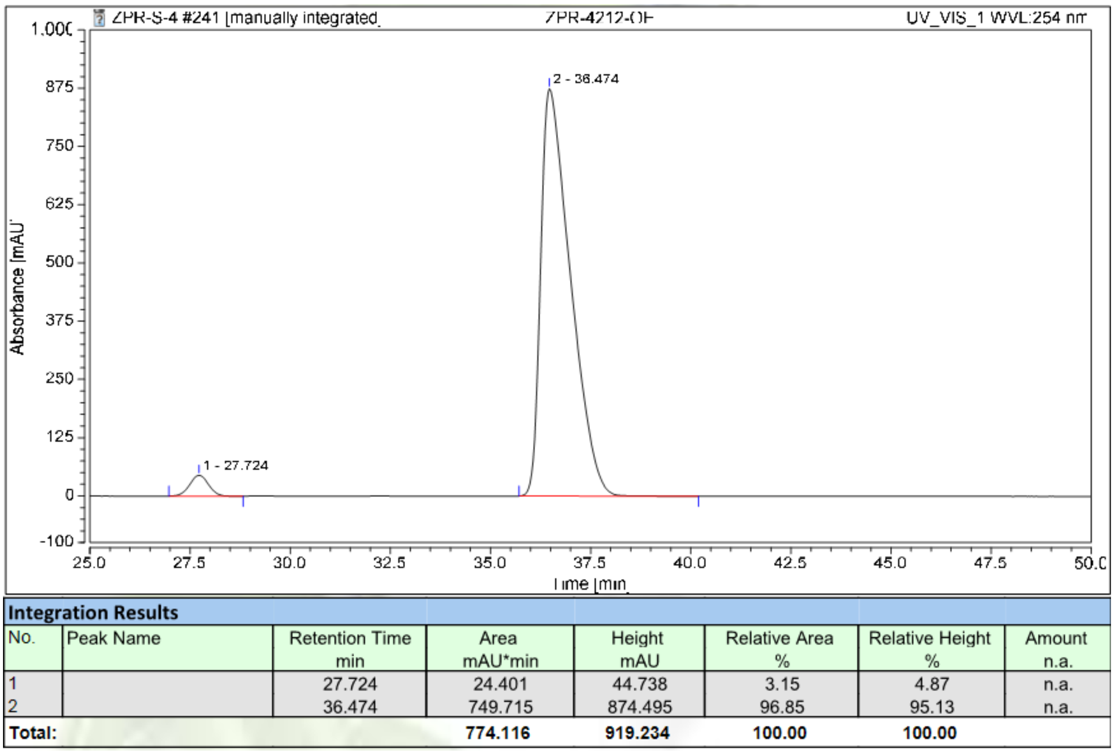

Integration Results							
No.	Peak Name	Retention Time min	Area mAU*min	Height mAU	Relative Area %	Relative Height %	Amount n.a.
1		27.724	24.401	44.738	3.15	4.87	n.a.
2		36.474	749.715	874.495	96.85	95.13	n.a.
**Total:**			**774.116**	**919.234**	**100.00**	**100.00**	

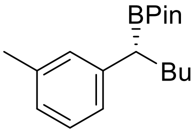

3l

The title compound **3l** was synthesized according to General Procedure B, and it was purified by column chromatography on silica gel (69% yield, 94% *ee*, 39.7 mg, colorless oil)

^{1}H NMR (400 MHz, Chloroform-*d*) δ 7.13 (dd, *J* = 7.6, 7.4 Hz, 1H), 7.04-6.98 (m, 2H), 6.94 (d, *J* = 7.6 Hz, 1H), 2.31 (s, 3H), 2.25 (dd, *J* = 7.8, 7.8 Hz, 1H), 1.88-1.78 (m, 1H), 1.66-1.60 (m, 1H), 1.34-1.24 (m, 4H), 1.21 (s, 6H), 1.19 (s, 6H), 0.86 (t, *J* = 7.0 Hz, 3H); ^{13}C NMR (101 MHz, Chloroform-*d*) δ 143.4, 137.6, 129.2, 128.0, 125.8, 125.3, 83.2, 32.4, 31.6, 24.6, 24.6, 22.7, 21.4, 14.0; HRMS: m/z (ESI) calculated $[M+H]^+$:289.2333, found: 289.2329. $[\alpha]_D^{25}$ = -19.643 (*c* = 0.25, $CHCl_3$).
The enantiomeric excess of **3l** was determined by chiral HPLC analysis compared to the corresponding racemate alcohol.
Conditions: ChiralPak IG column; hexane/^{i}PrOH = 99:1; flow rate = 1.0 mL/min; λ= 220 nm; t_{R1}(major)= 24.9 min; t_{R2}(minor)= 21.8 min.

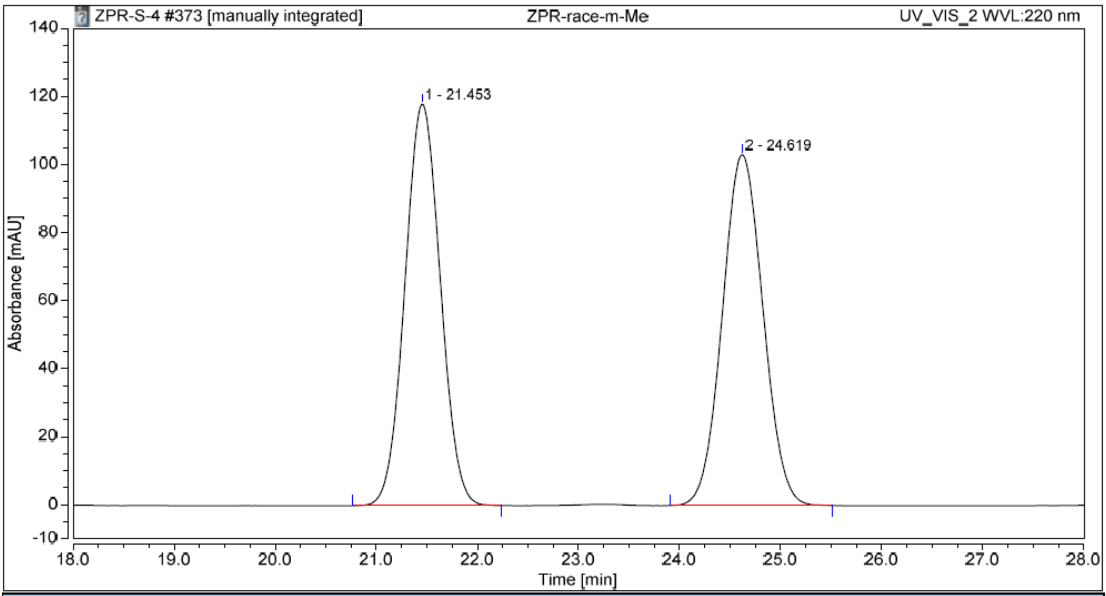

Integration Results

No.	Peak Name	Retention Time min	Area mAU*min	Height mAU	Relative Area %	Relative Height %	Amount n.a.
1		21.453	47.956	118.048	49.95	53.35	n.a.
2		24.619	48.055	103.222	50.05	46.65	n.a.
**Total:**			**96.011**	**221.270**	**100.00**	**100.00**	

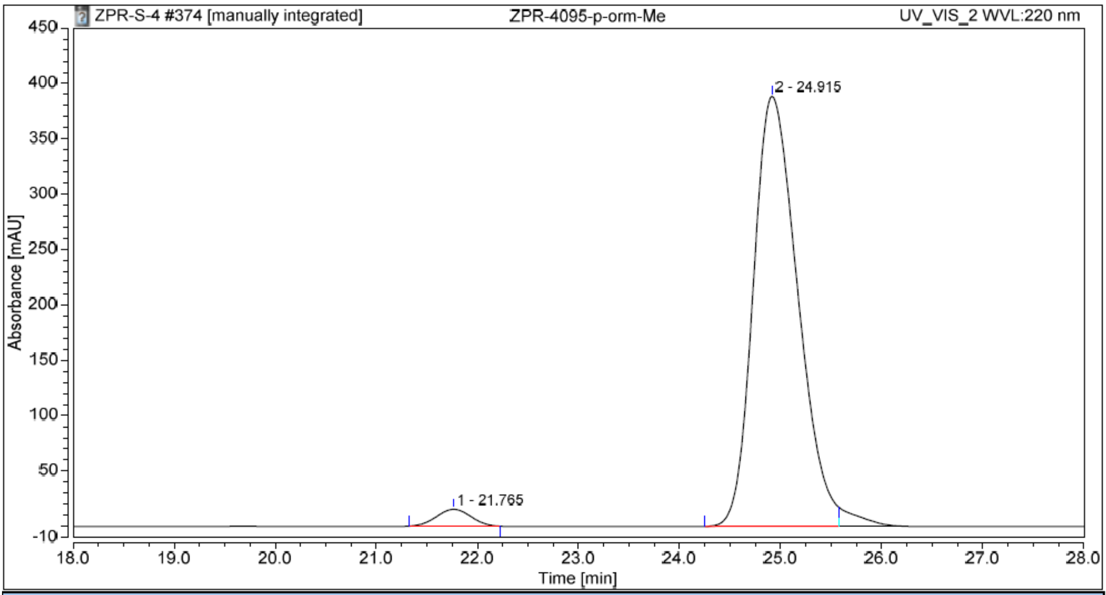

Integration Results

No.	Peak Name	Retention Time min	Area mAU*min	Height mAU	Relative Area %	Relative Height %	Amount n.a.
1		21.765	5.982	15.222	2.95	3.77	n.a.
2		24.915	196.724	388.254	97.05	96.23	n.a.
**Total:**			**202.706**	**403.476**	**100.00**	**100.00**	

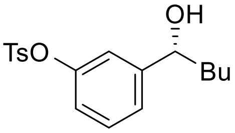

3m

The title compound **3m** was synthesized according to General Procedure B, and it was purified by column chromatography on silica gel (76% yield, 94% *ee*, 50.8 mg, colorless oil).

^{1}H NMR (400 MHz, Chloroform-*d*) δ 7.68 (d, *J* = 8.3 Hz, 2H), 7.35-7.24 (m, 3H), 7.22-7.17 (m, 1H), 6.94-6.86 (m, 2H), 4.57 (dd, *J* = 7.4, 5.7 Hz, 1H), 2.44 (s, 3H), 2.10 (s, 1H), 1.72-1.59 (m, 1H), 1.59-1.46 (m, 1H), 1.36-1.23 (m, 3H), 1.20-1.09 (m, 1H), 0.87 (t, *J* = 7.0 Hz, 3H); ^{13}C NMR (101 MHz, Chloroform-*d*) δ 149.6, 147.1, 145.3, 132.3, 129.7, 129.5, 128.5, 124.5, 121.2, 119.8, 73.7, 38.7, 27.6, 22.5, 21.6, 13.9; HRMS: m/z (ESI) calculated $[M+Na]^+$: 357.1131, found: 357.1129. $[\alpha]_D^{25}$ = 30.317 (*c* = 0.25, $CHCl_3$).
The enantiomeric excess of **3m** was determined by chiral HPLC analysis compared to the corresponding racemate alcohol.
Conditions: ChiralPak IG column; hexane/^{i}PrOH = 90:0; flow rate = 1.0 mL/min; λ= 220 nm; t_{R1}(major)= 41.2 min; t_{R2}(minor)= 50.9 min.

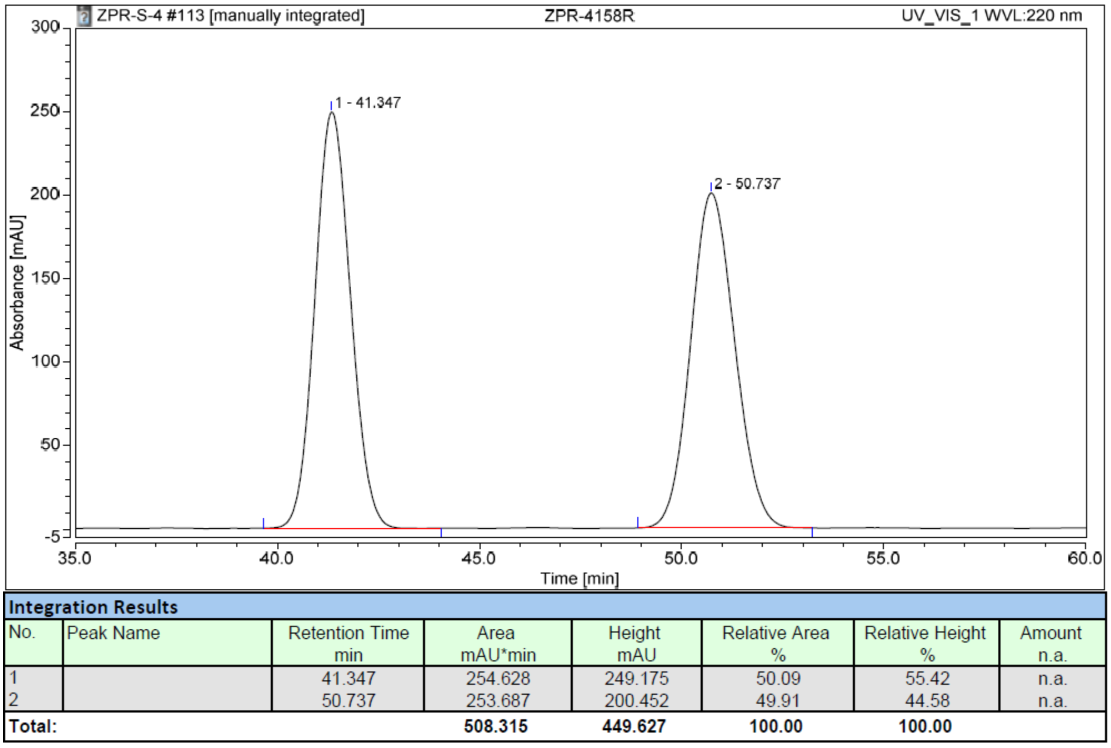

Integration Results

No.	Peak Name	Retention Time min	Area mAU*min	Height mAU	Relative Area %	Relative Height %	Amount n.a.
1		41.347	254.628	249.175	50.09	55.42	n.a.
2		50.737	253.687	200.452	49.91	44.58	n.a.
**Total:**			**508.315**	**449.627**	**100.00**	**100.00**	

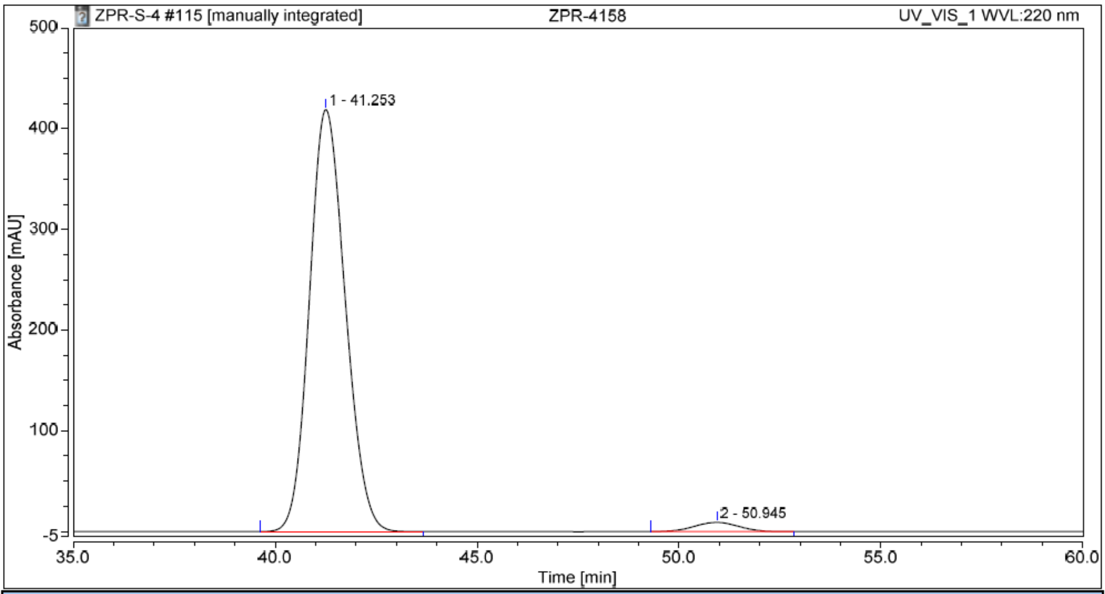

Integration Results

No.	Peak Name	Retention Time min	Area mAU*min	Height mAU	Relative Area %	Relative Height %	Amount n.a.
1		41.253	430.802	419.132	97.39	97.87	n.a.
2		50.945	11.539	9.104	2.61	2.13	n.a.
**Total:**			**442.341**	**428.236**	**100.00**	**100.00**	

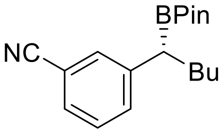

3n

The title compound **3n** was synthesized according to General Procedure B, and it was purified by column chromatography on silica gel (67% yield, 93% *ee*, 40.1 mg, colorless oil)

^{1}H NMR (400 MHz, Chloroform-*d*) δ 7.51 (s, 1H), 7.46-7.40 (m, 2H), 7.34 (dd, *J* = 7.6, 8.0 Hz, 1H), 2.33 (t, *J* = 8.0, 8.0 Hz, 1H), 1.91-1.78 (m, 1H), 1.68-1.56 (m, 1H), 1.40-1.24 (m, 4H), 1.21 (s, 6H), 1.20 (s, 6H), 0.86 (t, *J* = 7.2 Hz, 3H); ^{13}C NMR (101 MHz, Chloroform-*d*) δ 145.1, 133.0, 131.8, 128.9, 128.9, 119.3, 112.1, 83.6, 31.9, 31.3, 24.6, 24.5, 22.5, 13.9; HRMS: m/z (ESI) calculated $[M+H]^+$:190.1226, found: 190.1230. $[\alpha]_D^{25}$ = -18.665 (*c* = 0.25, $CHCl_3$).
The enantiomeric excess of **3n** was determined by chiral HPLC analysis compared to the corresponding racemate alcohol.
Conditions: ChiralPak IG column; hexane/^{i}PrOH = 90:0; flow rate = 1.0 mL/min; λ= 220 nm; t_{R1}(major)= 9.4 min; t_{R2}(minor)= 10.2 min.

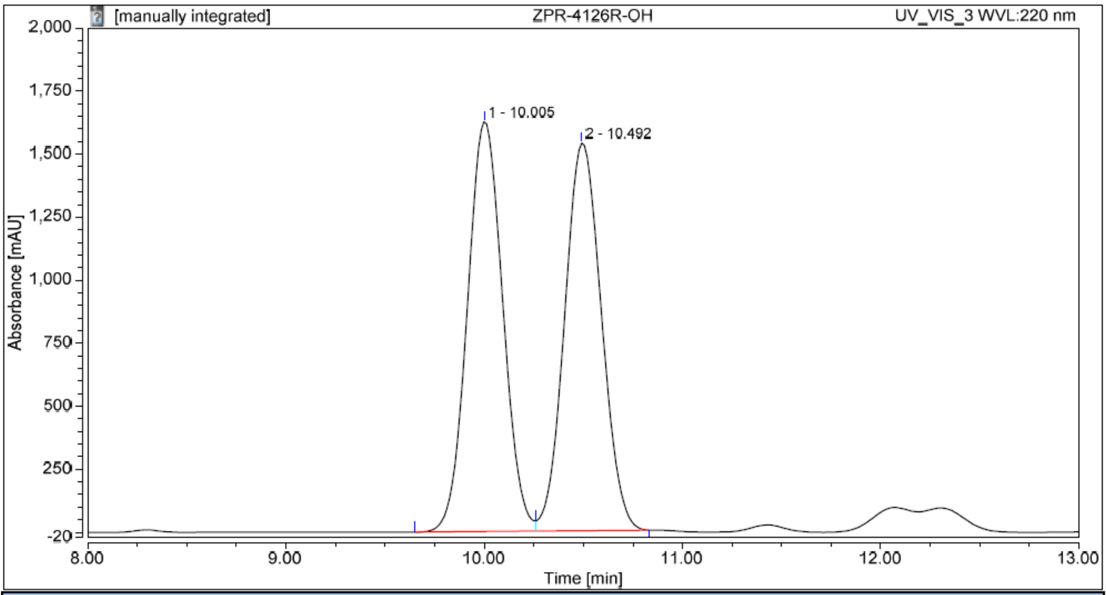

Integration Results							
No.	Peak Name	Retention Time min	Area mAU*min	Height mAU	Relative Area %	Relative Height %	Amount n.a.
1		10.005	335.939	1625.696	50.40	51.40	n.a.
2		10.492	330.583	1537.282	49.60	48.60	n.a.
**Total:**			**666.521**	**3162.977**	**100.00**	**100.00**	

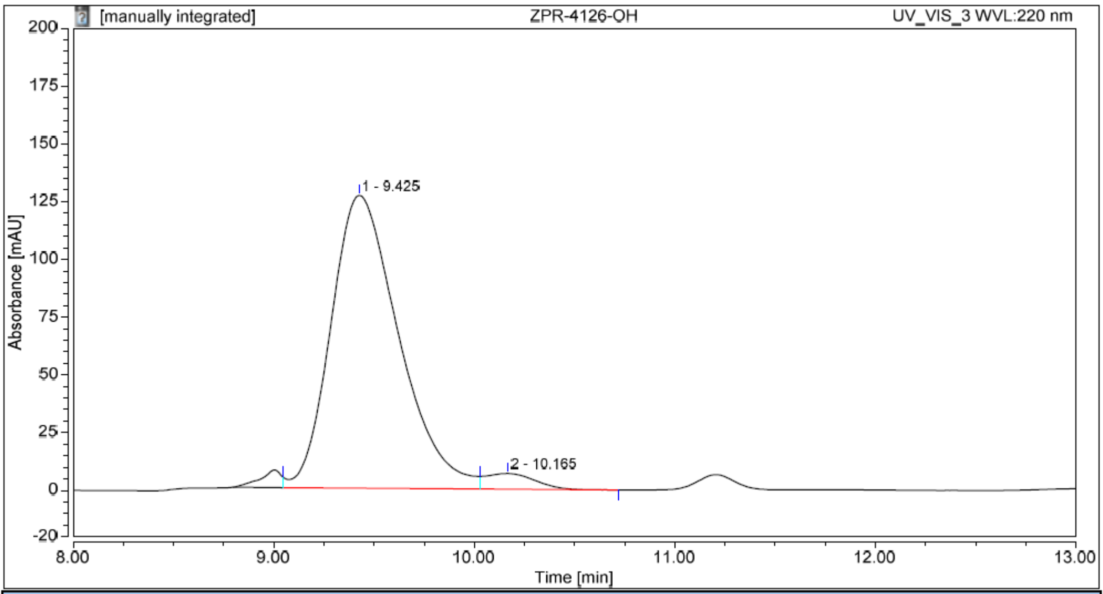

Integration Results							
No.	Peak Name	Retention Time min	Area mAU*min	Height mAU	Relative Area %	Relative Height %	Amount n.a.
1		9.425	49.584	126.734	96.33	94.94	n.a.
2		10.165	1.888	6.752	3.67	5.06	n.a.
**Total:**			**51.471**	**133.486**	**100.00**	**100.00**	

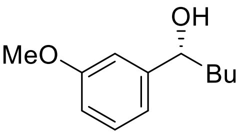

3o

The title compound **3o** was synthesized according to General Procedure B, and it was purified by column chromatography on silica gel (40% yield, 91% *ee*, 15.5 mg,

colorless oil)

^{1}H NMR (400 MHz, Chloroform-*d*) δ 7.33-7.21 (m, 1H), 6.97-6.86 (m, 2H), 6.81 (dd, *J* = 8.4, 2.5 Hz, 1H), 4.64 (dd, *J* = 6.6, 6.6 Hz, 1H), 3.81 (s, 3H), 1.91 (br, 1H), 1.85-1.75 (m, 1H), 1.75-1.66 (m, 1H), 1.45-1.27 (m, 4H), 0.89 (t, *J* = 7.0 Hz, 3H); ^{13}C NMR (101 MHz, Chloroform-*d*) δ 159.7, 146.7, 129.4, 118.2, 112.9, 111.4, 74.6, 55.2, 38.8, 28.0, 22.6, 14.0; HRMS: m/z (ESI) calculated $[M+Na]^+$:217.1199, found: 217.1195. $[\alpha]_D^{25}$ = +24.333 (*c* = 0.25, $CHCl_3$).

The enantiomeric excess of **3o** was determined by chiral HPLC analysis compared to the corresponding racemate alcohol.

Conditions: ChiralPak IG column; hexane/^{i}PrOH = 95:5; flow rate = 1.0 mL/min; λ= 220 nm; t_{R1}(major)= 14.2 min; t_{R2}(minor)= 13.6 min.

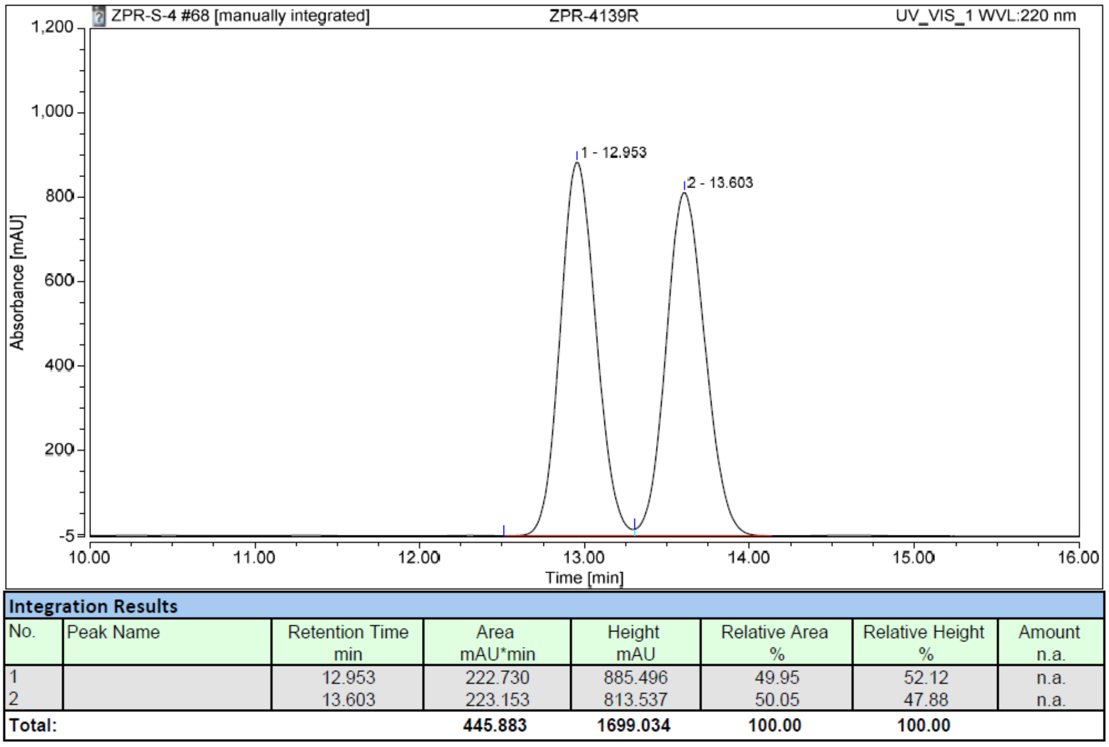

Integration Results

No.	Peak Name	Retention Time min	Area mAU*min	Height mAU	Relative Area %	Relative Height %	Amount n.a.
1		12.953	222.730	885.496	49.95	52.12	n.a.
2		13.603	223.153	813.537	50.05	47.88	n.a.
**Total:**			**445.883**	**1699.034**	**100.00**	**100.00**	

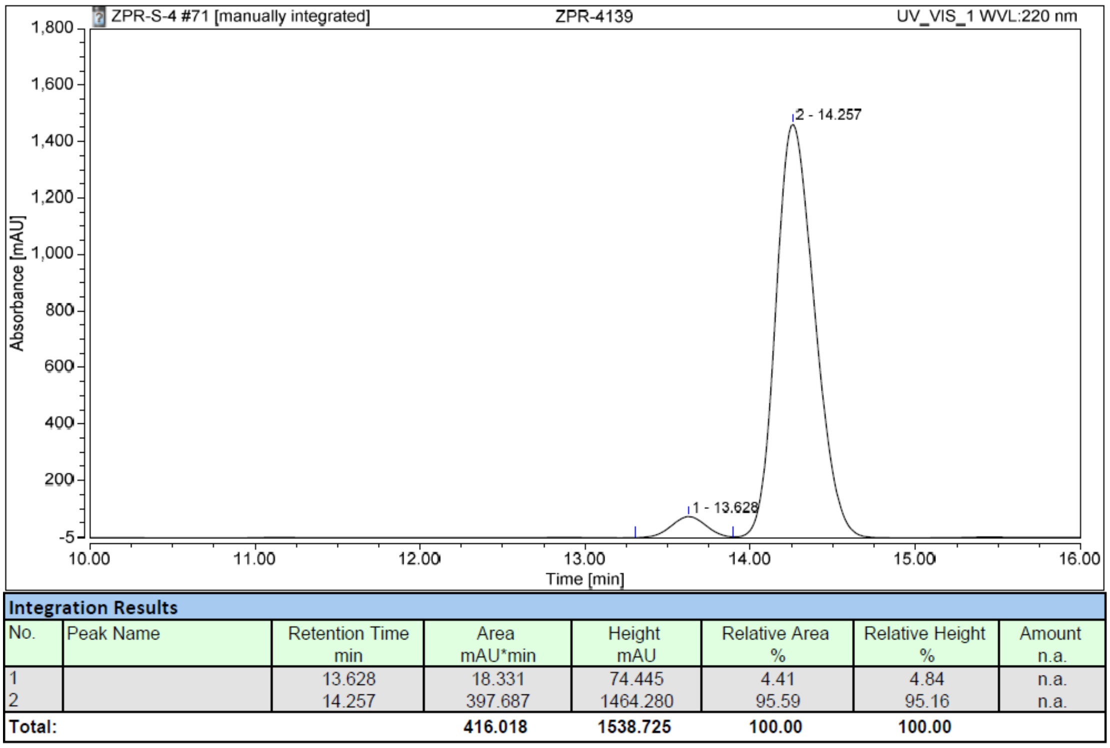

Integration Results							
No.	Peak Name	Retention Time min	Area mAU*min	Height mAU	Relative Area %	Relative Height %	Amount n.a.
1		13.628	18.331	74.445	4.41	4.84	n.a.
2		14.257	397.687	1464.280	95.59	95.16	n.a.
**Total:**			**416.018**	**1538.725**	**100.00**	**100.00**	

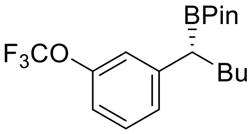

3p

The title compound **3p** was synthesized according to General Procedure B, and it was purified by column chromatography on silica gel (57% yield, 95% *ee*, 40.8 mg, colorless oil)

^{1}H NMR (600 MHz, Chloroform-*d*) δ 7.24 (dd, *J* = 7.8, 7.6 Hz, 1H), 7.13 (d, *J* = 7.6 Hz, 1H), 7.09 (s, 1H), 6.98 (d, *J* = 7.8 Hz, 1H), 2.32 (dd, *J* = 7.8, 7.8 Hz, 1H), 1.87-1.79 (m, 1H), 1.68-160 (m, 1H), 1.34-1.22 (m, 4H), 1.20 (s, 6H), 1.19 (s, 6H), 0.86 (t, *J* = 7.2 Hz, 3H); ^{13}C NMR (151 MHz, Chloroform-*d*) δ 149.3, 146.0, 129.3, 126.8, 120.8, 120.5 (q, *J* = 256.3 Hz), 117.6, 83.4, 32.1, 31.4, 24.5, 24.5, 22.6, 14.0; HRMS: m/z (ESI) calculated [M+Na]$^{+}$: 381.1819, found: 381.1832. $[\alpha]_D^{25}$ = -8.916 (*c* = 0.25, $CHCl_3$).

The enantiomeric excess of **3p** was determined by chiral HPLC analysis compared to the corresponding racemate alcohol.

Conditions: ChiralPak IG column; hexane/^{i}PrOH = 98:2; flow rate = 1.0 mL/min; λ= 220 nm; t_{R1}(major)= 6.8 min; t_{R2}(minor)= 7.1 min.

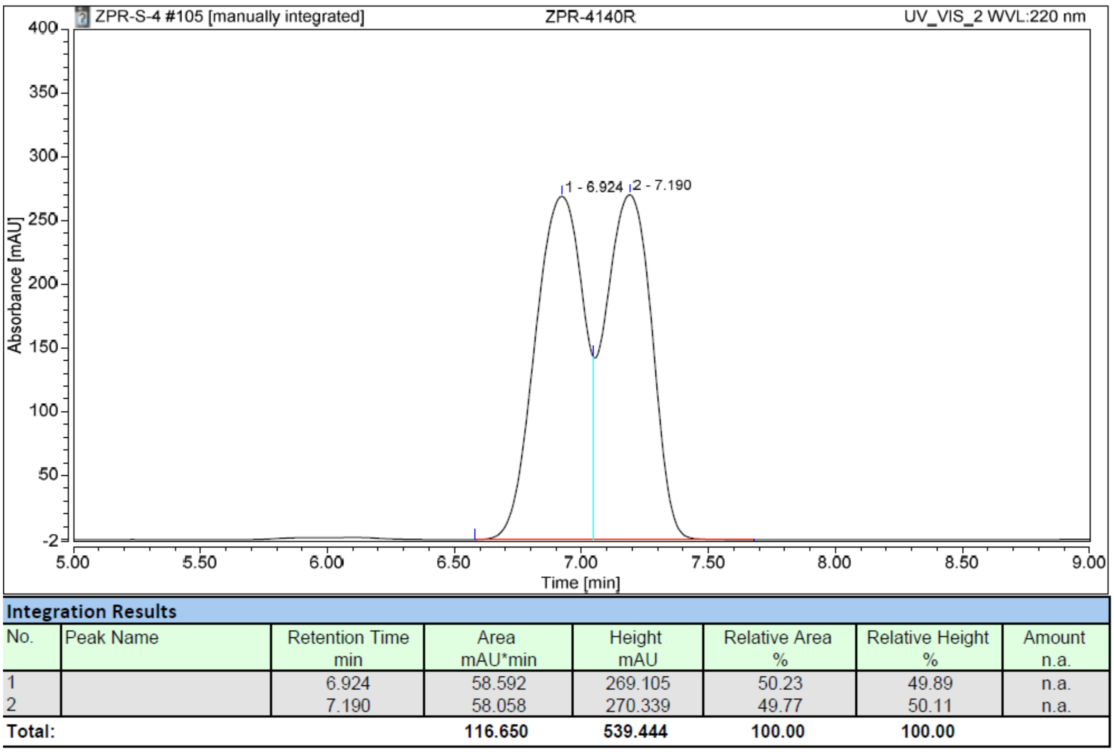

Integration Results

No.	Peak Name	Retention Time min	Area mAU*min	Height mAU	Relative Area %	Relative Height %	Amount n.a.
1		6.924	58.592	269.105	50.23	49.89	n.a.
2		7.190	58.058	270.339	49.77	50.11	n.a.
**Total:**			**116.650**	**539.444**	**100.00**	**100.00**	

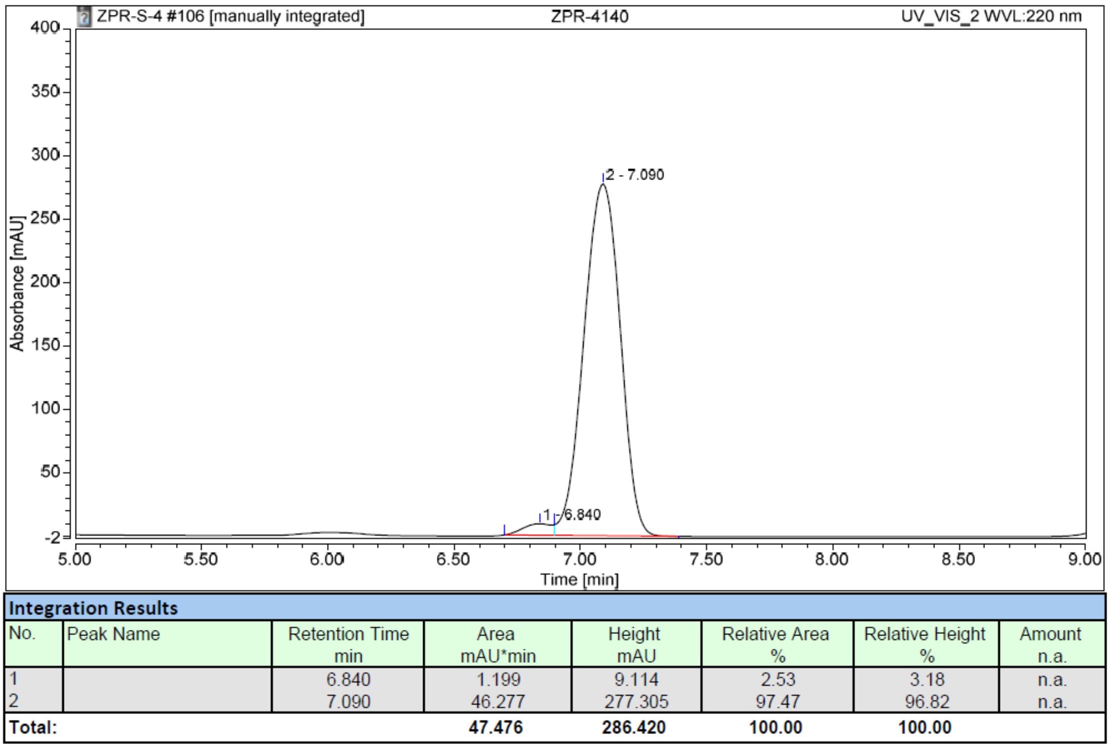

Integration Results

No.	Peak Name	Retention Time min	Area mAU*min	Height mAU	Relative Area %	Relative Height %	Amount n.a.
1		6.840	1.199	9.114	2.53	3.18	n.a.
2		7.090	46.277	277.305	97.47	96.82	n.a.
**Total:**			**47.476**	**286.420**	**100.00**	**100.00**	

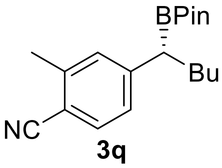

3q

The title compound **3q** was synthesized according to General Procedure B, and it was purified by column chromatography on silica gel (64% yield, 93% *ee*, 40.1 mg, white solid).

^{1}H NMR (400 MHz, Chloroform-*d*) δ 7.44 (d, *J* = 1.9 Hz, 1H), 7.31 (dd, *J* = 8.0, 1.9 Hz, 1H), 7.18 (d, *J* = 8.0 Hz, 1H), 2.49 (s, 3H), 2.28 (dd, *J* = 8.0, 8.0 Hz, 1H), 1.88-1.75 (m, 1H), 1.66-1.54 (m, 1H), 1.37-1.22 (m, 4H), 1.21 (s, 6H), 1.19 (s, 6H), 0.85 (t, *J* = 7.2 Hz, 3H); ^{13}C NMR (101 MHz, Chloroform-*d*) δ 141.9, 138.4, 132.9, 132.0, 130.0, 118.6, 112.4, 83.5, 32.0, 31.3, 24.6, 24.5, 22.5, 19.9, 13.9; HRMS: m/z (ESI) calculated $[M+H]^+$: 314.2286, found: 314.2289. $[\alpha]_D^{25}$ = -6.741 (*c* = 0.25, $CHCl_3$).
The enantiomeric excess of **3q** was determined by chiral HPLC analysis compared to the corresponding racemate alcohol.
Conditions: ChiralPak IA column; hexane/^{i}PrOH = 95:5; flow rate = 1.0 mL/min; λ= 220 nm; t_{R1}(major)= 14.7 min; t_{R2}(minor)= 16.5 min.

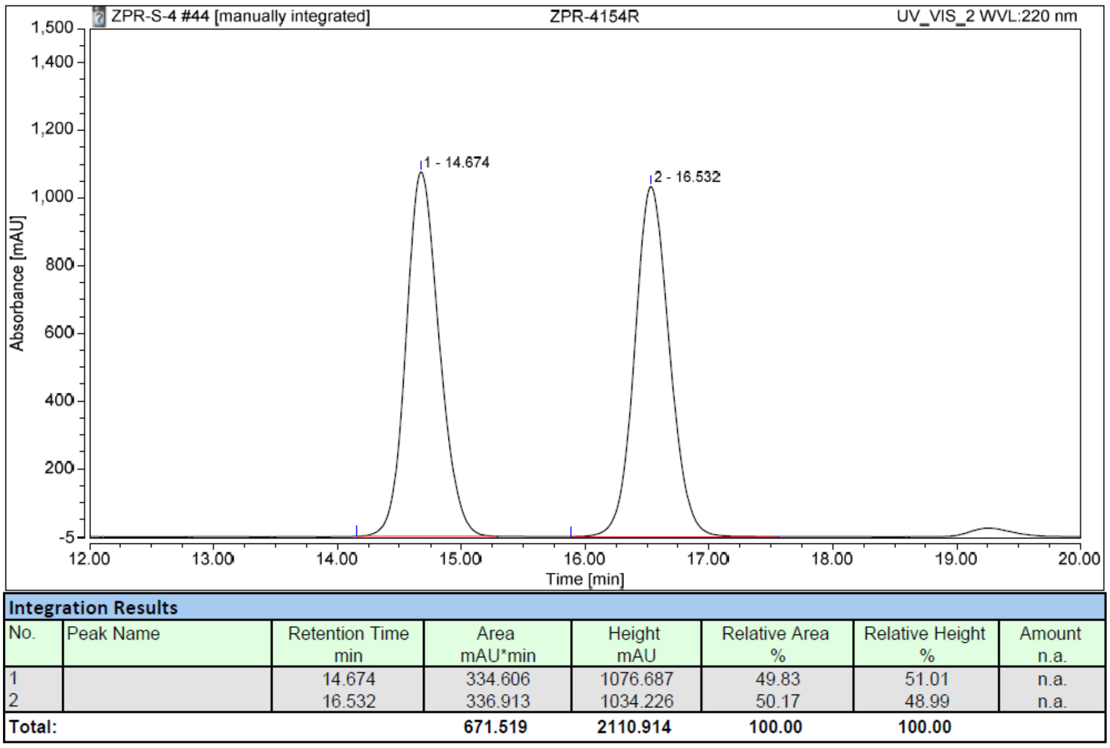

Integration Results

No.	Peak Name	Retention Time min	Area mAU*min	Height mAU	Relative Area %	Relative Height %	Amount n.a.
1		14.674	334.606	1076.687	49.83	51.01	n.a.
2		16.532	336.913	1034.226	50.17	48.99	n.a.
**Total:**			**671.519**	**2110.914**	**100.00**	**100.00**	

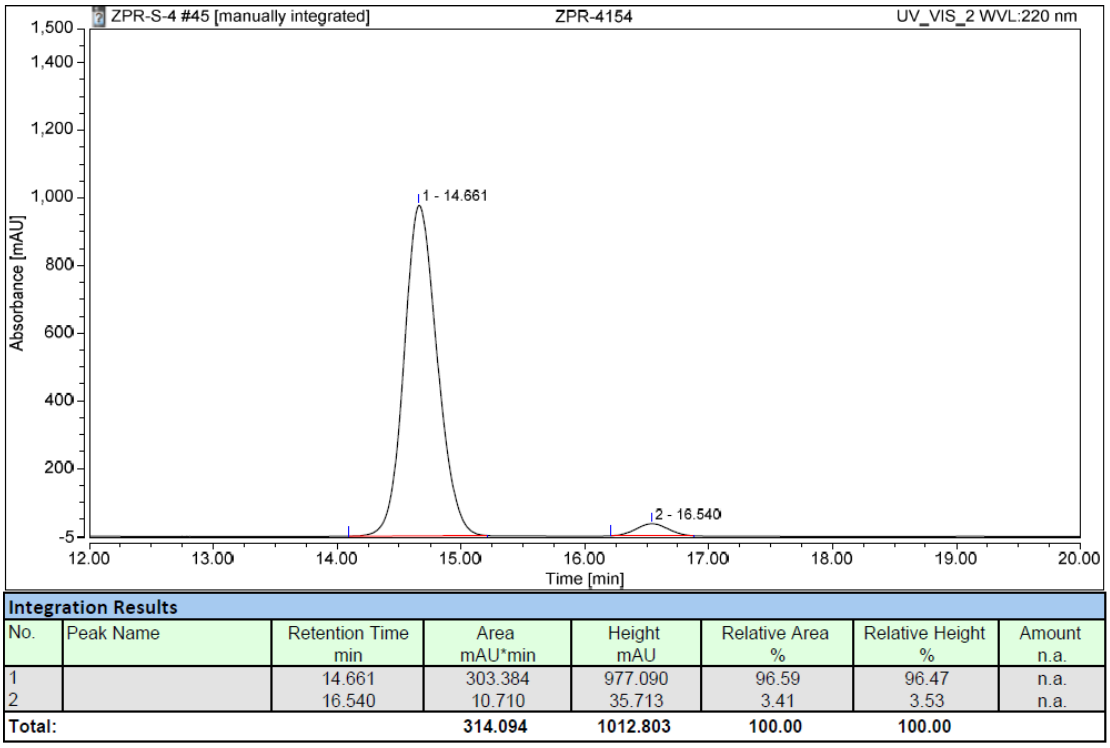

Integration Results

No.	Peak Name	Retention Time min	Area mAU*min	Height mAU	Relative Area %	Relative Height %	Amount n.a.
1		14.661	303.384	977.090	96.59	96.47	n.a.
2		16.540	10.710	35.713	3.41	3.53	n.a.
**Total:**			**314.094**	**1012.803**	**100.00**	**100.00**	

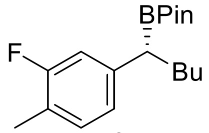

3r

The title compound **3r** was synthesized according to General Procedure B, and it was purified by column chromatography on silica gel (55% yield, 89% *ee*, 33.7 mg, colorless oil).

^{1}H NMR (600 MHz, Chloroform-*d*) δ 7.06-7.01 (m, 1H), 6.91- 6.83 (m, 2H), 2.27-2.22 (m, 1H), 2.21 (s, 3H), 1.84-1.77 (m, 1H), 1.63-1.57 (m, 1H), 1.29 (m, 4H), 1.21 (s, 6H), 1.19 (s, 6H), 0.85 (t, *J* = 7.2 Hz, 3H); ^{13}C NMR (151 MHz, Chloroform-*d*) δ 161.2 (d, *J* = 243.6 Hz), 143.2 (d, *J* = 7.1 Hz), 131.0 (d, *J* = 5.5 Hz), 123.7 (d, *J* = 3.0 Hz), 121.0 (d, *J* = 17.2 Hz), 114.6 (d, *J* = 21.8 Hz), 83.3, 32.2, 31.4, 24.6, 24.5, 22.6, 14.0; HRMS: m/z (ESI) calculated $[M+K]^+$:345.1798, found: 345.1803. $[\alpha]_D^{25}$ = -9.528 (*c* = 0.25, $CHCl_3$).

The enantiomeric excess of **3r** was determined by chiral HPLC analysis compared to the corresponding racemate alcohol.

Conditions: ChiralPak IC column; hexane/^{i}PrOH = 98:2; flow rate = 1.0 mL/min; λ= 220 nm; t_{R1}(major)= 23.7 min; t_{R2}(minor)= 27.6 min.

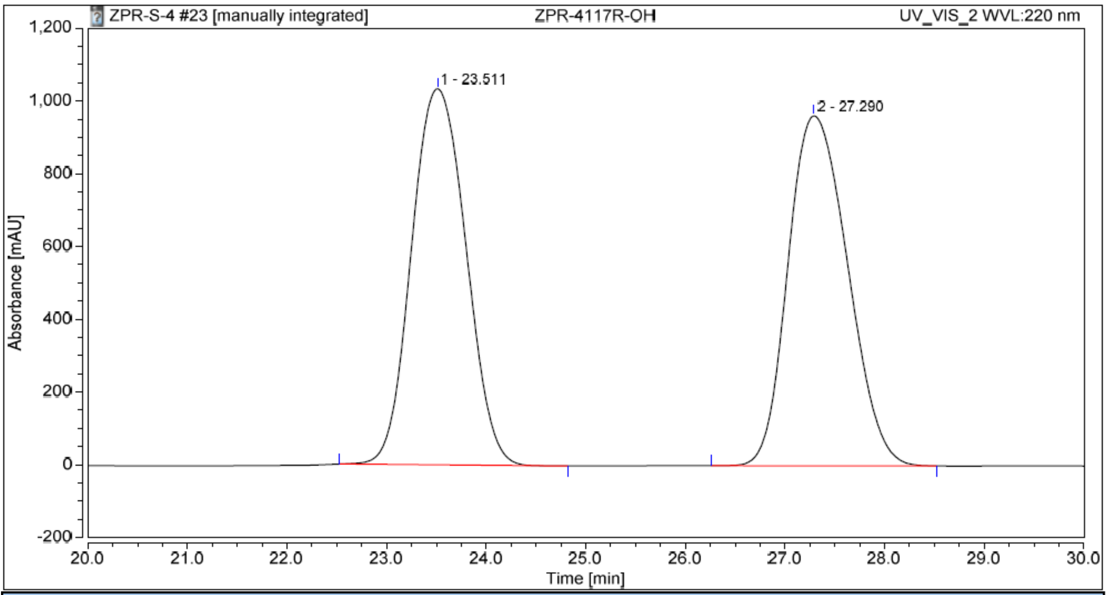

Integration Results							
No.	Peak Name	Retention Time min	Area mAU*min	Height mAU	Relative Area %	Relative Height %	Amount n.a.
1		23.511	654.632	1033.453	49.55	51.80	n.a.
2		27.290	666.654	961.782	50.45	48.20	n.a.
**Total:**			**1321.286**	**1995.236**	**100.00**	**100.00**	

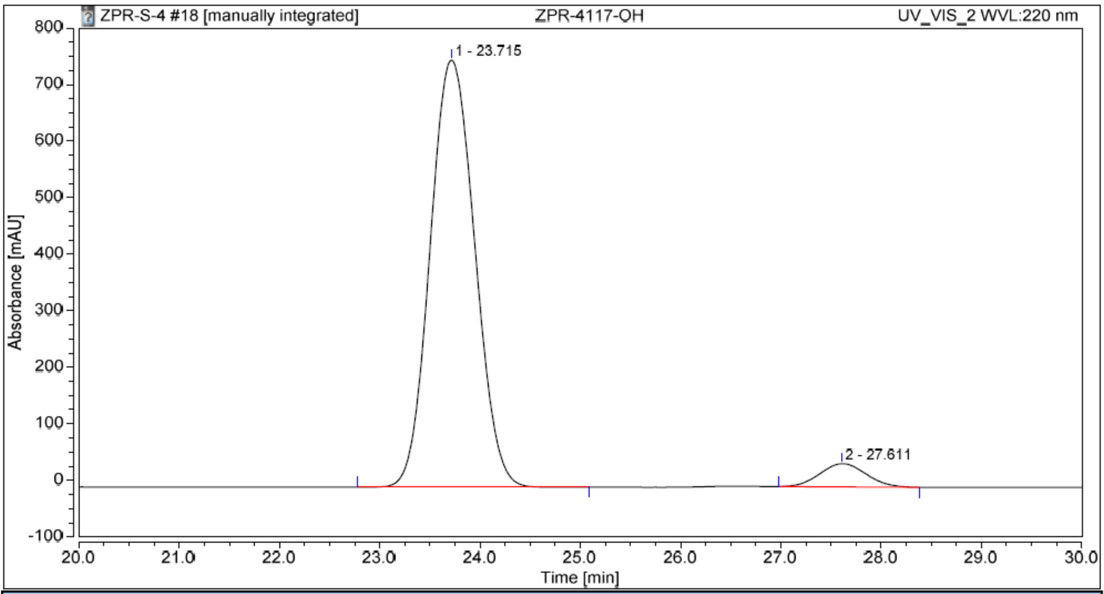

Integration Results							
No.	Peak Name	Retention Time min	Area mAU*min	Height mAU	Relative Area %	Relative Height %	Amount n.a.
1		23.715	389.046	755.612	94.69	94.86	n.a.
2		27.611	21.802	40.958	5.31	5.14	n.a.
**Total:**			**410.848**	**796.570**	**100.00**	**100.00**	

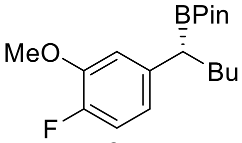

3s

The title compound **3s** was synthesized according to General Procedure B, and it was purified by column chromatography on silica gel (51% yield, 89% *ee*, 32.8 mg,

colorless oil)

^{1}H NMR (600 MHz, Chloroform-*d*) δ 6.94 (dd, *J* = 11.4, 8.2 Hz, 1H), 6.82 (dd, *J* = 8.2, 2.0 Hz, 1H), 6.75-6.68 (m, 1H), 3.87 (s, 3H), 2.24 (dd, *J* = 8.0, 8.0 Hz, 1H), 1.85 -1.77 (m, 1H), 1.64-1.56 (m, 1H), 1.36-1.23 (m, 4H), 1.21 (s, 6H), 1.20 (s, 6H), 0.86 (t, *J* = 7.2 Hz, 3H); ^{13}C NMR (151 MHz, Chloroform-*d*) δ 150.4 (d, *J* = 242.0 Hz), 147.1 (d, *J* = 10.6 Hz), 139.7 (d, *J* = 3.6 Hz), 120.3 (d, *J* = 6.5 Hz), 115.5 (d, *J* = 17.8 Hz), 113.4, 83.3, 56.1, 32.4, 31.4, 24.6, 24.6, 22.6, 14.0; HRMS: m/z (ESI) calculated $[M+NH_4]^+$: 340.2454, found: 340.2452. $[\alpha]_D^{25}$ = -9.846 (*c* = 0.25, $CHCl_3$).
The enantiomeric excess of **3s** was determined by chiral HPLC analysis compared to the corresponding racemate alcohol.
Conditions: ChiralPak IG column; hexane/^{i}PrOH = 90:10; flow rate = 1.0 mL/min; λ= 272 nm; t_{R1}(major)= 6.4 min; t_{R2}(minor)= 6.7 min.

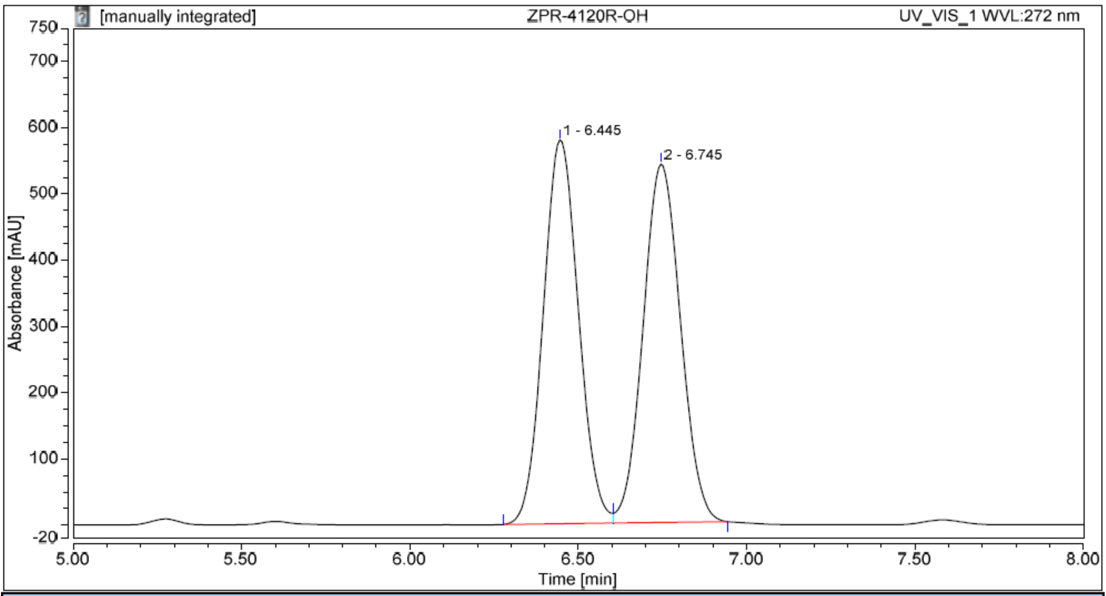

Integration Results

No.	Peak Name	Retention Time min	Area mAU*min	Height mAU	Relative Area %	Relative Height %	Amount n.a.
1		6.445	70.142	579.732	50.16	51.71	n.a.
2		6.745	69.704	541.306	49.84	48.29	n.a.
**Total:**			**139.846**	**1121.038**	**100.00**	**100.00**	

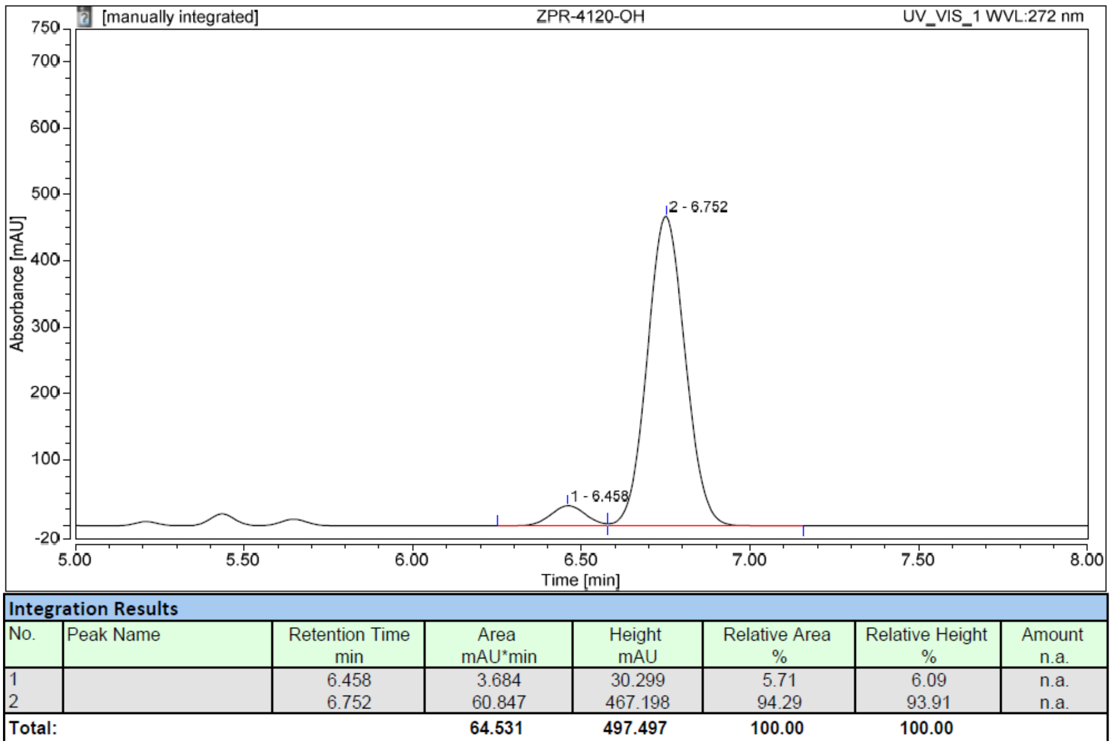

Integration Results							
No.	Peak Name	Retention Time min	Area mAU*min	Height mAU	Relative Area %	Relative Height %	Amount n.a.
1		6.458	3.684	30.299	5.71	6.09	n.a.
2		6.752	60.847	467.198	94.29	93.91	n.a.
Total:			64.531	497.497	100.00	100.00	

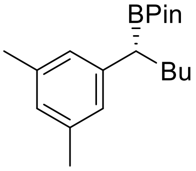

3t

The title compound **3t** was synthesized according to General Procedure B, and it was purified by column chromatography on silica gel (51% yield, 94% *ee*, 30.8 mg, colorless oil)

^{1}H NMR (400 MHz, Chloroform-*d*) δ 6.82 (s, 1H), 6.76 (s, 1H), 2.26 (s, 3H), 2.21 (dd, *J* = 8.0, 8.0 Hz, 3H), 1.87-1.74 (m, 1H), 1.66-1.53 (m, 1H), 1.36-1.22 (m, 4H), 1.21 (s, 6H), 1.19 (s, 6H), 0.86 (t, *J* = 7.0 Hz, 3H); ^{13}C NMR (101 MHz, Chloroform-*d*) δ 143.3, 137.4, 126.8, 126.2, 83.1, 32.5, 31.6, 24.6, 22.7, 21.3 , 14.0; HRMS: m/z (ESI) calculated $[M+H]^+$:303.2490, found: 303.2495. $[\alpha]_D^{25}$ = -15.541 (c = 0.25, $CHCl_3$).

The enantiomeric excess of **3t** was determined by chiral HPLC analysis compared to the corresponding racemate alcohol.

Conditions: ChiralPak IG column; hexane/^{i}PrOH = 99:1; flow rate = 1.0 mL/min; λ= 254 nm; t_{R1}(major)= 28.6 min; t_{R2}(minor)= 22.7 min.

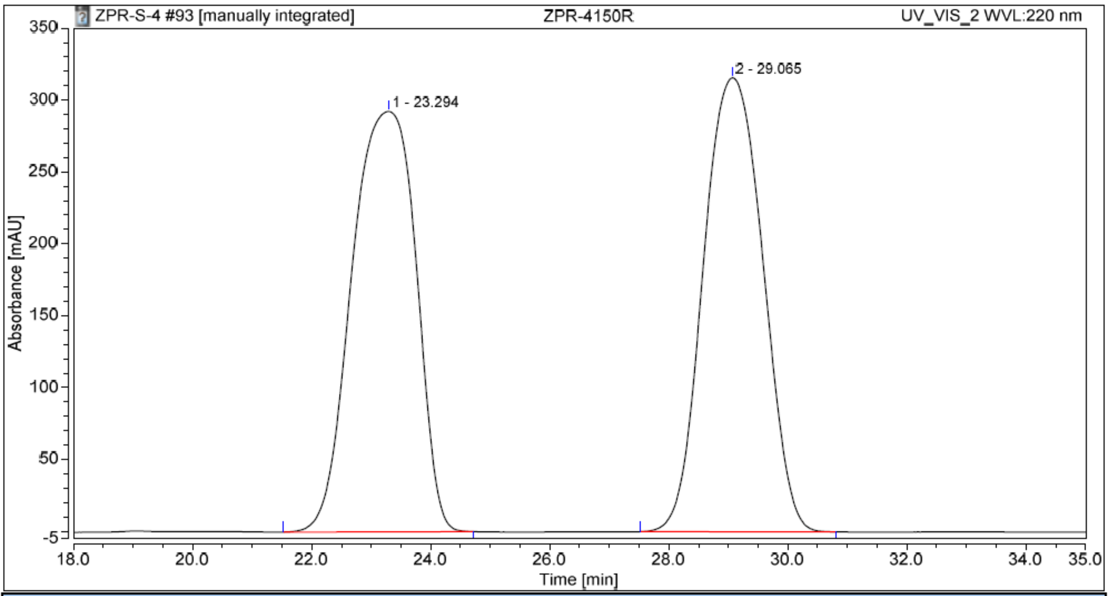

Integration Results

No.	Peak Name	Retention Time min	Area mAU*min	Height mAU	Relative Area %	Relative Height %	Amount n.a.
1		23.294	365.471	292.336	50.02	48.08	n.a.
2		29.065	365.198	315.737	49.98	51.92	n.a.
**Total:**			**730.669**	**608.073**	**100.00**	**100.00**	

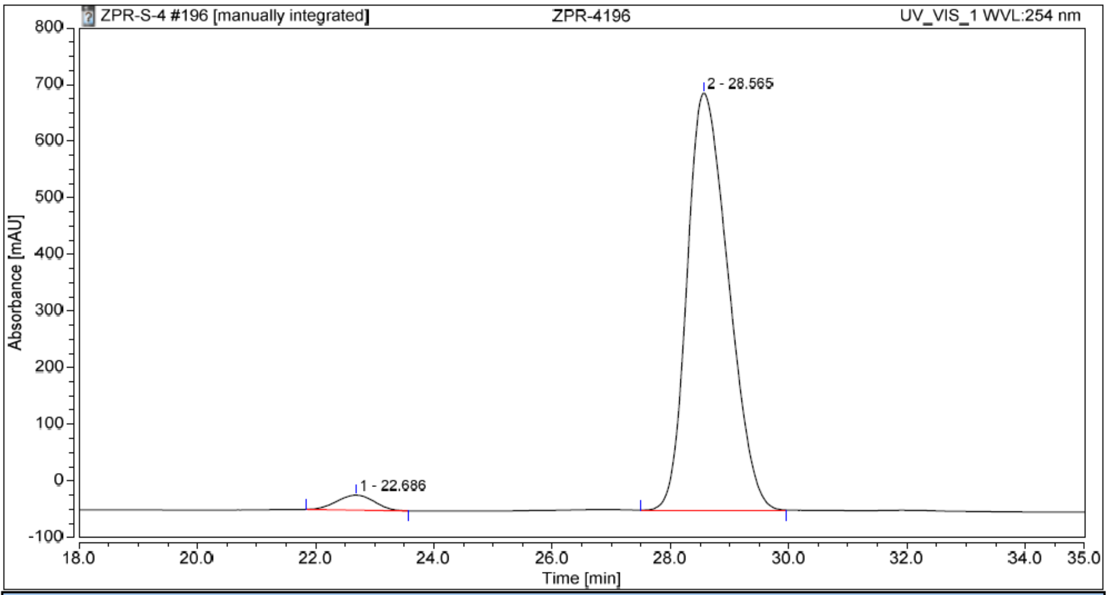

Integration Results

No.	Peak Name	Retention Time min	Area mAU*min	Height mAU	Relative Area %	Relative Height %	Amount n.a.
1		22.686	19.434	26.402	3.17	3.46	n.a.
2		28.565	593.372	737.019	96.83	96.54	n.a.
**Total:**			**612.805**	**763.422**	**100.00**	**100.00**	

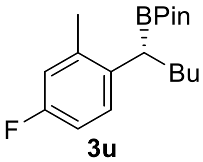

3u

The title compound **3u** was synthesized according to General Procedure B, and it was purified by column chromatography on silica gel (63% yield, 98% *ee*, 38.6 mg, colorless oil).

^{1}H NMR (400 MHz, Chloroform-*d*) δ 7.20-7.11 (m, 1H), 6.87-6.73 (m, 2H), 2.44 (dd, *J* = 7.8, 7.8 Hz, 1H), 2.30 (s, 3H), 1.90-1.76 (m, 1H), 1.64 -1.52 (m, 1H), 1.35-1.21 (m, 4H), 1.20 (s, 6H), 1.18 (s, 6H), 0.86 (t, *J* = 7.0 Hz, 3H); ^{13}C NMR (101 MHz, Chloroform-*d*) δ 160.4 (d, *J* = 241.8 Hz), 138.0 (d, *J* = 7.3 Hz), 137.4 (d, *J* = 3.0 Hz), 128.8 (d, *J* = 8.0 Hz), 116.5 (d, *J* = 20.6 Hz), 112.3 (d, *J* = 20.5 Hz), 83.2, 31.9, 31.6, 24.6, 24.5, 22.8, 20.2 (d, *J* = 1.5 Hz), 14.0; HRMS: m/z (ESI) calculated $[M+NH_4]^+$: 324.2505, found: 324.2511. $[\alpha]_D^{25}$ = -14.868 (*c* = 0.25, $CHCl_3$).

The enantiomeric excess of **3u** was determined by chiral HPLC analysis compared to the corresponding racemate alcohol.

Conditions: ChiralPak IG column; hexane/^{i}PrOH = 98:2; flow rate = 1.0 mL/min; λ= 254 nm; t_{R1}(major)= 11.6 min; t_{R2}(minor)= 13.0 min.

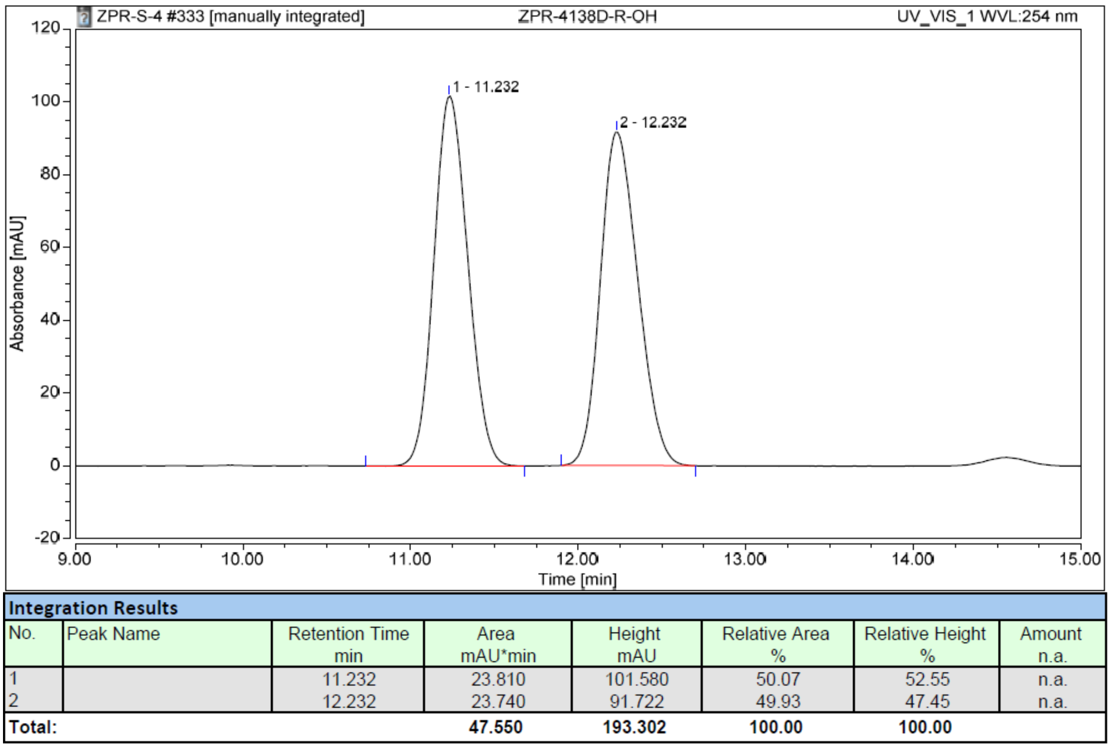

Integration Results							
No.	Peak Name	Retention Time min	Area mAU*min	Height mAU	Relative Area %	Relative Height %	Amount n.a.
1		11.232	23.810	101.580	50.07	52.55	n.a.
2		12.232	23.740	91.722	49.93	47.45	n.a.
**Total:**			**47.550**	**193.302**	**100.00**	**100.00**	

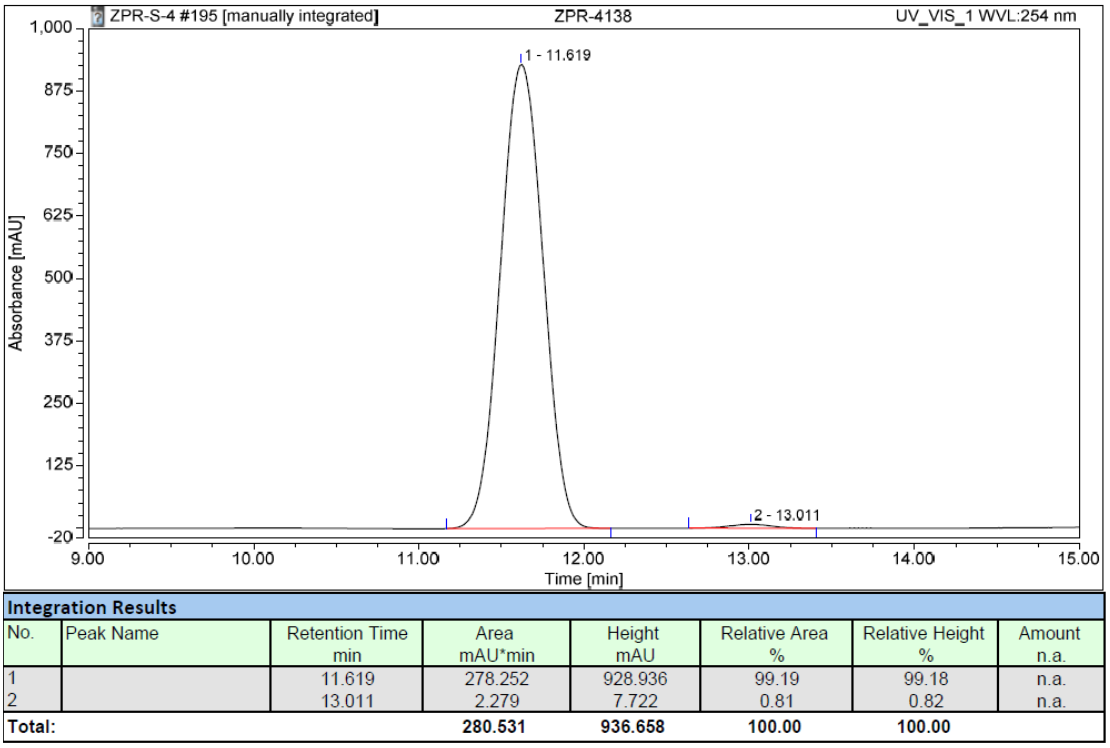

Integration Results							
No.	Peak Name	Retention Time min	Area mAU*min	Height mAU	Relative Area %	Relative Height %	Amount n.a.
1		11.619	278.252	928.936	99.19	99.18	n.a.
2		13.011	2.279	7.722	0.81	0.82	n.a.
**Total:**			**280.531**	**936.658**	**100.00**	**100.00**	

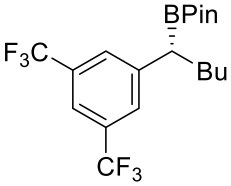

3v

The title compound **3v** was synthesized according to General Procedure B, and it was purified by column chromatography on silica gel (86% yield, 94% *ee*, 70.5 mg, colorless oil)

^{1}H NMR (400 MHz, Chloroform-*d*) δ 7.74-7.60 (m, 3H), 2.44 (dd, *J* = 8.0, 8.0 Hz, 1H), 1.95-1.83 (m, 1H), 1.72-1.61 (m, 1H), 1.39 -1.24 (m, 4H), 1.21 (s, 12H), 0.88 (t, *J* = 7.1 Hz, 3H); ^{13}C NMR (101 MHz, Chloroform-*d*) δ 146.3 , 131.3 (q, *J* = 32.9 Hz), 128.4 (q, *J* = 2.6 Hz), 123.6 (q, *J* = 272.5 Hz), 119.2 (p, *J* = 3.9 Hz), 83.8, 32.2, 31.4, 24.6, 24.5, 22.6, 13.9; HRMS: m/z (ESI) calculated $[M+Na]^+$:433.1744, found: 433.1747. $[\alpha]_D^{25}$ = -2.772 (*c* = 0.25, $CHCl_3$).

The enantiomeric excess of **3v** was determined by chiral HPLC analysis compared to the corresponding racemate alcohol.

Conditions: ChiralPak IB column; hexane/^{i}PrOH = 98:2; flow rate = 1.0 mL/min; λ= 220 nm; t_{R1}(major)= 6.6 min; t_{R2}(minor)= 6.3 min.

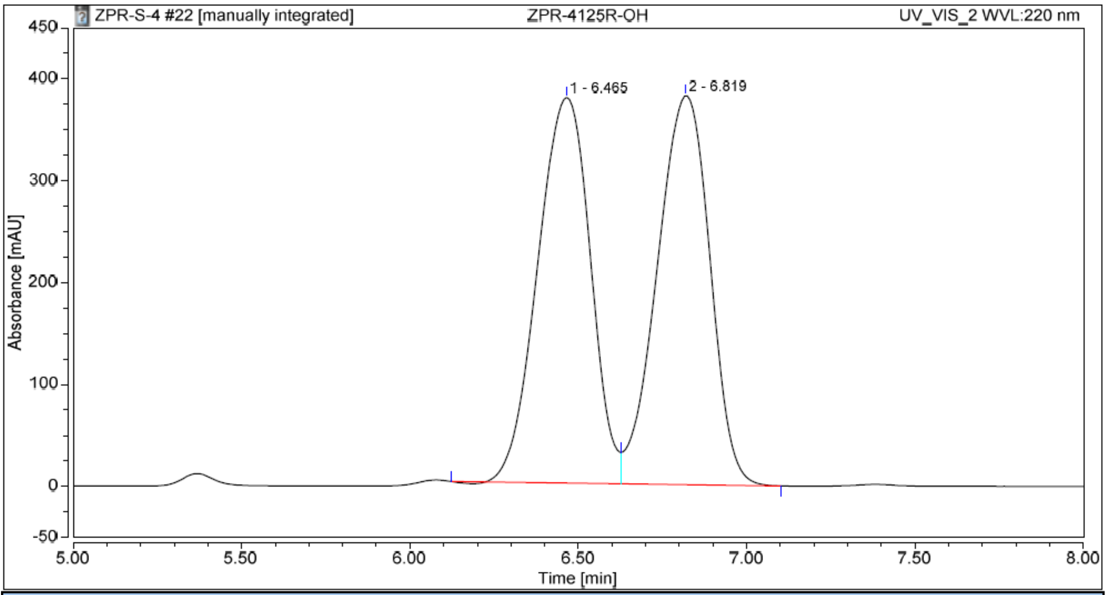

Integration Results							
No.	Peak Name	Retention Time min	Area mAU*min	Height mAU	Relative Area %	Relative Height %	Amount n.a.
1		6.465	68.632	378.642	49.76	49.78	n.a.
2		6.819	69.286	381.985	50.24	50.22	n.a.
**Total:**			**137.918**	**760.627**	**100.00**	**100.00**	

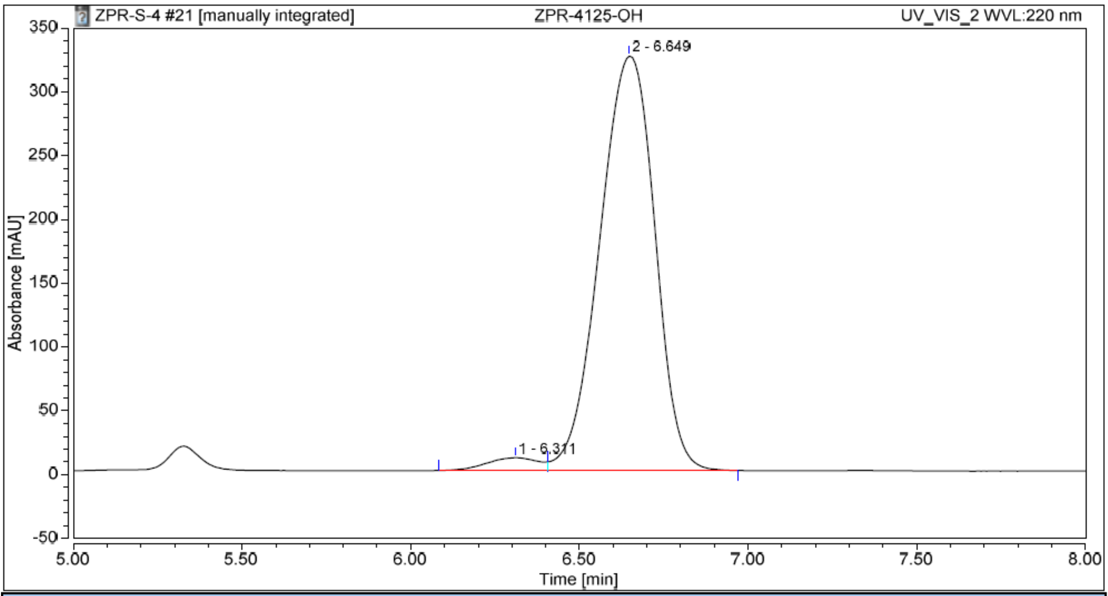

Integration Results							
No.	Peak Name	Retention Time min	Area mAU*min	Height mAU	Relative Area %	Relative Height %	Amount n.a.
1		6.311	1.774	9.939	2.80	2.97	n.a.
2		6.649	61.574	324.888	97.20	97.03	n.a.
**Total:**			**63.348**	**334.827**	**100.00**	**100.00**	

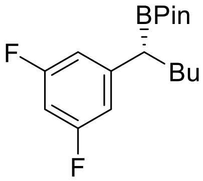

3w

The title compound **3w** was synthesized according to General Procedure B, and it was

purified by column chromatography on silica gel (59% yield, 90% *ee*, 36.6 mg, colorless oil)

^{1}H NMR (400 MHz, Chloroform-*d*) δ 6.78-6.67 (m, 2H), 6.62-6.52 (m, 2H), 2.29 (dd, *J* = 7.8, 8.0 Hz, 1H), 1.87-1.75 (m, 1H), 1.69-1.53 (m, 1H), 1.37-1.23 (m, 4H), 1.21 (s, 6H), 1.20 (s, 6H), 0.86 (t, *J* = 7.0 Hz, 3H); ^{13}C NMR (101 MHz, Chloroform-*d*) δ 162.9 (dd, *J* = 246.8, 13.2 Hz), 147.7 (dd, *J* = 9.0, 9.0 Hz), 110.8 (dd, *J* = 24.4, 6.4 Hz), 100.5 (dd, *J* = 25.4, 25.4 Hz), 83.5, 31.8, 31.3, 24.6, 24.5, 22.6, 14.0; HRMS: m/z (ESI) calculated $[M+Na]^+$:333.1813, found: 333.1829. $[\alpha]_D^{25}$ = -15.959 (*c* = 0.25, $CHCl_3$).

The enantiomeric excess of **3w** was determined by chiral HPLC analysis compared to the corresponding racemate.

Conditions: ChiralPak IG column; hexane/^{i}PrOH = 99:1; flow rate = 1.0 mL/min; λ= 220 nm; t_{R1}(major)= 21.8 min; t_{R2}(minor)= 20.3 min.

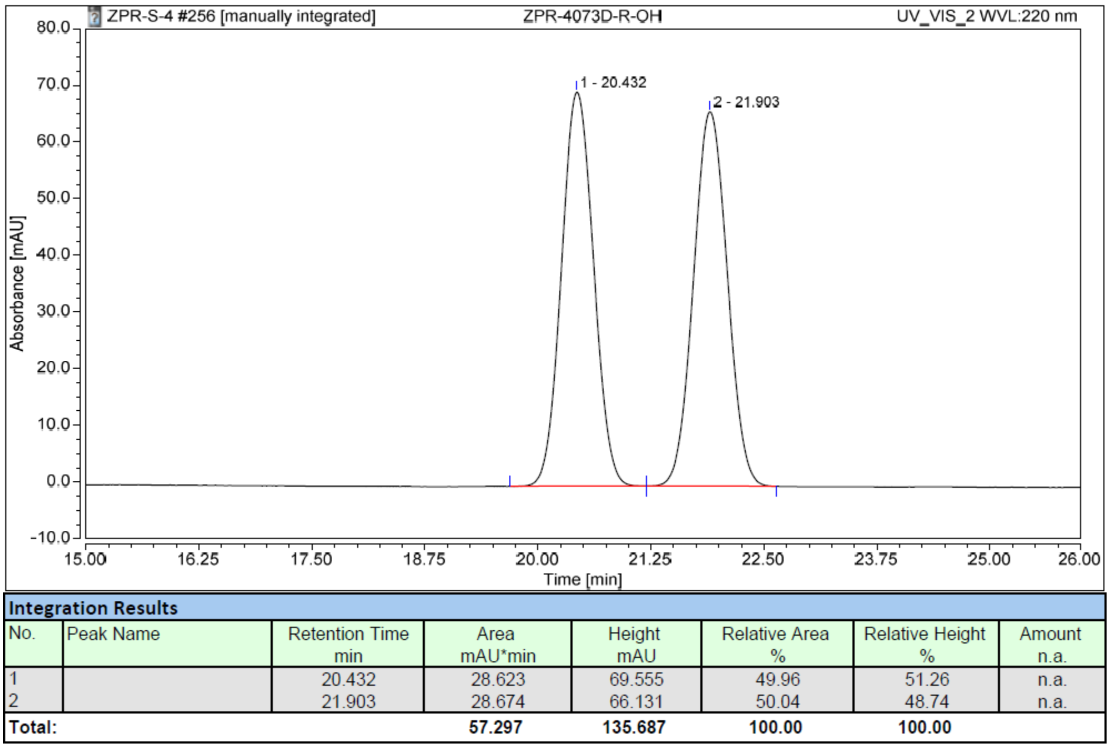

Integration Results

No.	Peak Name	Retention Time min	Area mAU*min	Height mAU	Relative Area %	Relative Height %	Amount n.a.
1		20.432	28.623	69.555	49.96	51.26	n.a.
2		21.903	28.674	66.131	50.04	48.74	n.a.
**Total:**			**57.297**	**135.687**	**100.00**	**100.00**	

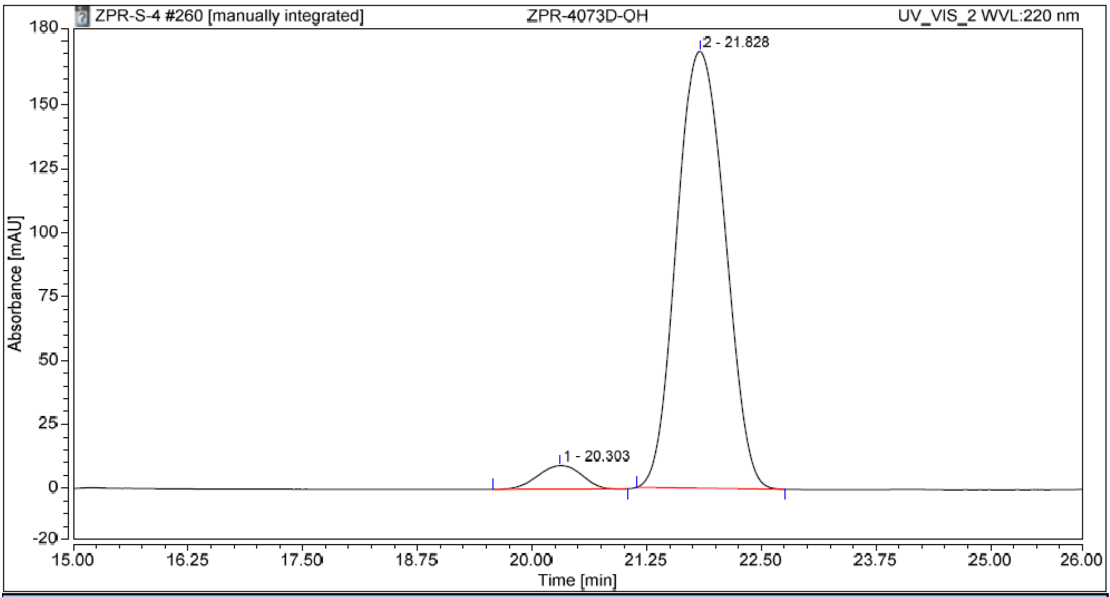

Integration Results							
No.	Peak Name	Retention Time min	Area mAU*min	Height mAU	Relative Area %	Relative Height %	Amount n.a.
1		20.303	5.051	9.204	4.62	5.11	n.a.
2		21.828	104.171	171.002	95.38	94.89	n.a.
Total:			109.221	180.206	100.00	100.00	

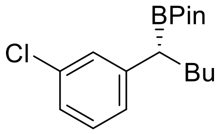

3x

The title compound **3x** was synthesized according to General Procedure B, and it was purified by column chromatography on silica gel (70% yield, 93% *ee*, 43.1 mg, colorless oil).

^{1}H NMR (600 MHz, Chloroform-*d*) δ 7.20 (s, 1H), 7.18-7.14 (m, 1H), 7.12-7.05 (m, 2H), 2.30-2.21 (m, 1H), 1.87-1.77 (m, 1H), 1.64-1.57 (m, 1H), 1.34-1.25 (m, 4H), 1.21 (s, 6H), 1.19 (s, 6H), 0.86 (t, J = 7.4 Hz, 3H); ^{13}C NMR (151 MHz, Chloroform-*d*) δ 145.6, 133.9, 129.4, 128.4, 126.5, 125.2, 83.4, 32.0, 31.4, 24.6, 24.5, 22.6, 14.0; HRMS: m/z (ESI) calculated $[M+Na]^+$:333.1607, found: 331.1615. $[\alpha]_D^{25}$ = -9.298 (c = 0.25, $CHCl_3$).

The enantiomeric excess of **3x** was determined by chiral HPLC analysis compared to the corresponding racemate.

Conditions: ChiralPak IC column; hexane/^{i}PrOH = 98:2; flow rate = 1.0 mL/min; λ= 220 nm; t_{R1}(major)= 20.8 min; t_{R2}(minor)= 22.1 min.

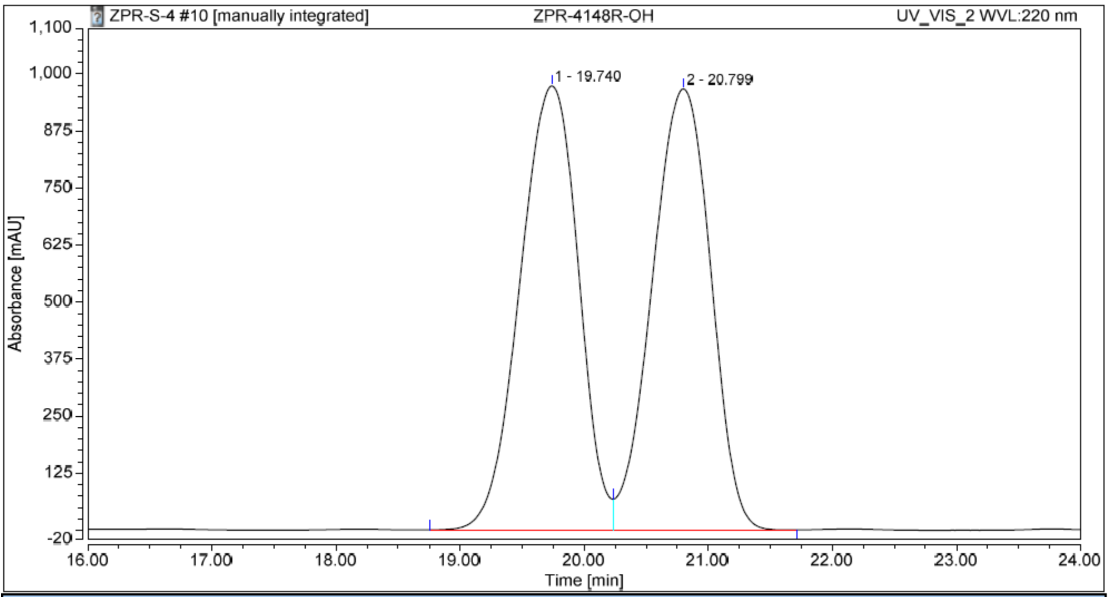

Integration Results

No.	Peak Name	Retention Time min	Area mAU*min	Height mAU	Relative Area %	Relative Height %	Amount n.a.
1		19.740	530.622	973.140	50.13	50.17	n.a.
2		20.799	527.963	966.673	49.87	49.83	n.a.
**Total:**			**1058.585**	**1939.814**	**100.00**	**100.00**	

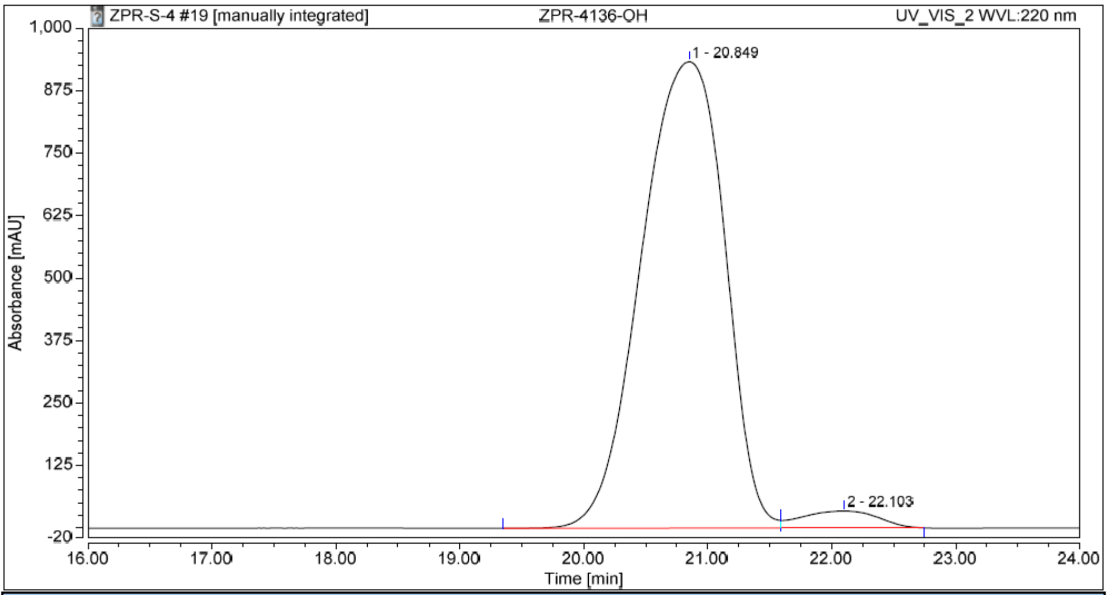

Integration Results

No.	Peak Name	Retention Time min	Area mAU*min	Height mAU	Relative Area %	Relative Height %	Amount n.a.
1		20.849	726.561	933.266	96.79	96.49	n.a.
2		22.103	24.120	33.942	3.21	3.51	n.a.
**Total:**			**750.680**	**967.208**	**100.00**	**100.00**	

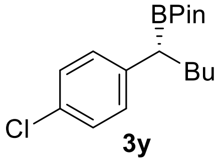

3y

The title compound **3y** was synthesized according to General Procedure B, and it was purified by column chromatography on silica gel (76% yield, 92% *ee*, 46.8 mg, colorless oil).

^{1}H NMR (600 MHz, Chloroform-*d*) δ 7.21 (d, *J* = 8.5 Hz, 2H), 7.13 (d, *J* = 8.5 Hz, 2H), 2.26 (dd, *J* = 8.0, 8.0 Hz, 1H), 1.86-1.78 (m, 1H), 1.63-1.57 (m, 1H), 1.39-1.24 (m, 4H), 1.20 (s, 6H), 1.18 (s, 6H), 0.85 (t, *J* = 7.2 Hz, 3H); ^{13}C NMR (151 MHz, Chloroform-*d*) δ 142.0, 130.7, 129.6, 128.3, 83.3, 32.1, 31.4, 24.6, 24.5, 22.6, 14.0; HRMS: m/z (ESI) calculated $[M+Na]^+$: 331.1607, found: 331.1616. $[\alpha]_D^{25}$ = -16.256 (*c* = 0.25, $CHCl_3$).
The enantiomeric excess of **3y** was determined by chiral HPLC analysis compared to the corresponding racemate alcohol.
Conditions: ChiralPak IG column; hexane/^{i}PrOH = 98:2; flow rate = 1.0 mL/min; λ= 220 nm; t_{R1}(major)= 16.1 min; t_{R2}(minor)= 14.6 min.

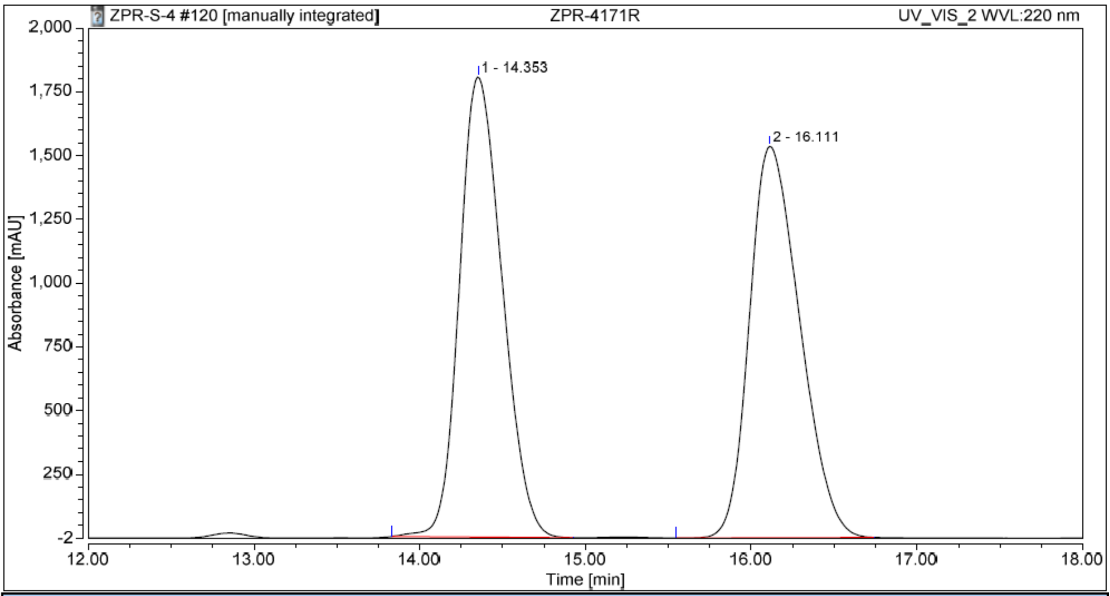

Integration Results

No.	Peak Name	Retention Time min	Area mAU*min	Height mAU	Relative Area %	Relative Height %	Amount n.a.
1		14.353	539.355	1804.908	49.98	54.04	n.a.
2		16.111	539.811	1535.074	50.02	45.96	n.a.
**Total:**			**1079.166**	**3339.982**	**100.00**	**100.00**	

Chromatogram

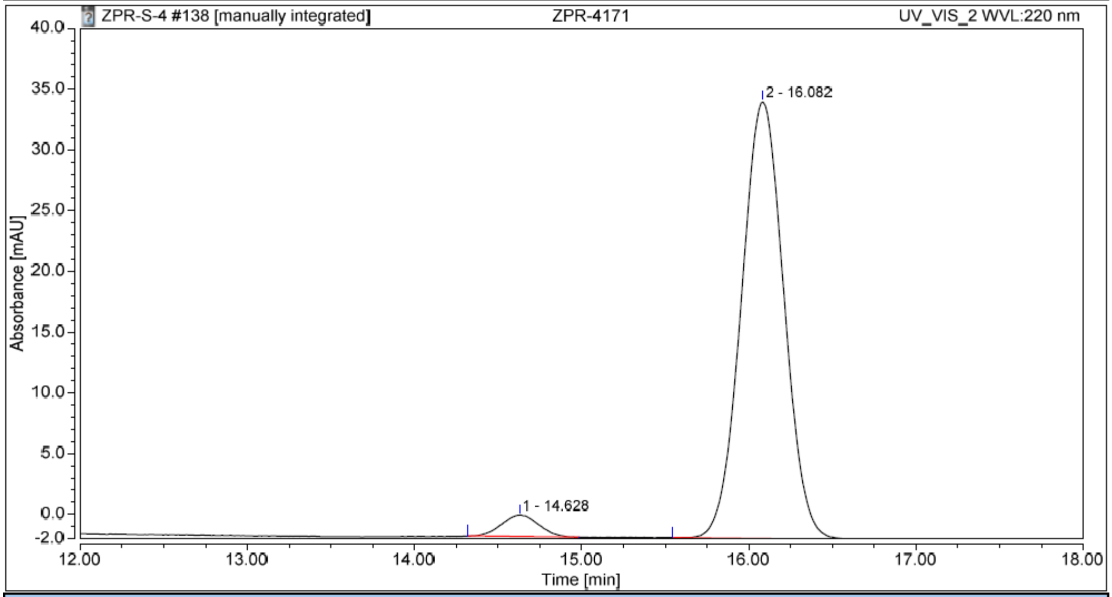

Integration Results

No.	Peak Name	Retention Time min	Area mAU*min	Height mAU	Relative Area %	Relative Height %	Amount n.a.
1		14.628	0.450	1.770	4.08	4.70	n.a.
2		16.082	10.577	35.937	95.92	95.30	n.a.
**Total:**			**11.027**	**37.707**	**100.00**	**100.00**	

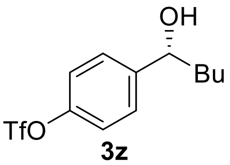

3z

The title compound **3z** was synthesized according to General Procedure B, and it was purified by column chromatography on silica gel (80% yield, 93% *ee*, 49.9 mg, colorless oil).

^{1}H NMR (400 MHz, Chloroform-*d*) δ 7.43 (d, J = 8.8 Hz, 2H), 7.25 (d, J = 8.8 Hz, 2H), 4.71 (dd, J = 7.6, 7.6 Hz, 1H), 1.96 (br, 1H), 1.83-1.64 (m, 2H), 1.44-1.28 (m, 4H), 0.90 (t, J = 7.0 Hz, 3H); ^{13}C NMR (101 MHz, Chloroform-*d*) δ 148.6, 145.4, 127.7, 121.2, 73.7, 39.0, 27.8, 22.5, 13.9; HRMS: m/z (ESI) calculated $[M+Na]^+$: 335.0535, found: 335.0538. $[\alpha]_D^{25}$ = +18.386 (c = 0.25, $CHCl_3$).
The enantiomeric excess of **3z** was determined by chiral HPLC analysis compared to the corresponding racemate alcohol.
Conditions: ChiralPak IG column; hexane/^{i}PrOH = 98:2; flow rate = 1.0 mL/min; λ= 220 nm; t_{R1}(major)= 15.2 min; t_{R2}(minor)= 13.9 min.

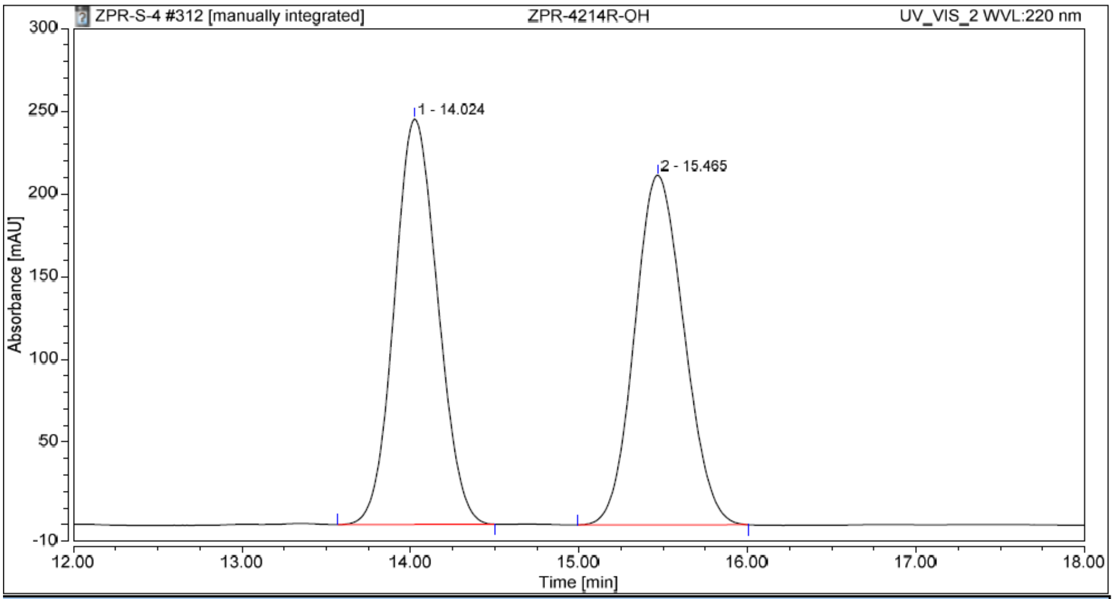

Integration Results

No.	Peak Name	Retention Time min	Area mAU*min	Height mAU	Relative Area %	Relative Height %	Amount n.a.
1		14.024	73.215	245.252	50.46	53.70	n.a.
2		15.465	71.881	211.454	49.54	46.30	n.a.
**Total:**			**145.096**	**456.706**	**100.00**	**100.00**	

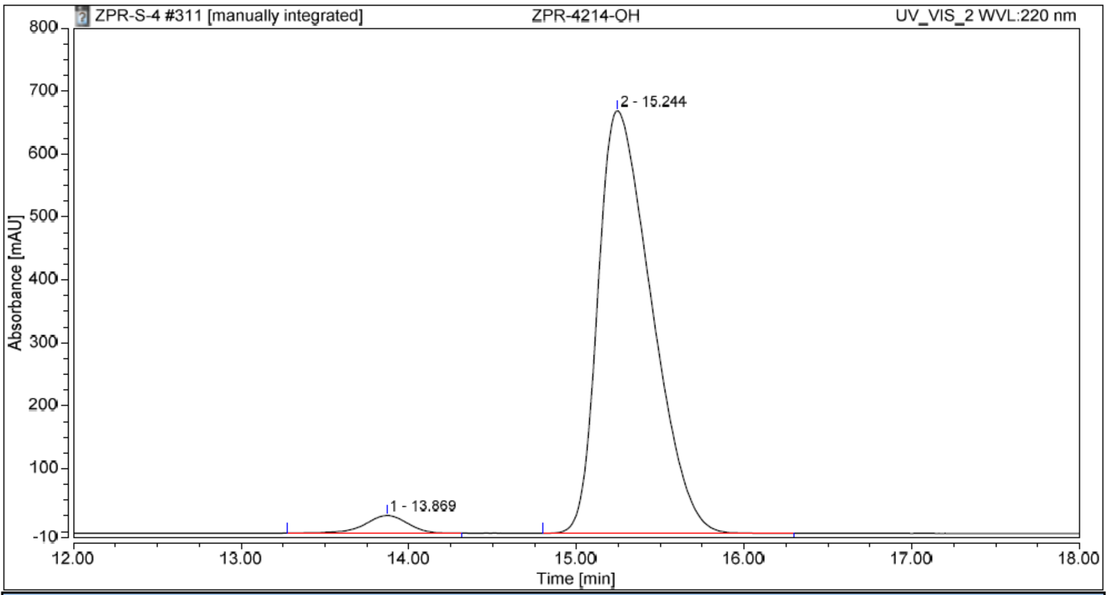

Integration Results

No.	Peak Name	Retention Time min	Area mAU*min	Height mAU	Relative Area %	Relative Height %	Amount n.a.
1		13.869	8.418	28.180	3.29	4.03	n.a.
2		15.244	247.110	671.777	96.71	95.97	n.a.
**Total:**			**255.528**	**699.957**	**100.00**	**100.00**	

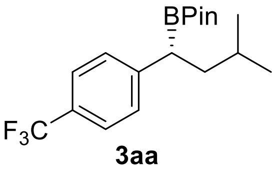

3aa

The title compound **3aa** was synthesized according to General Procedure B, and it was purified by column chromatography on silica gel (69% yield, 95% *ee*, 47.2 mg,

colorless oil)

^{1}H NMR (600 MHz, Chloroform-*d*) δ 7.49 (d, *J* = 8.0 Hz, 2H), 7.31 (d, *J* = 8.0 Hz, 2H), 2.50 (dd, *J* = 8.0, 8.0 Hz, 1H), 1.73-1.67 (m, 1H), 1.65-1.59 (m, 1H), 1.47-1.41 (m, 1H), 1.19 (s, 6H), 1.18 (s, 6H), 0.89 (d, *J* = 6.6 Hz, 3H), 0.87 (d, *J* = 6.8 Hz, 3H); ^{13}C NMR (151 MHz, Chloroform-*d*) δ 147.9, 128.5, 127.3 (q, *J* = 32.2 Hz), 125.1 (q, *J* = 3.6 Hz), 124.5 (d, *J* = 271.6 Hz), 83.5, 41.1, 26.8, 24.5, 24.5, 22.9, 22.0; HRMS: m/z (ESI) calculated $[M+Na]^+$:365.1870, found: 365.1875. $[\alpha]_D^{25}$ = -13.497 (*c* = 0.25, $CHCl_3$).

The enantiomeric excess of **3aa** was determined by chiral HPLC analysis compared to the corresponding racemate alcohol.

Conditions: ChiralPak IG column; hexane/^{i}PrOH = 99:1; flow rate = 1.0 mL/min; λ= 220 nm; t_{R1}(major)= 19.3 min; t_{R2}(minor)= 17.5 min.

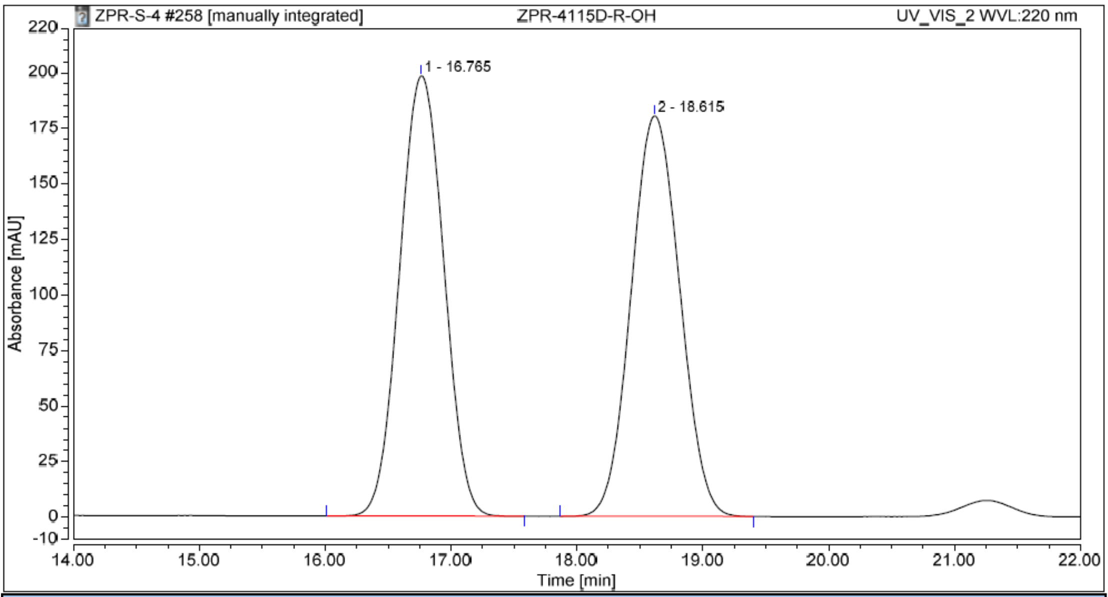

Integration Results							
No.	Peak Name	Retention Time min	Area mAU*min	Height mAU	Relative Area %	Relative Height %	Amount n.a.
1		16.765	80.655	198.139	50.00	52.37	n.a.
2		18.615	80.668	180.178	50.00	47.63	n.a.
**Total:**			**161.323**	**378.318**	**100.00**	**100.00**	

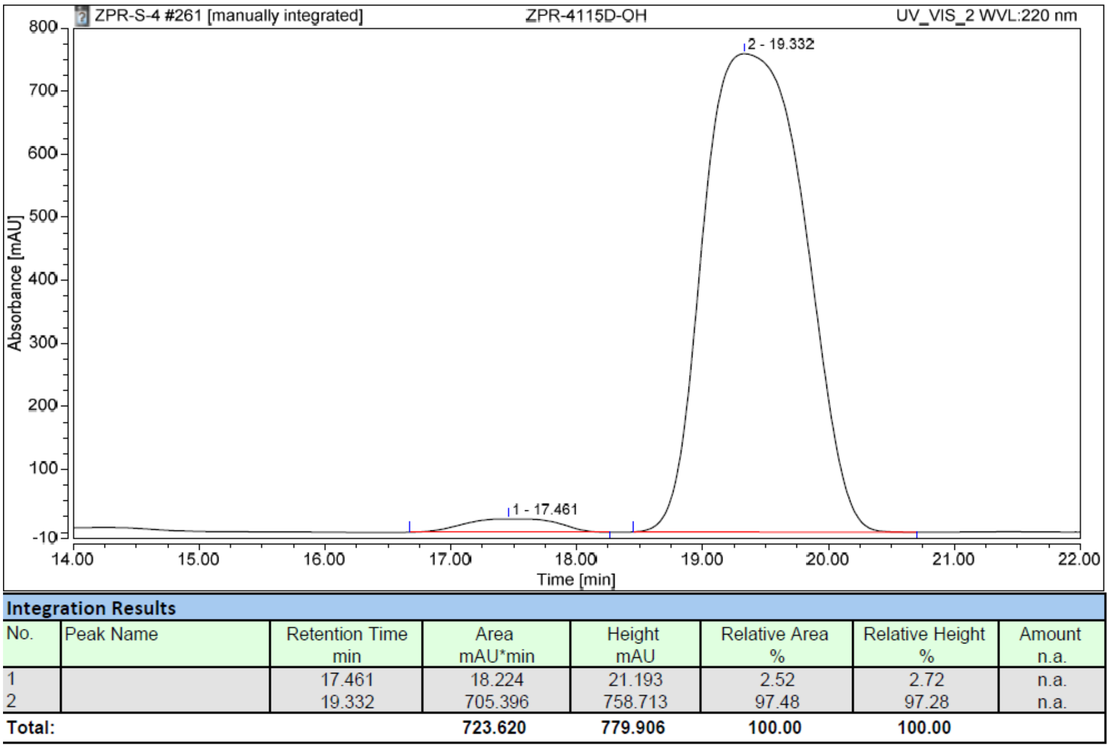

Integration Results

No.	Peak Name	Retention Time min	Area mAU*min	Height mAU	Relative Area %	Relative Height %	Amount n.a.
1		17.461	18.224	21.193	2.52	2.72	n.a.
2		19.332	705.396	758.713	97.48	97.28	n.a.
**Total:**			**723.620**	**779.906**	**100.00**	**100.00**	

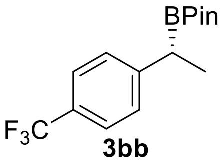

3bb

The title compound **3bb** was synthesized according to General Procedure B, and it was purified by column chromatography on silica gel (71% yield, 90% *ee*, 42.6 mg, colorless oil).

^{1}H NMR (400 MHz, Chloroform-d) δ 7.51 (d, *J* = 8.0 Hz, 2H), 7.32 (d, *J* = 8.0 Hz, 2H), 2.50 (q, *J* = 7.5 Hz, 1H), 1.35 (d, *J* = 7.5 Hz, 3H), 1.21 (s, 6H), 1.20 (s, 6H); ^{13}C NMR (101 MHz, Chloroform-*d*) δ 149.3 , 128.0 , 127.4 (q, *J* = 32.1 Hz), 125.2 (q, *J* = 3.6 Hz), 124.5 (q, *J* = 271.5, 270.8 Hz), 83.5, 24.6, 24.6, 16.7; HRMS: m/z (ESI) calculated $[M+Na]^+$: 323.1401, found: 323.1441. $[\alpha]_D^{25}$ = -2.025 (*c* = 0.25, $CHCl_3$). The enantiomeric excess of **3bb** was determined by chiral HPLC analysis compared to the corresponding racemate alcohol.
Conditions: ChiralPak IG column; hexane/^{i}PrOH = 98:2; flow rate = 1.0 mL/min; λ= 220 nm; t_{R1}(major)= 11.0 min; t_{R2}(minor)= 11.6 min.

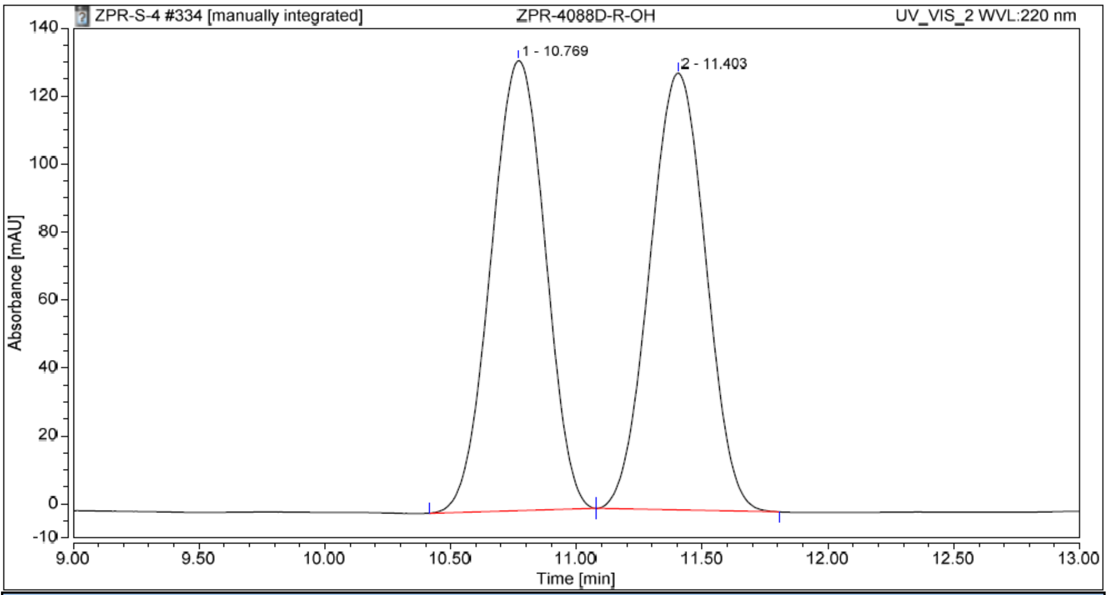

Integration Results

No.	Peak Name	Retention Time min	Area mAU*min	Height mAU	Relative Area %	Relative Height %	Amount n.a.
1		10.769	33.150	132.463	49.97	50.75	n.a.
2		11.403	33.188	128.571	50.03	49.25	n.a.
**Total:**			**66.338**	**261.033**	**100.00**	**100.00**	

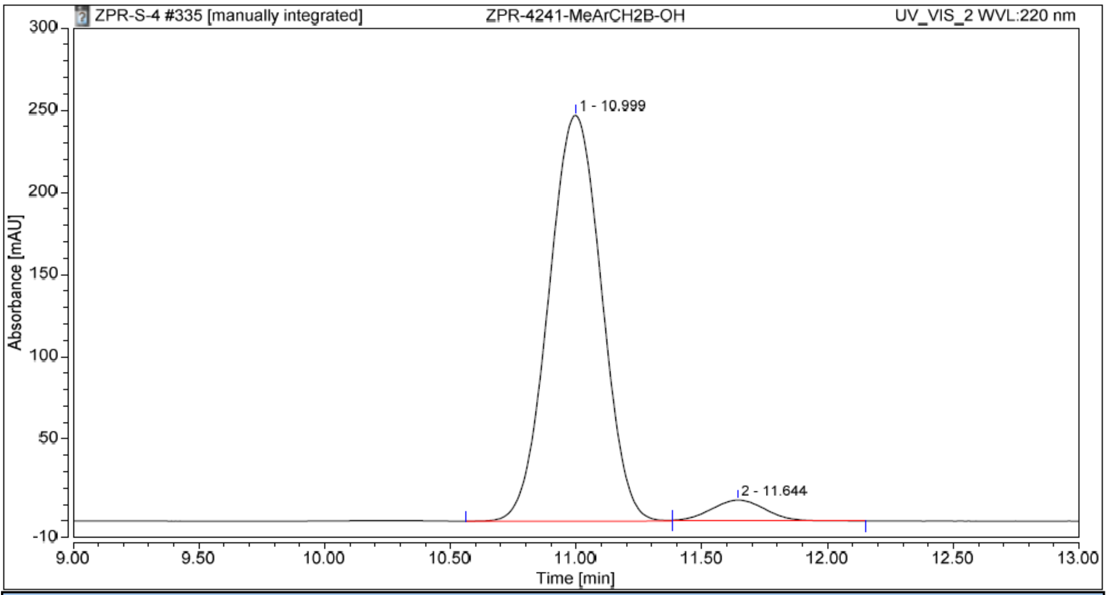

Integration Results

No.	Peak Name	Retention Time min	Area mAU*min	Height mAU	Relative Area %	Relative Height %	Amount n.a.
1		10.999	60.147	247.053	94.89	95.10	n.a.
2		11.644	3.237	12.736	5.11	4.90	n.a.
**Total:**			**63.384**	**259.789**	**100.00**	**100.00**	

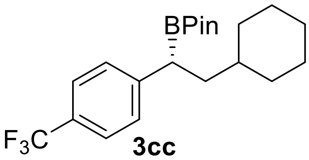

3cc

The title compound **3cc** was synthesized according to General Procedure B, and it was purified by column chromatography on silica gel (77% yield, 95% *ee*, 58.8 mg, colorless oil).

^{1}H NMR (400 MHz, Chloroform-*d*) δ 7.49 (d, *J* = 8.1 Hz, 1H), 7.31 (d, *J* = 8.0 Hz, 1H), 2.53 (t, *J* = 8.1 Hz, 0H), 1.83-1.54 (m, 7H), 1.19 (s, 3H), 1.18 (s, 3H), 1.12 (d, *J* = 8.1 Hz, 3H), 0.88 (p, *J* = 10.1, 8.7 Hz, 2H); ^{13}C NMR (101 MHz, Chloroform-*d*) δ 148.0, 128.5, 127.3 (q, *J* = 32.0 Hz), 125.1 (q, *J* = 3.7 Hz), 124.5 (q, *J* = 271.6 Hz), 83.5, 39.7, 36.5, 33.6, 32.9, 26.6, 26.3, 26.2, 24.5, 24.5; HRMS: m/z (ESI) calculated $[M+Na]^{+}$: 405.2183, found: 405.2201. $[\alpha]_D^{25}$ =-25.646 (*c* = 0.25, $CHCl_3$).
The enantiomeric excess of **3cc** was determined by chiral HPLC analysis compared to the corresponding racemate alcohol.
Conditions: ChiralPak IC column; hexane/^{i}PrOH = 95:5; flow rate = 1.0 mL/min; λ= 220 nm; t_{R1}(major)= 4.0 min; t_{R2}(minor)= 4.3 min.

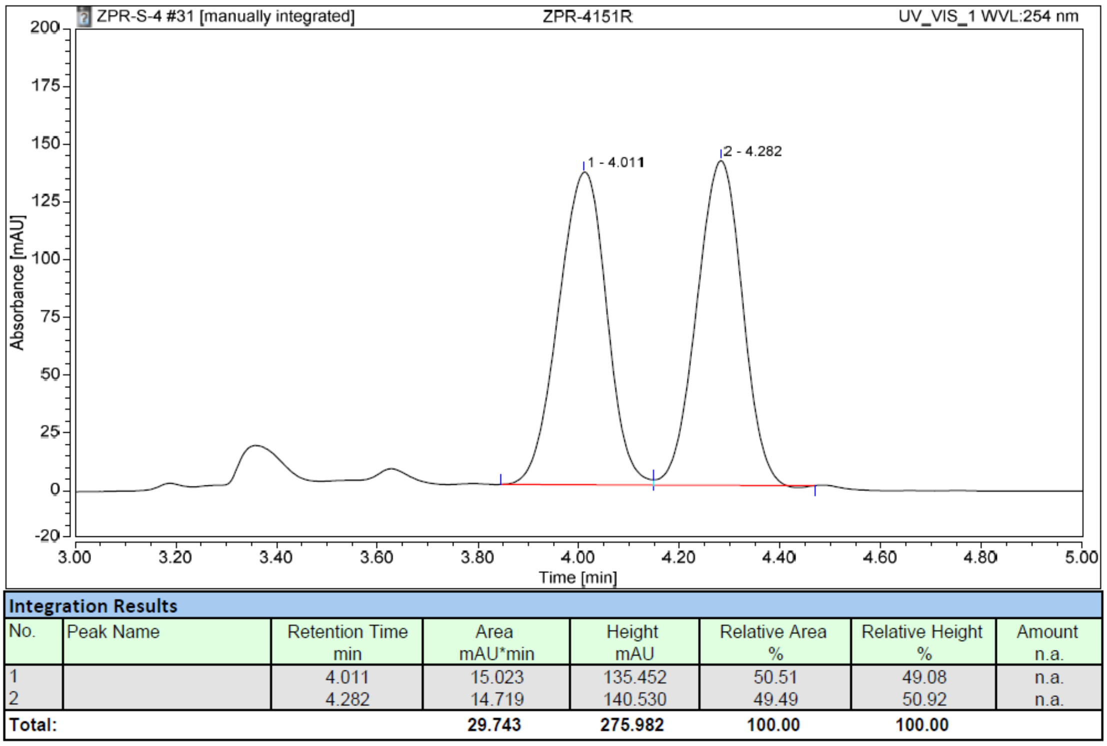

Integration Results

No.	Peak Name	Retention Time min	Area mAU*min	Height mAU	Relative Area %	Relative Height %	Amount n.a.
1		4.011	15.023	135.452	50.51	49.08	n.a.
2		4.282	14.719	140.530	49.49	50.92	n.a.
**Total:**			**29.743**	**275.982**	**100.00**	**100.00**	

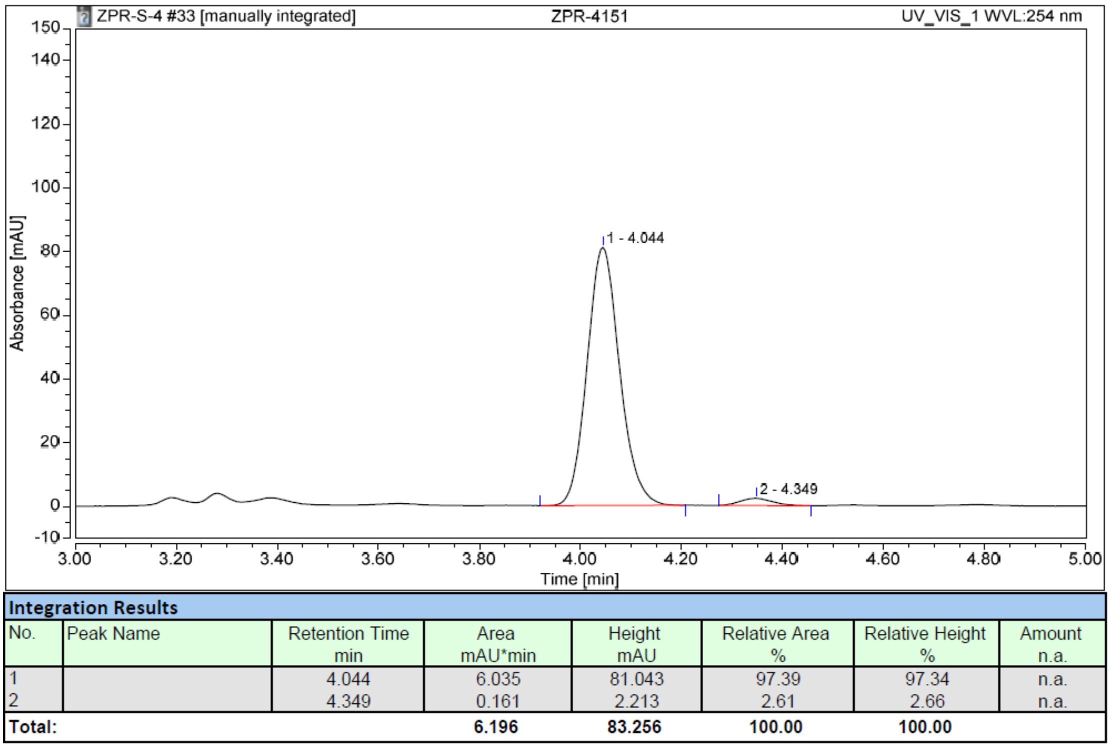

Integration Results

No.	Peak Name	Retention Time min	Area mAU*min	Height mAU	Relative Area %	Relative Height %	Amount n.a.
1		4.044	6.035	81.043	97.39	97.34	n.a.
2		4.349	0.161	2.213	2.61	2.66	n.a.
**Total:**			**6.196**	**83.256**	**100.00**	**100.00**	

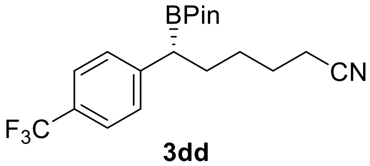

3dd

The title compound **3dd** was synthesized according to General Procedure B, and it was purified by column chromatography on silica gel (53% yield, 92% *ee*, 38.9 mg, colorless oil)

^{1}H NMR (600 MHz, Chloroform-*d*) δ 7.43 (d, *J* = 8.0 Hz, 2H), 7.23 (d, *J* = 8.0 Hz, 2H), 3.46-3.39 (m, 2H), 2.30 (dd, *J* = 7.8, 8.0 Hz, 1H), 1.85-1.77 (m, 1H), 1.72-1.66 (m, 2H), 1.64-1.58 (m, 1H), 1.40-1.26 (m, 2H), 1.13 (s, 6H), 1.11 (s, 6H); ^{13}C NMR (151 MHz, Chloroform-*d*) δ 147.3, 128.5, 127.5 (q, *J* = 32.2 Hz), 125.2 (q, *J* = 3.7 Hz), 124.4 (d, *J* = 271.7 Hz), 83.6, 44.8, 32.5, 31.5, 26.4, 24.8, 24.6, 24.5; HRMS: m/z (ESI) calculated $[M+Na]^+$:390.1823, found: 390.1829. $[\alpha]_D^{25}$ = 17.692 (*c* = 0.25, $CHCl_3$).

The enantiomeric excess of **3dd** was determined by chiral HPLC analysis compared to the corresponding racemate alcohol.

Conditions: ChiralPak IG column; hexane/^{i}PrOH = 98:2; flow rate = 1.0 mL/min; λ= 220 nm; t_{R1}(major)= 9.7 min; t_{R2}(minor)= 11.0 min.

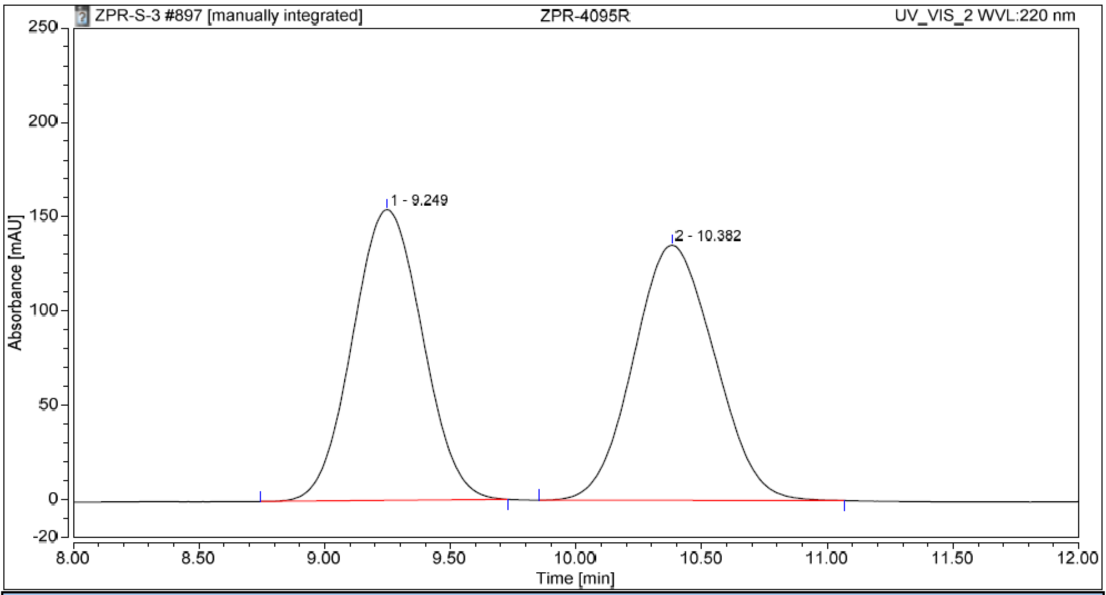

Integration Results							
No.	Peak Name	Retention Time min	Area mAU*min	Height mAU	Relative Area %	Relative Height %	Amount n.a.
1		9.249	49.716	154.061	49.57	53.26	n.a.
2		10.382	50.581	135.214	50.43	46.74	n.a.
**Total:**			**100.297**	**289.275**	**100.00**	**100.00**	

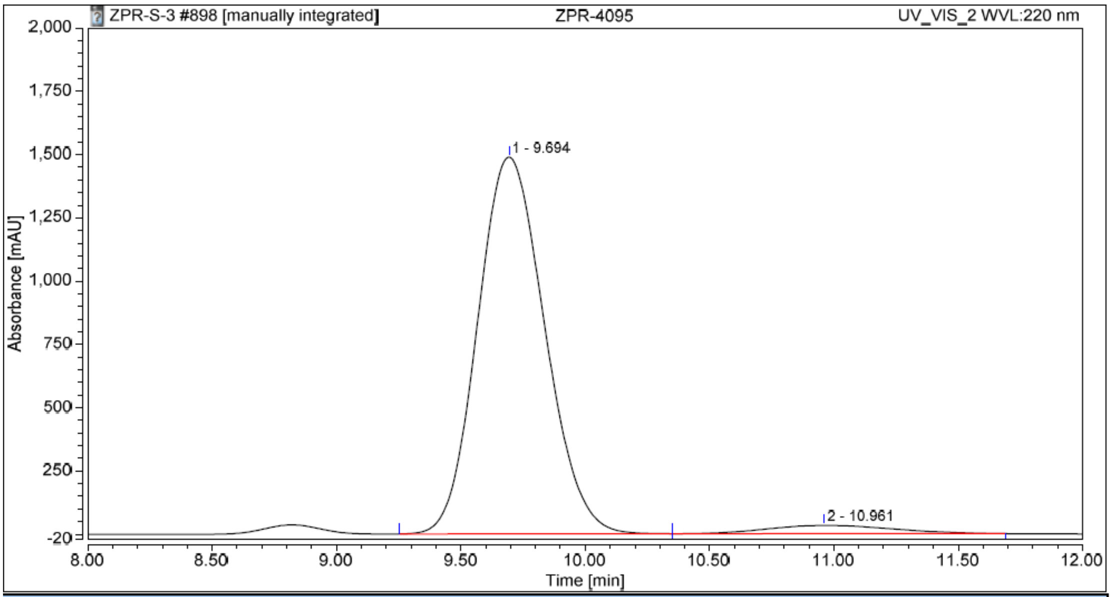

Integration Results							
No.	Peak Name	Retention Time min	Area mAU*min	Height mAU	Relative Area %	Relative Height %	Amount n.a.
1		9.694	463.890	1487.856	96.08	97.85	n.a.
2		10.961	18.941	32.755	3.92	2.15	n.a.
**Total:**			**482.831**	**1520.611**	**100.00**	**100.00**	

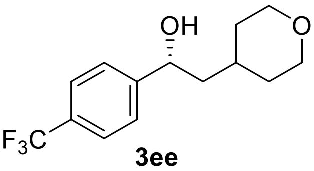

3ee

The title compound **2ee** was synthesized according to General Procedure B, and it was purified by column chromatography on silica gel (74% yield, 93% *ee*, 40.6 mg, colorless oil).

^{1}H NMR (400 MHz, Chloroform-*d*) δ 7.60 (d, *J* = 8.0 Hz, 2H), 7.45 (d, *J* = 8.0 Hz, 2H), 4.84 (t, *J* = 4.3 Hz, 1H), 4.05-3.80 (m, 2H), 3.36 (t, *J* = 11.6 Hz, 2H), 2.37 (br, 1H), 1.82-1.67 (m, 3H), 1.65-1.58 (m, 1H), 1.57-1.48 (m, 1H), 1.37-1.27 (m, 2H); ^{13}C NMR (101 MHz, Chloroform-*d*) δ 149.1, 129.7 (q, *J* = 32.3 Hz), 125.4 (q, *J* = 3.8 Hz), 124.1 (q, *J* = 271.8 Hz), 70.9, 67.9, 67.8, 46.4, 33.5, 32.5, 31.6; HRMS: m/z (ESI) calculated $[M+Na]^+$: 297.1073, found: 297.1070. $[\alpha]_D^{25}$ = +25.926 (*c* = 0.25, $CHCl_3$).
The enantiomeric excess of **3ee** was determined by chiral HPLC analysis compared to the corresponding racemate alcohol.
Conditions: ChiralPak IA column; hexane/^{i}PrOH = 90:10; flow rate = 1.0 mL/min; λ= 220 nm; t_{R1}(major)= 8.5 min; t_{R2}(minor)= 8.0 min.

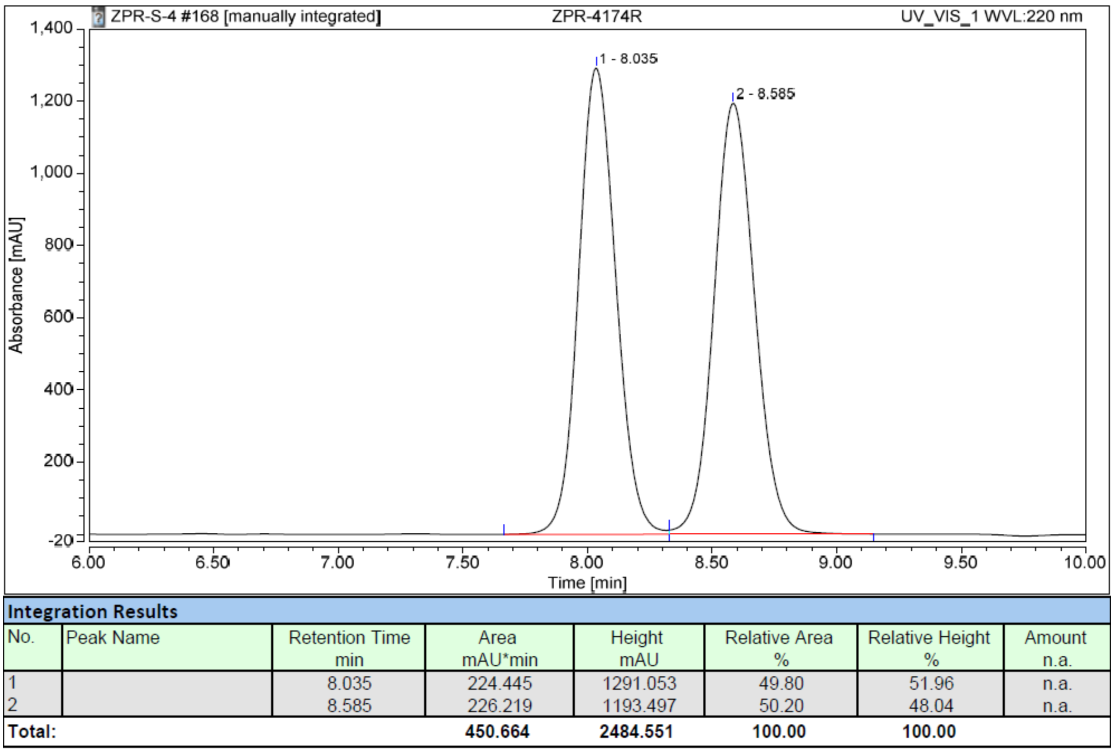

Integration Results

No.	Peak Name	Retention Time min	Area mAU*min	Height mAU	Relative Area %	Relative Height %	Amount n.a.
1		8.035	224.445	1291.053	49.80	51.96	n.a.
2		8.585	226.219	1193.497	50.20	48.04	n.a.
**Total:**			**450.664**	**2484.551**	**100.00**	**100.00**	

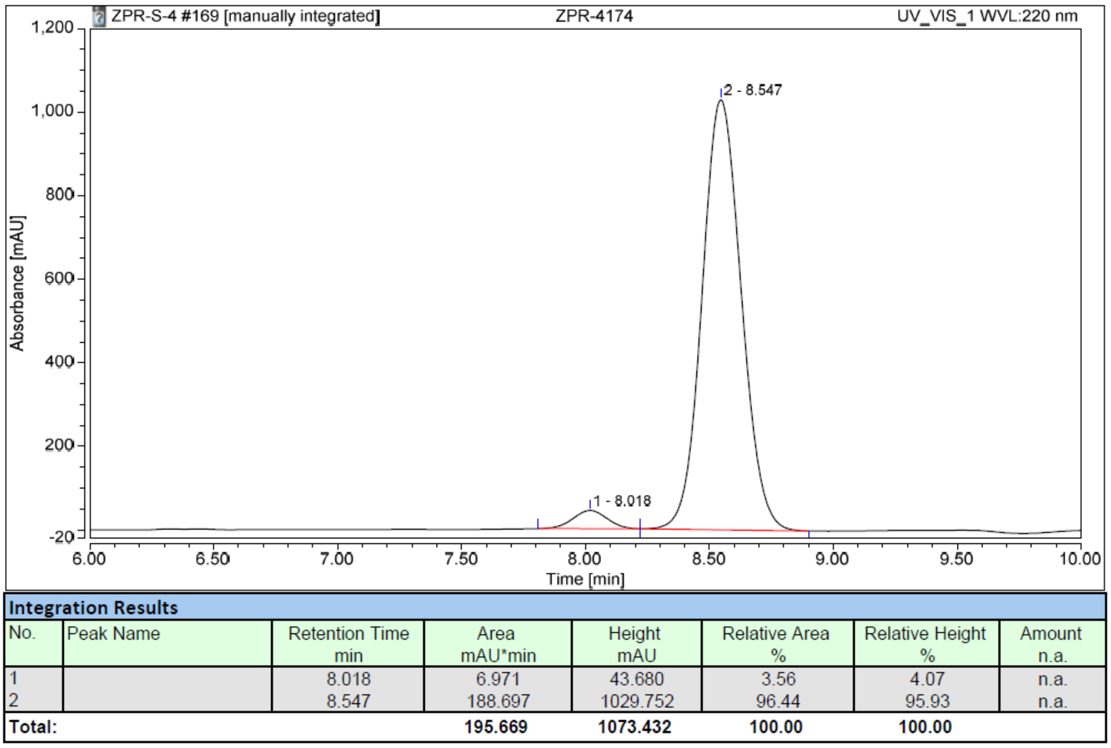

Integration Results

No.	Peak Name	Retention Time min	Area mAU*min	Height mAU	Relative Area %	Relative Height %	Amount n.a.
1		8.018	6.971	43.680	3.56	4.07	n.a.
2		8.547	188.697	1029.752	96.44	95.93	n.a.
**Total:**			**195.669**	**1073.432**	**100.00**	**100.00**	

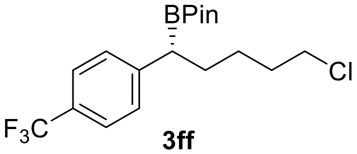

3ff

The title compound **3ff** was synthesized according to General Procedure B, and it was purified by column chromatography on silica gel (84% yield, 94% *ee*, 63.2 mg, colorless oil)

^{1}H NMR (600 MHz, Chloroform-*d*) δ 7.51 (d, *J* = 8.0 Hz, 2H), 7.30 (d, *J* = 8.0 Hz, 2H), 2.37 (dd, *J* = 7.8, 7.8 Hz, 1H), 2.33-2.29 (m, 2H), 1.93-1.83 (m, 1H), 1.70-1.63 (m, 3H), 1.46-1.38 (m, 2H), 1.21 (s, 6H), 1.20 (s, 6H); ^{13}C NMR (151 MHz, Chloroform-*d*) δ 147.0, 128.5, 127.6 (q, *J* = 32.4 Hz), 125.2 (q, *J* = 3.6 Hz), 123.5(q, *J* = 271.5 Hz), 119.6, 83.7, 31.4, 28.1, 25.3, 24.6, 24.5, 17.0; HRMS: m/z (ESI) calculated $[M+Na]^+$:399.1480, found: 399.1504. $[\alpha]_D^{25}$ = -8.621 (*c* = 0.25, $CHCl_3$).
The enantiomeric excess of **3ff** was determined by chiral HPLC analysis compared to the corresponding racemate alcohol.
Conditions: ChiralPak IA column; hexane/^{i}PrOH = 95:5; flow rate = 1.0 mL/min; λ= 220 nm; t_{R1}(major)= 10.7 min; t_{R2}(minor)= 11.6 min.

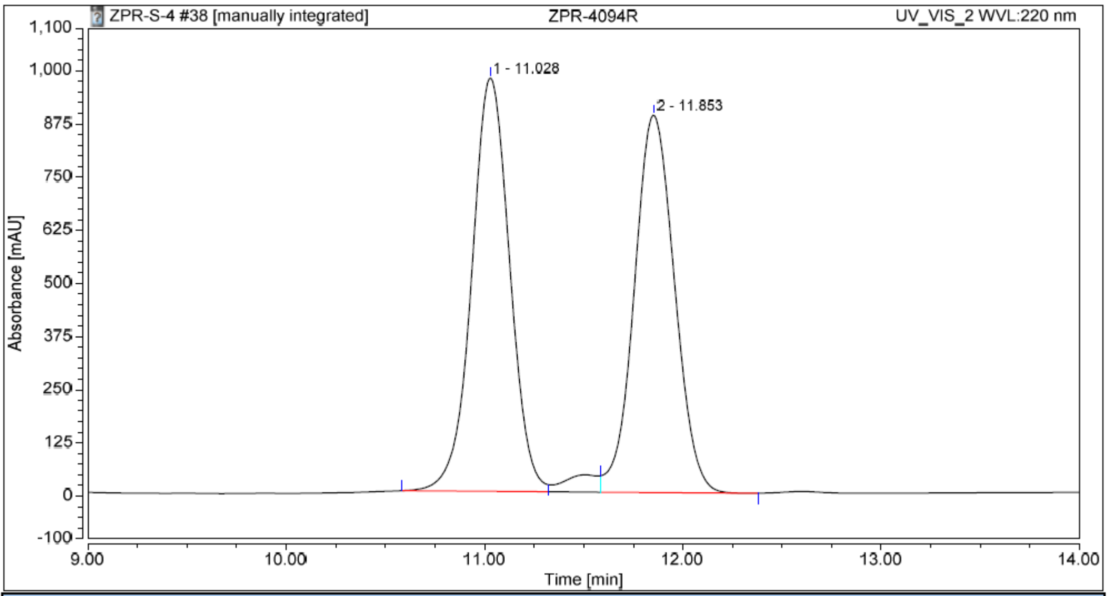

Integration Results							
No.	Peak Name	Retention Time min	Area mAU*min	Height mAU	Relative Area %	Relative Height %	Amount n.a.
1		11.028	216.099	972.453	50.88	52.28	n.a.
2		11.853	208.600	887.514	49.12	47.72	n.a.
**Total:**			**424.699**	**1859.966**	**100.00**	**100.00**	

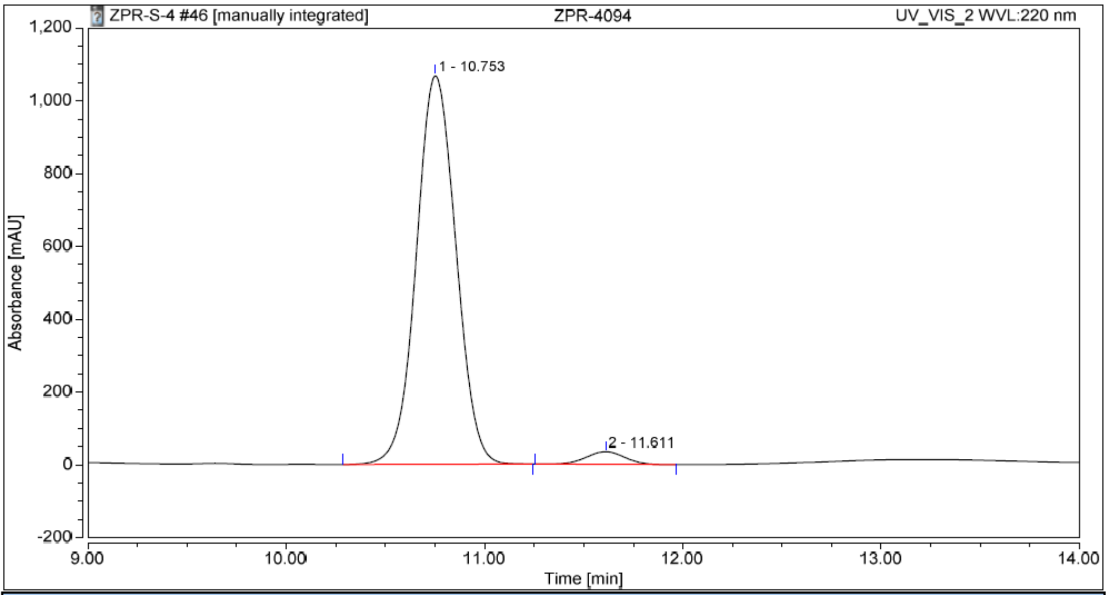

Integration Results							
No.	Peak Name	Retention Time min	Area mAU*min	Height mAU	Relative Area %	Relative Height %	Amount n.a.
1		10.753	246.817	1066.965	96.94	96.86	n.a.
2		11.611	7.804	34.626	3.06	3.14	n.a.
**Total:**			**254.621**	**1101.592**	**100.00**	**100.00**	

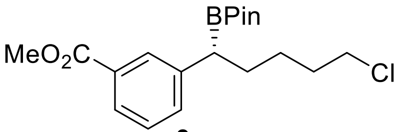

3gg

The title compound **3gg** was synthesized according to General Procedure B, and it was purified by column chromatography on silica gel (83% yield, 94% *ee*, 60.8 mg, colorless oil).

^{1}H NMR (400 MHz, Chloroform-*d*) δ 7.88 (s, 1H), 7.83 (d, *J* = 6.4 Hz, 1H), 7.41 (d, *J* = 7.6 Hz, 1H), 7.33 (dd, *J* = 7.6, 6.4 Hz, 1H), 3.90 (s, 3H), 3.50 (m, 2H), 2.36 (dd, *J* = 8.0, 8.0 Hz, 1H), 1.95-1.83 (m, 1H), 1.81-1.73 (m, 2H), 1.72-1.67 (m, 1H), 1.50-1.34 (m, 2H), 1.21 (s, 6H), 1.19 (s, 6H); ^{13}C NMR (101 MHz, Chloroform-*d*) δ 167.3, 143.4, 133.0, 130.1, 129.4, 128.3, 126.6, 83.5, 52.0, 44.9, 32.5, 31.6, 26.4, 24.6, 24.5; HRMS: m/z (ESI) calculated $[M+K]^+$: 405.1401, found: 405.1406. $[\alpha]_D^{25}$ = -9.725 (*c* = 0.25, $CHCl_3$).

The enantiomeric excess of **3gg** was determined by chiral HPLC analysis compared to the corresponding racemate alcohol.

Conditions: ChiralPak IG column; hexane/^{i}PrOH = 90:10; flow rate = 1.0 mL/min; λ= 220 nm; t_{R1}(major)= 19.6 min; t_{R2}(minor)= 20.1 min.

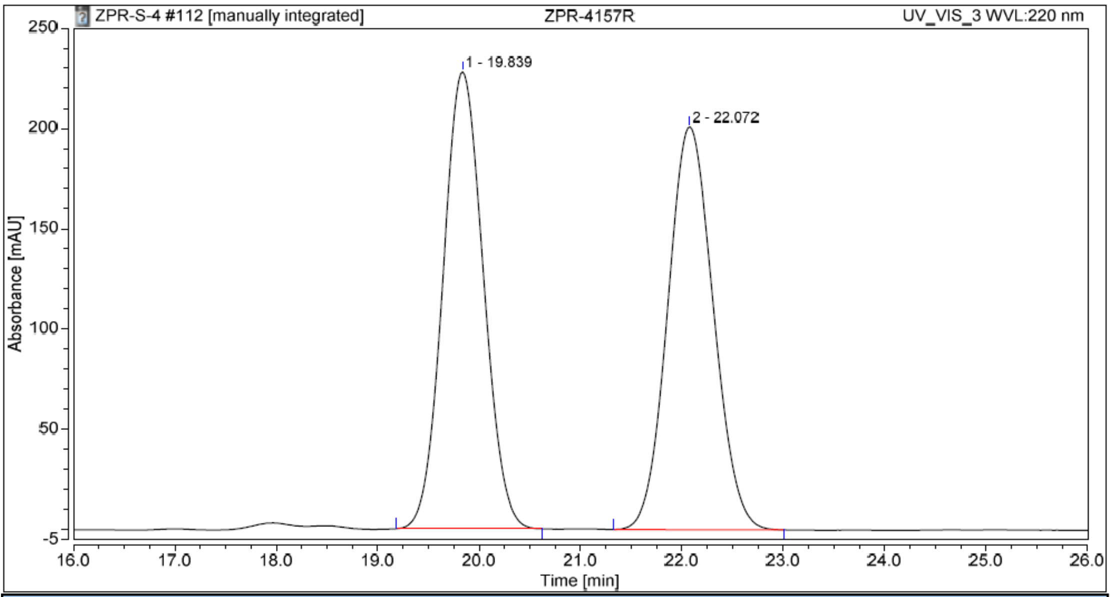

Integration Results							
No.	Peak Name	Retention Time min	Area mAU*min	Height mAU	Relative Area %	Relative Height %	Amount n.a.
1		19.839	105.170	227.952	50.13	53.15	n.a.
2		22.072	104.624	200.934	49.87	46.85	n.a.
**Total:**			**209.794**	**428.886**	**100.00**	**100.00**	

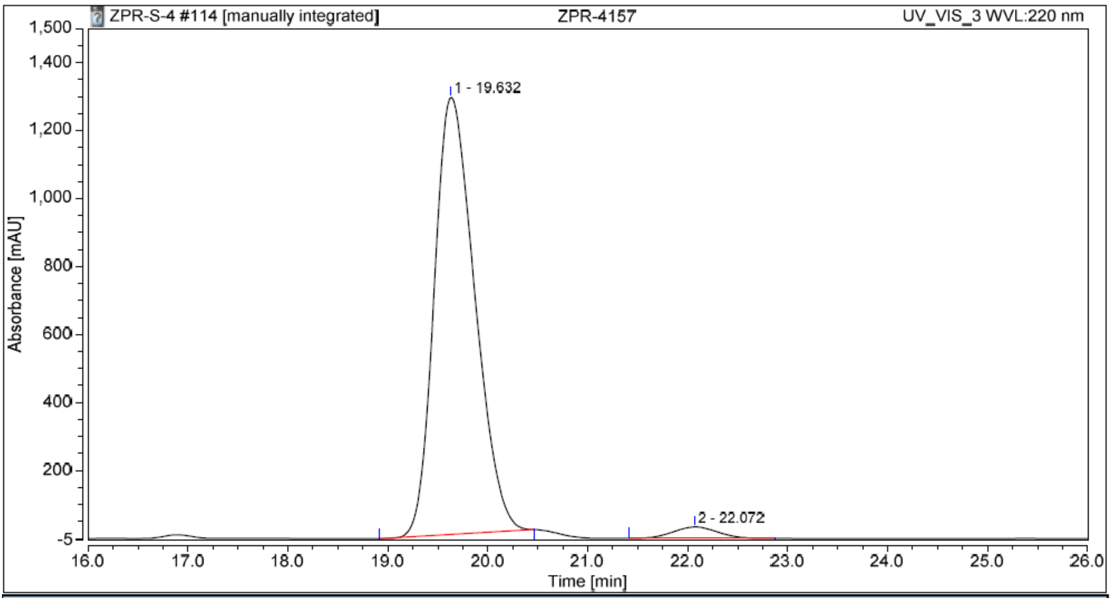

Integration Results							
No.	Peak Name	Retention Time min	Area mAU*min	Height mAU	Relative Area %	Relative Height %	Amount n.a.
1		19.632	601.435	1285.852	97.15	97.44	n.a.
2		22.072	17.672	33.758	2.85	2.56	n.a.
Total:			619.106	1319.609	100.00	100.00	

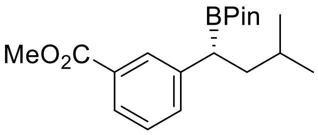

3hh

The title compound **3hh** was synthesized according to General Procedure B, and it was purified by column chromatography on silica gel (51% yield, 95% *ee*, 33.9 mg, white solid).

^{1}H NMR (400 MHz, Chloroform-*d*) δ 7.89 (s, 1H), 7.81 (d, *J* = 7.8 Hz, 1H), 7.42 (d, *J* = 7.4 Hz, 1H), 7.32 (dd, *J* = 7.8, 7.4 Hz, 1H), 3.90 (s, 3H), 2.48 (dd, *J* = 8.0, 8.0 Hz, 1H), 1.74-1.67 (m, 1H), 1.65-1.57 (m, 1H), 1.50-1.41 (m, 2H), 1.19 (s, 6H), 1.18 (s, 6H), 0.88 (t, *J* = 7.4 Hz, 6H); ^{13}C NMR (101 MHz, Chloroform-*d*) δ 167.4, 143.9, 133.0, 130.0, 129.4, 128.2, 126.4, 83.3, 51.9, 41.3, 26.8, 24.5, 24.5, 22.9, 22.1; HRMS: m/z (ESI) calculated $[M+H]^+$: 333.2232, found: 333.2237. $[\alpha]_D^{25}$ = -13.431 (*c* = 0.25, $CHCl_3$).
The enantiomeric excess of **3hh** was determined by chiral HPLC analysis compared to the corresponding racemate.
Conditions: ChiralPak IF column; hexane/^{i}PrOH = 95:5; flow rate = 1.0 mL/min; λ= 220 nm; t_{R1}(major)= 18.0 min; t_{R2}(minor)= 19.5 min.

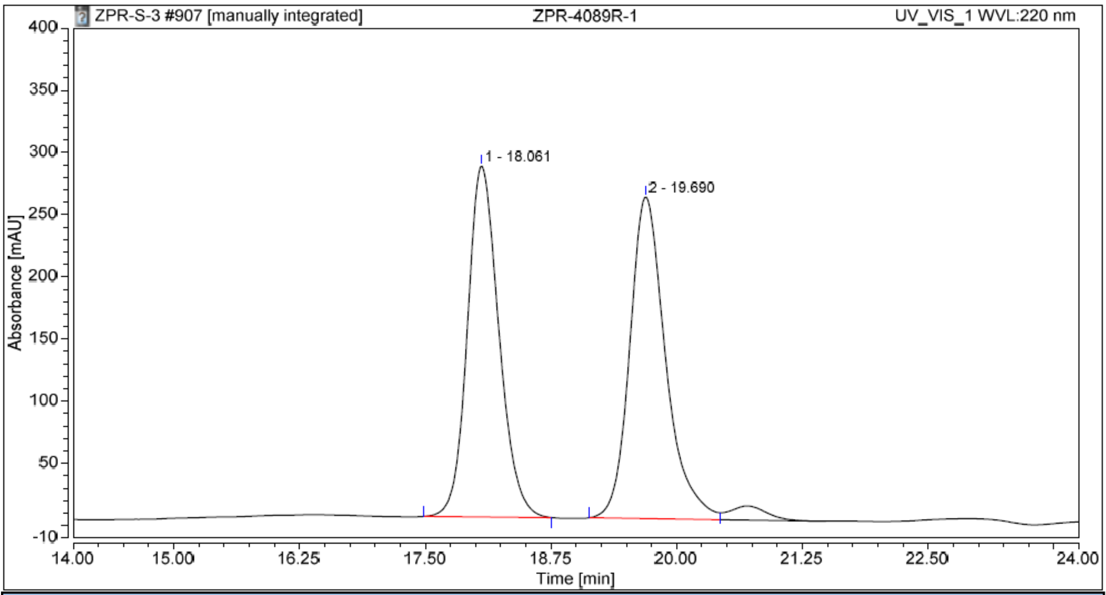

Integration Results

No.	Peak Name	Retention Time<br>min	Area<br>mAU*min	Height<br>mAU	Relative Area<br>%	Relative Height<br>%	Amount<br>n.a.
1		18.061	102.500	282.477	48.92	52.18	n.a.
2		19.690	107.004	258.837	51.08	47.82	n.a.
**Total:**			**209.504**	**541.314**	**100.00**	**100.00**	

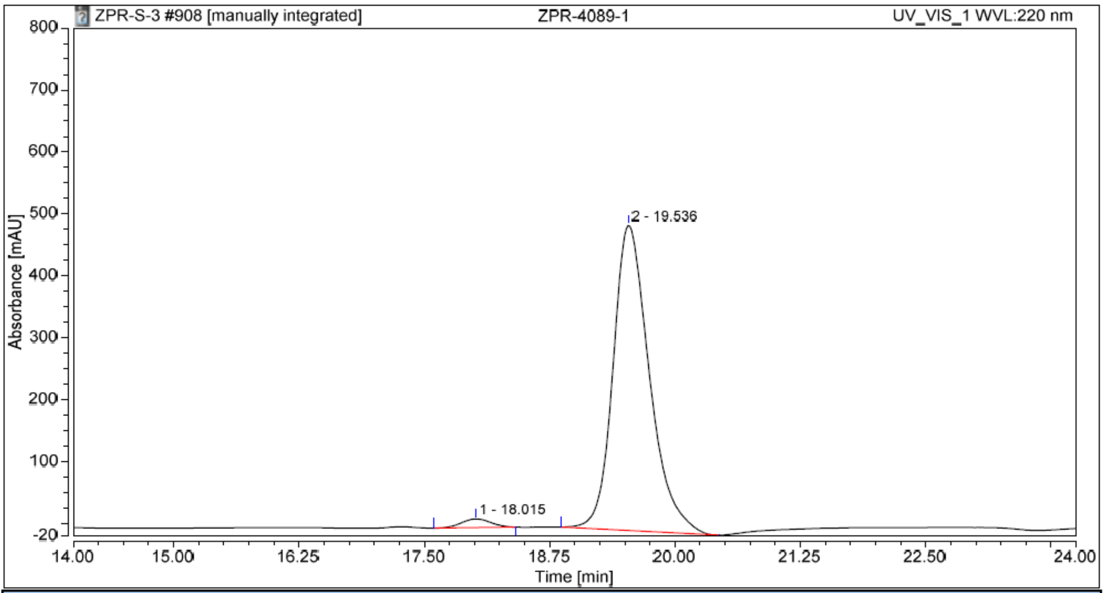

Integration Results

No.	Peak Name	Retention Time<br>min	Area<br>mAU*min	Height<br>mAU	Relative Area<br>%	Relative Height<br>%	Amount<br>n.a.
1		18.015	4.740	13.985	2.24	2.76	n.a.
2		19.536	206.886	492.417	97.76	97.24	n.a.
**Total:**			**211.626**	**506.402**	**100.00**	**100.00**	

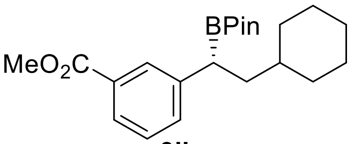

3ii

The title compound **3ii** was synthesized according to General Procedure B, and it was purified by column chromatography on silica gel (59% yield, 94% *ee*, 43.9 mg, white solid).

^{1}H NMR (400 MHz, Chloroform-*d*) δ 7.89 (s, 1H), 7.81 (d, *J* = 7.8 Hz, 1H), 7.40 (d, *J* = 7.8 Hz, 1H), 7.31 (dd, *J* = 7.8, 7,8 Hz, 1H), 3.90 (s, 3H), 2.51 (dd, *J* = 8.0, 8.0 Hz, 1H), 1.83-1.51 (m, 8H), 1.19 (s, 6H), 1.18 (s, 6H), 1.16-1.06 (m, 4H), 0.94-0.81 (m, 2H); ^{13}C NMR (101 MHz, Chloroform-*d*) δ 167.4, 144.1, 133.0, 130.0, 129.4, 128.2, 126.4, 83.3, 51.9, 39.8, 36.6, 33.6, 32.9, 26.6, 26.3, 26.2, 24.5, 24.5; HRMS: m/z (ESI) calculated $[M+Na]^+$: 395.2364, found: 395.2367. $[\alpha]_D^{25}$ = -26.032 (*c* = 0.25, $CHCl_3$).
The enantiomeric excess of **3ii** was determined by chiral HPLC analysis compared to the corresponding racemate alcohol.
Conditions: ChiralPak IG column; hexane/^{i}PrOH = 90:10; flow rate = 1.0 mL/min; λ= 220 nm; t_{R1}(major)= 17.7 min; t_{R2}(minor)= 15.7 min.

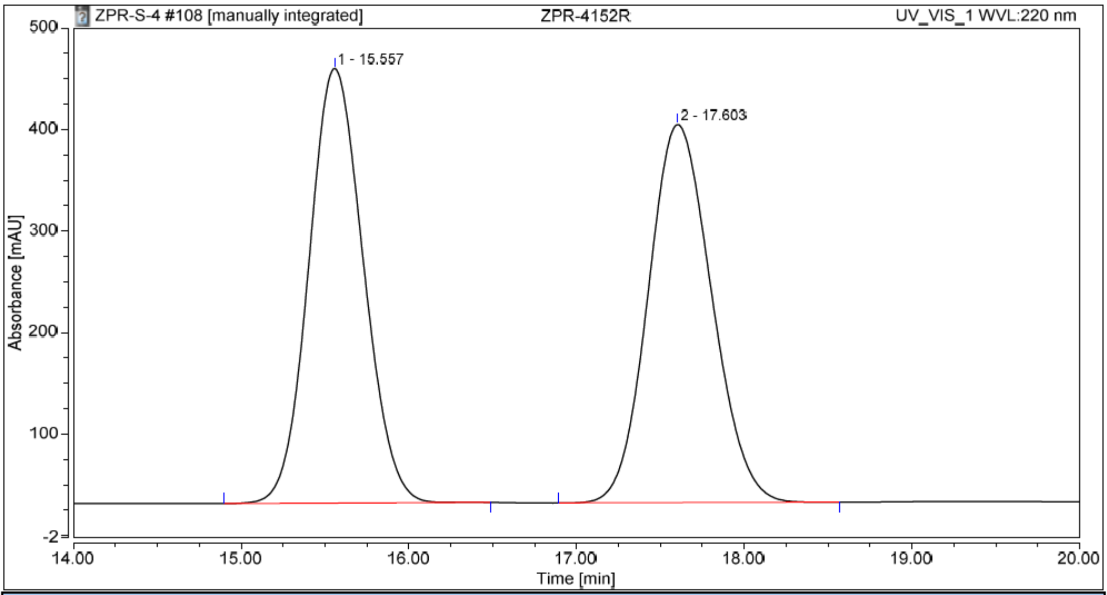

Integration Results

No.	Peak Name	Retention Time min	Area mAU*min	Height mAU	Relative Area %	Relative Height %	Amount n.a.
1		15.557	161.310	428.071	49.70	53.48	n.a.
2		17.603	163.238	372.370	50.30	46.52	n.a.
**Total:**			**324.547**	**800.441**	**100.00**	**100.00**	

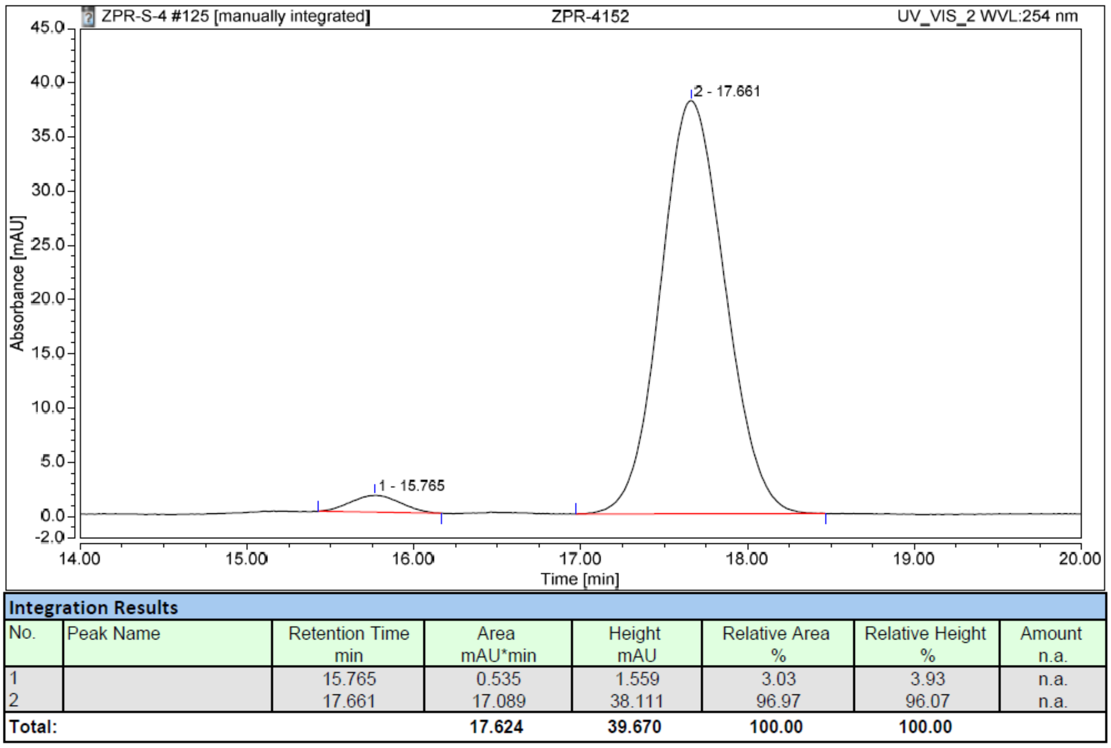

Integration Results							
No.	Peak Name	Retention Time min	Area mAU*min	Height mAU	Relative Area %	Relative Height %	Amount n.a.
1		15.765	0.535	1.559	3.03	3.93	n.a.
2		17.661	17.089	38.111	96.97	96.07	n.a.
**Total:**			**17.624**	**39.670**	**100.00**	**100.00**	

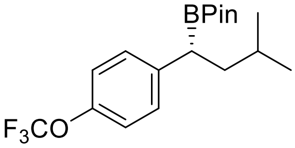

3jj

The title compound **3jj** was synthesized according to General Procedure B, and it was purified by column chromatography on silica gel (69% yield, 95% *ee*, 49.4 mg, colorless oil)

^{1}H NMR (600 MHz, Chloroform-*d*) δ 7.21 (d, *J* = 8.6 Hz, 2H), 7.08 (d, *J* = 8.6 Hz, 2H), 2.43 (dd, *J* = 8.0, 8.0 Hz, 1H), 1.69-1.64 (m, 1H), 1.60-1.53 (m, 1H), 1.49-1.42 (m, 1H), 1.19 (s, 6H), 1.18 (s, 6H), 0.88 (d, *J* = 6.5 Hz, 3H), 0.87 (d, *J* = 6.6 Hz, 3H); ^{13}C NMR (151 MHz, Chloroform-*d*) δ 146.8, 142.2, 129.4, 120.7, 120.5 (q, *J* = 256.3 Hz), 83.4, 41.4, 26.8, 24.5, 24.5, 22.9, 22.1; HRMS: m/z (ESI) calculated $[M+Na]^+$:381.1819, found: 381.1832. $[\alpha]_D^{25}$ = -25.377 (*c* = 0.25, $CHCl_3$).
The enantiomeric excess of **3jj** was determined by chiral HPLC analysis compared to the corresponding racemate alcohol.
Conditions: ChiralPak IA column; hexane/^{i}PrOH = 99:1; flow rate = 1.0 mL/min; λ= 220 nm; t_{R1}(major)= 17.7 min; t_{R2}(minor)= 16.7 min.

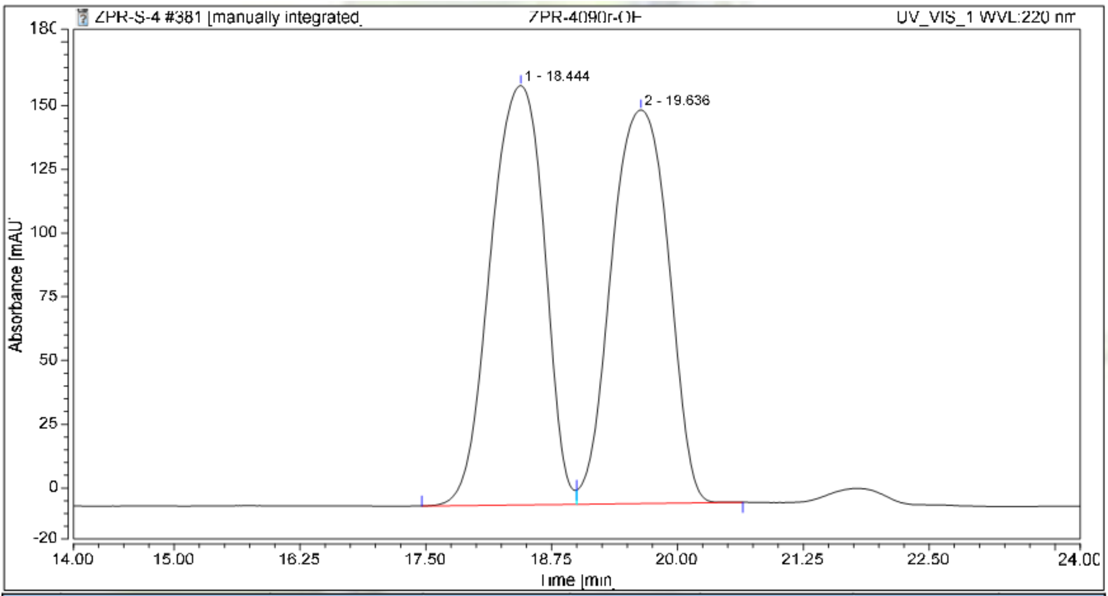

Integration Results							
No.	Peak Name	Retention Time min	Area mAU*min	Height mAU	Relative Area %	Relative Height %	Amount n.a.
1		18.444	102.110	164.537	50.08	51.58	n.a.
2		19.636	101.766	154.441	49.92	48.42	n.a.
**Total:**			**203.876**	**318.977**	**100.00**	**100.00**	

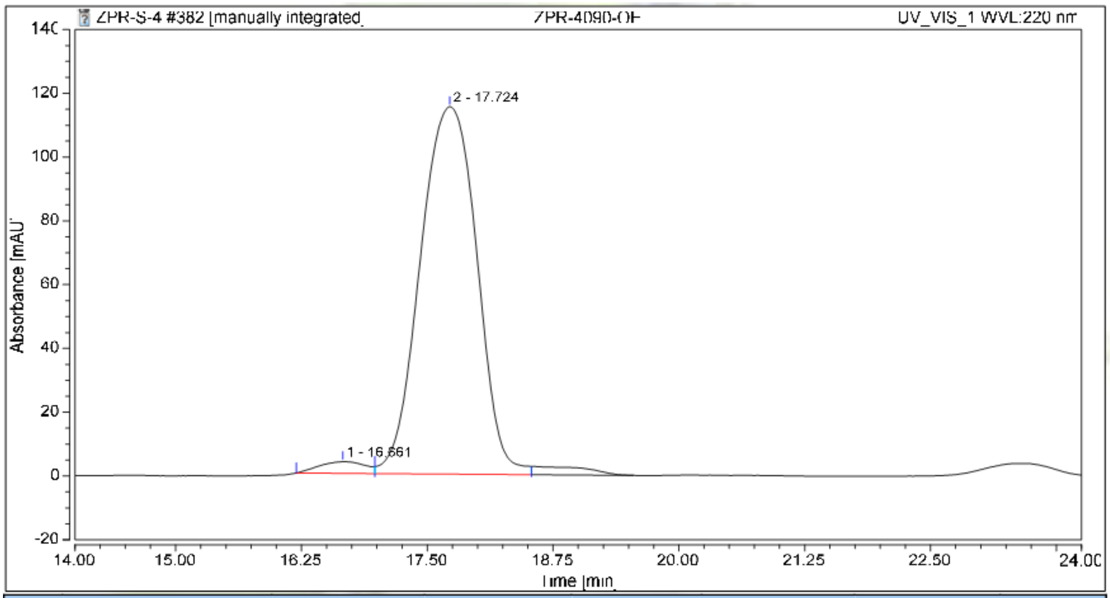

Integration Results							
No.	Peak Name	Retention Time min	Area mAU*min	Height mAU	Relative Area %	Relative Height %	Amount n.a.
1		16.661	1.919	3.633	2.49	3.06	n.a.
2		17.724	75.113	115.187	97.51	96.94	n.a.
**Total:**			**77.032**	**118.819**	**100.00**	**100.00**	

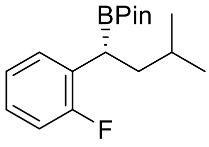

3kk

The title compound **3kk** was synthesized according to General Procedure B, and it

was purified by column chromatography on silica gel (55% yield, 92% *ee*, 32.1 mg, colorless oil)

^{1}H NMR (400 MHz, Chloroform-*d*) δ 7.26-7.20 (m, 1H), 7.13-7.06 (m, 1H), 7.05-7.00 (m, 1H), 7.00-6.93 (m, 1H), 2.67-2.56 (m, 1H), 1.66-1.61 (m, 2H), 1.47-1.39 (m, 1H), 1.22 (s, 6H), 1.20 (s, 6H), 0.89 (d, *J* = 6.4 Hz, 3H), 0.86 (d, *J* = 6.6 Hz, 3H); ^{13}C NMR (151 MHz, Chloroform-*d*) δ 160.9 (d, *J* = 243.5 Hz), 130.5 (d, *J* = 16.0 Hz), 130.3 (d, *J* = 5.0 Hz), 126.5 (d, *J* = 8.2 Hz), 123.8 (d, *J* = 3.4 Hz), 115.0 (d, *J* = 22.9 Hz), 83.3, 40.1, 26.6, 24.6, 24.5, 23.1, 21.8; HRMS: m/z (ESI) calculated $[M+Na]^+$:315.1902, found: 315.1903. $[\alpha]_D^{25}$ = -14.208 (*c* = 0.25, $CHCl_3$).
The enantiomeric excess of **3kk** was determined by chiral HPLC analysis compared to the corresponding racemate alcohol.
Conditions: ChiralPak IG column; hexane/^{i}PrOH = 99:1; flow rate = 1.0 mL/min; λ= 254 nm; t_{R1}(major)= 21.1 min; t_{R2}(minor)= 18.7 min.

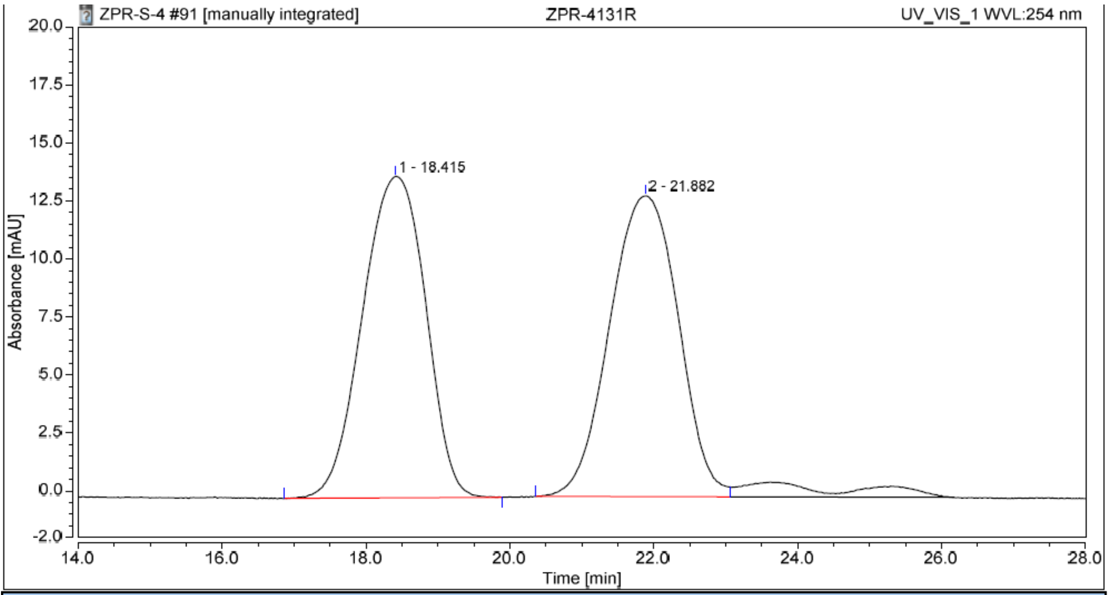

Integration Results

No.	Peak Name	Retention Time min	Area mAU*min	Height mAU	Relative Area %	Relative Height %	Amount n.a.
1		18.415	14.085	13.852	49.59	51.65	n.a.
2		21.882	14.319	12.968	50.41	48.35	n.a.
**Total:**			**28.403**	**26.820**	**100.00**	**100.00**	

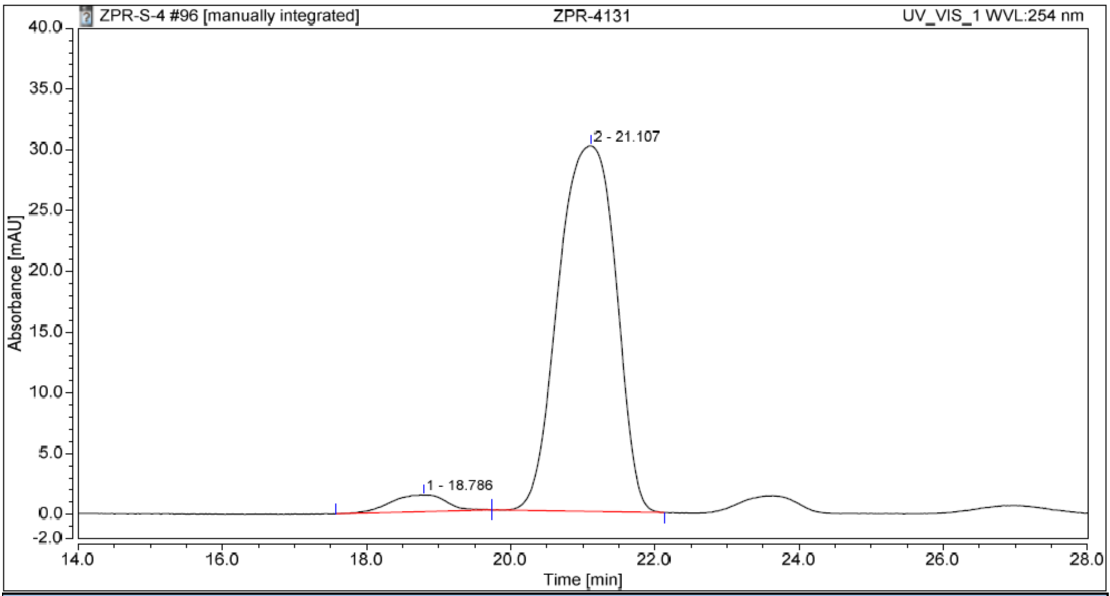

Integration Results

No.	Peak Name	Retention Time min	Area mAU*min	Height mAU	Relative Area %	Relative Height %	Amount n.a.
1		18.786	1.213	1.377	4.18	4.38	n.a.
2		21.107	27.810	30.072	95.82	95.62	n.a.
**Total:**			**29.024**	**31.449**	**100.00**	**100.00**	

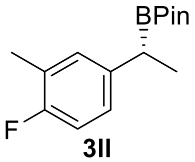

3ll

The title compound **3ll** was synthesized according to General Procedure B, and it was purified by column chromatography on silica gel (35% yield, 87% *ee*, 18.5 mg, colorless oil).

^{1}H NMR (400 MHz, Chloroform-d) δ 7.03-6.94 (m, 2H), 6.92-6.83 (m,1H), 2.36 (q, J = 7.5 Hz, 1H), 2.23 (d, J = 1.9 Hz, 3H), 1.29 (d, J = 7.5 Hz, 3H), 1.21 (s, 6H), 1.20 (s, 6H); ^{13}C NMR (101 MHz, Chloroform-d) δ 159.4 (d, J = 241.2 Hz), 140.2 (d, J = 3.6 Hz), 130.6 (d, J = 4.9 Hz), 126.2 (d, J = 7.7 Hz), 124.2 (d, J = 17.1 Hz), 114.6 (d, J = 22.0 Hz), 83.3, 24.6, 24.6, 17.3, 14.6 (d, J = 3.4 Hz); HRMS: m/z (ESI) calculated $[M+Na]^+$: 287.1589, found: 287.1599. $[\alpha]_D^{25}$ = +1.816 (c = 0.25, $CHCl_3$).
The enantiomeric excess of **3ll** was determined by chiral HPLC analysis compared to the corresponding racemate alcohol.
Conditions: ChiralPak IA column; hexane/^{i}PrOH = 99:1; flow rate = 1.0 mL/min; λ= 220 nm; t_{R1}(major)= 23.4 min; t_{R2}(minor)= 24.8 min.

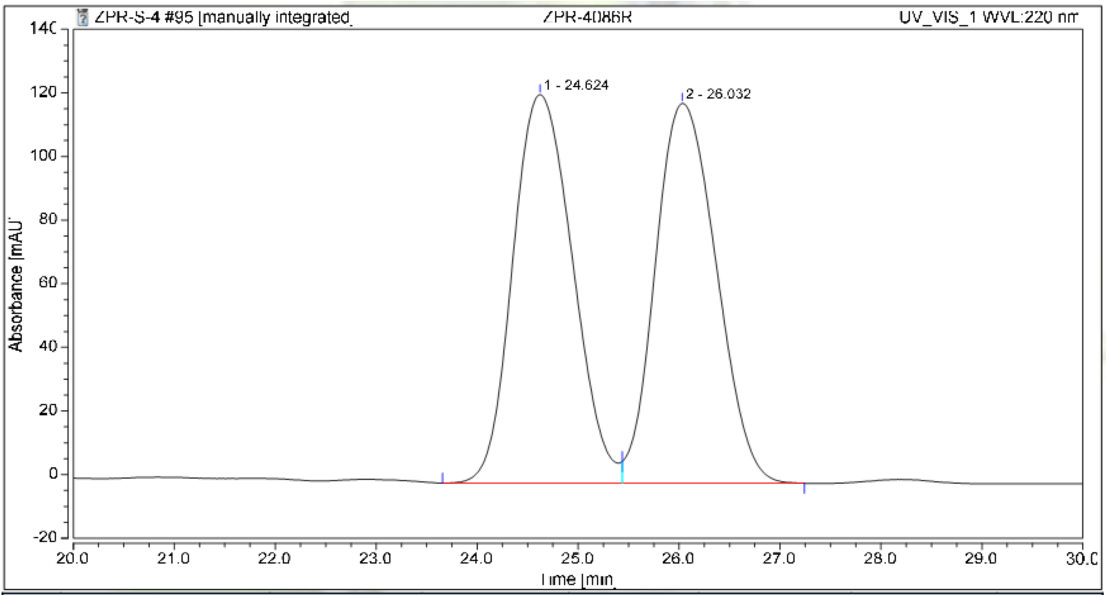

Integration Results

No.	Peak Name	Retention Time<br>min	Area<br>mAU*min	Height<br>mAU	Relative Area<br>%	Relative Height<br>%	Amount<br>n.a.
1		24.624	83.605	122.164	50.04	50.57	n.a.
2		26.032	83.480	119.411	49.96	49.43	n.a.
**Total:**			**167.085**	**241.575**	**100.00**	**100.00**	

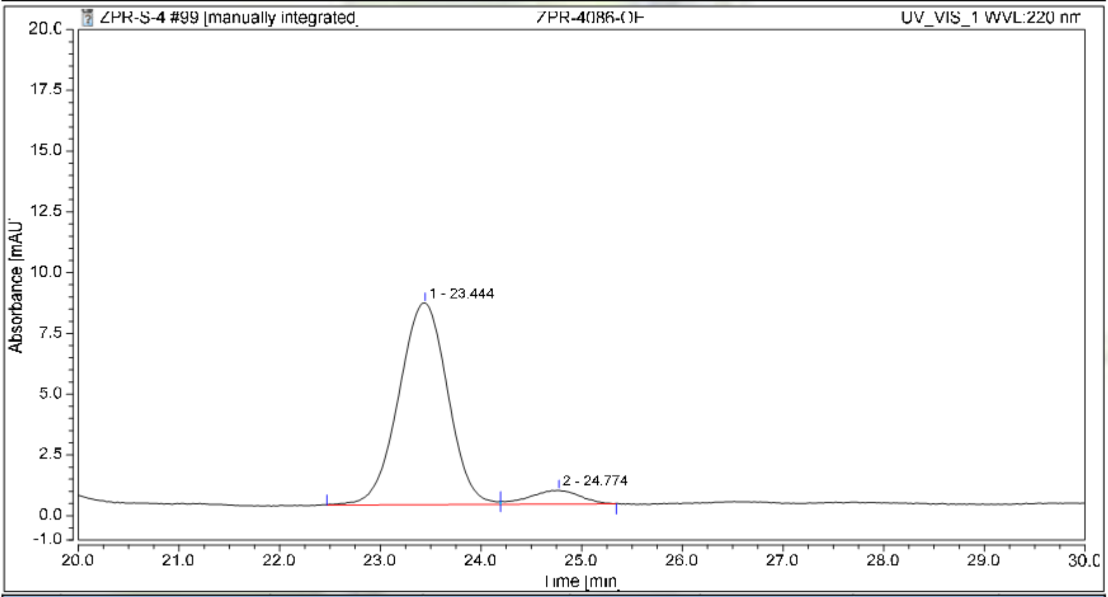

Integration Results

No.	Peak Name	Retention Time<br>min	Area<br>mAU*min	Height<br>mAU	Relative Area<br>%	Relative Height<br>%	Amount<br>n.a.
1		23.444	4.660	8.298	93.48	93.59	n.a.
2		24.774	0.325	0.568	6.52	6.41	n.a.
**Total:**			**4.985**	**8.866**	**100.00**	**100.00**	

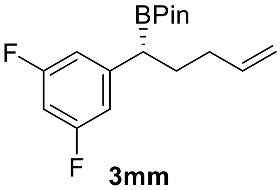

3mm

The title compound **3mm** was synthesized according to General Procedure B, and it was purified by column chromatography on silica gel (40% yield, 83% *ee*, 24.6 mg, colorless oil).

^{1}H NMR (600 MHz, Chloroform-*d*) δ 6.76-6.68 (m, 2H), 6.62-6.50 (m, 1H), 5.78 (ddt, *J* = 17.0, 10.2, 6.7 Hz, 1H), 5.05-4.90 (m, 1H), 2.33(dd, *J* = 7.9 Hz, 1H), 2.04-1.97 (m, 2H), 1.94-1.87 (m, 1H), 1.75-1.68 (m, 1H), 1.21 (s, 6H), 1.20 (s, 6H); ^{13}C NMR (151 MHz, Chloroform-*d*) δ 162.9 (dd, *J* = 247.1, 13.2 Hz), 147.2 (t, *J* = 8.9 Hz), 138.1, 115.1, 111.0 (dd, *J* = 19.6, 4.7 Hz), 100.7 (t, *J* = 25.4 Hz), 83.6, 33.0, 31.2, 24.6, 24.5; HRMS: m/z (ESI) calculated $[M+NH_4]^+$: 326.2097, found: 326.2095. $[\alpha]_D^{25}$ = -6.696 (*c* = 0.25, $CHCl_3$).

The enantiomeric excess of **3mm** was determined by chiral HPLC analysis compared to the corresponding racemate.

Conditions: ChiralPak IG column; hexane/^{i}PrOH = 98:2; flow rate = 1.0 mL/min; λ= 254 nm; t_{R1}(major)= 22.6 min; t_{R2}(minor)= 25.6 min.

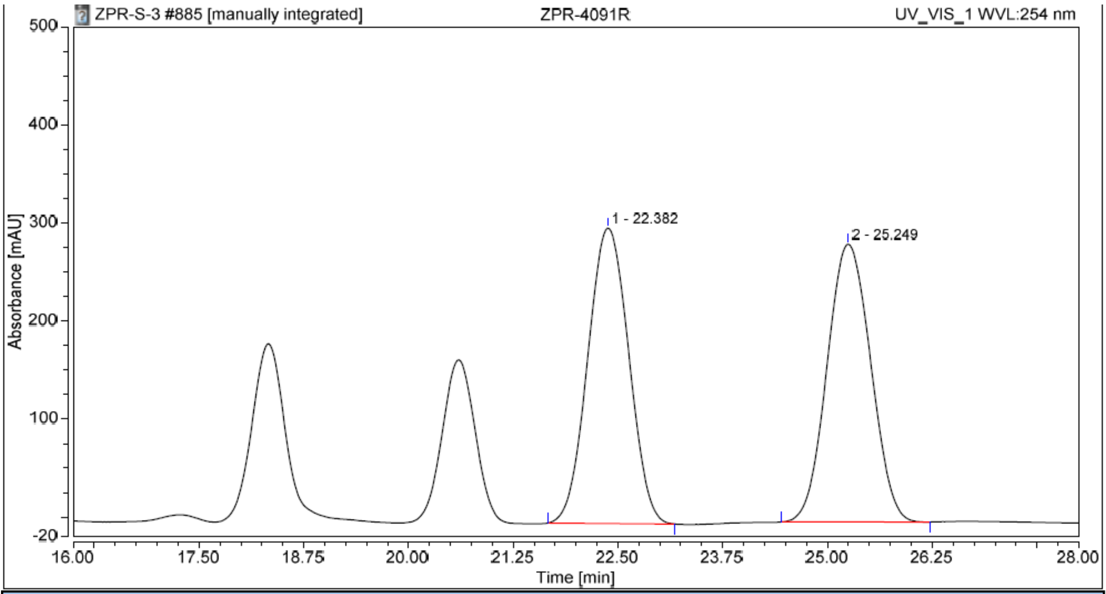

Integration Results							
No.	Peak Name	Retention Time min	Area mAU*min	Height mAU	Relative Area %	Relative Height %	Amount n.a.
1		22.382	170.606	301.269	49.97	51.54	n.a.
2		25.249	170.787	283.290	50.03	48.46	n.a.
**Total:**			**341.393**	**584.559**	**100.00**	**100.00**	

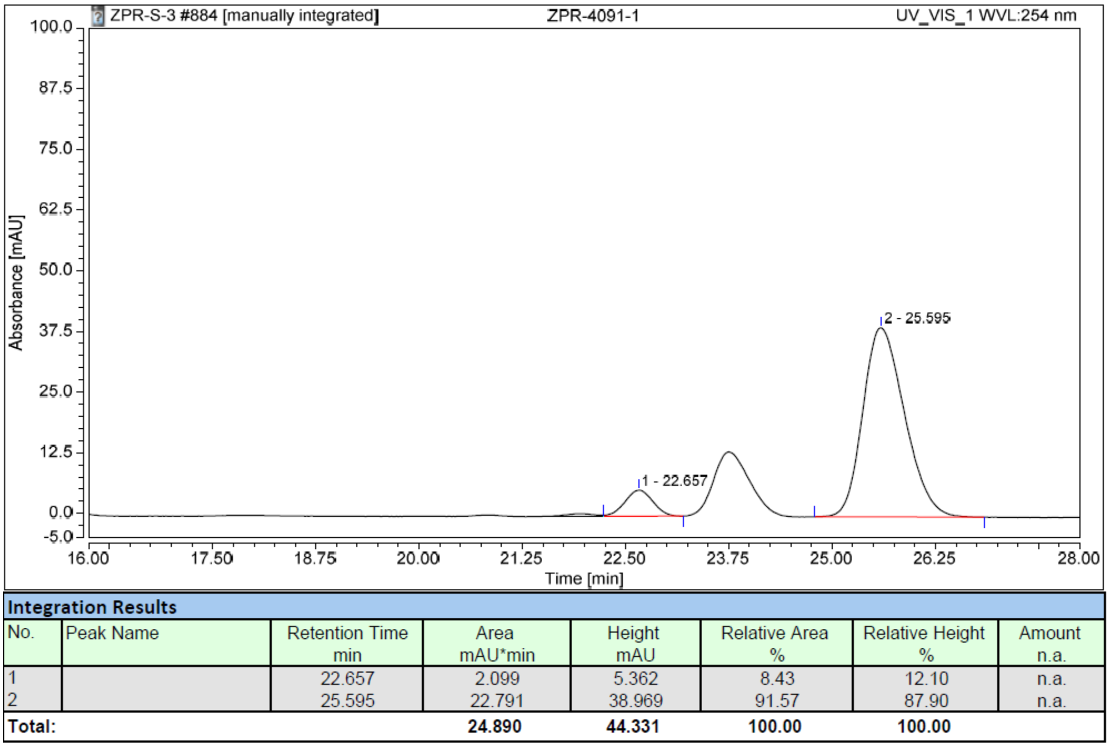

Integration Results

No.	Peak Name	Retention Time min	Area mAU*min	Height mAU	Relative Area %	Relative Height %	Amount n.a.
1		22.657	2.099	5.362	8.43	12.10	n.a.
2		25.595	22.791	38.969	91.57	87.90	n.a.
**Total:**			**24.890**	**44.331**	**100.00**	**100.00**	

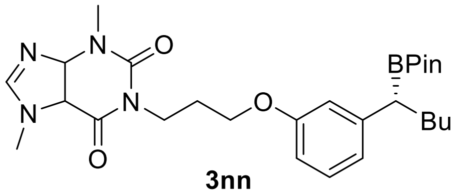

3nn

The title compound **3nn** was synthesized according to General Procedure B, and it was purified by column chromatography on silica gel (46% yield, 94% *ee*, 47.1 mg, white solid).

^{1}H NMR (400 MHz, Chloroform-*d*) δ 7.50 (s, 1H), 7.11 (t, *J* = 7.9 Hz, 1H), 6.77 (d, *J* = 7.6 Hz, 1H), 6.68 (s, 1H), 6.59 (dd, *J* = 8.2, 2.5 Hz, 1H), 4.23 (t, *J* = 6.8 Hz, 2H), 4.04 (t, *J* = 6.1 Hz, 2H), 3.94 (s, 3H), 3.57 (s, 3H), 2.22 (dd, *J* = 7.9, 8.0 Hz, 1H), 2.20 -2.11 (m, 2H), 1.84-1.77 (m, 1H), 1.66-1.56 (m, 1H), 1.32-1.22 (m, 2H), 1.21 (s, 6H), 1.19 (s, 6H), 0.86 (t, *J* = 7.0 Hz, 3H); ^{13}C NMR (101 MHz, Chloroform-*d*) δ 158.8, 155.3, 151.5, 148.8, 145.0, 141.3, 128.9, 120.7, 114.5, 111.1, 107.7, 83.2, 65.9, 39.1, 33.5, 32.2, 31.5, 29.6, 28.0, 24.60, 24.58, 22.7, 14.0; HRMS: m/z (ESI) calculated $[M]^+$: 512.3165, found: 512.3139. $[\alpha]_D^{25}$ = -13.896 (*c* = 0.25, $CHCl_3$).
The enantiomeric excess of **3nn** was determined by chiral HPLC analysis compared to the corresponding racemate.
Conditions: ChiralPak IA column; hexane/^{i}PrOH = 95:5; flow rate = 1.0 mL/min; λ= 254 nm; t_{R1}(major)= 41.6 min; t_{R2}(minor)= 40.9 min.

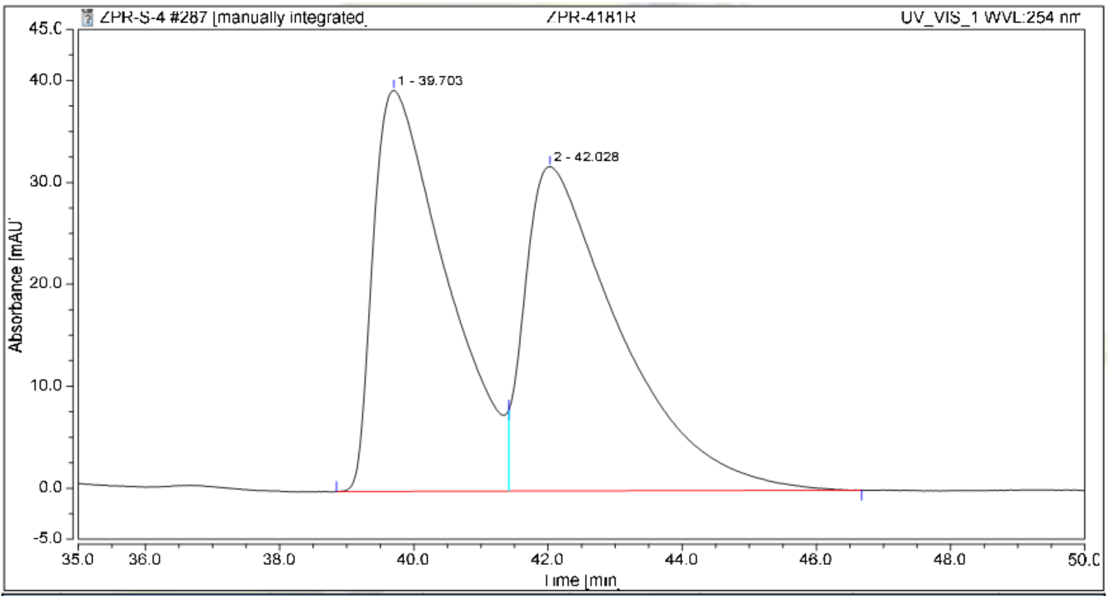

Integration Results							
No.	Peak Name	Retention Time min	Area mAU*min	Height mAU	Relative Area %	Relative Height %	Amount n.a.
1		39.703	49.843	39.365	48.76	55.28	n.a.
2		42.028	52.368	31.841	51.24	44.72	n.a.
**Total:**			**102.211**	**71.206**	**100.00**	**100.00**	

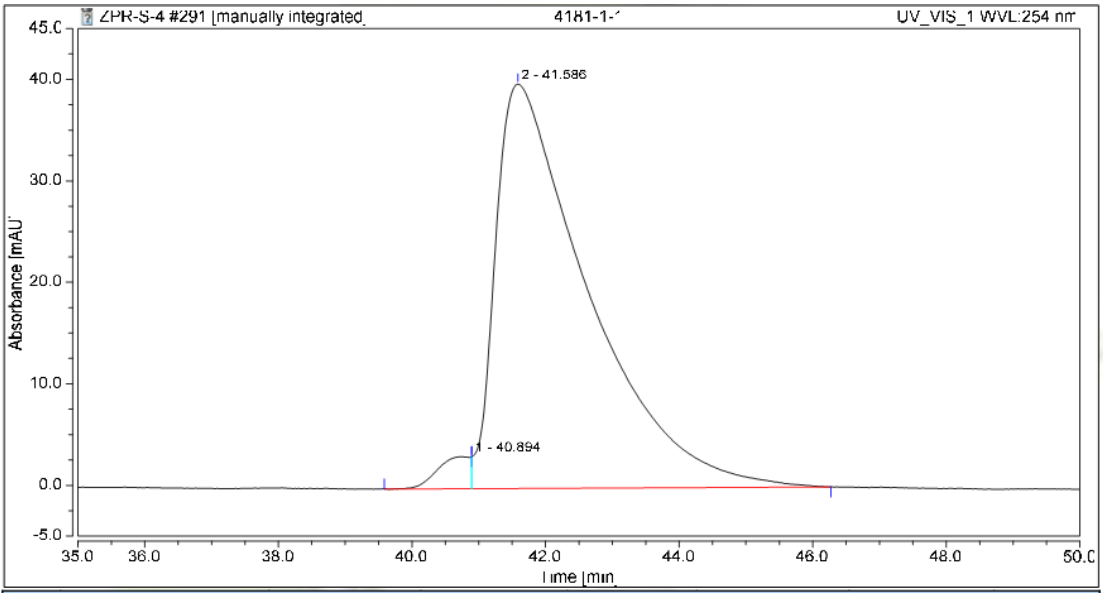

Integration Results							
No.	Peak Name	Retention Time min	Area mAU*min	Height mAU	Relative Area %	Relative Height %	Amount n.a.
1		40.894	1.783	3.153	2.72	7.33	n.a.
2		41.586	63.882	39.845	97.28	92.67	n.a.
**Total:**			**65.665**	**42.998**	**100.00**	**100.00**	

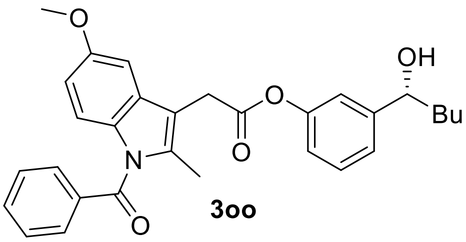

3oo

The title compound **3oo** was synthesized according to General Procedure B, and it was purified by column chromatography on silica gel (43% yield, 90% *ee*, 41.7 mg, white solid).

^{1}H NMR (400 MHz, Chloroform-*d*) δ 7.68 (d, *J* = 8.5 Hz, 2H), 7.47 (d, *J* = 8.5 Hz, 2H), 7.32 (dd, *J* = 7.8, 8.0 Hz, 1H), 7.17 (d, *J* = 7.8 Hz, 1H), 7.11-7.02 (m, 2H), 7.02 -6.93 (m, 1H), 6.90 (d, *J* = 9.0 Hz, 1H), 6.69 (dd, *J* = 9.0, 2.5 Hz, 1H), 4.64 (dd, *J* = 7.6, 7.8 Hz, 1H), 3.90 (s, 2H), 2.45 (s, 3H), 1.81-1.59 (m, 3H), 1.43-1.24 (m, 4H), 0.87 (t, *J* = 6.9 Hz, 3H); ^{13}C NMR (101 MHz, Chloroform-*d*) δ 169.3, 168.3, 156.1, 150.8, 146.9, 139.3, 136.2, 133.8, 131.2, 130.8, 130.5, 129.3, 129.1, 123.4, 120.3, 118.8, 115.0, 112.0, 111.8, 101.2, 74.0, 55.7, 38.8, 30.5, 27.8, 22.5, 14.0, 13.4; HRMS: m/z (ESI) calculated $[M+Na]^+$: 508.2094, found: 508.2107. $[\alpha]_D^{25}$ = +34.1050 (*c* = 0.25, $CHCl_3$).

The enantiomeric excess of **3oo** was determined by chiral HPLC analysis compared to the corresponding racemate.

Conditions: ChiralPak IA column; hexane/^{i}PrOH = 90:10; flow rate = 1.0 mL/min; λ= 254 nm; t_{R1}(major)= 50.1 min; t_{R2}(minor)= 43.7 min.

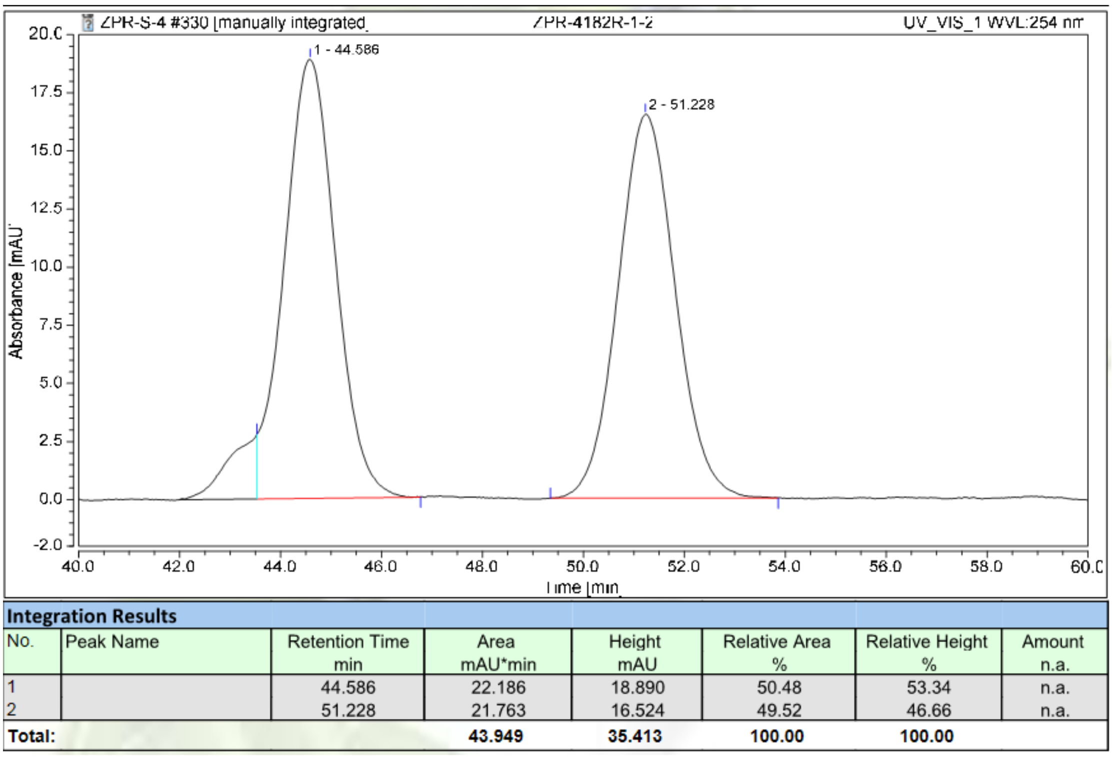

Integration Results							
No.	Peak Name	Retention Time min	Area mAU*min	Height mAU	Relative Area %	Relative Height %	Amount n.a.
1		44.586	22.186	18.890	50.48	53.34	n.a.
2		51.228	21.763	16.524	49.52	46.66	n.a.
Total:			43.949	35.413	100.00	100.00	

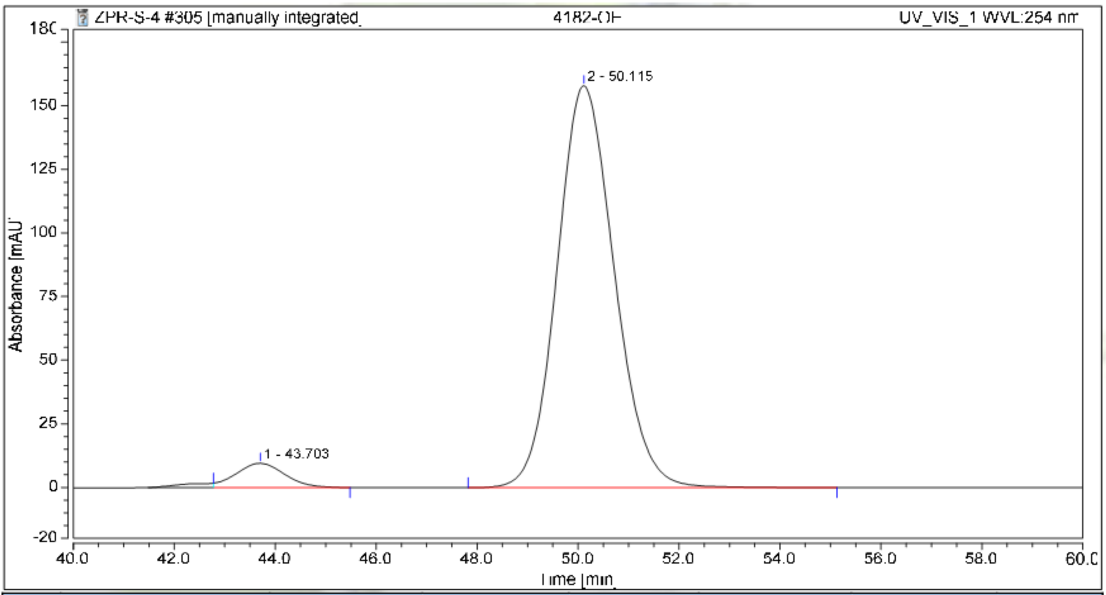

Integration Results

No.	Peak Name	Retention Time min	Area mAU*min	Height mAU	Relative Area %	Relative Height %	Amount n.a.
1		43.703	10.711	9.551	4.90	5.70	n.a.
2		50.115	207.679	158.013	95.10	94.30	n.a.
**Total:**			**218.389**	**167.564**	**100.00**	**100.00**	

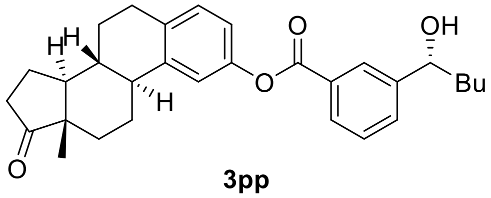

3pp

The title compound **3pp** was synthesized according to General Procedure B, and it was purified by column chromatography on silica gel (62% yield, dr = 18:1, 57.0 mg, white solid).

^{1}H NMR (400 MHz, Chloroform-*d*) δ 8.16 (s, 1H), 8.10 (d, *J* = 7.8 Hz, 1H), 7.63 (d, *J* = 7.8 Hz, 1H), 7.49 (dd, *J* = 7.8, 7.8 Hz, 1H), 7.34 (d, *J* = 8.5 Hz, 1H), 7.02-6.91 (m, 2H), 4.77 (dd, *J* = 6.4, 6.6 Hz, 1H), 2.99-2.92 (m, 2H), 2.51 (dd, *J* = 18.8, 8.6 Hz, 1H), 2.47-2.39 (m, 1H), 2.36-2.27 (m, 1H), 2.22-2.11 (m, 2H), 2.12-1.96 (m, 3H), 1.89-1.71 (m, 2H), 1.66-1.58 (m, 2H), 1.56-1.43 (m, 4H), 1.40-1.26 (m, 4H), 0.92 (s, 3H), 0.90 (t, J = 7.0 Hz, 3H).; ^{13}C NMR (101 MHz, Chloroform-*d*) δ 220.9, 165.4, 148.8, 145.6, 138.0, 137.4, 131.1, 129.7, 129.2, 128.6, 127.5, 126.4, 121.6, 118.8, 74.1, 50.4, 47.9, 44.1, 38.9, 38.0, 35.8, 31.5, 29.4, 27.8, 26.3, 25.7, 22.5, 21.6, 14.0, 13.8; HRMS: m/z (ESI) calculated [M+H]$^+$:461.2686, found: 461.2673. $[\alpha]_D^{25}$ = +90.789 (*c* = 0.25, $CHCl_3$).

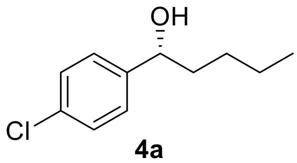

4a

The title compound **4a** was synthesized according to General Procedure E, and it was purified by column chromatography on silica gel (95% yield, 92% *ee*, 37.6 mg, colorless oil).

^{1}H NMR (400 MHz, Chloroform-*d*) δ 7.35-7.23 (m, 4H), 4.64 (dd, *J* = 6.6, 6.4 Hz, 1H), 1.87 (br, 1H), 1.81-1.72 (m, 2H), 1.71-1.61 (m, 2H), 1.35-1.13 (m, 4H), 0.88 (t, *J* = 7.0 Hz, 3H); ^{13}C NMR (101 MHz, Chloroform-*d*) δ 143.3, 133.0, 128.5, 127.3, 74.0, 38.8, 27.8, 22.5, 14.0; (The NMR data were in accordance with those reported [S7]) $[\alpha]_D^{25}$ = +28.575 (*c* = 0.25, $CHCl_3$).

The enantiomeric excess of **4a** was determined by chiral HPLC analysis compared to the corresponding racemate.

Conditions: ChiralPak IG column; hexane/^{i}PrOH = 98:2; flow rate = 1.0 mL/min; λ= 220 nm; t_{R1}(major)= 16.1 min; t_{R2}(minor)= 14.6 min.

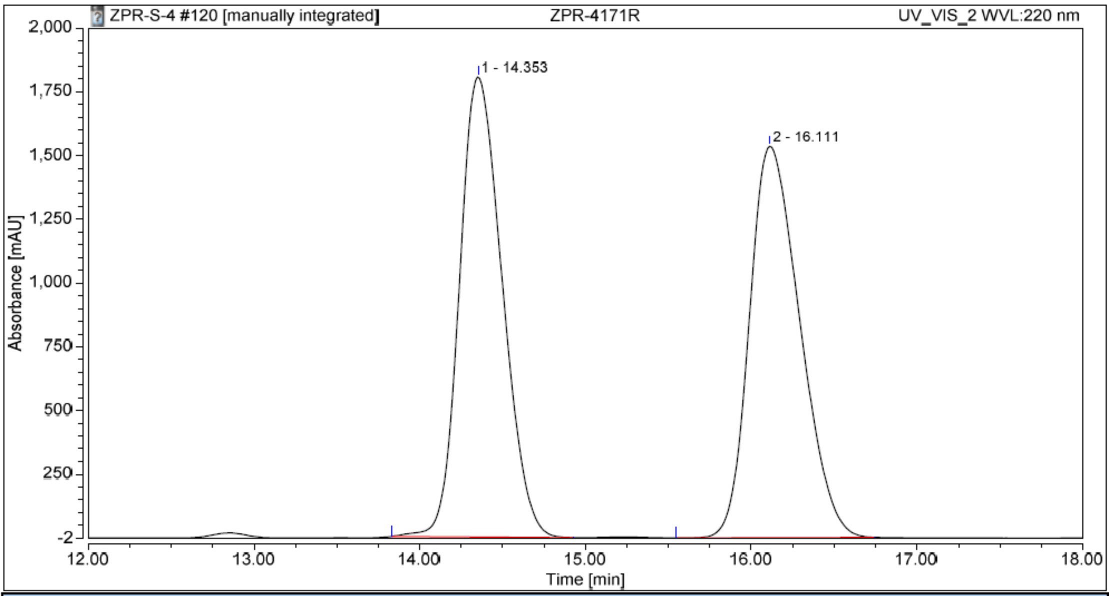

Integration Results

No.	Peak Name	Retention Time min	Area mAU*min	Height mAU	Relative Area %	Relative Height %	Amount n.a.
1		14.353	539.355	1804.908	49.98	54.04	n.a.
2		16.111	539.811	1535.074	50.02	45.96	n.a.
**Total:**			**1079.166**	**3339.982**	**100.00**	**100.00**	

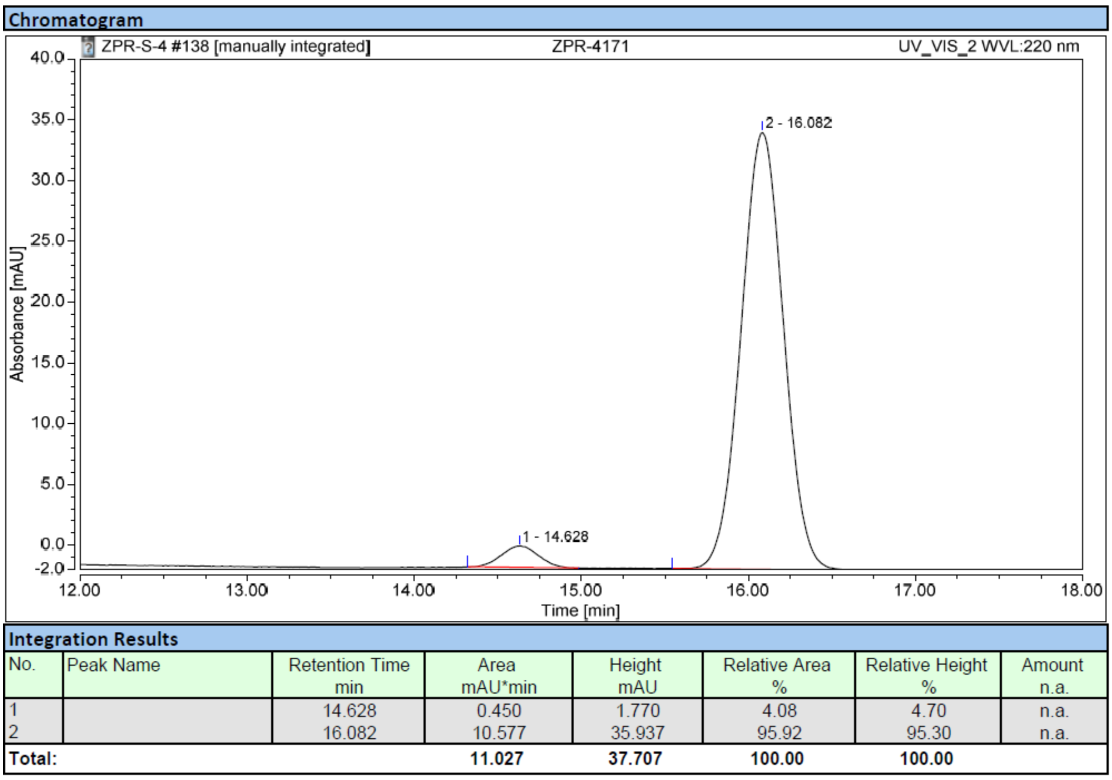

Integration Results

No.	Peak Name	Retention Time min	Area mAU*min	Height mAU	Relative Area %	Relative Height %	Amount n.a.
1		14.628	0.450	1.770	4.08	4.70	n.a.
2		16.082	10.577	35.937	95.92	95.30	n.a.
**Total:**			**11.027**	**37.707**	**100.00**	**100.00**	

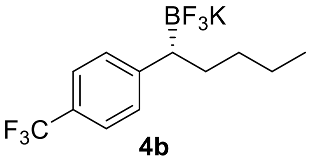

4b

The title compound **4b** was synthesized according to General Procedure E, and it was purified by column chromatography on silica gel (59% yield, 38.0 mg, white solid).

^{1}H NMR (400 MHz, Acetone-d_6) δ 7.39 (d, J = 8.0 Hz, 2H), 7.30 (d, J = 8.0 Hz, 2H), 1.82-1.74 (m, 2H), 1.72-1.60 (m, 1H), 1.34-1.20 (m, 2H), 1.18-1.02 (m, 2H), 0.80 (t, J = 7.2 Hz, 3H); ^{13}C NMR (101 MHz, Acetone-d_6) δ 157.1, 129.6, 126.3 (q, J = 270.2 Hz), 125.0 (q, J = 31.3 Hz), 124.5 (q, J = 3.7 Hz), 32.8, 31.8, 23.7, 14.5; ^{19}F NMR (376 MHz, Acetone-d_6) δ -62.0, -145.3; ^{11}B NMR (128 MHz, Acetone-d_6) δ 4.6. HRMS: m/z (ESI) calculated [M-K]$^-$:283.1087, found:283.1095.

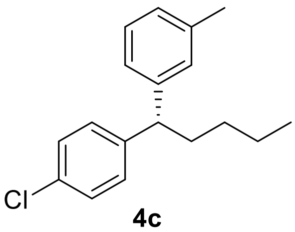

4c

The title compound **4c** was synthesized according to General Procedure E, and it was purified by column chromatography on silica gel (77% yield, 88% *ee*, 41.9 mg, colorless oil).

^{1}H NMR (400 MHz, Chloroform-*d*) δ 7.23 (d, *J* = 8.4 Hz, 2H), 7.19-7.12 (m, 3H), 7.06-6.90 (m, 2H), 3.81 (dd, *J* = 7.8, 7.8 Hz, 1H), 2.30 (s, 3H), 2.08-1.90 (m, 2H), 1.37-1.28 (m, 2H), 1.24-1.16 (m, 2H), 0.86 (t, *J* = 7.2 Hz, 3H); ^{13}C NMR (101 MHz, Chloroform-*d*) δ 144.8, 144.0, 138.0, 131.6, 129.2, 128.6, 128.4, 128.3, 127.0, 124.7, 50.7, 35.3, 30.2, 22.7, 21.5, 14.0; HRMS: m/z (EI) calculated $[M]^+$:272.1329, found: 272.1326. $[\alpha]_D^{25}$ = -15.937 (*c* = 0.25, $CHCl_3$).

The enantiomeric excess of **4c** was determined by chiral HPLC analysis compared to the corresponding racemate.

Conditions: ChiralPak IB column; hexane/^{i}PrOH = 100:0; flow rate = 0.5 mL/min; λ= 220 nm; t_{R1}(major)= 10.1 min; t_{R2}(minor)= 10.7 min.

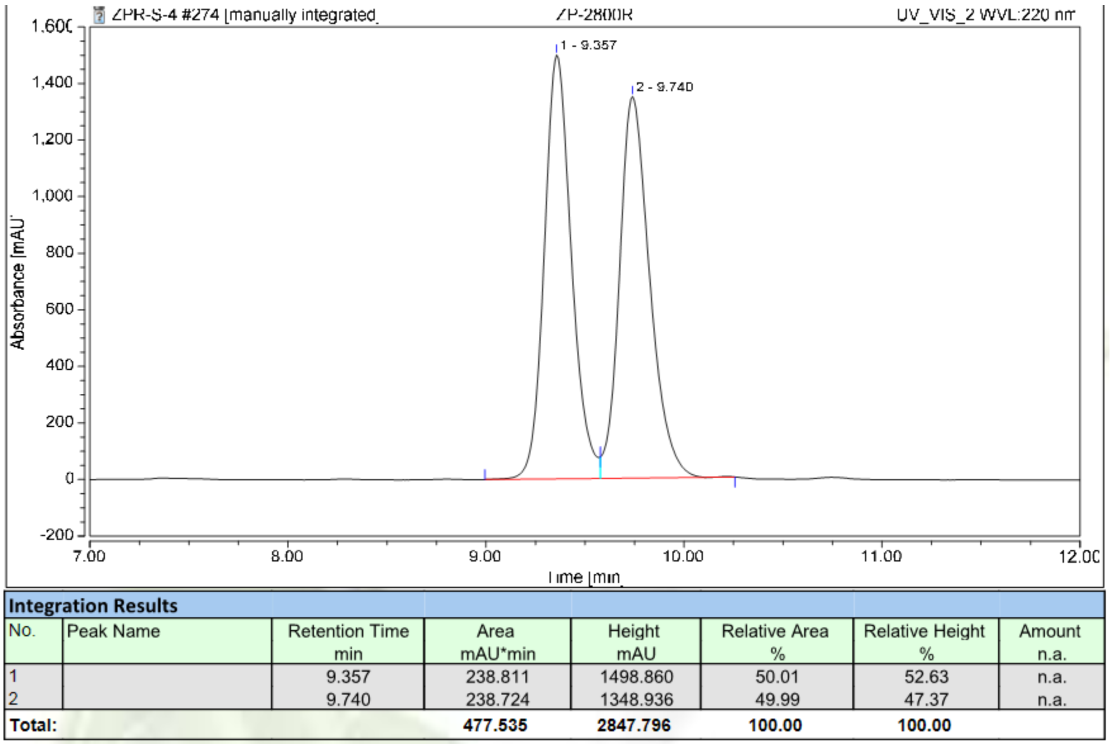

Integration Results							
No.	Peak Name	Retention Time min	Area mAU*min	Height mAU	Relative Area %	Relative Height %	Amount n.a.
1		9.357	238.811	1498.860	50.01	52.63	n.a.
2		9.740	238.724	1348.936	49.99	47.37	n.a.
**Total:**			**477.535**	**2847.796**	**100.00**	**100.00**	

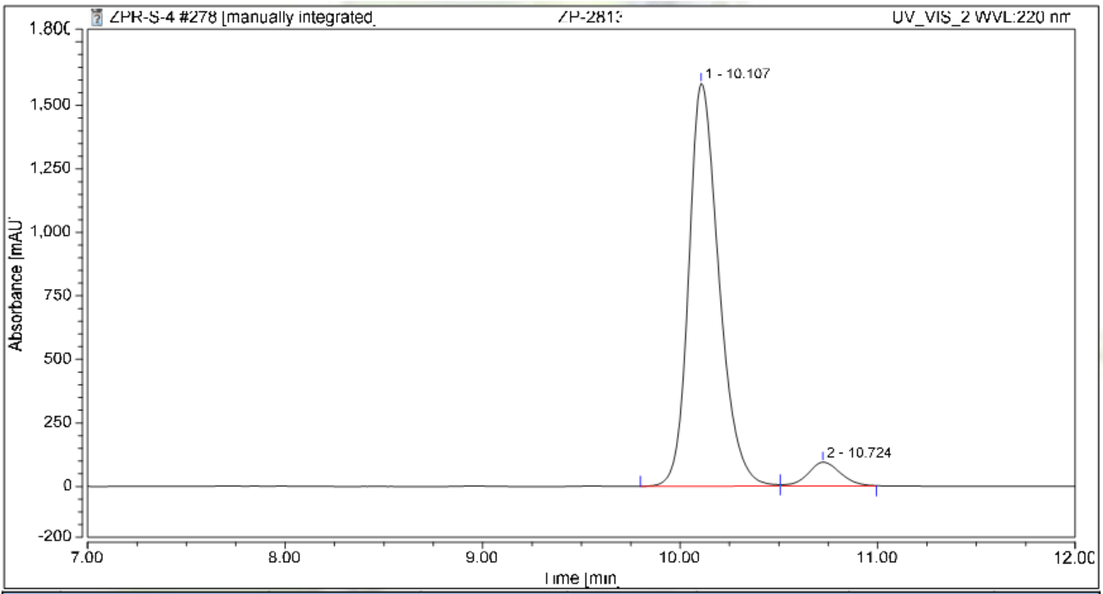

Integration Results

No.	Peak Name	Retention Time min	Area mAU*min	Height mAU	Relative Area %	Relative Height %	Amount n.a.
1		10.107	280.543	1585.653	94.08	94.44	n.a.
2		10.724	17.656	93.375	5.92	5.56	n.a.
**Total:**			**298.199**	**1679.028**	**100.00**	**100.00**	

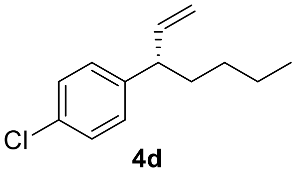

4d

The title compound **4d** was synthesized according to General Procedure E, and it was purified by column chromatography on silica gel (53% yield, 92% *ee*, 22.0 mg, colorless oil).

^{1}H NMR (400 MHz, Chloroform-*d*) δ 7.26 (d, *J* = 8.4 Hz, 2H), 7.11 (d, *J* = 8.4 Hz, 2H), 5.90 (ddd, *J* = 17.4, 10.4, 7.5 Hz, 1H), 5.06-4.94 (m, 2H), 3.26-3.15 (m, 1H), 1.73-1.61 (m, 2H), 1.34 -1.26 (m, 3H), 1.20-1.13 (m, 1H), 0.86 (t, *J* = 7.1 Hz, 3H); ^{13}C NMR (101 MHz, Chloroform-*d*) δ 143.1, 142.0, 131.7, 129.0, 128.5, 114.2, 49.2, 35.0, 29.6, 22.6, 14.0; ^{13}C NMR (101 MHz, Chloroform-*d*) δ 143.1, 142.0, 131.7, 129.0, 128.5, 114.2, 49.2, 35.0, 29.6, 22.6 , 14.0; $[\alpha]_D^{25}$ = +36.364 (*c* = 0.25, $CHCl_3$). The HNMR data were in accordance with those reported[1], and the optical rotation value of *S*-**4d** (97% *ee*) reported in reference [S8] is: $[\alpha]_D^{25}$ = +36.6 (*c* = 1.0, $CHCl_3$). The absolute configuration of **4d** is assumed to be *S*.
The enantiomeric excess of **4d** was determined by chiral HPLC analysis compared to the corresponding racemate the relative alcohol obtained after hydroboration of vinyl group and oxidation of the boryl group successively.
Conditions: ChiralPak IA column; hexane/^{i}PrOH = 99:1; flow rate = 1 mL/min; λ= 220 nm; t_{R1}(major)= 28.6 min; t_{R2}(minor)= 27.9 min.

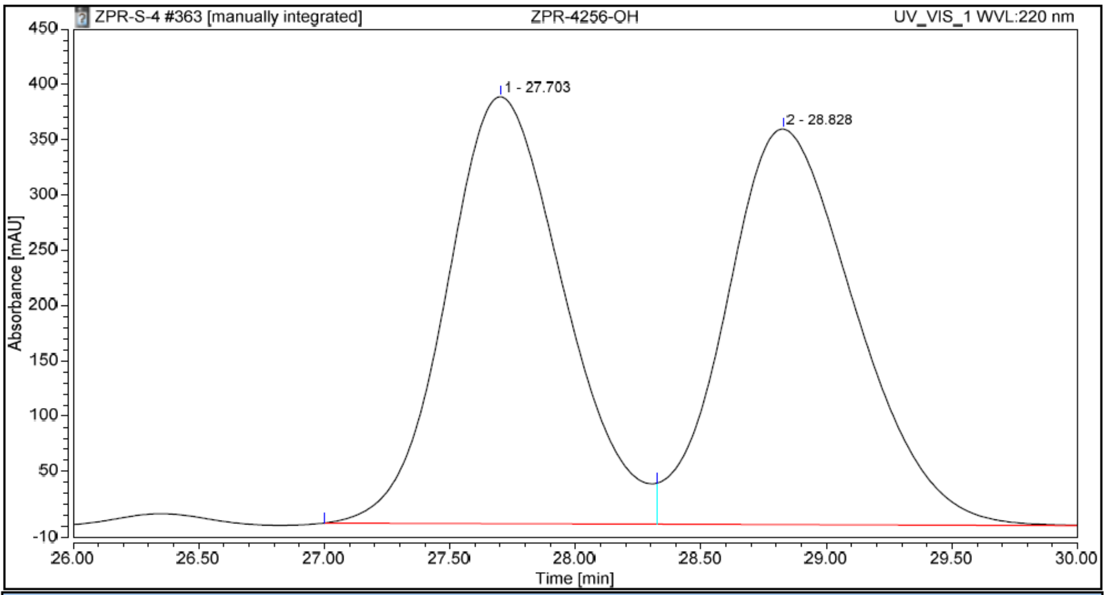

Integration Results

No.	Peak Name	Retention Time min	Area mAU*min	Height mAU	Relative Area %	Relative Height %	Amount n.a.
1		27.703	209.590	386.166	49.41	51.90	n.a.
2		28.828	214.630	357.913	50.59	48.10	n.a.
**Total:**			**424.220**	**744.079**	**100.00**	**100.00**	

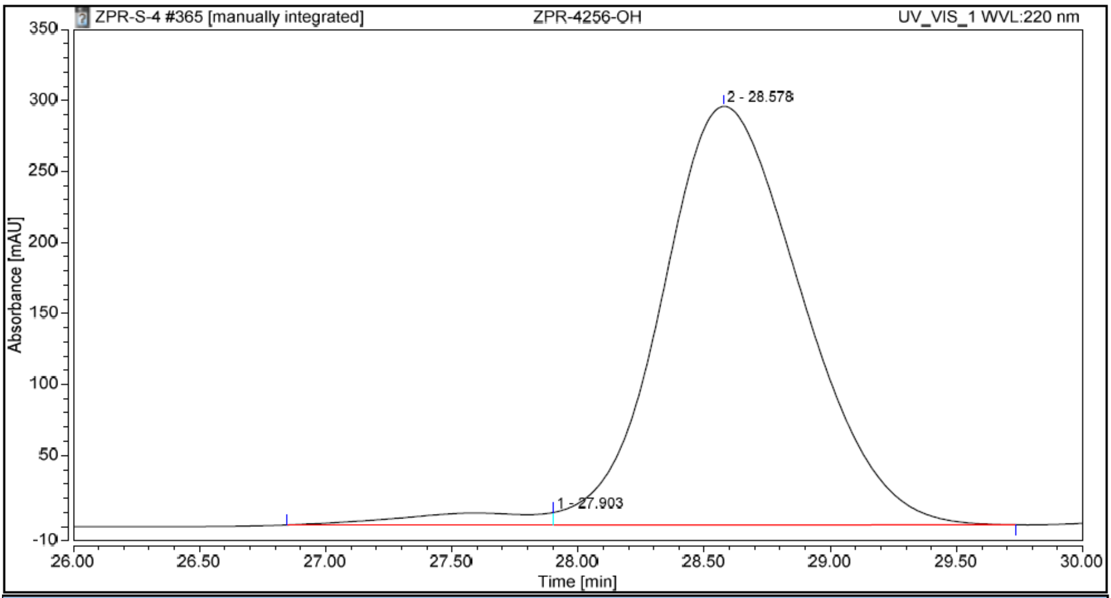

Integration Results

No.	Peak Name	Retention Time min	Area mAU*min	Height mAU	Relative Area %	Relative Height %	Amount n.a.
1		27.903	5.507	8.783	2.83	2.89	n.a.
2		28.578	189.352	294.794	97.17	97.11	n.a.
**Total:**			**194.859**	**303.577**	**100.00**	**100.00**	

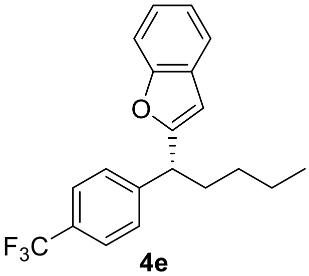

4e

The title compound **4e** was synthesized according to General Procedure (SI 4), and it was purified by column chromatography on silica gel (71% yield, 93% *ee*, 47.1 mg, colorless oil).

^{1}H NMR (400 MHz, Chloroform-*d*) δ 7.57 (d, *J* = 8.0 Hz, 2H), 7.53-7.48 (m, 1H), 7.47-7.37 (m, 3H), 7.24-7.15 (m, 2H), 6.48 (s, 1H), 4.10 (d, *J* = 7.6, 7.8 Hz, 1H), 2.30 -2.17 (m, 1H), 2.05-1.90 (m, 1H), 1.44-1.27 (m, 4H), 0.88 (t, *J* = 7.0 Hz, 3H); ^{13}C NMR (101 MHz, Chloroform-*d*) δ 160.1, 154.8, 146.2, 129.0 (q, *J* = 32.2 Hz), 128.4, 128.3, 125.5 (q, *J* = 4.5, 3.8 Hz), 123.6, 122.6, 124.2 (q, *J* = 272.0 Hz), 120.5, 111.0, 102.7, 45.6, 34.0, 29.7, 22.5, 13.9; HRMS: m/z (EI) calculated $[M]^+$:332.1383, found: 332.1389. $[\alpha]_D^{25}$ = -19.205 (*c* = 0.25, $CHCl_3$).
The enantiomeric excess of **4e** was determined by chiral HPLC analysis compared to the corresponding racemate.
Conditions: ChiralPak IB column; hexane/^{i}PrOH = 100:0; flow rate = 0.5 mL/min; λ= 220 nm; t_{R1}(major)= 15.7 min; t_{R2}(minor)= 11.8 min.

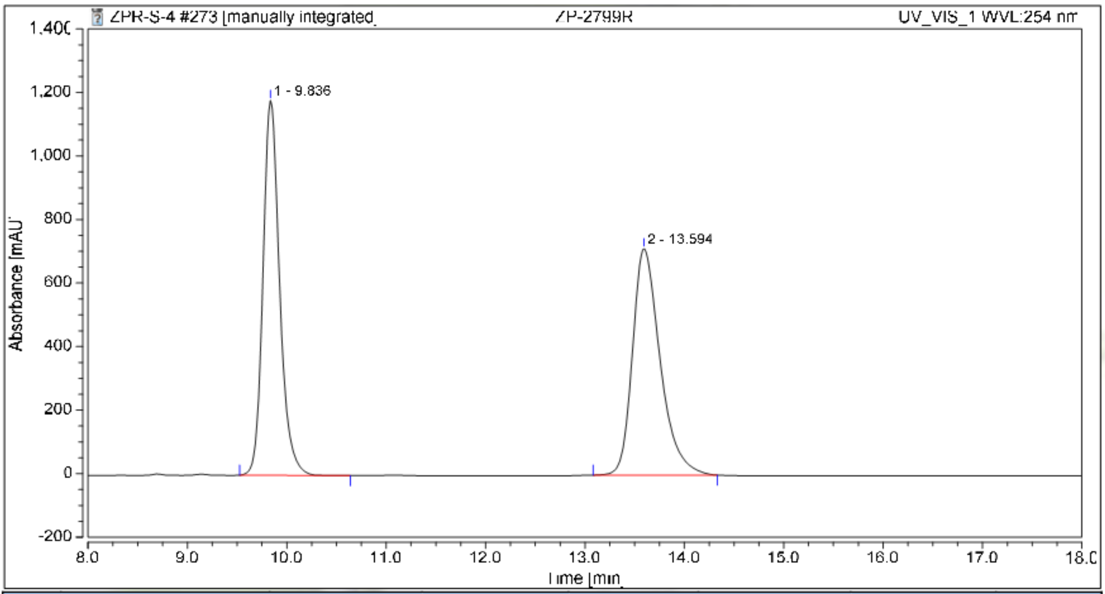

Integration Results							
No.	Peak Name	Retention Time min	Area mAU*min	Height mAU	Relative Area %	Relative Height %	Amount n.a.
1		9.836	228.812	1180.332	49.90	62.34	n.a.
2		13.594	229.686	712.920	50.10	37.66	n.a.
**Total:**			**458.498**	**1893.252**	**100.00**	**100.00**	

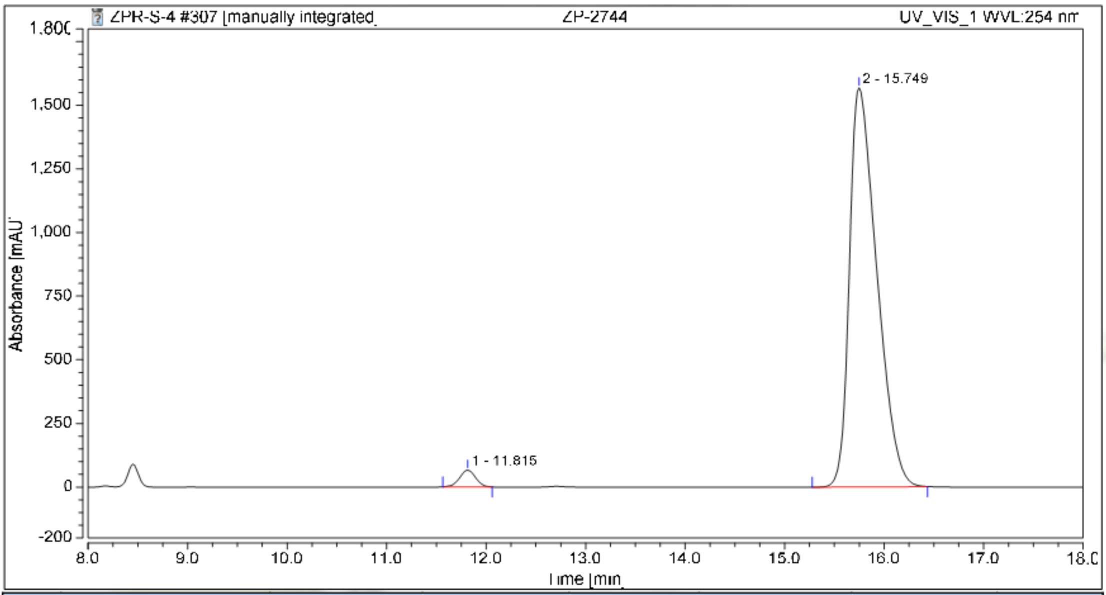

Integration Results							
No.	Peak Name	Retention Time min	Area mAU*min	Height mAU	Relative Area %	Relative Height %	Amount n.a.
1		11.815	12.307	66.181	2.40	4.05	n.a.
2		15.749	499.709	1568.306	97.60	95.95	n.a.
**Total:**			**512.016**	**1634.486**	**100.00**	**100.00**	

2.5 Spectra data of the compounds

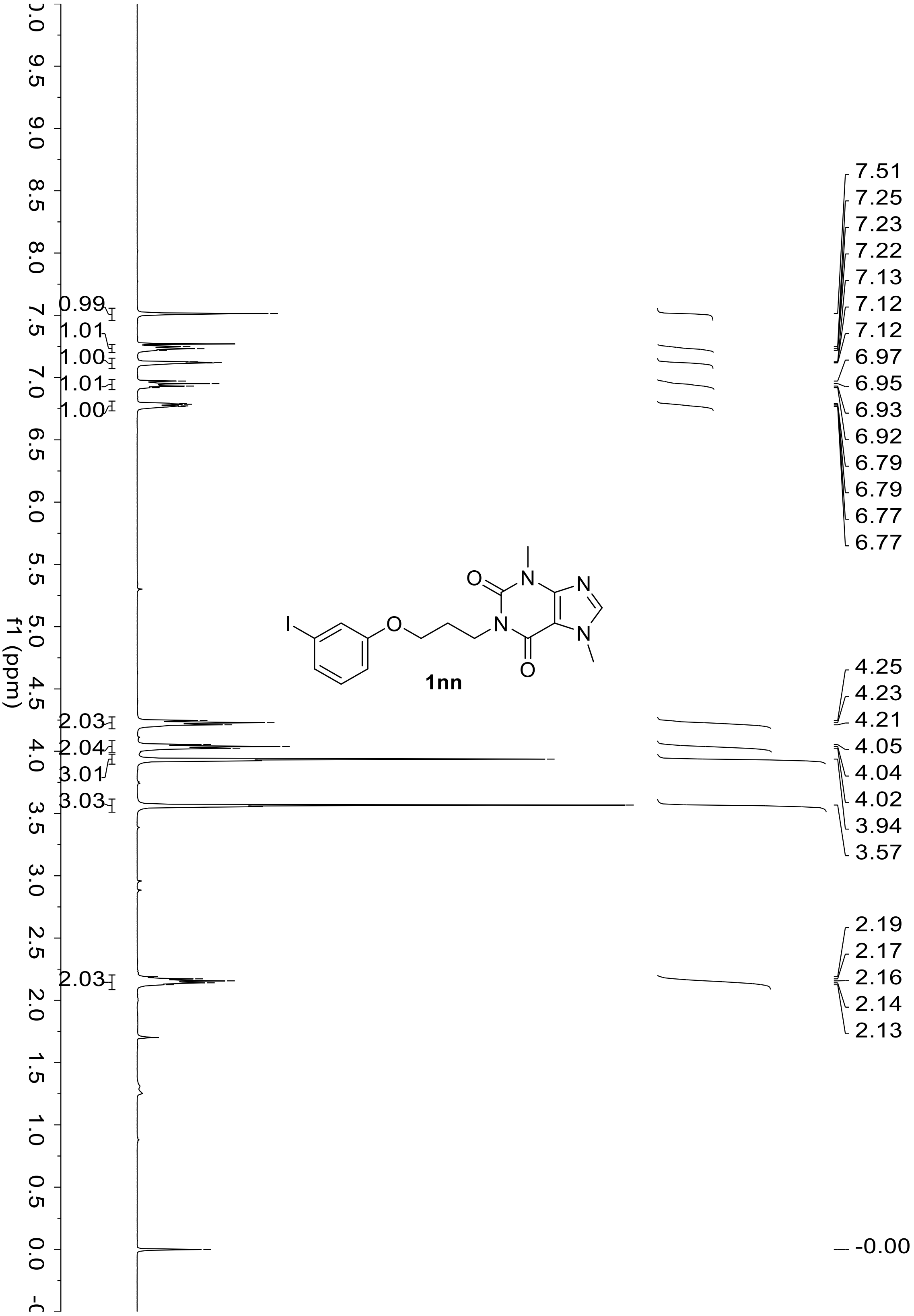

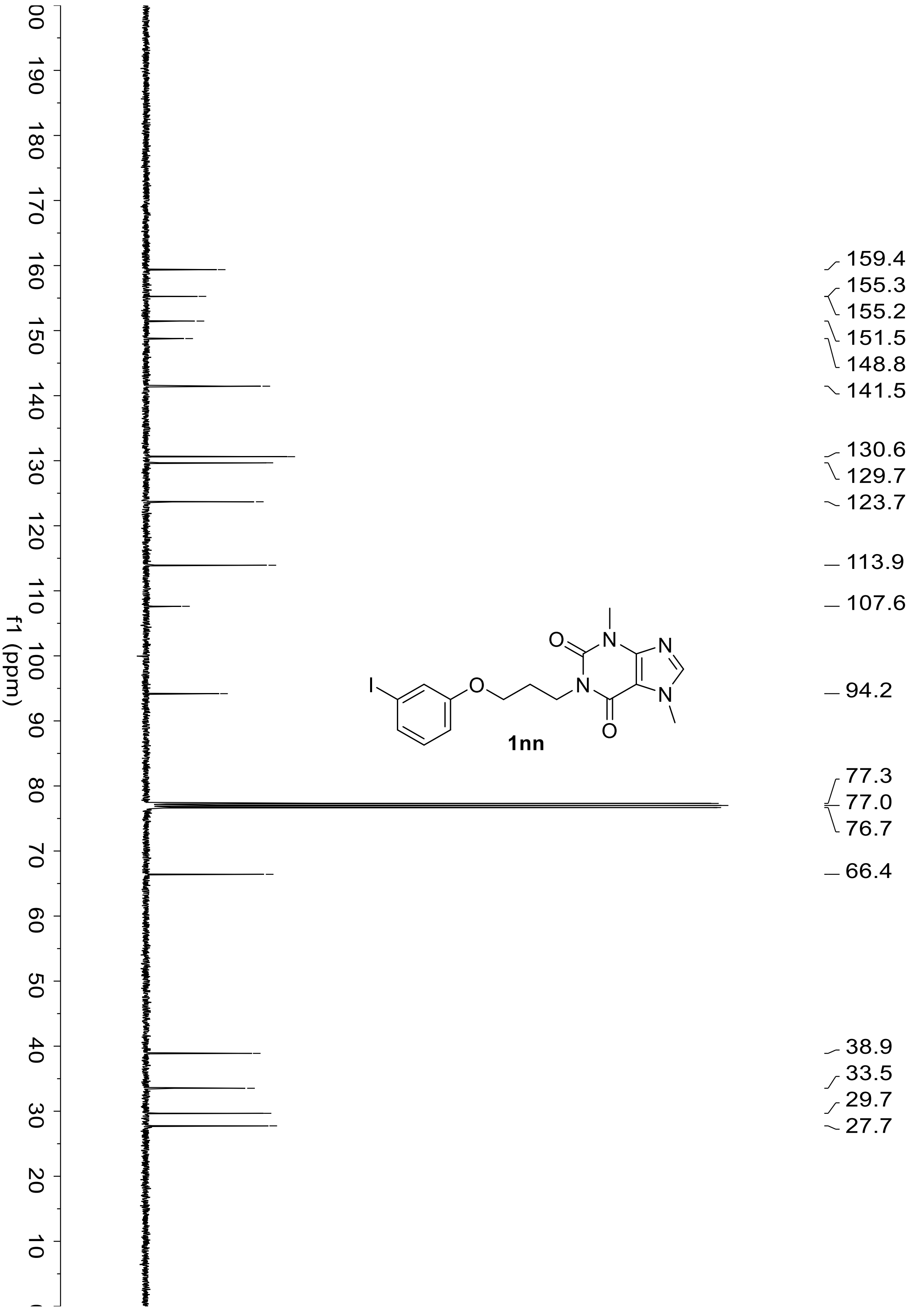
159.4
155.3
155.2
151.5
148.8
141.5
130.6
129.7
123.7
113.9
107.6
94.2
77.3
77.0
76.7
66.4
38.9
33.5
29.7
27.7
1nn
f1 (ppm)

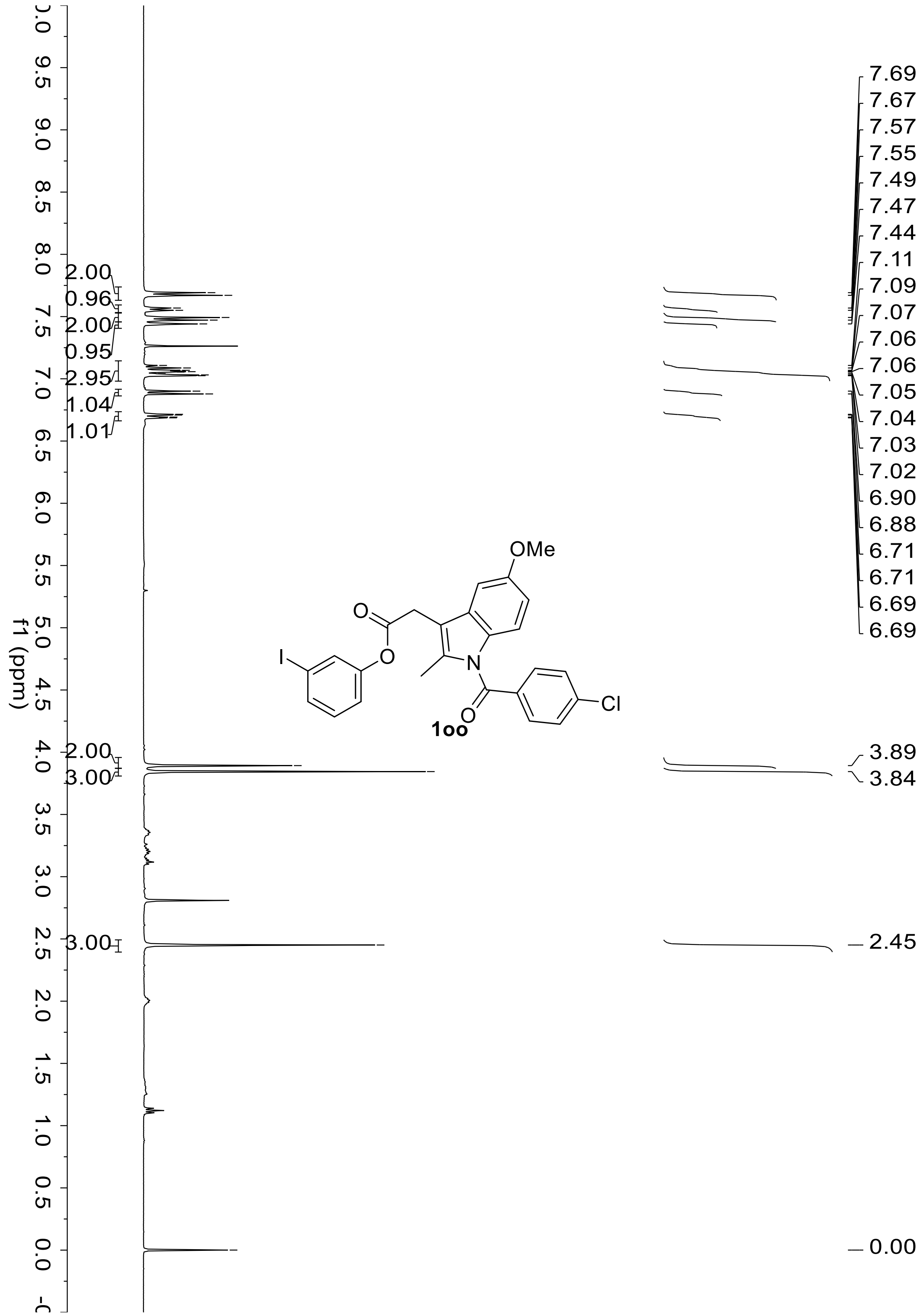
7.69
7.67
7.57
7.55
7.49
7.47
7.44
7.11
7.09
7.07
7.06
7.06
7.05
7.04
7.03
7.02
6.90
6.88
6.71
6.71
6.69
6.69
3.89
3.84
2.45
0.00
2.00
0.96
2.00
0.95
2.95
1.04
1.01
2.00
3.00
3.00
f1 (ppm)
OMe
O
O
I
N
O
Cl
1oo

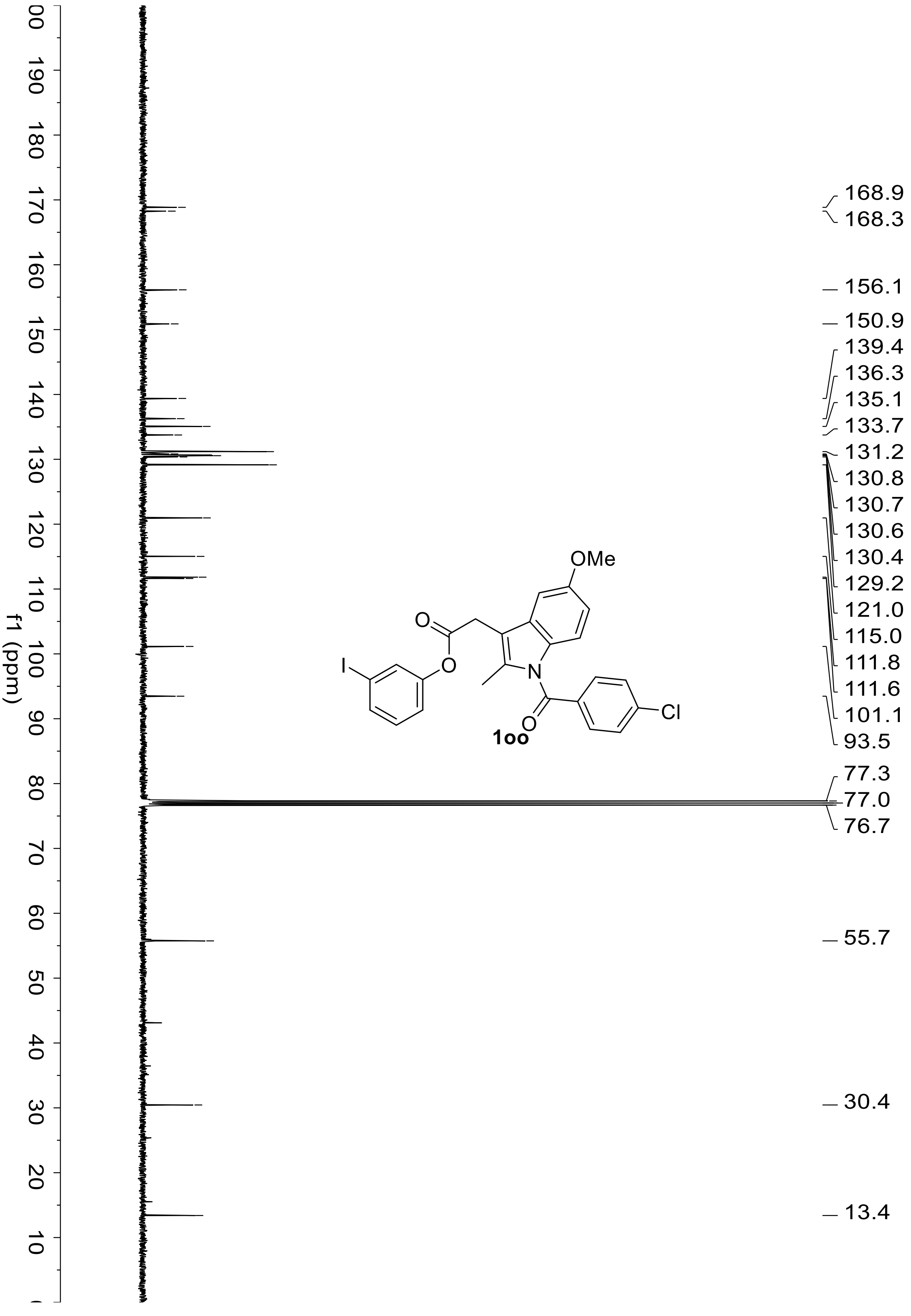
168.9
168.3
156.1
150.9
139.4
136.3
135.1
133.7
131.2
130.8
130.7
130.6
130.4
129.2
121.0
115.0
111.8
111.6
101.1
93.5
77.3
77.0
76.7
55.7
30.4
13.4
OMe
O
O
I
N
O
Cl
1oo
f1 (ppm)

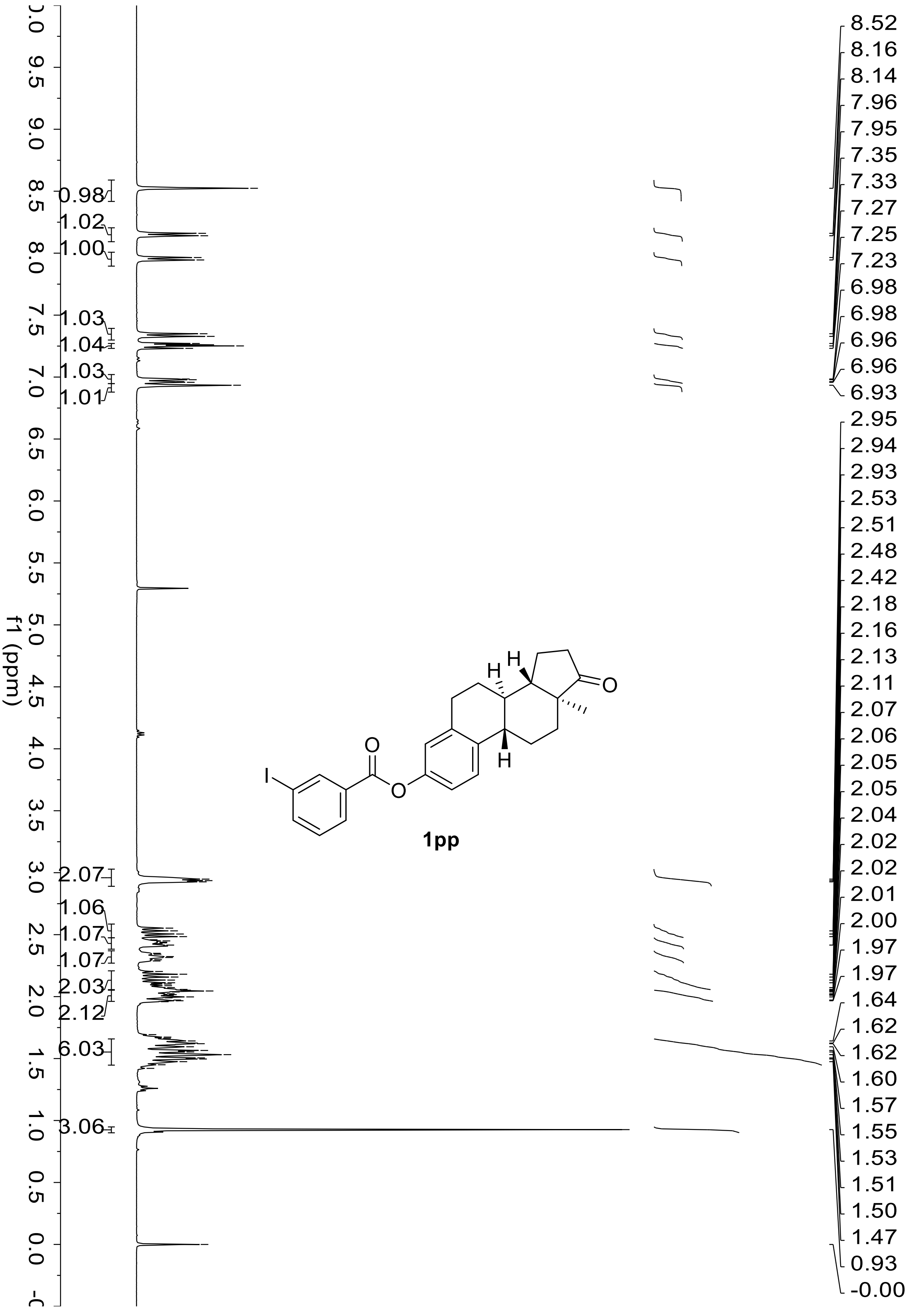
8.52
8.16
8.14
7.96
7.95
7.35
7.33
7.27
7.25
7.23
6.98
6.98
6.96
6.96
6.93
2.95
2.94
2.93
2.53
2.51
2.48
2.42
2.18
2.16
2.13
2.11
2.07
2.06
2.05
2.05
2.04
2.02
2.02
2.01
2.00
1.97
1.97
1.64
1.62
1.62
1.60
1.57
1.55
1.53
1.51
1.50
1.47
0.93
-0.00
0.98
1.02
1.00
1.03
1.04
1.03
1.01
2.07
1.06
1.07
1.07
2.03
2.12
6.03
3.06
f1 (ppm)
1pp

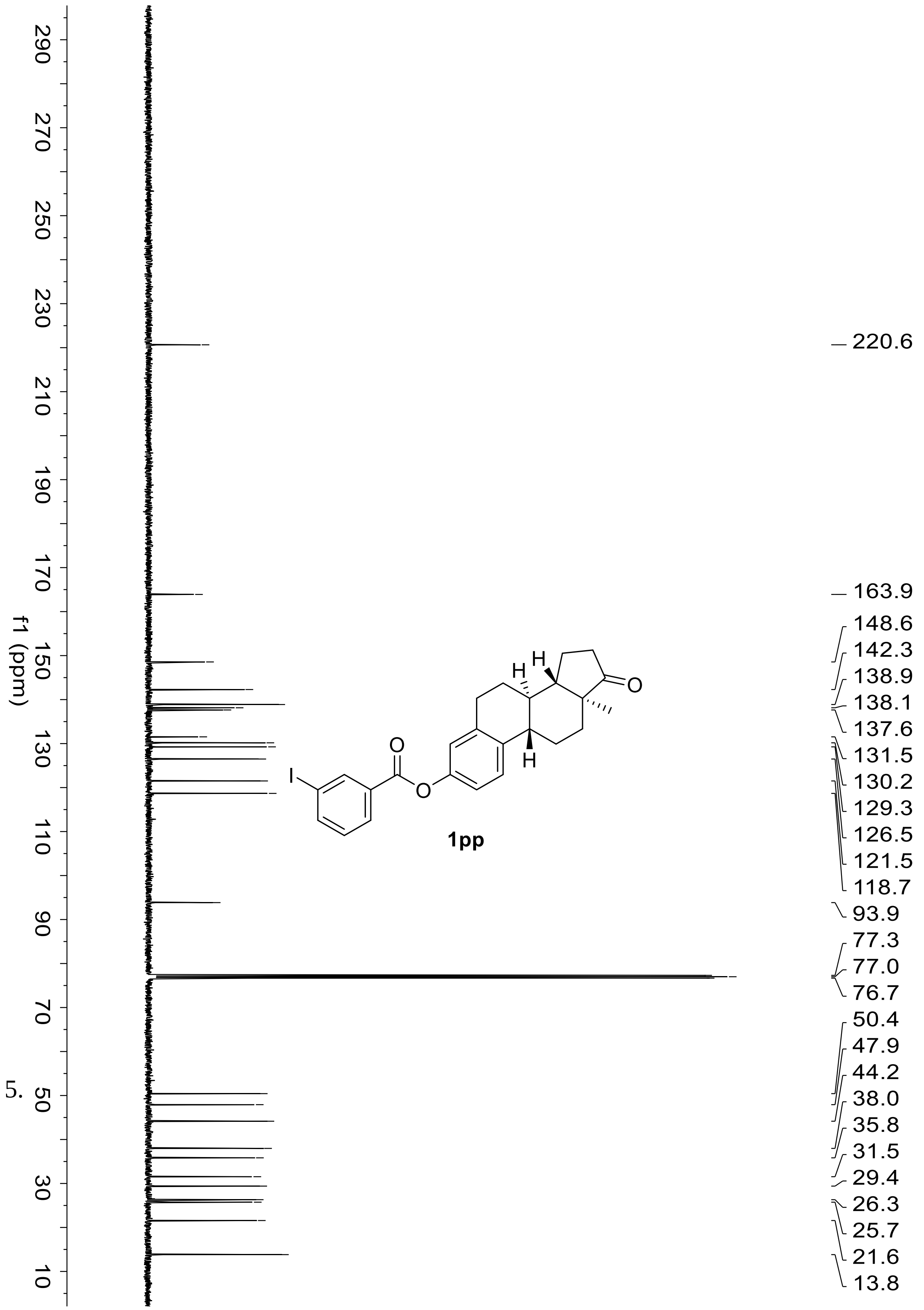
220.6
163.9
148.6
142.3
138.9
138.1
137.6
131.5
130.2
129.3
126.5
121.5
118.7
93.9
77.3
77.0
76.7
50.4
47.9
44.2
38.0
35.8
31.5
29.4
26.3
25.7
21.6
13.8
f1 (ppm)

1pp

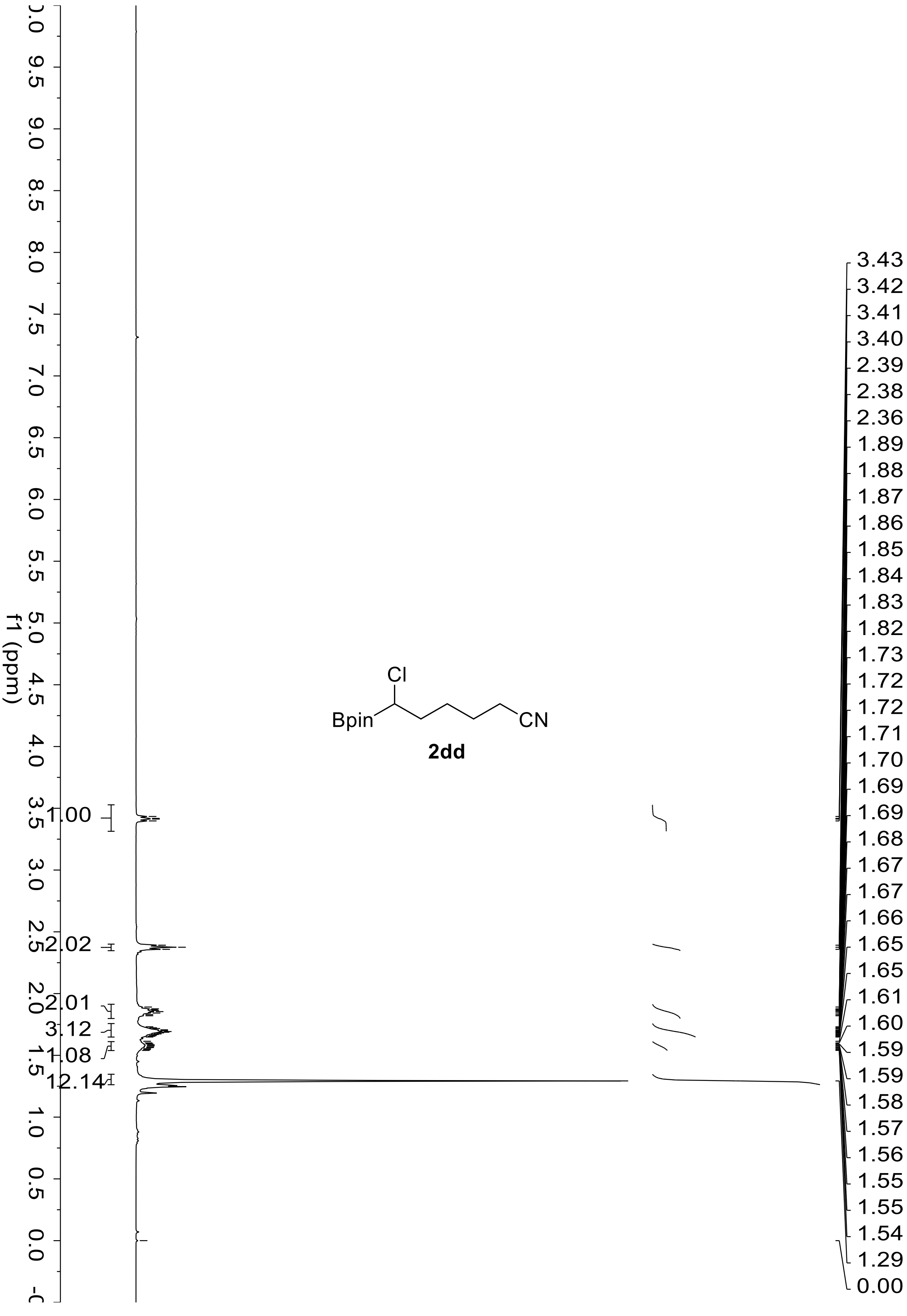

3.43
3.42
3.41
3.40
2.39
2.38
2.36
1.89
1.88
1.87
1.86
1.85
1.84
1.83
1.82
1.73
1.72
1.72
1.71
1.70
1.69
1.69
1.68
1.67
1.67
1.66
1.65
1.65
1.61
1.60
1.59
1.59
1.58
1.57
1.56
1.55
1.55
1.54
1.29
0.00
Cl
Bpin
CN
2dd
1.00
2.02
2.01
3.12
1.08
12.14
f1 (ppm)

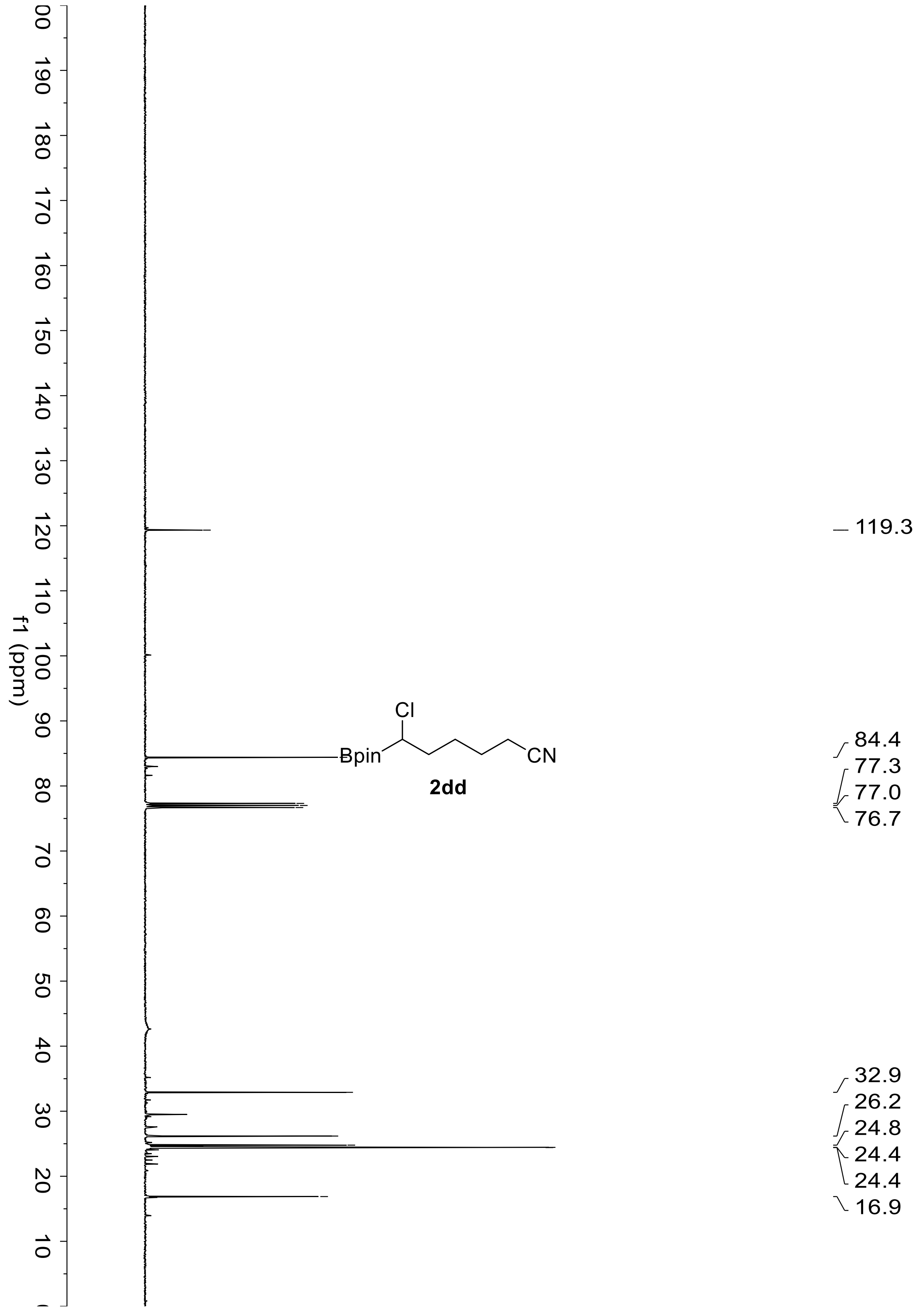
119.3
84.4
77.3
77.0
76.7
32.9
26.2
24.8
24.4
24.4
16.9
f1 (ppm)
Cl
Bpin
CN
2dd

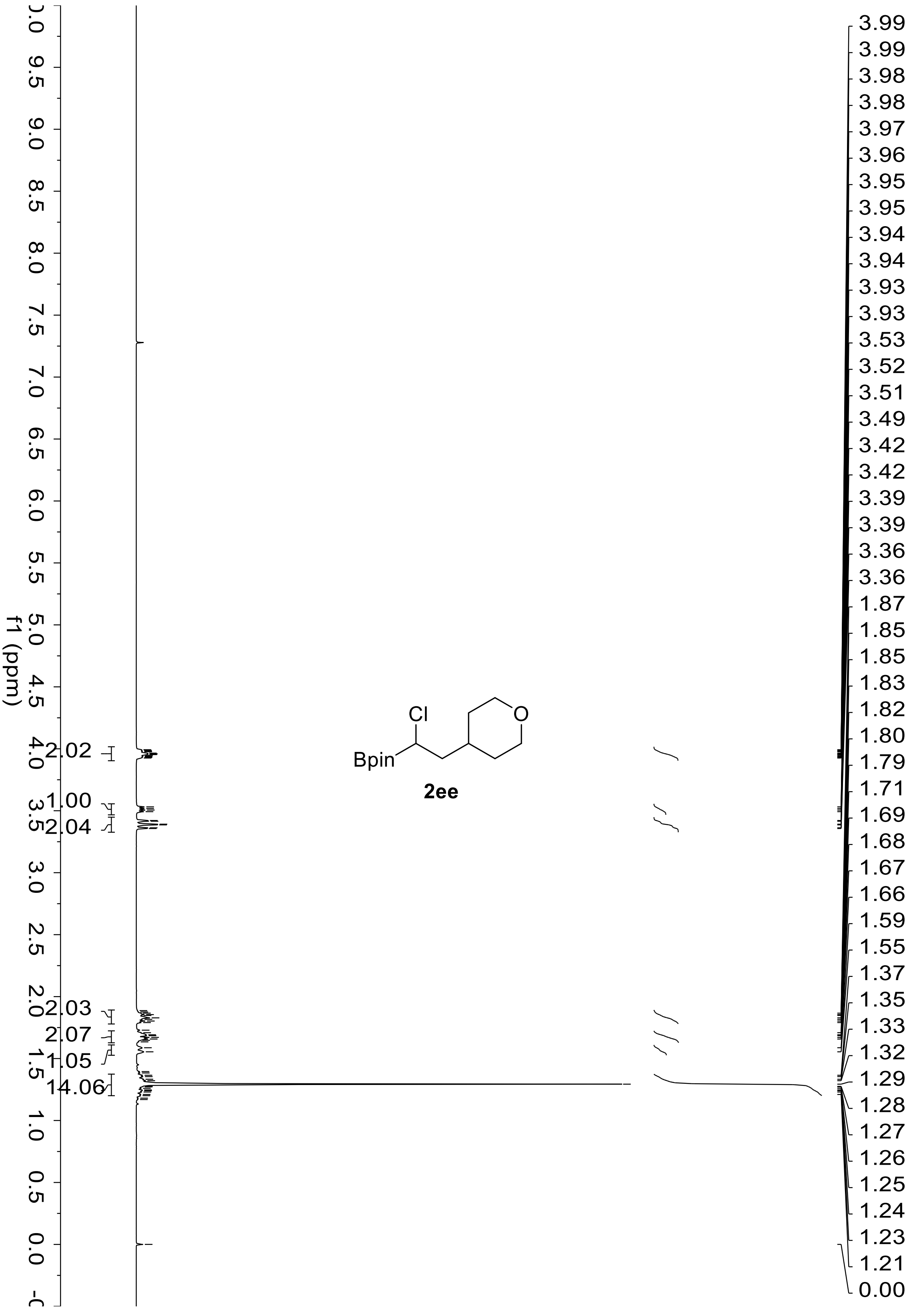
Cl
O
Bpin
2ee
f1 (ppm)
2.02
1.00
2.04
2.03
2.07
1.05
14.06
3.99
3.99
3.98
3.98
3.97
3.96
3.95
3.95
3.94
3.94
3.93
3.93
3.53
3.52
3.51
3.49
3.42
3.42
3.39
3.39
3.36
3.36
1.87
1.85
1.85
1.83
1.82
1.80
1.79
1.71
1.69
1.68
1.67
1.66
1.59
1.55
1.37
1.35
1.33
1.32
1.29
1.28
1.27
1.26
1.25
1.24
1.23
1.21
0.00

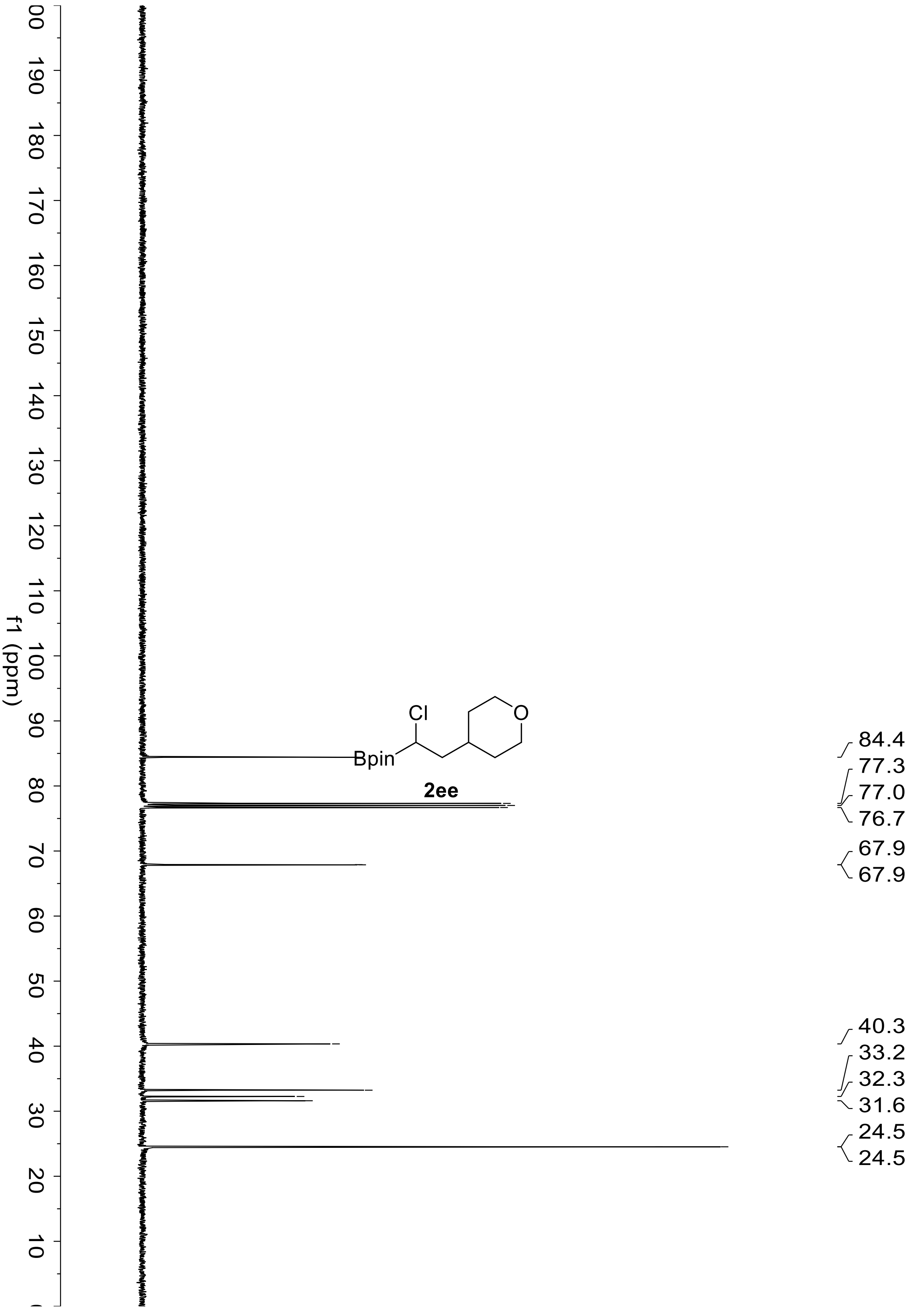
Cl
O
Bpin
2ee
84.4
77.3
77.0
76.7
67.9
67.9
40.3
33.2
32.3
31.6
24.5
24.5
f1 (ppm)

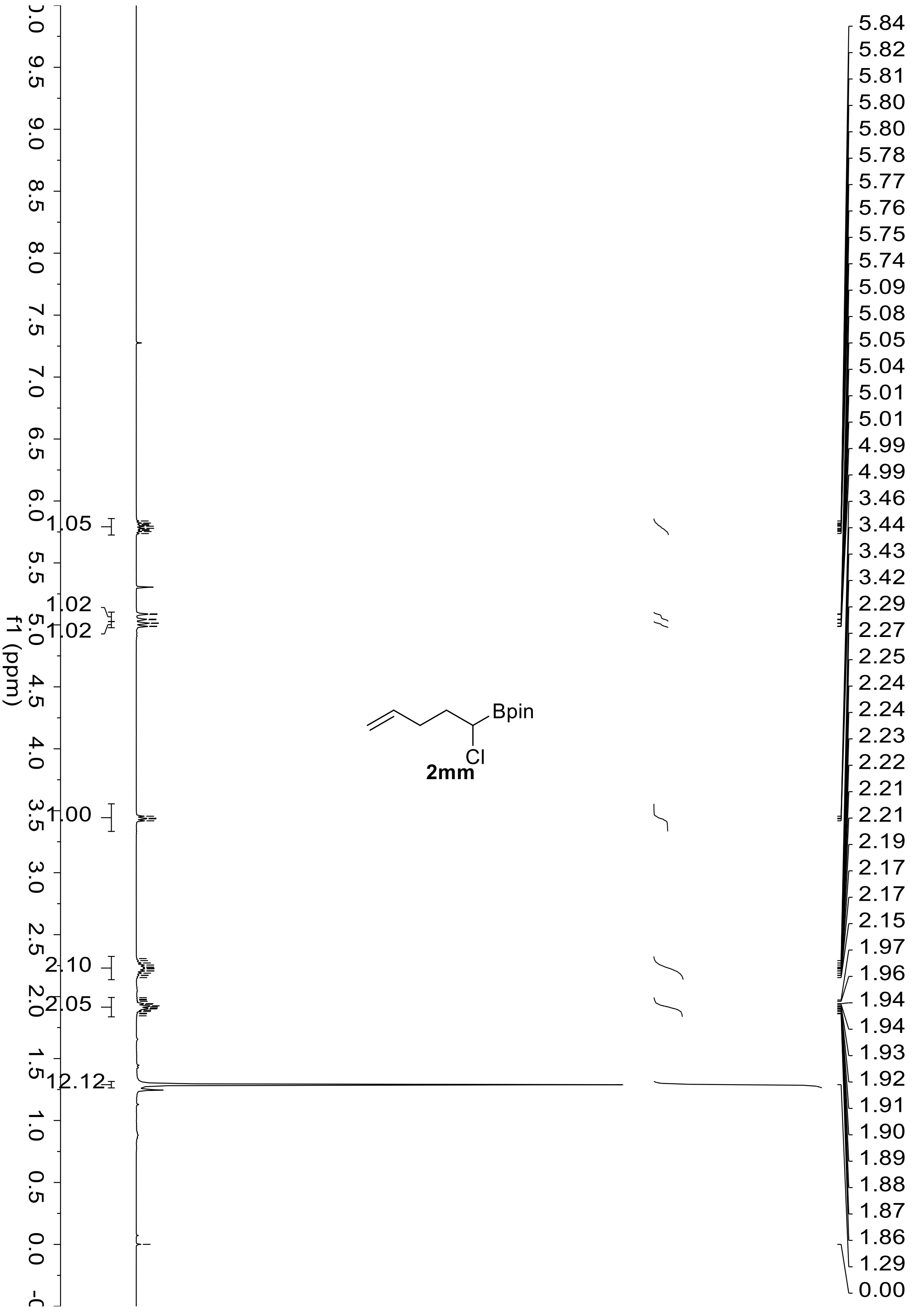
5.84
5.82
5.81
5.80
5.80
5.78
5.77
5.76
5.75
5.74
5.09
5.08
5.05
5.04
5.01
5.01
4.99
4.99
3.46
3.44
3.43
3.42
2.29
2.27
2.25
2.24
2.24
2.23
2.22
2.21
2.21
2.19
2.17
2.17
2.15
1.97
1.96
1.94
1.94
1.93
1.92
1.91
1.90
1.89
1.88
1.87
1.86
1.29
0.00
Bpin
Cl
2mm
1.05
1.02
1.02
1.00
2.10
2.05
12.12
f1 (ppm)

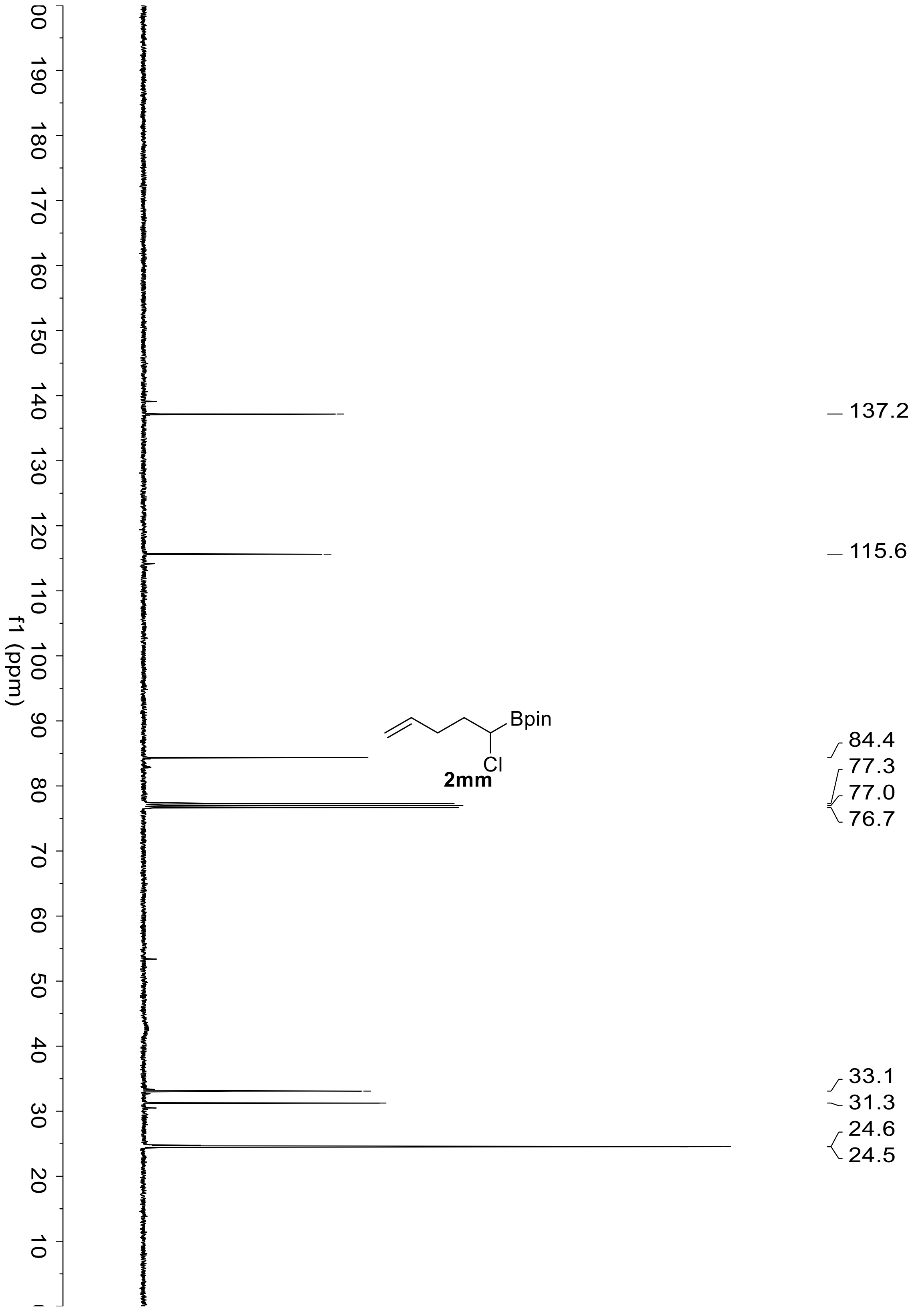

137.2
115.6
84.4
77.3
77.0
76.7
33.1
31.3
24.6
24.5
Bpin
Cl
2mm
f1 (ppm)

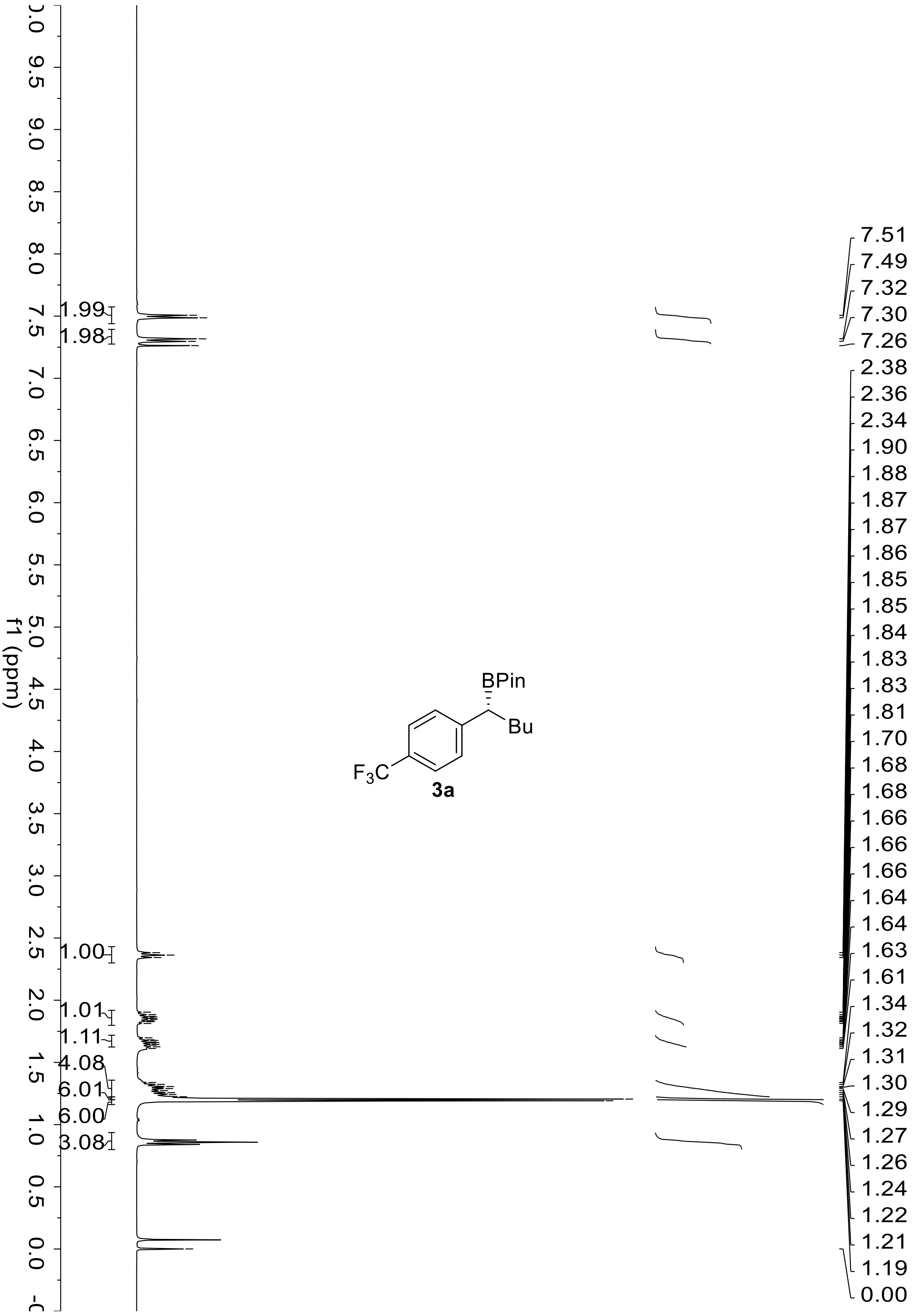
BPin
Bu
F3C
3a
f1 (ppm)
7.51
7.49
7.32
7.30
7.26
2.38
2.36
2.34
1.90
1.88
1.87
1.87
1.86
1.85
1.85
1.84
1.83
1.83
1.81
1.70
1.68
1.68
1.66
1.66
1.66
1.64
1.64
1.63
1.61
1.34
1.32
1.31
1.30
1.29
1.27
1.26
1.24
1.22
1.21
1.19
0.00
1.99
1.98
1.00
1.01
1.11
4.08
6.01
6.00
3.08

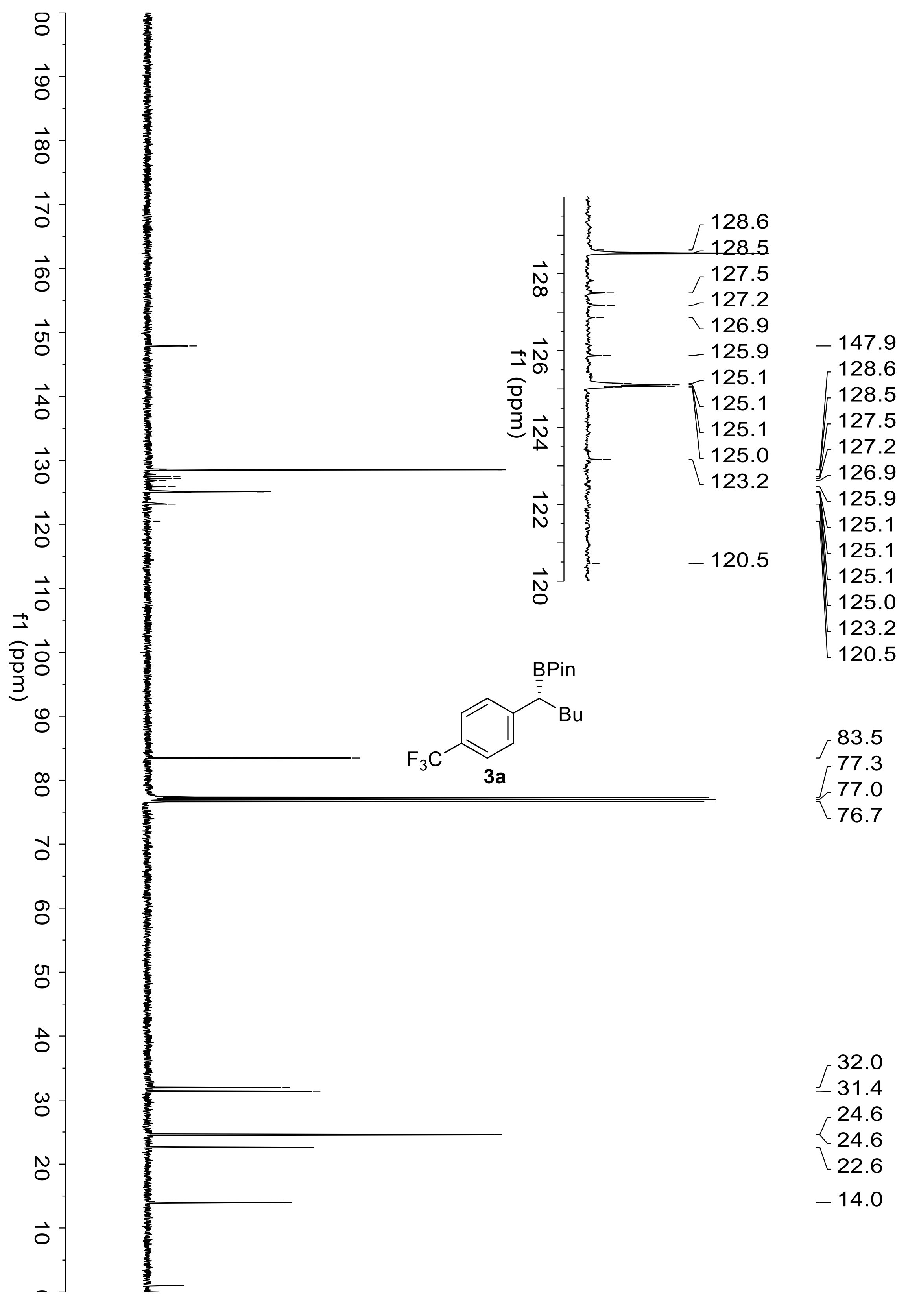
f1 (ppm)
BPin
Bu
F3C
3a
128.6
128.5
127.5
127.2
126.9
125.9
125.1
125.1
125.1
125.0
123.2
120.5
147.9
83.5
77.3
77.0
76.7
32.0
31.4
24.6
24.6
22.6
14.0

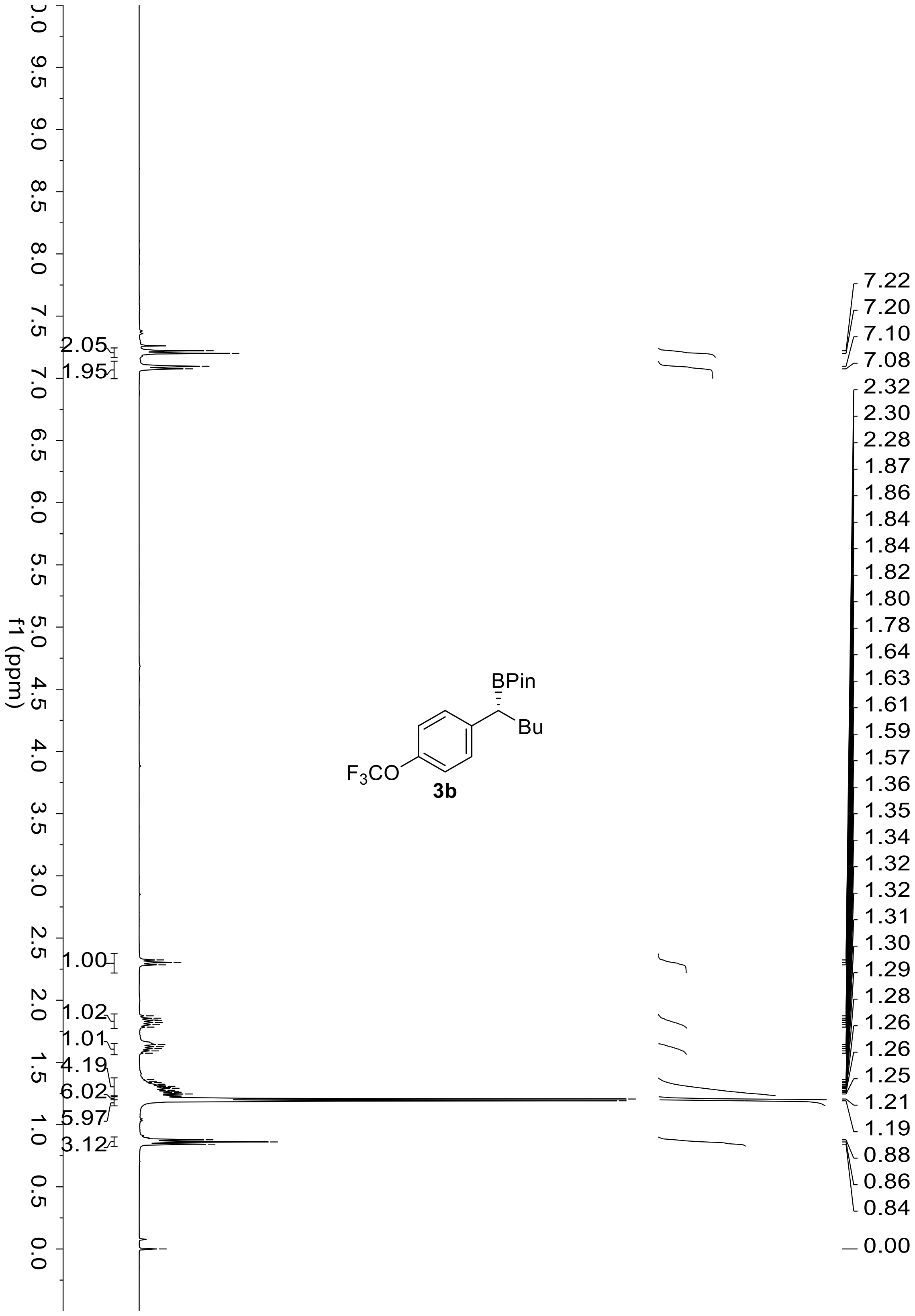
7.22
7.20
7.10
7.08
2.32
2.30
2.28
1.87
1.86
1.84
1.84
1.82
1.80
1.78
1.64
1.63
1.61
1.59
1.57
1.36
1.35
1.34
1.32
1.32
1.31
1.30
1.29
1.28
1.26
1.26
1.25
1.21
1.19
0.88
0.86
0.84
0.00
2.05
1.95
1.00
1.02
1.01
4.19
6.02
5.97
3.12
f1 (ppm)
BPin
Bu
F3CO
3b

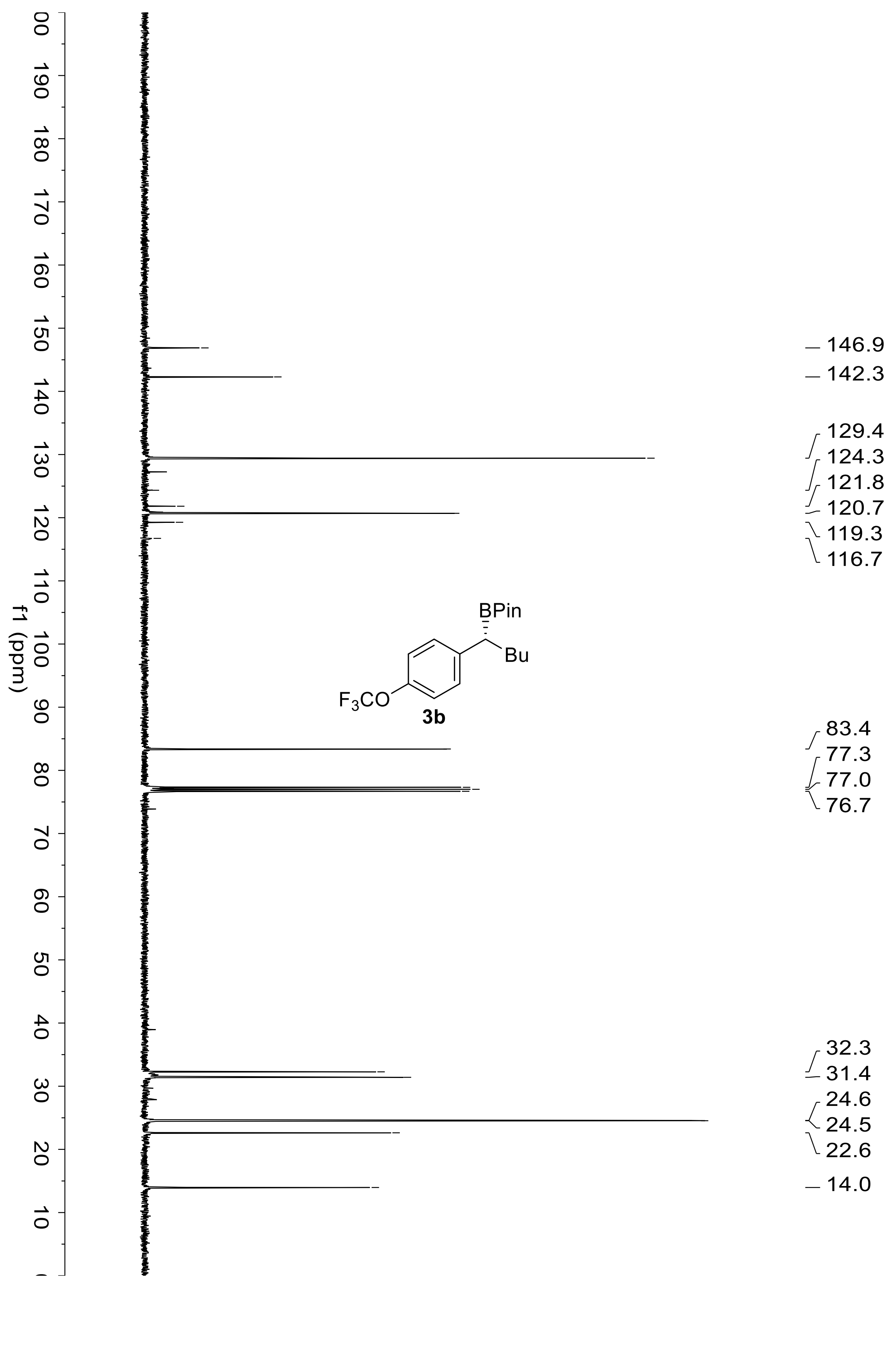
146.9
142.3
129.4
124.3
121.8
120.7
119.3
116.7
BPin
Bu
F_3CO
3b
83.4
77.3
77.0
76.7
32.3
31.4
24.6
24.5
22.6
14.0
f1 (ppm)

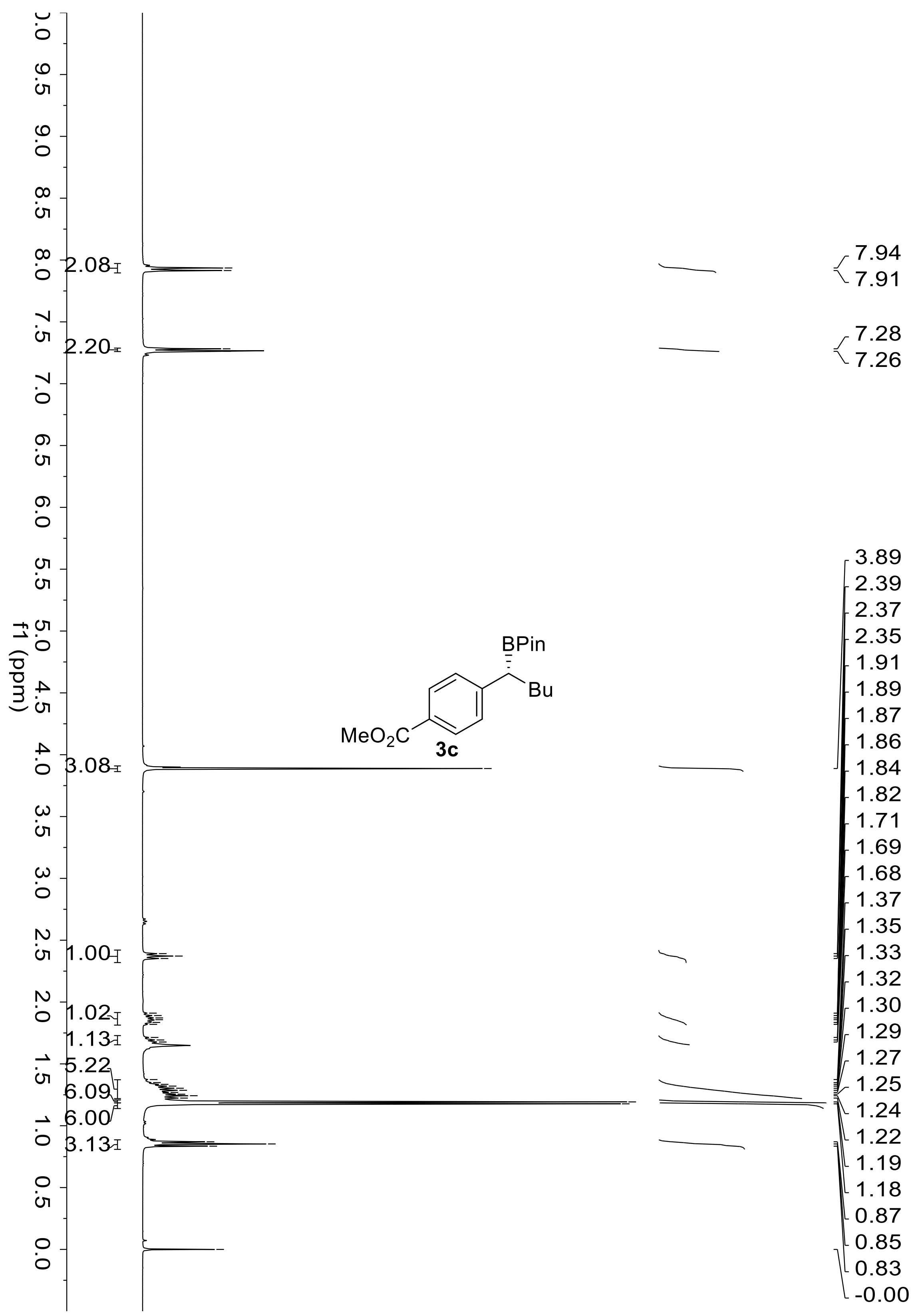

BPin
Bu
MeO2C
3c
f1 (ppm)
2.08
2.20
3.08
1.00
1.02
1.13
5.22
6.09
6.00
3.13
7.94
7.91
7.28
7.26
3.89
2.39
2.37
2.35
1.91
1.89
1.87
1.86
1.84
1.82
1.71
1.69
1.68
1.37
1.35
1.33
1.32
1.30
1.29
1.27
1.25
1.24
1.22
1.19
1.18
0.87
0.85
0.83
-0.00

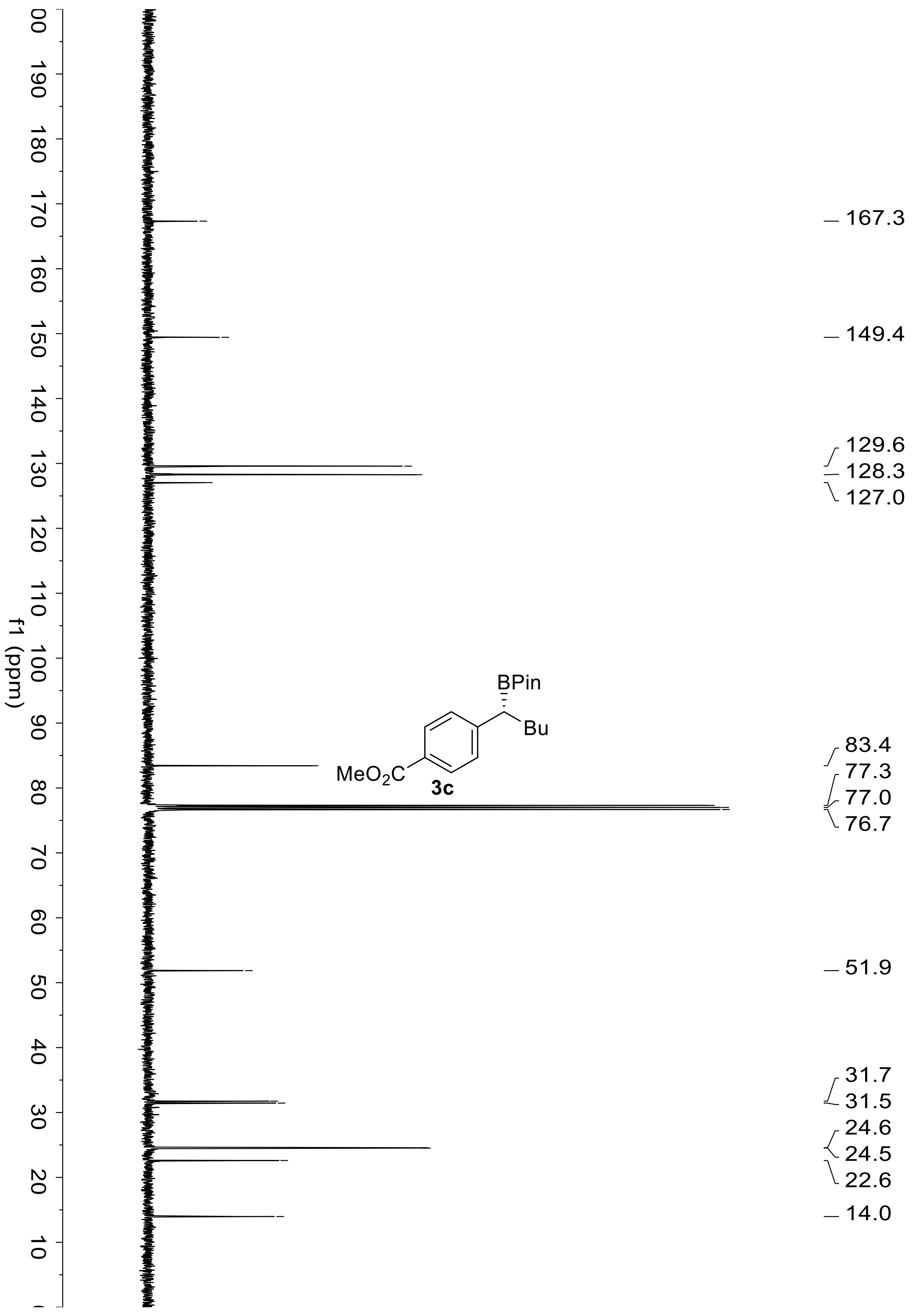
167.3
149.4
129.6
128.3
127.0
83.4
77.3
77.0
76.7
51.9
31.7
31.5
24.6
24.5
22.6
14.0
BPin
Bu
MeO2C
3c
f1 (ppm)

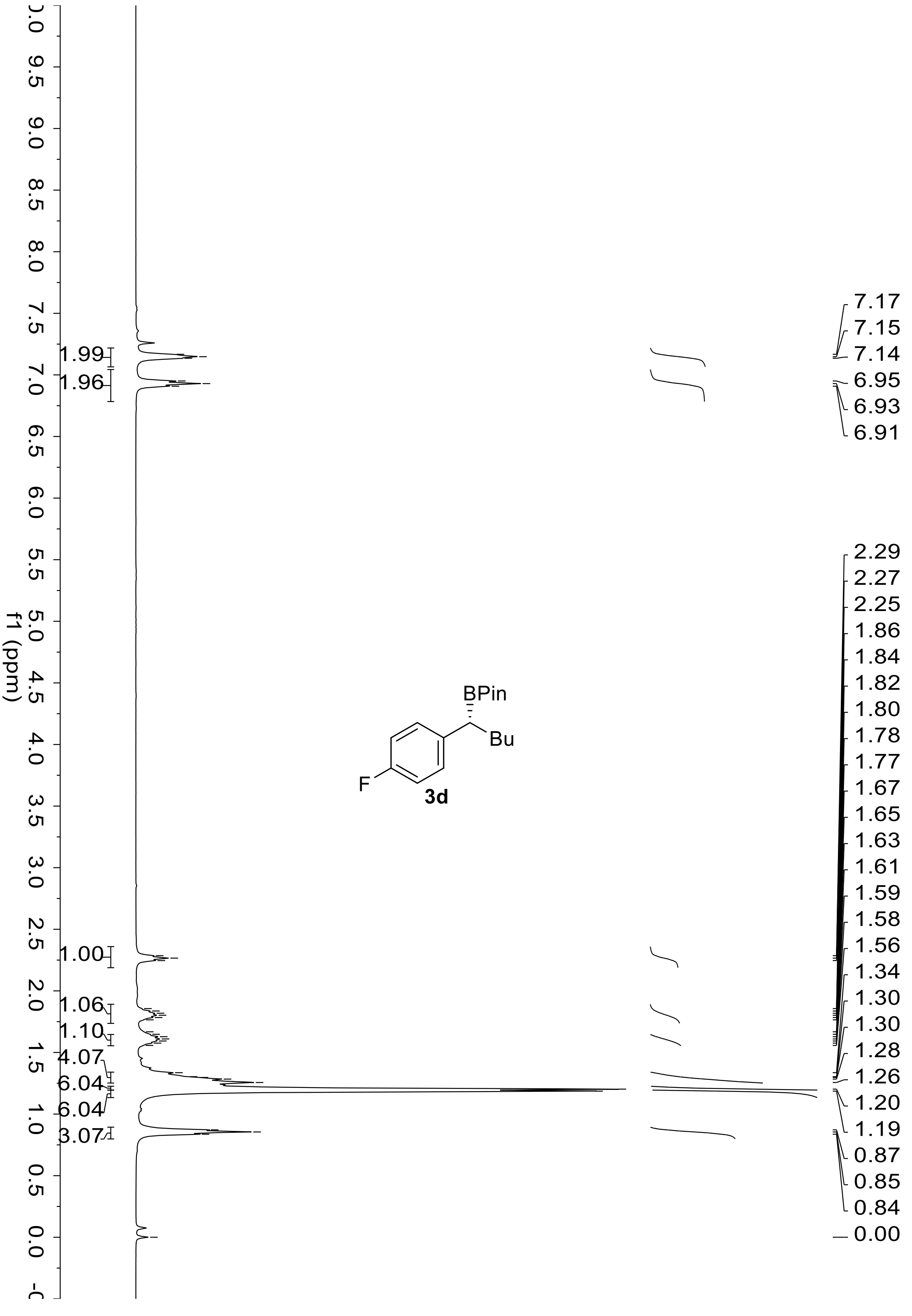
BPin
Bu
F
3d
7.17
7.15
7.14
6.95
6.93
6.91
2.29
2.27
2.25
1.86
1.84
1.82
1.80
1.78
1.77
1.67
1.65
1.63
1.61
1.59
1.58
1.56
1.34
1.30
1.30
1.28
1.26
1.20
1.19
0.87
0.85
0.84
0.00
1.99
1.96
1.00
1.06
1.10
4.07
6.04
6.04
3.07
f1 (ppm)

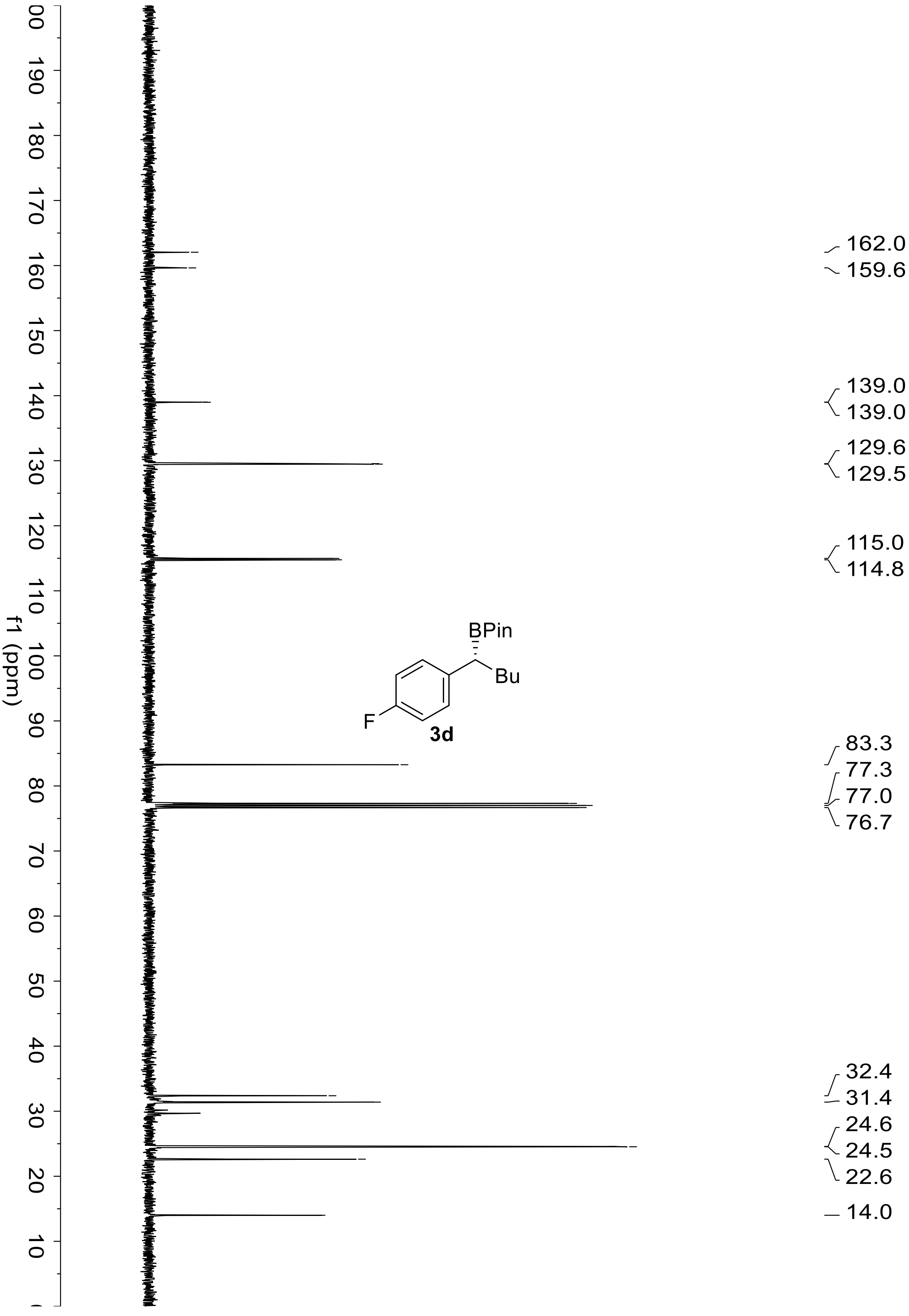

162.0
159.6
139.0
139.0
129.6
129.5
115.0
114.8
83.3
77.3
77.0
76.7
32.4
31.4
24.6
24.5
22.6
14.0
BPin
Bu
F
3d
f1 (ppm)

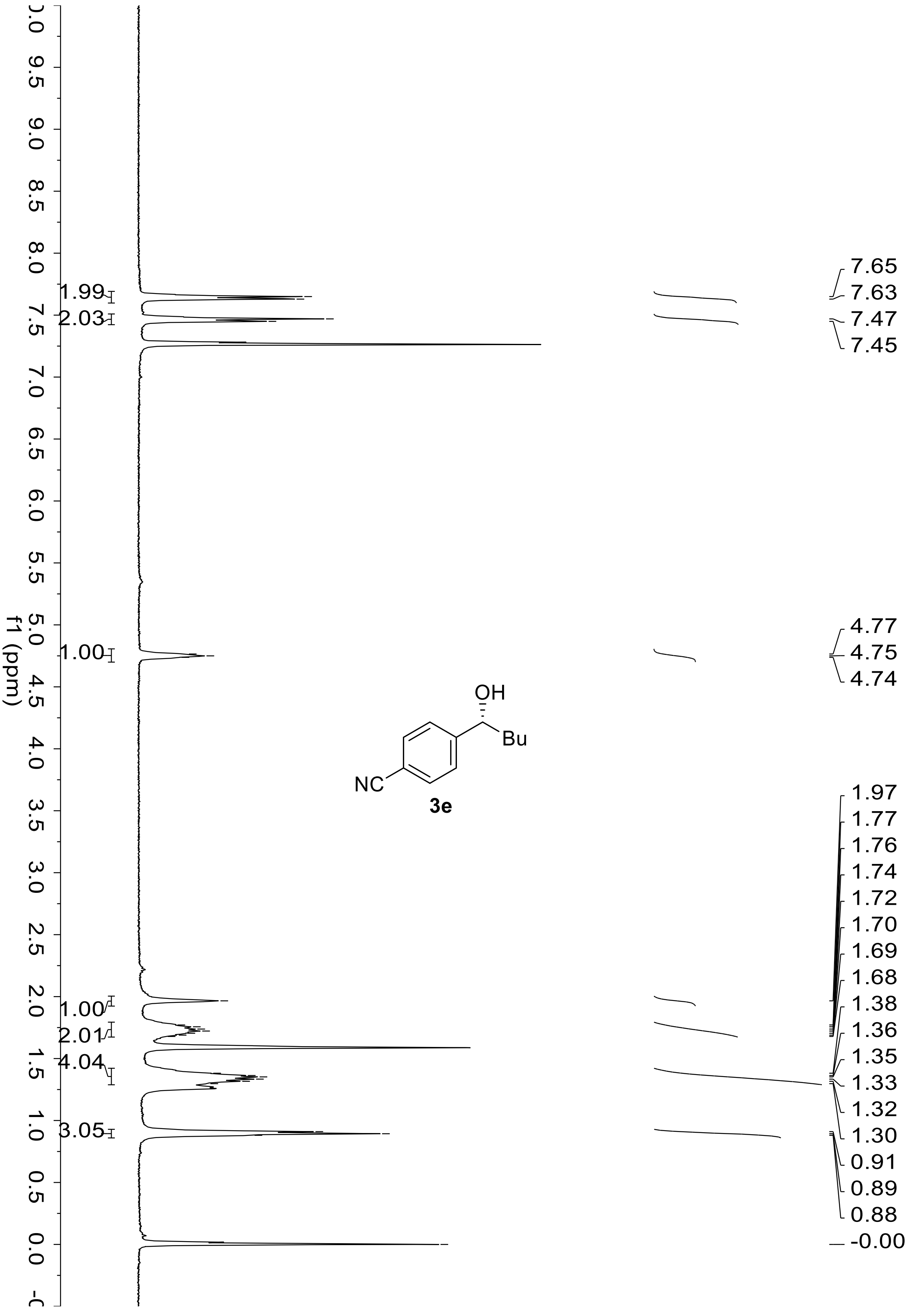
7.65
7.63
7.47
7.45
4.77
4.75
4.74
1.97
1.77
1.76
1.74
1.72
1.70
1.69
1.68
1.38
1.36
1.35
1.33
1.32
1.30
0.91
0.89
0.88
-0.00
1.99
2.03
1.00
1.00
2.01
4.04
3.05
OH
Bu
NC
3e
f1 (ppm)

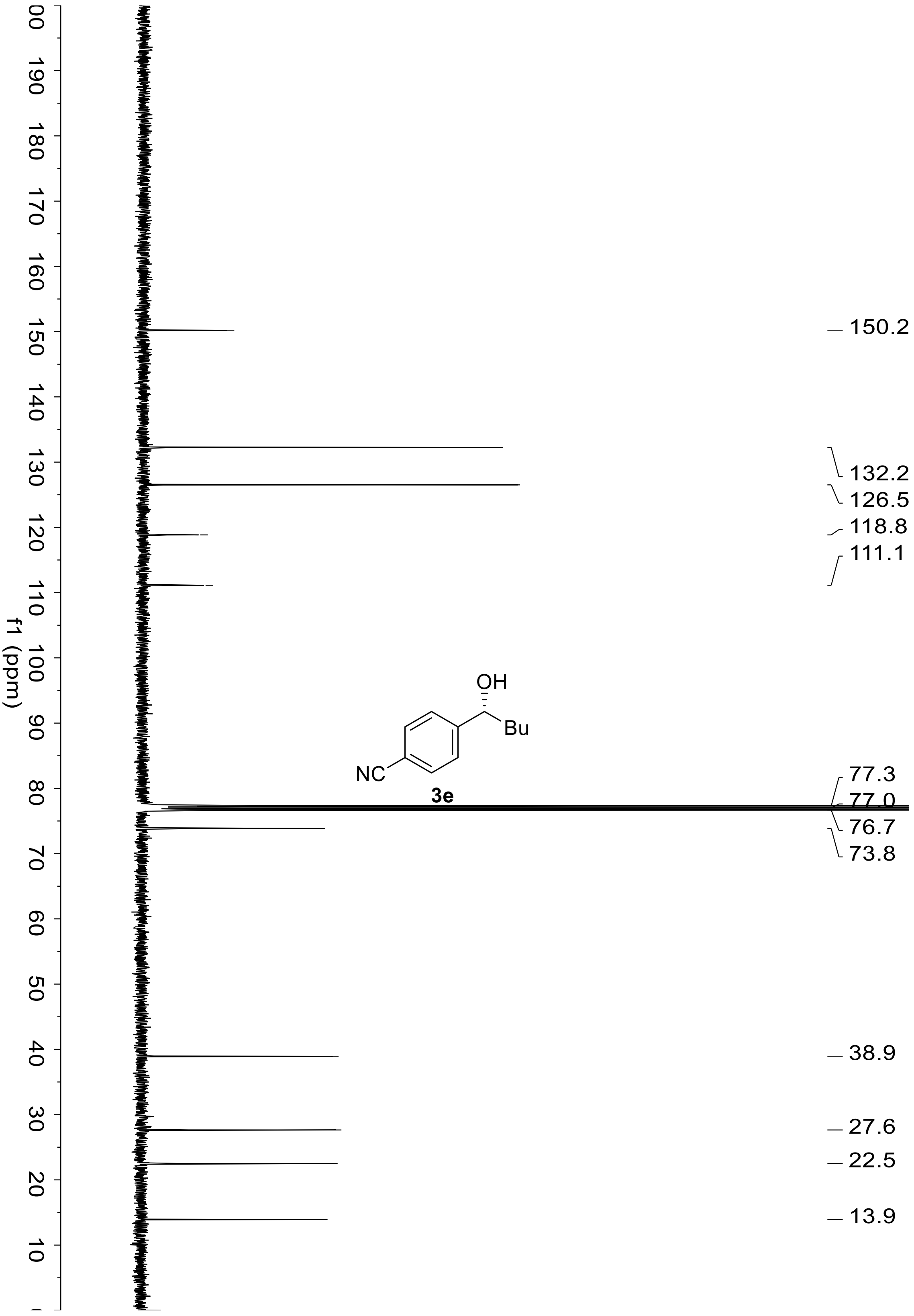
150.2
132.2
126.5
118.8
111.1
77.3
77.0
76.7
73.8
38.9
27.6
22.5
13.9
OH
Bu
NC
3e
f1 (ppm)
190
180
170
160
150
140
130
120
110
100
90
80
70
60
50
40
30
20
10

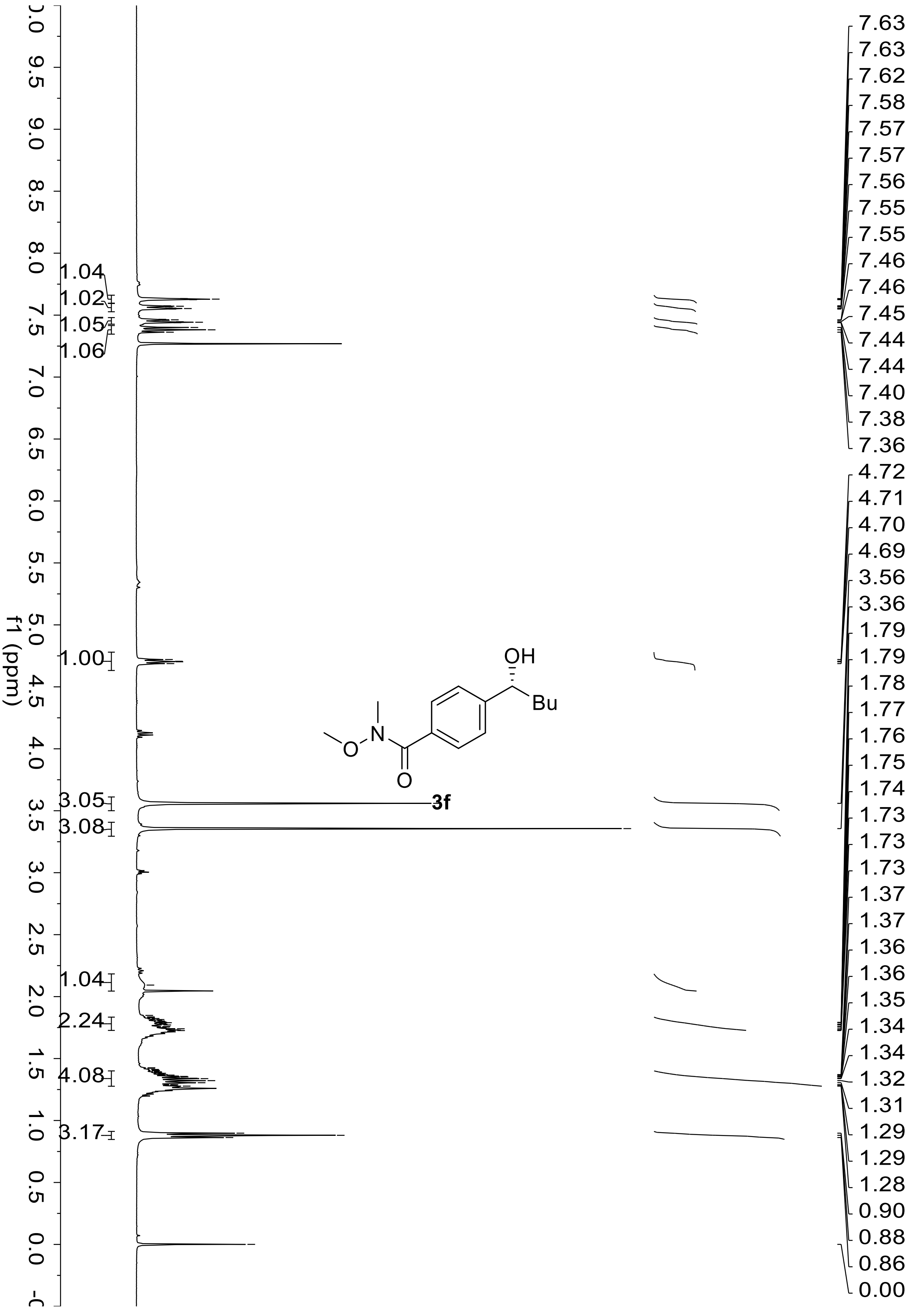
7.63
7.63
7.62
7.58
7.57
7.57
7.56
7.55
7.55
7.46
7.46
7.45
7.44
7.44
7.40
7.38
7.36
4.72
4.71
4.70
4.69
3.56
3.36
1.79
1.79
1.78
1.77
1.76
1.75
1.74
1.73
1.73
1.73
1.37
1.37
1.36
1.36
1.35
1.34
1.34
1.32
1.31
1.29
1.29
1.28
0.90
0.88
0.86
0.00
1.04
1.02
1.05
1.06
1.00
3.05
3.08
1.04
2.24
4.08
3.17
f1 (ppm)
OH
Bu
N
O
O
3f

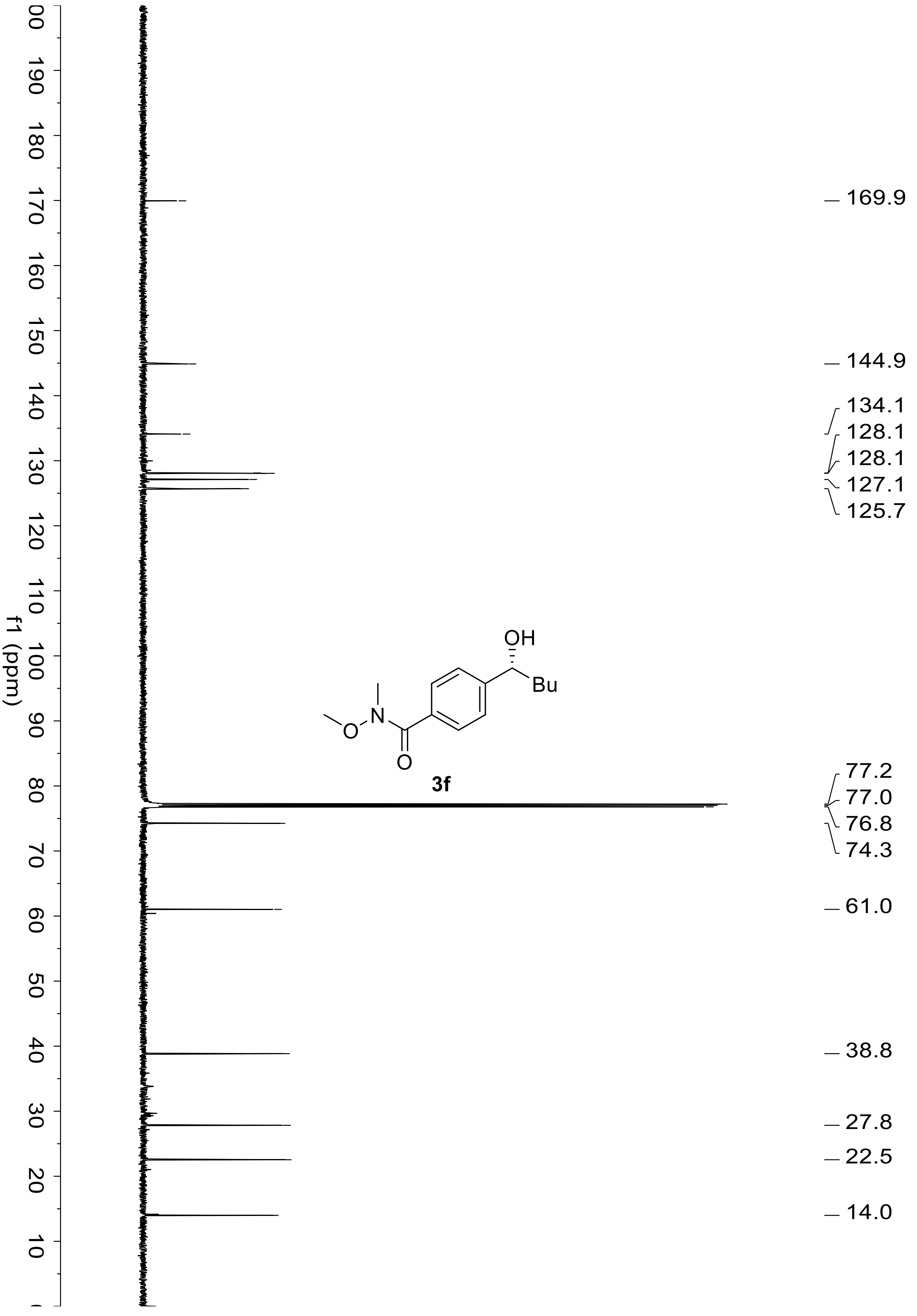

169.9
144.9
134.1
128.1
128.1
127.1
125.7
77.2
77.0
76.8
74.3
61.0
38.8
27.8
22.5
14.0
OH
Bu
N
O
O
3f
f1 (ppm)

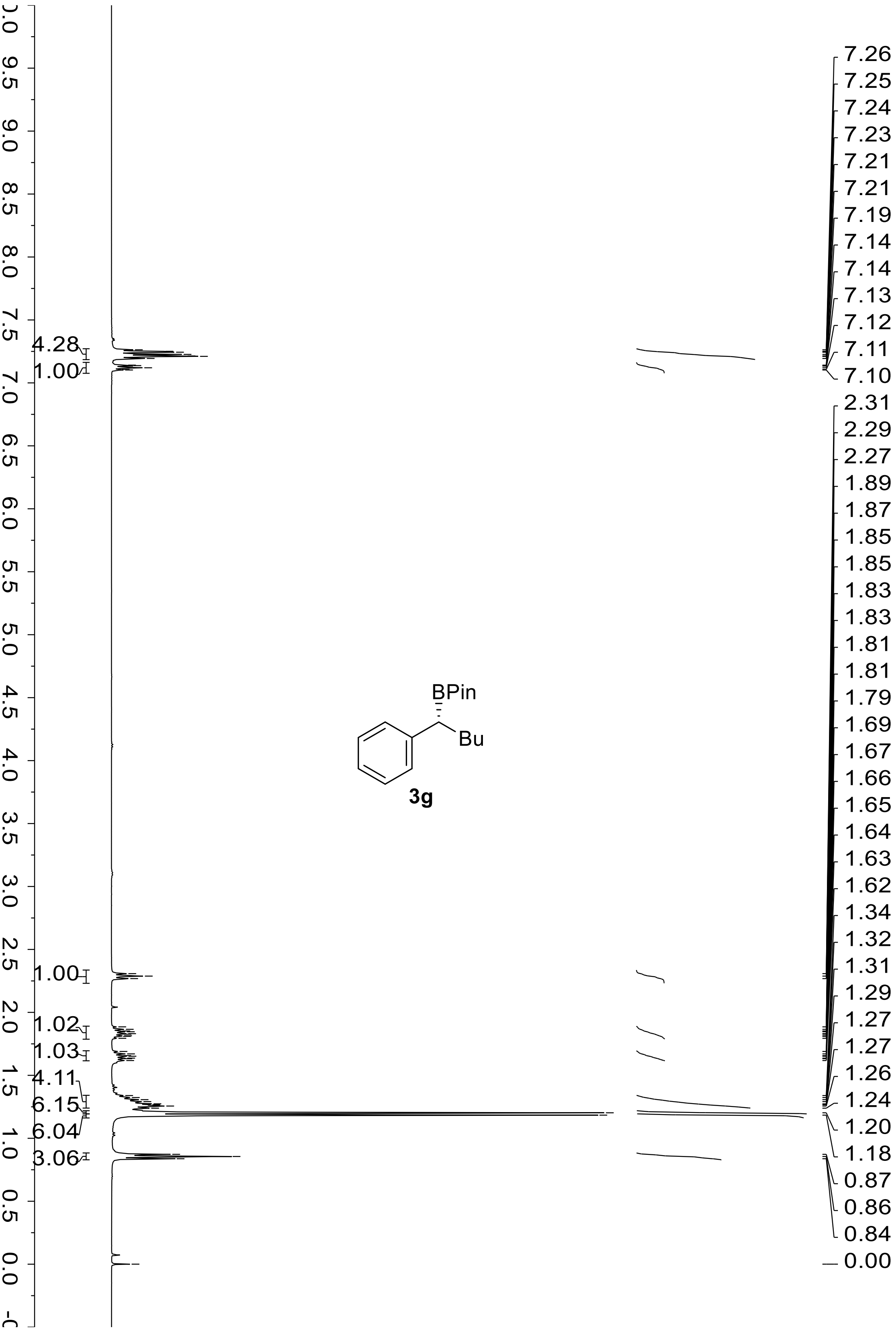
BPin
Bu
3g
f1 (ppm)
7.26
7.25
7.24
7.23
7.21
7.21
7.19
7.14
7.14
7.13
7.12
7.11
7.10
2.31
2.29
2.27
1.89
1.87
1.85
1.85
1.83
1.83
1.81
1.81
1.79
1.69
1.67
1.66
1.65
1.64
1.63
1.62
1.34
1.32
1.31
1.29
1.27
1.27
1.26
1.24
1.20
1.18
0.87
0.86
0.84
0.00
4.28
1.00
1.00
1.02
1.03
4.11
6.15
6.04
3.06

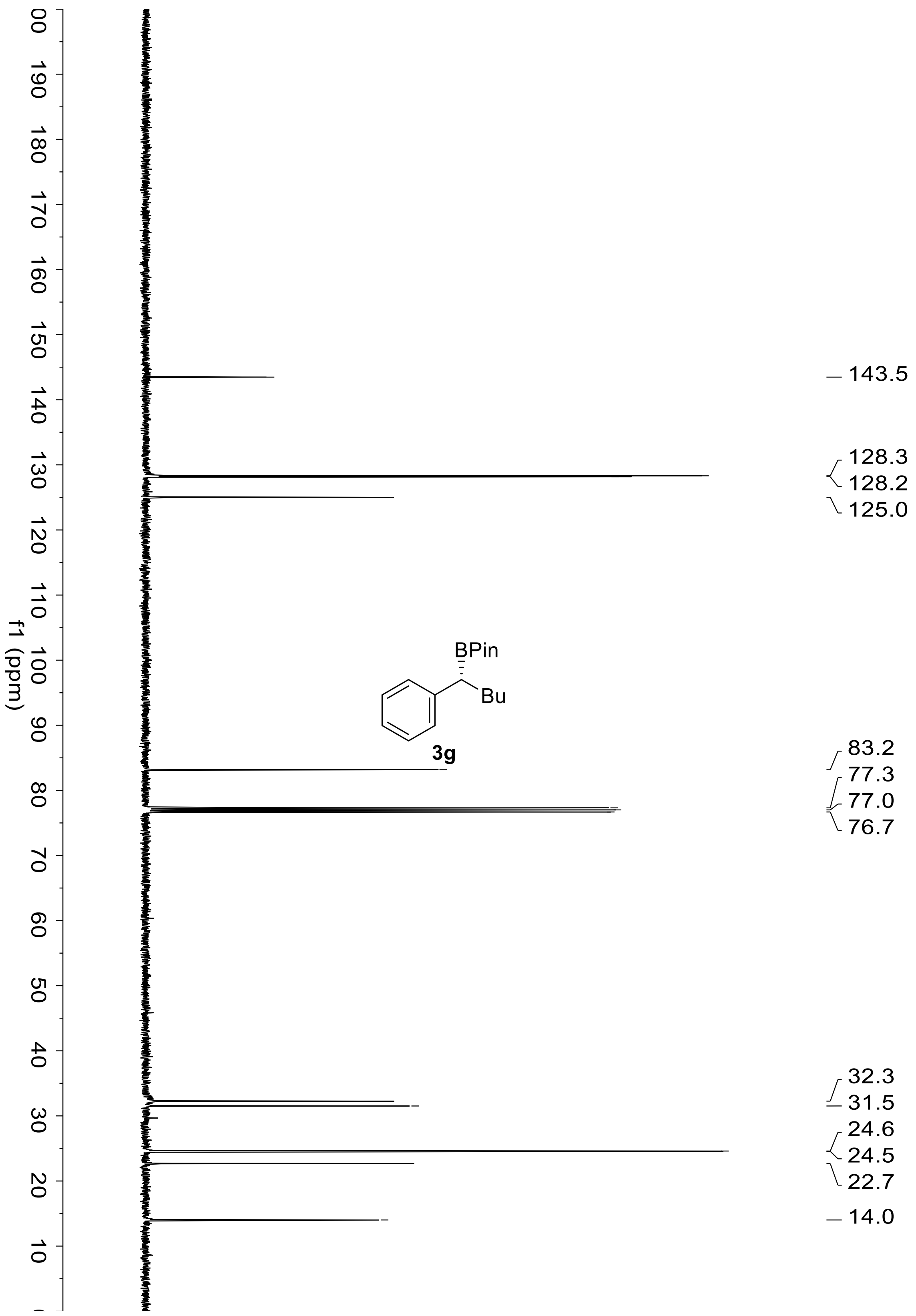

BPin
Bu
3g
143.5
128.3
128.2
125.0
83.2
77.3
77.0
76.7
32.3
31.5
24.6
24.5
22.7
14.0
f1 (ppm)

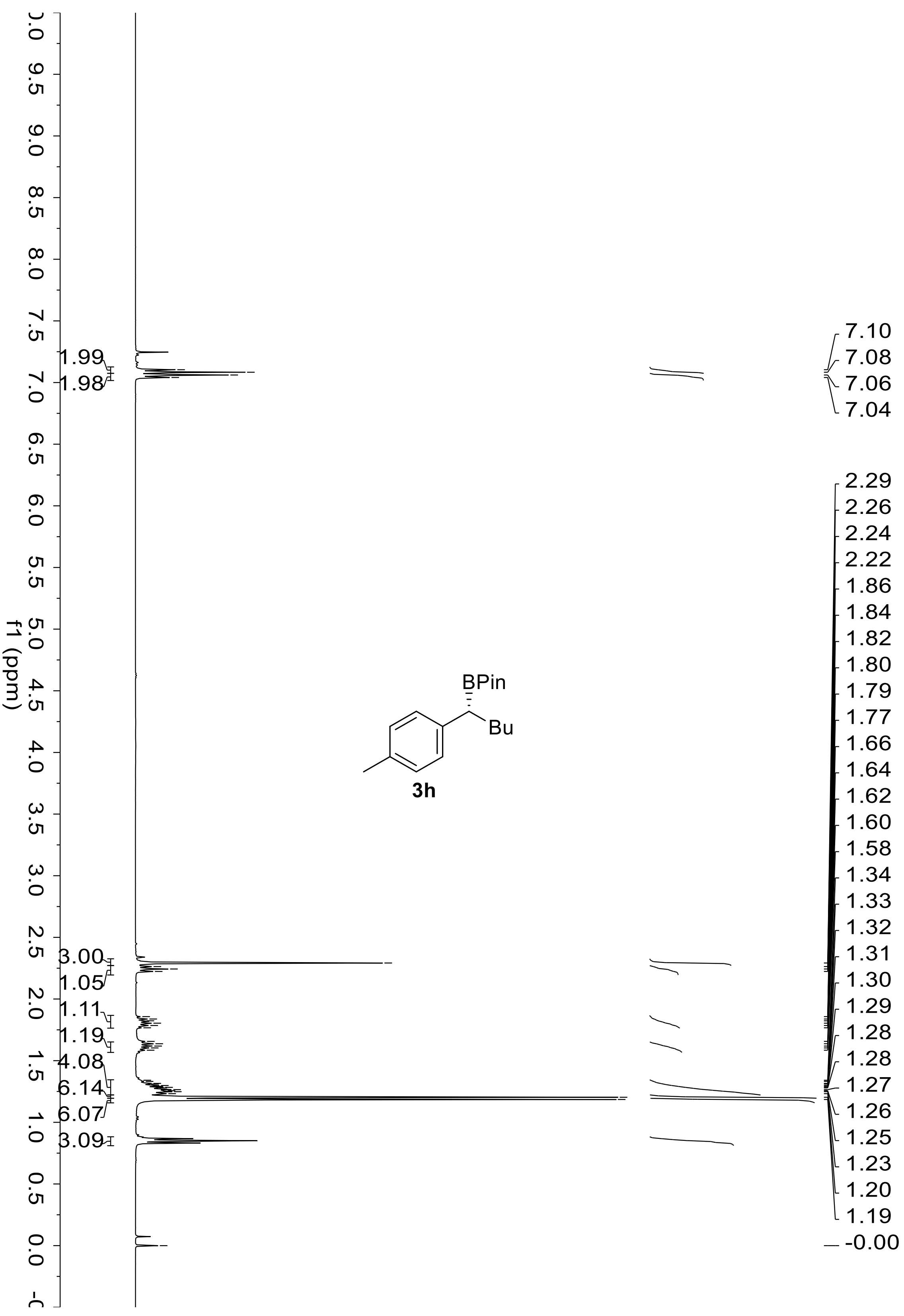

BPin
Bu
3h
7.10
7.08
7.06
7.04
2.29
2.26
2.24
2.22
1.86
1.84
1.82
1.80
1.79
1.77
1.66
1.64
1.62
1.60
1.58
1.34
1.33
1.32
1.31
1.30
1.29
1.28
1.28
1.27
1.26
1.25
1.23
1.20
1.19
-0.00
1.99
1.98
3.00
1.05
1.11
1.19
4.08
6.14
6.07
3.09
f1 (ppm)

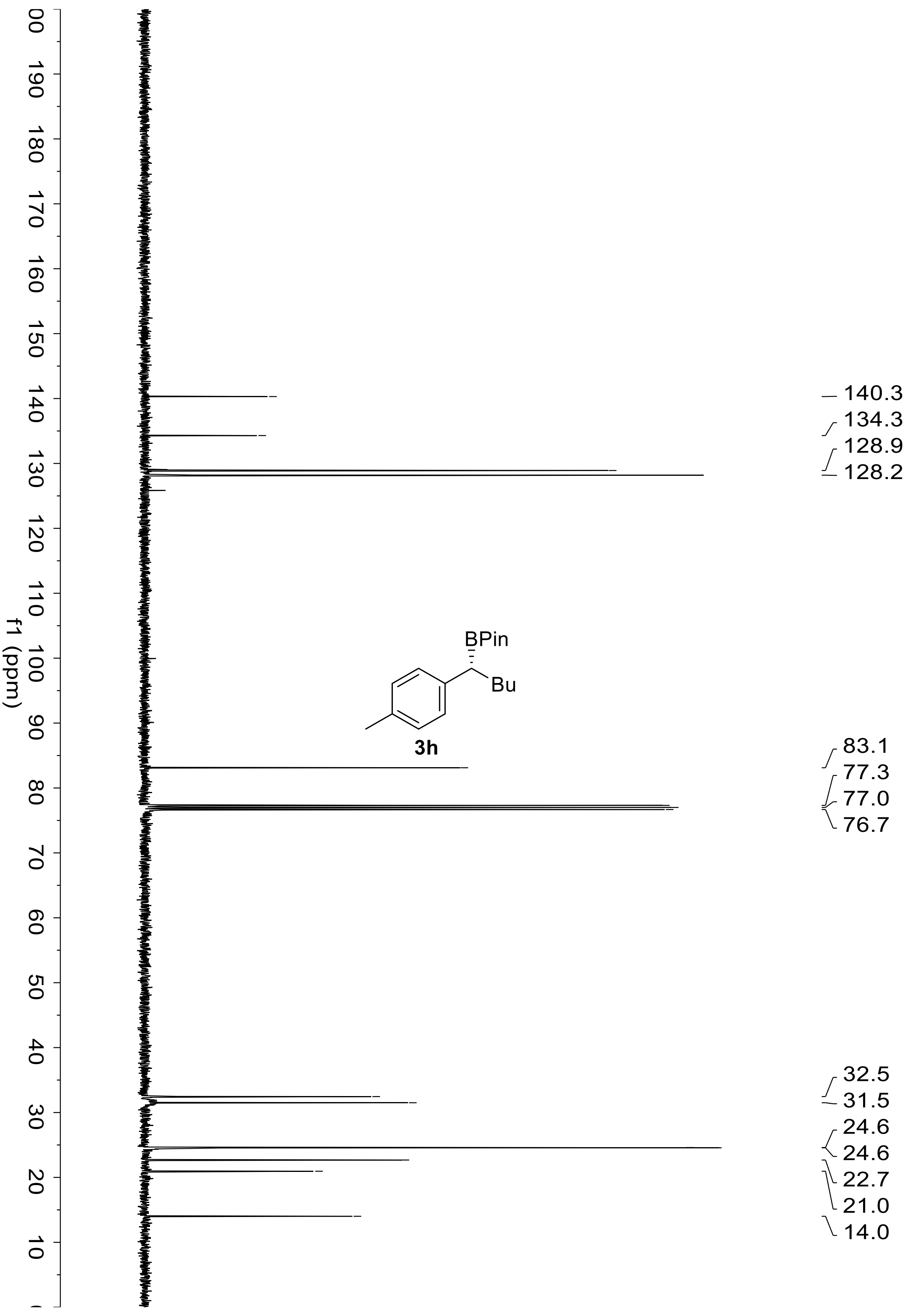

140.3
134.3
128.9
128.2
BPin
Bu
3h
83.1
77.3
77.0
76.7
32.5
31.5
24.6
24.6
22.7
21.0
14.0
f1 (ppm)

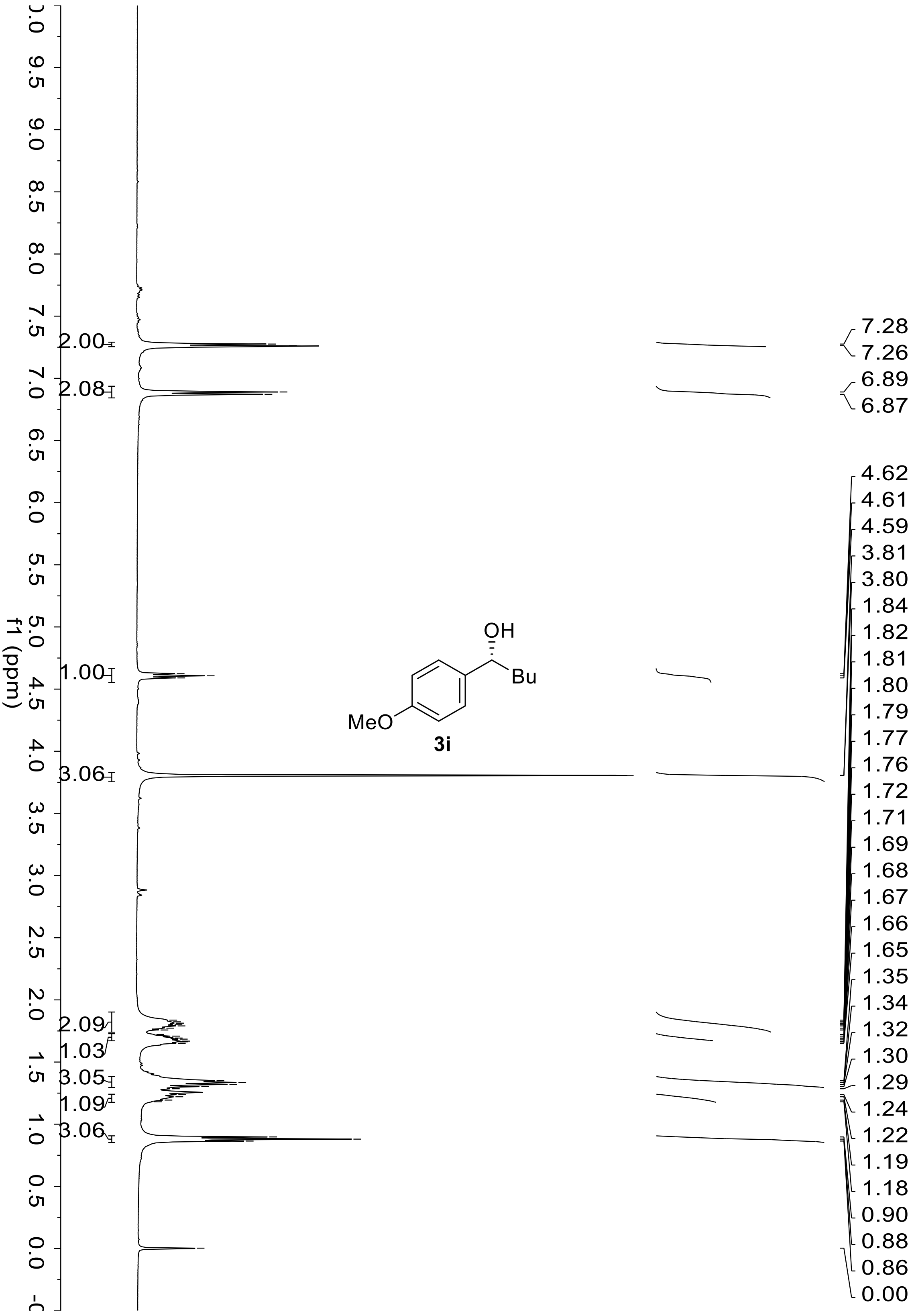
OH
Bu
MeO
3i
f1 (ppm)
7.28
7.26
6.89
6.87
4.62
4.61
4.59
3.81
3.80
1.84
1.82
1.81
1.80
1.79
1.77
1.76
1.72
1.71
1.69
1.68
1.67
1.66
1.65
1.35
1.34
1.32
1.30
1.29
1.24
1.22
1.19
1.18
0.90
0.88
0.86
0.00
2.00
2.08
1.00
3.06
2.09
1.03
3.05
1.09
3.06

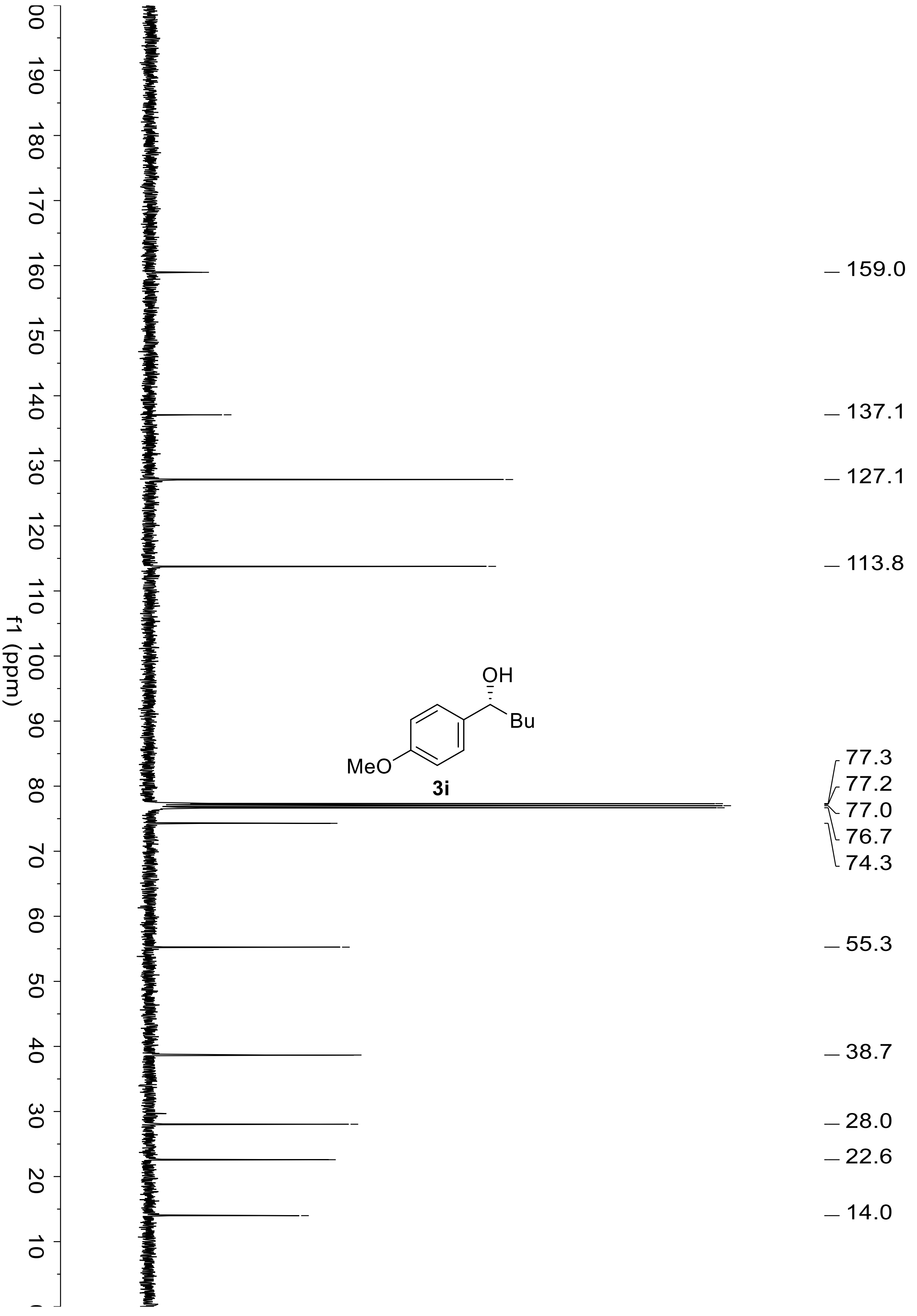

159.0
137.1
127.1
113.8
77.3
77.2
77.0
76.7
74.3
55.3
38.7
28.0
22.6
14.0
OH
Bu
MeO
3i
f1 (ppm)

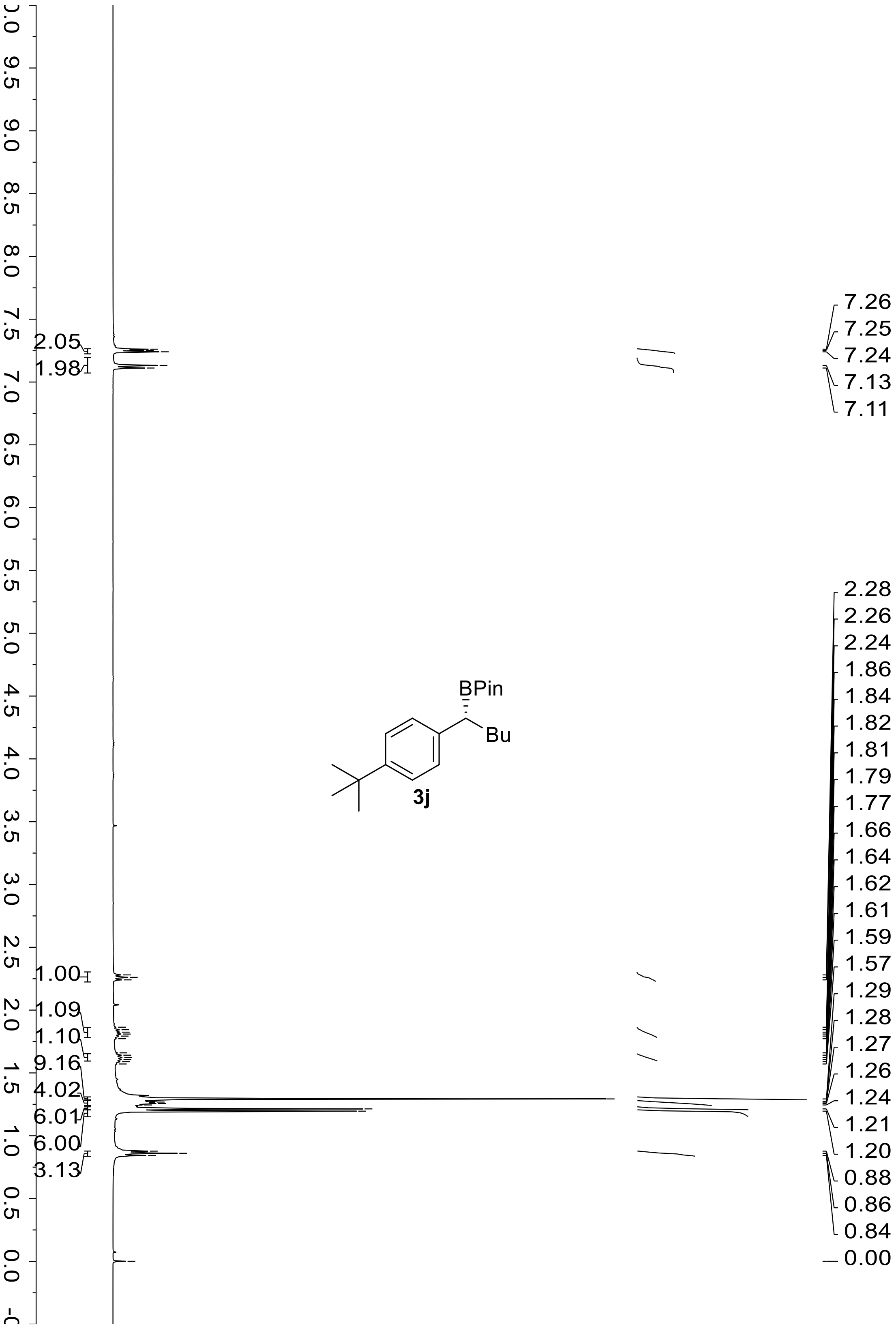
7.26
7.25
7.24
7.13
7.11
2.28
2.26
2.24
1.86
1.84
1.82
1.81
1.79
1.77
1.66
1.64
1.62
1.61
1.59
1.57
1.29
1.28
1.27
1.26
1.24
1.21
1.20
0.88
0.86
0.84
0.00
BPin
Bu
3j
2.05
1.98
1.00
1.09
1.10
9.16
4.02
6.01
6.00
3.13
f1 (ppm)

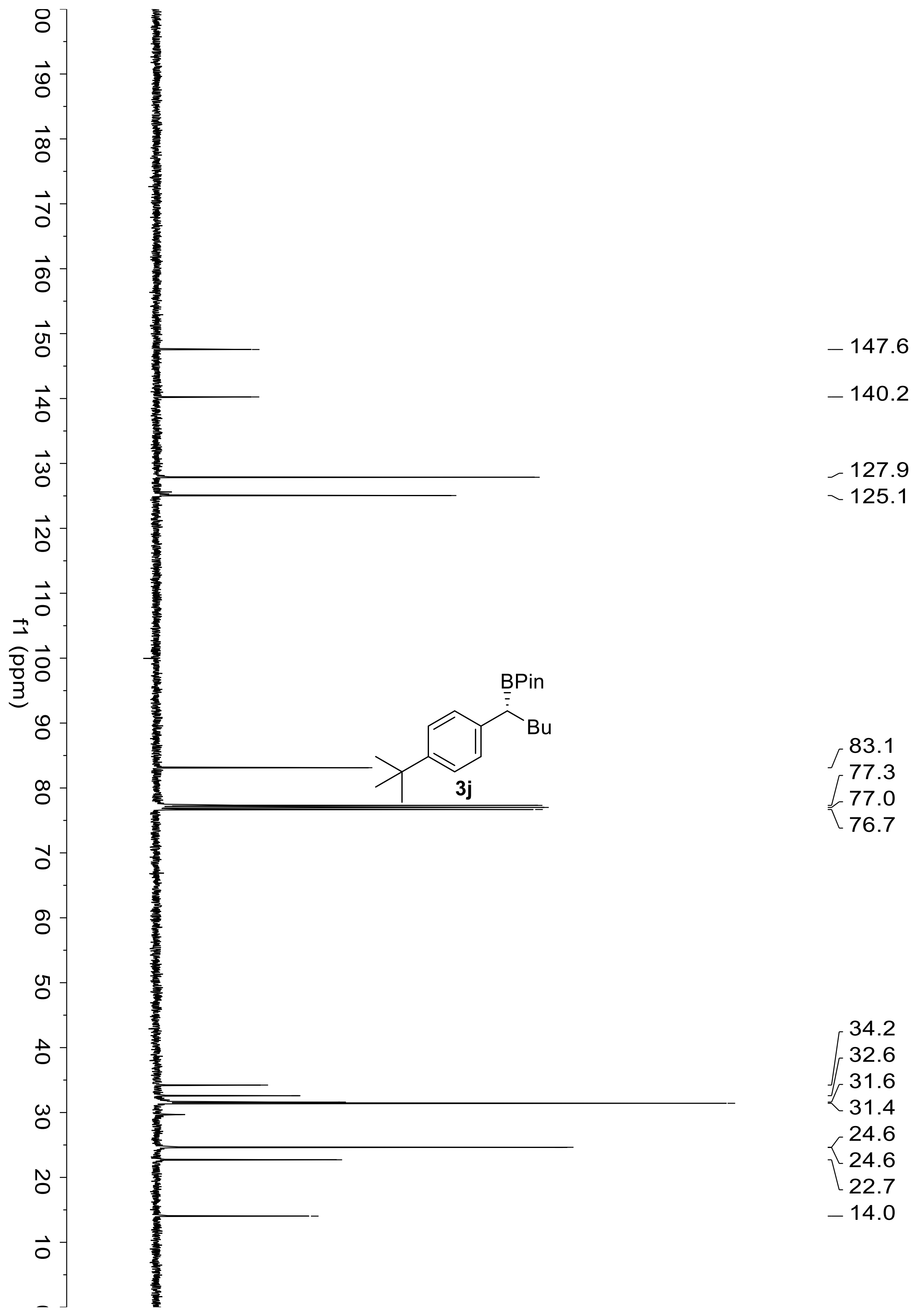
147.6
140.2
127.9
125.1
83.1
77.3
77.0
76.7
34.2
32.6
31.6
31.4
24.6
24.6
22.7
14.0
BPin
Bu
3j
f1 (ppm)
190
180
170
160
150
140
130
120
110
100
90
80
70
60
50
40
30
20
10

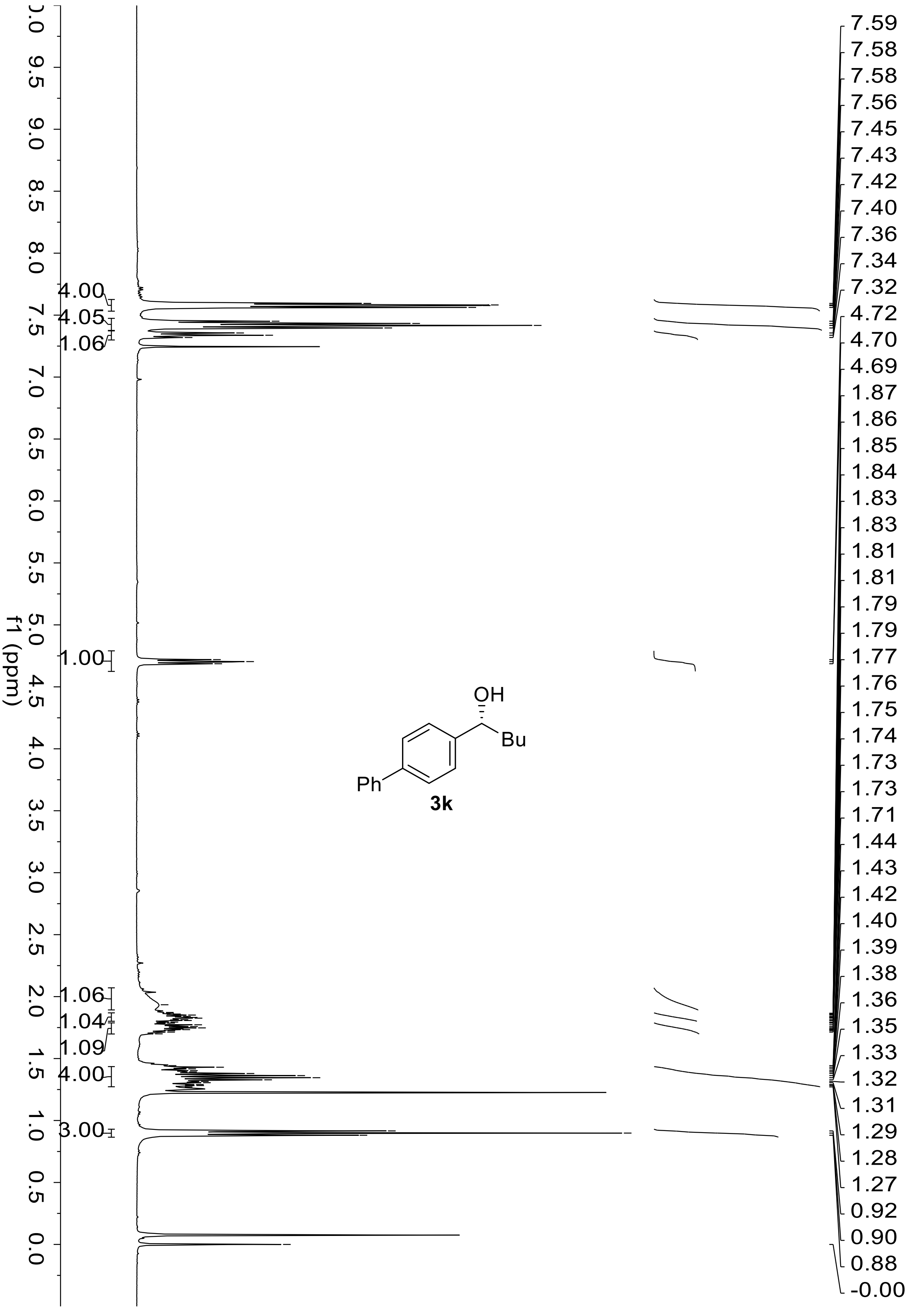
7.59
7.58
7.58
7.56
7.45
7.43
7.42
7.40
7.36
7.34
7.32
4.72
4.70
4.69
1.87
1.86
1.85
1.84
1.83
1.83
1.81
1.81
1.79
1.79
1.77
1.76
1.75
1.74
1.73
1.73
1.71
1.44
1.43
1.42
1.40
1.39
1.38
1.36
1.35
1.33
1.32
1.31
1.29
1.28
1.27
0.92
0.90
0.88
-0.00
4.00
4.05
1.06
1.00
1.06
1.04
1.09
4.00
3.00
f1 (ppm)
OH
Bu
Ph
3k

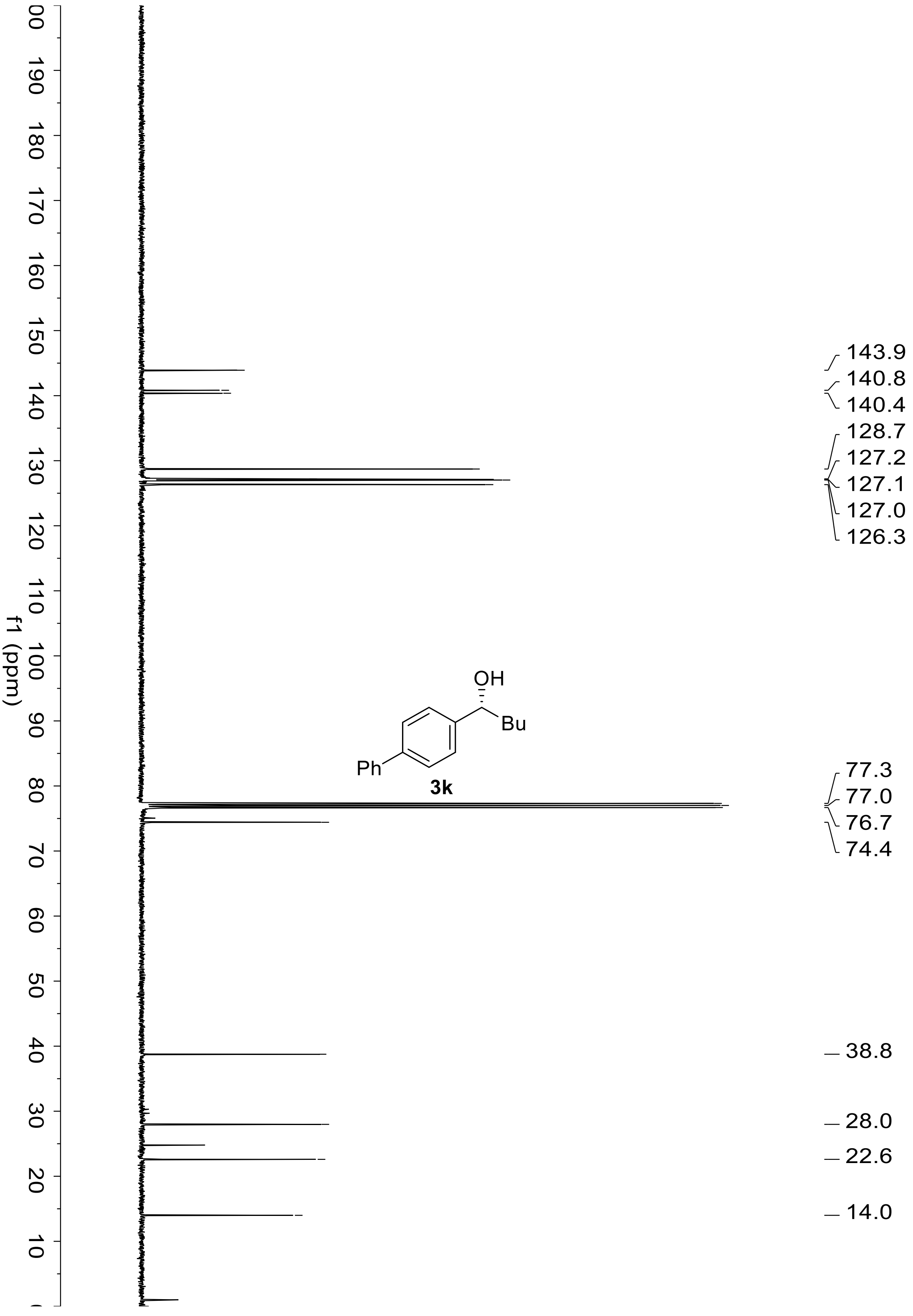
143.9
140.8
140.4
128.7
127.2
127.1
127.0
126.3
77.3
77.0
76.7
74.4
38.8
28.0
22.6
14.0
OH
Bu
Ph
3k
f1 (ppm)

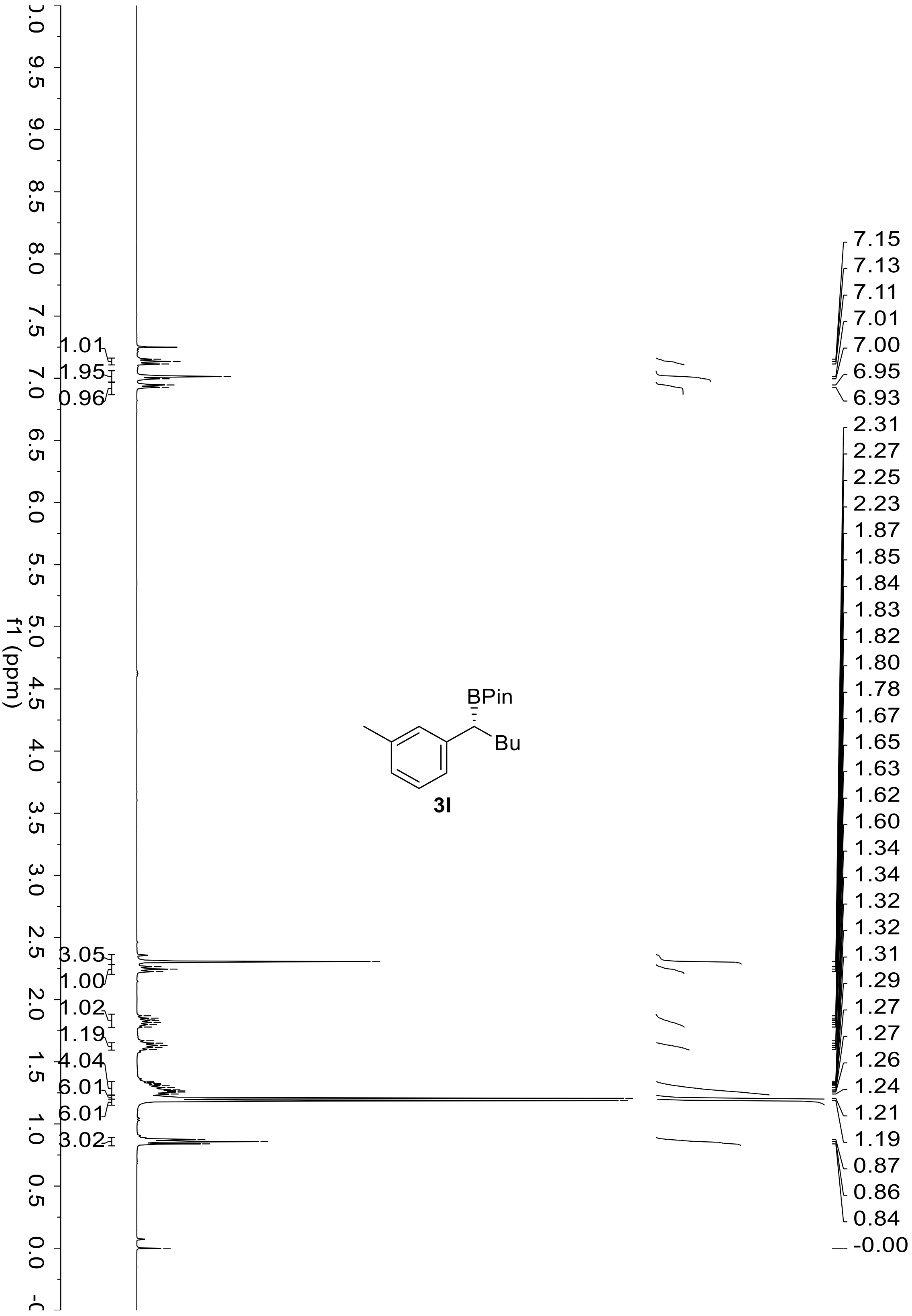
7.15
7.13
7.11
7.01
7.00
6.95
6.93
2.31
2.27
2.25
2.23
1.87
1.85
1.84
1.83
1.82
1.80
1.78
1.67
1.65
1.63
1.62
1.60
1.34
1.34
1.32
1.32
1.31
1.29
1.27
1.27
1.26
1.24
1.21
1.19
0.87
0.86
0.84
-0.00
BPin
Bu
3l
1.01
1.95
0.96
3.05
1.00
1.02
1.19
4.04
6.01
6.01
3.02
f1 (ppm)

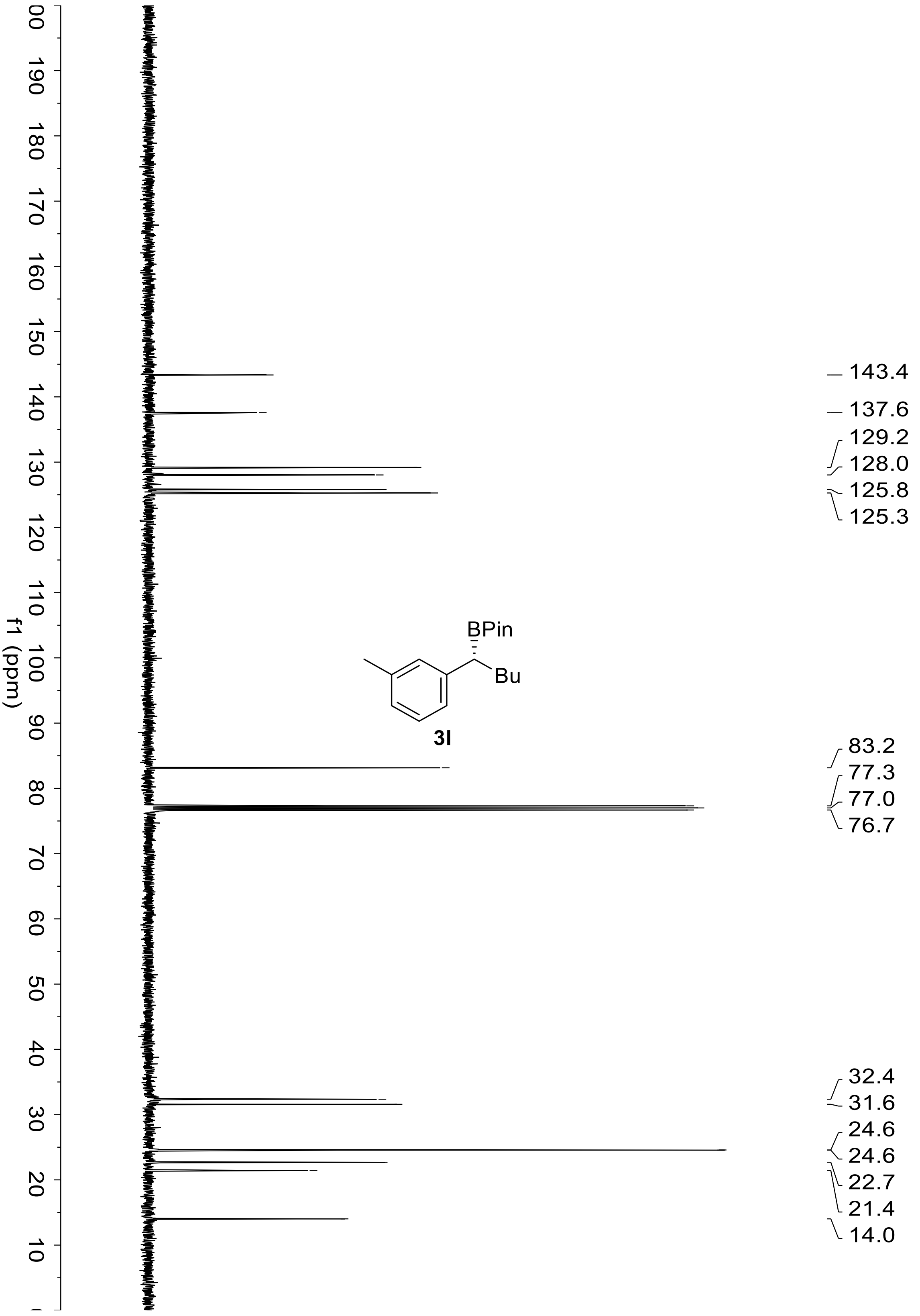
143.4
137.6
129.2
128.0
125.8
125.3
BPin
Bu
3l
83.2
77.3
77.0
76.7
32.4
31.6
24.6
24.6
22.7
21.4
14.0
f1 (ppm)

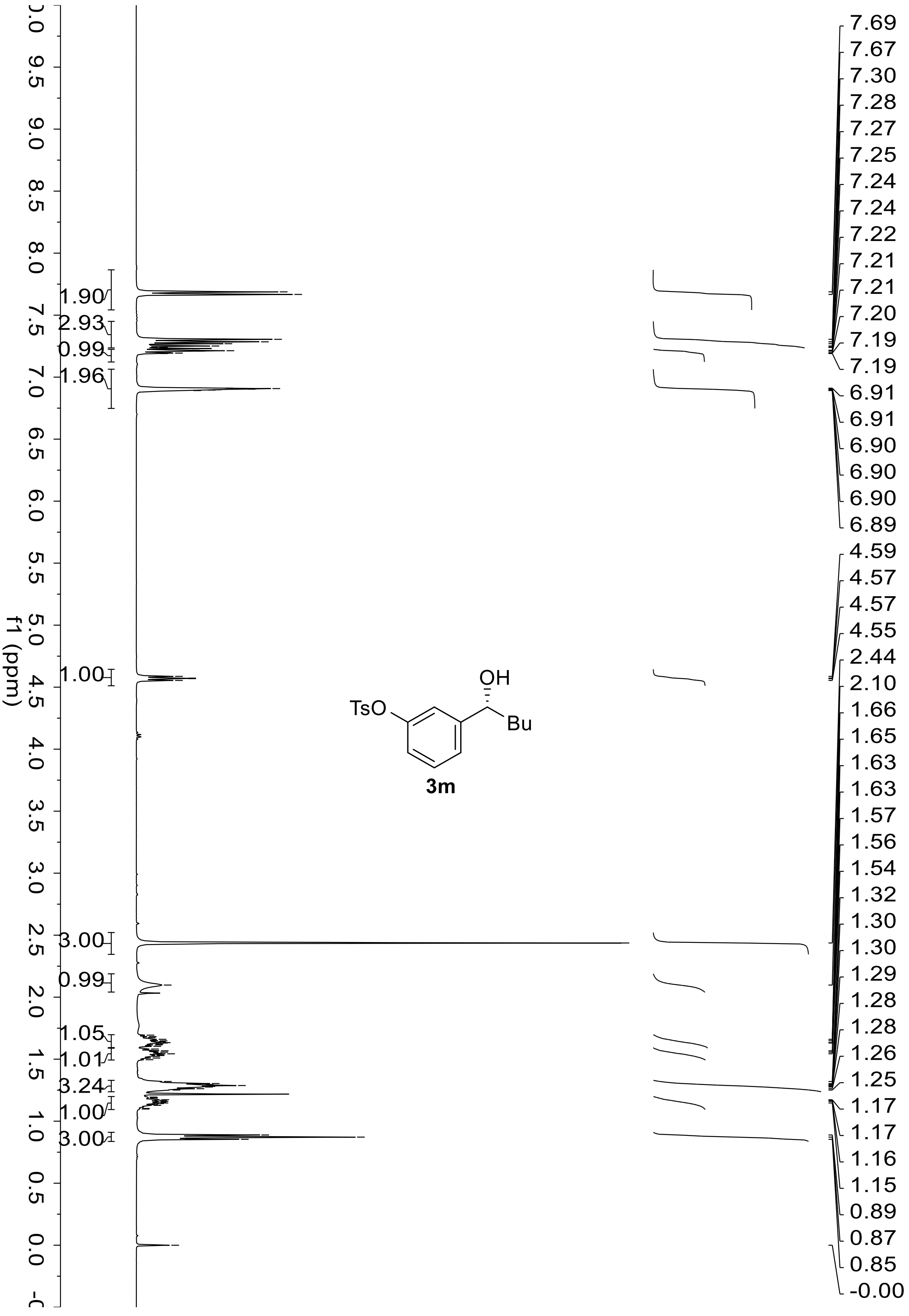

7.69
7.67
7.30
7.28
7.27
7.25
7.24
7.24
7.22
7.21
7.21
7.20
7.19
7.19
6.91
6.91
6.90
6.90
6.90
6.89
4.59
4.57
4.57
4.55
2.44
2.10
1.66
1.65
1.63
1.63
1.57
1.56
1.54
1.32
1.30
1.30
1.29
1.28
1.28
1.26
1.25
1.17
1.17
1.16
1.15
0.89
0.87
0.85
-0.00
OH
TsO
Bu
3m
1.90
2.93
0.99
1.96
1.00
3.00
0.99
1.05
1.01
3.24
1.00
3.00
f1 (ppm)

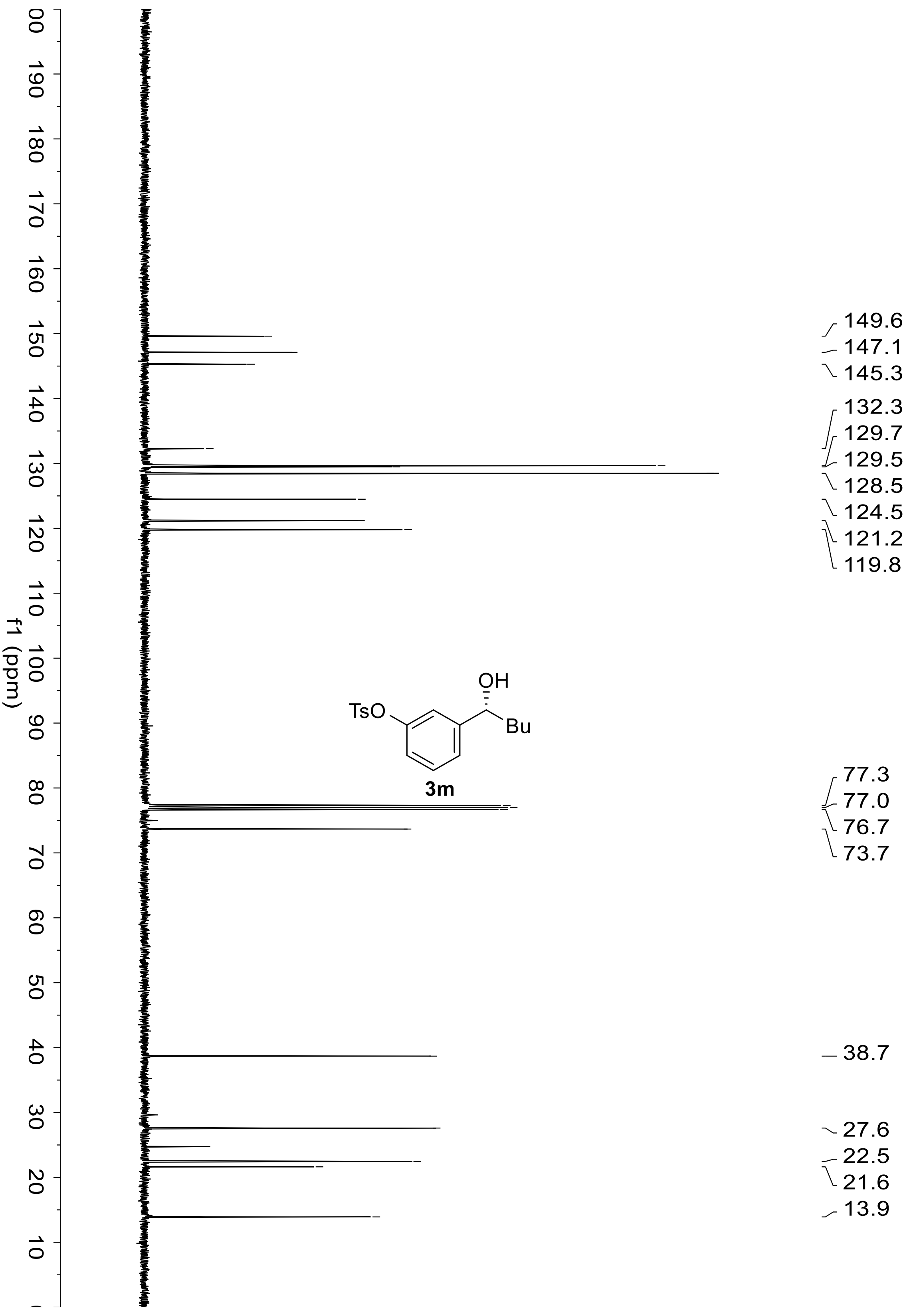
149.6
147.1
145.3
132.3
129.7
129.5
128.5
124.5
121.2
119.8
77.3
77.0
76.7
73.7
38.7
27.6
22.5
21.6
13.9
OH
TsO
Bu
3m
f1 (ppm)

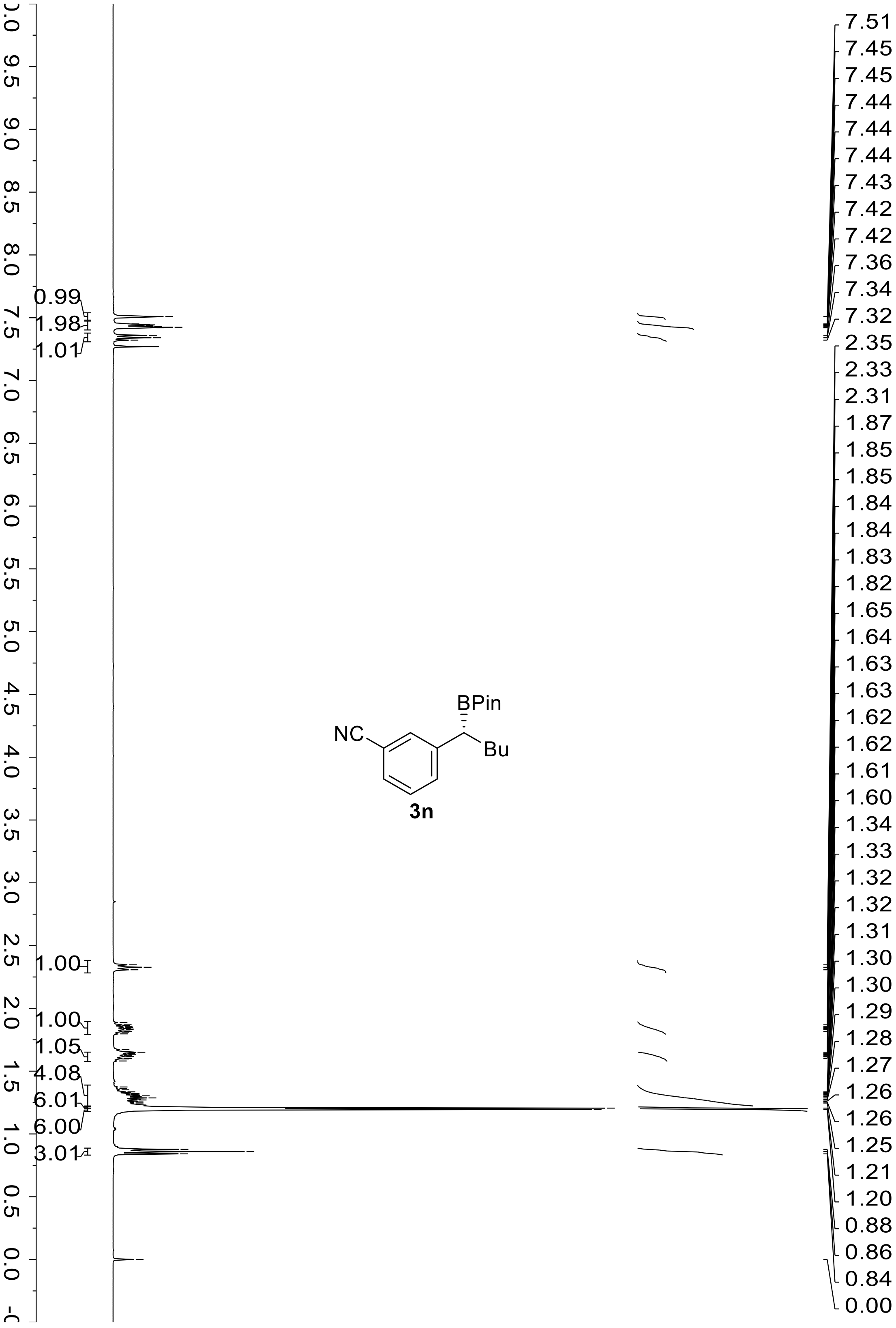
7.51
7.45
7.45
7.44
7.44
7.44
7.43
7.42
7.42
7.36
7.34
7.32
2.35
2.33
2.31
1.87
1.85
1.85
1.84
1.84
1.83
1.82
1.65
1.64
1.63
1.63
1.62
1.62
1.61
1.60
1.34
1.33
1.32
1.32
1.31
1.30
1.30
1.29
1.28
1.27
1.26
1.26
1.25
1.21
1.20
0.88
0.86
0.84
0.00
0.99
1.98
1.01
1.00
1.00
1.05
4.08
6.01
6.00
3.01
f1 (ppm)
BPin
NC
Bu
3n

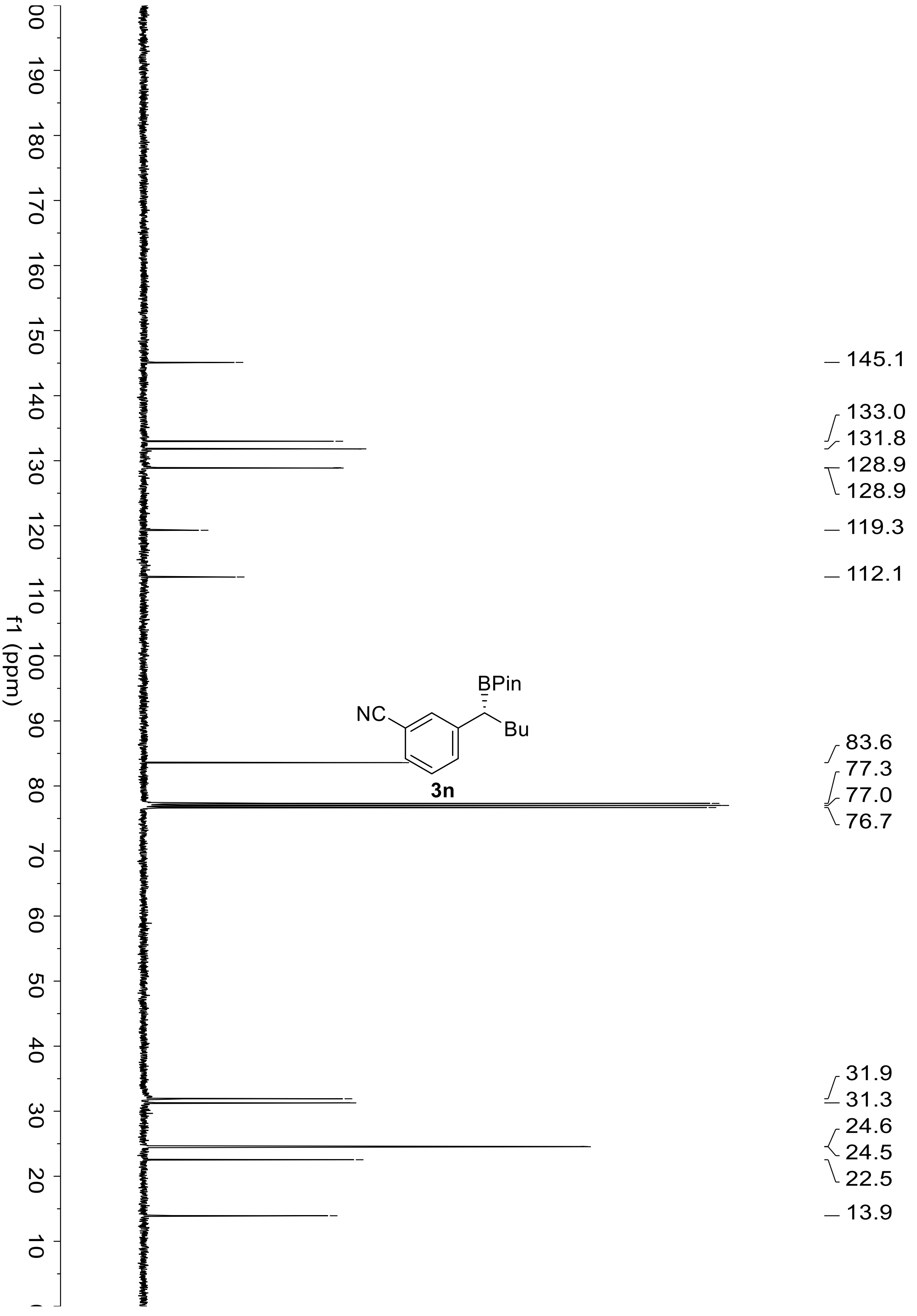
145.1
133.0
131.8
128.9
128.9
119.3
112.1
83.6
77.3
77.0
76.7
31.9
31.3
24.6
24.5
22.5
13.9
BPin
NC
Bu
3n
f1 (ppm)

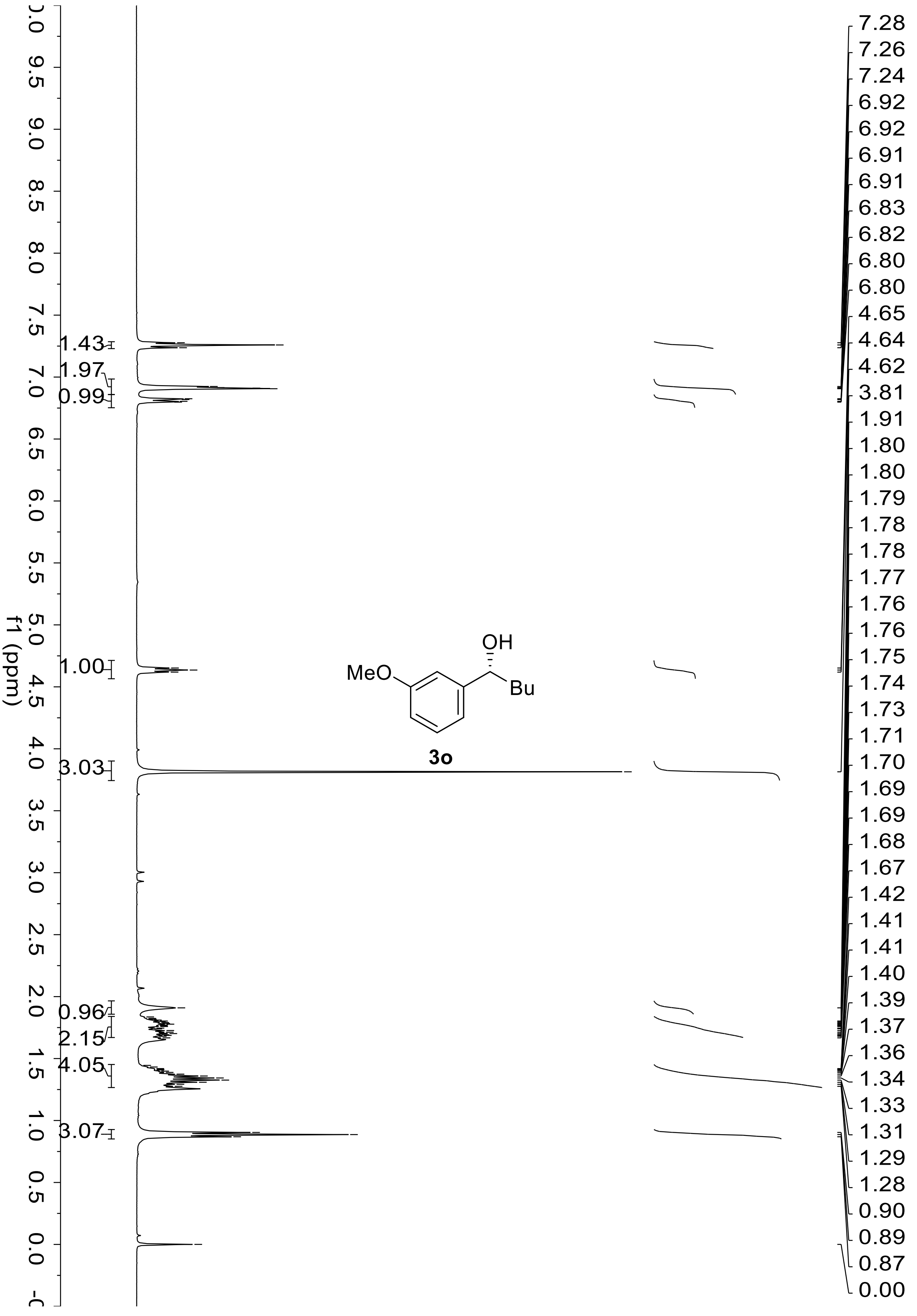
7.28
7.26
7.24
6.92
6.92
6.91
6.91
6.83
6.82
6.80
6.80
4.65
4.64
4.62
3.81
1.91
1.80
1.80
1.79
1.78
1.78
1.77
1.76
1.76
1.75
1.74
1.73
1.71
1.70
1.69
1.69
1.68
1.67
1.42
1.41
1.41
1.40
1.39
1.37
1.36
1.34
1.33
1.31
1.29
1.28
0.90
0.89
0.87
0.00
OH
MeO
Bu
3o
1.43
1.97
0.99
1.00
3.03
0.96
2.15
4.05
3.07
f1 (ppm)

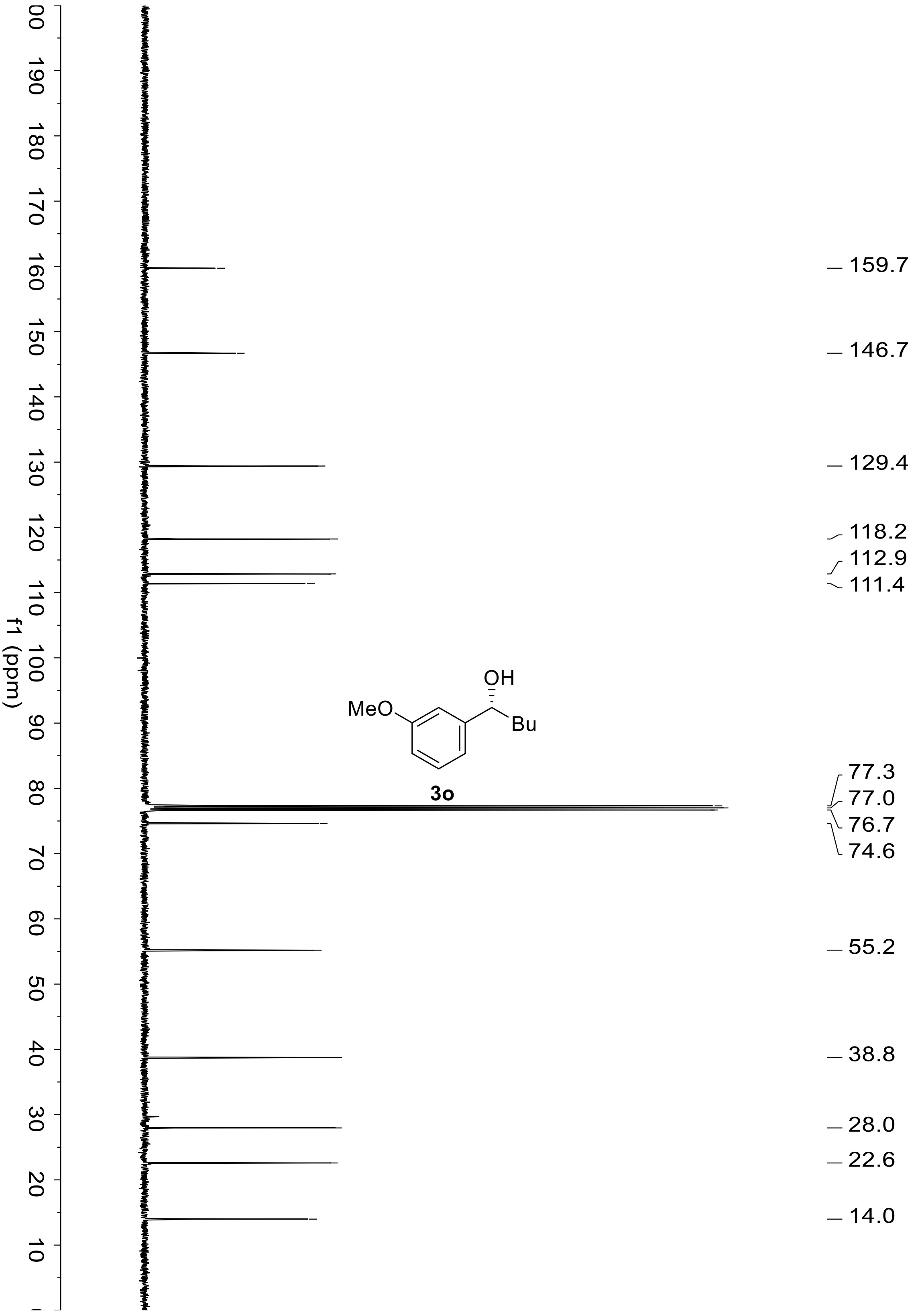
159.7
146.7
129.4
118.2
112.9
111.4
77.3
77.0
76.7
74.6
55.2
38.8
28.0
22.6
14.0
OH
MeO
Bu
3o
f1 (ppm)

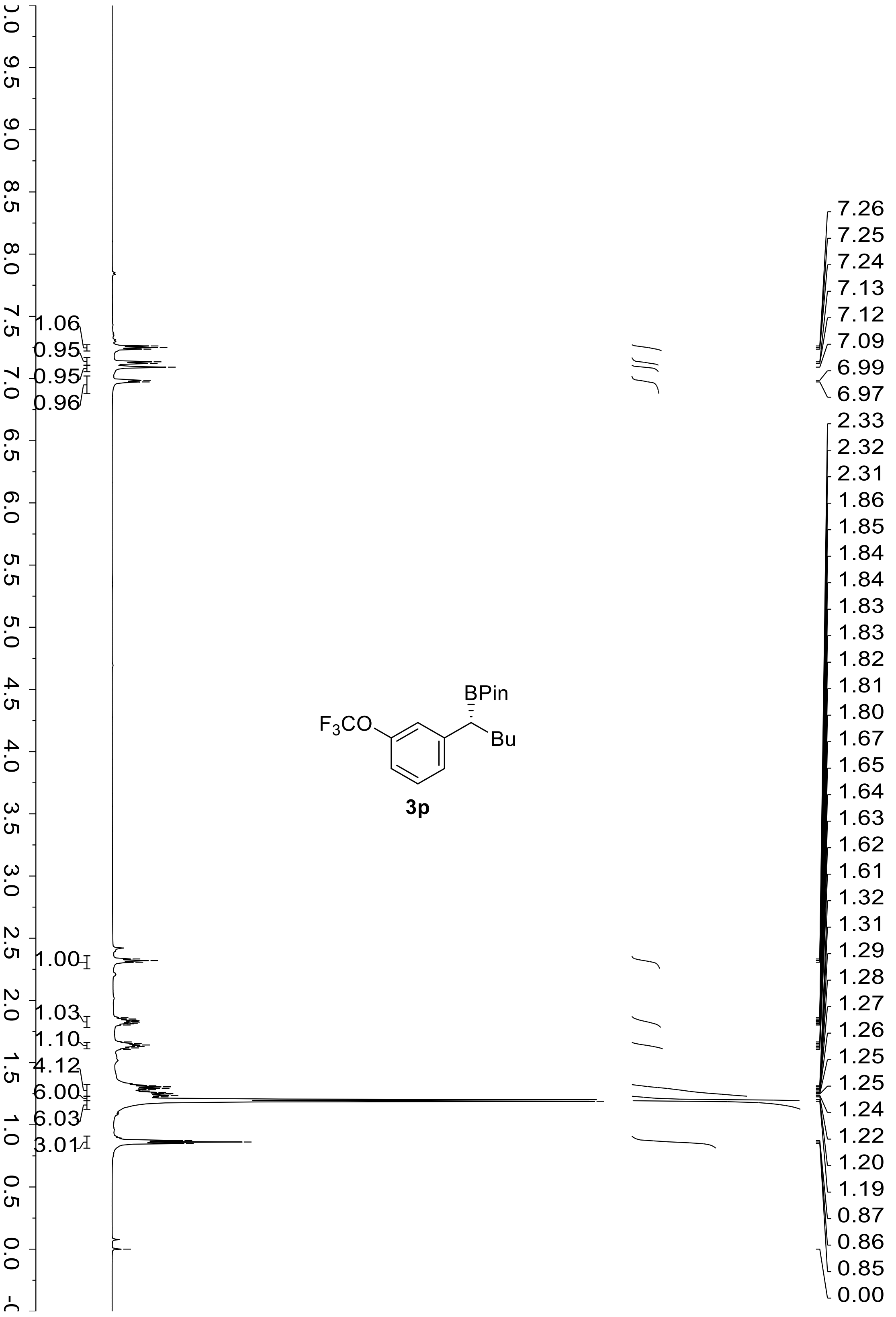

BPin
F3CO
Bu
3p
f1 (ppm)
7.26
7.25
7.24
7.13
7.12
7.09
6.99
6.97
2.33
2.32
2.31
1.86
1.85
1.84
1.84
1.83
1.83
1.82
1.81
1.80
1.67
1.65
1.64
1.63
1.62
1.61
1.32
1.31
1.29
1.28
1.27
1.26
1.25
1.25
1.24
1.22
1.20
1.19
0.87
0.86
0.85
0.00
1.06
0.95
0.95
0.96
1.00
1.03
1.10
4.12
6.00
6.03
3.01

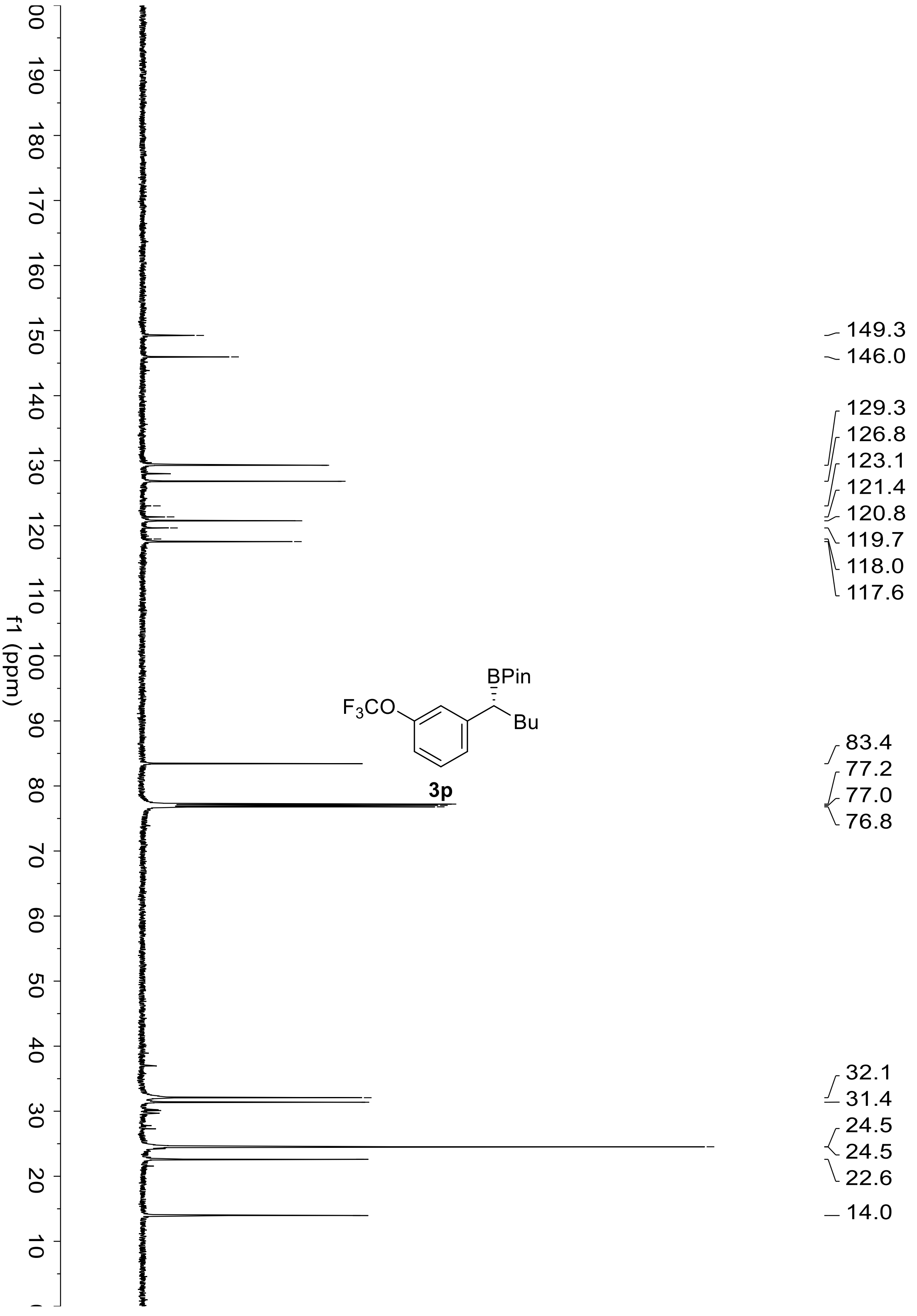
149.3
146.0
129.3
126.8
123.1
121.4
120.8
119.7
118.0
117.6
83.4
77.2
77.0
76.8
32.1
31.4
24.5
24.5
22.6
14.0
f1 (ppm)
BPin
F_3CO
Bu
3p

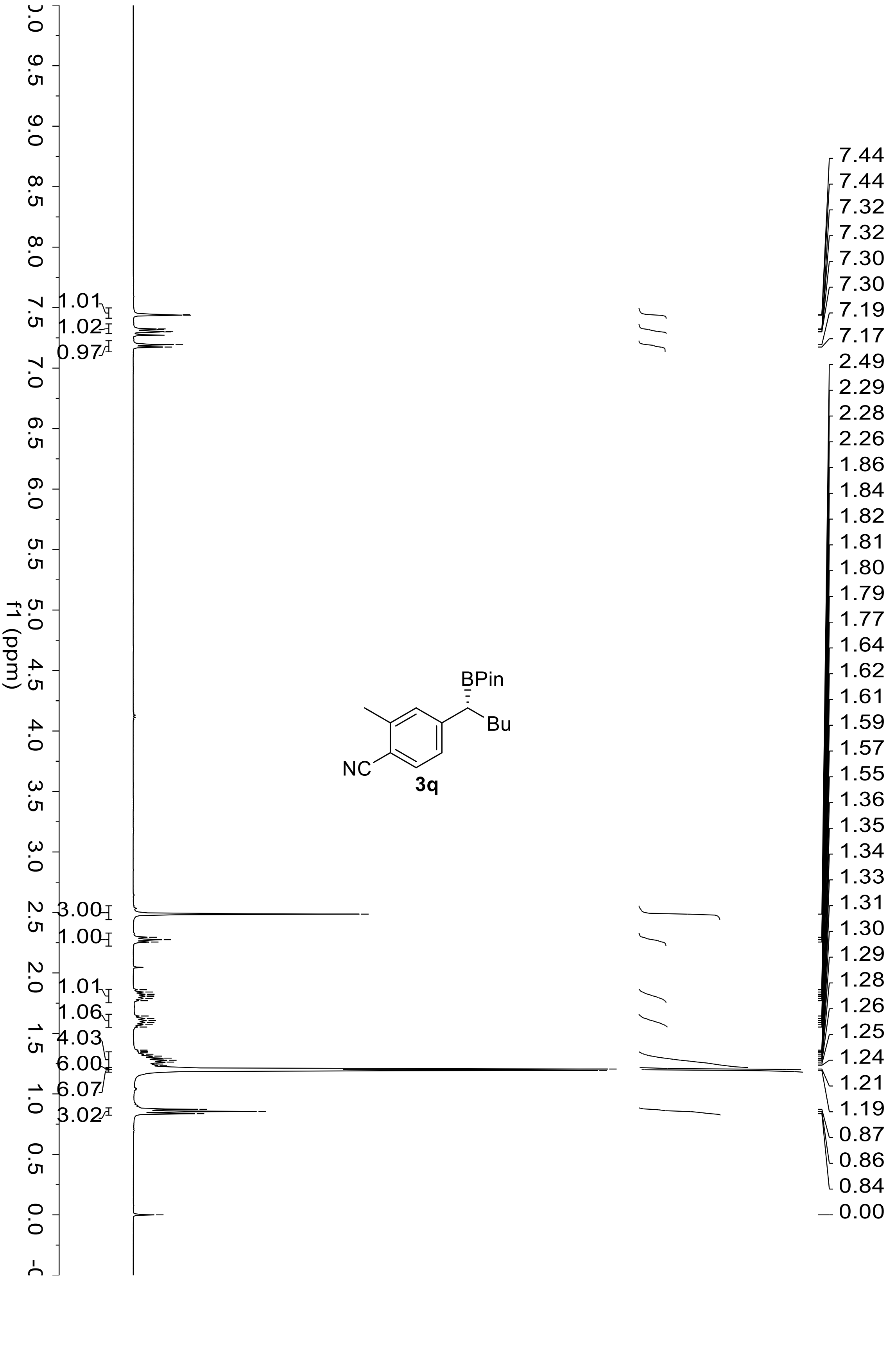
7.44
7.44
7.32
7.32
7.30
7.30
7.19
7.17
2.49
2.29
2.28
2.26
1.86
1.84
1.82
1.81
1.80
1.79
1.77
1.64
1.62
1.61
1.59
1.57
1.55
1.36
1.35
1.34
1.33
1.31
1.30
1.29
1.28
1.26
1.25
1.24
1.21
1.19
0.87
0.86
0.84
0.00
1.01
1.02
0.97
3.00
1.00
1.01
1.06
4.03
6.00
6.07
3.02
f1 (ppm)
BPin
Bu
NC
3q

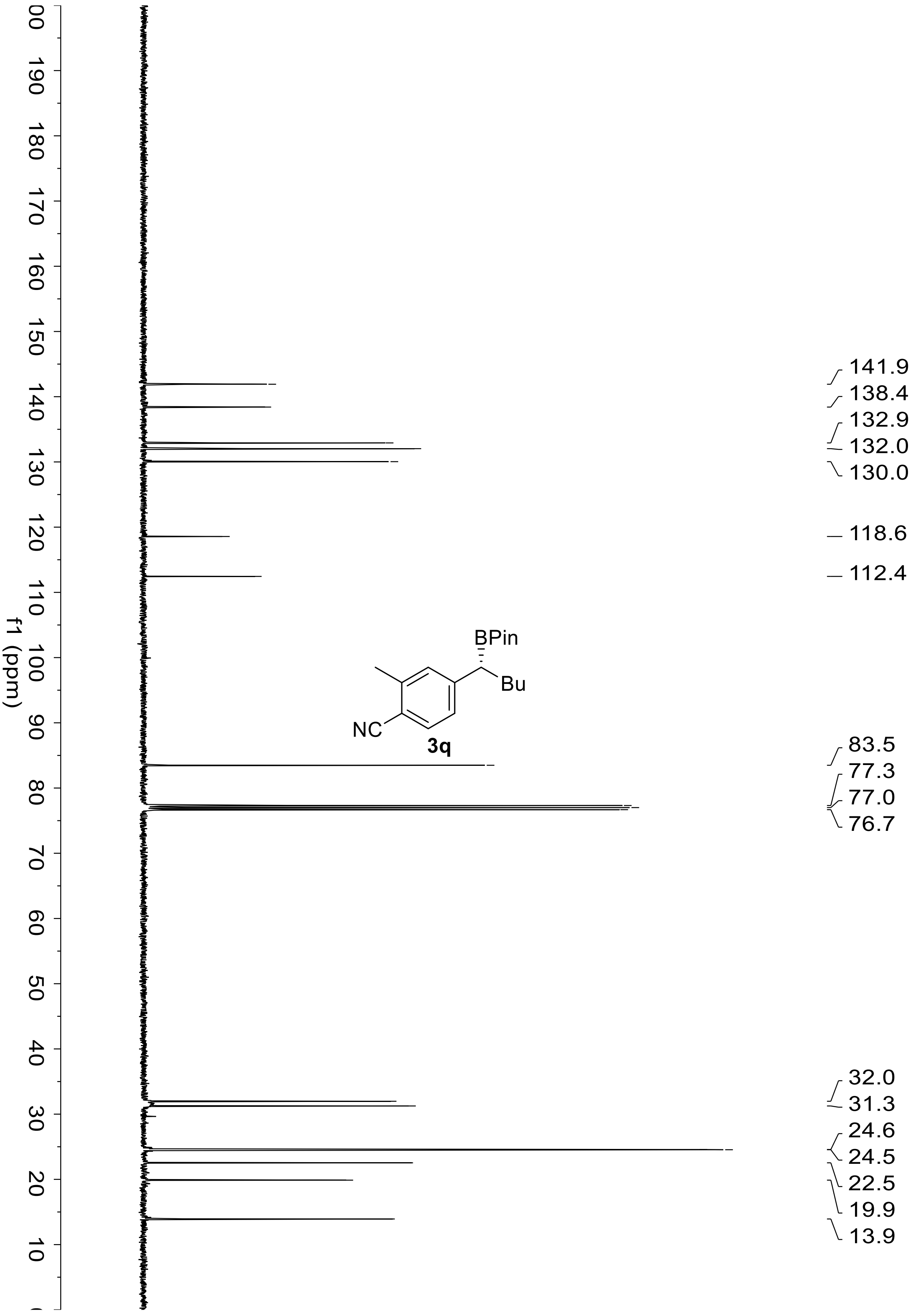
141.9
138.4
132.9
132.0
130.0
118.6
112.4
BPin
Bu
NC
3q
83.5
77.3
77.0
76.7
32.0
31.3
24.6
24.5
22.5
19.9
13.9
f1 (ppm)

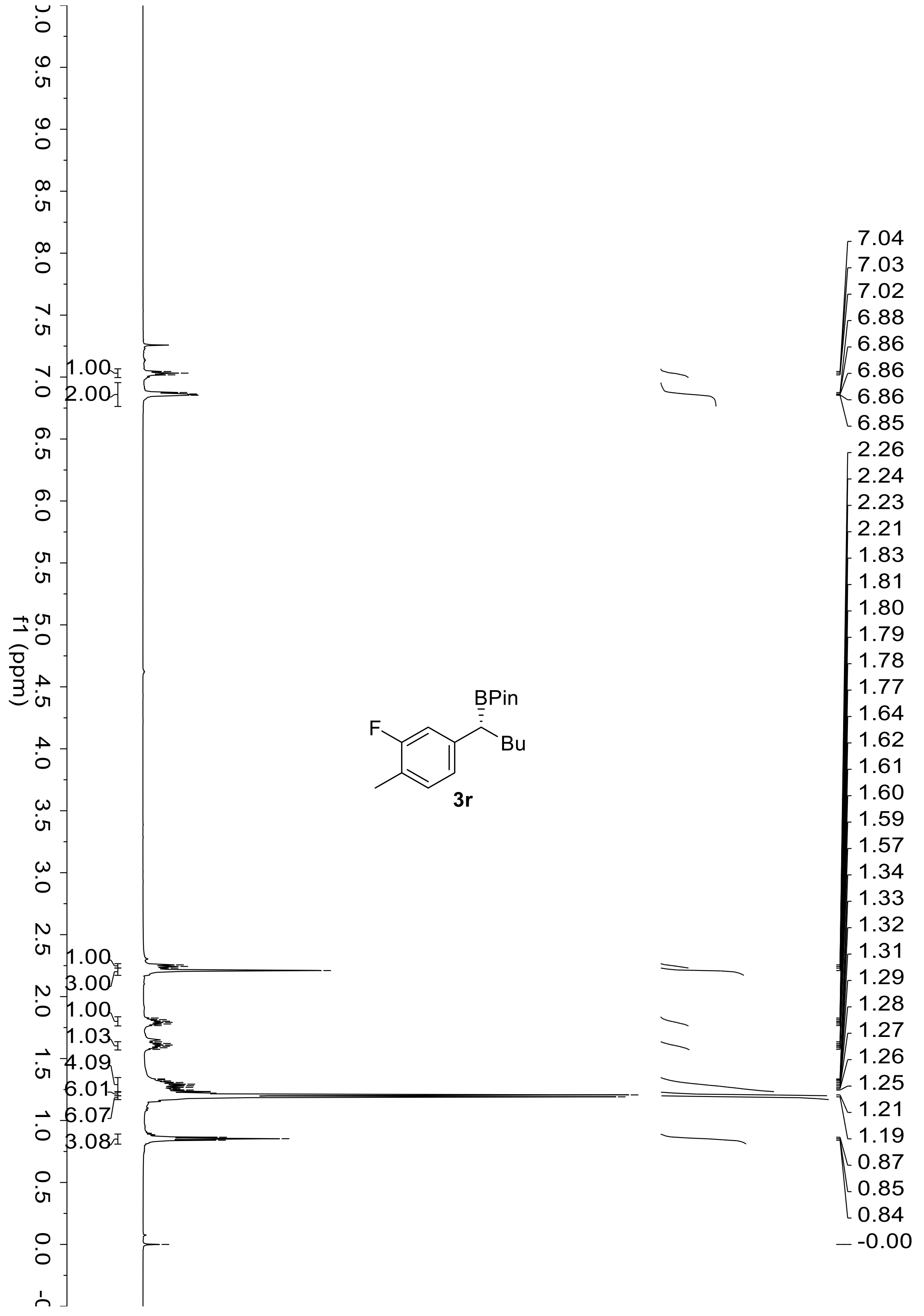
7.04
7.03
7.02
6.88
6.86
6.86
6.86
6.85
2.26
2.24
2.23
2.21
1.83
1.81
1.80
1.79
1.78
1.77
1.64
1.62
1.61
1.60
1.59
1.57
1.34
1.33
1.32
1.31
1.29
1.28
1.27
1.26
1.25
1.21
1.19
0.87
0.85
0.84
-0.00
BPin
F
Bu
3r

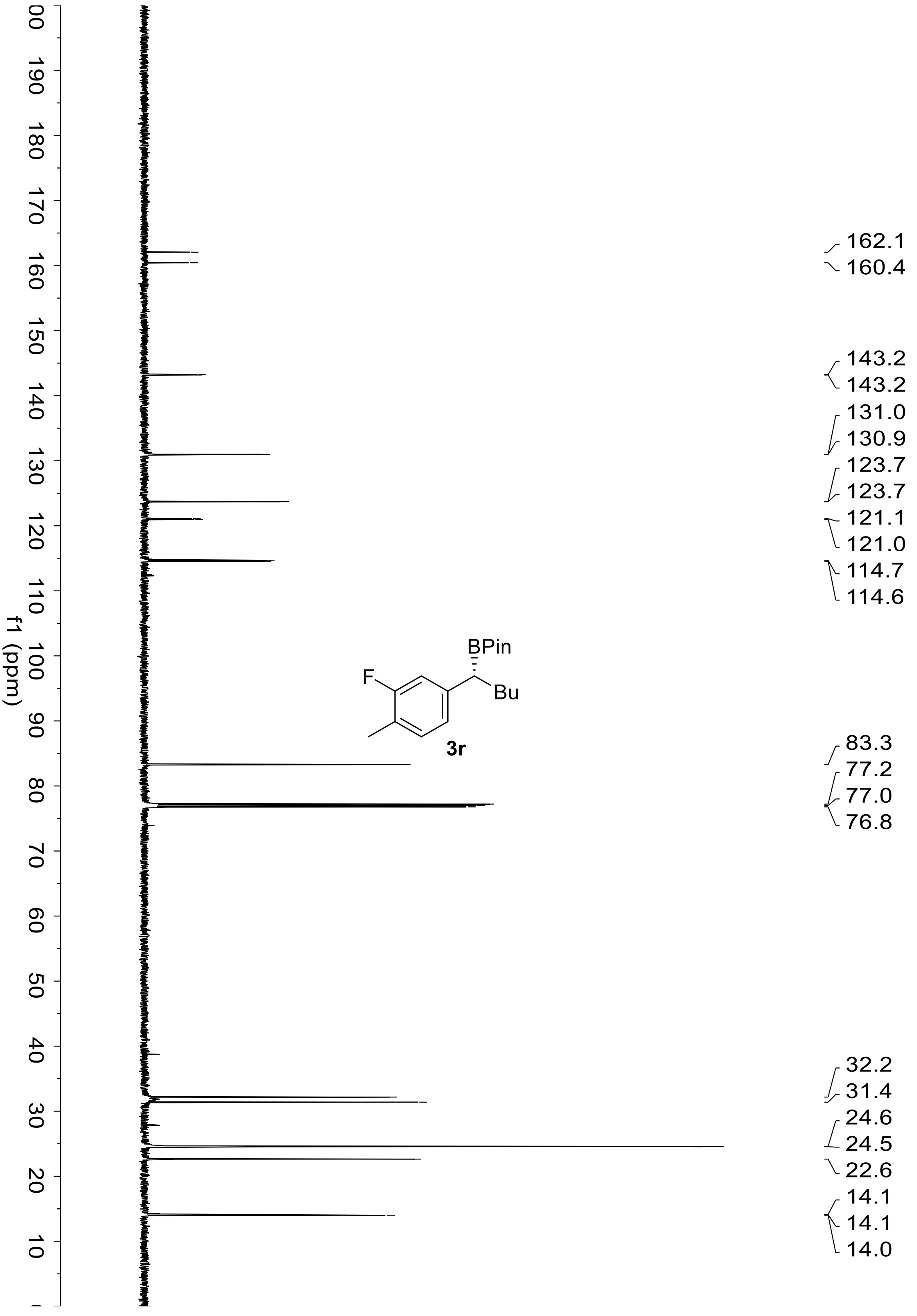
162.1
160.4
143.2
143.2
131.0
130.9
123.7
123.7
121.1
121.0
114.7
114.6
83.3
77.2
77.0
76.8
32.2
31.4
24.6
24.5
22.6
14.1
14.1
14.0
BPin
F
Bu
3r
f1 (ppm)

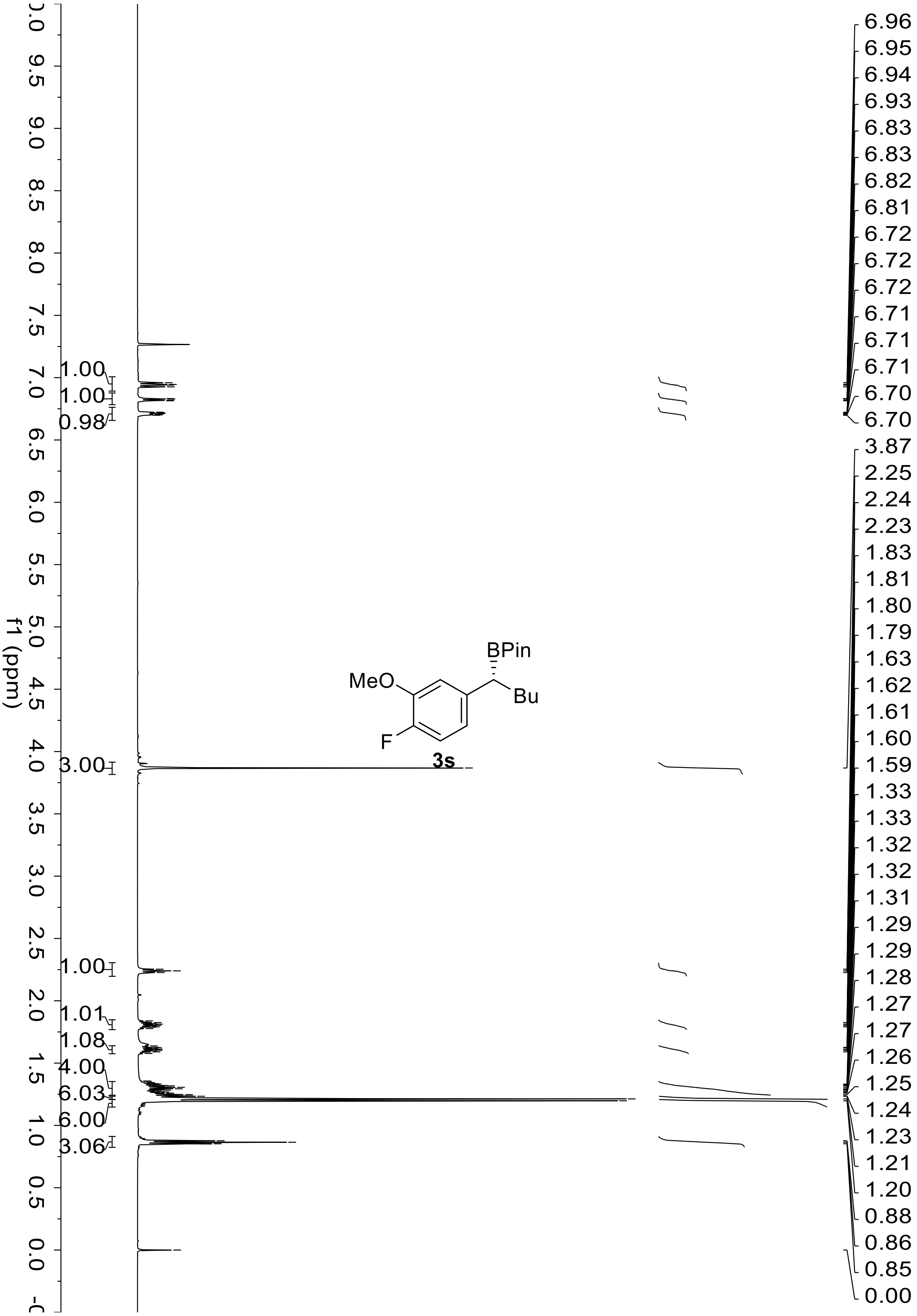

BPin
MeO
Bu
F
3s
f1 (ppm)
6.96
6.95
6.94
6.93
6.83
6.83
6.82
6.81
6.72
6.72
6.72
6.71
6.71
6.71
6.70
6.70
3.87
2.25
2.24
2.23
1.83
1.81
1.80
1.79
1.63
1.62
1.61
1.60
1.59
1.33
1.33
1.32
1.32
1.31
1.29
1.29
1.28
1.27
1.27
1.26
1.25
1.24
1.23
1.21
1.20
0.88
0.86
0.85
0.00
1.00
1.00
0.98
3.00
1.00
1.01
1.08
4.00
6.03
6.00
3.06

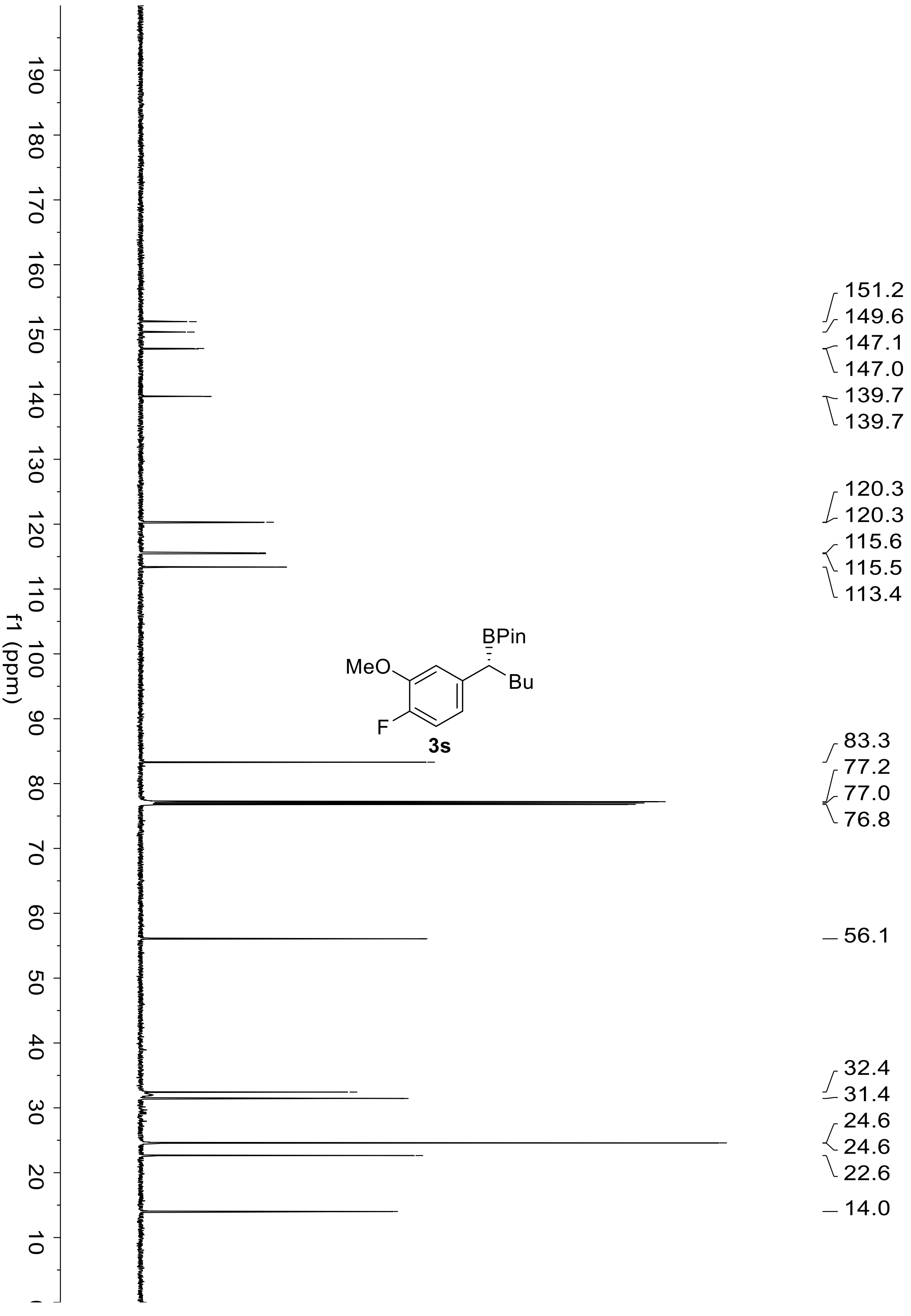

151.2
149.6
147.1
147.0
139.7
139.7
120.3
120.3
115.6
115.5
113.4
83.3
77.2
77.0
76.8
56.1
32.4
31.4
24.6
24.6
22.6
14.0
f1 (ppm)
BPin
MeO
Bu
F
3s

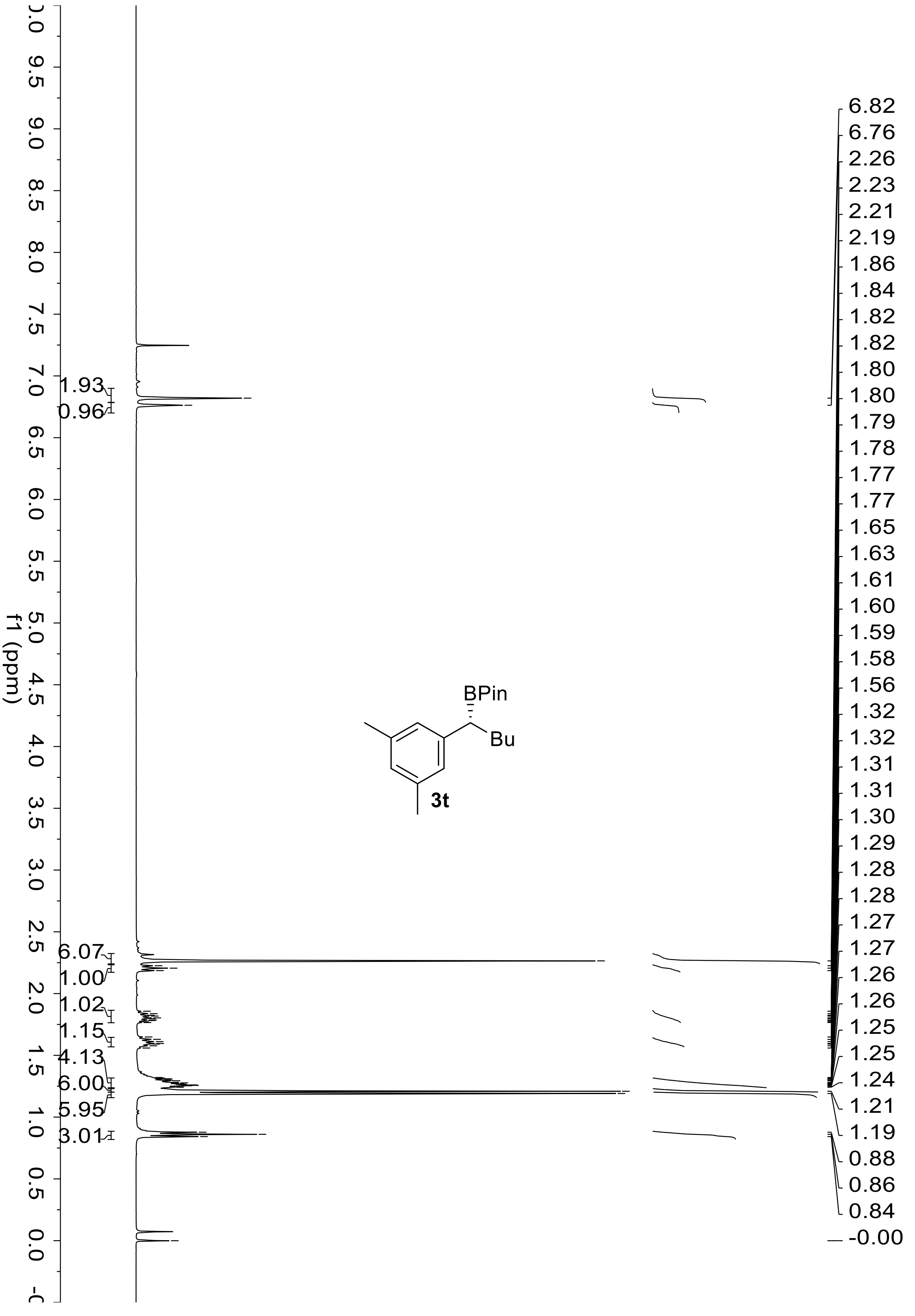
BPin
Bu
3t
f1 (ppm)
6.82
6.76
2.26
2.23
2.21
2.19
1.86
1.84
1.82
1.82
1.80
1.80
1.79
1.78
1.77
1.77
1.65
1.63
1.61
1.60
1.59
1.58
1.56
1.32
1.32
1.31
1.31
1.30
1.29
1.28
1.28
1.27
1.27
1.26
1.26
1.25
1.25
1.24
1.21
1.19
0.88
0.86
0.84
-0.00
1.93
0.96
6.07
1.00
1.02
1.15
4.13
6.00
5.95
3.01

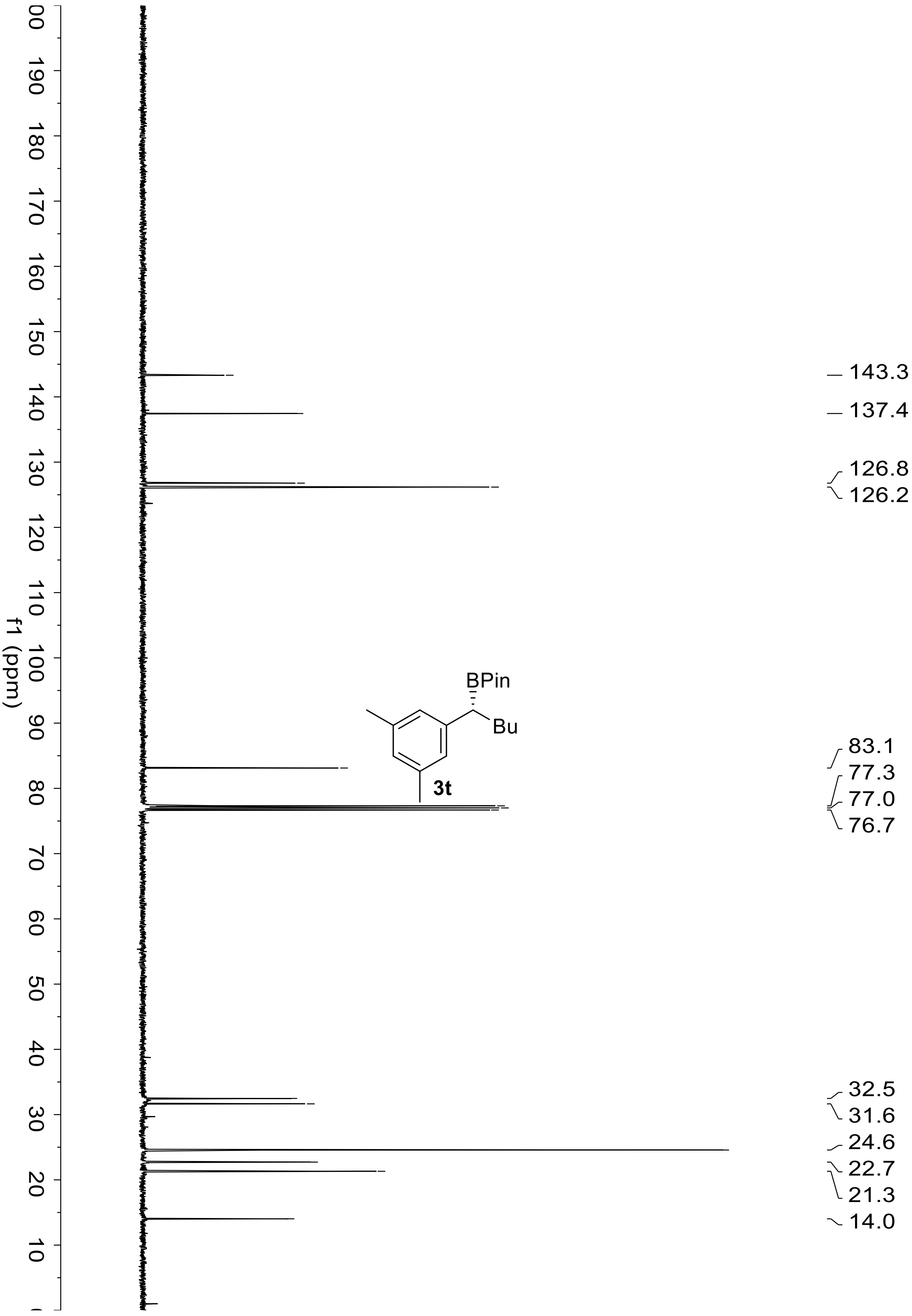
143.3
137.4
126.8
126.2
83.1
77.3
77.0
76.7
32.5
31.6
24.6
22.7
21.3
14.0
BPin
Bu
3t
f1 (ppm)

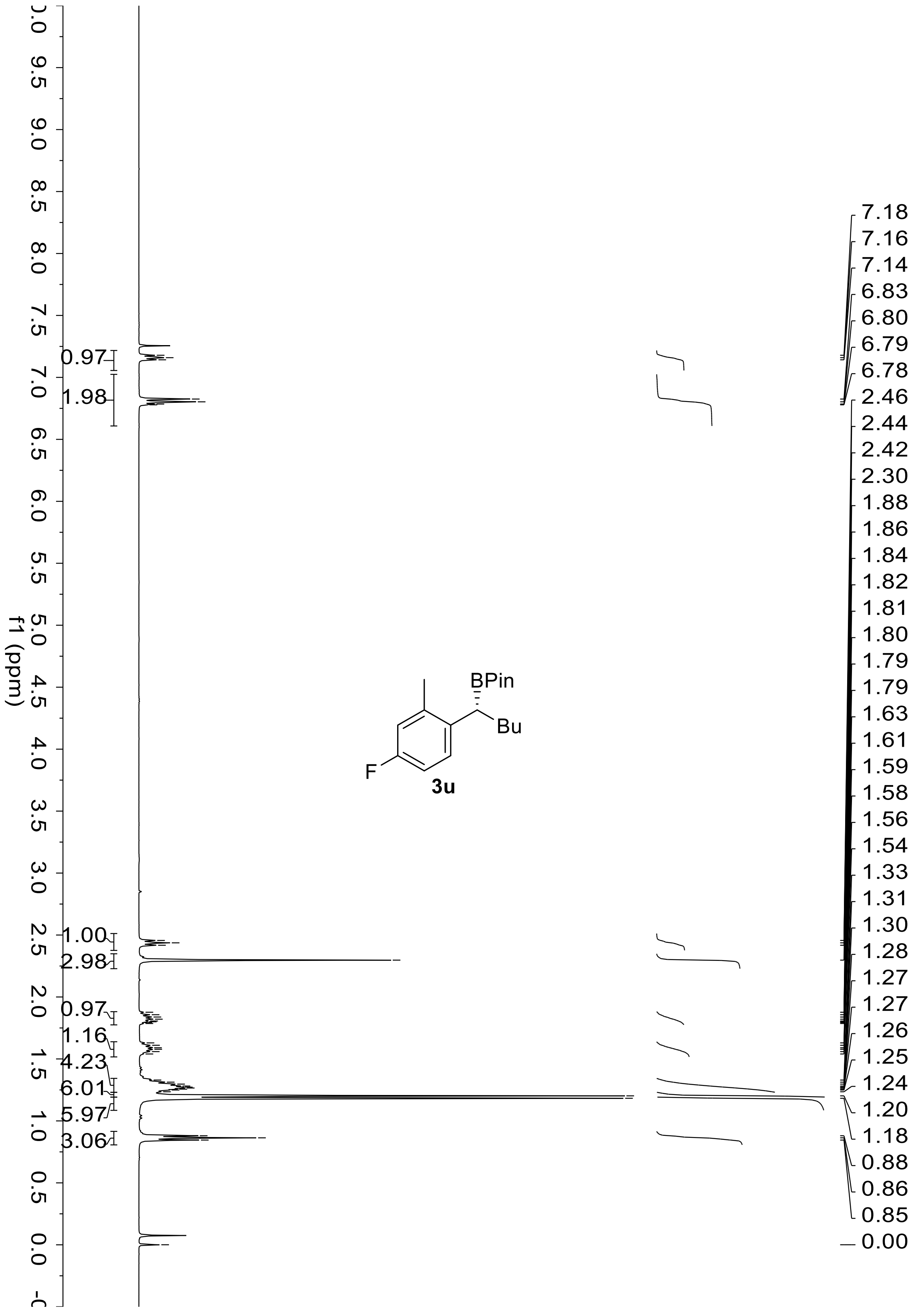
BPin
Bu
F
3u
f1 (ppm)
7.18
7.16
7.14
6.83
6.80
6.79
6.78
2.46
2.44
2.42
2.30
1.88
1.86
1.84
1.82
1.81
1.80
1.79
1.79
1.63
1.61
1.59
1.58
1.56
1.54
1.33
1.31
1.30
1.28
1.27
1.27
1.26
1.25
1.24
1.20
1.18
0.88
0.86
0.85
0.00
0.97
1.98
1.00
2.98
0.97
1.16
4.23
6.01
5.97
3.06

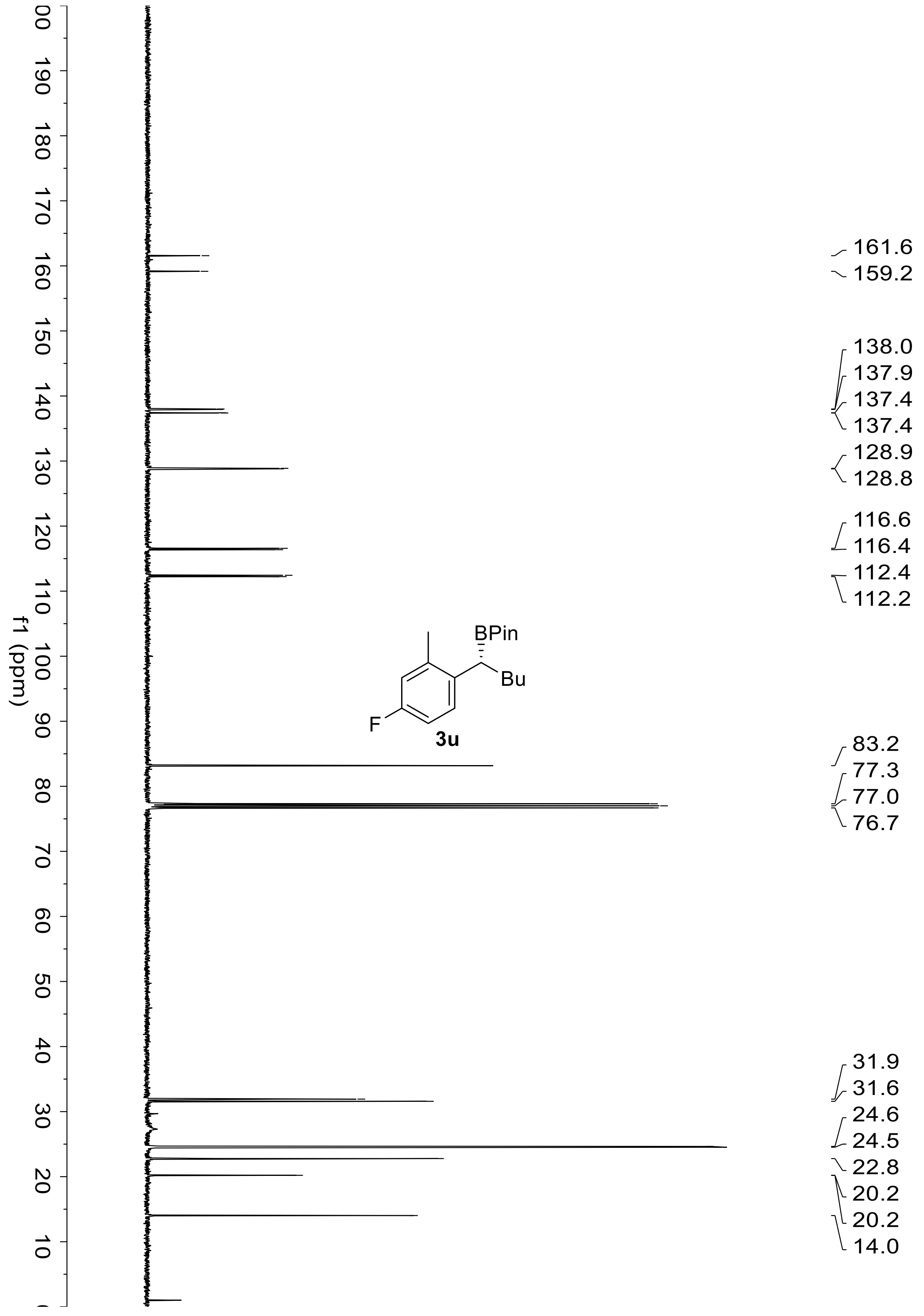
161.6
159.2
138.0
137.9
137.4
137.4
128.9
128.8
116.6
116.4
112.4
112.2
83.2
77.3
77.0
76.7
31.9
31.6
24.6
24.5
22.8
20.2
20.2
14.0
BPin
Bu
F
3u
f1 (ppm)

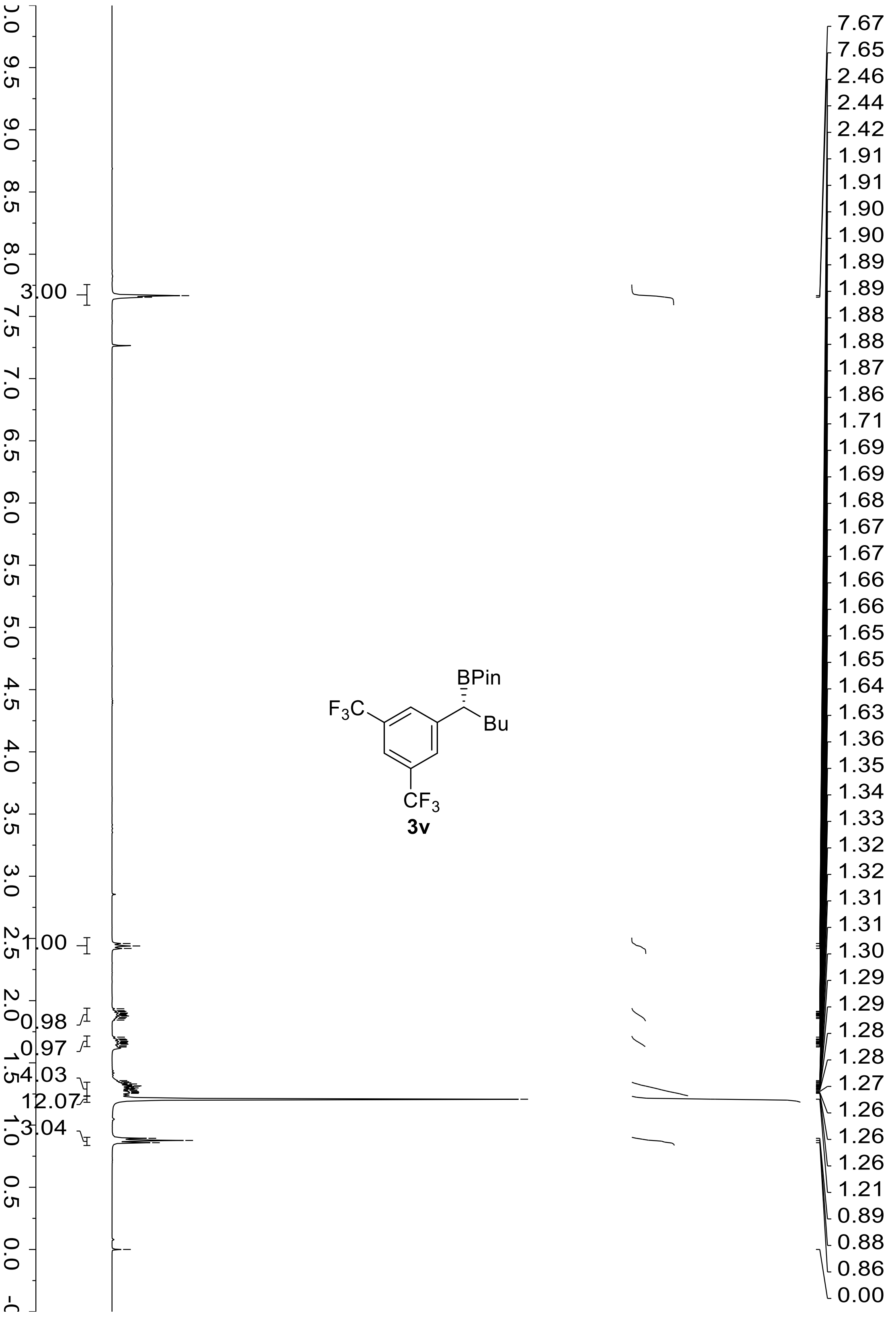

7.67
7.65
2.46
2.44
2.42
1.91
1.91
1.90
1.90
1.89
1.89
1.88
1.88
1.87
1.86
1.71
1.69
1.69
1.68
1.67
1.67
1.66
1.66
1.65
1.65
1.64
1.63
1.36
1.35
1.34
1.33
1.32
1.32
1.31
1.31
1.30
1.29
1.29
1.28
1.28
1.27
1.26
1.26
1.26
1.21
0.89
0.88
0.86
0.00
3.00
1.00
0.98
0.97
4.03
12.07
3.04
f1 (ppm)
BPin
Bu
F_3C
CF_3
3v

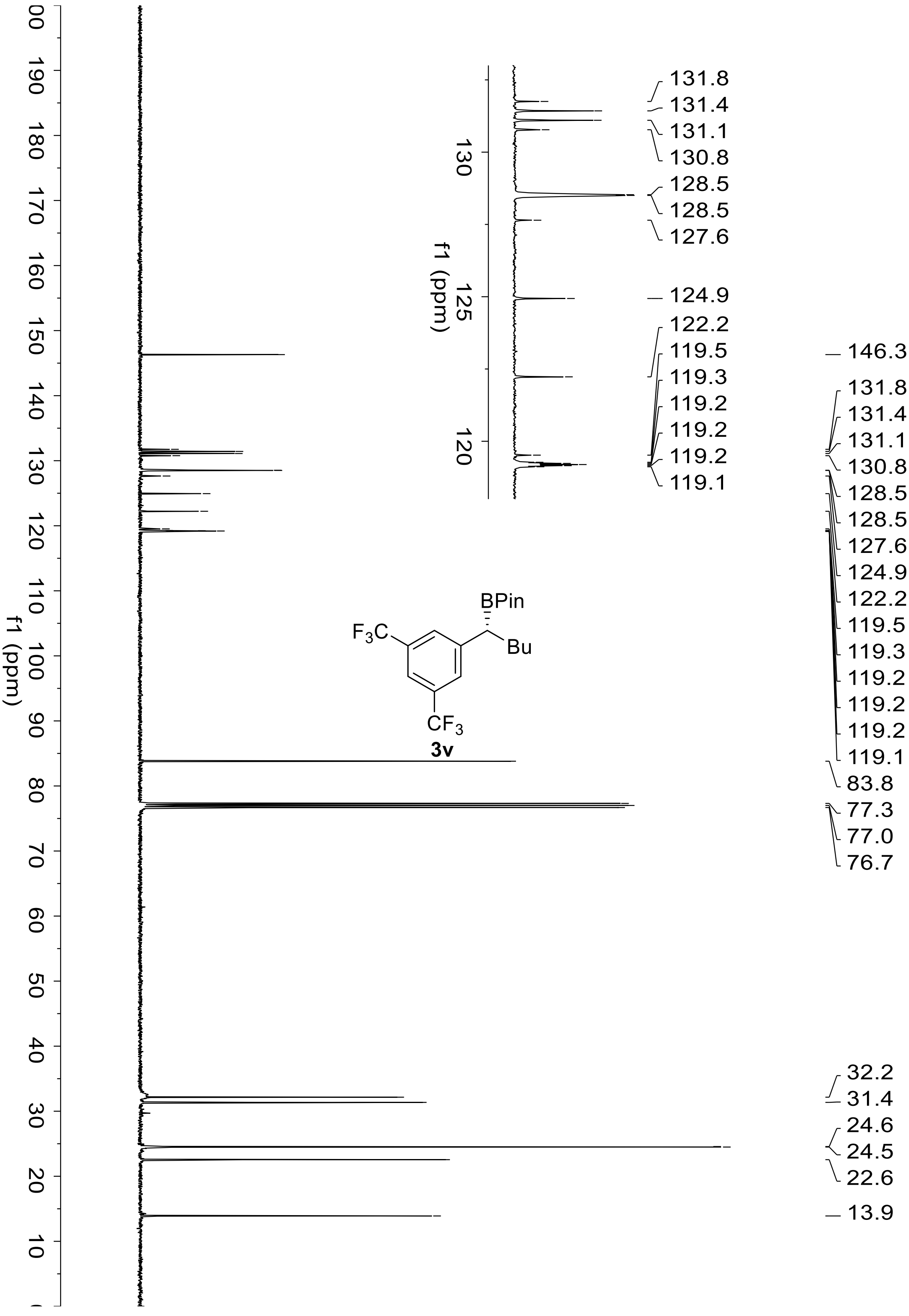
131.8
131.4
131.1
130.8
128.5
128.5
127.6
124.9
122.2
119.5
119.3
119.2
119.2
119.2
119.1
f1 (ppm)
146.3
131.8
131.4
131.1
130.8
128.5
128.5
127.6
124.9
122.2
119.5
119.3
119.2
119.2
119.2
119.1
83.8
77.3
77.0
76.7
32.2
31.4
24.6
24.5
22.6
13.9
BPin
Bu
F_3C
CF_3
3v
f1 (ppm)

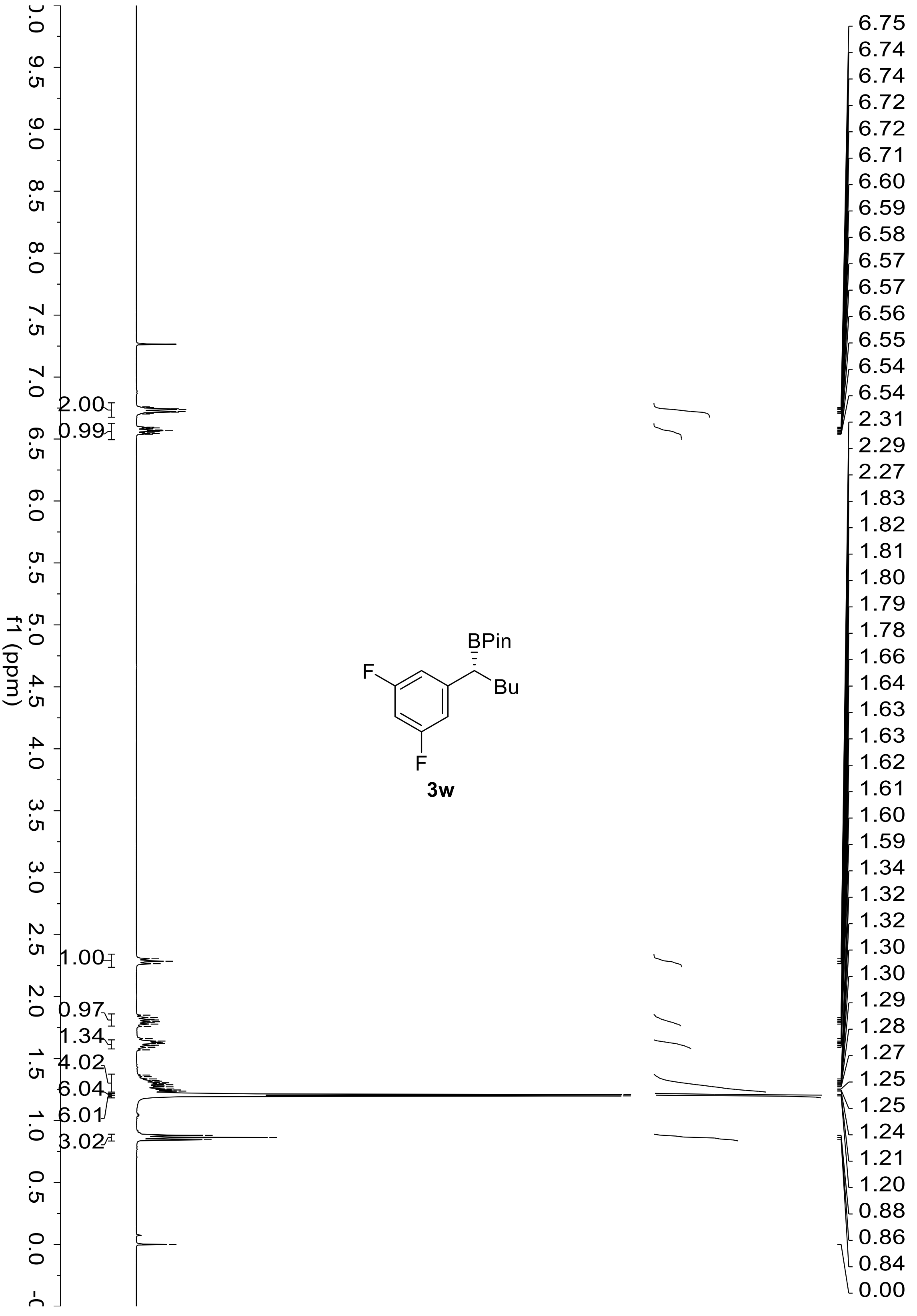
6.75
6.74
6.74
6.72
6.72
6.71
6.60
6.59
6.58
6.57
6.57
6.56
6.55
6.54
6.54
2.31
2.29
2.27
1.83
1.82
1.81
1.80
1.79
1.78
1.66
1.64
1.63
1.63
1.62
1.61
1.60
1.59
1.34
1.32
1.32
1.30
1.30
1.29
1.28
1.27
1.25
1.25
1.24
1.21
1.20
0.88
0.86
0.84
0.00
2.00
0.99
1.00
0.97
1.34
4.02
6.04
6.01
3.02
f1 (ppm)
BPin
Bu
F
F
3w

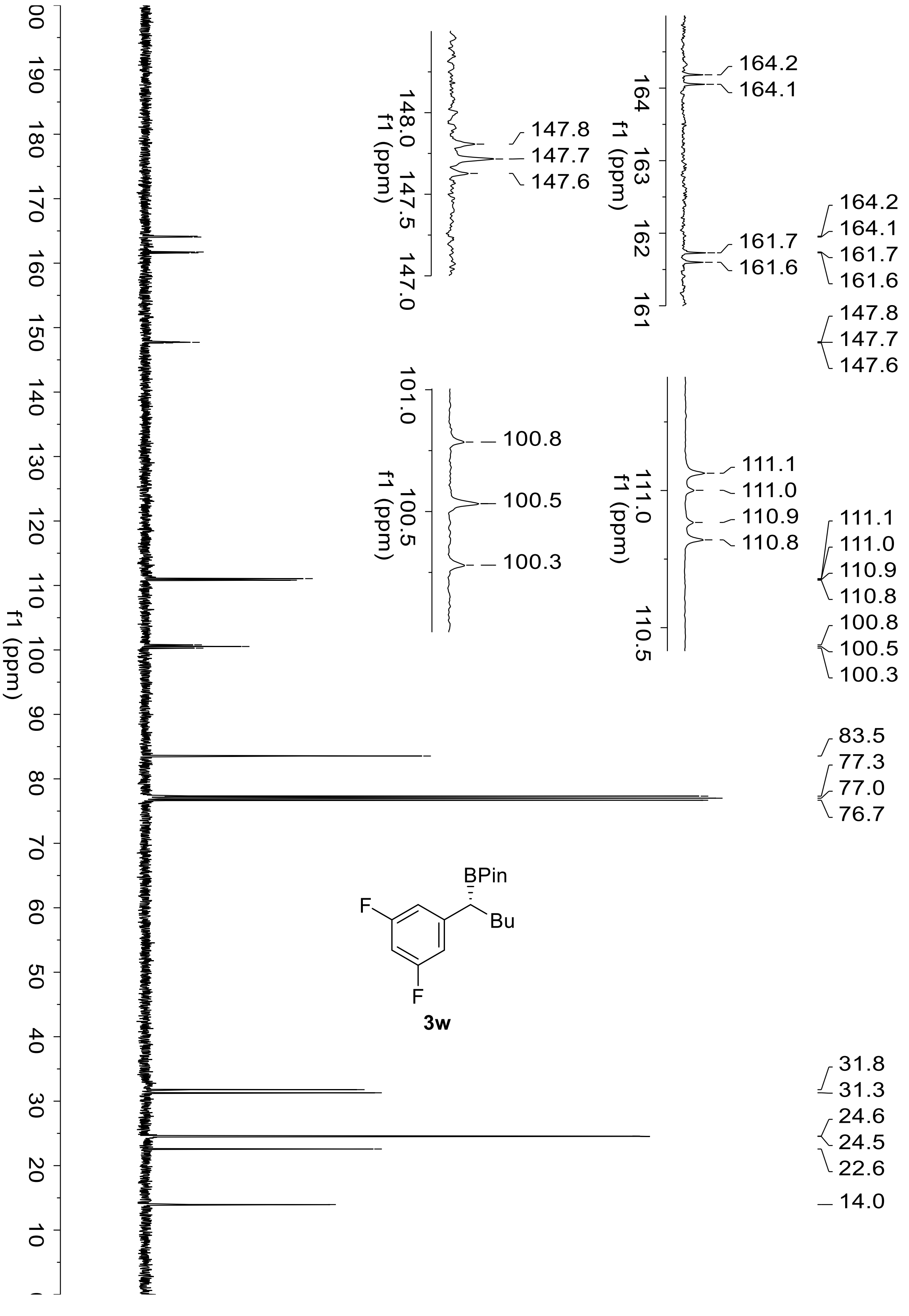

164.2
164.1
161.7
161.6
147.8
147.7
147.6
111.1
111.0
110.9
110.8
100.8
100.5
100.3
83.5
77.3
77.0
76.7
31.8
31.3
24.6
24.5
22.6
14.0
f1 (ppm)
BPin
Bu
F
F
3w

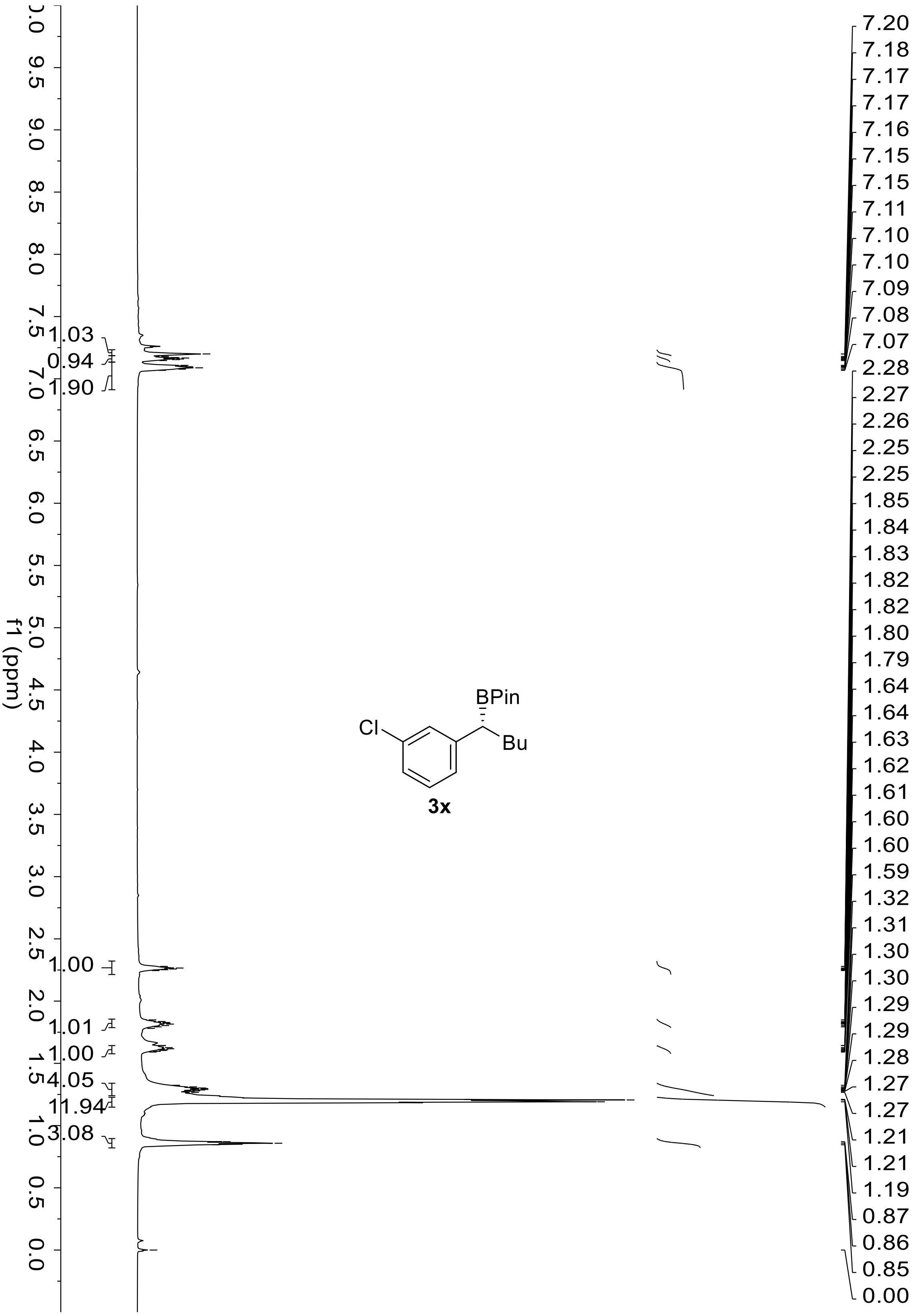

BPin
Cl
Bu
3x
f1 (ppm)

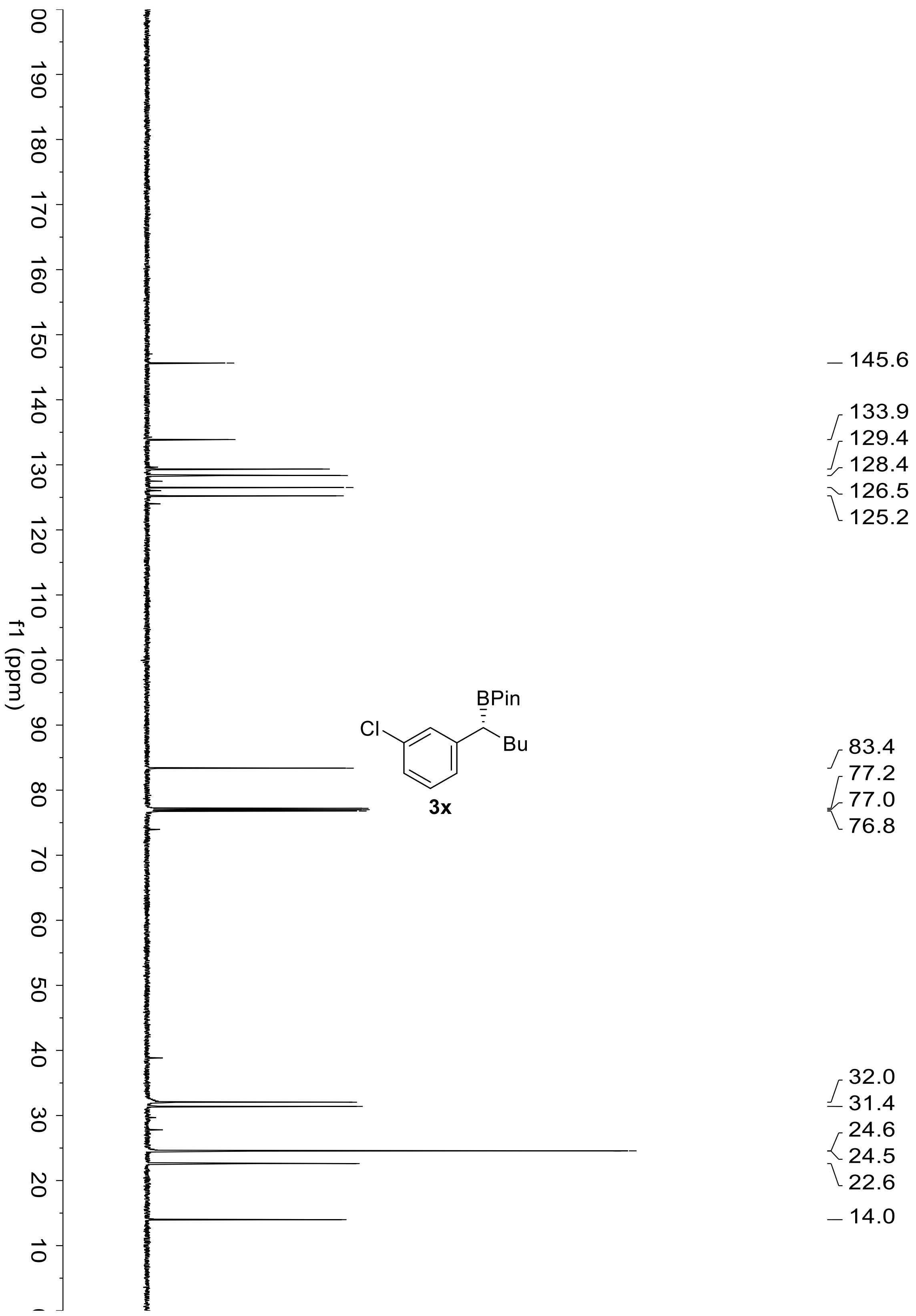
145.6
133.9
129.4
128.4
126.5
125.2
83.4
77.2
77.0
76.8
32.0
31.4
24.6
24.5
22.6
14.0
f1 (ppm)
BPin
Cl
Bu
3x

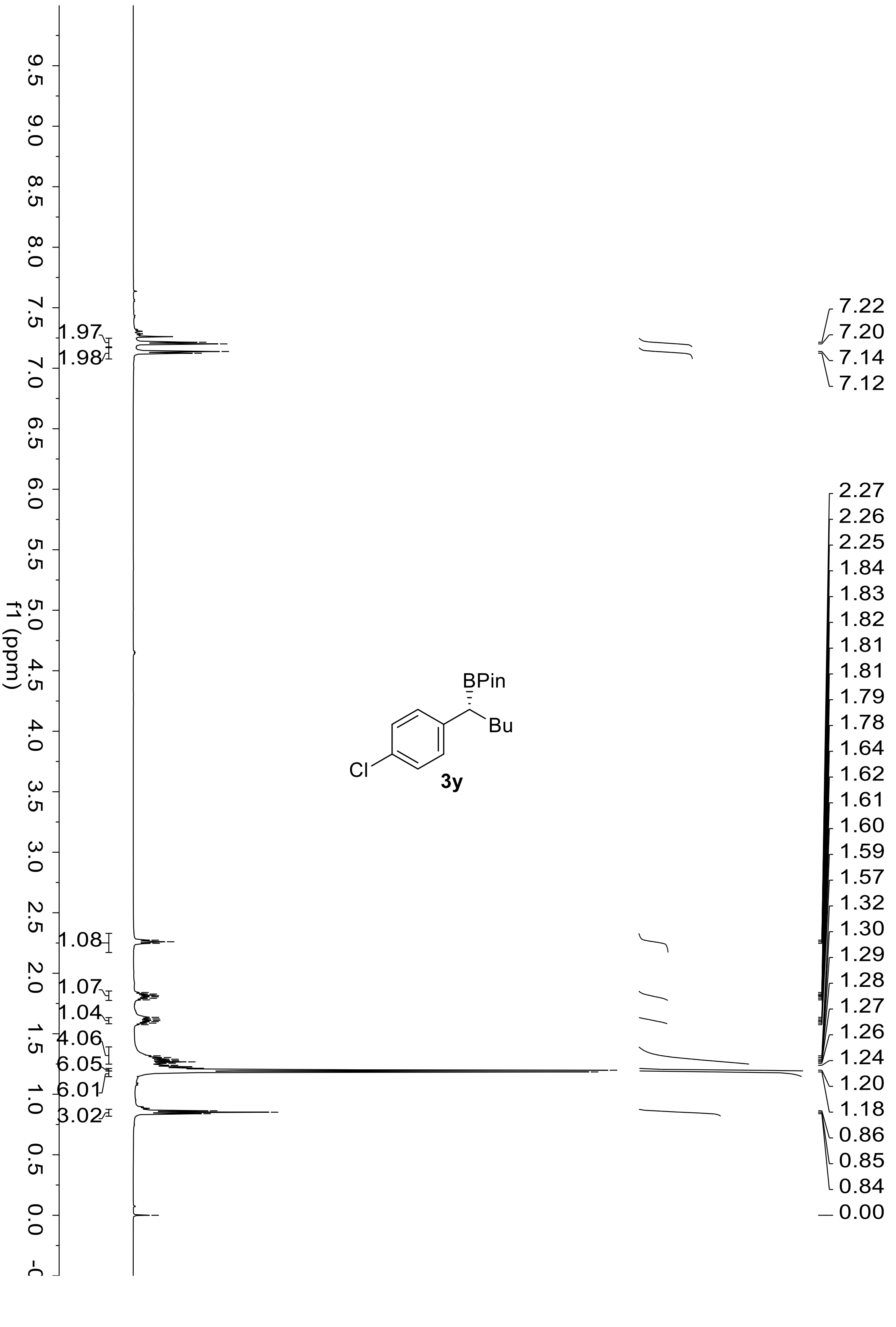
BPin
Bu
Cl
3y
f1 (ppm)
7.22
7.20
7.14
7.12
2.27
2.26
2.25
1.84
1.83
1.82
1.81
1.81
1.79
1.78
1.64
1.62
1.61
1.60
1.59
1.57
1.32
1.30
1.29
1.28
1.27
1.26
1.24
1.20
1.18
0.86
0.85
0.84
0.00
1.97
1.98
1.08
1.07
1.04
4.06
6.05
6.01
3.02

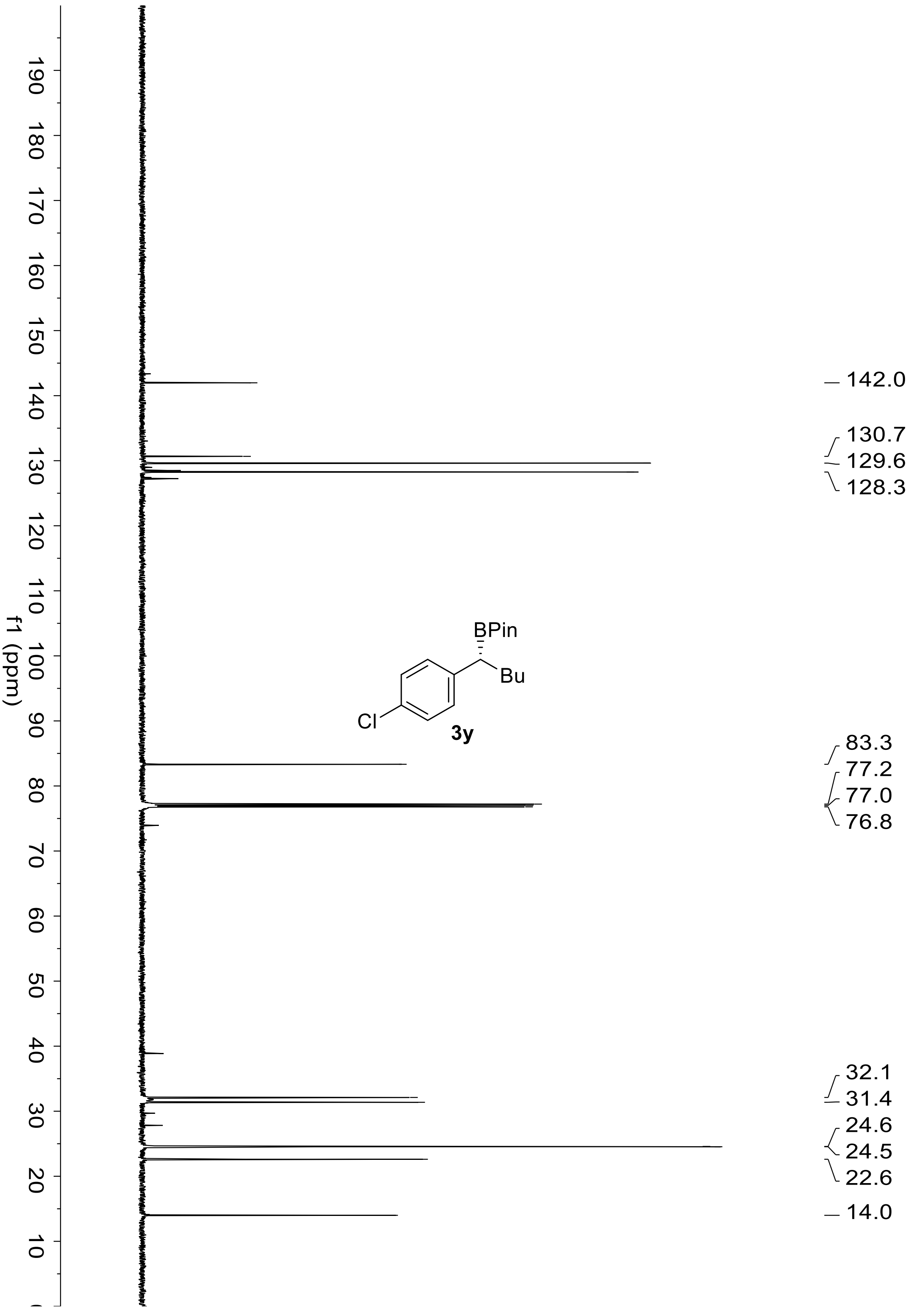
142.0
130.7
129.6
128.3
83.3
77.2
77.0
76.8
32.1
31.4
24.6
24.5
22.6
14.0
f1 (ppm)
BPin
Bu
Cl
3y

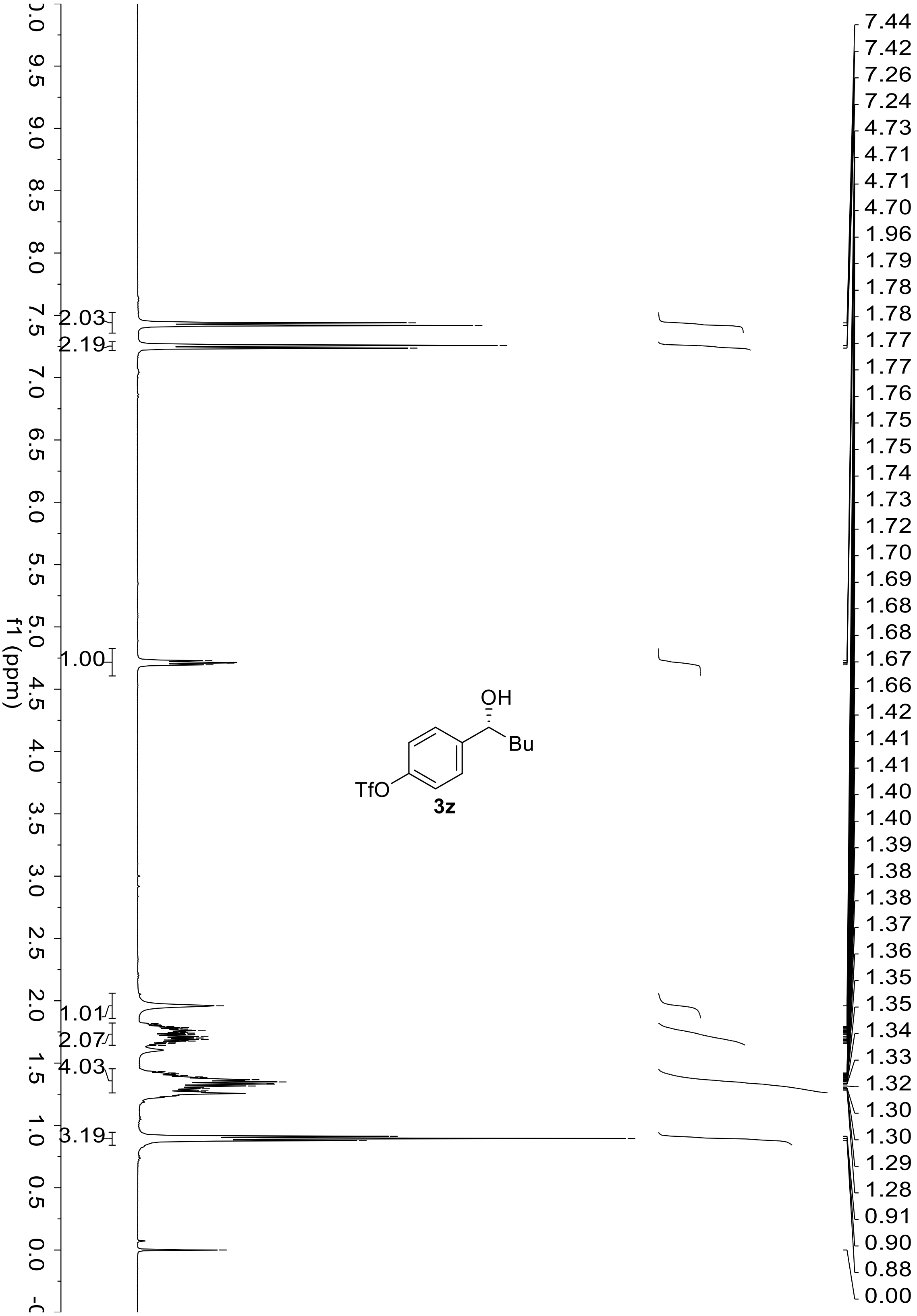

f1 (ppm)
2.03
2.19
1.00
1.01
2.07
4.03
3.19
OH
Bu
TfO
3z
7.44
7.42
7.26
7.24
4.73
4.71
4.71
4.70
1.96
1.79
1.78
1.78
1.77
1.77
1.76
1.75
1.75
1.74
1.73
1.72
1.70
1.69
1.68
1.68
1.67
1.66
1.42
1.41
1.41
1.40
1.40
1.39
1.38
1.38
1.37
1.36
1.35
1.35
1.34
1.33
1.32
1.30
1.30
1.29
1.28
0.91
0.90
0.88
0.00

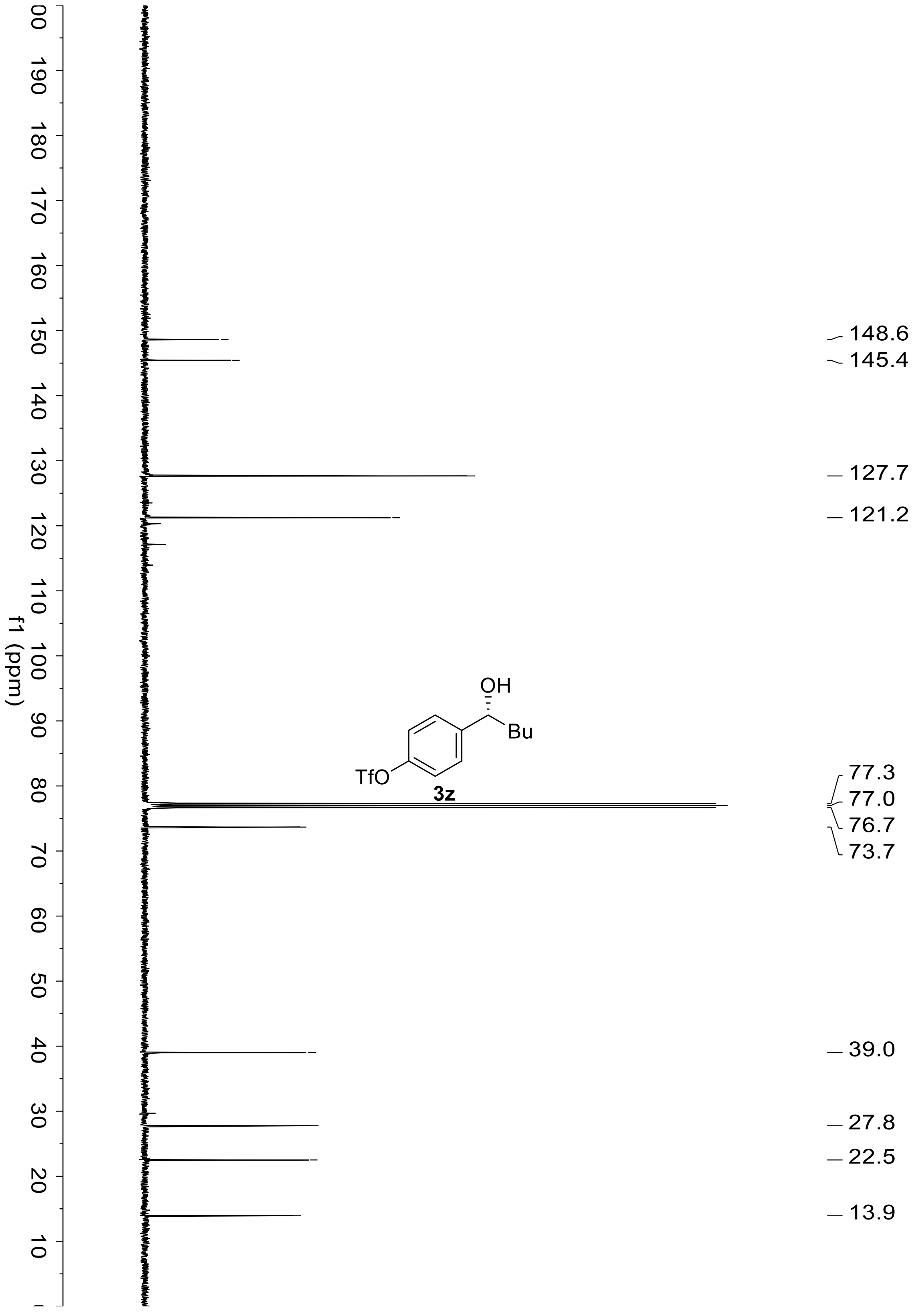
148.6
145.4
127.7
121.2
77.3
77.0
76.7
73.7
39.0
27.8
22.5
13.9
OH
Bu
TfO
3z
f1 (ppm)

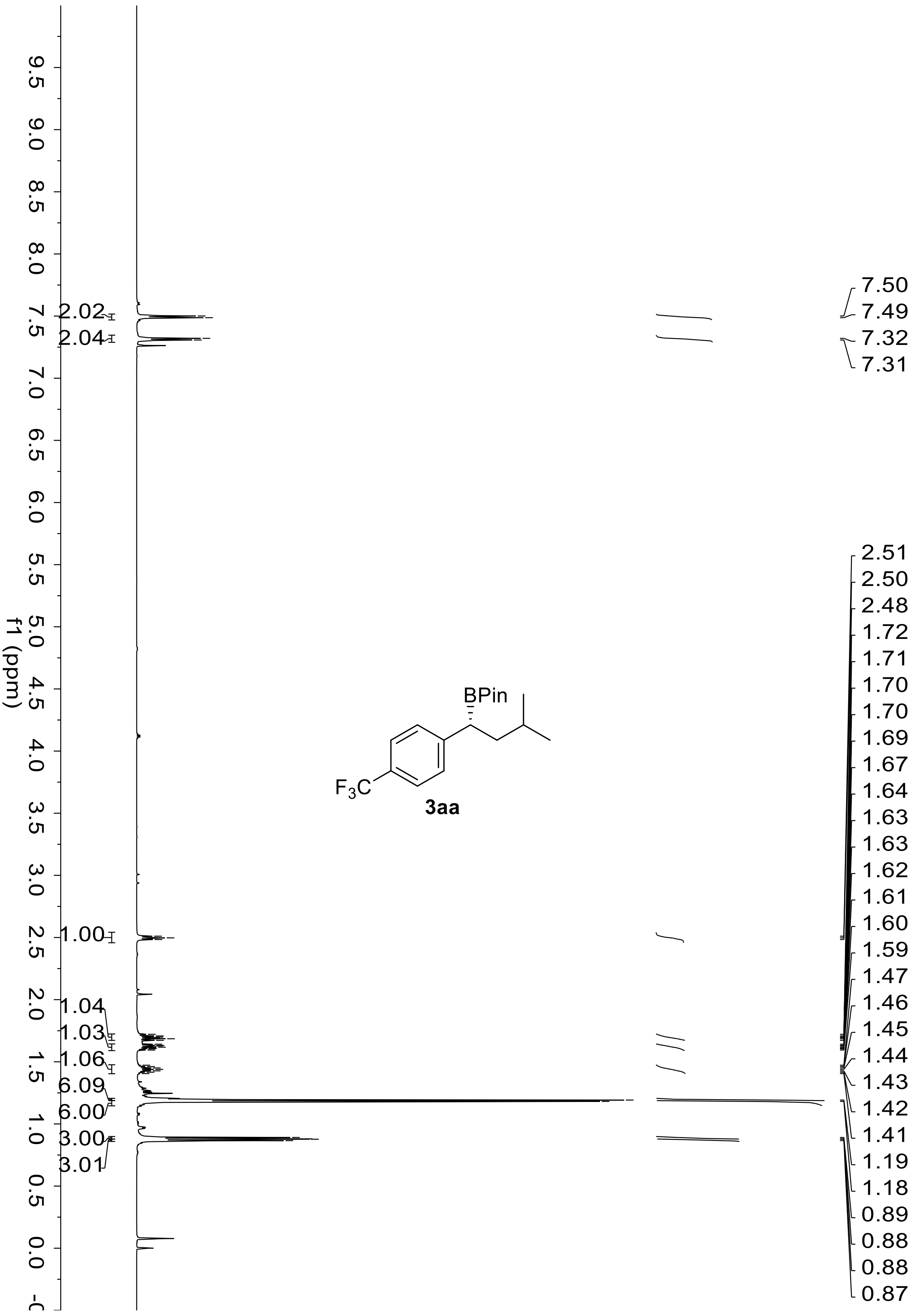

BPin
F3C
3aa
7.50
7.49
7.32
7.31
2.51
2.50
2.48
1.72
1.71
1.70
1.70
1.69
1.67
1.64
1.63
1.63
1.62
1.61
1.60
1.59
1.47
1.46
1.45
1.44
1.43
1.42
1.41
1.19
1.18
0.89
0.88
0.88
0.87
2.02
2.04
1.00
1.04
1.03
1.06
6.09
6.00
3.00
3.01
f1 (ppm)

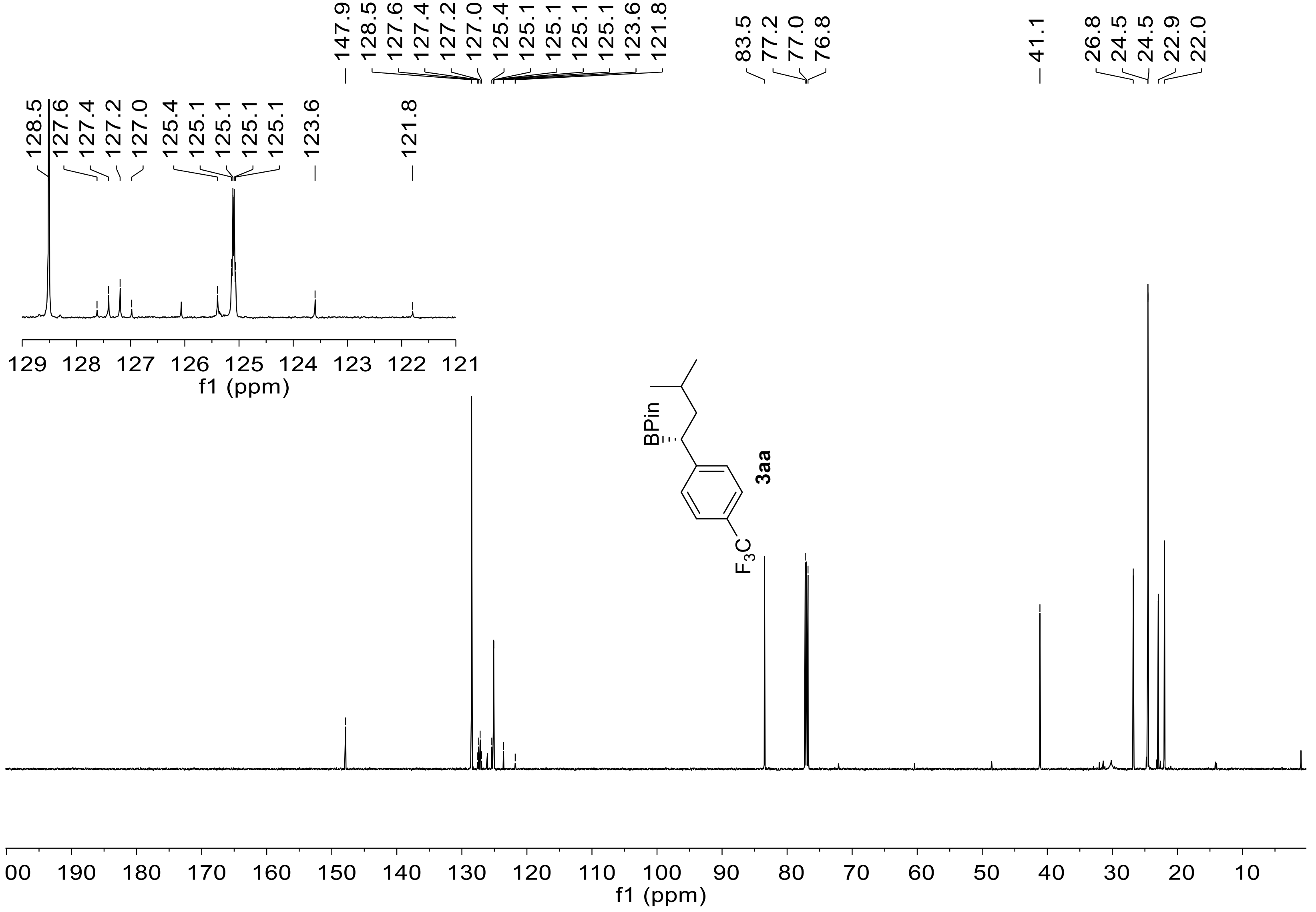

128.5
127.6
127.4
127.2
127.0
125.4
125.1
125.1
125.1
125.1
123.6
121.8
129 128 127 126 125 124 123 122 121
f1 (ppm)
147.9
128.5
127.6
127.4
127.2
127.0
125.4
125.1
125.1
125.1
125.1
123.6
121.8
83.5
77.2
77.0
76.8
41.1
26.8
24.5
24.5
22.9
22.0
BPin
F_3C
3aa
00 190 180 170 160 150 140 130 120 110 100 90 80 70 60 50 40 30 20 10
f1 (ppm)

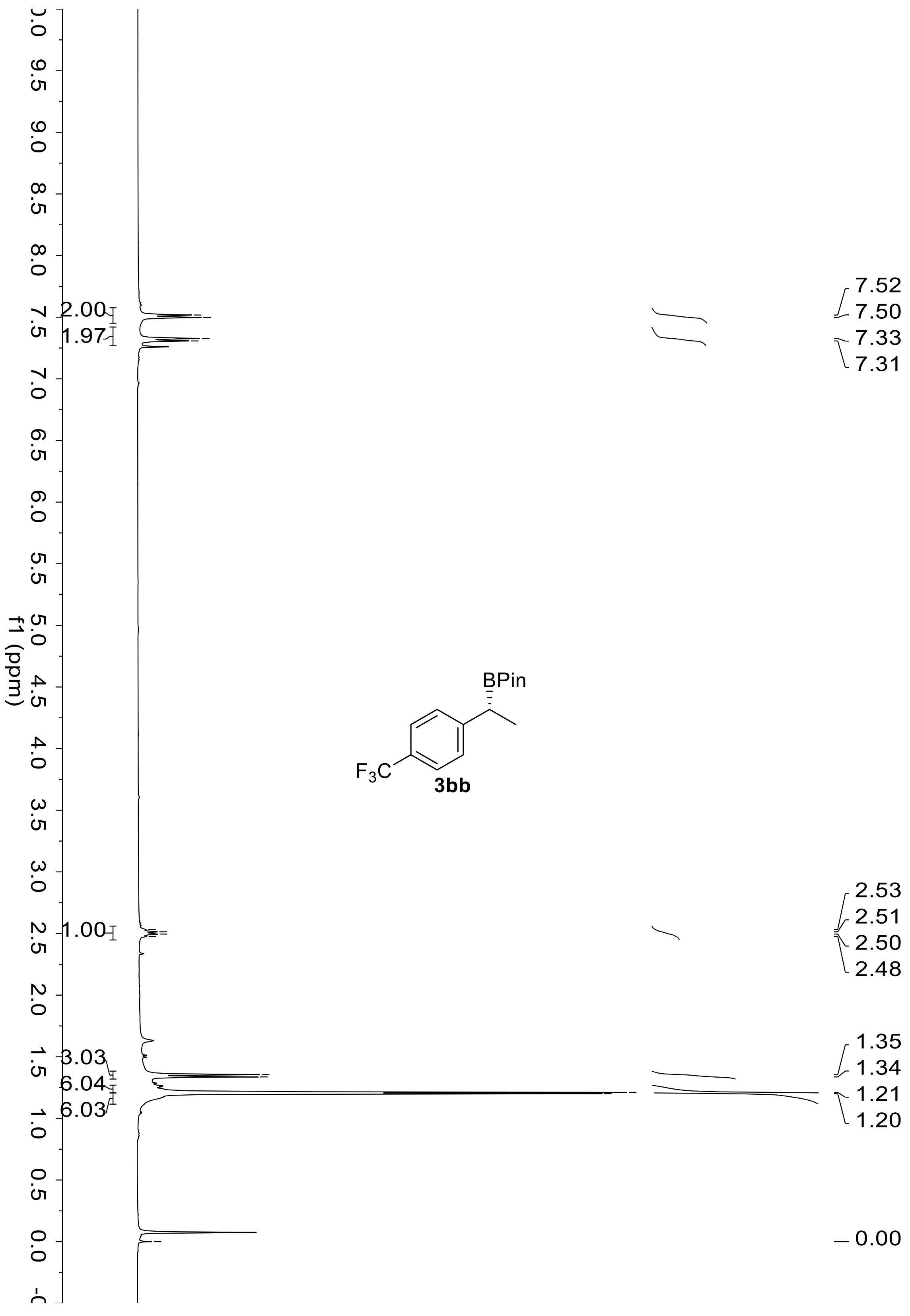
BPin
F3C
3bb
7.52
7.50
7.33
7.31
2.53
2.51
2.50
2.48
1.35
1.34
1.21
1.20
0.00
2.00
1.97
1.00
3.03
6.04
6.03
f1 (ppm)

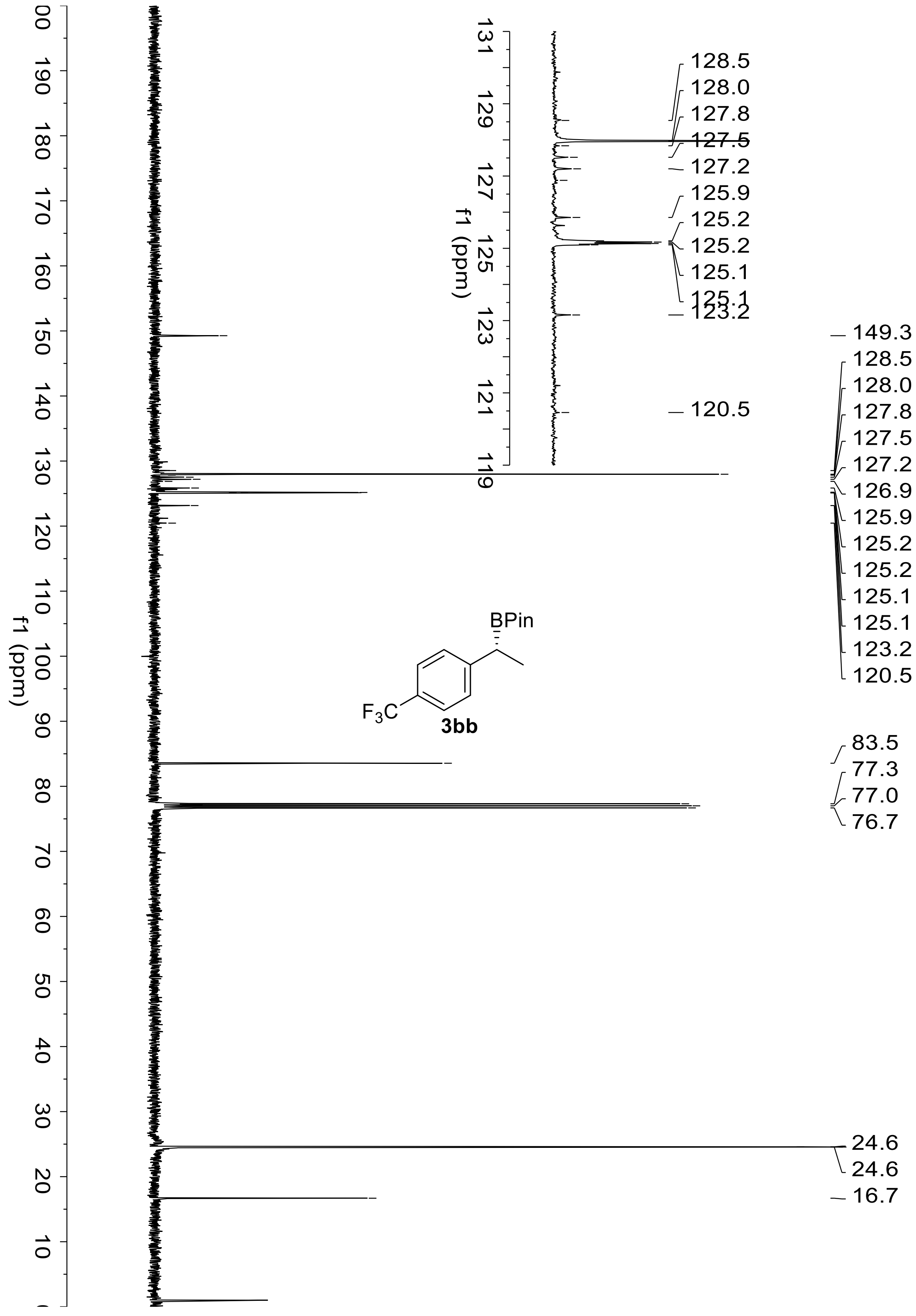
128.5
128.0
127.8
127.5
127.2
125.9
125.2
125.2
125.1
125.1
123.2
120.5
149.3
128.5
128.0
127.8
127.5
127.2
126.9
125.9
125.2
125.2
125.1
125.1
123.2
120.5
83.5
77.3
77.0
76.7
24.6
24.6
16.7
f1 (ppm)
BPin
F_3C
3bb

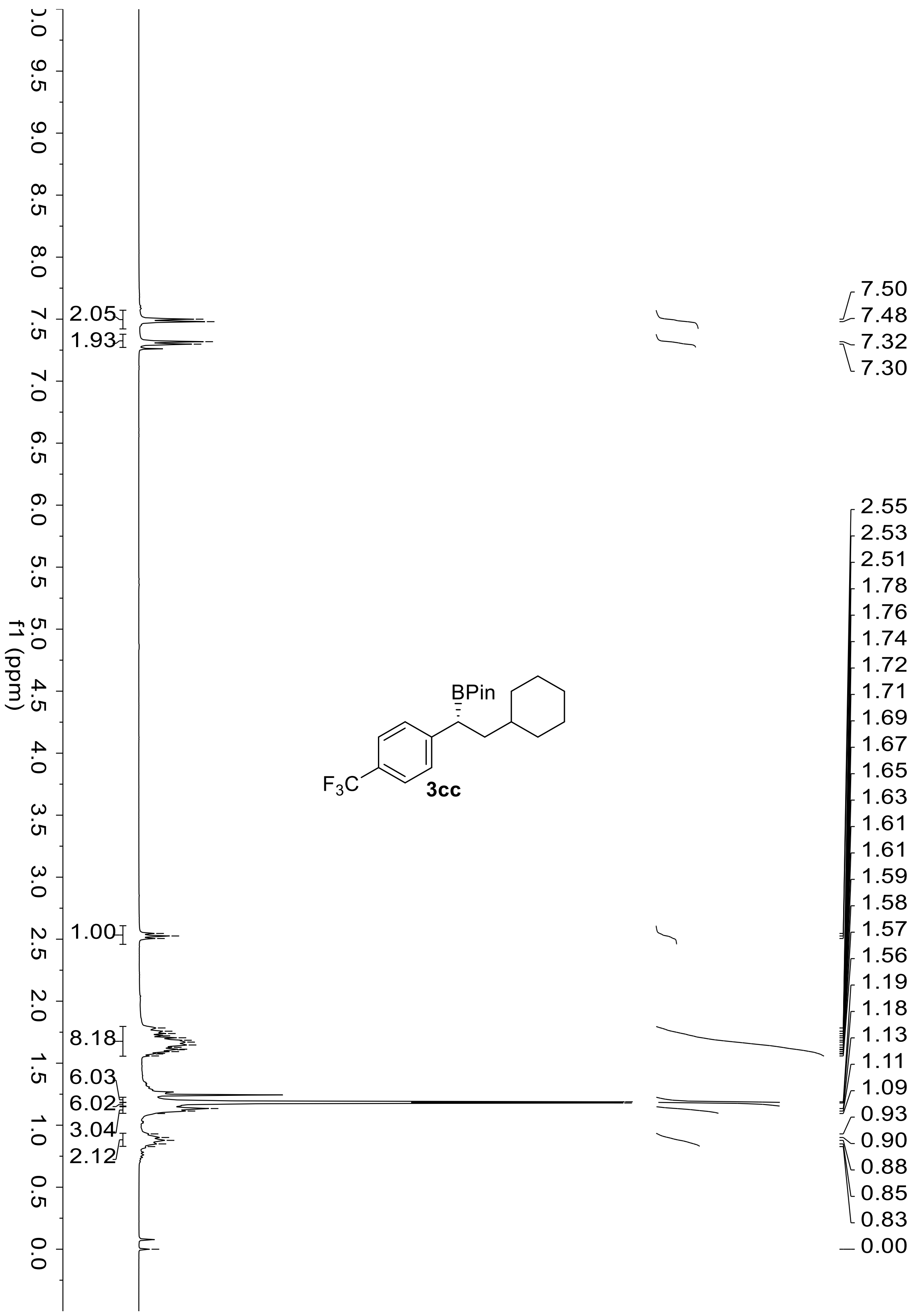
BPin
F3C
3cc
f1 (ppm)

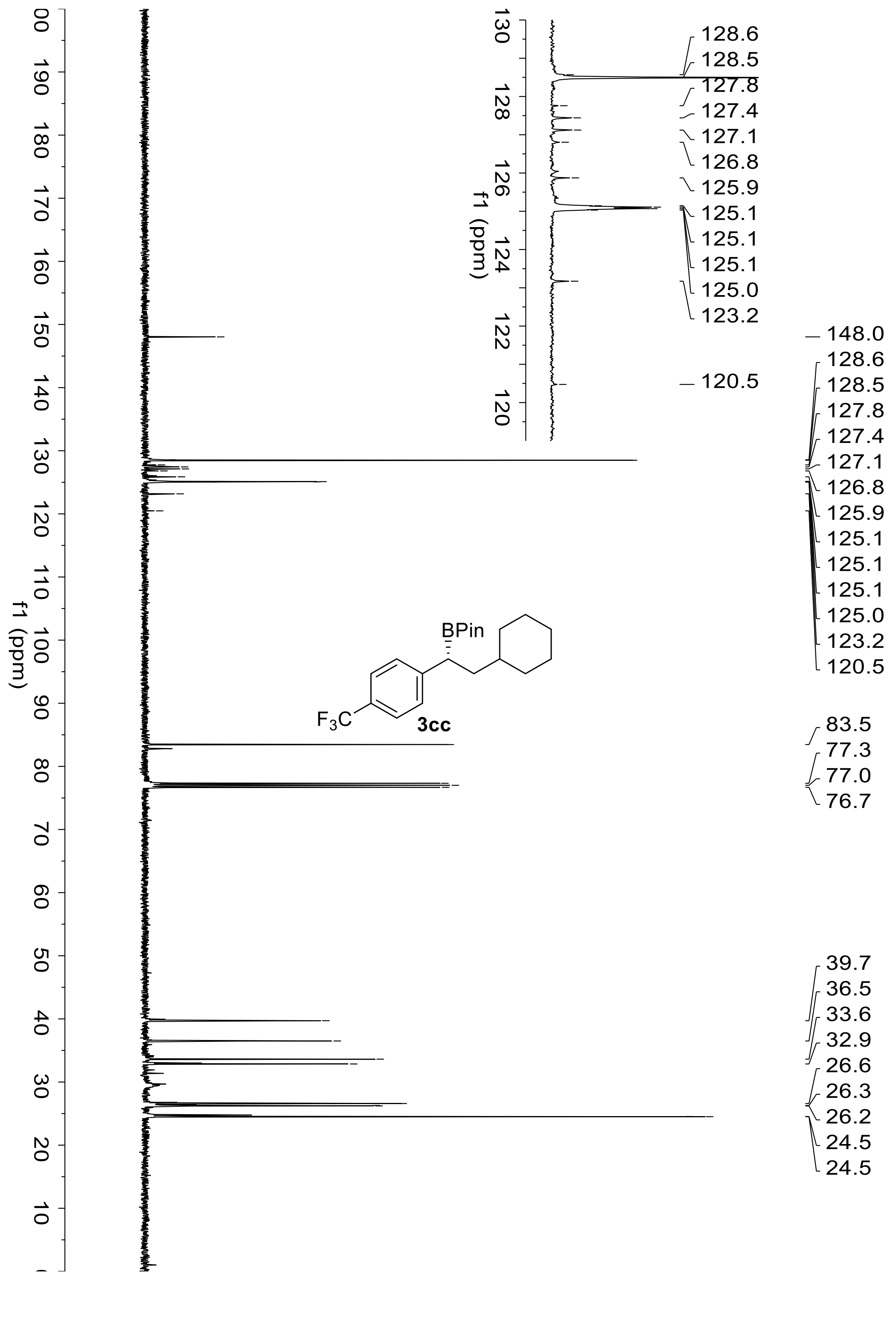

BPin
F$_3$C
3cc
128.6
128.5
127.8
127.4
127.1
126.8
125.9
125.1
125.1
125.1
125.0
123.2
120.5
148.0
128.6
128.5
127.8
127.4
127.1
126.8
125.9
125.1
125.1
125.1
125.0
123.2
120.5
83.5
77.3
77.0
76.7
39.7
36.5
33.6
32.9
26.6
26.3
26.2
24.5
24.5
f1 (ppm)

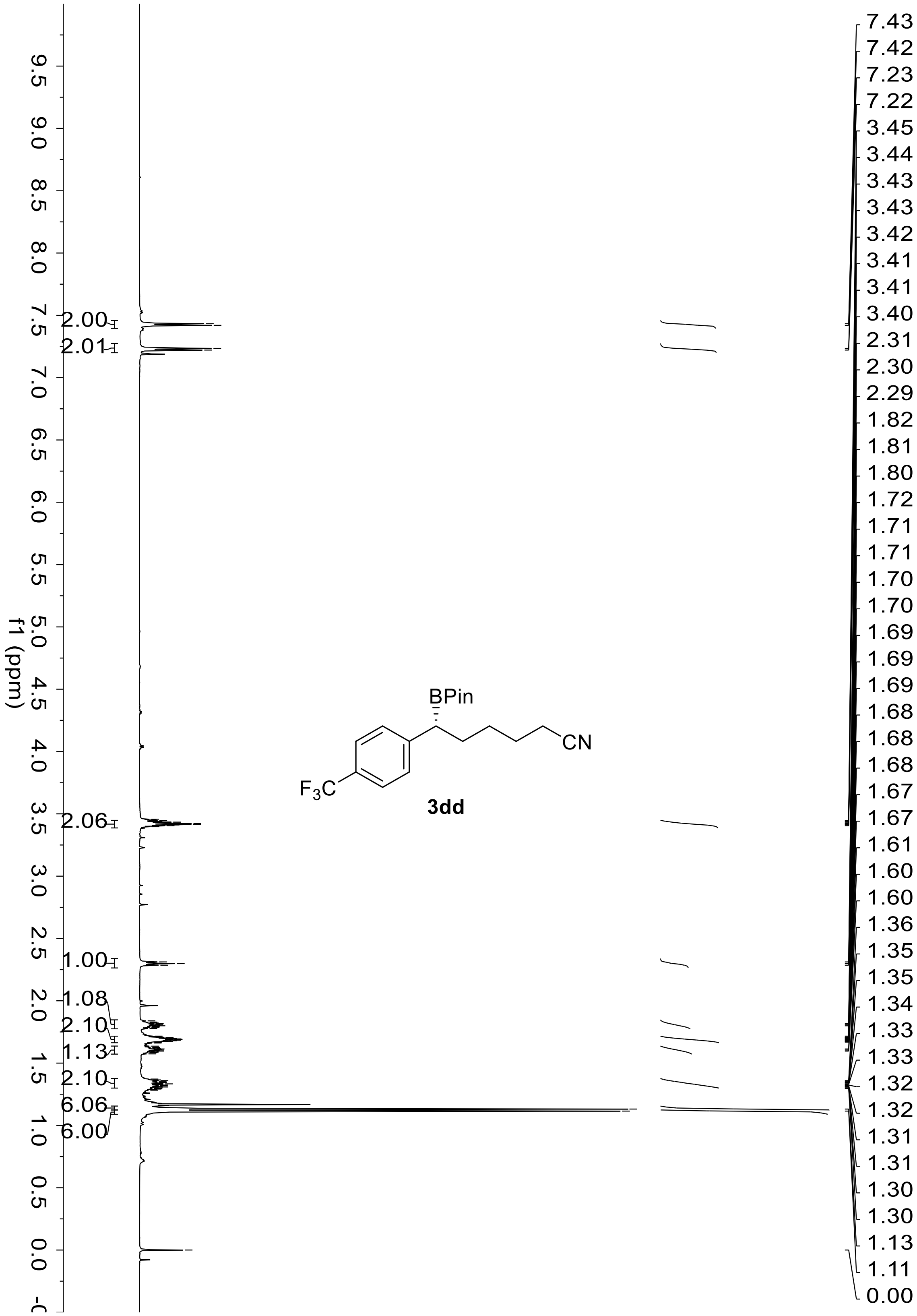
7.43
7.42
7.23
7.22
3.45
3.44
3.43
3.43
3.42
3.41
3.41
3.40
2.31
2.30
2.29
1.82
1.81
1.80
1.72
1.71
1.71
1.70
1.70
1.69
1.69
1.69
1.68
1.68
1.68
1.67
1.67
1.61
1.60
1.60
1.36
1.35
1.35
1.34
1.33
1.33
1.32
1.32
1.31
1.31
1.30
1.30
1.13
1.11
0.00
2.00
2.01
2.06
1.00
1.08
2.10
1.13
2.10
6.06
6.00
BPin
CN
F3C
3dd
9.5 9.0 8.5 8.0 7.5 7.0 6.5 6.0 5.5 5.0 4.5 4.0 3.5 3.0 2.5 2.0 1.5 1.0 0.5 0.0
f1 (ppm)

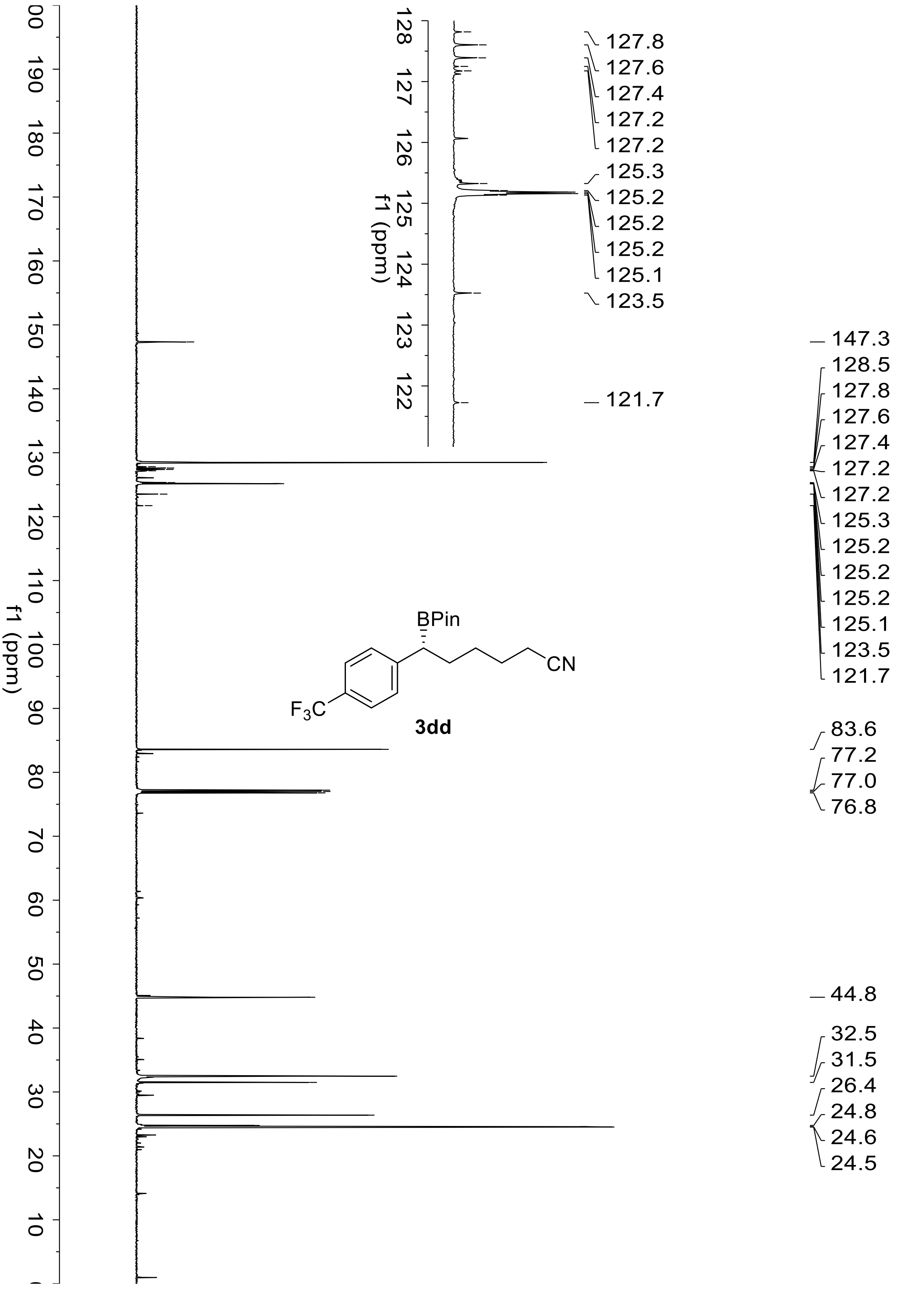
127.8
127.6
127.4
127.2
127.2
125.3
125.2
125.2
125.2
125.1
123.5
121.7
147.3
128.5
127.8
127.6
127.4
127.2
127.2
125.3
125.2
125.2
125.2
125.1
123.5
121.7
83.6
77.2
77.0
76.8
44.8
32.5
31.5
26.4
24.8
24.6
24.5
BPin
CN
F3C
3dd
f1 (ppm)

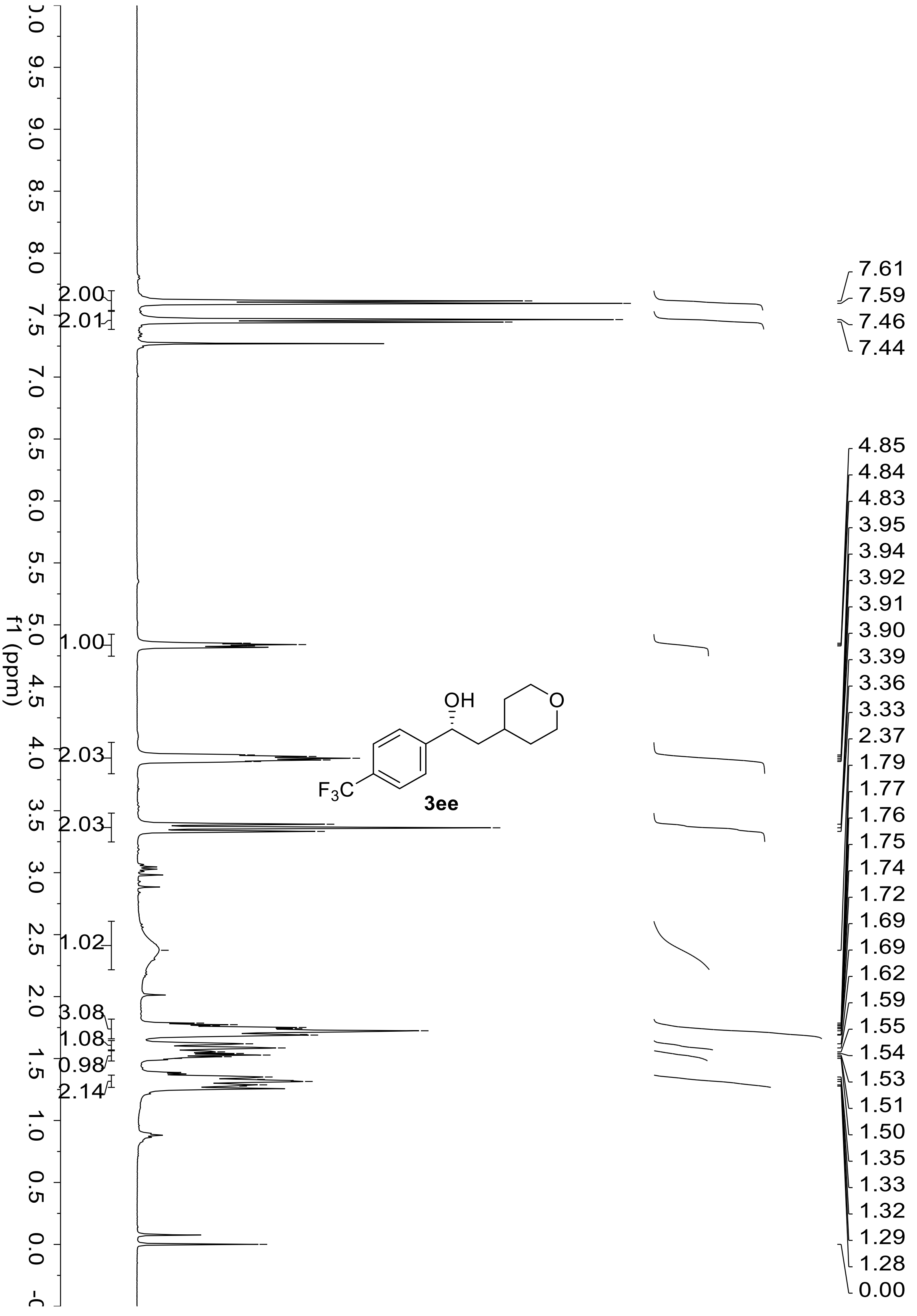

OH
O
F3C
3ee
f1 (ppm)
7.61
7.59
7.46
7.44
4.85
4.84
4.83
3.95
3.94
3.92
3.91
3.90
3.39
3.36
3.33
2.37
1.79
1.77
1.76
1.75
1.74
1.72
1.69
1.69
1.62
1.59
1.55
1.54
1.53
1.51
1.50
1.35
1.33
1.32
1.29
1.28
0.00
2.00
2.01
1.00
2.03
2.03
1.02
3.08
1.08
0.98
2.14

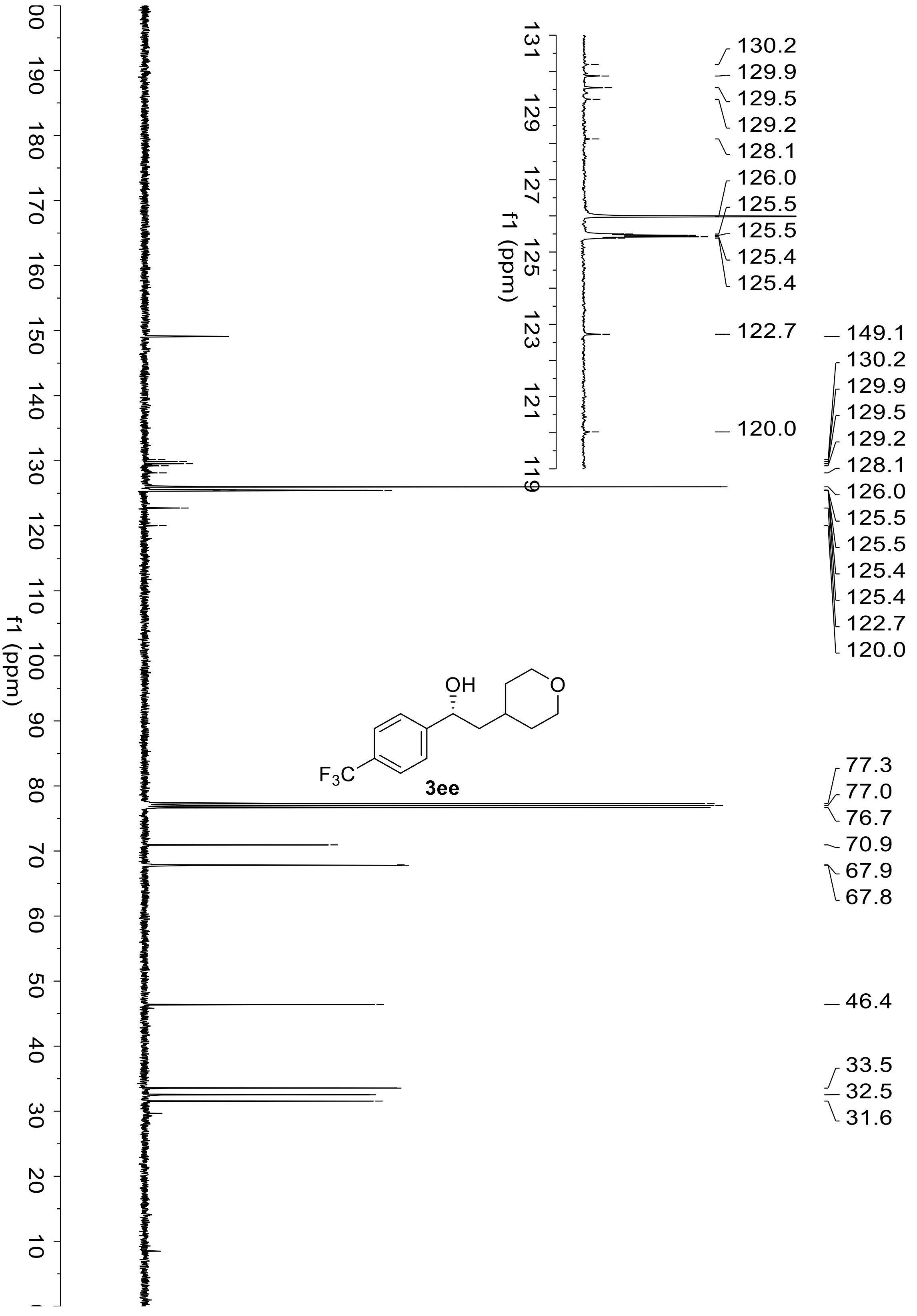

130.2
129.9
129.5
129.2
128.1
126.0
125.5
125.5
125.4
125.4
122.7
120.0
149.1
130.2
129.9
129.5
129.2
128.1
126.0
125.5
125.5
125.4
125.4
122.7
120.0
77.3
77.0
76.7
70.9
67.9
67.8
46.4
33.5
32.5
31.6
OH
O
F_3C
3ee
f1 (ppm)

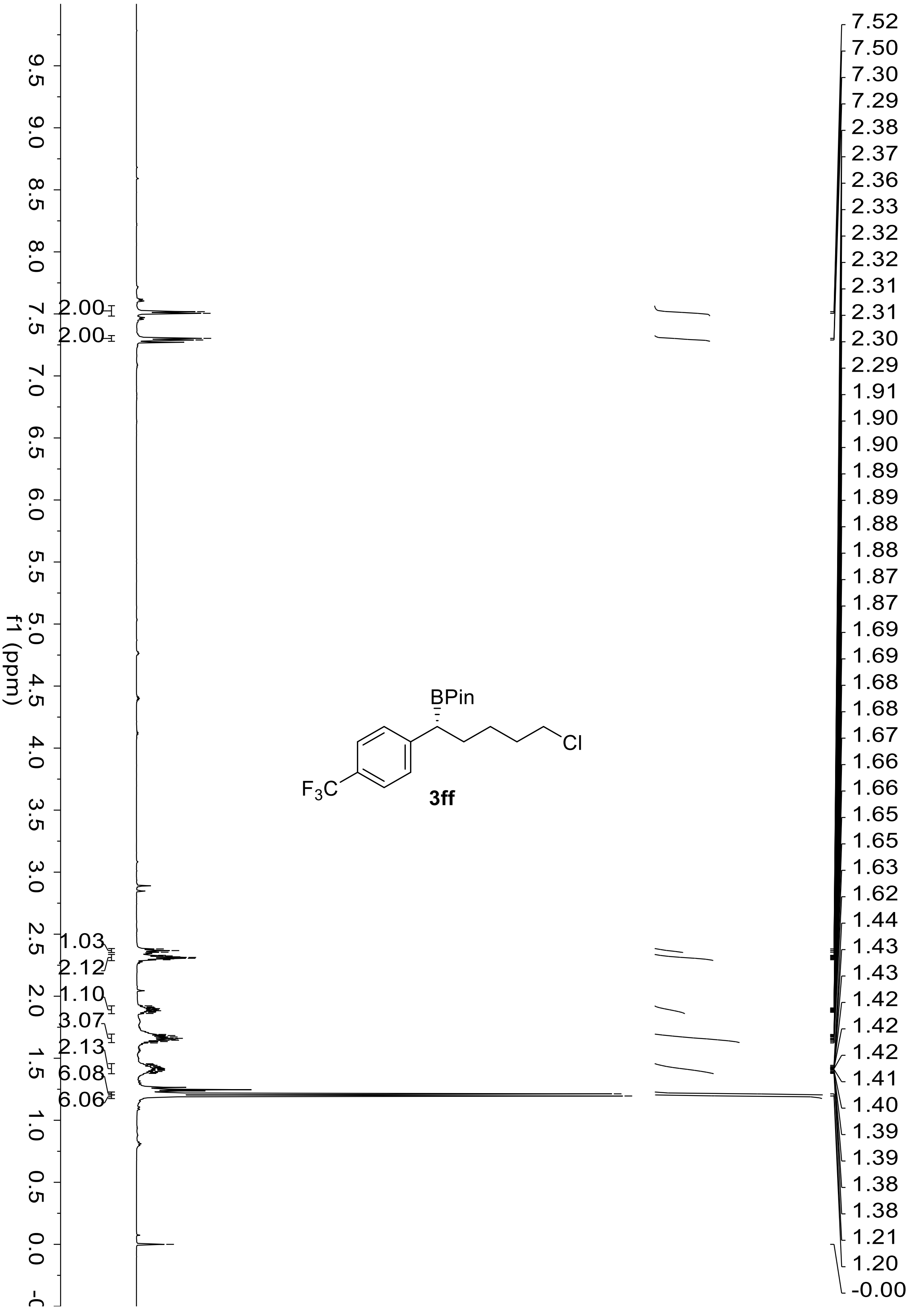

BPin
Cl
F_3C
3ff
f1 (ppm)
9.5
9.0
8.5
8.0
7.5
7.0
6.5
6.0
5.5
5.0
4.5
4.0
3.5
3.0
2.5
2.0
1.5
1.0
0.5
0.0
2.00
2.00
1.03
2.12
1.10
3.07
2.13
6.08
6.06
7.52
7.50
7.30
7.29
2.38
2.37
2.36
2.33
2.32
2.32
2.31
2.31
2.30
2.29
1.91
1.90
1.90
1.89
1.89
1.88
1.88
1.87
1.87
1.87
1.69
1.69
1.68
1.68
1.67
1.66
1.66
1.65
1.65
1.63
1.62
1.44
1.43
1.43
1.42
1.42
1.42
1.41
1.40
1.39
1.39
1.38
1.38
1.21
1.20
-0.00

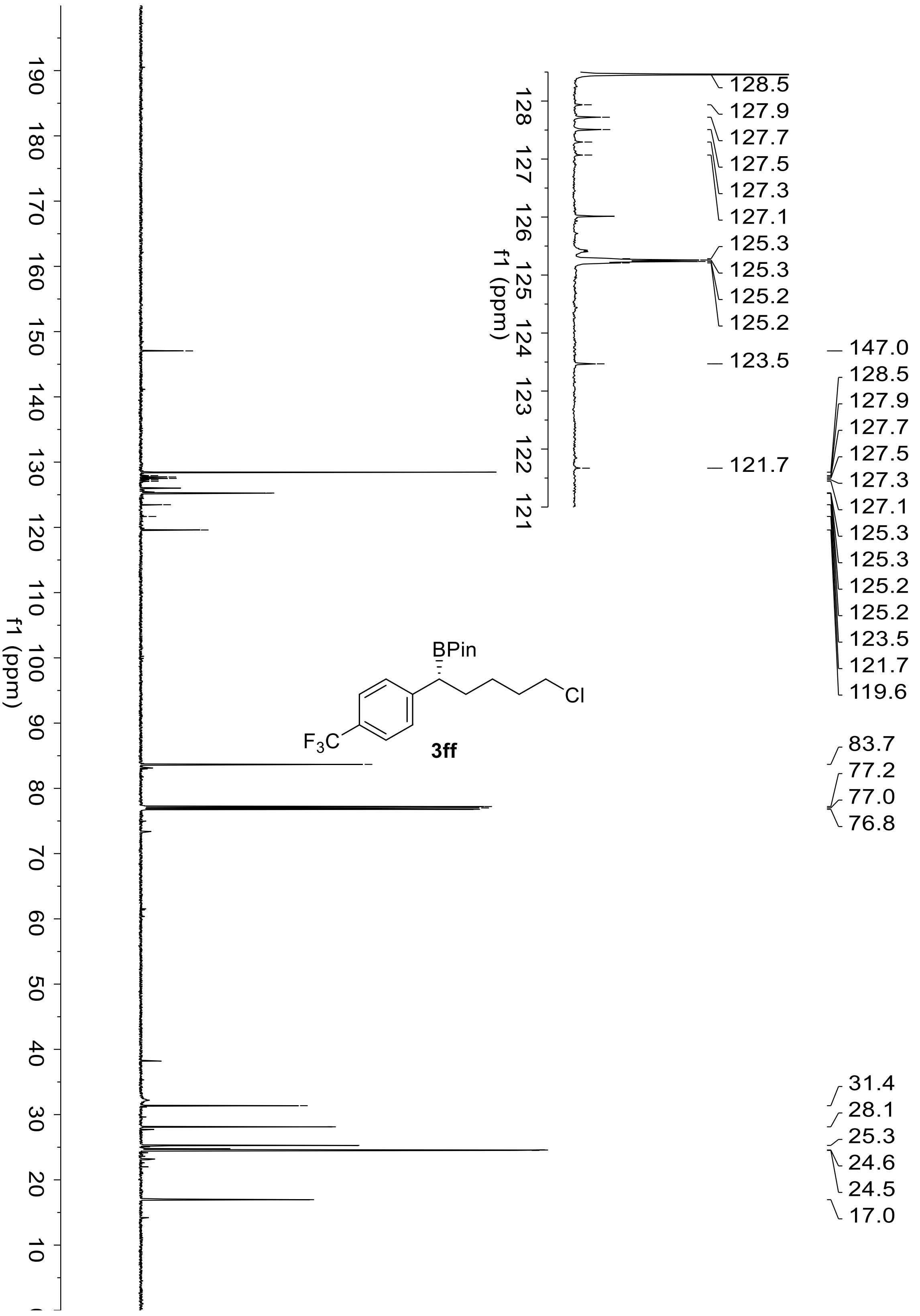
128.5
127.9
127.7
127.5
127.3
127.1
125.3
125.3
125.2
125.2
123.5
121.7
147.0
128.5
127.9
127.7
127.5
127.3
127.1
125.3
125.3
125.2
125.2
123.5
121.7
119.6
83.7
77.2
77.0
76.8
31.4
28.1
25.3
24.6
24.5
17.0
f1 (ppm)
f1 (ppm)
BPin
Cl
F_3C
3ff

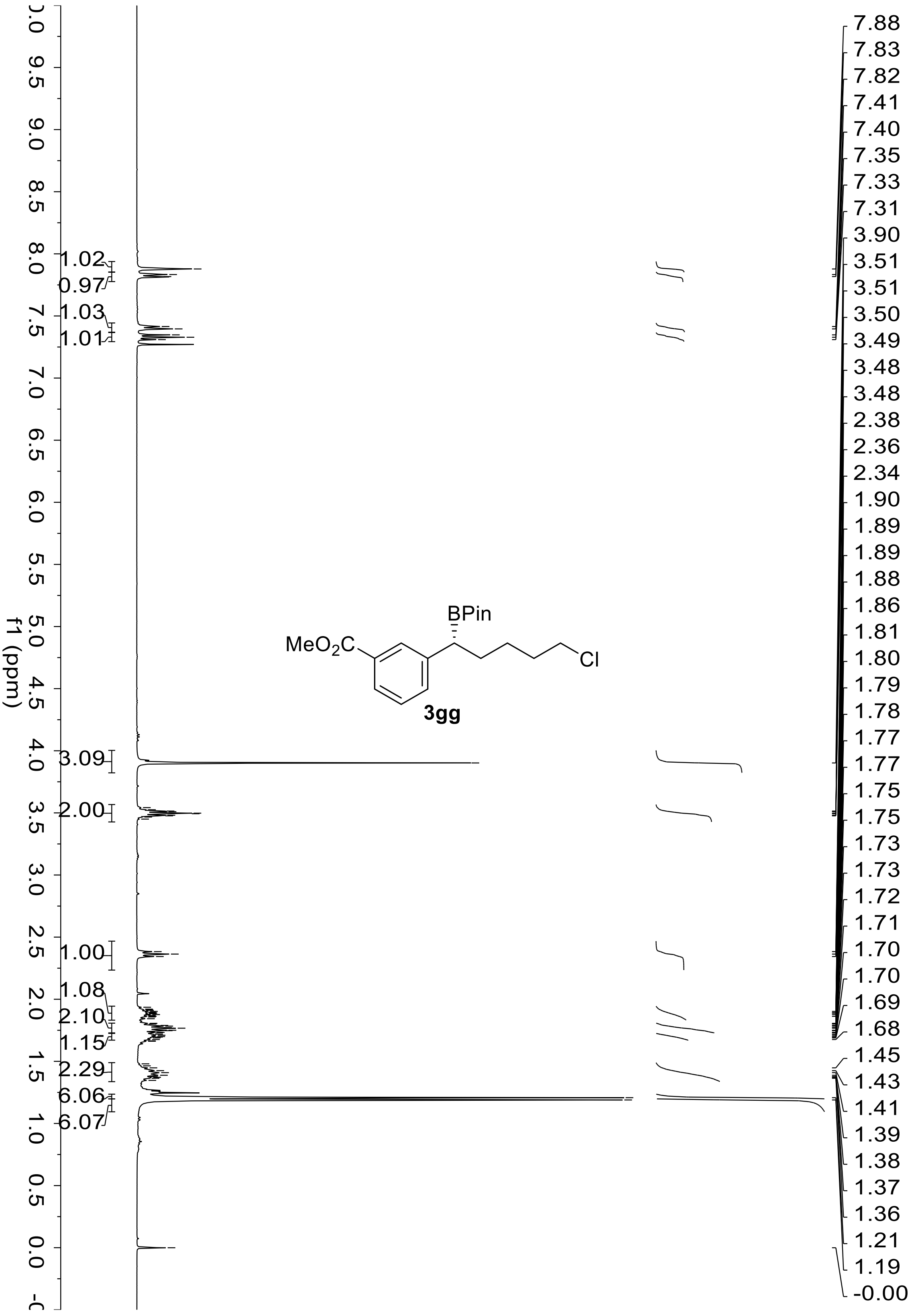
MeO2C
BPin
Cl
3gg
f1 (ppm)
7.88 7.83 7.82 7.41 7.40 7.35 7.33 7.31 3.90 3.51 3.51 3.50 3.49 3.48 3.48 2.38 2.36 2.34 1.90 1.89 1.89 1.88 1.86 1.81 1.80 1.79 1.78 1.77 1.77 1.75 1.75 1.73 1.73 1.72 1.71 1.70 1.70 1.69 1.68 1.45 1.43 1.41 1.39 1.38 1.37 1.36 1.21 1.19 -0.00
1.02 0.97 1.03 1.01 3.09 2.00 1.00 1.08 2.10 1.15 2.29 6.06 6.07

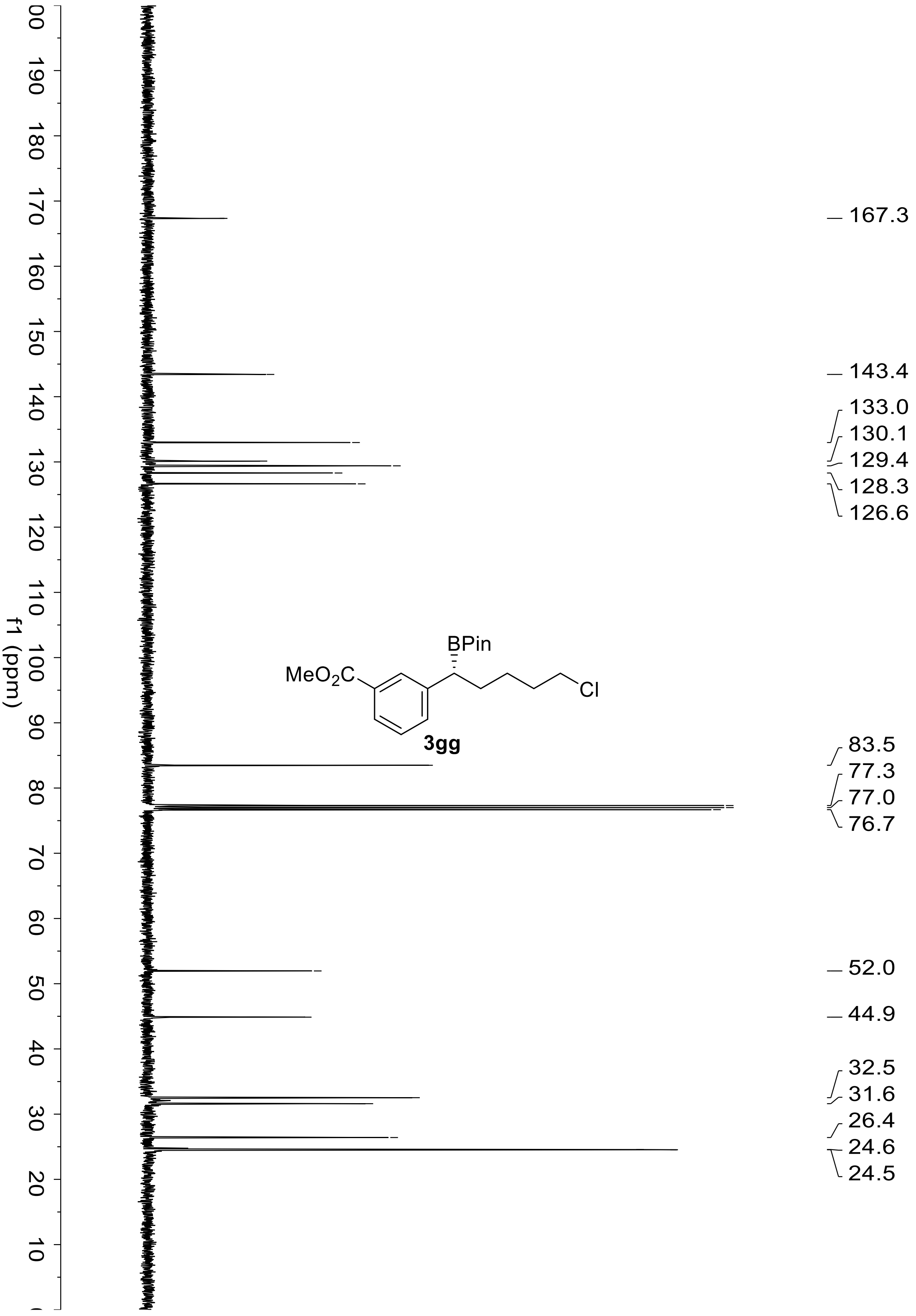
MeO2C
BPin
Cl
3gg
167.3
143.4
133.0
130.1
129.4
128.3
126.6
83.5
77.3
77.0
76.7
52.0
44.9
32.5
31.6
26.4
24.6
24.5
f1 (ppm)

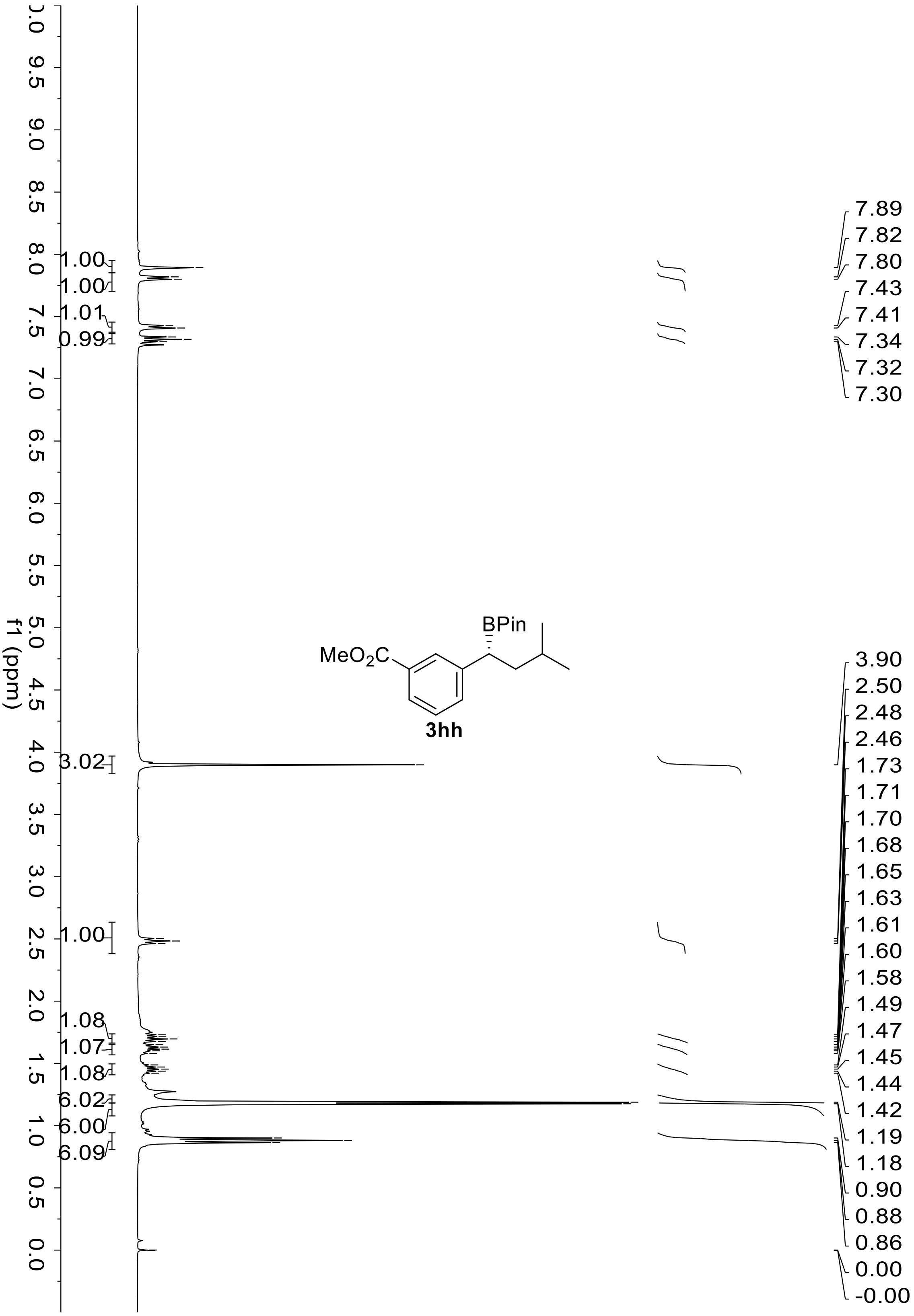
7.89
7.82
7.80
7.43
7.41
7.34
7.32
7.30
3.90
2.50
2.48
2.46
1.73
1.71
1.70
1.68
1.65
1.63
1.61
1.60
1.58
1.49
1.47
1.45
1.44
1.42
1.19
1.18
0.90
0.88
0.86
0.00
-0.00
BPin
MeO_2C
3hh
1.00
1.00
1.01
0.99
3.02
1.00
1.08
1.07
1.08
6.02
6.00
6.09
f1 (ppm)

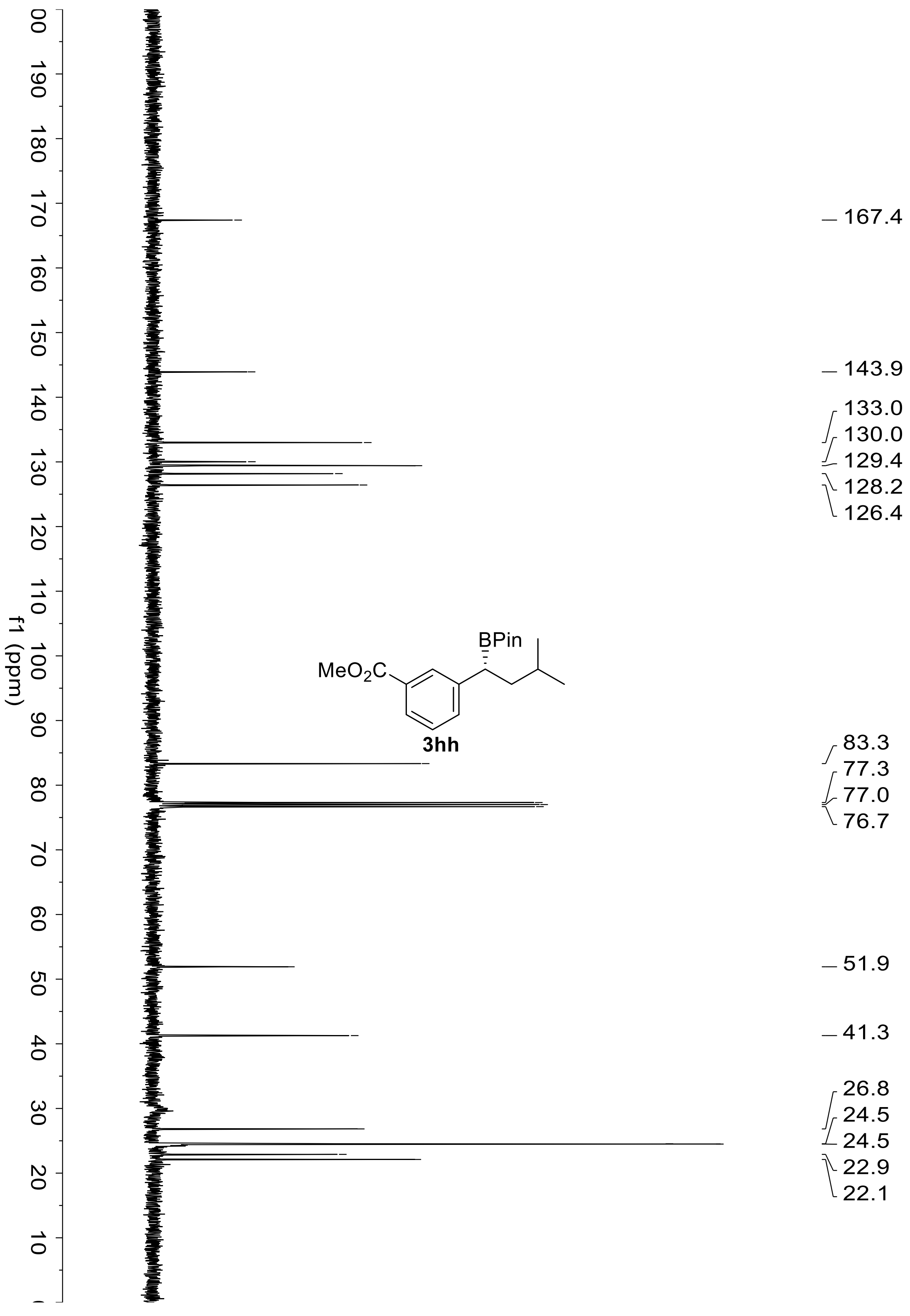
167.4
143.9
133.0
130.0
129.4
128.2
126.4
83.3
77.3
77.0
76.7
51.9
41.3
26.8
24.5
24.5
22.9
22.1
f1 (ppm)
BPin
MeO_2C
3hh

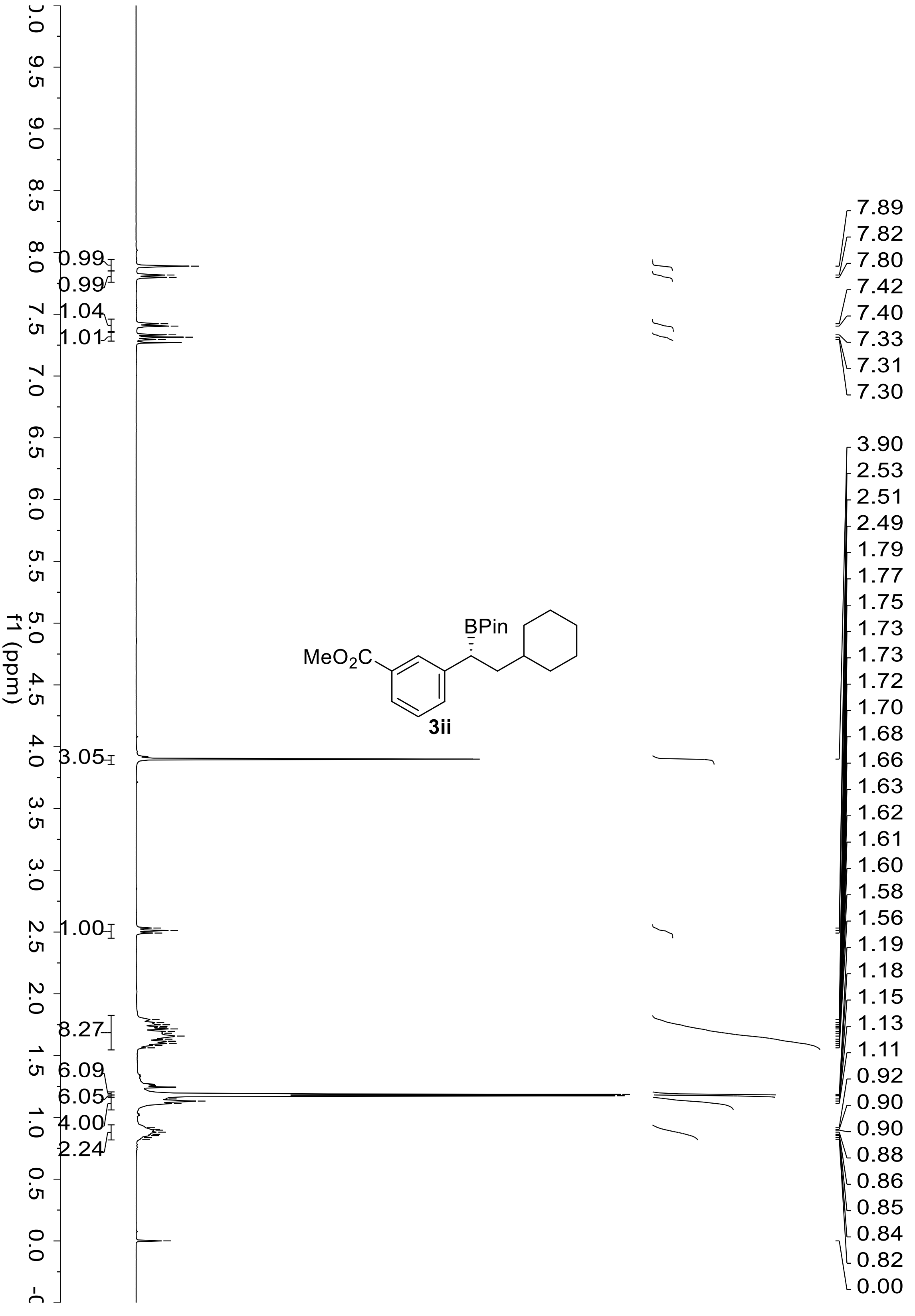

MeO2C
BPin
3ii
f1 (ppm)

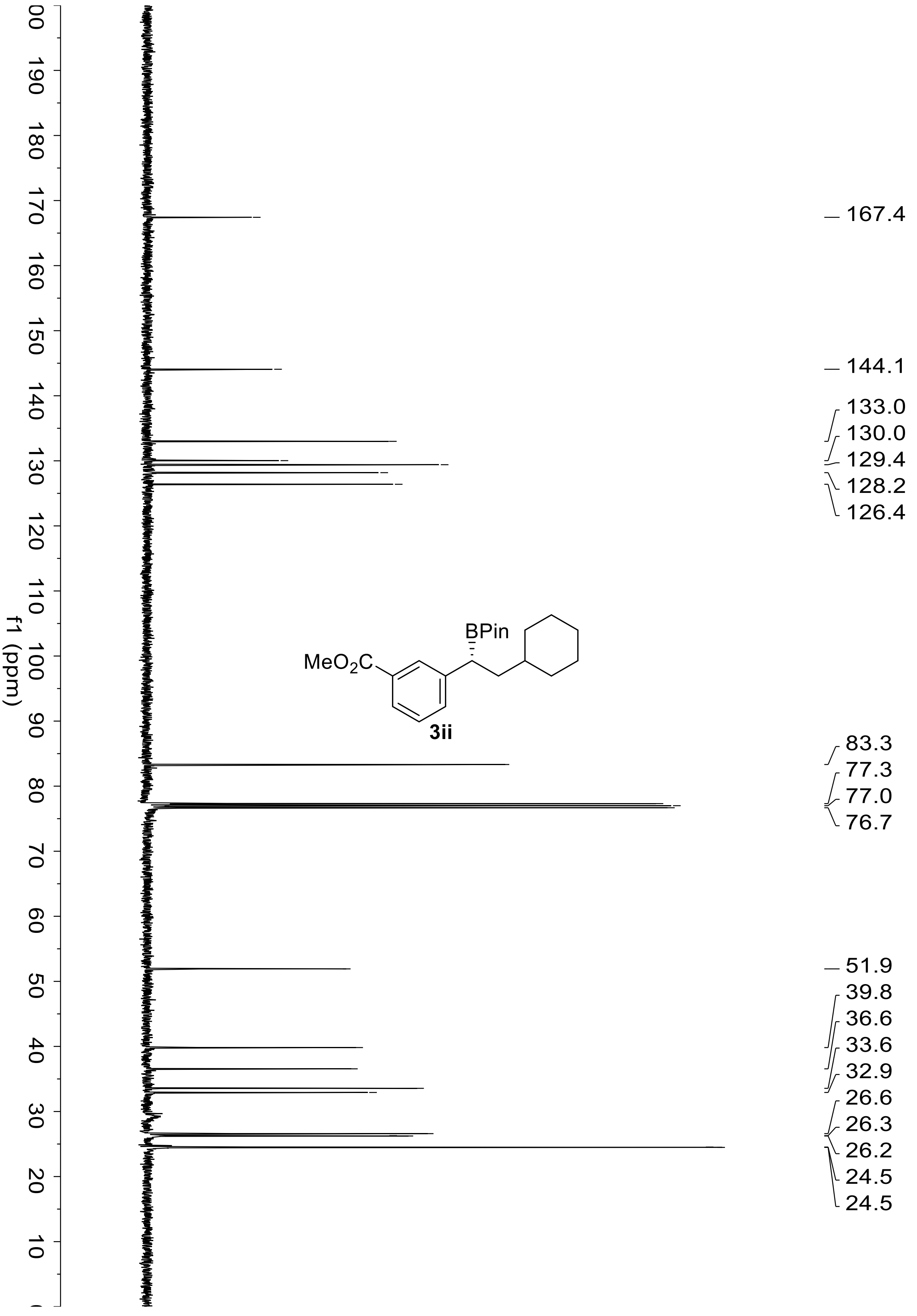
167.4
144.1
133.0
130.0
129.4
128.2
126.4
83.3
77.3
77.0
76.7
51.9
39.8
36.6
33.6
32.9
26.6
26.3
26.2
24.5
24.5
BPin
MeO2C
3ii
f1 (ppm)

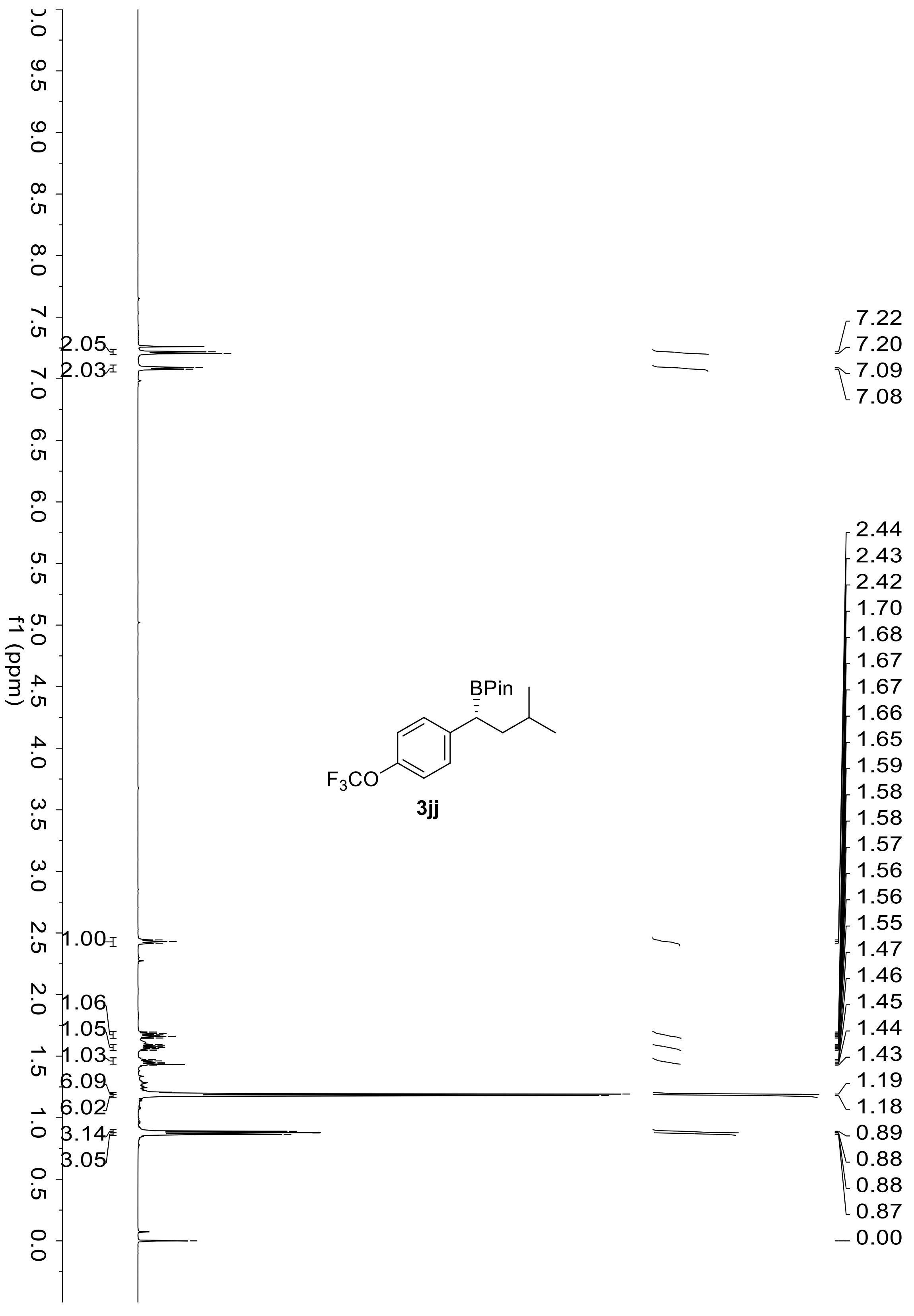
7.22
7.20
7.09
7.08
2.44
2.43
2.42
1.70
1.68
1.67
1.67
1.66
1.65
1.59
1.58
1.58
1.57
1.56
1.56
1.55
1.47
1.46
1.45
1.44
1.43
1.19
1.18
0.89
0.88
0.88
0.87
0.00
2.05
2.03
1.00
1.06
1.05
1.03
6.09
6.02
3.14
3.05
BPin
F3CO
3jj
f1 (ppm)

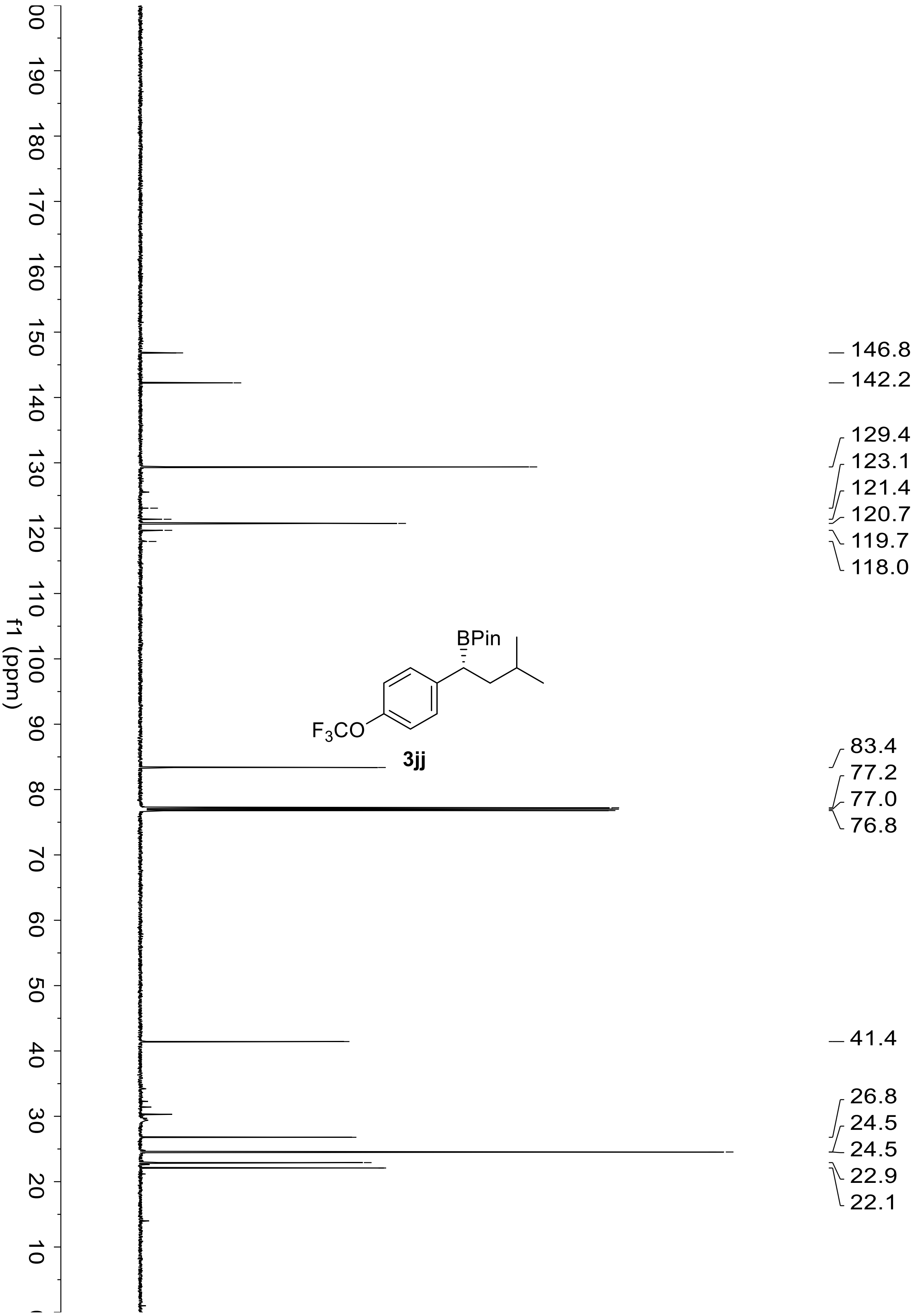

146.8
142.2
129.4
123.1
121.4
120.7
119.7
118.0
BPin
F3CO
3jj
83.4
77.2
77.0
76.8
41.4
26.8
24.5
24.5
22.9
22.1
f1 (ppm)

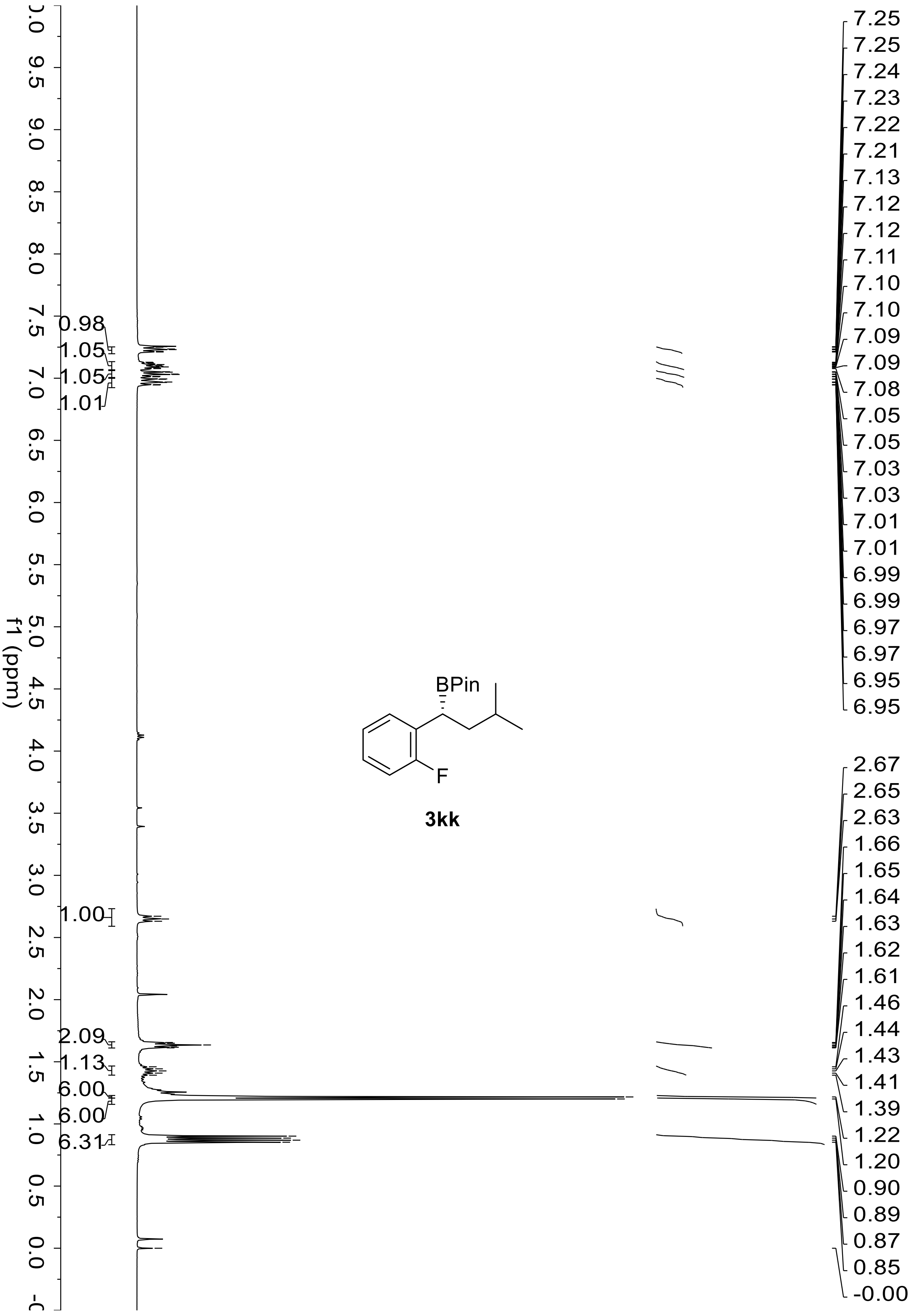
7.25
7.25
7.24
7.23
7.22
7.21
7.13
7.12
7.12
7.11
7.10
7.10
7.09
7.09
7.08
7.05
7.05
7.03
7.03
7.01
7.01
6.99
6.99
6.97
6.97
6.95
6.95
2.67
2.65
2.63
1.66
1.65
1.64
1.63
1.62
1.61
1.46
1.44
1.43
1.41
1.39
1.22
1.20
0.90
0.89
0.87
0.85
-0.00
0.98
1.05
1.05
1.01
1.00
2.09
1.13
6.00
6.00
6.31
BPin
F
3kk
f1 (ppm)

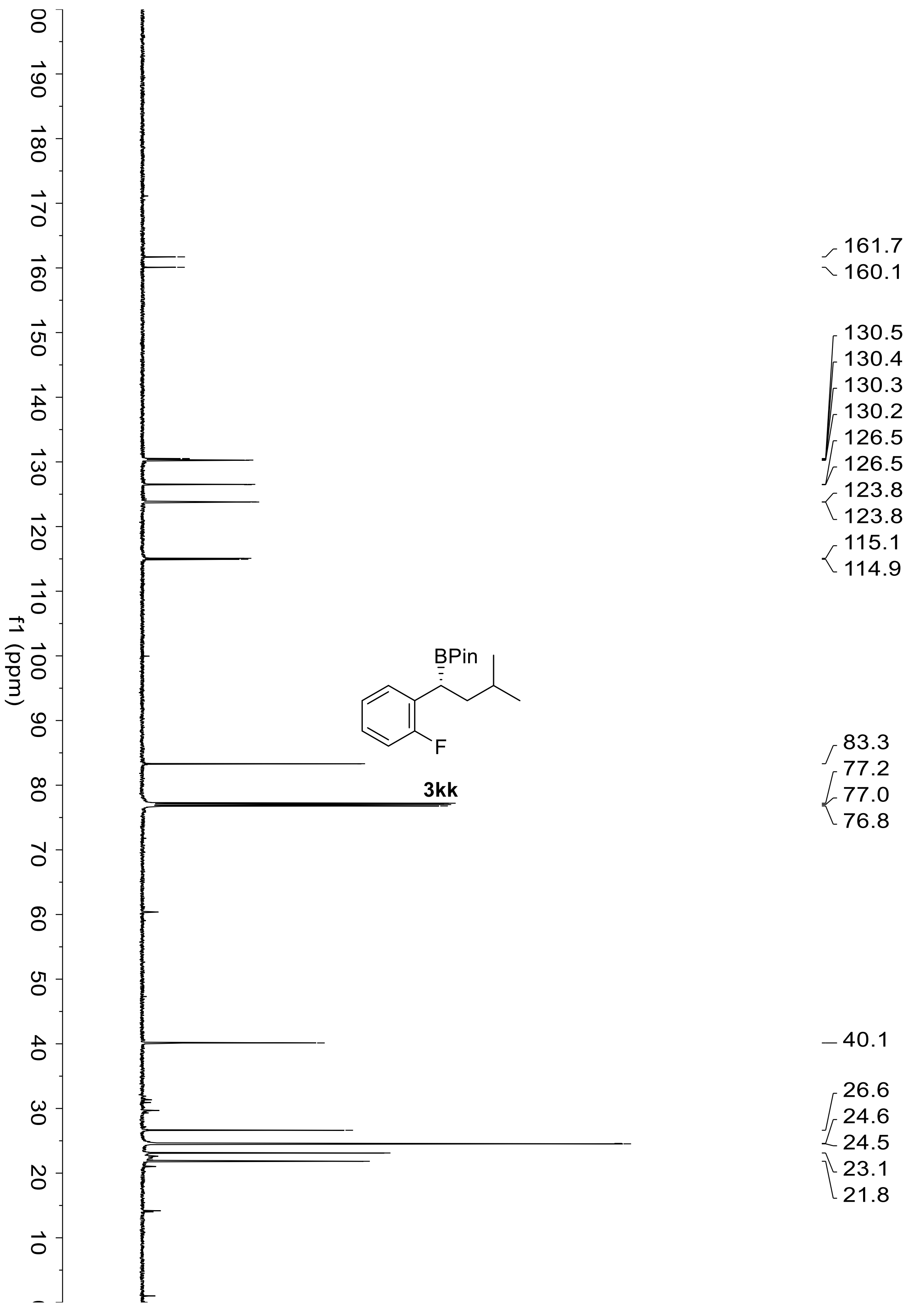

BPin
F
3kk
161.7
160.1
130.5
130.4
130.3
130.2
126.5
126.5
123.8
123.8
115.1
114.9
83.3
77.2
77.0
76.8
40.1
26.6
24.6
24.5
23.1
21.8
f1 (ppm)

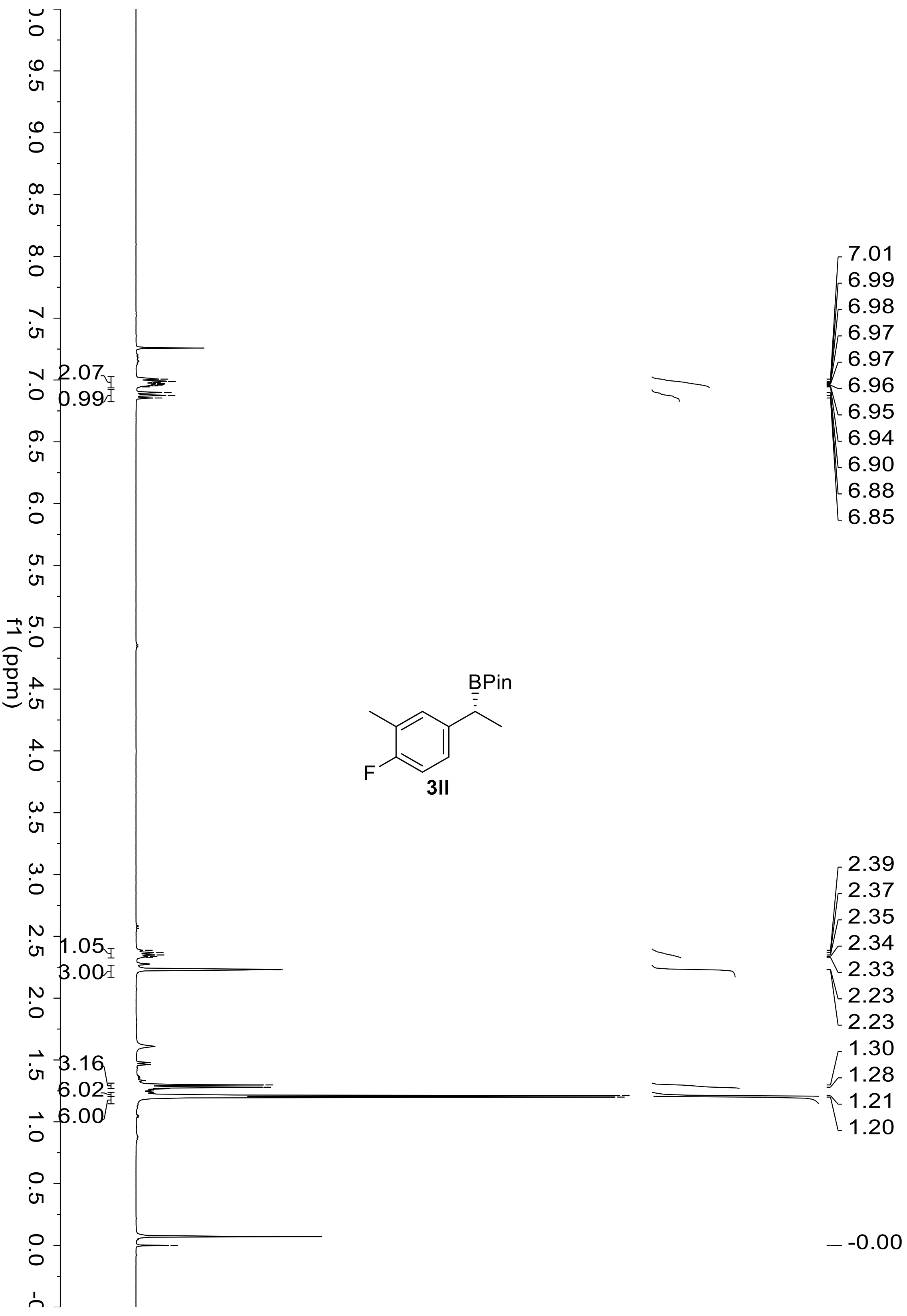

BPin
F
3ll
f1 (ppm)
7.01
6.99
6.98
6.97
6.97
6.96
6.95
6.94
6.90
6.88
6.85
2.39
2.37
2.35
2.34
2.33
2.23
2.23
1.30
1.28
1.21
1.20
-0.00
2.07
0.99
1.05
3.00
3.16
6.02
6.00

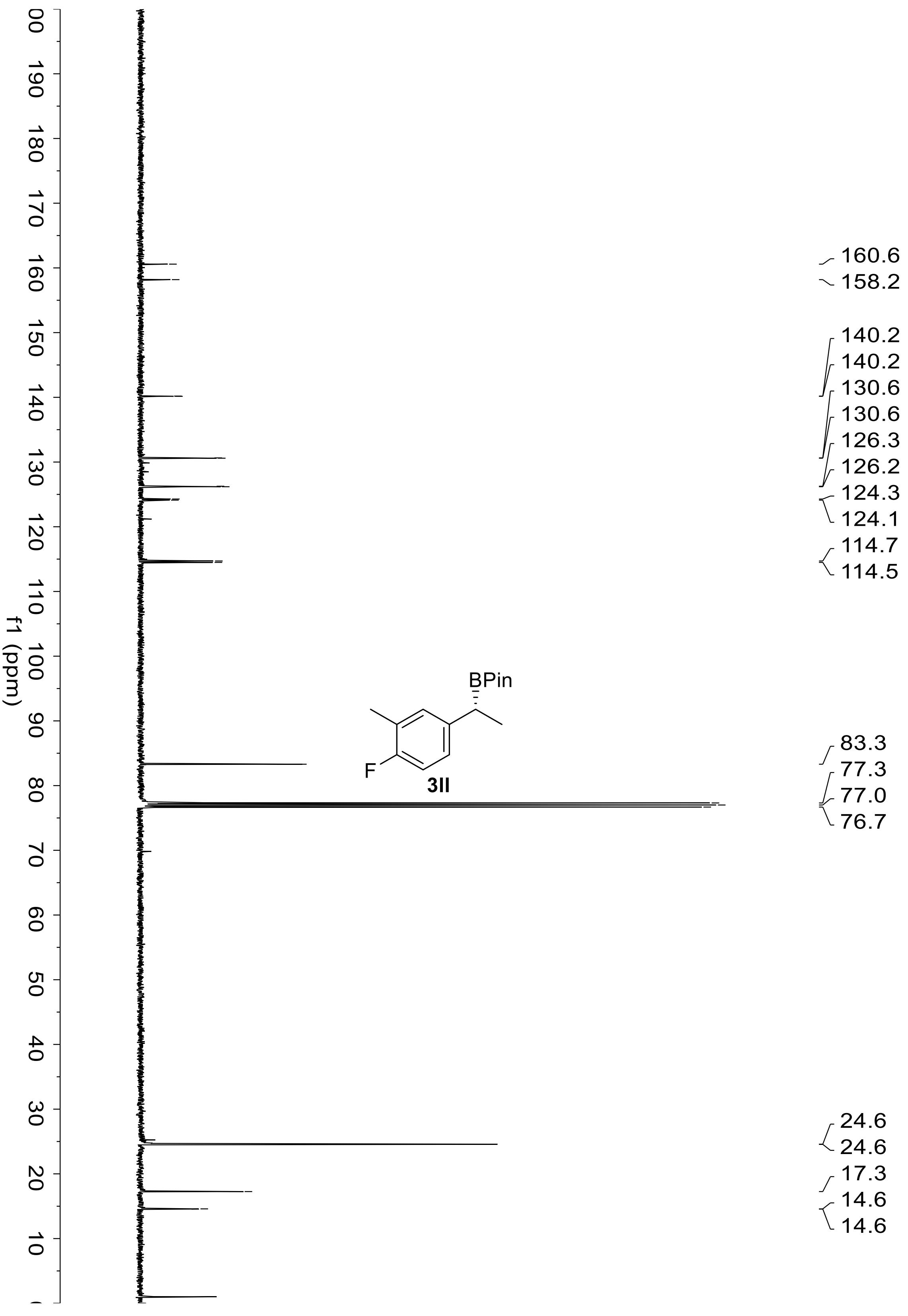
160.6
158.2
140.2
140.2
130.6
130.6
126.3
126.2
124.3
124.1
114.7
114.5
83.3
77.3
77.0
76.7
24.6
24.6
17.3
14.6
14.6
BPin
F
3ll
f1 (ppm)

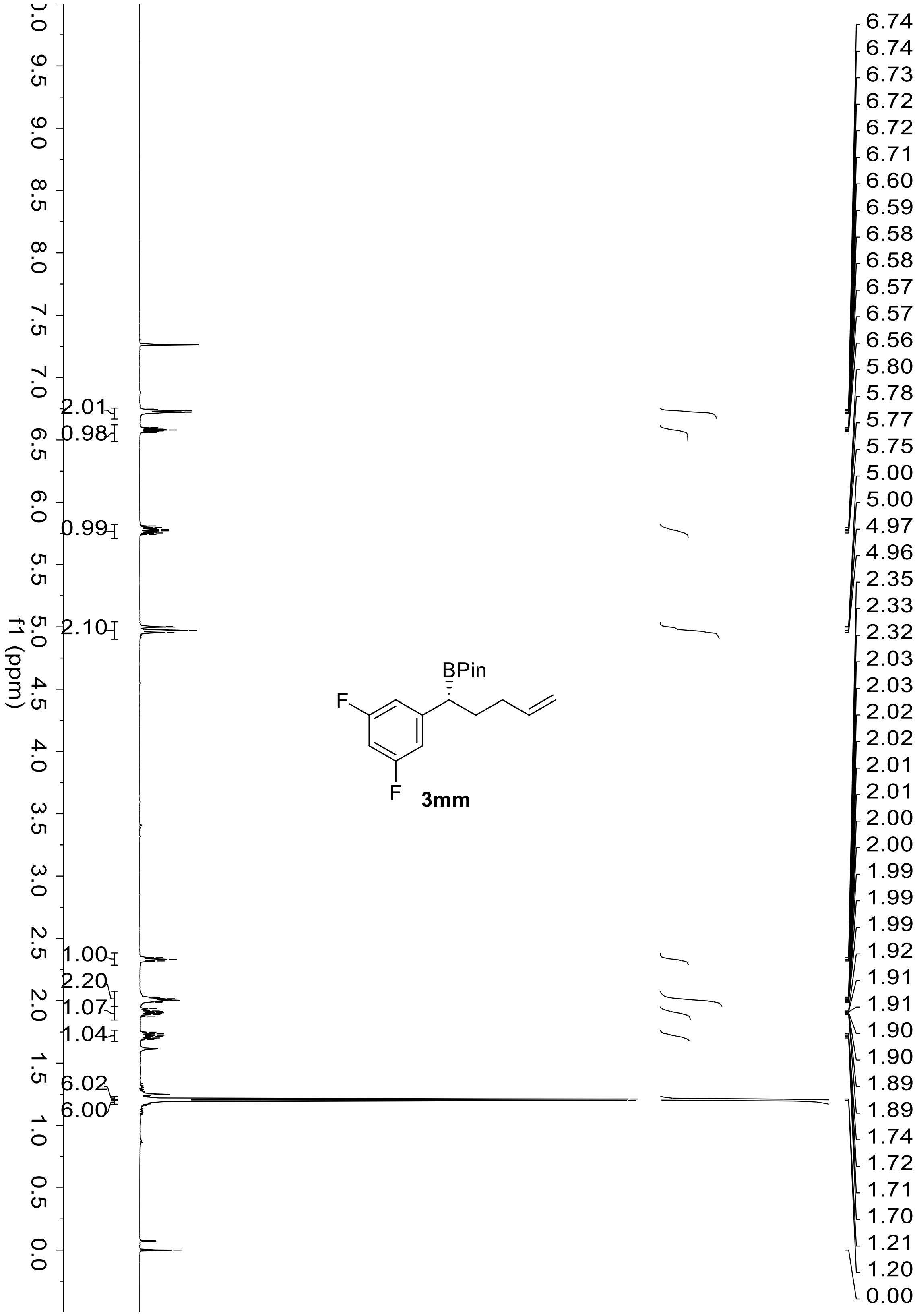
BPin
F
F
3mm
f1 (ppm)

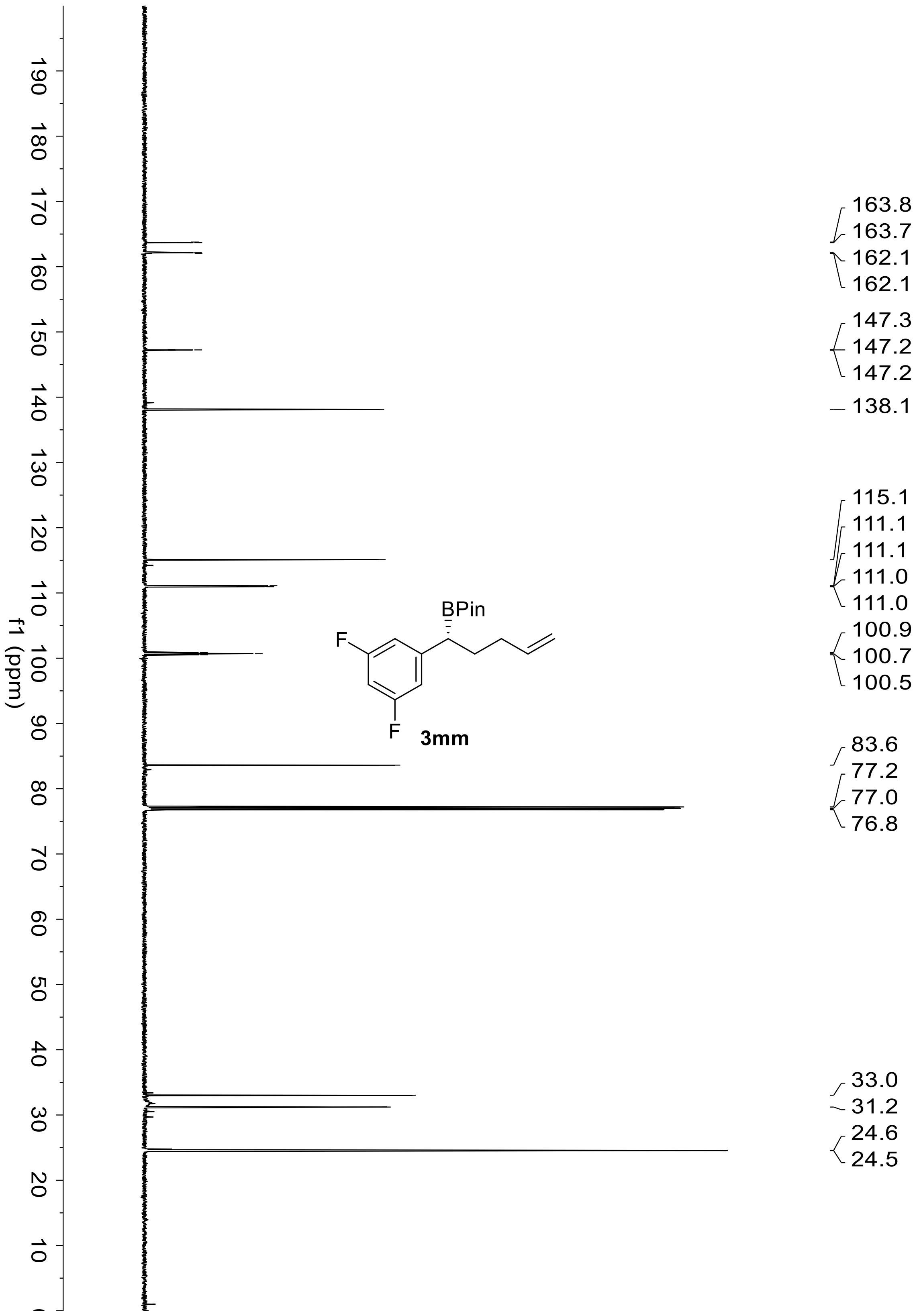
163.8
163.7
162.1
162.1
147.3
147.2
147.2
138.1
115.1
111.1
111.1
111.0
111.0
100.9
100.7
100.5
83.6
77.2
77.0
76.8
33.0
31.2
24.6
24.5
BPin
F
F
3mm
f1 (ppm)
190
180
170
160
150
140
130
120
110
100
90
80
70
60
50
40
30
20
10

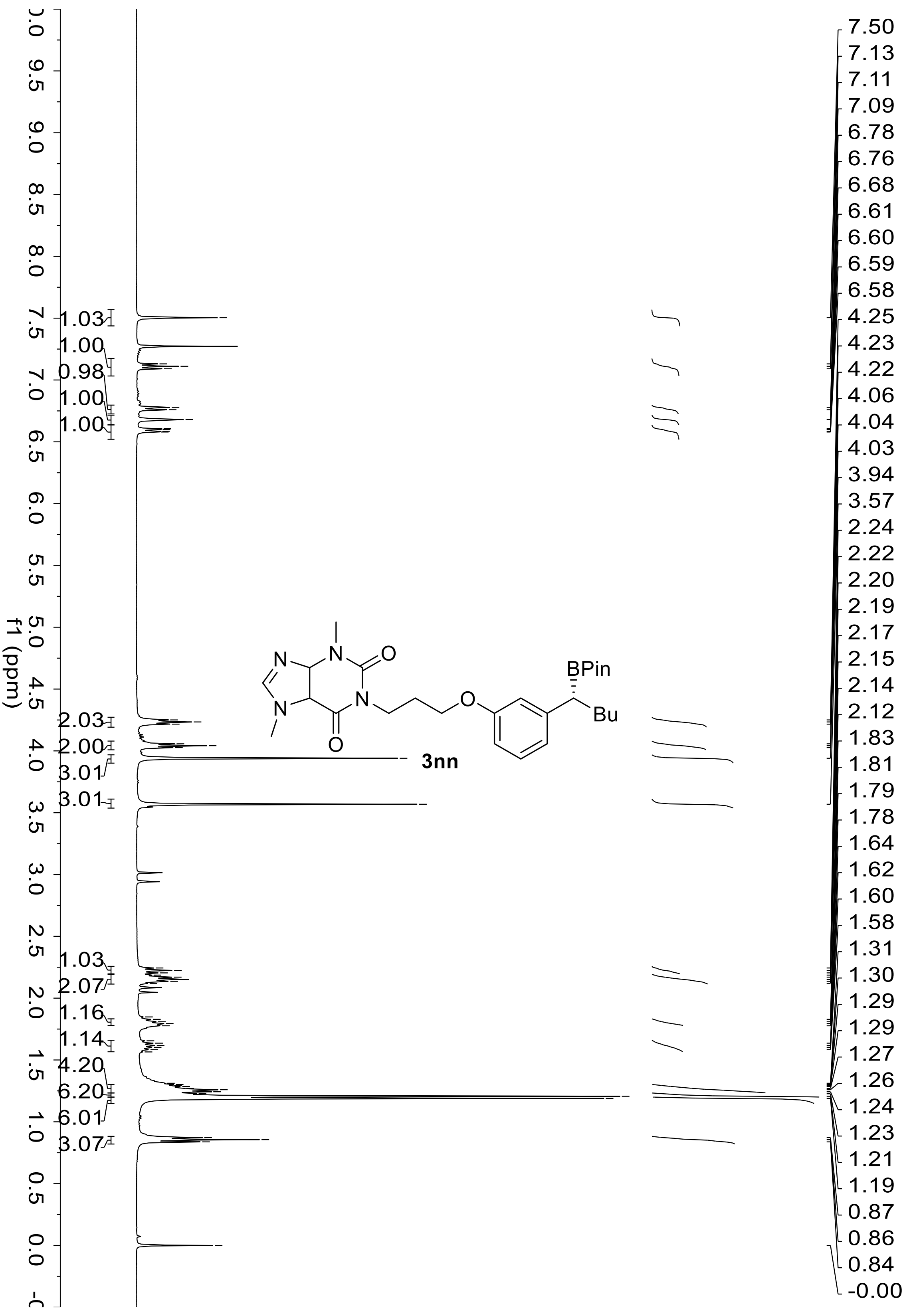
7.50 7.13 7.11 7.09 6.78 6.76 6.68 6.61 6.60 6.59 6.58 4.25 4.23 4.22 4.06 4.04 4.03 3.94 3.57 2.24 2.22 2.20 2.19 2.17 2.15 2.14 2.12 1.83 1.81 1.79 1.78 1.64 1.62 1.60 1.58 1.31 1.30 1.29 1.29 1.27 1.26 1.24 1.23 1.21 1.19 0.87 0.86 0.84 -0.00
BPin
Bu
3nn
1.03 1.00 0.98 1.00 1.00 2.03 2.00 3.01 3.01 1.03 2.07 1.16 1.14 4.20 6.20 6.01 3.07
f1 (ppm)

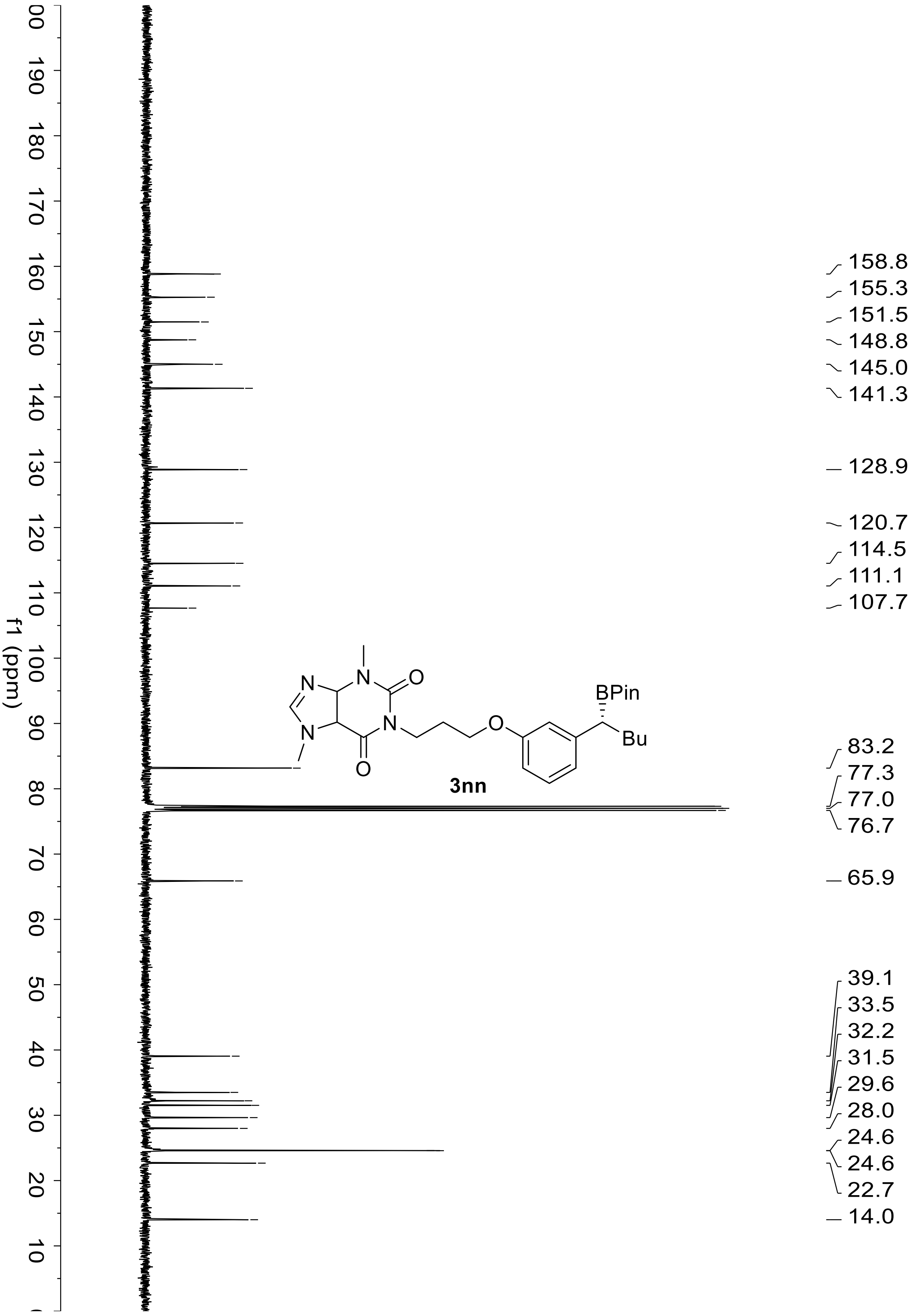
158.8
155.3
151.5
148.8
145.0
141.3
128.9
120.7
114.5
111.1
107.7
83.2
77.3
77.0
76.7
65.9
39.1
33.5
32.2
31.5
29.6
28.0
24.6
24.6
22.7
14.0
BPin
Bu
3nn
f1 (ppm)

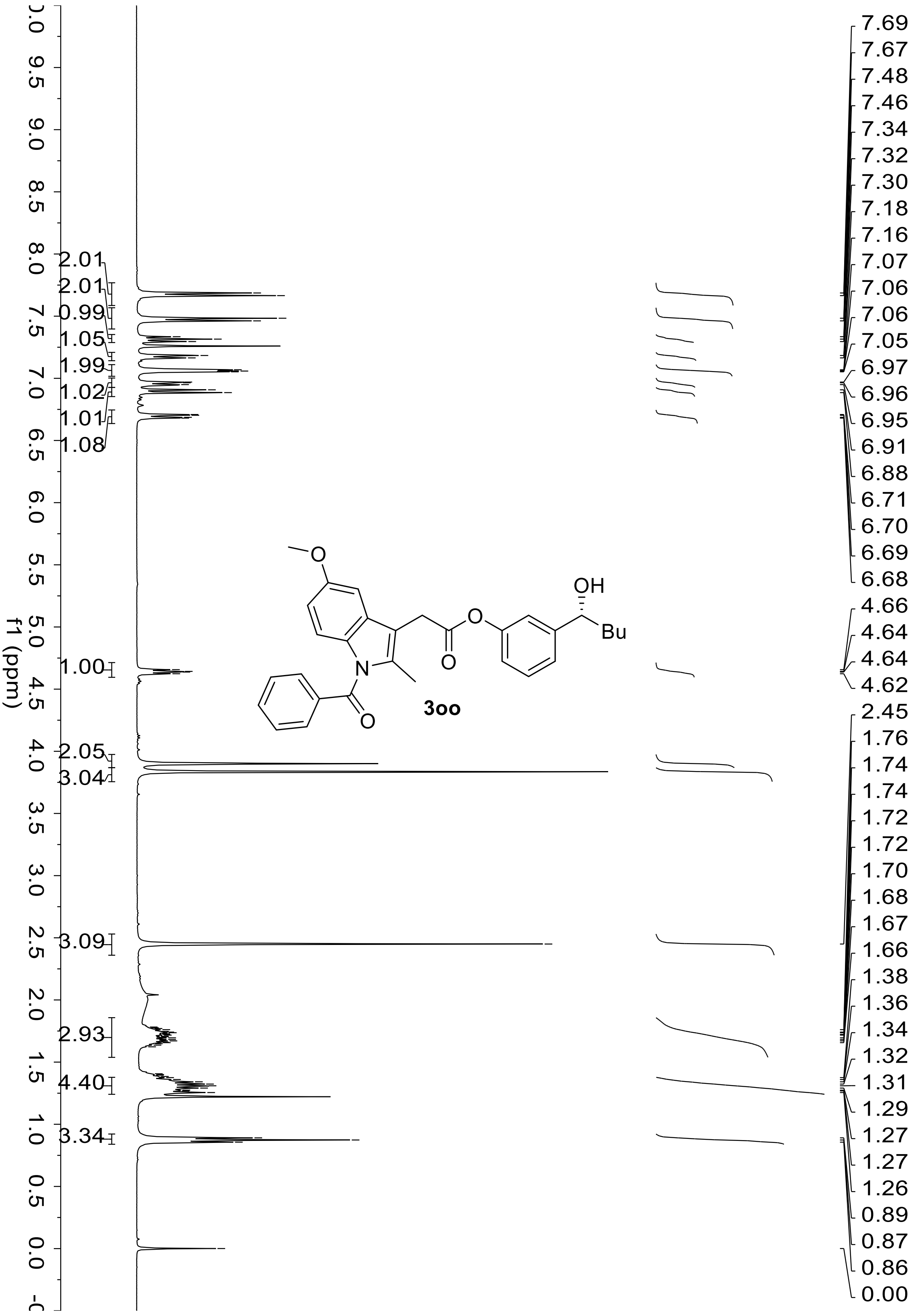
7.69
7.67
7.48
7.46
7.34
7.32
7.30
7.18
7.16
7.07
7.06
7.06
7.05
6.97
6.96
6.95
6.91
6.88
6.71
6.70
6.69
6.68
4.66
4.64
4.64
4.62
2.45
1.76
1.74
1.74
1.72
1.72
1.70
1.68
1.67
1.66
1.38
1.36
1.34
1.32
1.31
1.29
1.27
1.27
1.26
0.89
0.87
0.86
0.00
2.01
2.01
0.99
1.05
1.99
1.02
1.01
1.08
1.00
2.05
3.04
3.09
2.93
4.40
3.34
f1 (ppm)
OH
Bu
O
N
3oo

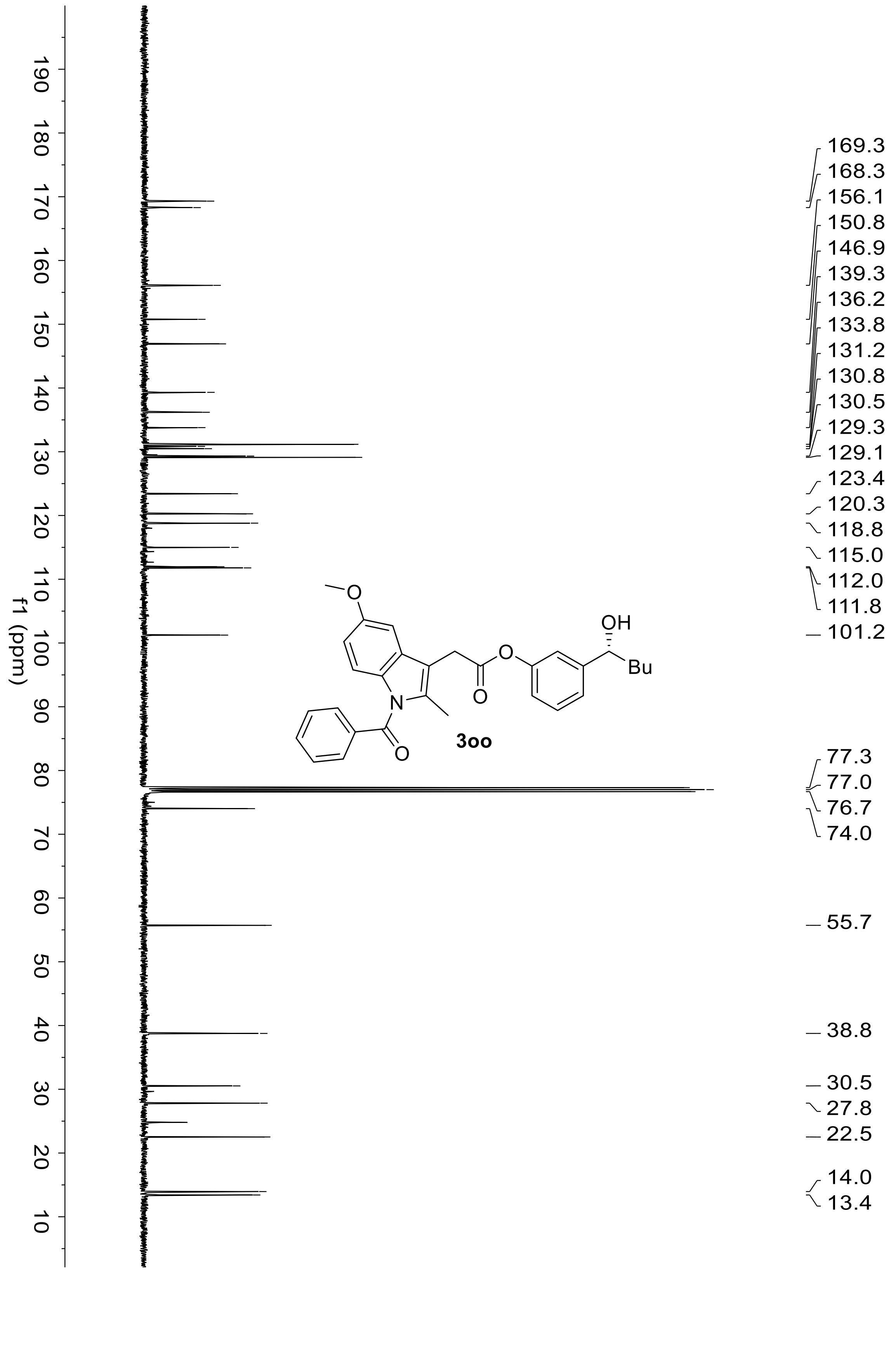
169.3
168.3
156.1
150.8
146.9
139.3
136.2
133.8
131.2
130.8
130.5
129.3
129.1
123.4
120.3
118.8
115.0
112.0
111.8
101.2
77.3
77.0
76.7
74.0
55.7
38.8
30.5
27.8
22.5
14.0
13.4
OH
Bu
O
N
3oo
f1 (ppm)
190
180
170
160
150
140
130
120
110
100
90
80
70
60
50
40
30
20
10

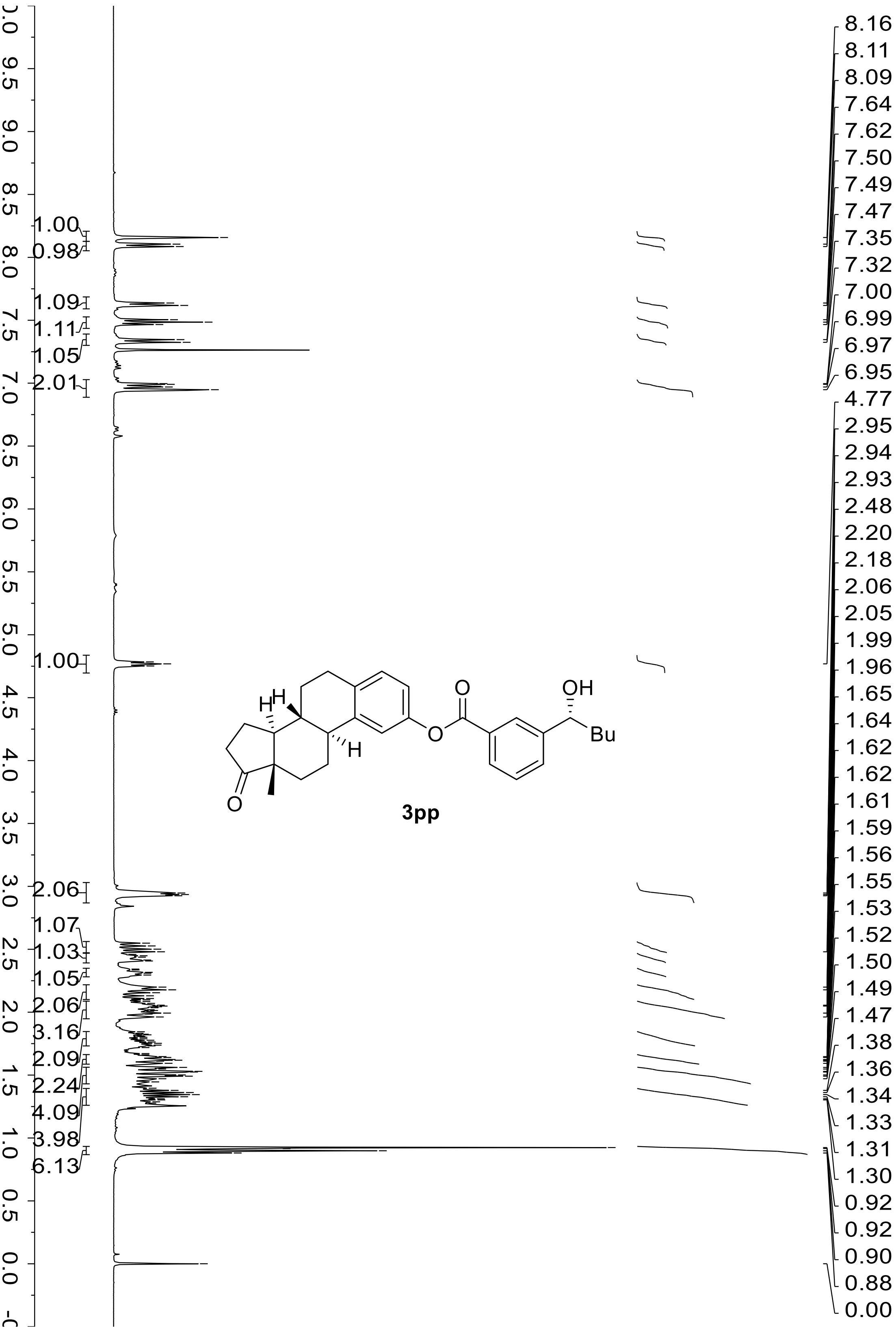
8.16
8.11
8.09
7.64
7.62
7.50
7.49
7.47
7.35
7.32
7.00
6.99
6.97
6.95
4.77
2.95
2.94
2.93
2.48
2.20
2.18
2.06
2.05
1.99
1.96
1.65
1.64
1.62
1.62
1.61
1.59
1.56
1.55
1.53
1.52
1.50
1.49
1.47
1.38
1.36
1.34
1.33
1.31
1.30
0.92
0.92
0.90
0.88
0.00
1.00
0.98
1.09
1.11
1.05
2.01
1.00
2.06
1.07
1.03
1.05
2.06
3.16
2.09
2.24
4.09
3.98
6.13
f1 (ppm)
O
OH
H
H
H
O
Bu
O
3pp

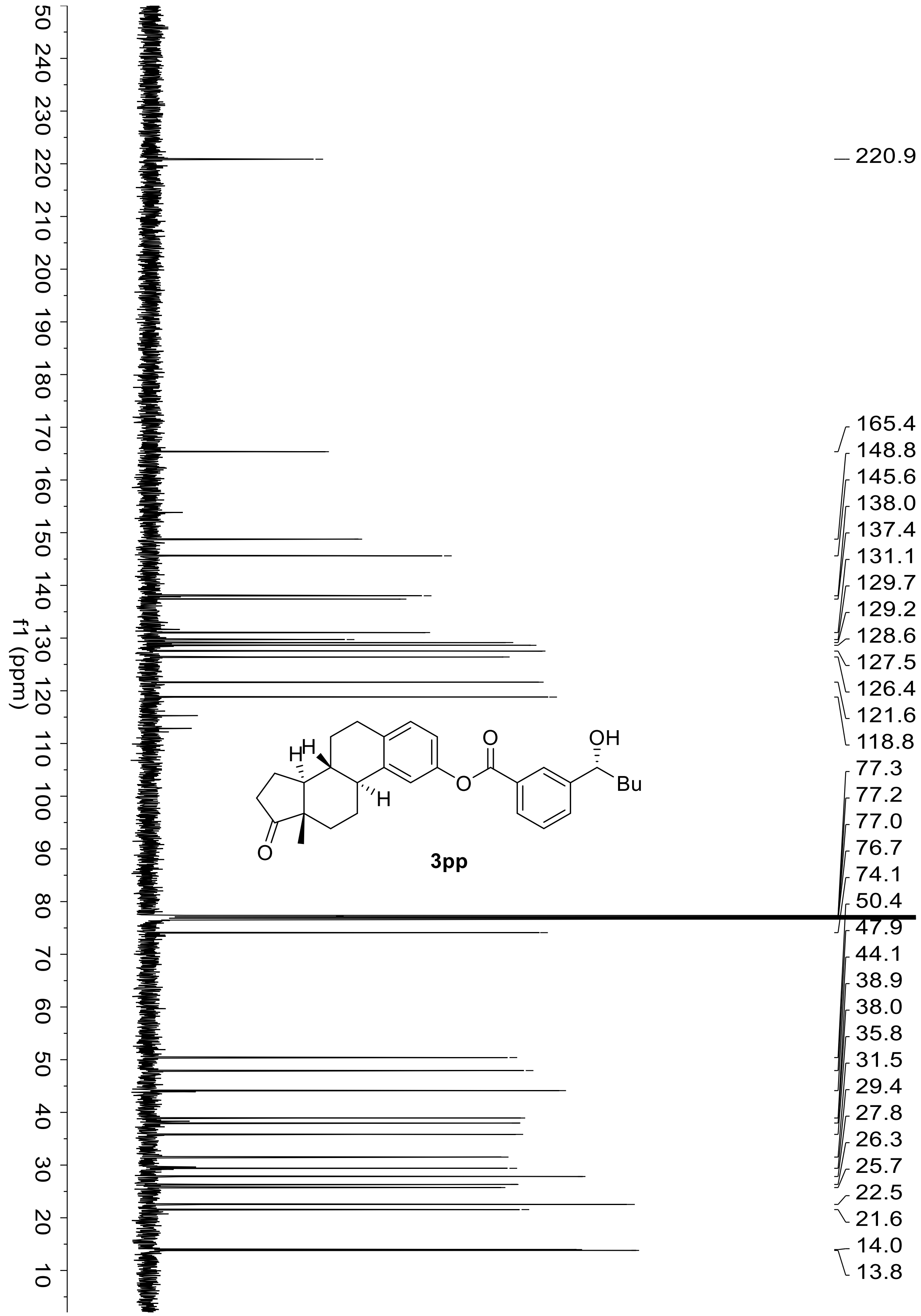
220.9
165.4
148.8
145.6
138.0
137.4
131.1
129.7
129.2
128.6
127.5
126.4
121.6
118.8
77.3
77.2
77.0
76.7
74.1
50.4
47.9
44.1
38.9
38.0
35.8
31.5
29.4
27.8
26.3
25.7
22.5
21.6
14.0
13.8
OH
Bu
O
H
3pp
f1 (ppm)
250 240 230 220 210 200 190 180 170 160 150 140 130 120 110 100 90 80 70 60 50 40 30 20 10

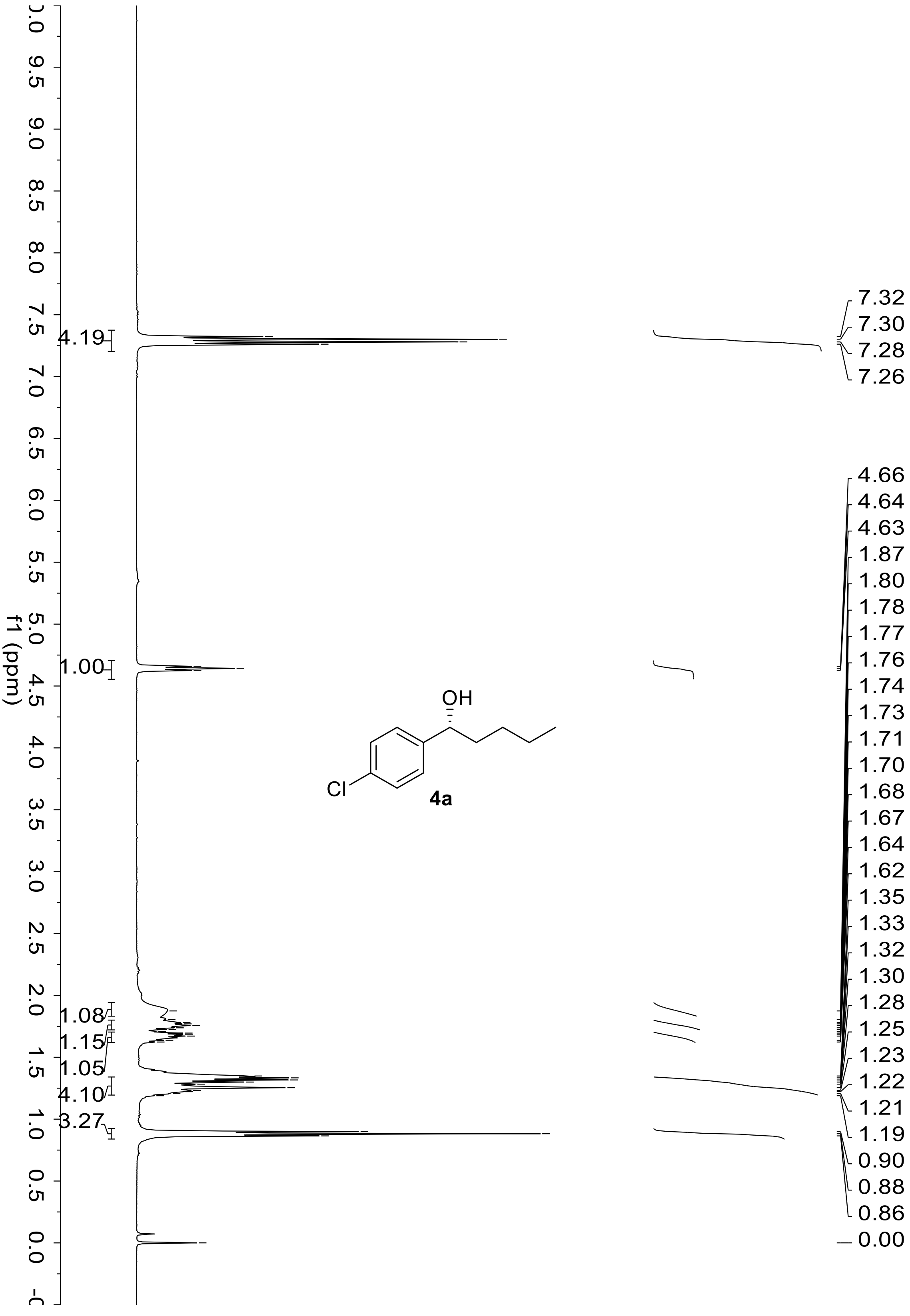
7.32
7.30
7.28
7.26
4.66
4.64
4.63
1.87
1.80
1.78
1.77
1.76
1.74
1.73
1.71
1.70
1.68
1.67
1.64
1.62
1.35
1.33
1.32
1.30
1.28
1.25
1.23
1.22
1.21
1.19
0.90
0.88
0.86
0.00
4.19
1.00
1.08
1.15
1.05
4.10
3.27
OH
Cl
4a
f1 (ppm)

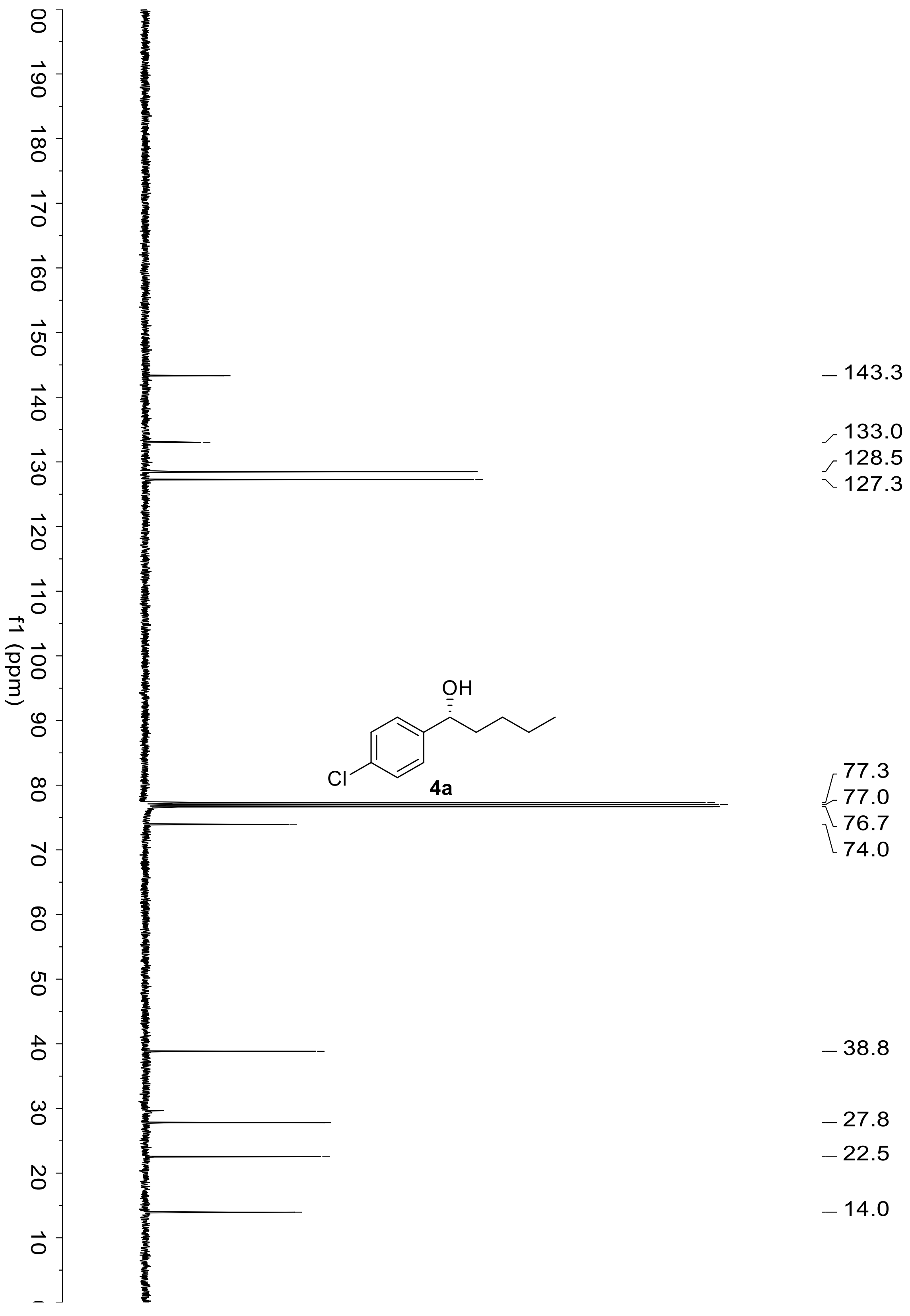

143.3
133.0
128.5
127.3
77.3
77.0
76.7
74.0
38.8
27.8
22.5
14.0
OH
Cl
4a
f1 (ppm)

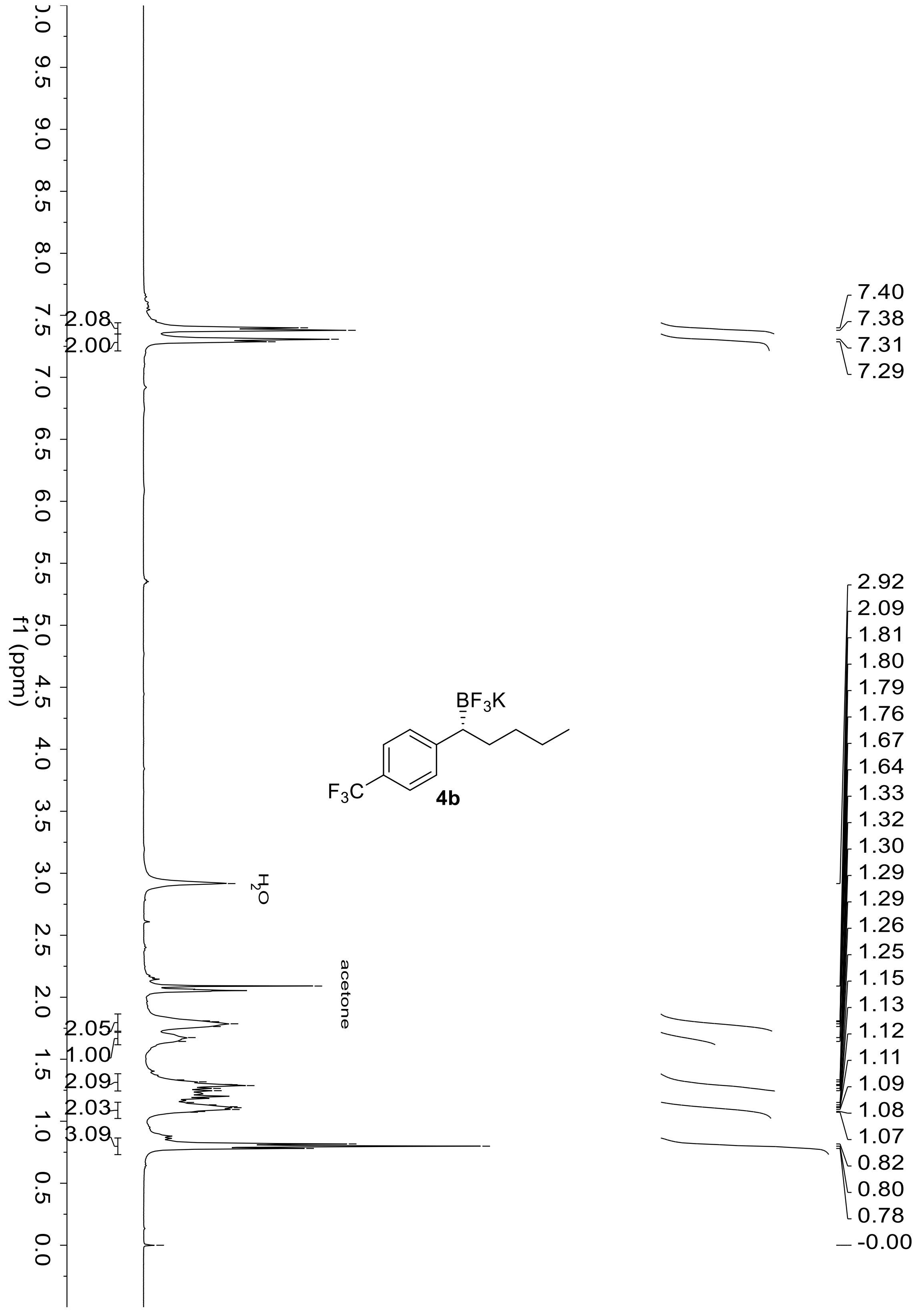

7.40
7.38
7.31
7.29
2.92
2.09
1.81
1.80
1.79
1.76
1.67
1.64
1.33
1.32
1.30
1.29
1.29
1.26
1.25
1.15
1.13
1.12
1.11
1.09
1.08
1.07
0.82
0.80
0.78
-0.00
BF3K
F3C
4b
H2O
acetone
2.08
2.00
2.05
1.00
2.09
2.03
3.09
f1 (ppm)

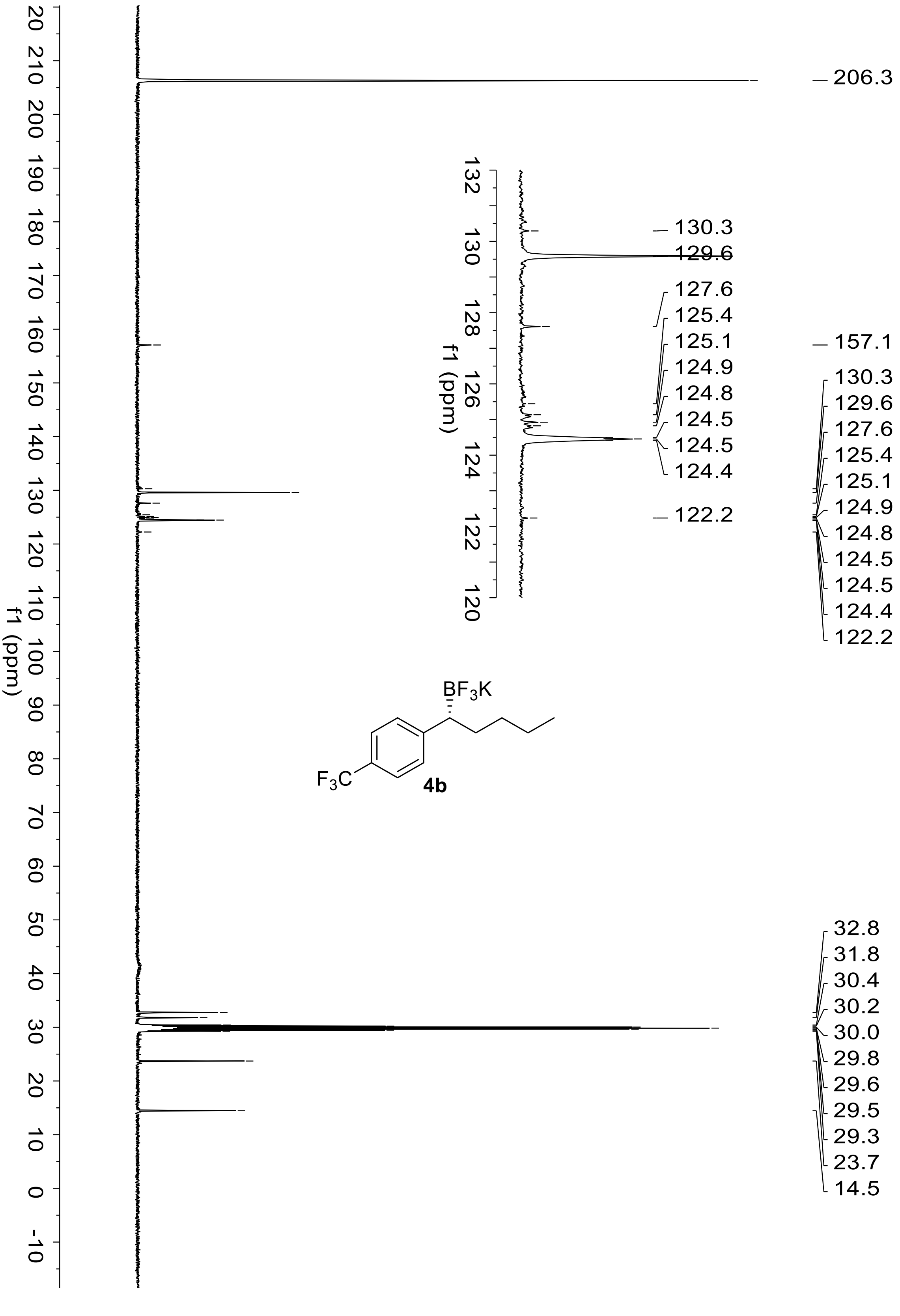
206.3
130.3
129.6
127.6
125.4
125.1
124.9
124.8
124.5
124.5
124.4
122.2
157.1
32.8
31.8
30.4
30.2
30.0
29.8
29.6
29.5
29.3
23.7
14.5
f1 (ppm)
BF3K
F3C
4b

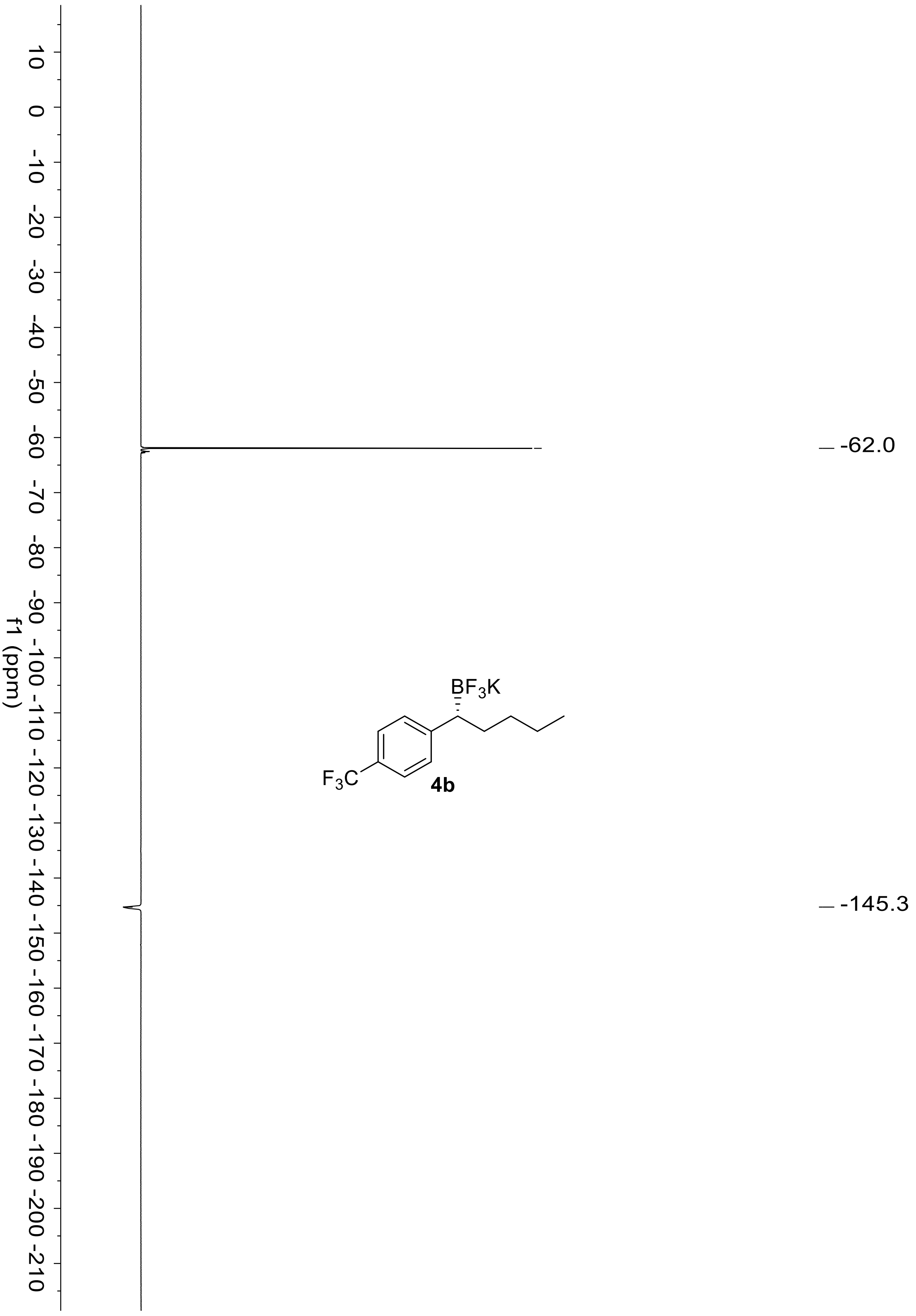
-62.0
-145.3
BF3K
F3C
4b
f1 (ppm)
10 0 -10 -20 -30 -40 -50 -60 -70 -80 -90 -100 -110 -120 -130 -140 -150 -160 -170 -180 -190 -200 -210

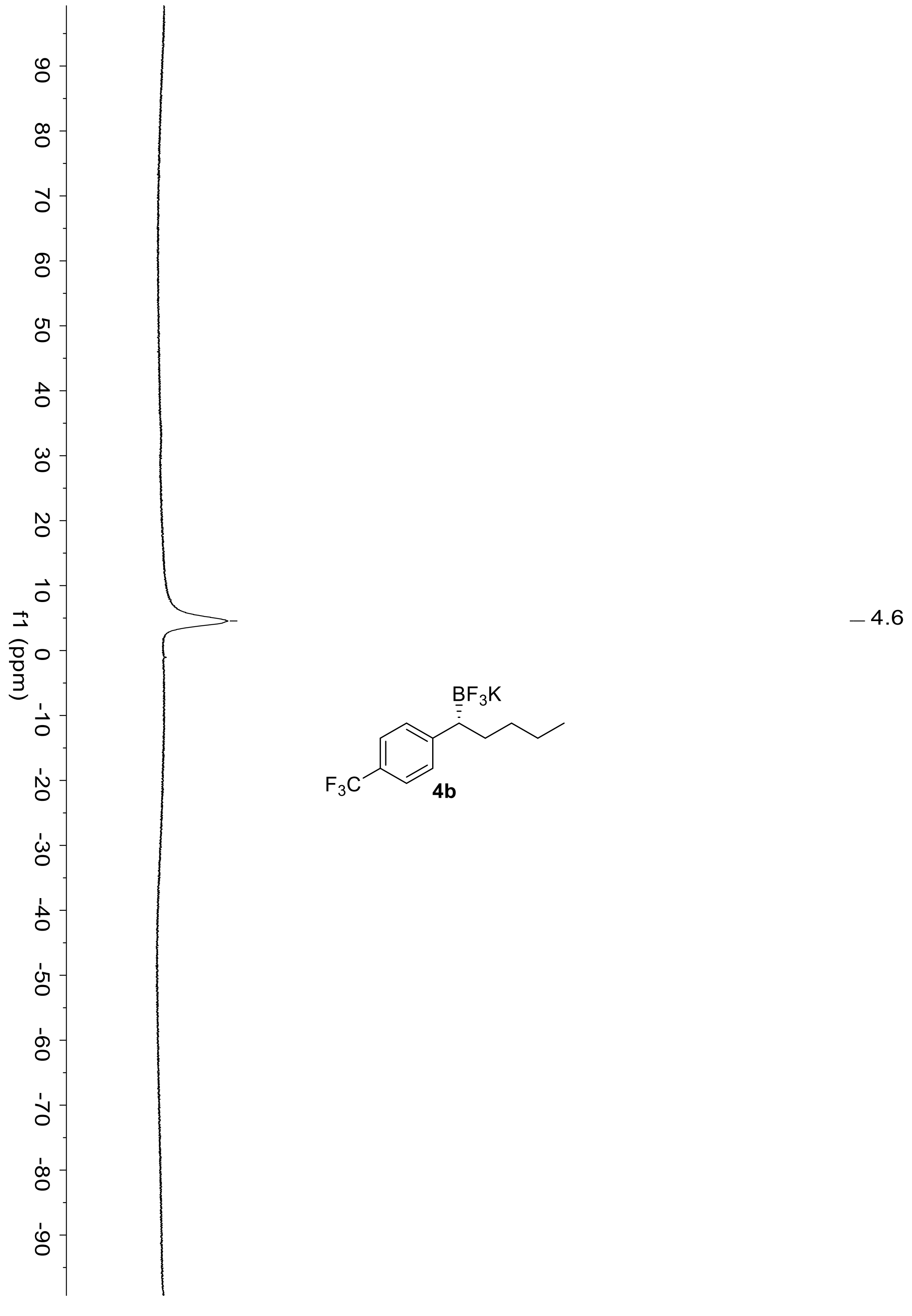
4.6
BF3K
F3C
4b
f1 (ppm)

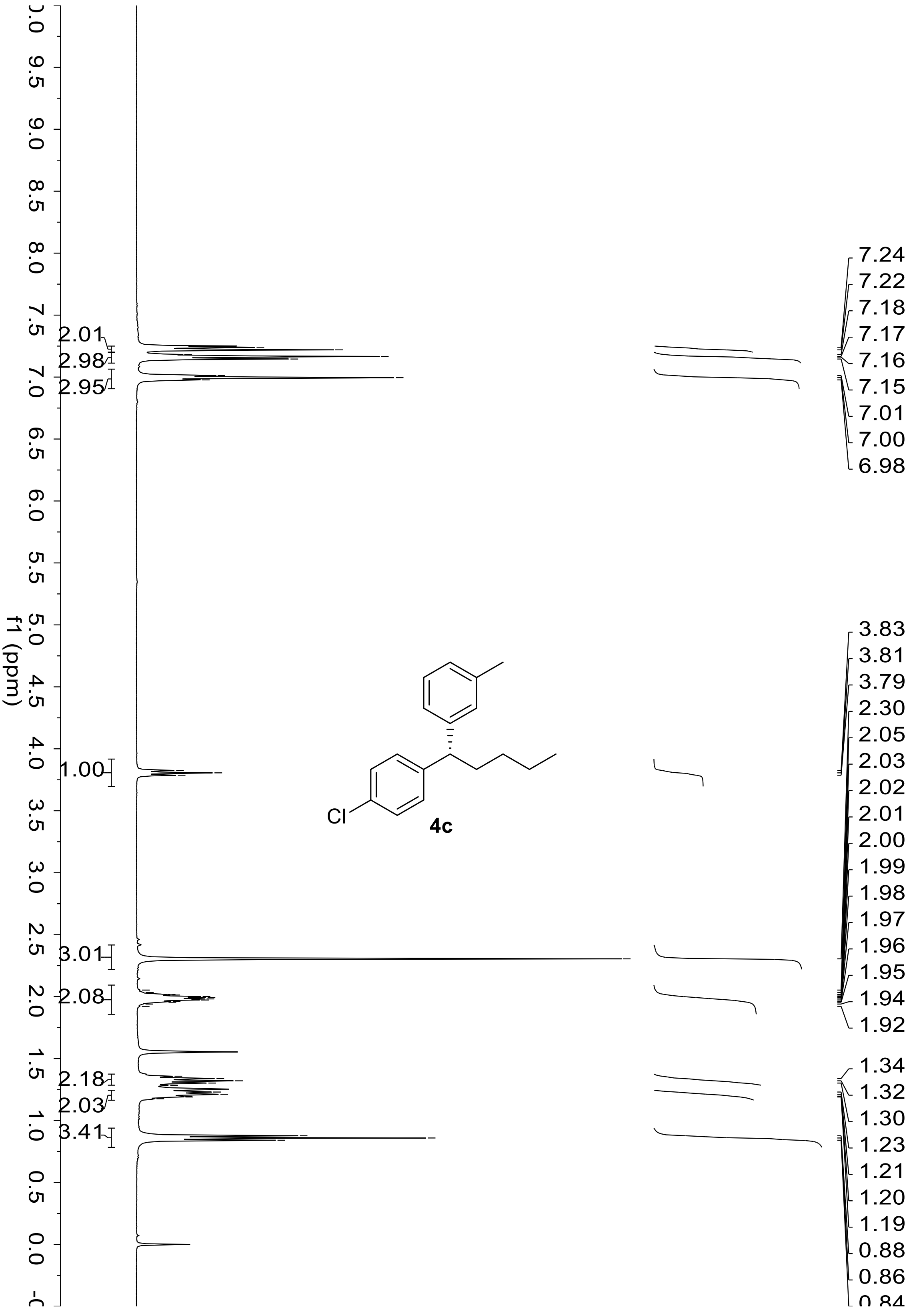
Cl
4c
f1 (ppm)
7.24
7.22
7.18
7.17
7.16
7.15
7.01
7.00
6.98
3.83
3.81
3.79
2.30
2.05
2.03
2.02
2.01
2.00
1.99
1.98
1.97
1.96
1.95
1.94
1.92
1.34
1.32
1.30
1.23
1.21
1.20
1.19
0.88
0.86
2.01
2.98
2.95
1.00
3.01
2.08
2.18
2.03
3.41

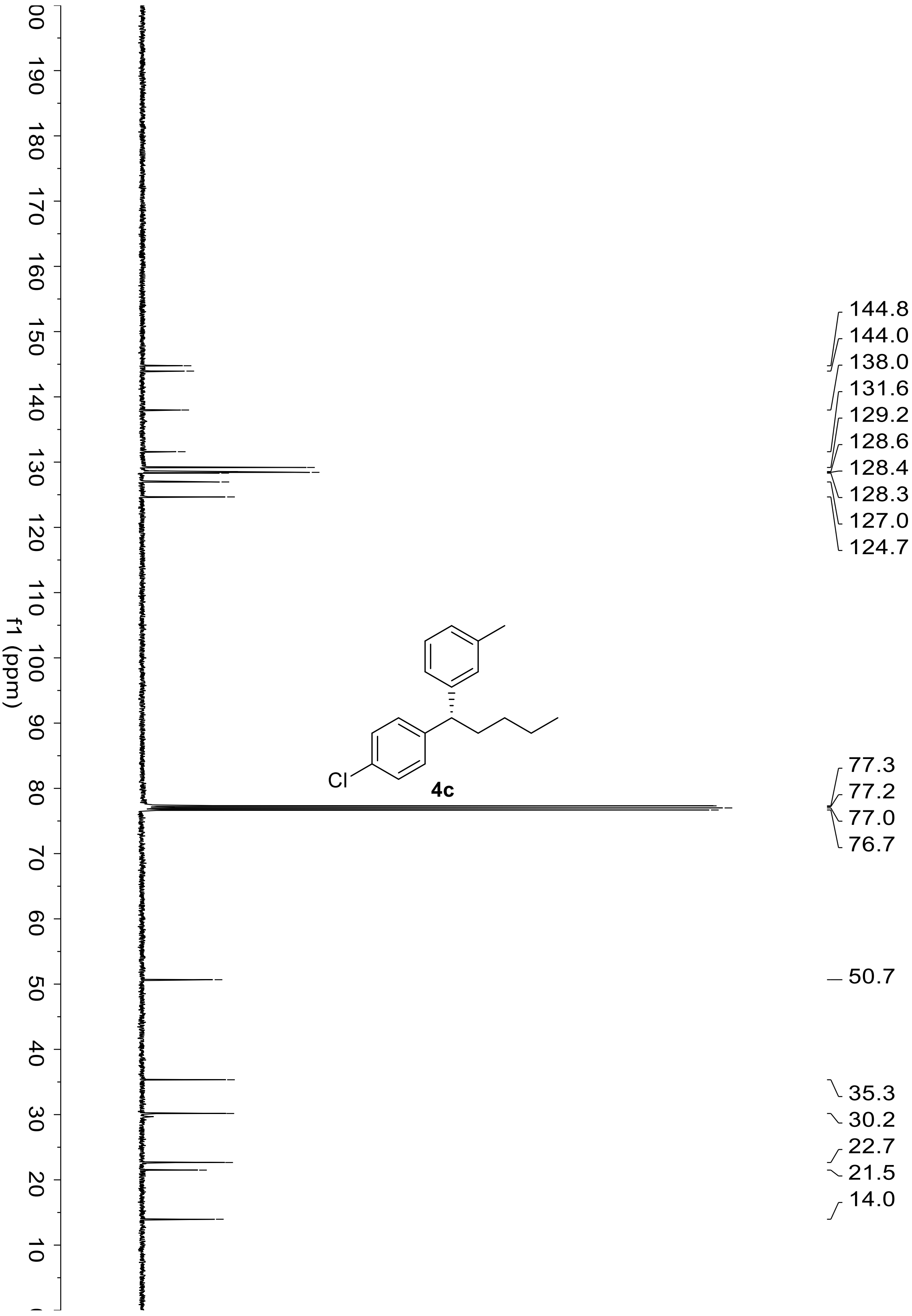
144.8
144.0
138.0
131.6
129.2
128.6
128.4
128.3
127.0
124.7
77.3
77.2
77.0
76.7
50.7
35.3
30.2
22.7
21.5
14.0
Cl
4c
f1 (ppm)

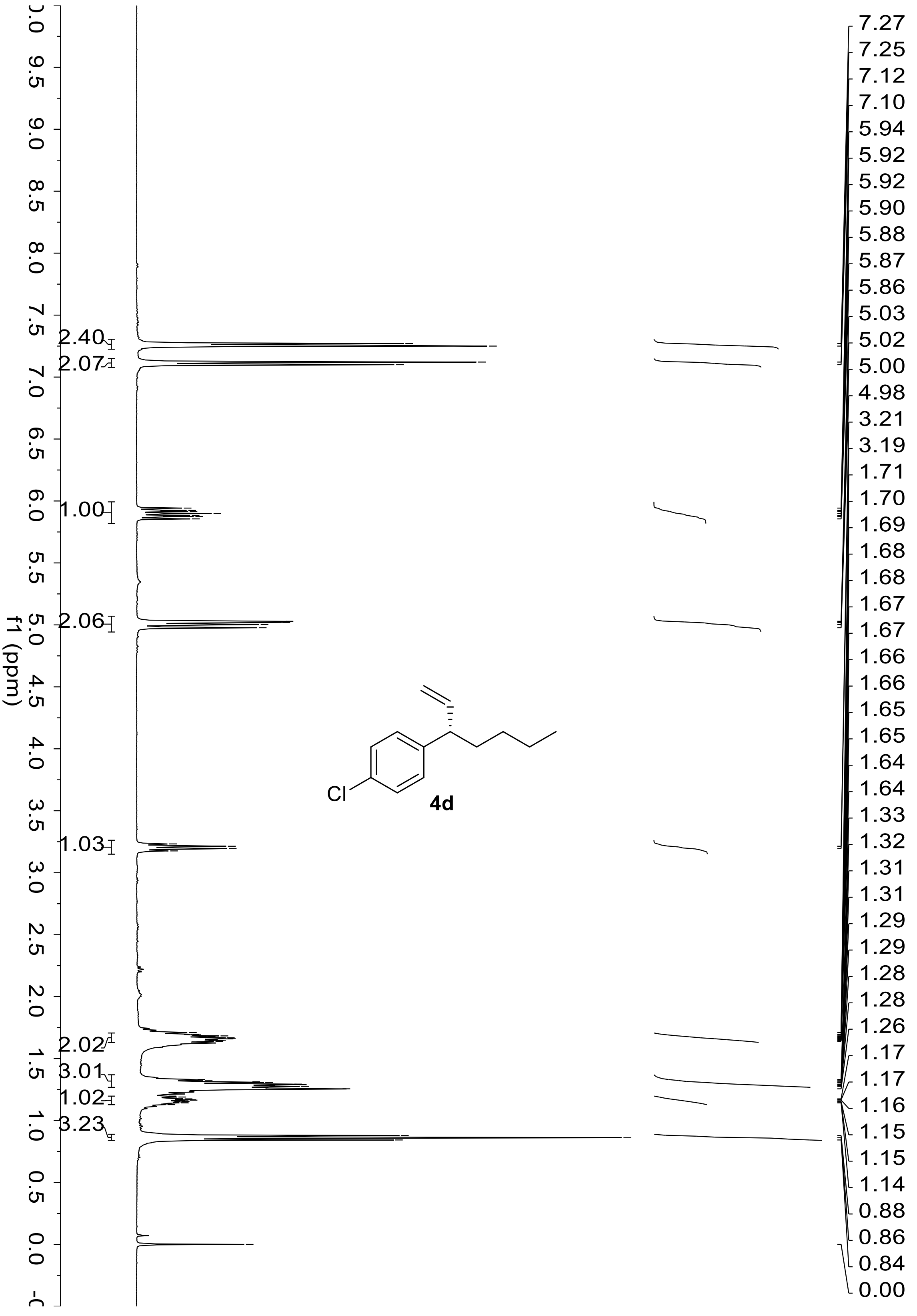
7.27
7.25
7.12
7.10
5.94
5.92
5.92
5.90
5.88
5.87
5.86
5.03
5.02
5.00
4.98
3.21
3.19
1.71
1.70
1.69
1.68
1.68
1.67
1.67
1.66
1.66
1.65
1.65
1.64
1.64
1.33
1.32
1.31
1.31
1.29
1.29
1.28
1.28
1.26
1.17
1.17
1.16
1.15
1.15
1.14
0.88
0.86
0.84
0.00
2.40
2.07
1.00
2.06
1.03
2.02
3.01
1.02
3.23
Cl
4d
f1 (ppm)

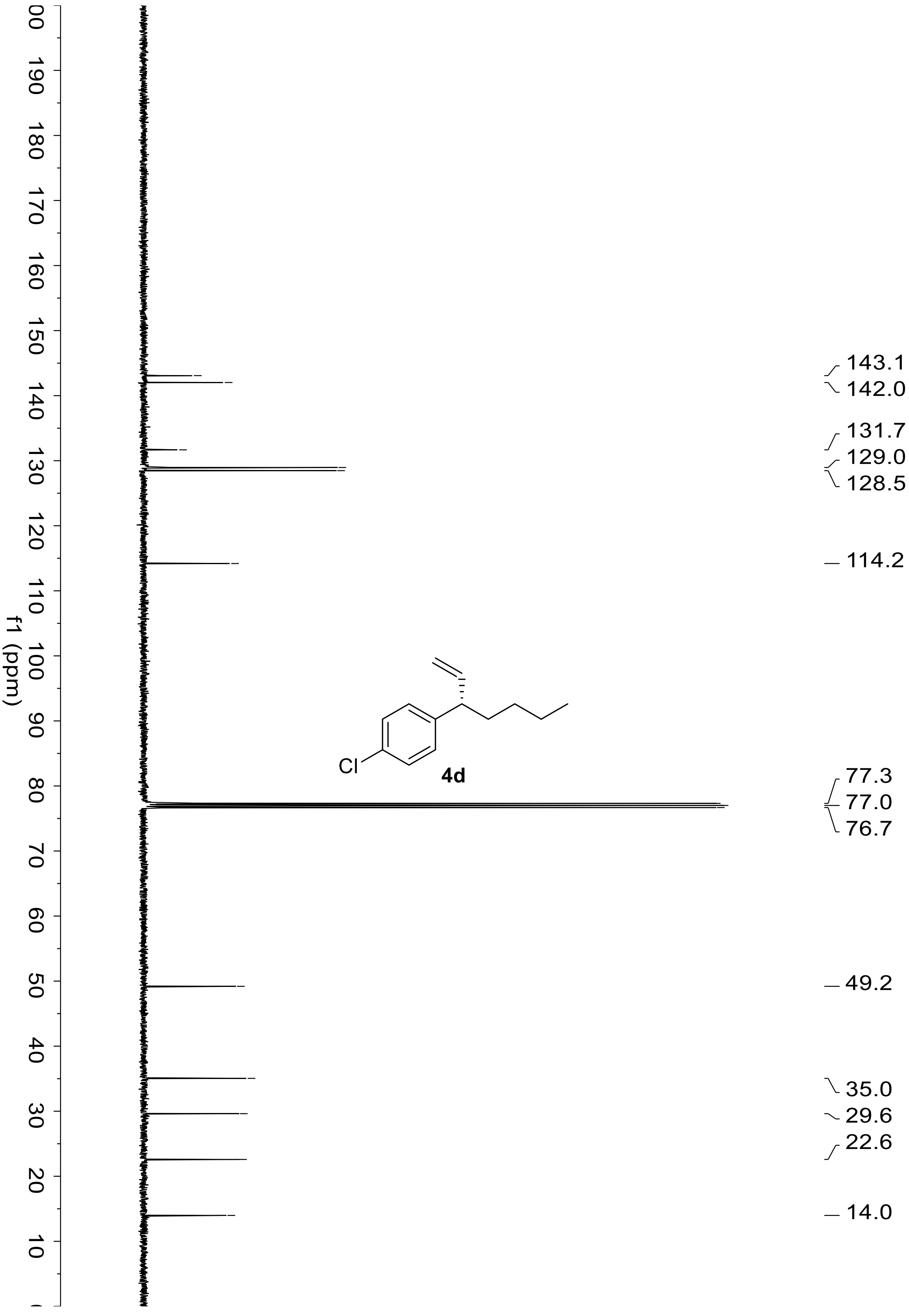
143.1
142.0
131.7
129.0
128.5
114.2
77.3
77.0
76.7
49.2
35.0
29.6
22.6
14.0
Cl
4d
f1 (ppm)

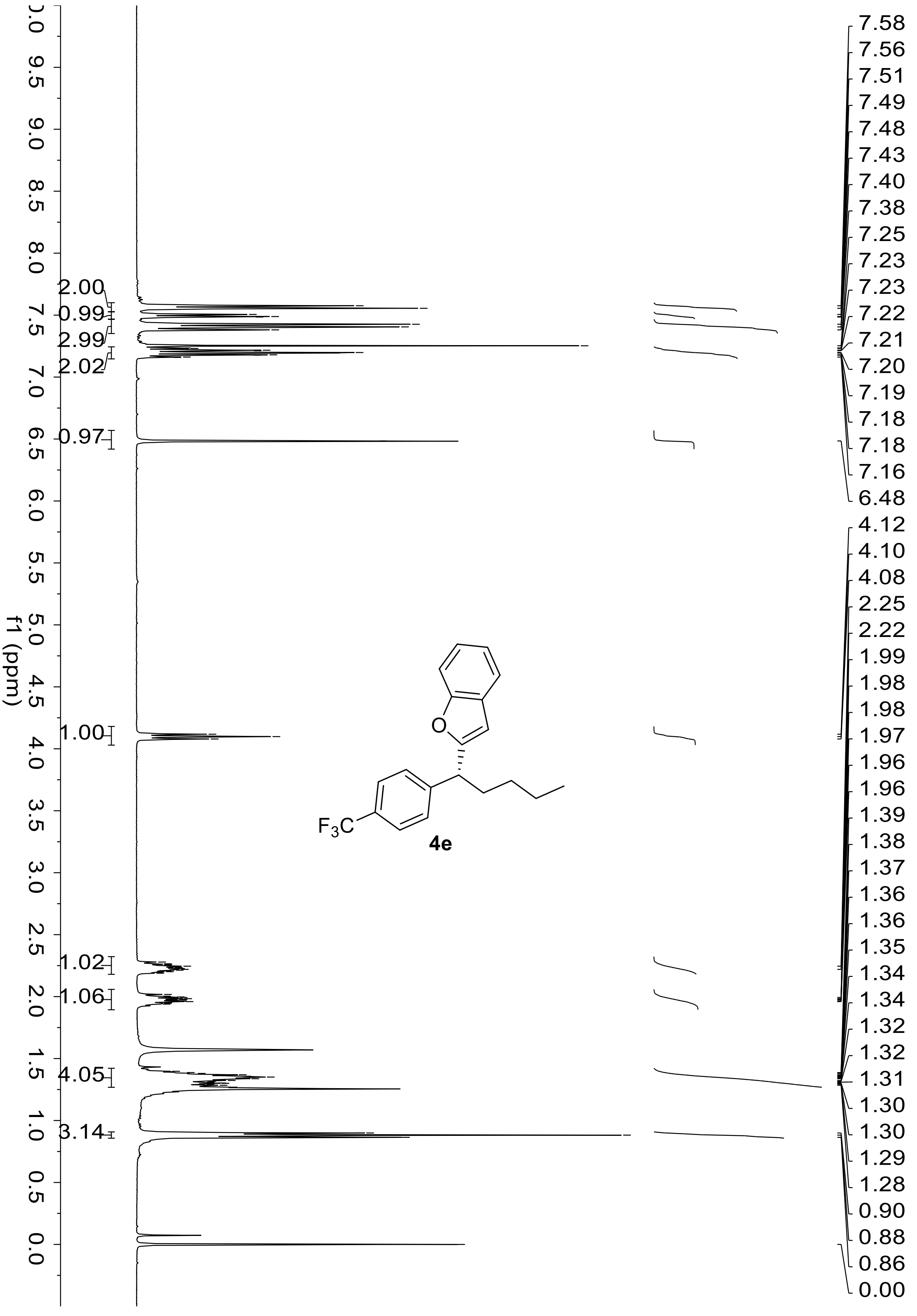
7.58
7.56
7.51
7.49
7.48
7.43
7.40
7.38
7.25
7.23
7.23
7.22
7.21
7.20
7.19
7.18
7.18
7.16
6.48
4.12
4.10
4.08
2.25
2.22
1.99
1.98
1.98
1.97
1.96
1.96
1.39
1.38
1.37
1.36
1.36
1.35
1.34
1.34
1.32
1.32
1.31
1.30
1.30
1.29
1.28
0.90
0.88
0.86
0.00
2.00
0.99
2.99
2.02
0.97
1.00
1.02
1.06
4.05
3.14
f1 (ppm)
F_3C
4e

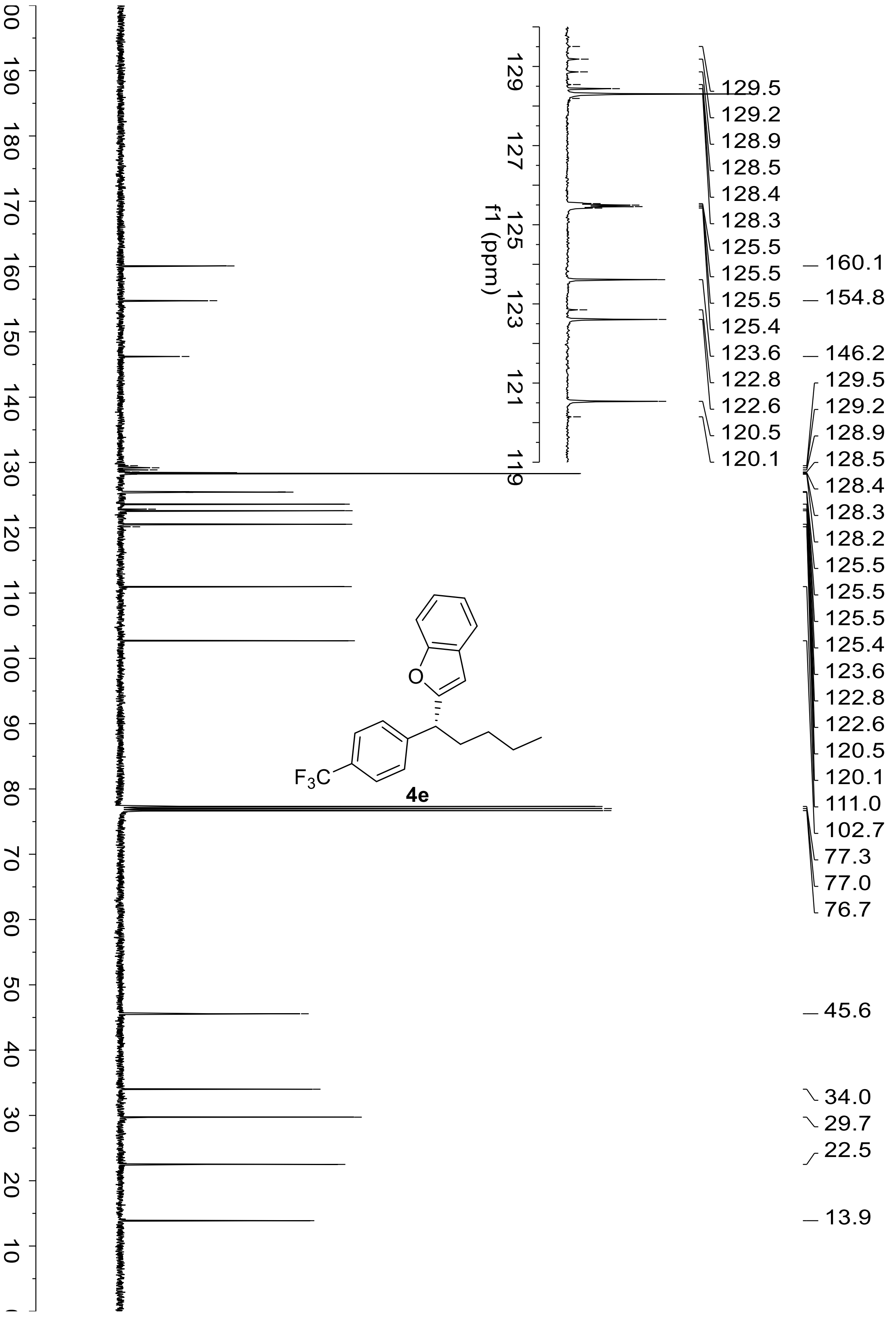
129.5
129.2
128.9
128.5
128.4
128.3
125.5
125.5
125.5
125.4
123.6
122.8
122.6
120.5
120.1
160.1
154.8
146.2
129.5
129.2
128.9
128.5
128.4
128.3
128.2
125.5
125.5
125.5
125.4
123.6
122.8
122.6
120.5
120.1
111.0
102.7
77.3
77.0
76.7
45.6
34.0
29.7
22.5
13.9
f1 (ppm)
f1 (ppm)
O
F3C
4e

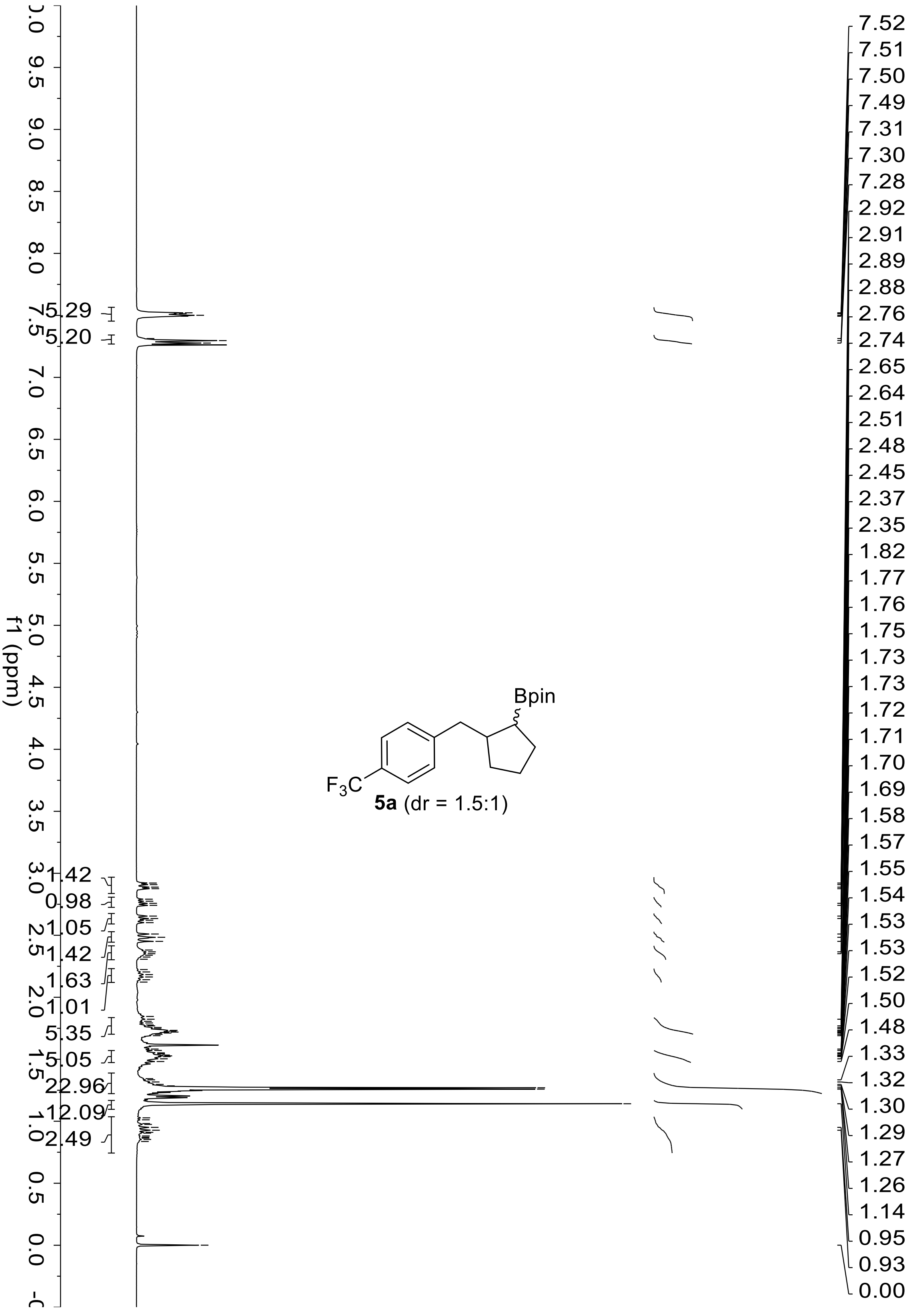
Bpin
F3C
5a (dr = 1.5:1)
f1 (ppm)
7.52 7.51 7.50 7.49 7.31 7.30 7.28 2.92 2.91 2.89 2.88 2.76 2.74 2.65 2.64 2.51 2.48 2.45 2.37 2.35 1.82 1.77 1.76 1.75 1.73 1.73 1.72 1.71 1.70 1.69 1.58 1.57 1.55 1.54 1.53 1.53 1.52 1.50 1.48 1.33 1.32 1.30 1.29 1.27 1.26 1.14 0.95 0.93 0.00

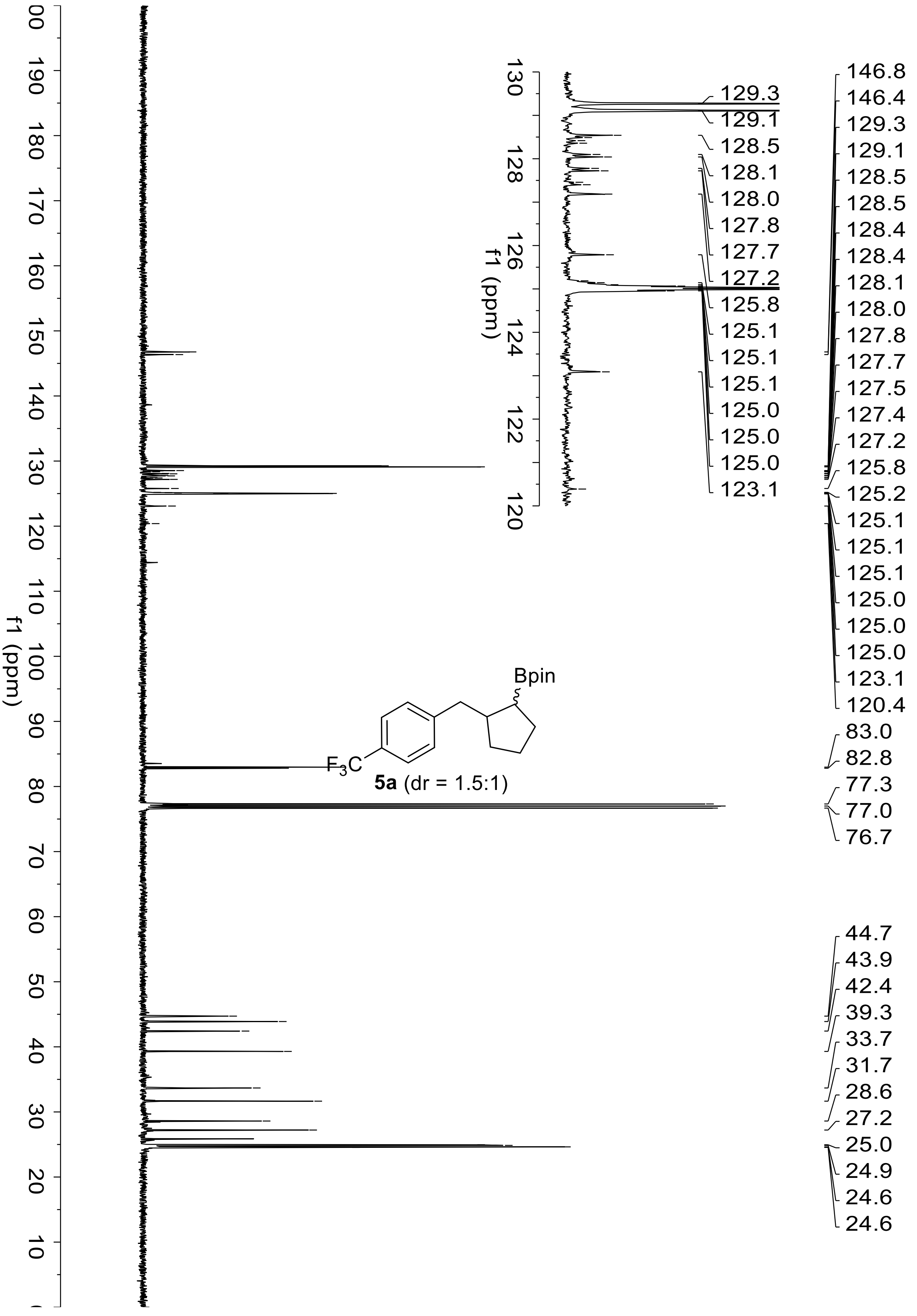

Bpin
F3C
5a (dr = 1.5:1)
f1 (ppm)
146.8
146.4
129.3
129.1
128.5
128.5
128.4
128.4
128.1
128.0
127.8
127.7
127.5
127.4
127.2
125.8
125.2
125.1
125.1
125.1
125.0
125.0
125.0
123.1
120.4
83.0
82.8
77.3
77.0
76.7
44.7
43.9
42.4
39.3
33.7
31.7
28.6
27.2
25.0
24.9
24.6
24.6

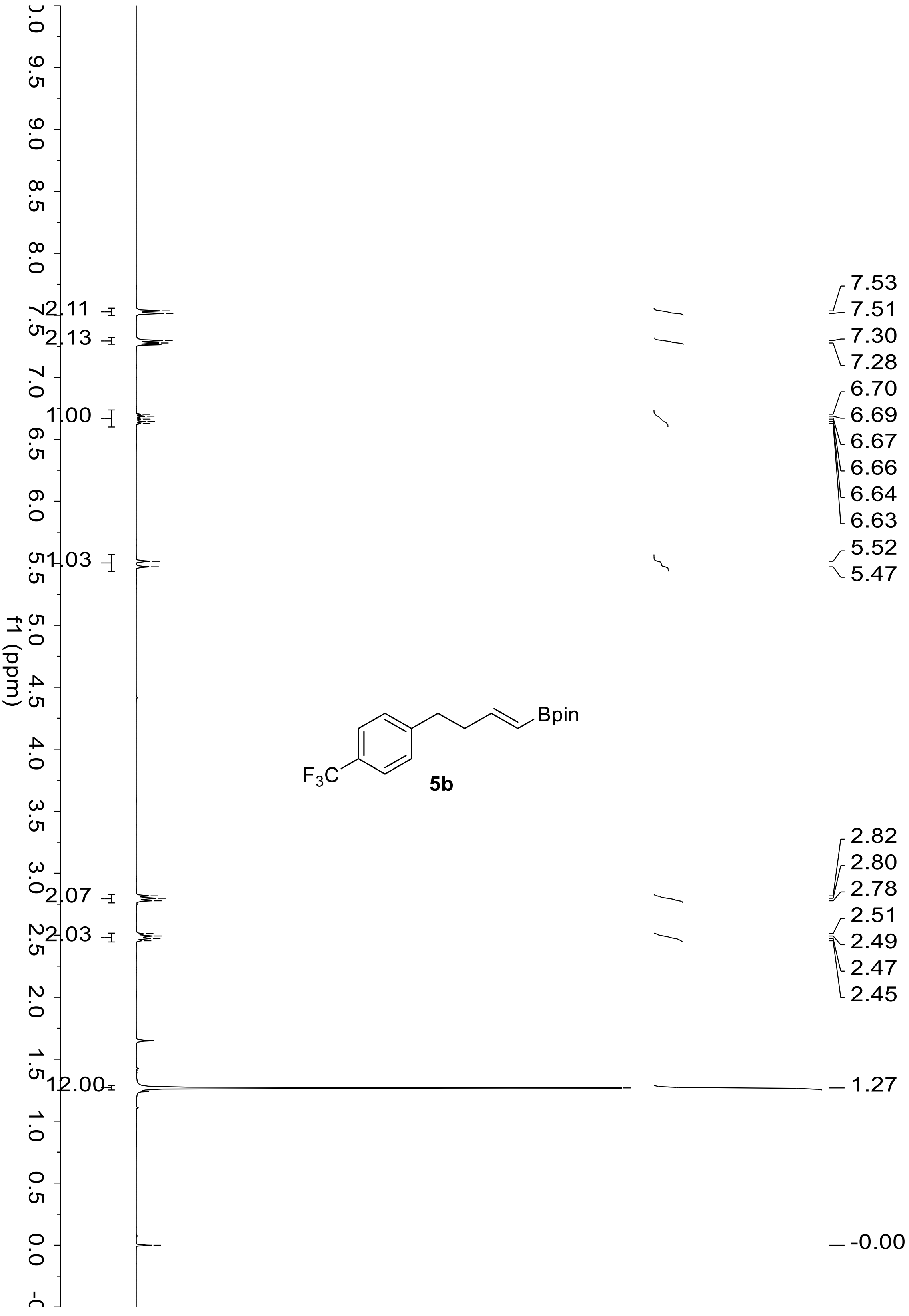

7.53
7.51
7.30
7.28
6.70
6.69
6.67
6.66
6.64
6.63
5.52
5.47
2.82
2.80
2.78
2.51
2.49
2.47
2.45
1.27
-0.00
2.11
2.13
1.00
1.03
2.07
2.03
12.00
f1 (ppm)
Bpin
F3C
5b

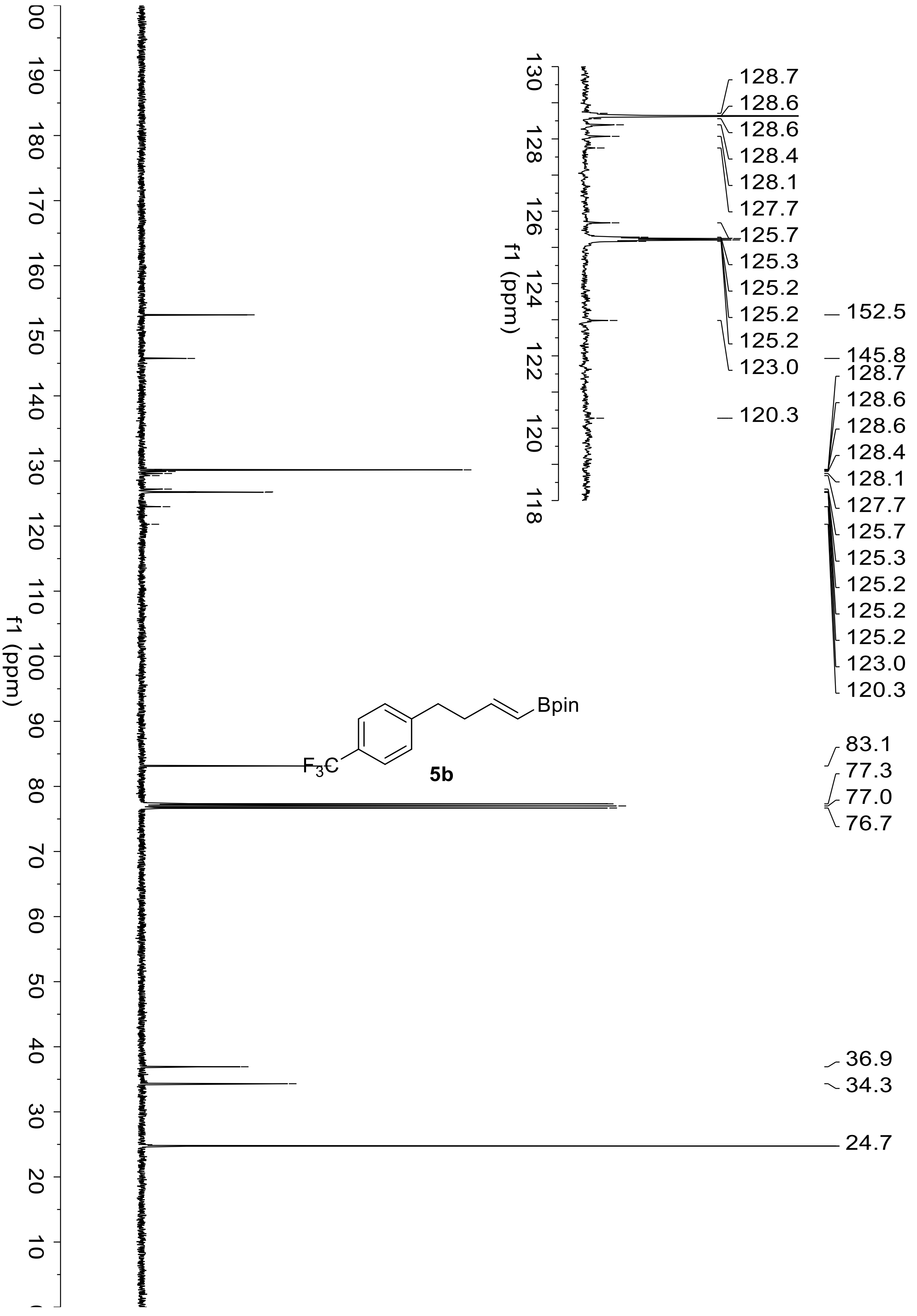
152.5
145.8
128.7
128.6
128.6
128.4
128.1
127.7
125.7
125.3
125.2
125.2
125.2
123.0
120.3
83.1
77.3
77.0
76.7
36.9
34.3
24.7
f1 (ppm)
Bpin
F3C
5b

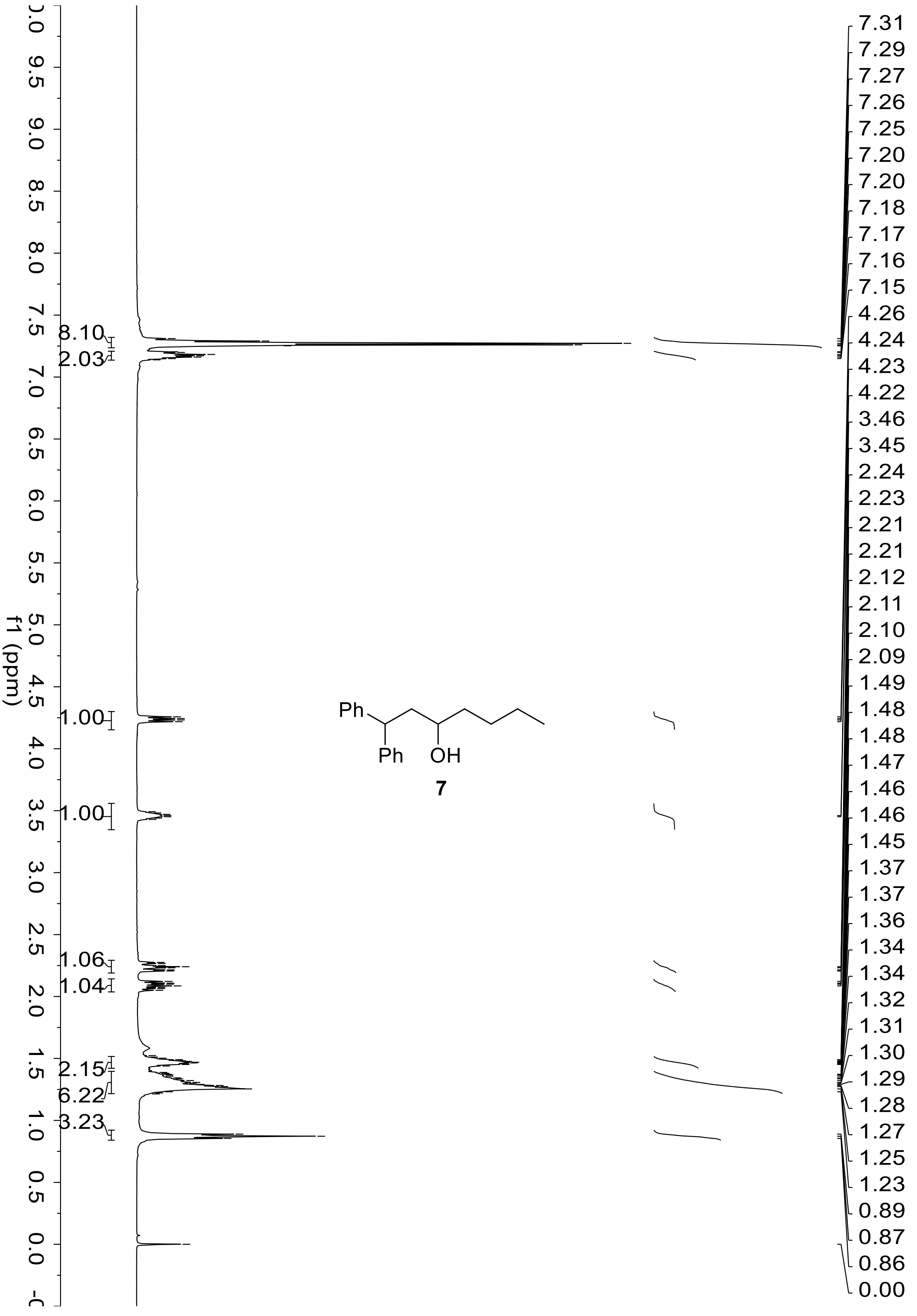
7.31
7.29
7.27
7.26
7.25
7.20
7.20
7.18
7.17
7.16
7.15
4.26
4.24
4.23
4.22
3.46
3.45
2.24
2.23
2.21
2.21
2.12
2.11
2.10
2.09
1.49
1.48
1.48
1.47
1.46
1.46
1.45
1.37
1.37
1.36
1.34
1.34
1.32
1.31
1.30
1.29
1.28
1.27
1.25
1.23
0.89
0.87
0.86
0.00
Ph
Ph
OH
7
8.10
2.03
1.00
1.00
1.06
1.04
2.15
6.22
3.23
f1 (ppm)

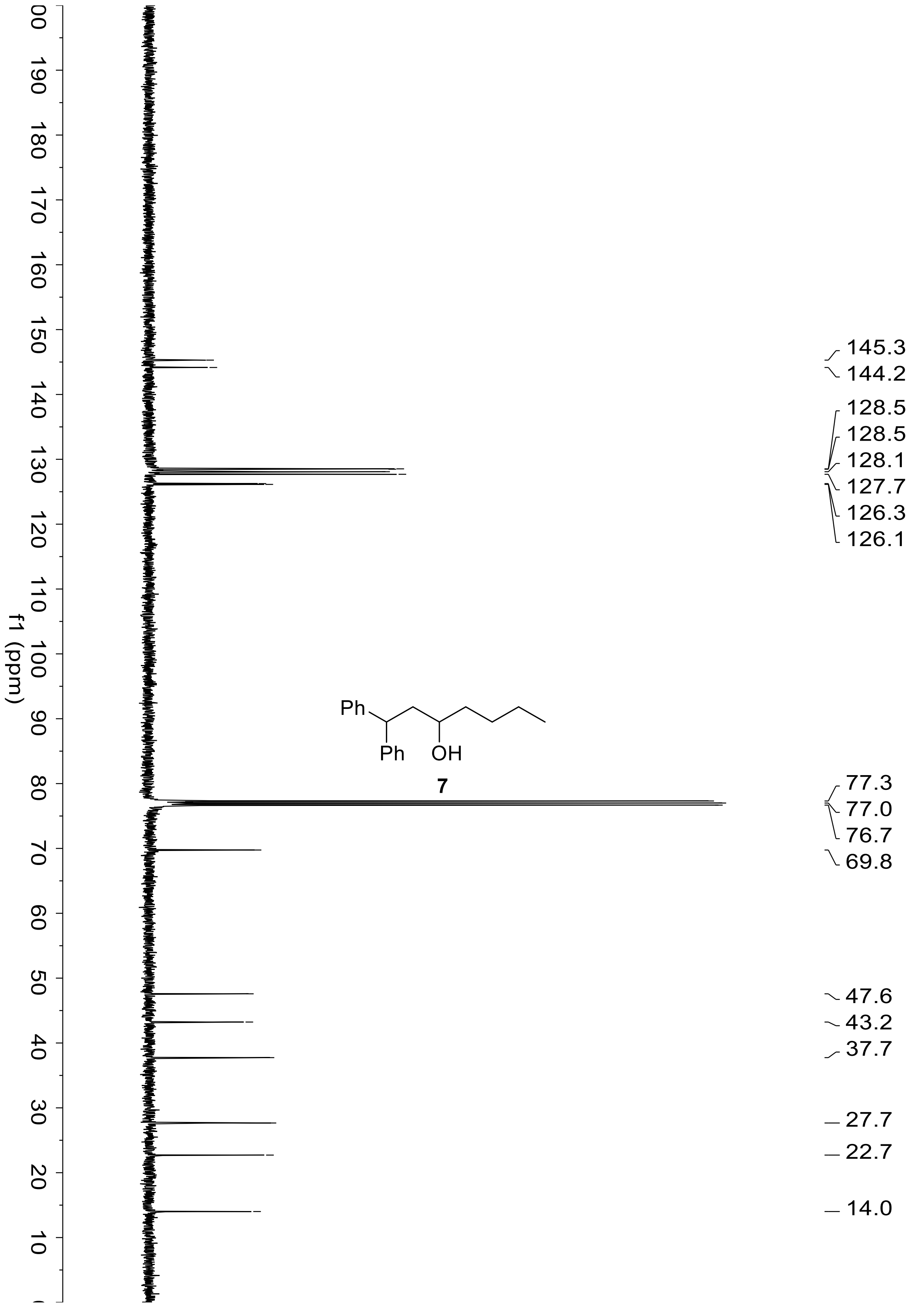
145.3
144.2
128.5
128.5
128.1
127.7
126.3
126.1
77.3
77.0
76.7
69.8
47.6
43.2
37.7
27.7
22.7
14.0
f1 (ppm)
Ph
Ph
OH
7

3. Supplementary References

S1. Sun, S-Z., Martin, R. Nickel-catalyzed umpolung arylation of ambiphilic α-bromoalkyl boronic esters. *Angew. Chem. Int. Ed.* **57**, 3622-3625 (2018).

S2. Schmidt, J., Choi, J., Liu, A., Slusarczyk, M., Fu, G. C. A general, modular method for the catalytic asymmetric synthesis of alkylboronate esters. *Science* **354**, 1265-1269 (2016).

S3. Petrone, D. A., Lischka, M., Lautens, M. Harnessing reversible oxidative addition: application of diiodinated aromatic compounds in the carboiodination process. *Angew. Chem. Int. Ed.* **52**, 10635-10638 (2013).

S4. Mukherjee, S., Patra, T., Glorius, F. Cooperative catalysis: a strategy to synthesize trifluoromethyl-thioesters from aldehydes. *ACS Catal.* **8**, 5842-5846 (2018).

S5. Xiong, W., Xu, G., Yu, X., Tang, W. P-chiral monophosphorus ligands for asymmetric copper-catalyzed allylic alkylation. *Organometallics* **38**, 4003-4013 (2019).

S6. Guo, J., Cheng, B., Shen, X., Lu, Z. Cobalt-catalyzed asymmetric sequential hydroboration/hydrogenation of internal alkynes. *J. Am. Chem. Soc.* **139**, 15316-15319 (2017).

S7. Hatano, M., Miyamoto, T., Ishihara, K. 3,3'-Diphosphoryl-1,1'-bi-2-naphthol-Zn(II) complexes as conjugate acid-base zatalysts for enantioselective dialkylzinc addition to aldehydes. *J. Org. Chem.* **71**, 6474-6484 (2006).

S8. Pérez, M., Fañanás-Mastral, M., Bos, P. H., Rudolph, A., Harutyunyan, S. R., Feringa, B. L. Catalytic asymmetric carbon–carbon bond formation via allylic alkylations with organolithium compounds. *Nature Chem.* **3**, 377-381 (2011).
